# An adaptable *in silico* ensemble model of the arachidonic acid cascade†

**DOI:** 10.1039/d3mo00187c

**Published:** 2024-06-03

**Authors:** Megan Uttley, Grace Horne, Areti Tsigkinopoulou, Francesco Del Carratore, Aliah Hawari, Magdalena Kiezel-Tsugunova, Alexandra C. Kendall, Janette Jones, David Messenger, Ranjit Kaur Bhogal, Rainer Breitling, Anna Nicolaou

**Affiliations:** a Laboratory for Lipidomics and Lipid Biology, Division of Pharmacy and Optometry, School of Health Sciences, Faculty of Biology Medicine and Health, Manchester Academic Health Science Centre, The University of Manchester Manchester UK anna.nicolaou@manchester.ac.uk; b Manchester Institute of Biotechnology, Faculty of Science and Engineering, The University of Manchester Manchester UK; c Department of Biochemistry, Cell and Systems Biology, Institute of Integrative, Systems and Molecular Biology, University of Liverpool Liverpool UK; d Unilever R&D Quarry Road East Bebington Wirral CH63 3JW UK; e Unilever R&D Colworth Science Park Sharnbrook Bedfordshire MK44 1LQ UK; f Lydia Becker Institute of Immunology and Inflammation, Faculty of Biology Medicine and Health, The University of Manchester Manchester UK

## Abstract

Eicosanoids are a family of bioactive lipids, including derivatives of the ubiquitous fatty acid arachidonic acid (AA). The intimate involvement of eicosanoids in inflammation motivates the development of predictive *in silico* models for a systems-level exploration of disease mechanisms, drug development and replacement of animal models. Using an ensemble modelling strategy, we developed a computational model of the AA cascade. This approach allows the visualisation of plausible and thermodynamically feasible predictions, overcoming the limitations of fixed-parameter modelling. A quality scoring method was developed to quantify the accuracy of ensemble predictions relative to experimental data, measuring the overall uncertainty of the process. Monte Carlo ensemble modelling was used to quantify the prediction confidence levels. Model applicability was demonstrated using mass spectrometry mediator lipidomics to measure eicosanoids produced by HaCaT epidermal keratinocytes and 46BR.1N dermal fibroblasts, treated with stimuli (calcium ionophore A23187), (ultraviolet radiation, adenosine triphosphate) and a cyclooxygenase inhibitor (indomethacin). Experimentation and predictions were in good qualitative agreement, demonstrating the ability of the model to be adapted to cell types exhibiting differences in AA release and enzyme concentration profiles. The quantitative agreement between experimental and predicted outputs could be improved by expanding network topology to include additional reactions. Overall, our approach generated an adaptable, tuneable ensemble model of the AA cascade that can be tailored to represent different cell types and demonstrated that the integration of *in silico* and *in vitro* methods can facilitate a greater understanding of complex biological networks such as the AA cascade.

## Abstract 2

Ensemble modelling approaches, which account for the uncertainty surrounding model parameters, were applied to the arachidonic acid cascade for the first time. The adaptable, tuneable model was tailored to represent different cell types and stimuli.

## Graphical abstract

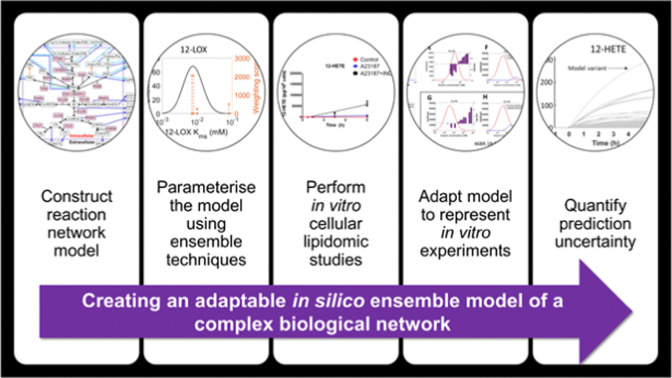

## 1. Introduction

Eicosanoids are a class of bioactive lipid mediators with hormone-like effects, known for their involvement in inflammation and immune reactions.^1–5^ They are derivatives of the 20-carbon (C-20) polyunsaturated fatty acid (PUFA) arachidonic acid (AA) and, to a lesser extent, the C-20 PUFAs dihomo-γ-linolenic acid (DGLA) and eicosapentaenoic acid (EPA). Eicosanoids control many physiological and pathophysiological processes; they are produced in response to cellular stressors and/or stimuli, are exported passively or *via* specific transporters, signal *via* dedicated G-protein coupled receptors, and can be enzymatically deactivated.^4–9^ This cascade of reactions allows cellular systems to control both the level and the activity of eicosanoids, offering a rapid response to inflammation and its resolution. Due to their involvement in various biological systems (*e.g.*, cardiovascular, renal, reproductive, nervous, ocular, skin) in both health and disease, the eicosanoids have been targeted for the development of pharmacological agents including the widely used non-steroidal anti-inflammatory drugs.^10–17^

The eicosanoid cascade begins with the release of AA (or another precursor C-20-PUFA) from membrane glycerophospholipids through the action of phospholipases (PL)A_2_—this reaction is the rate-limiting step of the pathway.^18,19^ The resulting free AA is rapidly metabolised by cyclooxygenase (COX), lipoxygenase (LOX) and cytochrome P450 monooxygenase (CYP450) isoforms, to form various eicosanoid classes including prostaglandins (PG), leukotrienes (LT), thromboxanes (TX), and a range of epoxy, mono-hydroxy and poly-hydroxy fatty acids.^4,5^ Some eicosanoids, mainly hydroxyeicosatetraenoic acid (HETE) species, are also found esterified in membrane glycerophospholipids.^20^ Overall, the prevalence and profiles of eicosanoids are cell and/or organ-specific, depending on the expression of the relevant biosynthetic enzymes.

Given the important role of eicosanoids in physiological and pathophysiological cellular responses, there is a strong interest in developing computational models that can be reliably used to assess hypotheses, predict adverse reactions, and support the development of novel therapeutics.^21–28^ Currently, the majority of such *in silico* metabolic models focus on the AA cascade, as AA is the most abundant cellular C20-PUFA and the bioactivities of its metabolites are best understood. Existing models have simulated the AA cascade in human polymorphonuclear leukocytes (PMNL), endothelial cells and platelets,^21,22^ and murine RAW 264.7 macrophages,^24,26,27^ and have been validated using experimental lipidomic and transcriptomic data.

Most of the existing mathematical models of the AA cascade are based upon the principles of continual adaptation and refinement against experimental data. This method enables a critical assessment of the similarity of the *in silico* simulation to its *in vitro* experimental counterpart, and allows for its iterative improvement and adaptation as new information is acquired. However, a common limitation of the approach is the lack of quantification of the uncertainty and confidence of the resulting predictions. Parameter fitting is often employed to overcome the issue of incomplete data availability, but this can impact predictive power as parameter values may be over-fitted in the computational model, resulting in model predictions that closely fit existing experimental data, but contain kinetic parameters of limited accuracy and fail to predict system behaviour in new conditions.^29^ Recent developments in ensemble modelling strategies, which allow a rigorous assessment of prediction confidence, can overcome these limitations.^30,31^ In contrast to other modelling strategies, ensemble modelling does not directly use individual parameter values from databases. Instead, all available parameter data is processed into probability distributions of plausible values; these distributions can then be sampled repeatedly to produce an ensemble of model variants with unique sets of parameter values. To date, the principles of uncertainty and ensemble modelling have not been applied to computational models of the AA cascade.

Here, we use a combination of *in silico* and *in vitro* approaches to implement the first predictive adaptable ensemble model for a generic AA cascade. This approach allows explicit quantification of the uncertainty of the modelling process and simulated predictions, which has not been addressed in the existing models of the AA cascade and facilitates greater model flexibility than the published methods. The benefits of this approach are demonstrated by adapting the metabolic model to represent HaCaT epidermal keratinocytes and 46BR.1N dermal fibroblasts, at baseline and following stimulation to mimic the release of AA and consequential biochemical responses. This versatile predictive model can be adapted and expanded in the future as a tool to investigate the behaviour of the AA cascade in various systems.

## 2. Materials and methods

### 2.1. Cell culture

HaCaT human epidermal keratinocytes (CLS Cell Lines Service GmbH; Eppelheim, Germany) were cultured in Dulbecco's modified Eagle medium (DMEM; Sigma; Dorset, UK) supplemented with 10% heat-inactivated foetal bovine serum (FBS; Sigma, UK). 46BR.1N human dermal fibroblasts (The European Collection of Cell Culture; Salisbury, UK) were cultured in minimum essential Eagle medium (MEM; Sigma, UK) supplemented with 15% FBS, l-glutamine, non-essential amino acids and sodium pyruvate (200 nM each; Sigma, UK). Cells were grown at 37 °C, 5% CO_2_ and 95% humidity; media were changed every 2–3 days; trypsin/EDTA (Sigma, UK) was used to detach the cells when needed.

### 2.2. Cell treatments

Cells were grown to 80% confluency and treated as follows. Calcium ionophore experiments: calcium ionophore A23187 (5 μM; Sigma, UK) and calcium chloride (1.8 mM; Sigma, UK) were added to cell-appropriate serum-free media, cells were incubated for the required amount of time (0.5, 1, 3 and 6 h). When appropriate, cells were pre-treated with the COX-inhibitor indomethacin (IND) (10 μM; Sigma, UK) for 1 h; they were then washed with phosphate buffered saline (PBS; Sigma, UK) and treated with A23187 in serum-free media, as described above. ATP experiments: cells were treated with ATP (2 mM; Sigma, UK) in serum free media for the required amount of time (0.5, 1, 3 and 6 h). Ultraviolet radiation (UVR) experiments: cells were grown in Petri dishes and were transferred to PBS prior to UV irradiation (15 mJ cm^−2^) using a Herbert Waldmann 236 B (UV6) lamp (Villingen-Schwenningen, Germany). The cells were then transferred to the appropriate serum-free media and incubated for the required amount of time (0.5, 1, 3 and 6 h). In all cases, conditioned media and cells were collected at the end of treatment and stored at –80 °C awaiting analysis.

### 2.3. UPLC/ESI-MS/MS analysis of eicosanoids

Eicosanoid production was measured in the cell culture media by mediator lipidomics, using ultra-high performance liquid chromatography coupled to tandem mass spectrometry with electrospray ionisation (UPLC/ESI-MS/MS), as described in ref. 32 and 33. In brief, cell culture media samples were mixed with ice-cold methanol to a final concentration of 15% methanol (v/v), internal standards were added (20 ng each; PGB_2_-d_4_ and 12-HETE-d_8_; Cayman Chemical; Michigan, USA) and the pH of the solution was adjusted to 3.0. Samples were then semi-purified by solid-phase extraction (C18-E 500 mg cartridges, 6 mL; Phenomenex; Macclesfield, UK), lipids eluted with methyl formate and dried under nitrogen. Eicosanoids (PGE_2_, 15-keto PGE_2_, PGF_2α_, 13,14-dihydro-15-keto PGE_2_, 12-HETE, 15-HETE) were analysed by ultraperformance liquid chromatography (UPLC) (Acquity pump; Waters; Wilmslow, UK) coupled to a triple quadrupole mass spectrometer with electrospray ionisation (ESI-MS/MS) (Xevo TQ-S; Waters, UK). Details on the multiple reaction monitoring transitions and other settings used, are provided in ref. 32. Results are reported as pg of eicosanoid/million cells.

### 2.4. Fatty acid analysis

Cellular lipids were extracted using chloroform : methanol (2 : 1 v/v; Fisher Scientific; Loughborough, UK) containing butylated hydroxytoluene (0.01% w/v; Fisher Scientific, UK). Fatty acids, including AA, were then trans-esterified into fatty acid methyl esters (FAME) using boron trifluoride in methanol and heneicosanoic acid (21:0; Sigma Aldrich; Gillingham, UK) as the internal standard. FAME were analysed by gas chromatography with flame ionisation detection (GC-FID), as previously described in ref. 34. Results are reported as ng of AA/million cells.

### 2.5. Western blot analysis

Cell pellets were lysed in radioimmunoprecipitation assay buffer (RIPA, Sigma Aldrich, UK) containing a protease inhibitor cocktail (Sigma Aldrich, UK). Sample protein content was determined using the DC Protein Assay Kit II (Sigma Aldrich, UK) and was used to normalise the cellular extracts. Protein extracts were reduced (Laemmli sample buffer; Sigma Aldrich, UK) and separated on sodium dodecyl sulfate (SDS) gels (10%; Bio-Rad, UK). Trans-blotting was performed using polyvinylidene difluoride (PVDF) membranes (Fisher Scientific, UK). The membranes were then blocked with powder milk solution (5% w/v) and incubated with primary monoclonal antibodies for COX-2 (1:50 000 dilution) (Cayman Chemicals, USA) and GAPDH (1:30 000 dilution) (AbCam; Cambridge, UK), followed by the secondary HRP-linked anti-mouse (1:1000 dilution) (GE Healthcare; Amersham, UK), and the anti-biotin molecular weight ladder HRP-linked antibody (1:2000 dilution) (Cell Signalling Technology; Leiden, Netherlands). Treated membranes were overlaid with enhanced chemiluminescence solution and developed using a ChemiDOC MP Imaging System (Blot/Chemi-Sensitivity mode). Images were taken using Image Lab 4.1 (Bio-Rad, UK); ImageJ software was used for densitometry. The relative expression of proteins was calculated by normalising band intensity against the GAPDH loading control.

### 2.6. Kinetic model

#### 2.6.1. Construction of the AA cascade metabolic model

The construction of the generic model was carried out using the biochemical system simulator COPASI version 4.21.166^35^ and MATLAB® R2016A (MathWorks). The AA cascade reaction network was defined on the basis of existing literature and described in terms of reaction rate laws and differential equations for transport events and metabolite concentration changes, respectively (eqn (1)–(8), Table 1, ESI,† Tables S1–S6 and S12–S16). These equations show the generic formula used to describe substrate release, protein induction, enzymatic reactions, non-enzymatic reactions, transport reactions and degradation in the model. Each reaction in the model has a unique reaction number (reaction (1)–(113); ESI,† Tables S1–S6). The parameterisation of each reaction is described in Supplementary Documents S7–S11 (ESI†), whereupon each reaction is provided a unique identifier which is found in the contents of each Supplementary Document (ESI†).

**Table 1:** Summary of network rate laws used for the construction of the AA cascade *in silico* model

Reaction type	Rate law	Equation number
Substrate release	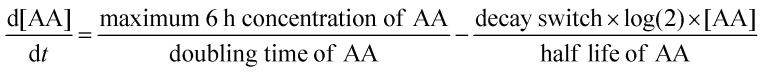 .	(1)
Protein induction switch	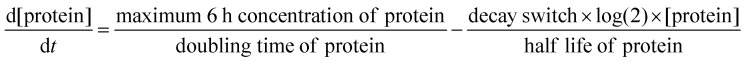	(2)
Enzymatic	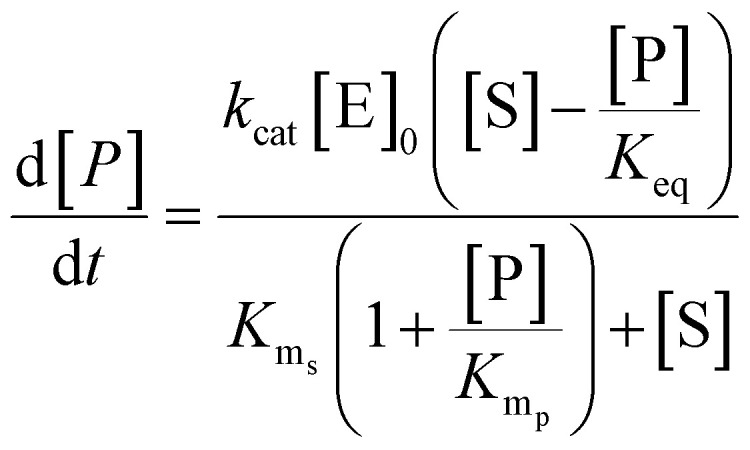	(3)
Non-enzymatic	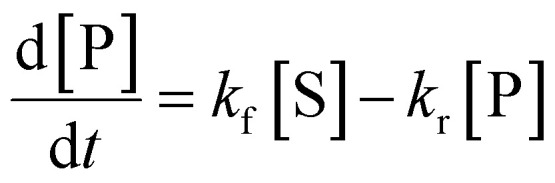	(4)
Transport	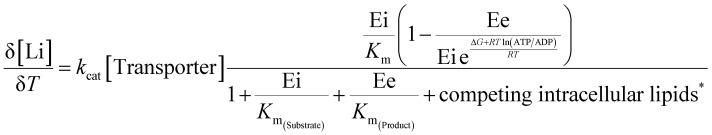	(5)
Competing intracellular lipids (*)	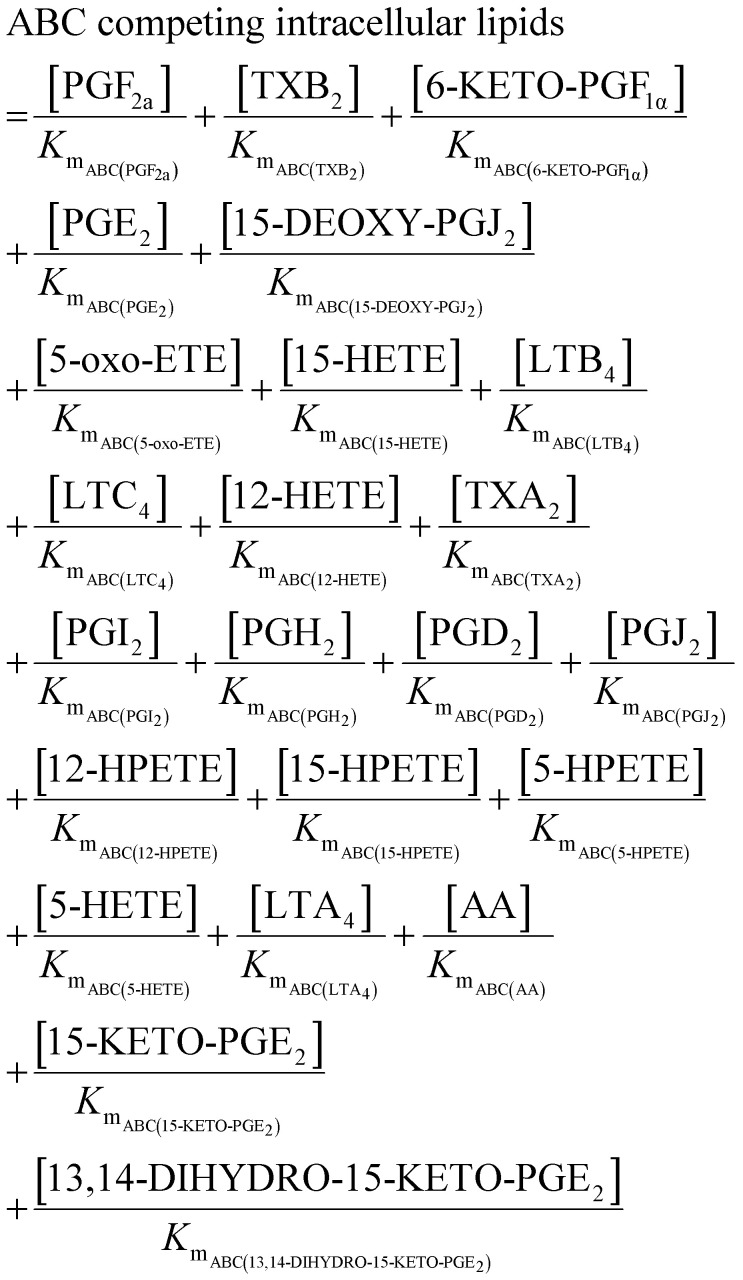	(6)
	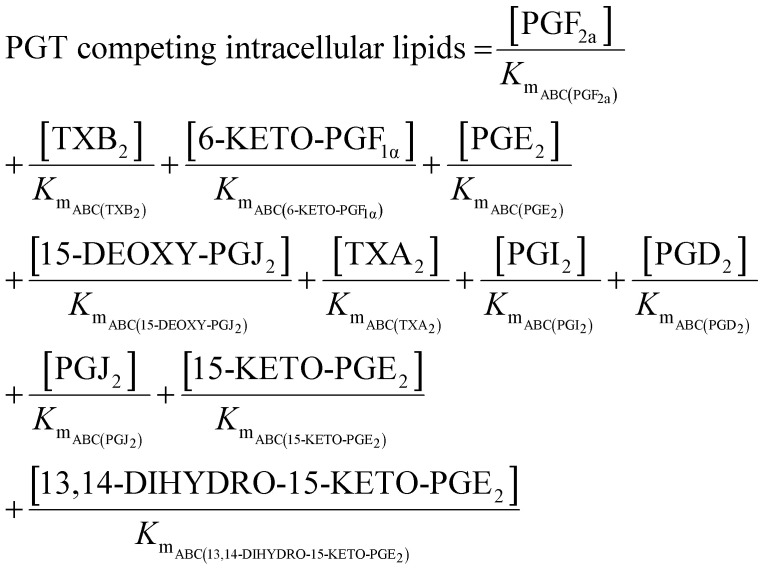	(7)
Degradation	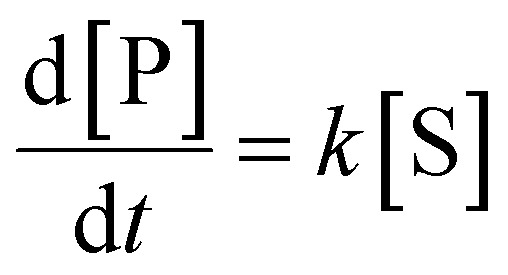	(8)

The initial metabolite concentrations were set as 10^−28^ mM, and simulations took place over 7 total simulated hours. The first simulated hour is an equilibration period to allow the system to reach steady state. Following this, an optional event occurs which mimics cell simulation, and the model runs for 6 further simulated hours. The optional event was controlled by adjusting the values of parameters in eqn (1) (Table 1) to alter the concentration of AA available to the model. The response of the model over the next 6 simulated hours was then recorded.

The parameters in eqn (1) (Table 1) represent different dynamics of AA release. For instance, maximum 6 h concentration of AA represents an unspecified source of AA from which intracellular AA is derived. The other parameters (Doubling Time of AA, Half Life of AA) were used to alter the rate at which AA became available upon stimulation and disappears afterwards, without assuming a specific mechanism underlying these dynamics. The Boolean parameter Decay Switch determines if the available AA concentration decreases subsequent to stimulation or not.

The enzyme abundances were assumed to remain constant for the duration of the 6 h simulated period after 1 h equilibration, representing the concentration of active enzymes. This assumption is a simplification of what is expected in the real biological systems, where enzyme concentrations are expected change in response to regulatory cascades activated by the stimulus. The intracellular volume was set as 1 pL and the extracellular volume to 1 nL, approximating the cell-to-media ratio in cell culture dishes.^36^

#### 2.6.2. Parameterisation of the AA cascade model

The differential equations describing the AA cascade were parameterised by assigning numerical values to the kinetic parameters, protein concentrations of all relevant enzymes and transporters, decay rates and physical constants contained within them. The same elementary Michaelis–Menten kinetics were assumed for all enzymes, as data justifying more complex kinetics was not available. Parameterisation was performed according to our recently published ensemble modelling protocol,^31^ and involved collecting literature values for parameters alongside information pertaining to experimental conditions. This standardised pipeline was then utilised to score and weight values in a semi-automated fashion. log-normal distributions of *k*_cat_, *K*_ms_, *K*_mp_, *K*_eq_, *k*_f_ and *k*_r_ values were defined from their corresponding weighted literature values to allow sampling of kinetic parameter and enzyme protein concentration values by Monte Carlo ensemble modelling.^37–39^ Each distribution was determined using weighted experimental data from multiple cell types reported in the protein database PaxDb,^40^ and represents the range of concentrations at which each enzyme has been detected.

An assumption of the model was that all chemical reactions (enzymatic and non-enzymatic) are theoretically reversible.^41^ To account for this, the terms for the Michaelis constant for the product (*K*_mp_) and reverse reaction rate constant (*k*_r_) were included in each kinetic equation. However, no values for the *K*_mp_ or *k*_r_ parameters could be found in the literature. Therefore, the pipeline developed by Tsigkinopoulou *et al.* (2018)^31^ was employed to generate thermodynamically consistent values for *K*_mp_ and *k*_r_, on the basis of the known (or estimated) equilibrium constant for each reaction. These parameters were assigned as the dependent parameters in enzymatic quadruplets or non-enzymatic triplets of kinetic parameters. Multivariate distributions were produced for each quadruplet or triplet, allowing a thermodynamically consistent value for the unknown parameters (*K*_mp_ and *k*_r_) to be calculated.

A comprehensive description of literature values underpinning the distributions of kinetic parameters (*k*_cat_, *K*_ms_, *K*_mp_, *K*_eq_, *k*_f_ and *k*_r_ values), enzyme and transporter protein concentrations, decay rates, physical constants (*e.g.* gas constant), model equations/reactions, weighting system and log-normal distributions are provided in the Supplementary Documents S7–S11 (ESI†) and project MediaWiki (http://www.systemsbiology.ls.manchester.ac.uk/wiki/index.php/Welcome_to_AA-Model-MediaWiki). Model scripts are also available at https://github.com/GS-Horne-UoM/Uttley_Adaptable_Model.

#### 2.6.3. Solving the AA cascade model

Ordinary differential equations (ODEs) (ESI,† Table S12) were solved using the ode15s solver in MATLAB® R2016A (MathWorks). Ensembles of model variants were generated by randomly sampling 1000 values from the log-normal probability distribution of each parameter. Each model variant was assigned one of the unique parameter sets. In order to account for the competitive inhibition of multiple species passing through the same transporter, a parameter was introduced into the ODEs for transport reactions. This term represents the affinity of each metabolite for the transporter using their individual *K*_m_ parameter.

#### 2.6.4. Adapting and quality scoring the model

To further adapt the model, the option to change protein expression was incorporated into the adaptable structure, as key enzymes of the eicosanoid cascade (*e.g.*, COX-2) are inducible, leading to protein concentration and enzymatic activity changes following exposure to various stimuli (eqn (2) – Table 1). This option of the model allows the user to change the concentration of any enzyme in the cascade by altering the value of protein induction parameters, at set time points during the simulation.

In order to adapt the enzyme abundance profile, the Kolmogorov–Smirnov (KS) test was used to detect significant differences between the parameter distributions in the most accurate members of the ensemble and distributions from which the parameters were originally sampled. The accuracy of model variant predictions was evaluated by comparing the concentration of metabolites in the model predictions to those of the *in vitro* experiments, and calculating a quality score as described by eqn (9). The quality score is based on the logarithm of the probability density function of a Gaussian distribution, thus yielding a weighted distance between the model prediction and the corresponding experimental data. Lower non-zero quality scores indicate a more accurate model variant.9
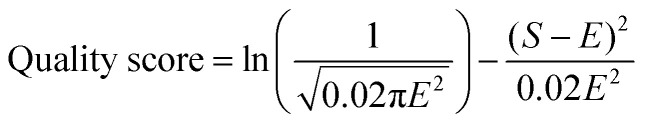
where *E* is the log of the experimental data point and *S* is the log of the simulation data.

To assess the overall quality of an ensemble of models in each *in silico* experiment, an additional ensemble-level quality score, the *Ψ* score, was introduced. This score represents the percentage of model variants predicting metabolite concentrations in a relatively close range to experimental data. Three types of *Ψ* scores were calculated, and the models were ranked accordingly. The scores were: (A) “Time point *Ψ*” score; this assesses the percentage of ensemble model variants predicting concentrations in relatively close range to individual data points (quality scores > −10), (B) “Metabolite *Ψ*” score; this assesses the percentage of ensemble model variants predicting concentrations in relatively close range to a series of data points for each metabolite (cumulative quality score > −40), (C) “Total *Ψ*” score; this assesses the percentage of ensemble model variants predicting metabolite concentrations that are relatively close to all data points (cumulative quality score > −500) (ESI,† Table S16).

#### 2.6.5. Statistical analysis of quality scores relative to experimental data

For each parameter in the model, a one-sample KS test was used to test for statistically significant differences between the distribution of the parameter values in the top ten percent best-fitting models, compared with the originally-sampled parameter distribution. A Bonferroni correction was applied to compensate for multiple testing and control the family-wise error rate at 1%, considering a total number of hypotheses of 184 (*i.e.*, total number of parameters).

## 3. Results and discussion

Here we present the first mathematical ensemble model of AA metabolism, which explicitly quantifies prediction uncertainty by employing a Monte Carlo ensemble modelling method to acknowledge parameter uncertainty.^30,37–39^ Experimentally, six eicosanoid species were detected above the limit of quantification of the UPLC-MS/MS assay (Section 2.3), in HaCaT keratinocytes (PGE_2_, 15-keto PGE_2_, PGF_2α_, 13,14-dihydro-15-keto PGE_2_, 12-HETE, 15-HETE), whilst three species were detected above the limit of quantification in 46BR.1N fibroblasts (PGE_2_, 12-HETE, 15-HETE). Thus, we explore the experimental and computational data for products of COX, 12-LOX and 15-LOX as no experimental data was available for leukotrienes. Subsequently, we developed a novel quality scoring method to quantitatively investigate the proximity between an ensemble of *in silico* model variants and *in vitro* data. This facilitated model adaptation by inferring the probable enzyme concentrations of the eicosanoid reaction network (*e.g.*, COX-1, COX-2, 12-LOX, 15-LOX) in HaCaT keratinocytes and 46BR.1N fibroblasts.

### 3.1. Creating a model of the AA reaction network

**Fig. 1:**
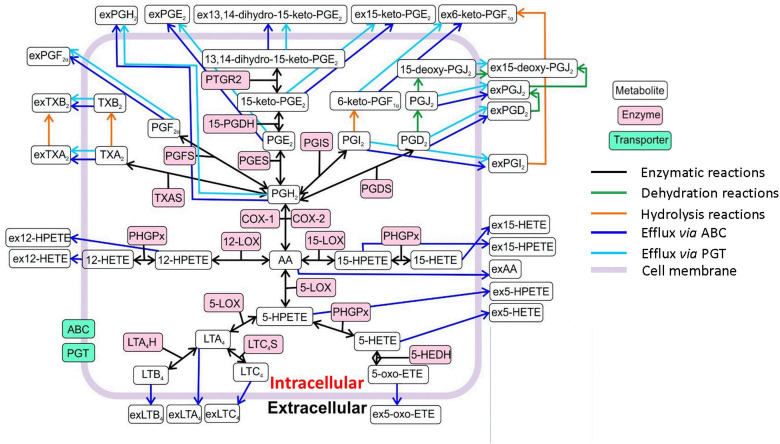
Overview of the reaction network of the *in silico* model of the AA cascade. The model includes intracellular production by enzymatic and non-enzymatic reactions (oxygenation; dehydration; hydrolysis), efflux to the extracellular compartment (ABC transporters) and prostaglandin transporter (PGT) mediated reactions. Abbreviations: arachidonic acid (AA), extracellular prostaglandin H_2_ (exPGH_2_), extracellular prostaglandin E_2_ (exPGE_2_), extracellular 13,14-dihydro-15-keto-prostalgandin E_2_ (ex13,14-dihydro-15-keto-PGE_2_), extracellular 15-keto-prostalgandin E_2_ (ex15-keto-PGE_2_), extracellular 6-keto-prostaglandin F_1α_ (ex6-keto-PGF_1α_), extracellular prostaglandin F_1α_ (exPGF_1α_), 13,14-dihydro-15-keto-prostaglandin E_2_ (13,14-dihydro-15-keto-PGE_2_), 15-deoxy-prostaglandin J_2_ (15-deoxy-PGJ_2_), extracellular 15-deoxy-prostaglandin J_2_ (ex15-deoxy-PGJ_2_), extracellular thromboxane B_2_ (exTXB_2_), thromboxane B_2_ (TXB_2_), prostaglandin reductase 2 (PTGR2), prostaglandin F_2α_ (PGF_2α_), 15-keto-prostaglandin E_2_ (15-keto-PGE2), 6-keto-PGF_1α_ (6-keto-PGF_1α_), prostaglandin J_2_ (PGJ_2_), extracellular prostaglandin J_2_ (exPGJ_2_), extracellular prostaglandin D_2_ (exPGD_2_), 15-prostaglandin dehydrogenase (15-PGDH), extracellular thromboxane A_2_ (exTXA_2_), thromboxane A_2_ (TXA_2_), prostaglandin F synthase (PGFS), prostaglandin E synthase (PGES), prostaglandin E_2_ (PGE_2_), prostaglandin I synthase (PGIS), prostaglandin I_2_ (PGI_2_), prostaglandin D_2_ (PGD_2_), extracellular prostaglandin I_2_ (exPGI_2_), thromboxane A synthase (TXAS), prostaglandin H_2_ (PGH_2_), prostaglandin D synthase (PGDS), cyclooxygenase-1 (COX-1), cyclooxygenase-2 (COX-2), extracellular 12-hydroperoxy-eicosatetraenoic acid (ex12-HPETE), phospholipid hydroperoxide glutathione peroxidase (PHGPx), 12-lipoxygenase (12-LOX), 15-lipoxygenase (15-LOX), extracellular 15-hydroxy-eicosatetraenoic acid (ex15-HETE), extracellular 15-hydroperoxy-eicosatetraenoic acid (ex15-HPETE), extracellular 12-hydroxy-eicosatetraenoic acid (ex12-HETE), 12-hydroperoxy-eicosatetraenoic acid (12-HPETE), 15-hydroxy-eicosatetraenoic acid (15-HETE), extracellular arachidonic acid (ex-AA), 5-lipoxygenase (5-LOX), 5-hydroperoxy-eicosatetraenoic acid (5-HPETE), extracellular 5-hydroperoxy-eicosatetraenoic (ex5-HPETE), extracellular 5-hydroxy-eicosatetraenoic acid (ex5-HETE), 5-hydroxy-eicosatetraenoic acid (5-HETE), 5-hydroxyeicosanoid dehydrogenase (5-HEDH), 5-oxo-eicosatetraenoic acid (5-oxo-ETE), extracellular 5-oxo-eicosatetraenoic acid (ex5-oxo-ETE), leukotriene A_4_ (LTA_4_), leukotriene C_4_ synthase (LTC_4_S), leukotriene A_4_ hydrolase (LTA_4_H), leukotriene B_4_ (LTB_4_), leukotriene C_4_ (LTC_4_), extracellular leukotriene B_4_ (exLTB_4_), extracellular leukotriene A_4_ (exLTA_4_), extracellular leukotriene C_4_ (exLTC_4_), ATP-binding cassette transporter (ABC), prostaglandin transporter (PGT).

Our ensemble model of the AA cascade includes 113 reactions and 49 metabolites; thus, it provides a more comprehensive view of the AA cascade compared with the currently published models^21,22,24,26,27^ (Fig. 1 and ESI,† Tables S1–S6, S12–S16, Supplementary Documents S7–S11). The network includes key lipid mediators as well as enzymatic, non-enzymatic and transport reactions taking place in the intracellular and extracellular compartments of a generic cell. To reduce the complexity of the model, we opted to disregard feedback mechanisms that are included in some previously-published AA cascade models.^21,27,28^ The metabolism of AA *via* CYP, hydrolysis of glycerophospholipid-esterified HETE, and formation of cysteinyl leukotrienes and related feedback loops were not included either. These reactions could be included in future iterations of our model, as it becomes adapted to a wider range of cell types including immune cells. Our model also excludes the processes of transcription and translation, which are considered to occur at a much slower timescale than the dynamics we simulate.^42,43^ Future iterations of the model could include these processes to allow application to a wider range of scenarios.

PLA_2_-mediated AA release acts as a trigger for eicosanoid cascade activation. As this process involves multiple isoforms and glycerophospholipid substrates that are beyond the scope of this model,^18,19^ we instead simulated the initiation of the AA cascade based upon enzymatic release of AA (reaction (1), ESI,† Table S1), non-enzymatic accumulation of AA (reaction (95); Table 1, ESI,† Table S1 and Supplementary Document S.11.1.) and the release of AA from membrane-bound sources *via* a non-enzymatic route (reaction (113); ESI,† Table S1, Supplementary Document S.11.3.). Furthermore, each reaction can be turned on/off by changing the value of its parameters (*k*_cat_, *K*_ms_, *K*_mp_, *K*_eq_ and [Enzyme]_0_). In the applications described here, reaction (95) (ESI,† Table S1 and Supplementary Document S.11.1.); represented in eqn (1) was turned on, leading to a sudden release of AA into the system, whilst reactions (1) and (113) were turned off. The simplified dynamics of AA release in Reaction (95) were manually defined to match the observed dynamics of AA (ESI,† Table S1 and Supplementary Document S.11.1.). This mechanism ensured that metabolite concentration matched our *in vitro* observations and allowed multiple AA release dynamics to be explored during model refinement.

### 3.2. Modelling parameterisation uncertainty

Parameterisation using an ensemble modelling pipeline allowed for standardised and semi-automated scoring of published data to produce log-normal distributions of *k*_cat_, *K*_ms_, *K*_mp_, *K*_eq_, *k*_f_ and *k*_r_ and [Enzyme]_0_ in the network.^31^ Ensemble parameterisation also enables the incorporation of literature data from multiple sources (*e.g.*, PaxDB, BRENDA, MetaCyc),^44–46^ and the subsequent supplementation of parameters should new data become available. In some cases, where incomplete sets of kinetic information were obtained, parameter distributions were produced from the most analogous information available (*e.g.*, 5-LOX and 5-LOX/5-LOX activating protein (5-LOX/FLAP) parameter distributions for *K*_ms_ and *k*_cat_ were produced from the same set of literature values) (ESI,† Tables S7–S11). As PaxDB does not report exact enzyme abundance values, future proteomic studies would enable further optimisations leading to more accurate models.

**Fig. 2:**
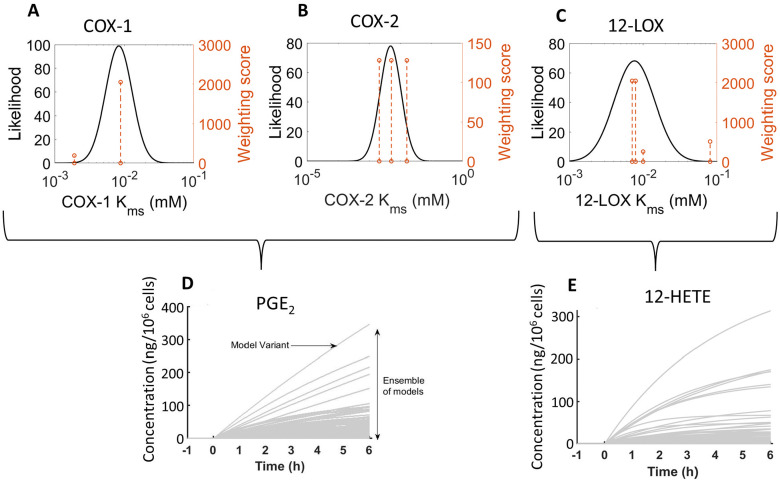
log-normal distribution of COX-1^48,49^ (A), COX-2^50–52^ (B), and 12-LOX^53–56^ (C) *K*_ms_ values and resultant predictions of PGE_2_ (D) and 12-HETE (E) concentration by an ensemble model (1000 model variants). The dashed orange line represents published values and the height corresponds to the weighting score from the parameter estimation pipeline.^31^ The source of this demonstration data was the HaCaT model of indomethacin (IND) + calcium ionophore (A23187) stimulated *in silico* model (“HaCaT + IND + A23187”).

As an example of our approach, we demonstrate how we used the *K*_ms_ literature values for COX-1, COX-2, and 12-LOX, to produce their likelihood weighting (Fig. 2(A)–(C)). Upon initiation, we randomly sampled the log-normal distributions of all kinetic parameters, to generate ensembles of 1000 model variants with unique parameter values. Each model variant produced a single set of predictions of metabolite concentrations over 6 h, resulting in a range of simulated behaviours for the concentration of the respective reaction product (*e.g.*, PGE_2_ and 12-HETE, respectively, Fig. 2(D) and (E)). In comparison, published *in silico* models of the AA cascade rely on single literature value parameters or parameter fitting, which may poorly describe the modelled system.^24,26,27^ While fitting methods result in parameters that optimally describe existing experimental results, overfitting may produce implausible values or ignore alternative, plausible values.^29,47^

By capturing the uncertainty associated with each parameter value, our approach allows us to simulate the behaviour of an ensemble of models covering a range of plausible parameter combinations and thus assess the full range of system behaviour compatible with our current knowledge of the parameter values and network topology. However, overestimation of the uncertainty of kinetic parameters could occur as values observed in a wide range of circumstances are considered when determining parameter distributions. For instance, if experimental outliers are included in the parameterisation pipeline, this could result in overestimating the range of plausible values and thus the uncertainty of the kinetic parameters. The weighting process is designed to account for this, and other scenarios such as data sources which may be unsuitable due to their quality, reliability or relevance. Kinetic values of this nature can be further discounted in a principled and documented way using the parameterisation pipeline we employed^31^ to generate more informative priors.

### 3.3. The AA cascade in HaCaT keratinocytes and 46BR.1N fibroblasts

**Fig. 3:**
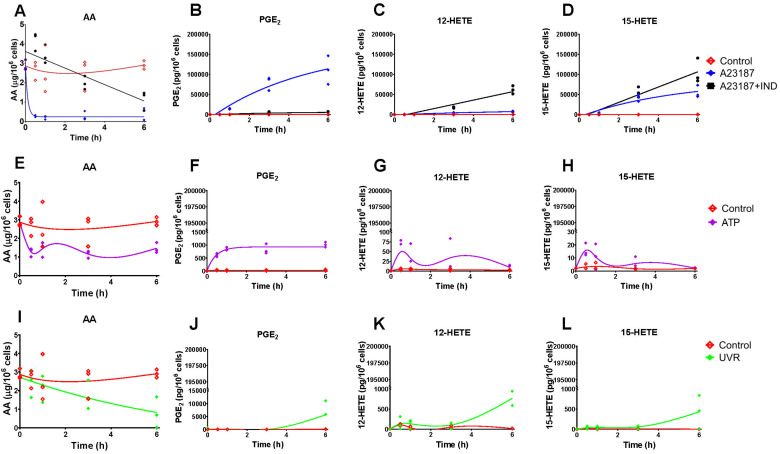
Levels of cellular arachidonic acid (AA) and eicosanoids produced by HaCaT keratinocytes stimulated with calcium ionophore A23187 and indomethacin (IND) (A)–(D), ATP (E)–(H) and ultraviolet irradiation (UVR) (I)–(L). Eicosanoid production by untreated control cells (CTR; red) and post stimulation with A23187 (5 μM, blue), A23187 + IND (10 μM, black), ATP (2 mM, purple) and UVR (15 mJ cm^−2^, green) treatment was measured over time (0, 0.5, 1, 3 and 6 h) by UPLC/ESI-MS/MS; AA release was measured by GC-FID. Data shown as individual points, *n* = 3 independent experiments. Non-linear lines of best fit are shown for untreated control (red) and treated cells as a visual guide only, and do not imply a mechanistic model or most likely dynamics. Hydroxy-eicosatetraenoic acid, HETE; prostaglandin E_2_, PGE_2_.

**Fig. 4:**
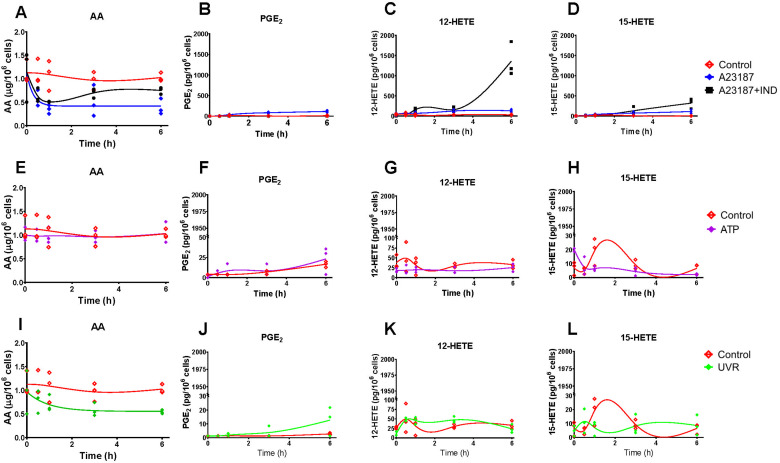
Levels of cellular arachidonic acid (AA) and eicosanoid produced by 46BR.1N fibroblasts stimulated with calcium ionophore A23187 and indomethacin (IND) (A)–(D), ATP (E)–(H) and ultraviolet irradiation (UVR) (I)–(L). Eicosanoid production by untreated control cells (CTR; red) and post stimulation with A23187 (5 μM, blue), A23187 + IND (10 μM, black), ATP (2 mM, purple) and UVR (15 mJ cm^−2^, green) treatment was measured over time (0, 0.5, 1, 3 and 6 h) by UPLC/ESI-MS/MS; AA release was measured by GC-FID. Data shown as individual points, *n* = 3 independent experiments. Non-linear lines of best fit are shown for untreated control (red) and treated cells as a visual guide only and do not imply a mechanistic model or most likely dynamics. Hydroxy eicosatetraenoic acid, HETE; prostaglandin E_2_, PGE_2_.

In order to generate *in vitro* data to assess and support adaptations of the model, we used two human cell lines, HaCaT epidermal keratinocytes and 46BR.1N dermal fibroblasts. Cells were exposed to four treatments, each representing distinct biochemical/pharmacological events: (a) calcium ionophore A23187 to initiate the AA cascade; (b) A23187 in the presence of the COX inhibitor IND; (c) stimulation of the AA cascade by ATP; (d) UV irradiation to activate the AA cascade and upregulate COX-2 expression. Eicosanoid production was assessed by mediator lipidomic analysis of the cell culture media^32,33^ (HaCaT epidermal keratinocytes and 46BR.1N dermal fibroblasts, Fig. 3 and 4, respectively).

**Fig. 5:**
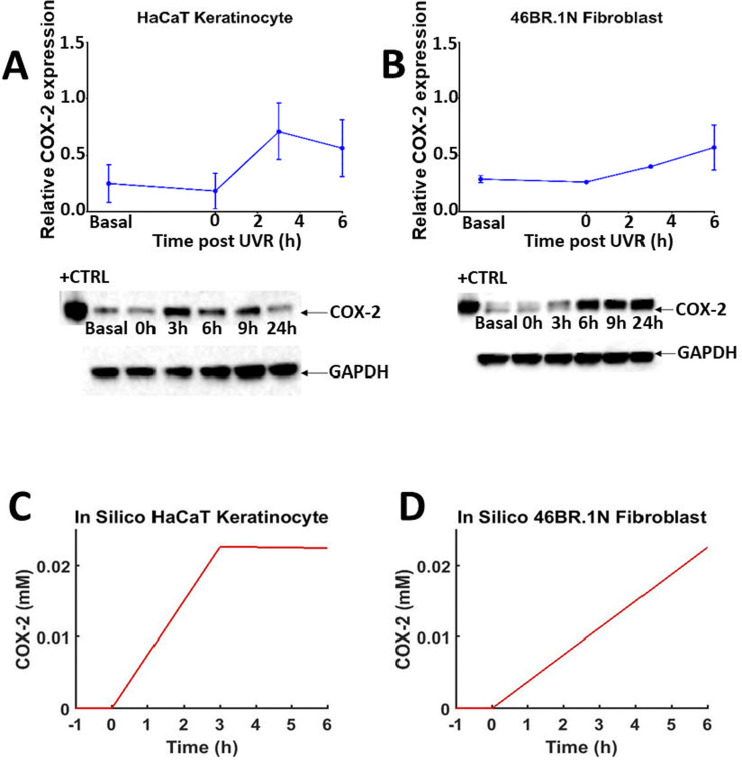
Time-dependent COX-2 protein expression post UVR treatment in HaCaT keratinocytes and 46BR.1N fibroblasts (A) and (B) and *in silico* prediction (C) and (D). Normalised mean protein expression (*n* = 3 independent experiments) and representative western blots for COX-2 and GAPDH (loading control) in HaCaT keratinocytes (A) and 46BR.1N fibroblasts (B) before (basal) and post UVR treatment (15 mJ cm^−2^ UVR). Data expressed as mean ± SEM. “HaCaT + UVR” (C) and “46BR.1N + UVR” (D) *in silico* experiments, showing median COX-2 protein concentration over 6 h in 1000 model variants.

Experimentally, a major distinction between the two cell types examined here was that 46BR.1N fibroblasts had lower levels of cellular AA (Fig. 4(A), (E), (I) and 3(A), (E), (I) respectively), were not as responsive to the stimuli (Fig. 4(B)–(D), (F)–(H), (J)–(L) and 3(B)–(D), (F)–(H), (J)–(L), respectively) and had different COX-2 expression profiles (Fig. 5(B) and (A), respectively) than HaCaT keratinocytes. Cellular AA concentration was found approximately 3-fold higher in unstimulated HaCaT keratinocytes (2.9 μg/10^6^ cells) compared with 46BR.1N fibroblasts (1.1 μg/10^6^ cells) (Fig. 3(A) and 4(A)). Treatment with A23187 decreased cellular AA with concomitant increased production of COX-derived PGE_2_ and LOX-derived 12-HETE and 15-HETE, in both cell lines; HaCaT keratinocytes produced these eicosanoids at much higher concentrations than 46BR1.N fibroblasts, ranging from one to four orders of magnitude, also reflecting the higher cellular AA levels (Fig. 3(A)–(D) and 4(A)–(D)). COX inhibition directed AA to the LOX pathway increasing concentrations of 12-HETE and 15-HETE (Fig. 3(A)–(D) and 4(A)–(D)). Treatment with ATP, a stimulus of the AA cascade successfully used in murine RAW 264.7 macrophages,^57^ increased PGE_2_ production but had little impact on the production of 12- and 15-HETE by HaCaT keratinocytes, whilst little to no activation of the AA cascade was observed in the 46BR.1N fibroblasts (Fig. 3(E)–(H) and 4(E)–(H)).

Cell treatment with UVR increased production of PGE_2_ 3–6 h post stimulation (Fig. 3(J) and 4(J)), an effect attributed to UVR-induced upregulation of COX-2.^58,59^ PGE_2_ production was much more pronounced in UVR-treated HaCaT keratinocytes than UVR-treated 46BR.1N fibroblasts. Western blot analysis confirmed upregulation of COX-2 protein expression in both cell lines (Fig. 5). In HaCaT keratinocytes COX-2 expression was faster and peaked at 3 h post UVR treatment, whereas in 46BR.1N fibroblasts it increased gradually up to 6 h post UVR treatment (Fig. 5(A) and (B)), in accordance with the higher concentrations of eicosanoids produced by HaCaT keratinocytes.

### 3.4. Adapting the model to represent HaCaT keratinocytes and 46BR.1N fibroblasts

**Fig. 6:**
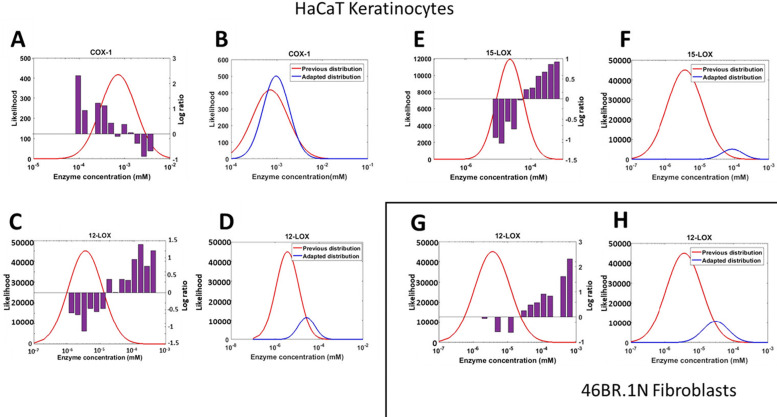
Adapting the protein (enzyme) concentration profile in the generic *in silico* model to generate HaCaT keratinocyte and 46BR.1N fibroblast models. Comparison between the originally sampled parameter distribution and the actual parameters in ensemble members that best predicted the experimental data of HaCaT keratinocytes. The purple bar plots represent the log-ratio of the number of high-quality models containing values in this range, relative to their expected frequency in the sample (A), (C) and (E). The adapted protein (enzyme) concentration distributions for the model of HaCaT keratinocytes, compared with the original distribution (COX-1, 12-LOX and 15-LOX; all *P* < 0.01); the apparent smaller area under the curve for the adjusted distributions is due to the log-scale of the *x*-axis (B), (D) and (F). Comparison between the expected parameter values according to the parameter distribution and the actual parameters that belonged to the model variants which predicted the experimental data in 46BR.1N fibroblasts; the purple bar plots represent the log-ratio of the number of high-quality models containing values in this range, relative to their expected frequency in the sample (G). The adapted protein (enzyme) concentration distribution of 46BR.1N fibroblasts, compared with the original distribution (*P* < 0.01); the apparent smaller area under the curve for the adjusted distribution is due to the log-scale of the *x*-axis (H).

The generic *in silico* model of the AA cascade was further adapted based on the *in vitro* experimental data, to represent the HaCaT keratinocytes and 46BR.1N fibroblasts. Predictions from each generic model variant were assessed for accuracy *via* our novel quality score (eqn (9)). The distribution of [Enzyme]_0_ values in the most accurate models was then assessed against the mode of the original distribution, identifying parts of the distribution where values were enriched or depleted in the best-performing members of the ensemble (Fig. 6). If the parameter distribution of the most accurate model variants were significantly different from the original distribution, the distribution was adjusted to favour the sampling of [Enzyme]_0_ values that are enriched in well-performing models. Therefore, comparison of the range of predicted metabolite concentrations and experimental data allowed further constraints to be placed on the range of plausible values which [Enzyme]_0_ could take.

Generic model variants that most accurately predicted the HaCaT keratinocyte *in vitro* data demonstrated statistically significantly different parameter values for COX-1, 12-LOX and 15-LOX enzyme concentrations compared with the originally sampled distribution (*P* < 0.0001) (Fig. 6(A)–(F)). COX-1 shows a narrower range of values than anticipated, leading to a tightening of the distribution in the successful model (Fig. 6(A) and (B)). Initially, COX-1 concentrations in the range 10^−4^–10^−2^ mM were expected in HaCaT keratinocytes; however, the adapted distribution shows that the true distribution was much tighter around 10^−3^ mM. Accurate model variants also had higher concentrations of 12-LOX and 15-LOX than expected from published information (Fig. 6(C)–(F)). The concentrations of both 12-LOX and 15-LOX were initially distributed between 10^−7^ and 10^−4^ mM; however, accurate model variants used 12-LOX concentrations in the range 10^−6^–10^−4^ mM, and 15-LOX concentrations in the range 10^−5^–10^−4^ mM.

When comparing the predictions of the generic model to the 46BR.1N fibroblast data, only the concentration of 12-LOX was statistically significantly different (*P* < 0.0001) (Fig. 6(G) and (H)). Again, the adapted distribution shifts towards higher concentrations and becomes narrower, as value ranges that yielded inaccurate predictions are excluded. The 46BR.1N fibroblast model was adapted from the same initial model as the HaCaT keratinocytes, so the initial 12-LOX concentration distribution was identical for both cell types (10^−7^–10^−4^ mM); however, in accurate models of 46BR.1N fibroblasts, the sampled 12-LOX concentrations were found in a much narrower range of 10^−5^–10^−4^ mM.

In a published model of anti-inflammatory drug targets in the AA cascade, cell-specific model adaptations were performed by excluding reactions with no experimentally detected products.^22^ However, the network topology could be oversimplified by deleting reactions, as all reactions may be possible in every cell type but not with the same favourable kinetics. Furthermore, deleting reactions can impede model adaptability, as subsequent work may need to reintroduce reactions if the relevant products are detected.^22^ Our approach facilitated more robust model adaptation, as the overall network topology was retained and reaction kinetics were adjusted by constraining the probability distribution of published values. Related to this, if specific pathways are not present in a cell type of interest, the corresponding enzyme concentrations should be set to zero. Adapting the model's protein profile using cell-specific protein data would help with further refining this process. Moreover, in future model adaptations, additional targeted experimentation could be used to constrain plausible parameter values that currently have particularly high uncertainty and/or strong influence on model behaviour.

### 3.5. Adaptations to represent responses to stimuli and inhibitors *in silico*

As cellular eicosanoid profiles depend on both the expression of relevant enzymes (*e.g.*, COX) and availability of substrate fatty acid (*e.g.*, AA), the options to modify both parameters were included in the *in silico* model. To simulate the differences in COX-2 protein induction in various cell types *in silico*, an equation describing protein expression and activity was introduced (eqn (2) and Table 1). The parameters of this equation were chosen so that the COX-2 concentration increased at fixed timepoints across a time-course approximating the dynamics of COX-2 induction seen in the *in vitro* data (ESI,† Table S15 and Fig. 5(C), (D)). Future adaptations of the model could expand to accommodate protein and gene expression of further inducible enzymes important for the AA cascade in various systems.

**Fig. 7:**
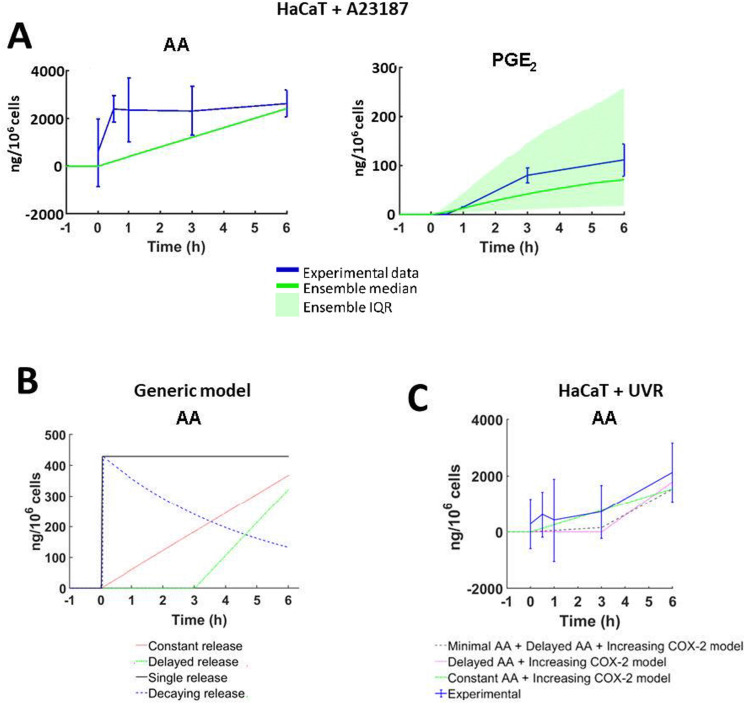
Stimulus-specific adaptations to the arachidonic acid (AA) network models. (A) *In silico* AA concentrations simulated to reflect total *in vitro* eicosanoid production by HaCaT keratinocytes stimulated with calcium ionophore A23187 (5 μM). Experimental data shown as mean ± SD; (*n* = 3 independent experiments; *in silico* data shown as median ± the interquartile range (IQR) of 1000 model variants). (B) Four examples of the dynamic way AA can be introduced into the cascade: single pulse (black), constant (red), decaying (blue) and delayed (green). These examples were created by amending the value of parameters in eqn (1), at set time points (ESI,† Table S14). (C) AA available for the eicosanoid cascade as predicted by the “HaCaT + UVR” optimisation models. ‘Minimal AA + Delayed AA + COX-2’ (grey), ‘Delayed AA’ (pink), ‘Constant AA + COX-2’ (green), ‘Constant AA’ (green) models; experimental data (blue) shown as mean ± SD, *n* = 3 independent experiments; *in silico* data shown as median ± the IQR of 1000 model variants.

To explore the dynamics of AA release in each cell type and following different treatments, eqn (1) was parameterised with a unique set of estimated values based upon *in vitro* data of cell lines and stimuli/inhibitors, respectively (eqn (1) and ESI,† Table S13). The parameter “Maximum 6 h Concentration of AA” was estimated based on the qualitative trend of eicosanoid production in corresponding *in vitro* cultures (Fig. 7(A) and ESI,† Table S13). In comparison, published models of the AA cascade perform model refinement against experimental data of a single stimulus and cell type. As a result, they cannot adapt the dynamics and magnitude of parameters such as AA release, to reflect the behaviour of different cell types and stimuli.^21–28^ To overcome this limitation, Yang *et al.* adapted the flexibility of their models by excluding reactions from the system.^22^ A specific advantage of our model is that alternative AA release dynamics can be tailored to alter the release of AA depending on experimental design or hypotheses tested. Fig. 7(B) and ESI,† Table S14 illustrate four examples of putative patterns of AA release: single release, decaying release, constant release and delayed release.

The easy adaptability of the model afforded by the flexible AA release dynamics was useful when adjusting the model for simulations of UVR treatment (Fig. 7(C)). “Minimal” AA release was simulated by releasing 10% of the total AA concentration between 0–3 h post-simulation, and the remaining 90% was released between 3–6 h post-simulation. Flexible control of AA release provides a robust tool for future adaptations, as a range of stimuli and cell types can be simulated without the need to add or remove reactions. Further iterations of our model could accommodate complex biological reactions including single or multiple substrate availability and inducible enzyme expression.

### 3.6. Quantification of prediction uncertainty and confidence of the adapted *in silico* models

**Fig. 8:**
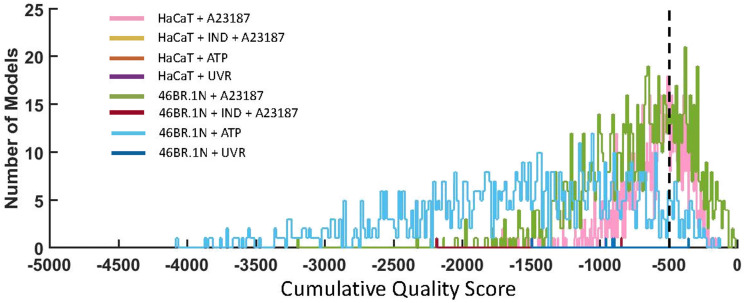
Quality scores comparing the proximity of all ensemble *in silico* model variants with their *in vitro* counterparts using eqn (9). The accuracy of HaCaT keratinocyte models were calculated by comparing the predicted and measured concentration of six eicosanoids (PGE_2_, PGF_2α_, 12-HETE, 15-HETE, 15-keto-PGE_2_ and 13,14-dihydro-15-keto-PGE_2_) at four time points (0.5 h, 1 h, 3 h and 6 h post stimulation). The accuracy of 46BR.1N fibroblast models was calculated by comparing the predicted and measured concentration of three eicosanoids (PGE_2_, 12-HETE and 15-HETE) at four time points (0.5 h, 1 h, 3 h and 6 h post stimulation). A cumulative quality score of >−500 (black, dashed line) indicated that a model variant predicted metabolite concentrations within a relatively close range to experimental data for all datapoints.

Quality score analysis (eqn (9)) was undertaken to assess the accuracy of the *in silico* experiment predictions, compared with the experimental data. Predictions from our simulated scenarios indicated a good qualitative agreement with experimental counterparts, with the A23187 stimulated models producing the most accurate predictions, for both HaCaT keratinocytes and 46BR.1N fibroblasts. Their “Total *Ψ*” scores are 38 and 31 respectively, meaning that 38% of HaCaT + A23187 model variants had a cumulative quality score of >−500 for all timepoints, whilst 31% of 46BR.1N + A23187 model variants had a cumulative quality score of >−500 for all timepoints (Fig. 8).

**Fig. 9:**
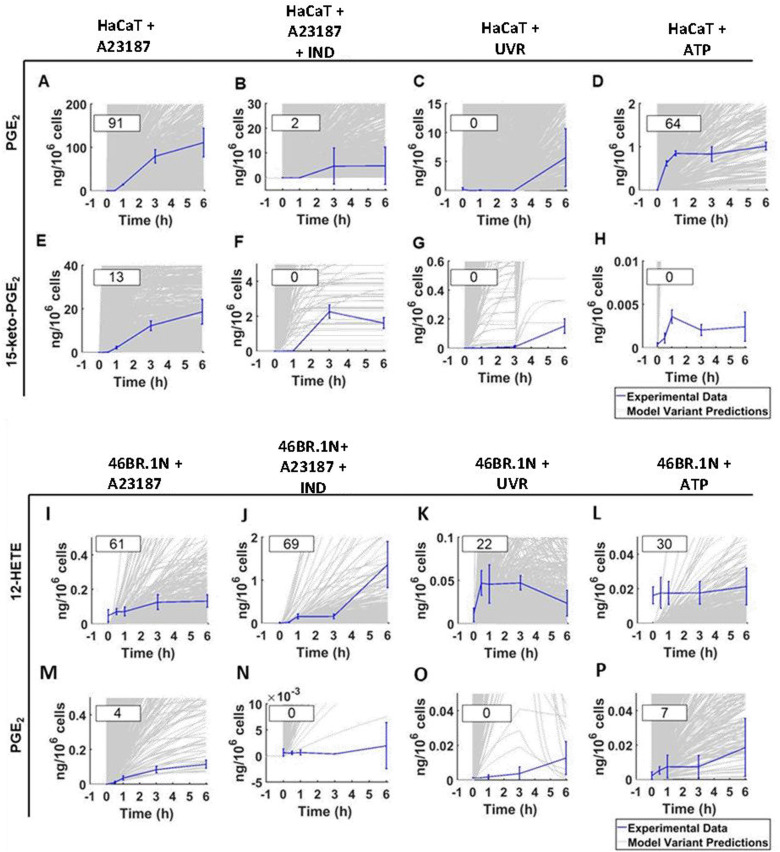
Experimental data compared with the predicted data for examples of highly accurately and inaccurately predicted metabolites in the “HaCaT keratinocyte” *in silico* experiments (PGE_2_, high accuracy example; 15-keto-PGE_2_, low accuracy example) and “46BR.1N fibroblast” *in silico* experiments (12-HETE, high accuracy example; PGE_2_, low accuracy example). Predicted and experimental data, alongside calculated “Metabolite *Ψ*” scores, are shown for HaCaT keratinocytes stimulated with (A) and (E) calcium ionophore A23187, (B) and (F) calcium ionophore A23187 and the COX inhibitor IND, (C) and (G) UVR and (D) and (H) ATP. The HaCaT eicosanoid profiles shown are PGE_2_ (A)–(D) and 15-keto-PGE_2_ (E)–(H). Predicted and experimental data are shown for 46BR.1N fibroblasts stimulated with (I) and (M) calcium ionophore A23187, (J) and (N) calcium ionophore A23187 with the COX inhibitor IND, (K) and (O) UVR and (L) and (P) ATP. The 46BR.1N eicosanoid profiles shown are 12-HETE (I)–(L) and PGE_2_ (M)–(P). Experimental data shown as mean ± SD, *n* = 3 independent experiments (blue); *in silico* data shown based on an ensemble of 1000 model variants (grey); calculated “Metabolite *Ψ*” scores are listed in the upper left corner of each panel.

When comparing the two models, the average “Metabolite *Ψ*” scores indicated that the HaCaT keratinocyte model is more accurate in predicting COX-mediated AA metabolism. “Metabolite *Ψ*” scores indicated that 57% of model variants accurately predicted PGF_2α_ and 39% of model variants accurately predicted PGE_2_, whilst 12-HETE and 15-HETE were predicted accurately in 3% and 2% of models respectively (ESI,† Table S16). Conversely, the 46BR.1N model is more accurate in predicting LOX-derived AA metabolites. The metabolite 12-HETE was predicted accurately in 46% of 46BR.1N model variants, whilst PGE_2_ was predicted accurately in only 3% of 46BR.1N model variants according to the “Metabolite *Ψ*” score (Fig. 9 and ESI,† Table S16). Overall, these findings show that the *in silico* model can differentiate between the main metabolic pathways active in each cell type examined (Fig. 3 and 4).

Furthermore, the lack of a convincing fit between our simulated and experimental data indicates that HaCaT keratinocytes and 46BR.1N fibroblasts may respond differently to the stimuli applied, in ways not accounted for in our model AA cascade. Crosstalk of the eicosanoid cascade with other biochemical reactions that were not modelled here, and additional AA metabolic pathways (*e.g.*, CYP450-mediated reactions, hydrolysis of glycerophospholipid-esterified HETE, production of leukotrienes, *etc.*) which were omitted from our network might account for some of the differences between simulated and experimental observations. Our model also does not include the transcription and translation processes, which are considered to occur at a much slower timescale than the processes studied here.^42,43^ Currently, the low prediction accuracy limits the applicability of the model when predicting experimental outcomes for other cell types and stimuli. Further work is needed to expand the network complexity in future iterations of the model, alongside additional experimentation to reduce the parameter uncertainty in critical areas of the model, as identified by the ensemble modelling strategy.

## 4. Conclusion

This work presents the first model of the AA cascade which employs the Monte Carlo ensemble modelling method. Overall, our approach allows the visualisation of plausible and thermodynamically feasible predictions, overcoming the limitations of fixed-parameter modelling.

The integration of *in vitro* metabolite concentration estimates facilitated the adaptation of our model into cell- and stimulus-specific versions even when no additional condition-specific parameter data were available. The “generic” model proved to be readily adaptable when representing HaCaT keratinocytes or 46BR.1N fibroblasts in response to stimuli and inhibitors of the AA cascade. Furthermore, validation against experimental results using a novel scoring method allowed us to quantify prediction accuracy. Published models report close quantitative agreements between experimental and simulated data, which are only achieved *via* inflexible and fixed parameter fitting methods. We avoided the use of such methods to produce a model that can simulate a wider range of scenarios, whilst accounting for the uncertainty surrounding biological events. This not only avoids the issue of overfitting, but also provides additional transparency regarding areas of remaining uncertainty and identifies gaps in the current knowledge that can be targeted in future experiments. Therefore, our approach generated an adaptable, tuneable ensemble model of the AA cascade that can be tailored to represent different cell types.

In addition, the ensemble modelling approach allowed us to quantify the confidence in individual predictions, highlighting aspects of system behaviour that are robust to uncertainties in the enzyme kinetic parameters, but also openly revealing areas of the system for which confident predictions are currently not yet possible. Thus, this model offers a comprehensive survey of the state of our understanding of the AA cascade, which can form the basis of future modelling activities to enhance our mechanistic understanding of this important pathway.

Future application of *in silico* models like the one presented here, can facilitate the development of new therapeutics by providing statistically rigorous predictions of systems behaviour, support mechanistic insight into the AA cascade which can underpin inflammatory diseases, and contribute to reduction and replacement of preclinical animal models.

## Abbreviations

AA: Arachidonic acid
C-20: 20-Carbon
COX: Cyclooxygenase
CYP450: Cytochrome P450
DGLA: Dihomo-γ-linolenic acid
DMEM: Dulbecco's modified Eagle medium
[Enzyme]_0_: Initial enzyme concentration
EPA: Eicosapentaenoic acid
ESI-MS/MS: Triple quadrupole mass spectrometer with electrospray ionisation
FAME: Fatty acid methyl ester
FBS: Foetal bovine serum
FLAP: 5-Lipoxygenase activating protein
GC-FID: Gas chromatography with flame ionisation
HETE: Hydroxyeicosatetraenoic acid
IND: Indomethacin
KS: Kolmogorov–Smirnov
LOX: Lipoxygenase
LT: Leukotriene
MEM: Minimum essential Eagle medium
ODE: Ordinary differential equation
PBS: Phosphate buffered saline
PG: Prostaglandin
PLA_2_: Phospholipase A_2_
PMNL: Polymorphonuclear leukocyte
PVDF: Polyvinylidene difluoride
PUFA: Polyunsaturated fatty acid
RIPA: Radioimmunoprecipitation assay buffer
SDS: Sodium dodecyl sulfate
TX: Thromboxane
UPLC: Ultraperformance liquid chromatography
UVR: Ultraviolet irradiation

## Data availability

All data supporting this study are provided in full in the ‘Results and Discussion’ section of this paper and in the Supplementary Information files accompanying this paper.

## Author contributions

MU performed cell culture, lipidomic assays, analysed data, generated the *in silico* model and figures. GH edited and amended figures, reproduced and validated *in silico* data. AT, AH and FDC assisted with the design of the model. MK-T performed protein analysis. ACK supervised lipidomic assays. RB, DM, RKB and JJ designed and co-directed the research. AN raised research funds, designed and co-directed the research. All authors contributed to the drafting, reviewing and final approval of the manuscript.

## Conflicts of interest

The project was funded in part through a grant from Unilever to the University of Manchester. DM, RKB and JJ are Unilever employees. The authors have no conflicts to declare.

## Supplementary Material

MO-020-D3MO00187C-s001

MO-020-D3MO00187C-s002

MO-020-D3MO00187C-s003

MO-020-D3MO00187C-s004
