# Supplementary material for: An adaptable in silico ensemble model of the arachidonic acid cascade
Source: Mol Omics. 2024 Jun 3;20(7):453–68. doi: 10.1039/d3mo00187c (PMC11318654; doi:10.1039/d3mo00187c)
Supplement: MO-020-D3MO00187C-s002 [file MO-020-D3MO00187C-s002.pdf]

Electronic Supplementary Material (ESI) for Molecular Omics.
This journal is © The Royal Society of Chemistry 2024

# Supplementary Documents

# An adaptable *in silico* model of the arachidonic acid cascade

Megan Uttley[1#], Grace Horne[1#], Areti Tsigkinopoulou[2], Francesco Del Carratore[2,3], Aliah Hawari[2], Magdalena Kiezel-Tsugunova[1], Alexandra C Kendall[1], Janette Jones[4], David Messenger[5], Ranjit Kaur Bhogal[5], Rainer Breitling[2] and Anna Nicolaou[1,6,*]

## Table of Contents

**Supplementary Table S1.** ........ 2

Description of the event reactions within the model.

**Supplementary Table S2.** ........ 3

Description of the enzymatic reactions in the model.

**Supplementary Table S3.** ........ 6

Description of the non-enzymatic reactions included in the model.

**Supplementary Table S4.** ........ 7

Description of the decay reactions in the model.

**Supplementary Table S5.** ........ 10

Description of the transporter reactions in the model.

**Supplementary Table S6.** ........ 14

Equations used to calculate enzyme kinetic parameters in the AA cascade *in silico* model.

**Supplementary Document S7.** ........ 15

Enzymatic reaction structure and parameterization.

**Supplementary Document S8.** ........ 236

Non-enzymatic reaction structure and parameterization.

**Supplementary Document S9.** ........ 311

Decay reaction structure and parameterization.

**Supplementary Document S10.** ........ 455

Transporter-mediated reaction structure and parameterization.

**Supplementary Document S11.** ........ 763

Initiation reaction structure and parameterization.

**Supplementary Table S12.** ........ 771

Ordinary differential equations for each metabolite in the model.

**Supplementary Table S13.** ........ 774

Parameter changes used to model cell-specific AA release.

**Supplementary Table S14.** ........ 776

Parameter changes used to model generic to produce examples of release of AA.

**Supplementary Table S15.** ........ 777

Parameter changes used to model the *in silico* COX-2 induction.

**Supplementary Table S16.** ........ 778

"Metabolite Ψ" scores used in the cell-specific *in silico* experiments for HaCaT keratinocytes and 46BR.1N fibroblasts.

**Supplementary Table S1. Description of the event reactions within the model.** The table includes the reaction number and reaction scheme of event reactions in the model. Abbreviations: Membrane phospholipid which contain esterified AA (MAA), arachidonic acid (AA), phospholipase $A_2$ (PLA2), generic pool (Φ), cyclooxygenase 2 (COX-2).

| Network Reaction Number | Reaction |
|---|---|
| 1 | $\text{MAA} \overset{PLA2}{\rightleftarrows} \text{AA}$ |
| 95 | Φ→ AA |
| 112 | Φ→ COX-2 |
| 113 | MAA → AA |

**Supplementary Table S2. Description of the enzymatic reactions in the model.** The table includes the reaction number and reaction scheme for each enzymatic reaction included in the model. All reactions take place in the intracellular compartment. Abbreviations: Arachidonic acid (AA), Prostaglandin $H_2$ ($PGH_2$), Cyclooxygenase 2 (COX2), Prostaglandin $F_{2\alpha}$ ($PGF_{2\alpha}$), Prostaglandin F synthase (PGFS), Thromboxane $A_2$ ($TXA_2$), Thromboxane A synthase (TXAS), Prostaglandin $I_2$ ($PGI_2$), Prostaglandin I synthase (PGIS), Prostaglandin $E_2$ ($PGE_2$), Prostaglandin E synthase (PGES), 5-Hydroperoxy-eicosatetraenoic acid (5-HPETE), 5-Lipoxygeanse (5-LOX), 5-Hydroxy-eicosatetraenoic acid (5-HETE), Phospholipid hydroperoxide glutathione peroxidase (PHGPx), Leukotriene $A_4$ ($LTA_4$), Arachidonate 5-lipoxygenase-activating protein (FLAP), 5-Oxo-eicosatetraenoic acid (5-oxo-ETE), 5-Hydroxyeicosanoid dehydrogenase (5-HEDH), Leukotriene $A_4$ hydrolase ($LTA_4H$), Leukotriene $B_4$ ($LTB_4$), Leukotriene $C_4$ ($LTC_4$), Leukotriene $C_4$ synthase ($LTC_4S$), 15-Hydroperoxy-eicosatetraenoic acid (15-HPETE), 15-Lipoxygenase (15-LOX), 12-Hydroperoxy-eicosatetraenoic acid (12-HPETE), 12-Lipoxygenase (12-LOX), 15-Hydroxy-eicosatetraenoic acid (15-HETE), Prostaglandin $D_2$ ($PGD_2$), Prostaglandin D synthase (PGDS), 15-Keto-prostaglandin $E_2$ (15-keto-$PGE_2$), 15-Hydroxyprostaglandin dehydrogenase (15-PGDH), 13,14-Dihydro-15-keto-prostalgandin $E_2$ (13,14-dihydro-15-keto-$PGE_2$), Prostaglandin reductase 2 (PTGR2).

| **Network Reaction Number** | **Reaction** |
|---|---|
| 2 | $\mathrm{AA} \overset{COX-2}{\rightleftarrows} \mathrm{PGH_2}$ |
| 3 | $\mathrm{PGH_2} \overset{PGFS}{\rightleftarrows} \mathrm{PGF_{2\alpha}}$ |
| 4 | $\mathrm{PGH_2} \overset{TXAS}{\rightleftarrows} \mathrm{TXA_2}$ |

| | |
|---|---|
| 5 | $PGIS$<br>$PGH_2 \rightleftarrows PGI_2$ |
| 10 | $PGES$<br>$PGH_2 \rightleftarrows PGE_2$ |
| 11 | $5-LOX$<br>AA $\rightleftarrows$ 5-HPETE |
| 12 | $PHGPx$<br>5-HPETE $\rightleftarrows$ 5-HETE |
| 13 | $5-LOX/FLAP$<br>5-HPETE $\rightleftarrows$ $LTA_4$ |
| 14 | $5-HEDH$<br>5-HETE $\rightleftarrows$ 5-oxo-ETE |
| 15 | $LTA_4H$<br>$LTA_4 \rightleftarrows LTB_4$ |
| 16 | $LTC_4S$<br>$LTA_4 \rightleftarrows LTC_4$ |
| 17 | $15-LOX$<br>AA $\rightleftarrows$ 15-HPETE |
| 18 | $PHGPx$<br>15-HPETE $\rightleftarrows$ 15-HETE |
| 19 | $12-LOX$<br>AA $\rightleftarrows$ 12-HPETE |
| 20 | $PHGPx$<br>12-HPETE $\rightleftarrows$ 12-HETE |
| 21 | $PGDS$<br>$PGH_2 \rightleftarrows PGD_2$ |
| 65 | $COX-1$<br>AA $\rightleftarrows$ $PGH_2$ |
| 66 | $15-PGDH$<br>$PGE_2$ $\rightleftarrows$ 15-keto-$PGE_2$ |

| | |
|---|---|
| 69 | $PTGR2$<br>15-keto-$PGE_2$ ⇄ 13,14-dihydro-15-keto-$PGE_2$ |

**Supplementary Table S3. Description of the non-enzymatic reactions included in the model.** The table includes the reaction number and reaction scheme for each non-enzymatic reaction in the model. Reactions 6–9 take place in the intracellular compartment and reactions 96–99 take place within the extracellular compartment. The prefix "ex" refers to the metabolite located in the extracellular compartment. Abbreviations: Thromboxane $A_2$ ($TXA_2$), Thromboxane $B_2$ ($TXB_2$), Prostaglandin $I_2$ ($PGI_2$), 6-Keto-prostaglandin $F_{1\alpha}$ (6-keto-$PGF_{1\alpha}$), Prostaglandin $D_2$ ($PGD_2$), Prostaglandin $J_2$ ($PGJ_2$), 15-Deoxy-prostalgandin $J_2$ (15-deoxy-$PGJ_2$), Extracellular thromboxane $A_2$ (ex$TXA_2$), Extracellular thromboxane $B_2$ (ex$TXB_2$), Extracellular prostaglandin $I_2$ (ex$PGI_2$), Extracellular 6-keto-prostaglandin $F_{1\alpha}$ (ex6-keto-$PGF_{1\alpha}$), Extracellular prostaglandin $D_2$ (ex$PGD_2$), Extracellular prostaglandin $J_2$ (ex$PGJ_2$), Extracellular 15-deoxy-prostaglandin $J_2$ (ex15-deoxy-$PGJ_2$).

| **Network Reaction Number** | **Reaction** |
|---|---|
| 6 | $TXA_2 \rightleftarrows TXB_2$ |
| 7 | $PGI_2 \rightleftarrows$ 6-keto-$PGF_{1\alpha}$ |
| 8 | $PGD_2 \rightleftarrows PGJ_2$ |
| 9 | $PGJ_2 \rightleftarrows$ 15-deoxy-$PGJ_2$ |
| 96 | ex$TXA_2 \rightleftarrows$ ex$TXB_2$ |
| 97 | ex$PGI_2 \rightleftarrows$ ex6-keto-$PGF_{1\alpha}$ |
| 98 | ex$PGD_2 \rightleftarrows$ ex$PGJ_2$ |
| 99 | ex$PGJ_2 \rightleftarrows$ ex15-deoxy-$PGJ_2$ |

**Supplementary Table S4. Description of the decay reactions in the model**. The table includes the reaction number and reaction scheme each decay reaction in the model. Reactions 72–94 take place in the intracellular compartment and reaction 44–71 take place in the extracellular compartment. The prefix "ex" refers to the metabolite being in the extracellular compartment. Abbreviations: Extracellular prostaglandin $F_{2\alpha}$ (exPG$F_{2\alpha}$), Extracellular thromboxane $B_2$ (exTX$B_2$), Extracellular thromboxane $A_2$ (exTX$A_2$), Extracellular 6-keto prostaglandin $F_{1\alpha}$ (ex6-keto-PG$F_{1\alpha}$), Extracellular prostaglandin $I_2$ (exPG$I_2$), Extracellular prostaglandin $E_2$ (exPG$E_2$), Extracellular 15-deoxy-prostaglandin $J_2$ (ex15-deoxy-PG$J_2$), Extracellular prostaglandin $J_2$ (exPG$J_2$), Extracellular prostaglandin $D_2$ (exPG$D_2$), Extracellular prostaglandin $H_2$ (exPG$H_2$), Extracellular 5-oxo- eicosatetraenoic acid (ex5-oxo-ETE), Extracellular 5-hydroxy-eicosatetraenoic acid (ex5-HETE), Extracellular leukotriene $B_4$ (exLT$B_4$), Extracellular leukotriene $C_4$ (exLT$C_4$), Extracellular leukotriene $A_4$ (exLT$A_4$), Extracellular 5-hydroperoxy-eicosatetraenoic acid (ex5-HPETE), Extracellular 15-hydroxy-eicosatetraenoic acid (ex15-HETE), Extracellular 15-hydroperoxy-eicosatetraenoic acid (ex15-HPETE), Extracellular 12-hydroxy-eicosatetraenoic acid (ex12-HETE), Extracellular 12-hydroperoxy-eicosatetraenoic acid (ex12-HPETE), Extracellular arachidonic acid (exAA), Extracellular 15-keto-prostaglandin $E_2$ (ex15-keto-PG$E_2$), Extracellular 13,14-dihydro-15-keto-prostaglandin $E_2$ (ex13,14-dihydro-15-keto-PG$E_2$), Prostaglandin $F_{2\alpha}$ (PG$F_{2\alpha}$), Thromboxane $B_2$ (TX$B_2$), Thromboxane $A_2$ (TX$A_2$), 6-Keto-prostaglandin $F_{1\alpha}$ (6-keto-PG$F_{1\alpha}$), Prostaglandin $I_2$ (PG$I_2$), Prostaglandin $E_2$ (PG$E_2$), 15-Deoxy-prostaglandin $J_2$ (15-deoxy-PG$J_2$), Prostaglandin $J_2$ (PG$J_2$), Prostaglandin $D_2$ (PG$D_2$), Prostaglandin $H_2$ (PG$H_2$), 5-Oxo-eicosatetraenoic acid (5-oxo-ETE), 5-Hydroxy-eicosatetraenoic acid (5-HETE), Leukotriene $B_4$ (LT$B_4$), Leukotriene $C_4$ (LT$C_4$), Leukotriene $A_4$ (LT$A_4$), 5-hydroperoxy-eicosatetraenoic acid (5-HPETE), 15-Hydroxy-eicosatetraenoic acid (15-HETE), 15-hydroperoxy-eicosatetraenoic acid (15-HPETE), 12-Hydroxy-

eicosatetraenoic acid (12-HETE), 12-hydroperoxy-eicosatetraenoic acid (12-HPETE), Arachidonic acid (AA), 13,14-Dihydro-15-keto-prostaglandin $E_2$ (13,14-dihydro-15-keto-$PGE_2$), 15-keto-prostaglandin $E_2$ (15-keto-$PGE_2$).

| **Network Reaction Number** | **Reaction** |
|---|---|
| 44 | ex$PGF_{2\alpha}$ → Miscellaneous metabolites |
| 45 | ex$TXB_2$ → Miscellaneous metabolites |
| 46 | ex$TXA_2$ → Miscellaneous metabolites |
| 47 | ex6-keto-$PGF_{1\alpha}$ → Miscellaneous metabolites |
| 48 | ex$PGI_2$ → Miscellaneous metabolites |
| 49 | ex$PGE_2$ → Miscellaneous metabolites |
| 50 | ex15-deoxy-$PGJ_2$ → Miscellaneous metabolites |
| 51 | ex$PGJ_2$ → Miscellaneous metabolites |
| 52 | ex$PGD_2$ → Miscellaneous metabolites |
| 53 | ex$PGH_2$ → Miscellaneous metabolites |
| 54 | ex5-oxo-ETE → Miscellaneous metabolites |
| 55 | ex5-HETE → Miscellaneous metabolites |
| 56 | ex$LTB_4$ → Miscellaneous metabolites |
| 57 | ex$LTC_4$ → Miscellaneous metabolites |
| 58 | ex$LTA_4$ → Miscellaneous metabolites |
| 59 | ex5-HPETE → Miscellaneous metabolites |
| 60 | ex15-HETE → Miscellaneous metabolites |
| 61 | ex15-HPETE → Miscellaneous metabolites |
| 62 | ex12-HETE → Miscellaneous metabolites |
| 63 | ex12-HPETE → Miscellaneous metabolites |
| 64 | exAA → Miscellaneous metabolites |
| 68 | ex15-keto-$PGE_2$ → Miscellaneous metabolites |
| 71 | ex13,14-dihydro-15-keto-$PGE_2$→ Miscellaneous metabolites |
| 72 | $PGF_{2\alpha}$ → Miscellaneous metabolites |
| 73 | $TXB_2$ → Miscellaneous metabolites |

| | |
|---|---|
| 74 | $TXA_2$ → Miscellaneous metabolites |
| 75 | 6-keto-$PGF_{1\alpha}$ → Miscellaneous metabolites |
| 76 | $PGI_2$ → Miscellaneous metabolites |
| 77 | $PGE_2$ → Miscellaneous metabolites |
| 78 | 15-deoxy-$PGJ_2$ → Miscellaneous metabolites |
| 79 | $PGJ_2$ → Miscellaneous metabolites |
| 80 | $PGD_2$ → Miscellaneous metabolites |
| 81 | $PGH_2$ → Miscellaneous metabolites |
| 82 | 5-oxo-ETE → Miscellaneous metabolites |
| 83 | 5-HETE → Miscellaneous metabolites |
| 84 | $LTB_4$ → Miscellaneous metabolites |
| 85 | $LTC_4$ → Miscellaneous metabolites |
| 86 | $LTA_4$ → Miscellaneous metabolites |
| 87 | 5-HPETE → Miscellaneous metabolites |
| 88 | 15-HETE → Miscellaneous metabolites |
| 89 | 15-HPETE → Miscellaneous metabolites |
| 90 | 12-HETE → Miscellaneous metabolites |
| 91 | 12-HPETE → Miscellaneous metabolites |
| 92 | AA → Miscellaneous metabolites |
| 93 | 13,14-dihydro-15-keto-$PGE_2$ → Miscellaneous metabolites |
| 94 | 15-keto-$PGE_2$ → Miscellaneous metabolites |

**Supplementary Table S5. Description of the transporter reactions in the model.** The table includes the reaction number for each transporter mediated reactions in the model. Reactions 22–42, 67 and 70 are mediated by the ABC transporter, and reactions 101–11 are mediated by the PGT transporter. The prefix “ex” refers to the metabolite being in the extracellular compartment. Abbreviations: ATP-binding cassette transporters (ABC), Prostaglandin transporter (PGT), Prostaglandin $F_{2\alpha}$ ($PGF_{2\alpha}$), Extracellular prostaglandin $F_{2\alpha}$ ($exPGF_{2\alpha}$), Thromboxane $B_2$ ($TXB_2$), Extracellular thromboxane $B_2$ ($exTXB_2$), 6-Keto-prostaglandin $F_{1\alpha}$ (6-keto-$PGF_{1\alpha}$), Extracellular 6-keto prostaglandin $F_{1\alpha}$ (ex6-keto-$PGF_{1\alpha}$), Prostaglandin $E_2$ ($PGE_2$), Extracellular prostaglandin $E_2$ ($exPGE_2$), 15-Deoxy-prostaglandin $J_2$ (15-deoxy-$PGJ_2$), Extracellular 15-deoxy-prostaglandin $J_2$ (ex15-deoxy-$PGJ_2$), 5-Oxo-eicosatetraenoic acid (5-oxo-ETE), Extracellular 5-oxo- eicosatetraenoic acid (ex5-oxo-ETE), 15-Hydroxy-eicosatetraenoic acid (15-HETE), Extracellular 15 -hydroxy-eicosatetraenoic acid (ex15-HETE), Leukotriene $B_4$ ($LTB_4$), Extracellular leukotriene $B_4$ ($exLTB_4$), Leukotriene $C_4$ ($LTC_4$), Extracellular leukotriene $C_4$ ($exLTC_4$), 12-Hydroxy-eicosatetraenoic acid (12-HETE), Extracellular 12-hydroxy-eicosatetraenoic acid (ex12-HETE), Thromboxane $A_2$ ($TXA_2$), Extracellular thromboxane $A_2$ ($exTXA_2$), Prostaglandin $I_2$ ($PGI_2$), Extracellular prostaglandin $I_2$ ($exPGI_2$), Prostaglandin $H_2$ ($PGH_2$), Extracellular prostaglandin $H_2$ ($exPGH_2$), Prostaglandin $D_2$ ($PGD_2$), Extracellular prostaglandin $D_2$ ($exPGD_2$), Prostaglandin $J_2$ ($PGJ_2$), Extracellular prostaglandin $J_2$ ($exPGJ_2$), 12-hydroperoxy-eicosatetraenoic acid (12-HPETE), Extracellular 12-hydroperoxy-eicosatetraenoic acid (ex12-HPETE), 15-hydroperoxy-eicosatetraenoic acid (15-HPETE), Extracellular 15-hydroperoxy-eicosatetraenoic acid (ex15-HPETE), 5-hydroperoxy-eicosatetraenoic acid (5-HPETE), Extracellular 5-hydroperoxy-eicosatetraenoic acid (ex5-HPETE), 5-Hydroxy-eicosatetraenoic acid (5-HETE), Extracellular 5-hydroxy-eicosatetraenoic acid (ex5-HETE), Leukotriene $A_4$ ($LTA_4$), Extracellular leukotriene $A_4$ ($exLTA_4$), Arachidonic acid (AA), Extracellular

arachidonic acid (exAA), 15-keto-prostaglandin $E_2$ (15-keto-$PGE_2$), Extracellular 15-keto-prostaglandin $E_2$ (ex15-keto-$PGE_2$), 13,14-Dihydro-15-keto-prostaglandin $E_2$ (13,14-dihydro-15-keto-$PGE_2$), Extracellular 13,14-dihydro-15-keto-prostaglandin $E_2$ (ex13,14-dihydro-15-keto-$PGE_2$).

| **Network Reaction Number** | **Reaction** |
|---|---|
| 22 | $PGF_{2\alpha} \overset{ABC}{\rightleftarrows} exPGF_{2\alpha}$ |
| 23 | $TXB_2 \overset{ABC}{\rightleftarrows} exTXB_2$ |
| 24 | $6\text{-keto-}PGF_{1\alpha} \overset{ABC}{\rightleftarrows} ex6\text{-keto-}PGF_{1\alpha}$ |
| 25 | $PGE_2 \overset{ABC}{\rightleftarrows} exPGE_2$ |
| 26 | $15\text{-deoxy-}PGJ_2 \overset{ABC}{\rightleftarrows} ex15\text{-deoxy-}PGJ_2$ |
| 27 | $5\text{-oxo-ETE} \overset{ABC}{\rightleftarrows} ex5\text{-oxo-ETE}$ |
| 28 | $15\text{-HETE} \overset{ABC}{\rightleftarrows} ex15\text{-HETE}$ |
| 29 | $LTB_4 \overset{ABC}{\rightleftarrows} exLTB_4$ |
| 30 | $LTC_4 \overset{ABC}{\rightleftarrows} exLTC_4$ |
| 31 | $12\text{-HETE} \overset{ABC}{\rightleftarrows} ex12\text{-HETE}$ |
| 32 | $TXA_2 \overset{ABC}{\rightleftarrows} exTXA_2$ |

| | |
|---|---|
| 33 | $ABC$<br>$PGI_2 \rightleftarrows exPGI_2$ |
| 34 | $ABC$<br>$PGH_2 \rightleftarrows exPGH_2$ |
| 35 | $ABC$<br>$PGD_2 \rightleftarrows exPGD_2$ |
| 36 | $ABC$<br>$PGJ_2 \rightleftarrows exPGJ_2$ |
| 37 | $ABC$<br>12-HPETE ⇄ ex12-HPETE |
| 38 | $ABC$<br>15-HPETE ⇄ ex15-HPETE |
| 39 | $ABC$<br>5-HPETE ⇄ ex5-HPETE |
| 40 | $ABC$<br>5-HETE ⇄ ex5-HETE |
| 41 | $ABC$<br>$LTA_4 \rightleftarrows exLTA_4$ |
| 42 | $ABC$<br>AA ⇄ exAA |
| 67 | $ABC$<br>15-keto-$PGE_2$ ⇄ ex15-keto-$PGE_2$ |
| 70 | $ABC$<br>13,14-dihydro-15-keto-$PGE_2$ ⇄ ex13,14-dihydro-15-keto-$PGE_2$ |
| 101 | $PGT$<br>$PGF_{2\alpha} \rightleftarrows exPGF_{2\alpha}$ |
| 102 | $PGT$<br>$PGE_2 \rightleftarrows exPGE_2$ |
| 103 | $PGT$<br>$PGI_2 \rightleftarrows exPGI_2$ |

| | |
|---|---|
| 104 | $PGD_2 \overset{PGT}{\rightleftarrows} exPGD_2$ |
| 105 | $PGJ_2 \overset{PGT}{\rightleftarrows} exPGJ_2$ |
| 106 | $TXB_2 \overset{PGT}{\rightleftarrows} exTXB_2$ |
| 107 | $\text{13,14-dihydro-15-keto-PGE}_2 \overset{PGT}{\rightleftarrows} \text{ex13,14-dihydro-15-keto-PGE}_2$ |
| 108 | $\text{15-keto-PGE}_2 \overset{PGT}{\rightleftarrows} \text{ex15-keto-PGE}_2$ |
| 109 | $\text{6-keto-PGF}_{1\alpha} \overset{PGT}{\rightleftarrows} \text{ex6-keto-PGF}_{1\alpha}$ |
| 110 | $TXA_2 \overset{PGT}{\rightleftarrows} exTXA_2$ |
| 111 | $\text{15-deoxy-PGJ}_2 \overset{PGT}{\rightleftarrows} \text{ex15-deoxy-PGJ}_2$ |

**Supplementary Table S6. Equations used to calculate enzyme kinetic parameters in the AA cascade *in silico* model.** Where $K_{eq}$ is the equilibrium constant, ΔG is the change in Gibbs Free Energy, R is the gas constant, T is the temperature of the reaction (Kelvin), $K_{Decay}$ is the decay constant, [E] is the enzyme concentration, $K_f$ forward reaction rate constant for non-enzymatic reactions, A is the pre-exponential constant of the Arrhenius equation, and $E_a$ is the activation energy.

## S.6.1. Calculations

**S.Eq.6.1.1.** $$K_{eq} = e^{-\Delta G/RT}$$

**S.Eq.6.1.2.** $$K_{Decay} = \frac{Ln(2)}{\text{Half-life } (min)}$$

**S.Eq.6.1.3.** $$[E](mM) = \frac{[E](ppm)\times \textit{Average number of proteins in a human cell } (10000)}{\textit{Avagradro's Number } (6.023\times10^{23})\times \textit{Cell volume } (3\times10^{-12}L)} \times 1000$$

**S.Eq.6.1.4.** $$K_f = Ae^{\frac{-Ea}{RT}}$$

## S.6.2. Protein concentration conversion

**S.Eq.6.2.1.1** $$Protein\ (mol) = \frac{abundance(ppm) \times \Sigma\ proteins\ in\ a\ cell\ (10^6)}{Na\ (mol)}$$

**S.Eq.6.2.2.** $$Protein\ (mM) = \frac{\text{Protein (mol)}}{volume\ of\ a\ cell\ (L)}$$

**Supplementary Document S7. Enzymatic Reaction Structure and Parameterisation.**

Documentation of parameter values obtained for all enzymatic reactions in the model (Reactions 2–5, 10–21, 65–66, 69; Supplementary Table S2) from the literature and associated uncertainty for the eicosanoid network model. Parameterisation was performed using the method of Tsigkinopoulou *et al.,* (2018). The table includes information regarding each reaction and its respective parameters are documented. This includes information such as the reaction rate law and the literature values that were used to define parameters, including experimental conditions, total weights and literature references from which the data were obtained. In this model some parameters are referred as "Dependent parameters", meaning that the log-normal distribution for that parameter was calculated using multivariate distributions (discussed in **Section 2.6.2**). As a result, no confidence interval factor or literature values were cited for the Dependent parameters.

**Contents**


S.7.1 Reaction 2: AA ⇌ PGH2 ..... 17

S.7.2 Reaction 3: $PGH_2$ ⇌ $PGF_{2\alpha}$ ..... 28

S.7.3 Reaction 4: $PGH_2$ ⇌ $TXA_2$ ..... 39

S.7.4 Reaction 5: $PGH_2$ ⇌ $PGI_2$ ..... 51

S.7.5 Reaction 10: $PGH_2$ ⇌ $PGE_2$ ..... 62

S.7.6 Reaction 11: AA ⇌ 5-HPETE ..... 73

S.7.7 Reaction 12: 5-HPETE ⇌ 5-HETE ..... 84

S.7.8 Reaction 13: 5-HPETE ⇌ $LTA_4$ ..... 95

S.7.9 Reaction 14: 5-HETE ⇌ 5-oxo-ETE ..... 106

S.7.10 Reaction 15: $LTA_4$ ⇌ $LTB_4$ ..... 117

S.7.11 Reaction 16: $LTA_4$ ⇌ $LTC_4$ ..... 130

S.7.12 Reaction 17: AA ⇌ 15-HPETE ..... 141

S.7.13 Reaction 18: 15-HPETE ⇌ 15-HETE ..... 153

S.7.14 Reaction 19: AA ⇌ 12-HPETE ..... 164

S.7.15 Reaction 20: 12-HPETE ⇌ 12-HETE ..... 175

S.7.16 Reaction 21: $PGH_2$ ⇌ $PGD_2$ ..... 186

S.7.17 Reaction 65: AA = $PGH_2$ ..... 197

S.7.18 Reaction 66: $PGE_2$ ⇌ 15-keto-$PGE_2$ ..... 208

S.7.19 Reaction 69: 15-keto-$PGE_2$ ⇌ 13,14-dihydro-15-keto-$PGE_2$ ..... 220

References ..... 231

### S.7.1 Reaction 2: AA ⇌ PGH2

The inducible isoform of COX, COX-2, catalyses the two-step reaction of cycloooxygenation and oxygenation, followed by a hydroperoxide reduction. This isoform is transiently induced in response to inflammatory stimuli, hormones and growth factors (Coffey et al., 1997; DeWitt and Meade, 1993; Fang et al., 2013; Herschman, 1994; Kargman et al., 1995; Kujubu et al., 1993; Lee et al., 1992; Ristimaki et al., 1994). This is a two-step reaction of cycloooxygenation and oxygenation, followed by a hydroperoxide reduction. The cyclooxygenase reaction occurs in the hydrophobic channel within the core of the protein and generates $PGG_2$. The subsequent peroxidase reaction produces $PGH_2$ and occurs at the heme-containing active site near the protein surface. The two step reaction results in the insertion of molecular oxygen across the C-9 and C-11 double bonds.

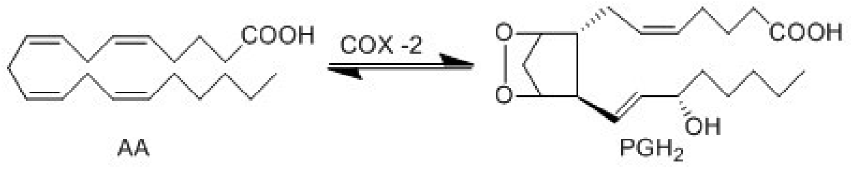


**Figure SF.7.1.** The cycloooxygenation and oxygenation reaction, followed by a hydroperoxide reduction of arachidonic acid (AA) into prostaglandin $H_2$ ($PGH_2$) by cyclooxygenase 2 (COX-2) (Reaction 2).

**SEq.7.1. Reaction rate law for Reaction 2.**

$$\boldsymbol{v_2} = \frac{k_{cat}\cdot[COX-2]\left([AA]-{[PGH_2]}/{K_{eq}}\right)}{K_{m_s}\left(1+\frac{[PGH_2]}{K_{m_p}}\right)+[AA]}$$

### S.7.1.1. Reaction parameters

#### S.7.1.1.1. Parameter: COX-2 $K_{ms}$

Parameter values for the $K_{ms}$ of Reaction 2 were obtained from the literature and summarised in Table ST.7.1.1.1.1. The log-normal distribution properties for the $K_{ms}$ of Reaction 2 are shown in Table ST.7.1.1.1.2 and plotted in Figure SF.7.1.1.1.1.

**Table ST.7.1.1.1.1.** Literature information used to design the COX-2 $K_{ms}$ parameter distribution. Each value was assigned a weight using the protocol described in Section 2.6.2. The type of error was assigned as 0 if it was additive and 1 if it was multiplicative.

| Value (mM) | Error (mM) | Experimental details | | | | | | Weight | Type of error | Reference |
|---|---|---|---|---|---|---|---|---|---|---|
| | | Species | Expression Vector | Enzyme | pH | Temperature (°C) | Other | | | |
| 2.10 $x10^{-3}$ | 4.00 $x10^{-4}$ | Human | Baculovirus | Wild Type Cycloxygenase-2 | 7.2 | 30 | | 128 | 0 | (Bambai et al., 2004) |
| 2.10 $x10^{-3}$ | 4.00 $x10^{-4}$ | Human | E. coli | Wild Type Cycloxygenase-2 | 8.5 | 30 | | 64 | 0 | (Rogge et al., 2004) |
| 5.14 $x10^{-3}$ | 2.90 $x10^{-4}$ | Mouse | Baculovirus | COX-2 | 8 | 37 | 1–200 µM of substrate | 128 | 0 | (Vecchio et al., 2010) |
| 1.62 $x10^{-2}$ | 2.20 $x10^{-3}$ | Human | Embryonic kidney cells | COX-2 | Not specified | Not specified | | 128 | 0 | (Kim et al., 2005) |

**Table ST.7.1.1.1.2.** The log-normal distribution properties of the COX-2 $K_{ms}$ distribution. These values were calculated using the functions described in Section 2.6.2.

| Mode (mM) | Confidence Interval | Location parameter (μ) | Scale parameter (σ) |
|---|---|---|---|
| 4.90 $x10^{-3}$ | 5.70 | -4.72 | 7.77 $x10^{-1}$ |

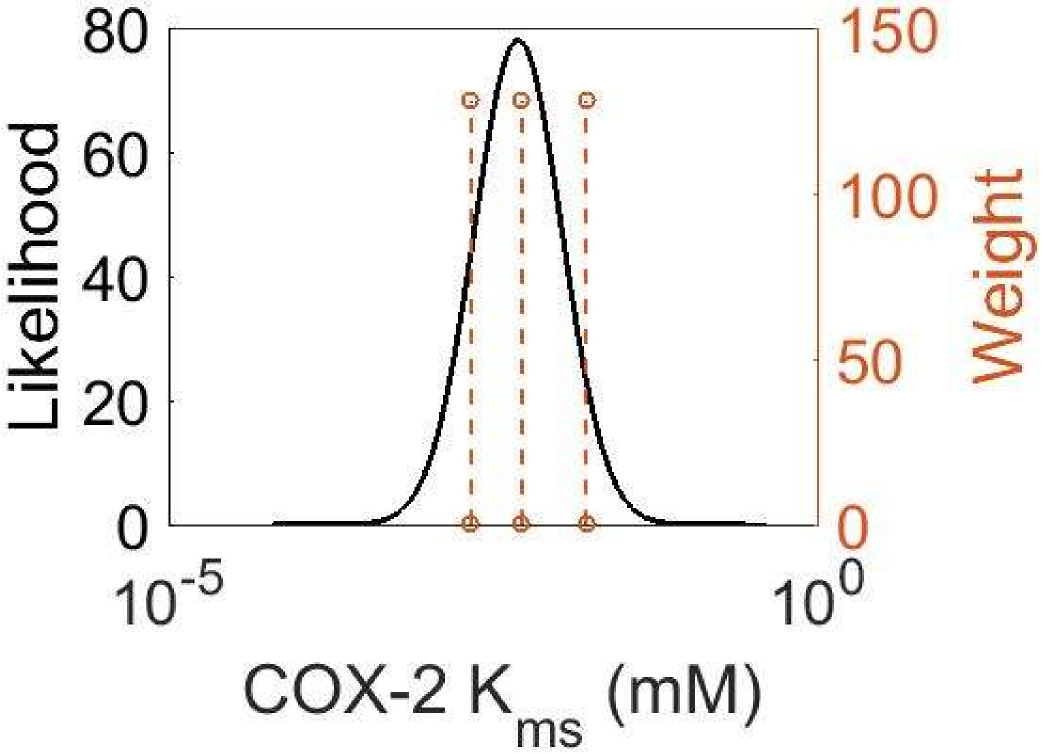


**Figure SF.7.1.1.1.1.** The estimated probability distribution for COX-2 $K_{ms}$. The value and weight of the literature values used to define the distribution are indicated by an orange dashed line. The x axis is plotted on a log-scale.

**S.7.1.1.2. Parameter: COX-2 $K_{mp}$ (Dependent parameter)**

The log-normal distribution for the parameter for the $K_{mp}$ of Reaction 2 was calculated using multivariate distributions. The log-normal distribution properties for the $K_{mp}$ of Reaction 2 are shown in Table ST.7.1.1.2.1 and plotted in Figure SF.7.1.1.2.1.

**Table ST.7.1.1.2.1.** The log-normal distribution properties of the COX-2 $K_{mp}$ distribution. These values were calculated using the functions described in Section 2.6.2.

| Mode (mM) | Location parameter (μ) | Scale parameter (σ) |
|---|---|---|
| 4.70 $x10^{-3}$ | -4.75 | 7.87 $x10^{-1}$ |

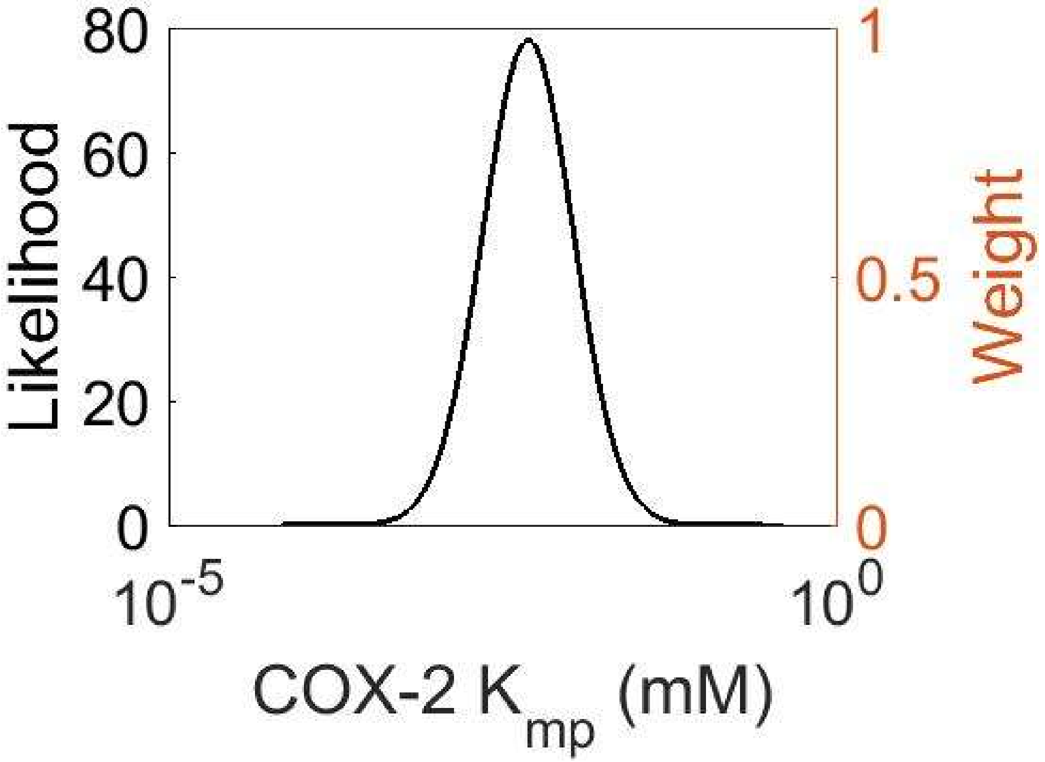


**Figure SF.7.1.1.2.1.** The estimated probability distribution for COX-2 $K_{mp}$. The value and weight of the literature values used to define the distribution are indicated by an orange dashed line. The x axis is plotted on a log-scale.

**S.7.1.1.3. Parameter: COX-2 $k_{cat}$**

Parameter values for the $k_{cat}$ of Reaction 2 were obtained from the literature and summarised in Table ST.7.1.1.3.1. The log-normal distribution properties for the $k_{cat}$ of Reaction 2 are shown in Table ST.7.1.1.3.2 and plotted in Figure SF.7.1.1.3.1.

**Table ST.7.1.1.3.1.** Literature information used to design the COX-2 $k_{cat}$ parameter distribution. Each value was assigned a weight using the protocol described in Section 2.6.2. The type of error was assigned as 0 if it was additive and 1 if it was multiplicative.

| Value ($min^{-1}$) | Error ($min^{-1}$) | Experimental details | | | | | | Weight | Type of error | Reference |
|---|---|---|---|---|---|---|---|---|---|---|
| | | Species | Expression Vector | Enzyme | pH | Temperature (°C) | Other | | | |
| 1.62 $x10^3$ | 2.40 $x10^1$ | Mouse | Baculovirus | COX-2 | 8 | 37 | 1–200 µM of substrate | 128 | 0 | (Vecchio et al., 2010) |

**Table ST.7.1.1.3.2.** The log-normal distribution properties of the COX-2 $k_{cat}$ distribution. These values were calculated using the functions described in Section 2.6.2.

| Mode ($min^{-1}$) | Confidence Interval | Location parameter (μ) | Scale parameter (σ) |
|---|---|---|---|
| 1.62 $x10^3$ | 1.01 | 7.39 | 1.48 $x10^{-2}$ |

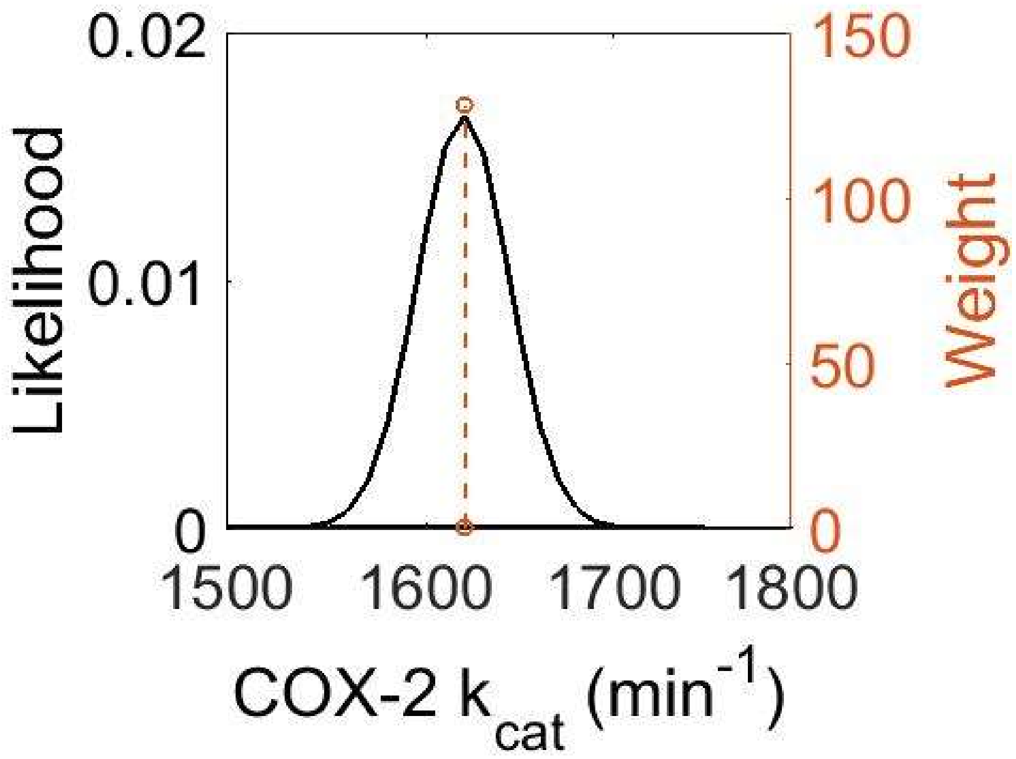


**Figure SF.7.1.1.3.1.** The estimated probability distribution for COX-2 $k_{cat}$. The value and weight of the literature values used to define the distribution are indicated by an orange dashed line. The x axis is plotted on a log-scale.

### S.7.1.1.4. Parameter: COX-2 $K_{eq}$

Parameter values for the $K_{eq}$ of Reaction 2 were obtained from the literature and summarised in Table ST.7.1.1.4.1. The log-normal distribution properties for the $K_{eq}$ of Reaction 2 are shown in Table ST.7.1.1.4.2 and plotted in Figure SF.7.1.1.4.1.

**Table ST.7.1.1.4.1.** Literature information used to design the COX-2 $K_{eq}$ parameter distribution. Each value was assigned a weight using the protocol described in Section 2.6.2. The type of error was assigned as 0 if it was additive and 1 if it was multiplicative.

| ΔG (kcal/mol) | $K_{eq}$ | Experimental details | | | | | | Weight | Type of error | Reference |
|---|---|---|---|---|---|---|---|---|---|---|
| | | Species | Expression Vector | Enzyme | pH | Temperature (°C) | Other | | | |
| $-3.90 \times 10^{1}$ | $4.18 \times 10^{28}$ | Human | Unknown | COX-2 | 7 | Unknown | | 64 | 0 | (Caspi et al., 2018) |

**Table ST.7.1.1.4.2.** The log-normal distribution properties of the COX-2 $K_{eq}$ distribution. These values were calculated using the functions described in Section 2.6.2.

| Mode | Confidence Interval | Location parameter (μ) | Scale parameter (σ) |
|---|---|---|---|
| $4.18 \times 10^{28}$ | $1.00 \times 10^{1}$ | $6.67 \times 10^{1}$ | $8.90 \times 10^{-1}$ |

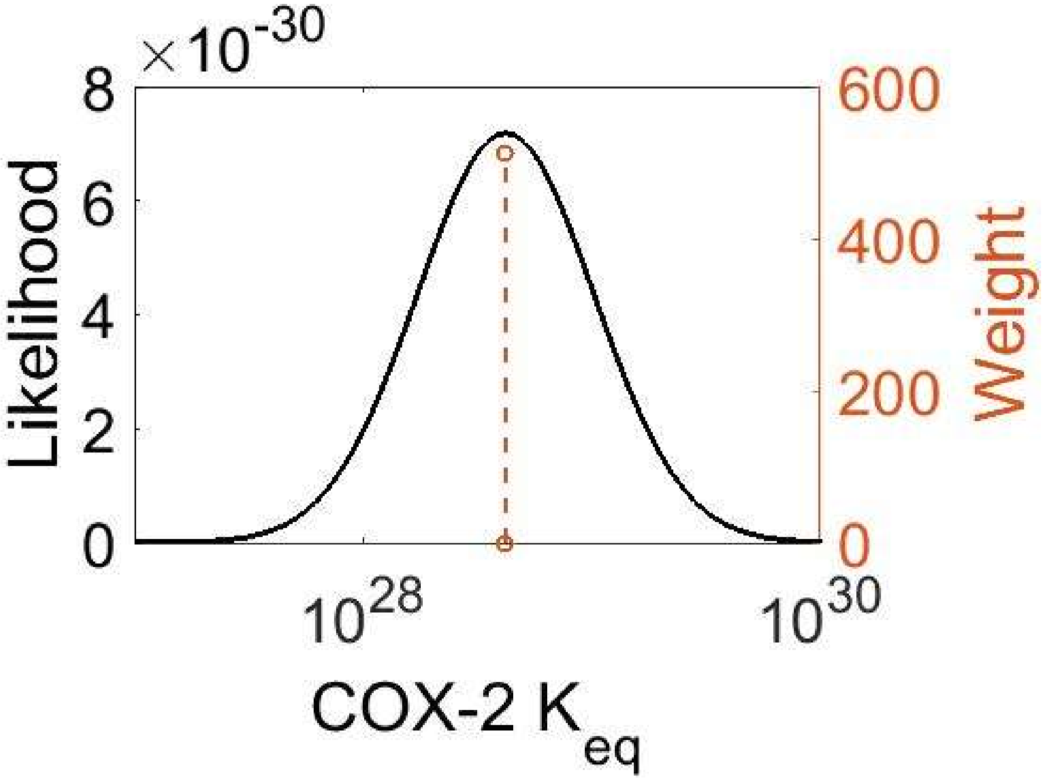


**Figure SF.7.1.1.4.1.** The estimated probability distribution for COX-2 $K_{eq}$. The value and weight of the literature values used to define the distribution are indicated by an orange dashed line. The x axis is plotted on a log-scale.

### S.7.1.1.5. Parameter: COX-2 concentration

Parameter values for the COX-2 concentration of Reaction 2 were obtained from the literature and summarised in Table ST.7.1.1.5.1. The abundance of COX-2 (ppm) was converted to COX-2 (mM) using **Equation S.7.2.** As a result, the concentration of COX-2 in unstimulated tissue was estimated as 2.27 x$10^{-5}$ mM. The upregulation of COX-2 in HaCaT keratinocytes was estimated using western blotting in (Kiezel-Tsugunova, 2017), Figure SF.7.1.1.5.1. shows an example. Using this information, *in silico* experiments which included an upregulation of COX-2, included the concentration of COX-2 reaching 100 times higher concentration than the estimated unstimulated concentration. Therefore, the COX-2 induction event includes the concentration of COX-2 eventually reaching a concentration of 2.27 x$10^{-3}$ mM after 6h post irradiation.

**Table ST.7.1.1.5.1.** Literature information used to determine the concentration of COX-2 in a selection of human tissues. Each value was assigned a weight using the protocol described in Section 2.6.2. The type of error was assigned as 0 if it was additive and 1 if it was multiplicative.

| Value (mM) | Error (mM) | Experimental details | | | | | | Weight | Type of error | Reference |
|---|---|---|---|---|---|---|---|---|---|---|
| | | Species | Expression Vector | Enzyme | pH | Temperature (°C) | Other | | | |
| 4.11 | NaN | Human | Stomach | COX-2 | 7.5 | 37 | | **1024** | 0 | (Wilhelm et al., 2014) |

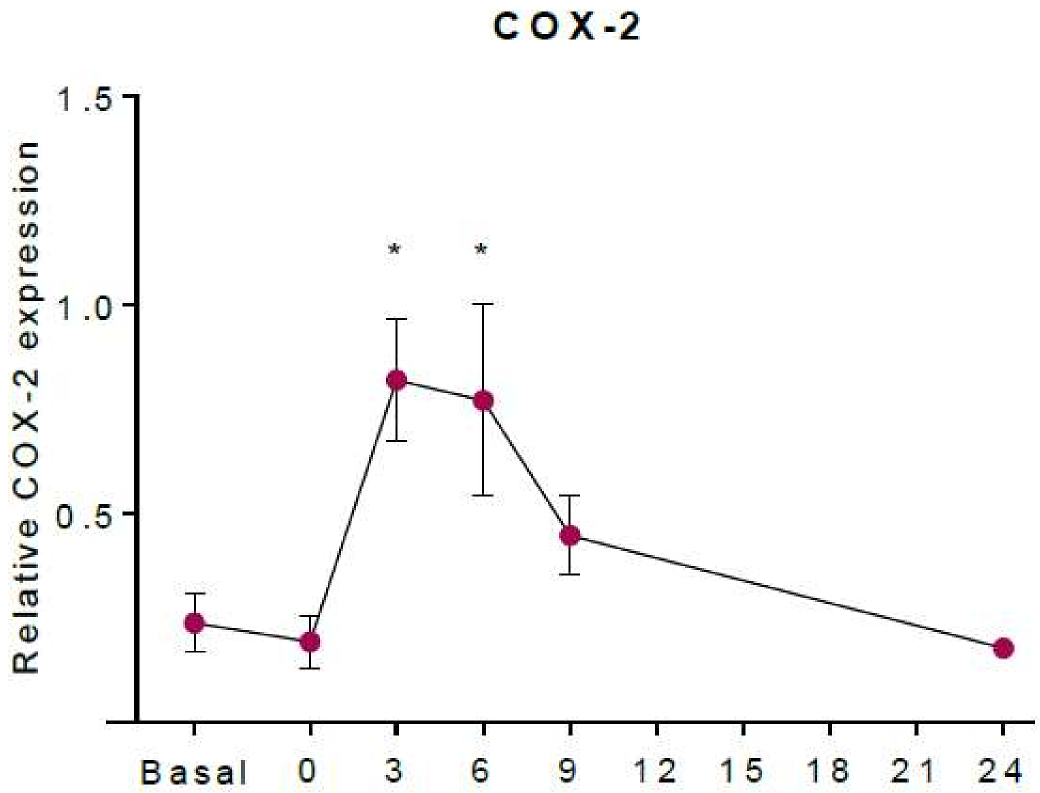


**Figure SF.7.1.1.5.1.** The relative expression of COX-2 protein in HaCaT keratinocytes (Kiezel-Tsugunova, 2017).

### S.7.2 Reaction 3: $PGH_2 \rightleftharpoons PGF_{2\alpha}$

The isomerisation of $PGH_2$ to $PGF_{2\alpha}$ is performed by prostaglandin F synthase (PGFS). This reaction results in the formation of two hydroxyl groups at C9 and C11 of $PGH_2$.

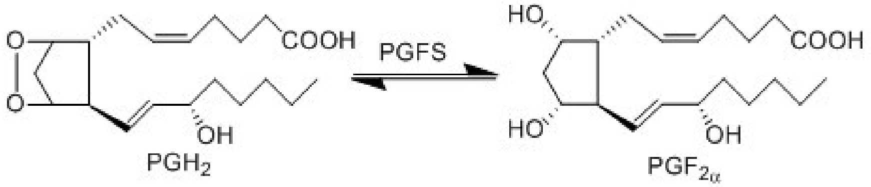


**Figure SF.7.2.** Isomerisation of prostaglandin $H_2$ (PGH) into prostaglandin $F_{2\alpha}$ ($PGF_{2\alpha}$) by prostaglandin F synthase (PGFS) (Reaction 3).

**SEq.7.2. Reaction rate law for Reaction 3.**

$$\boldsymbol{v_3} = \frac{k_{cat} \cdot [PGFS] \left( [PGH_2] - {[\mathrm{PGF}_{2\alpha}]}/{K_{eq}} \right)}{K_{m_s} \left( 1 + \frac{[\mathrm{PGF}_{2\alpha}]}{K_{m_p}} \right) + [PGH_2]}$$

### S.7.2.1. Reaction parameters

#### S.7.2.1.1. Parameter: PGFS $K_{ms}$

Parameter values for the $K_{ms}$ of Reaction 3 were obtained from the literature and summarised in Table ST.7.2.1.1.1. The log-normal distribution properties for the $K_{ms}$ of Reaction 3 are shown in Table ST.7.2.1.1.2 and plotted in Figure SF.7.2.1.1.1.

**Table ST.7.2.1.1.1.** Literature information used to design the PGFS $K_{ms}$ parameter distribution. Each value was assigned a weight using the protocol described in Section 2.6.2. The type of error was assigned as 0 if it was additive and 1 if it was multiplicative.

| Value (mM) | Error (mM) | Experimental details | | | | | | Weight | Type of error | Reference |
|---|---|---|---|---|---|---|---|---|---|---|
| | | Species | Expression Vector | Enzyme | pH | Temperature (°C) | Other | | | |
| 1.80 x10$^{-2}$ | | Human | E. coli | PGFS | 7 | 37 | Placental Aldose Reductase (AKR1B1) | 512 | 0 | (Kabututu et al., 2009) |
| 1.90 x10$^{-3}$ | 1.50 x10$^{-3}$ | Human | E. coli | PGFS | 7 | 37 | Lung PGF2a Synthase (AKR1C3) | 512 | 0 | (Kabututu et al., 2009) |

**Table ST.7.2.1.1.2.** The log-normal distribution properties of the PGFS $K_{ms}$ distribution. These values were calculated using the functions described in Section 2.6.2.

| Mode (mM) | Confidence Interval | Location parameter (μ) | Scale parameter (σ) |
|---|---|---|---|
| 1.33 x10$^{-2}$ | 3.81 | -3.45 | 9.33 x10$^{-1}$ |

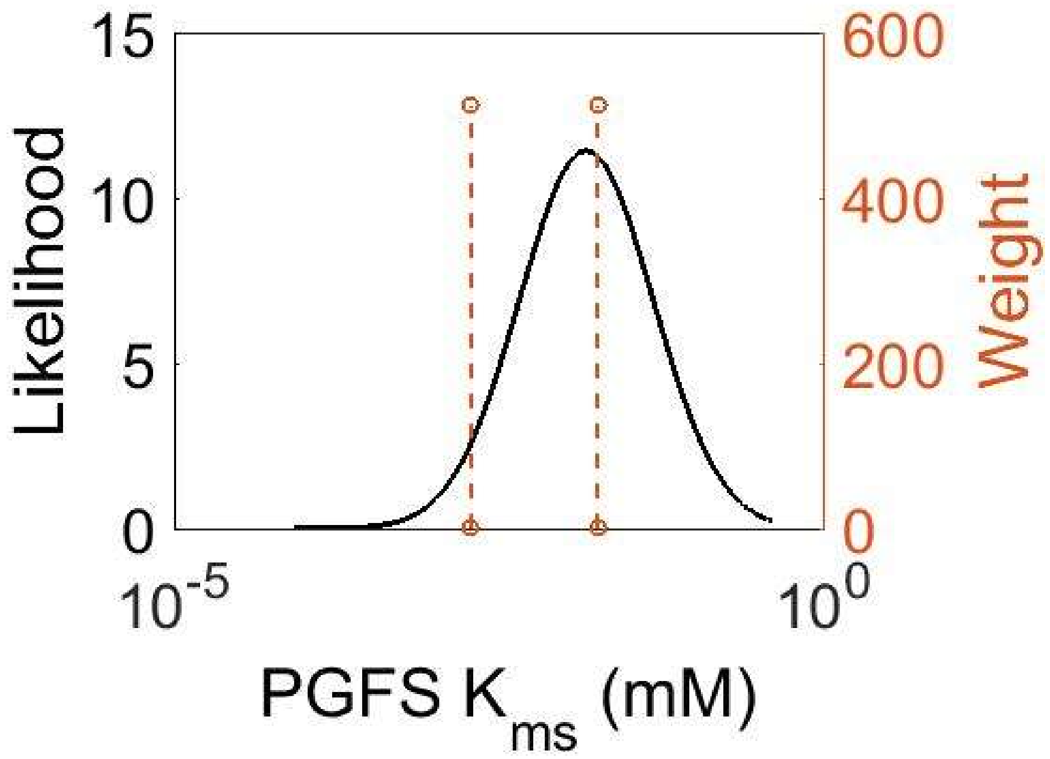


**Figure SF.7.2.1.1.1.** The estimated probability distribution for PGFS $K_{ms}$, plotted on a log-scale. The value and weight of the literature values used to define the distribution are indicated by an orange dashed line.

### S.7.2.1.2. Parameter: PGFS $K_{mp}$ (Dependent parameter)

The log-normal distribution for the parameter for the $K_{mp}$ of Reaction 3 was calculated using multivariate distributions. The log-normal distribution properties for the $K_{mp}$ of Reaction 3 are shown in Table ST.7.2.1.2.1 and plotted in Figure SF.7.2.1.2.1.

**Table ST.7.2.1.2.1.** The log-normal distribution properties of the PGFS-2 $K_{mp}$ distribution. These values were calculated using the functions described in Section 2.6.2.

| Mode (mM) | Location parameter (μ) | Scale parameter (σ) |
|---|---|---|
| $1.46 \times 10^{-2}$ | -2.86 | 1.17 |

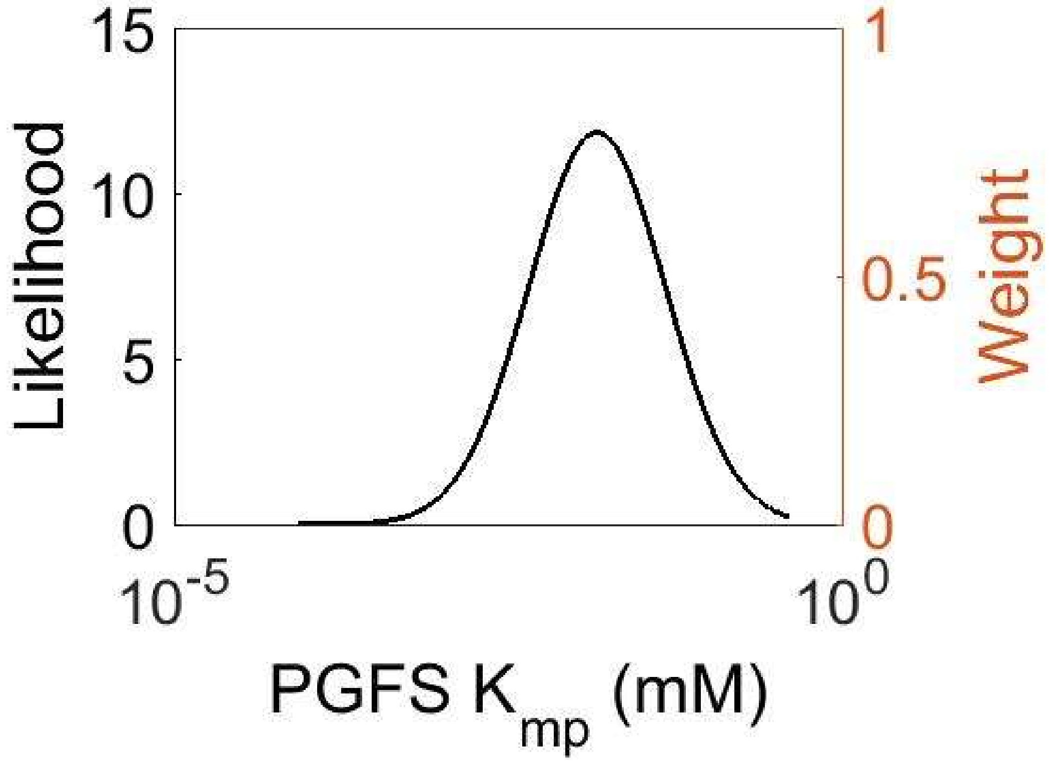


**Figure SF.7.2.1.2.1.** The estimated probability distribution for PGFS $K_{mp}$. The value and weight of the literature values used to define the distribution are indicated by an orange dashed line. The x axis is plotted on a log-scale.

### S.7.2.1.3. Parameter: PGFS $k_{cat}$

Parameter values for the $k_{cat}$ of Reaction 3 were obtained from the literature and summarised in Table ST.7.2.1.3.1. The log-normal distribution properties for the $k_{cat}$ of Reaction 3 are shown in Table ST.7.2.1.3.2 and plotted in Figure SF.7.2.1.3.1.

**Table ST.7.2.1.3.1.** Literature information used to design the PGFS $k_{cat}$ parameter distribution. Each value was assigned a weight using the protocol described in Section 2.6.2. The type of error was assigned as 0 if it was additive and 1 if it was multiplicative.

| Value ($min^{-1}$) | Error ($min^{-1}$) | Experimental details | | | | | | Weight | Type of error | Reference |
|---|---|---|---|---|---|---|---|---|---|---|
| | | Species | Expression Vector | Enzyme | pH | Temperature (°C) | Other | | | |
| 1.49 x$10^1$ | NaN | Mouse/Swine | E. coli | Prostamide/PGF Synthase | 7 | 37 | | 128 | 0 | (Moriuchi et al., 2008) |

**Table ST.7.2.1.3.2.** The log-normal distribution properties of the PGFS $k_{cat}$ distribution. These values were calculated using the functions described in Section 2.6.2.

| Mode ($min^{-1}$) | Confidence Interval | Location parameter (μ) | Scale parameter (σ) |
|---|---|---|---|
| 1.48 x$10^1$ | 1.10 | 2.71 | 9.49 x$10^{-2}$ |

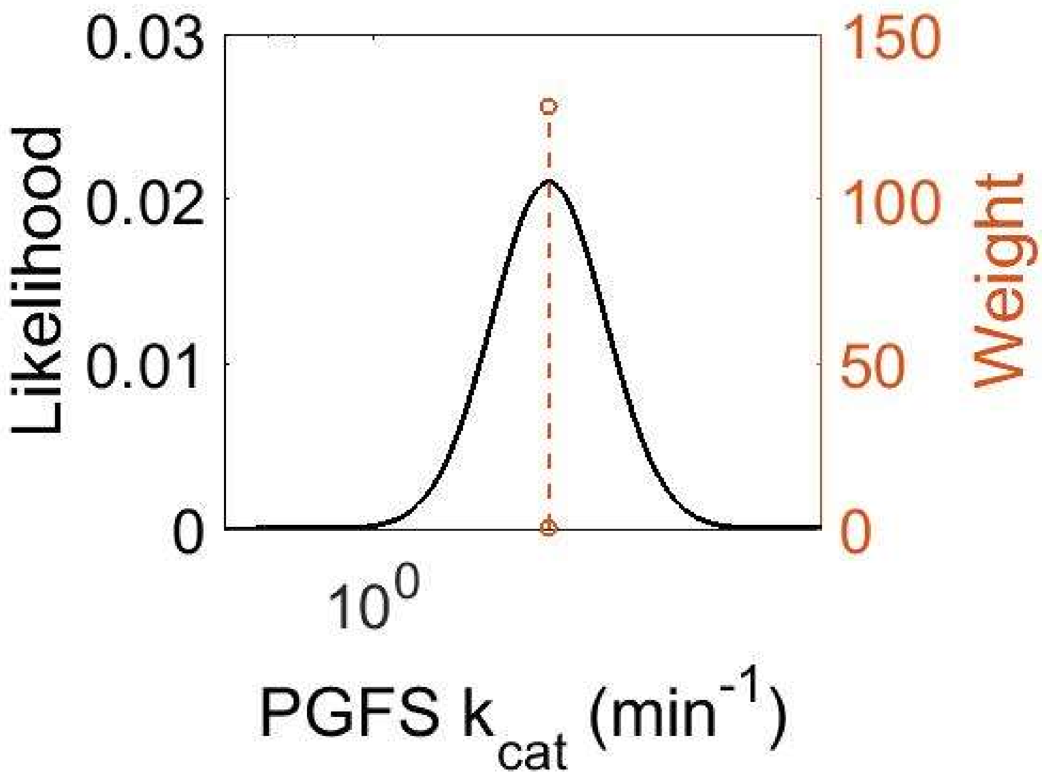


**Figure SF.7.2.1.3.1.** The estimated probability distribution for PGFS $k_{cat}$. The value and weight of the literature values used to define the distribution are indicated by an orange dashed line. The x axis is plotted on a log-scale.

### S.7.2.1.4. Parameter: PGFS $K_{eq}$

Parameter values for the $K_{eq}$ of Reaction 3 were obtained from the literature and summarised in Table ST.7.2.1.4.1. The log-normal distribution properties for the $K_{eq}$ of Reaction 3 are shown in Table ST.7.2.1.4.2 and plotted in Figure SF.7.2.1.4.1.

**Table ST.7.2.1.4.1.** Literature information used to design the PGFS $K_{eq}$ parameter distribution. Each value was assigned a weight using the protocol described in Section 2.6.2. The type of error was assigned as 0 if it was additive and 1 if it was multiplicative.

| ΔG (kcal/mol) | $K_{eq}$ | Experimental details | | | | | | Weight | Type of error | Reference |
|---|---|---|---|---|---|---|---|---|---|---|
| | | Species | Expression Vector | Enzyme | pH | Temperature (°C) | Other | | | |
| -6.64 | 7.46 x10$^{4}$ | Human | Unknown | PGFS | 7 | Unknown | | 64 | 0 | (Caspi et al., 2018) |

**Table ST.7.2.1.4.2.** The log-normal distribution properties of the PGFS $K_{eq}$ distribution. These values were calculated using the functions described in Section 2.6.2.

| Mode | Confidence Interval | Location parameter (μ) | Scale parameter (σ) |
|---|---|---|---|
| 7.46 x10$^{4}$ | 1.00 x10$^{1}$ | 1.20 x10$^{1}$ | 8.90 x10$^{-1}$ |

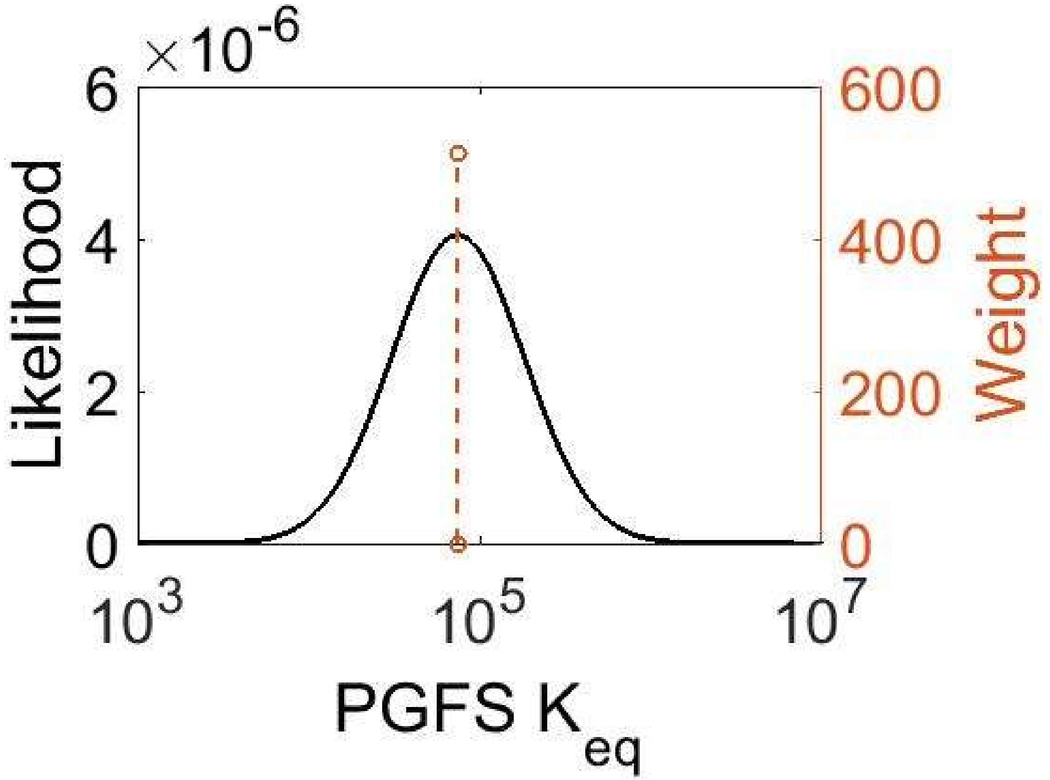


**Figure SF.7.2.1.4.1.** The estimated probability distribution for PGFS $K_{eq}$. The value and weight of the literature values used to define the distribution are indicated by an orange dashed line. The x axis is plotted on a log-scale.

### S.7.2.1.5. Parameter: PGFS concentration

Parameter values for the PGFS concentration of Reaction 3 were obtained from the literature and summarised in Table ST.7.2.1.5.1. The log-normal distribution properties for the PGFS concentration of Reaction 3 are shown in Table ST.7.2.1.5.2 and plotted in Figure SF.7.2.1.5.1. To convert the enzyme concentration from ppm to mM, **Equation S.6.2** was used.

**Table ST.7.2.1.5.1.** Literature information used to design the PGFS concentration parameter distribution. Each value was assigned a weight using the protocol described in Section 2.6.2. The type of error was assigned as 0 if it was additive and 1 if it was multiplicative.

| Value (ppm) | Error (ppm) | Experimental details | | | | | | Weight | Type of error | Reference |
|---|---|---|---|---|---|---|---|---|---|---|
| | | Species | Expression Vector | Enzyme | pH | Temperature (°C) | Other | | | |
| $1.99 \times 10^{1}$ | NaN | Human | Lung | PGFS | 7.5 | 37 | | **1024** | 0 | (Wilhelm et al., 2014) |
| $4.03 \times 10^{1}$ | NaN | Human | Oesophagus | PGFS | 7.5 | 37 | | **1024** | 0 | (Wilhelm et al., 2014) |
| $1.05 \times 10^{2}$ | NaN | Human | Platelet | PGFS | 7.5 | 37 | | **1024** | 0 | (Kim et al., 2014) |
| $1.10 \times 10^{2}$ | NaN | Human | Oral cavity | PGFS | 7.5 | 37 | | **1024** | 0 | (Wilhelm et al., 2014) |

**Table ST.7.2.1.5.2.** The log-normal distribution properties of the PGFS concentration distribution. These values were calculated using the functions described in Section 2.6.2.

| Mode (ppm) | Mode (mM) | Confidence Interval | Location parameter (μ) | Scale parameter (σ) |
|---|---|---|---|---|
| $6.41 \times 10^{1}$ | $3.55 \times 10^{-4}$ | 2.05 | 4.53 | $6.06 \times 10^{-1}$ |

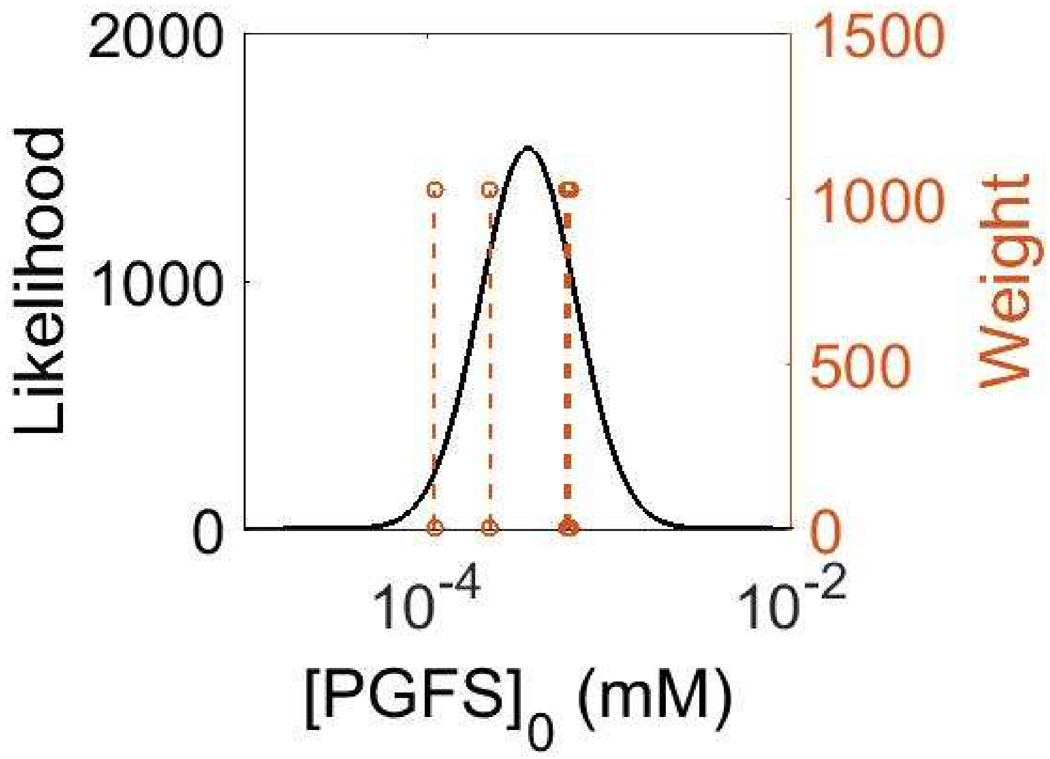


**Figure SF.7.2.1.5.1.** The estimated probability distribution for the PGFS concentration. The value and weight of the literature values used to define the distribution are indicated by an orange dashed line. The x axis is plotted on a log-scale.

### S.7.3 Reaction 4: $PGH_2 \rightleftharpoons TXA_2$

The isomerisation of $PGH_2$ to $TXA_2$ is performed by TXAS. This reaction includes the rearrangement of the peroxide functional group by the protein's heme group, whereby one oxygen is incorporated into the cyclopentane ring between C11 and C12 to form an oxane ring, whilst the other forms an epoxide group across the oxane ring between C9 and C11.

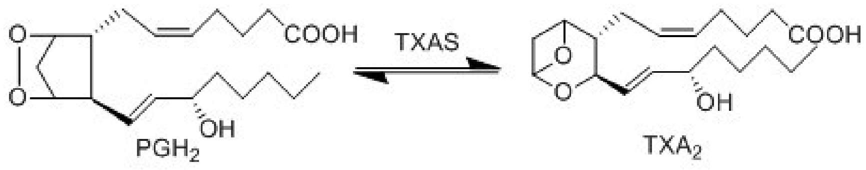


**Figure SF.7.3.** Isomerisation of prostaglandin $H_2$ ($PGH_2$) into thromboxane $A_2$ ($TXA_2$) by thromboxane A synthase (TXAS) (Reaction 4).

**SEq.7.3. Reaction rate law for Reaction 4.**

$$\boldsymbol{v_4} = \frac{\boldsymbol{K_{cat}} \cdot [\boldsymbol{TXAS}]\left([\boldsymbol{PGH_2}] - {[\boldsymbol{TXA_2}]}/{\boldsymbol{K_{eq}}}\right)}{\boldsymbol{K_{m_s}}\left(\mathbf{1} + \frac{[\boldsymbol{TXA_2}]}{\boldsymbol{K_{m_p}}}\right) + [\boldsymbol{PGH_2}]}$$

### S.7.3.1. Reaction parameters

#### S.7.3.1.1. Parameter: TXAS $K_{ms}$

Parameter values for the $K_{ms}$ of Reaction 4 were obtained from the literature and summarised in Table ST.7.3.1.1.1. The log-normal distribution properties for the $K_{ms}$ of Reaction 4 are shown in Table ST.7.3.1.1.2 and plotted in Figure SF.7.3.1.1.1.

**Table ST.7.3.1.1.1.** Literature information used to design the TXAS $K_{ms}$ parameter distribution. Each value was assigned a weight using the protocol described in Section 2.6.2. The type of error was assigned as 0 if it was additive and 1 if it was multiplicative.

| Value (mM) | Error (mM) | Experimental details | | | | | | Weight | Type of error | Reference |
|---|---|---|---|---|---|---|---|---|---|---|
| | | Species | Expression Vector | Enzyme | pH | Temperature (°C) | Other | | | |
| 4.00 x10$^{-3}$ | NaN | Human | Platelet Free Human Monocyte | TXAS | 7.4 | 37 | | 2048 | 0 | (Orlandi et al., 1994) |
| 1.00 x10$^{-2}$ | NaN | Human | Microsome | TXAS | 7.4 | Unknown | | 512 | 0 | (Nusing et al., 1990) |
| 2.20 x10$^{-2}$ | NaN | Human | Platelet | TXAS | 7.4 | 37 | | 2048 | 0 | (Hecker and Ullrich, 1989) |

**Table ST.7.3.1.1.2.** The log-normal distribution properties of the TXAS $K_{ms}$ distribution. These values were calculated using the functions described in Section 2.6.2.

| Mode (mM) | Confidence Interval | Location parameter (μ) | Scale parameter (σ) |
|---|---|---|---|
| 9.96 x10$^{-3}$ | 2.25 | -3.9721 | 0.78374 |

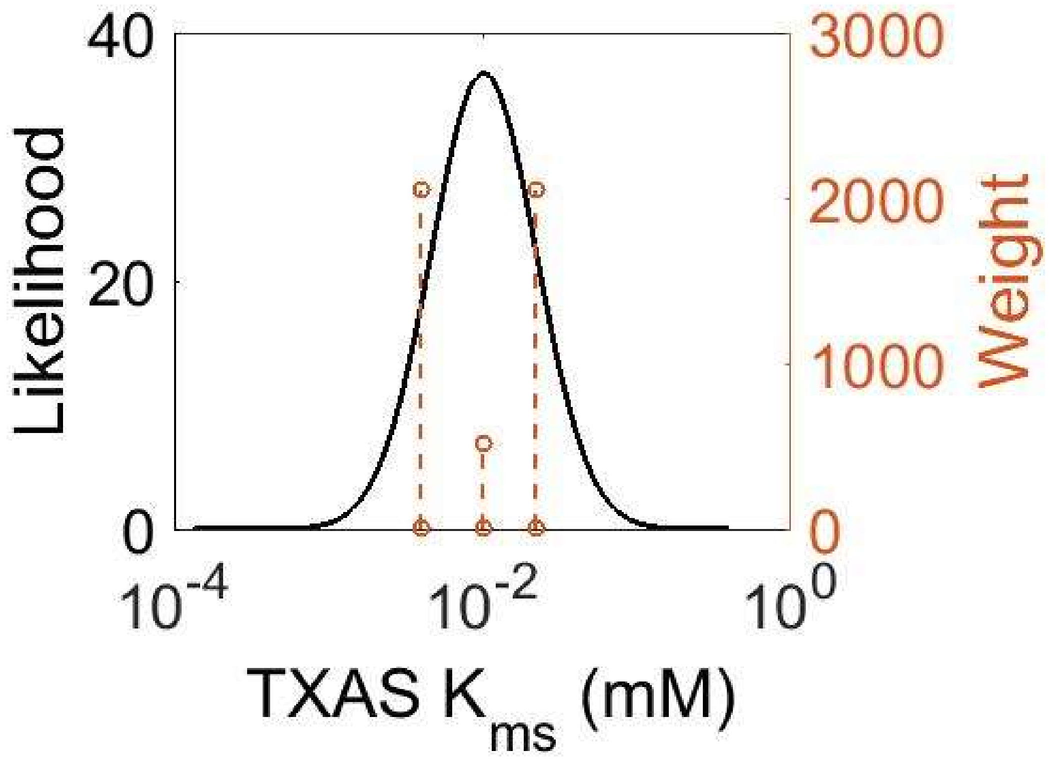


**Figure SF.7.3.1.1.1.** The estimated probability distribution for TXAS $K_{ms}$. The value and weight of the literature values used to define the distribution are indicated by an orange dashed line. The x axis is plotted on a log-scale.

### S.7.3.1.2. Parameter: TXAS $K_{mp}$ (Dependent Parameter)

The log-normal distribution for the parameter for the $K_{mp}$ of Reaction 4 was calculated using multivariate distributions. The log-normal distribution properties for the $K_{mp}$ of Reaction 4 are shown in Table ST.7.3.1.2.1 and plotted in Figure SF.7.3.1.2.1.

**Table ST.7.3.1.2.1.** The log-normal distribution properties of the TXAS $K_{mp}$ distribution. These values were calculated using the functions described in Section 2.6.2.

| Mode (mM) | Location parameter (μ) | Scale parameter (σ) |
|---|---|---|
| $1.03 \times 10^{-2}$ | -3.942048511 | 0.797944323 |

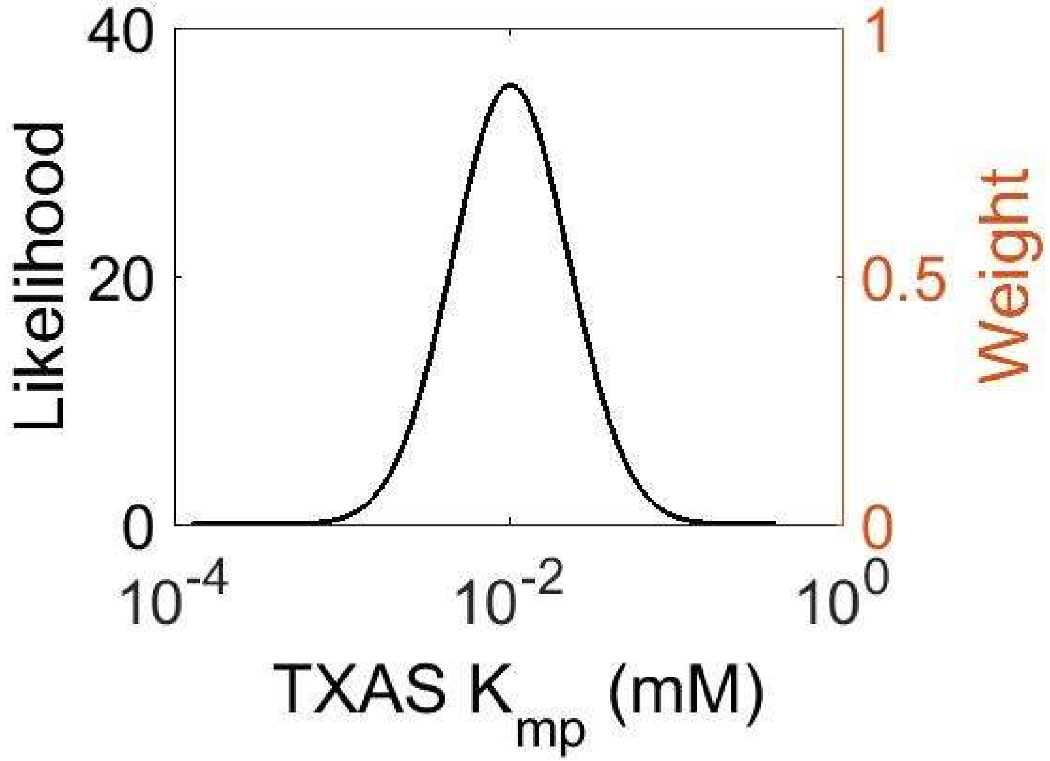


**Figure SF.7.3.1.2.1.** The estimated probability distribution for TXAS $K_{mp}$. The value and weight of the literature values used to define the distribution are indicated by an orange dashed line. The x axis is plotted on a log-scale.

### S.7.3.1.3. Parameter: TXAS $k_{cat}$

Parameter values for the $k_{cat}$ of Reaction 4 were obtained from the literature and summarised in Table ST.7.3.1.3.1. The log-normal distribution properties for the $k_{cat}$ of Reaction 4 are shown in Table ST.7.3.1.3.2 and plotted in Figure SF.7.3.1.3.1.

**Table ST.7.3.1.3.1.** Literature information used to design the TXAS $k_{cat}$ parameter distribution. Each value was assigned a weight using the protocol described in Section 2.6.2. The type of error was assigned as 0 if it was additive and 1 if it was multiplicative.

| Value ($min^{-1}$) | Error (min-1) | Experimental details | | | | | | Weight | Type of error | Reference |
|---|---|---|---|---|---|---|---|---|---|---|
| | | Species | Expression Vector | Enzyme | pH | Temperature (°C) | Other | | | |
| 1.63 $x10^3$ | NaN | Human | Platelet microsomes | TXAS | 7.4 | 37 | | 2048 | 0 | (Haurand and Ullrich, 1985) |
| 2.69 $x10^3$ | NaN | Human | Platelet | TXAS | 7.4 | 30 | | 512 | 0 | (Hecker et al., 1987) |

**Table ST.7.3.1.3.2.** The log-normal distribution properties of the TXAS $k_{cat}$ distribution. These values were calculated using the functions described in Section 2.6.2.

| Mode ($min^{-1}$) | Confidence Interval | Location parameter (μ) | Scale parameter (σ) |
|---|---|---|---|
| 1.67 $x10^3$ | 1.25 | 7.467597712 | 0.216816683 |

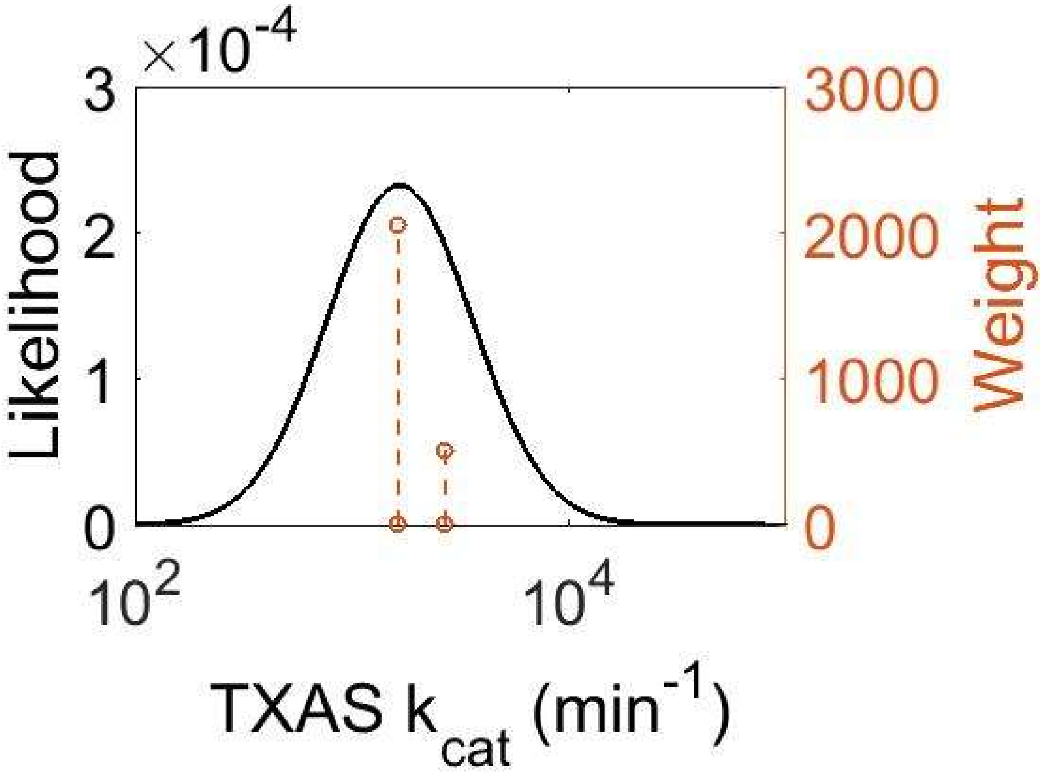


**Figure SF.7.3.1.3.1.** The estimated probability distribution for TXAS $k_{cat}$. The value and weight of the literature values used to define the distribution are indicated by an orange dashed line. The x axis is plotted on a log-scale.

#### S.7.3.1.4. Parameter: TXAS $K_{eq}$

Parameter values for the $K_{eq}$ of Reaction 4 were obtained from the literature and summarised in Table ST.7.3.1.4.1. The log-normal distribution properties for the $K_{eq}$ of Reaction 4 are shown in Table ST.7.3.1.4.2 and plotted in Figure SF.7.3.1.4.1.

**Table ST.7.3.1.4.1.** Literature information used to design the TXAS $K_{eq}$ parameter distribution. Each value was assigned a weight using the protocol described in Section 2.6.2. The type of error was assigned as 0 if it was additive and 1 if it was multiplicative.

| ΔG (kcal/mol) | Keq | Experimental details | | | | | | Weight | Type of error | Reference |
|---|---|---|---|---|---|---|---|---|---|---|
| | | Species | Expression Vector | Enzyme | pH | Temperature (°C) | Other | | | |
| 4.00 x10$^{-2}$ | 9.35 x10$^{-1}$ | Human | Unknown | PGIS | 7 | Unknown | The Gibbs free value for TXAS is 41.08 kcal/mol. This value was much higher than other reactions of similar activity, therefore the value for PGIS was used. | 64 | 0 | (Caspi et al., 2018) |

**Table ST.7.3.1.4.2.** The log-normal distribution properties of the TXAS $K_{eq}$ distribution. These values were calculated using the functions described in Section 2.6.2.

| Mode | Confidence Interval | Location parameter (μ) | Scale parameter (σ) |
|---|---|---|---|
| 9.35 $x10^{-1}$ | 1.00 $x10^{1}$ | 7.30 $x10^{-1}$ | 8.90 $x10^{-1}$ |

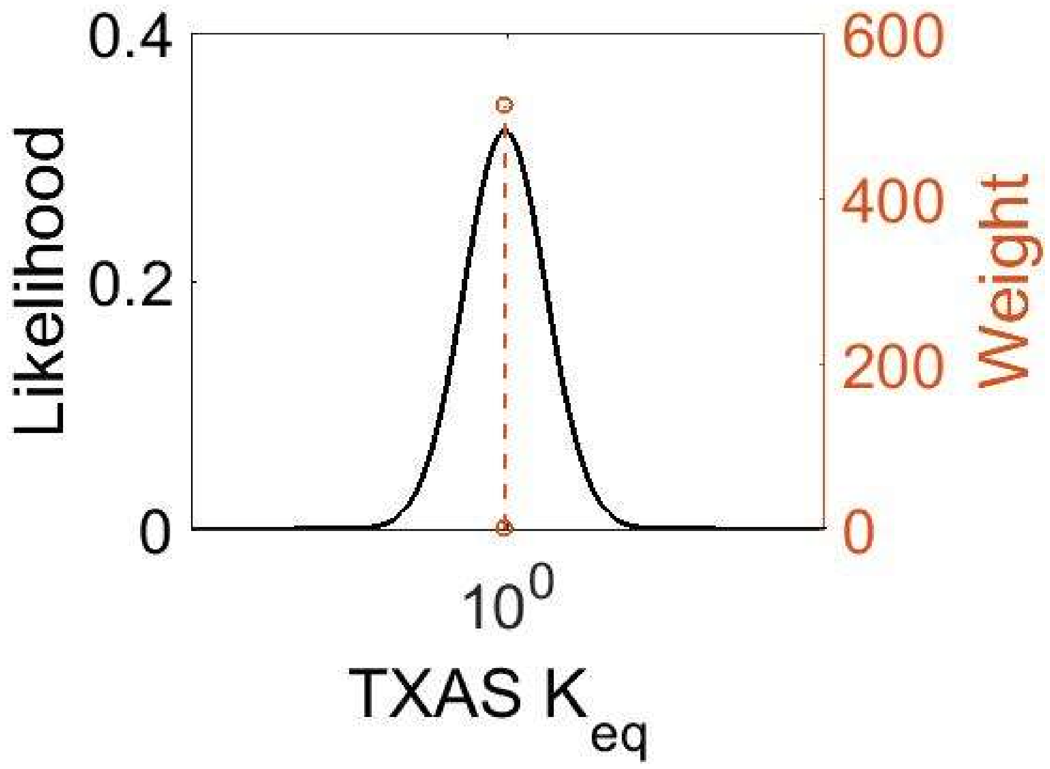


**Figure SF.7.3.1.4.1.** The estimated probability distribution for TXAS $K_{eq}$. The value and weight of the literature values used to define the distribution are indicated by an orange dashed line. The x axis is plotted on a log-scale.

### S.7.3.1.5. Parameter: TXAS concentration

Parameter values for the TXAS concentration of Reaction 4 were obtained from the literature and summarised in Table ST.7.3.1.5.1. The log-normal distribution properties for the TXAS concentration of Reaction 4 are shown in Table ST.7.3.1.5.2 and plotted in Figure SF.7.3.1.5.1. To convert the enzyme concentration from ppm to mM, **Equation S.6.2** was used.

**ST.7.3.1.5.1.** Literature information used to design the TXAS concentration parameter distribution. Each value was assigned a weight using the protocol described in Section 2.6.2. The type of error was assigned as 0 if it was additive and 1 if it was multiplicative.

| Value (ppm) | Error (ppm) | Experimental details | | | | | | Weight | Type of error | Reference |
|---|---|---|---|---|---|---|---|---|---|---|
| | | Species | Expression Vector | Enzyme | pH | Temperature (°C) | Other | | | |
| 4.58 | NaN | Human | Urinary bladder | TXAS | 7.5 | 37 | | **1024** | 0 | (Kim et al., 2014) |
| 5.62 x10$^{1}$ | NaN | Human | Lung | TXAS | 7.5 | 37 | | **1024** | 0 | (Kim et al., 2005) |
| 1.01 x10$^{2}$ | NaN | Human | Oral cavity | TXAS | 7.5 | 37 | | **1024** | 0 | (Wilhelm et al., 2014) |
| 9.49 x10$^{2}$ | NaN | Human | Platelet | TXAS | 7.5 | 37 | | **1024** | 0 | (Kim et al., 2014) |

**Table ST.7.3.1.5.2.** The log-normal distribution properties of the TXAS concentration distribution. These values were calculated using the functions described in Section 2.6.2.

| Mode (ppm) | Mode (mM) | Confidence Interval | Location parameter (μ) | Scale parameter (σ) |
|---|---|---|---|---|
| 7.50 x10$^{1}$ | 4.15 x10$^{-4}$ | 6.69 | 5.66 | 1.16 |

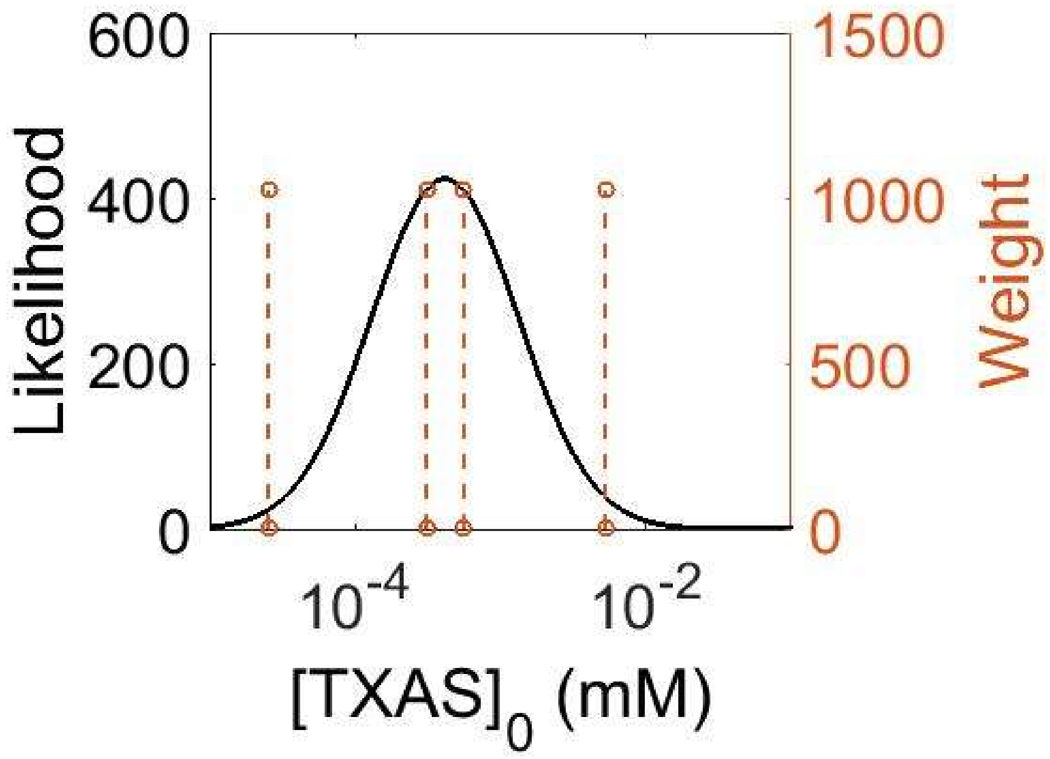


**Figure SF.7.3.1.5.1.** The estimated probability distribution for the TXAS concentration. The value and weight of the literature values used to define the distribution are indicated by an orange dashed line. The x axis is plotted on a log-scale.

### S.7.4 Reaction 5: $PGH_2 \rightleftharpoons PGI_2$

$PGH_2$ is metabolised into the prostacyclin, $PGI_2$, by prostaglandin I synthase (PGIS). This protein is a member of the CYP P450 family, but unlike most CYP P450 enzymes it does not oxidise $PGH_2$. $PGI_2$ is generated by the rearrangement of the peroxide functional group, whereby a hydroxyl group is formed at C11, and a new epoxide ring is formed between C9 and C6.

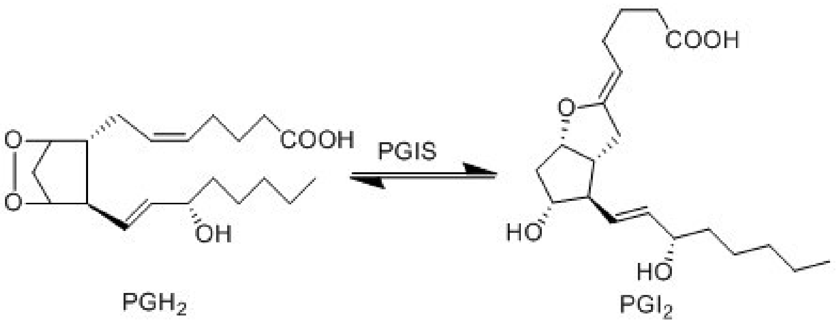


**Figure SF.7.4.** The metabolism of prostaglandin $H_2$ ($PGH_2$) into prostaglandin $I_2$ ($PGI_2$) by prostaglandin I synthase (PGIS) (Reaction 5).

**SEq.7.4. Reaction rate law for Reaction 5.**

$$\boldsymbol{v_5} = \frac{\boldsymbol{K_{cat}} \cdot [\boldsymbol{PGIS}] \left( [\boldsymbol{PGH_2}] - {[\boldsymbol{PGI_2}]}/{\boldsymbol{K_{eq}}} \right)}{\boldsymbol{K_{m_s}} \left( \mathbf{1} + \frac{[\boldsymbol{PGI_2}]}{\boldsymbol{K_{m_p}}} \right) + [\boldsymbol{PGH_2}]}$$

### S.7.4.1. Reaction parameters

#### S.7.4.1.1. Parameter: PGIS $K_{ms}$

Parameter values for the $K_{ms}$ of Reaction 5 were obtained from the literature and summarised in Table ST.7.4.1.1.1. The log-normal distribution properties for the $K_{ms}$ of Reaction 5 are shown in Table ST.7.4.1.1.2 and plotted in Figure SF.7.4.1.1.1.

**Table ST.7.4.1.1.1.** Literature information used to design the PGIS $K_{ms}$ parameter distribution. Each value was assigned a weight using the protocol described in Section 2.6.2. The type of error was assigned as 0 if it was additive and 1 if it was multiplicative.

| Value (mM) | Error (mM) | Experimental details | | | | | | Weight | Type of error | Reference |
|---|---|---|---|---|---|---|---|---|---|---|
| | | Species | Expression Vector | Enzyme | pH | Temperature (°C) | Other | | | |
| 9.00 x10$^{-3}$ | 5.00 x10$^{-3}$ | Bovine | Bovine Endothelial and Aorta Cells | PGIS | 7.4 | 24 | | 192 | 0 | (Hara et al., 1994) |
| 1.33 x10$^{-2}$ | 1.40 x10$^{-3}$ | Human | Bovine Endothelial and Aorta Cells | PGIS | 7.4 | 23 | | 256 | 0 | (Yeh et al., 2005) |

**Table ST.7.4.1.1.2.** The log-normal distribution properties of the PGIS $K_{ms}$ distribution. These values were calculated using the functions described in Section 2.6.2.

| Mode (mM) | Confidence Interval | Location parameter (μ) | Scale parameter (σ) |
|---|---|---|---|
| 1.24 x10$^{-2}$ | 2.46 | -4.22 | 4.20 x10$^{-1}$ |

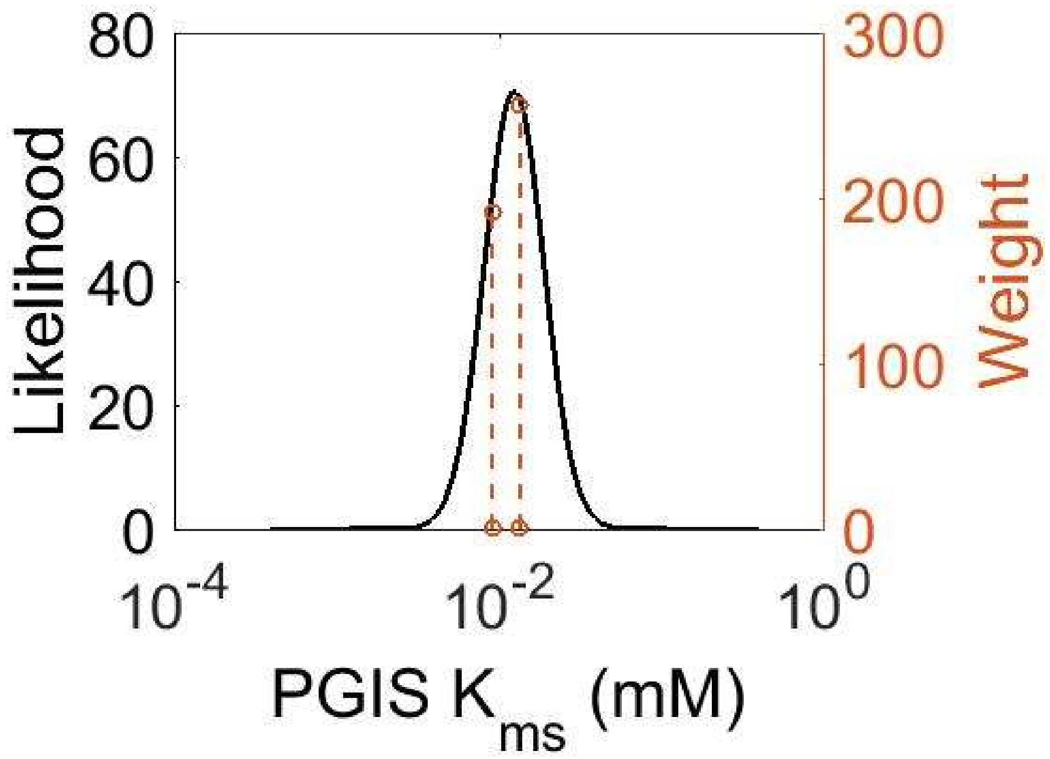


**Figure SF.7.4.1.1.1.** The estimated probability distribution for PGIS $K_{ms}$. The value and weight of the literature values used to define the distribution are indicated by an orange dashed line. The x axis is plotted on a log-scale.

### S.7.4.1.2. Parameter: PGIS $K_{mp}$ (Dependent parameter)

The log-normal distribution for the parameter for the $K_{mp}$ of Reaction 5 was calculated using multivariate distributions. The log-normal distribution properties for the $K_{mp}$ of Reaction 5 are shown in Table ST.7.4.1.2.1 and plotted in Figure SF.7.4.1.2.1.

**Table ST.7.4.1.2.1.** The log-normal distribution properties of the PGIS $K_{mp}$ distribution. These values were calculated using the functions described in Section 2.6.2.

| Mode (mM) | Location parameter (μ) | Scale parameter (σ) |
|---|---|---|
| $1.29 \times 10^{-2}$ | -4.182022605 | 0.414183521 |

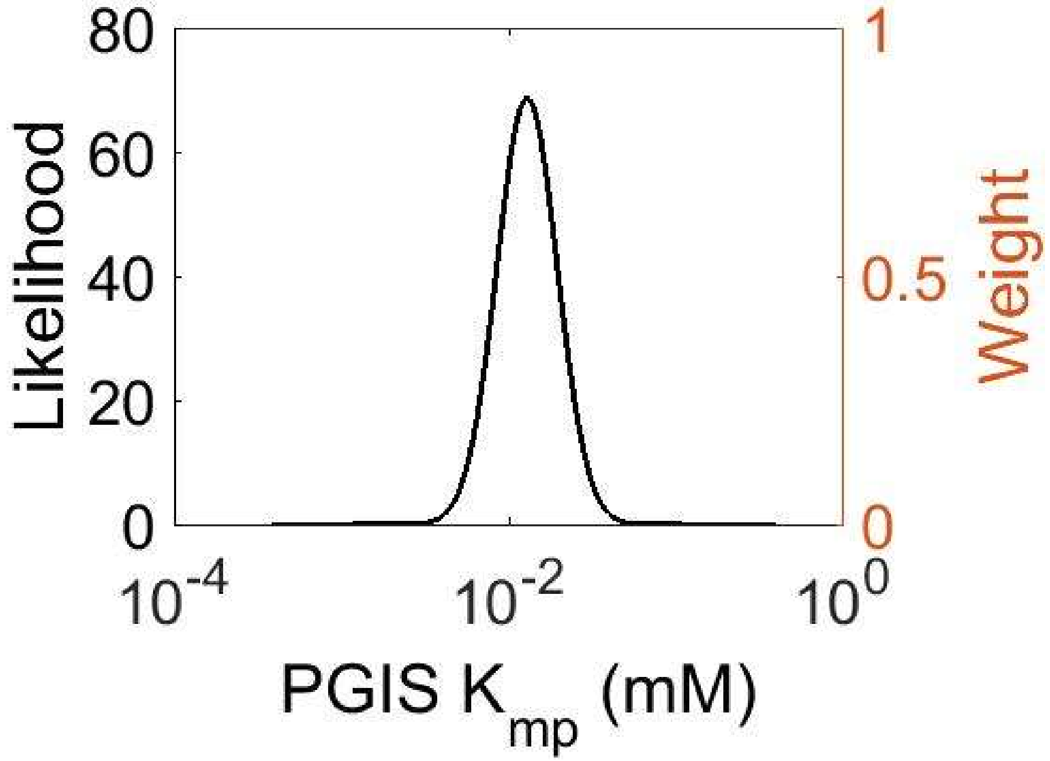


**Table SF.7.4.1.2.1.** The estimated probability distribution for PGIS $K_{mp}$. The value and weight of the literature values used to define the distribution are indicated by an orange dashed line. The x axis is plotted on a log-scale.

#### S.7.4.1.3. Parameter: PGIS $k_{cat}$

Parameter values for the $k_{cat}$ of Reaction 5 were obtained from the literature and summarised in Table ST.7.4.1.3.1. The log-normal distribution properties for the $k_{cat}$ of Reaction 5 are shown in Table ST.7.4.1.3.2 and plotted in Figure SF.7.4.1.3.1.

**Table ST.7.4.1.3.1.** Literature information used to design the PGIS $k_{cat}$ parameter distribution. Each value was assigned a weight using the protocol described in Section 2.6.2. The type of error was assigned as 0 if it was additive and 1 if it was multiplicative.

| Value ($min^{-1}$) | Error (min-1) | Experimental details | | | | | | Weight | Type of error | Reference |
|---|---|---|---|---|---|---|---|---|---|---|
| | | Species | Expression Vector | Enzyme | pH | Temperature (°C) | Other | | | |
| 1.47 $x10^2$ | 4.50 $x10^1$ | Bovine | E. coli | PGIS | 7.4 | 24 | | 192 | 0 | (Hara et al., 1994) |

**Table ST.7.4.1.3.2.** The log-normal distribution properties of the PGIS $k_{cat}$ distribution. These values were calculated using the functions described in Section 2.6.2.

| Mode ($min^{-1}$) | Confidence Interval | Location parameter (μ) | Scale parameter (σ) |
|---|---|---|---|
| 1.41 $x10^2$ | 1.35 | 5.028213628 | 0.287348692 |

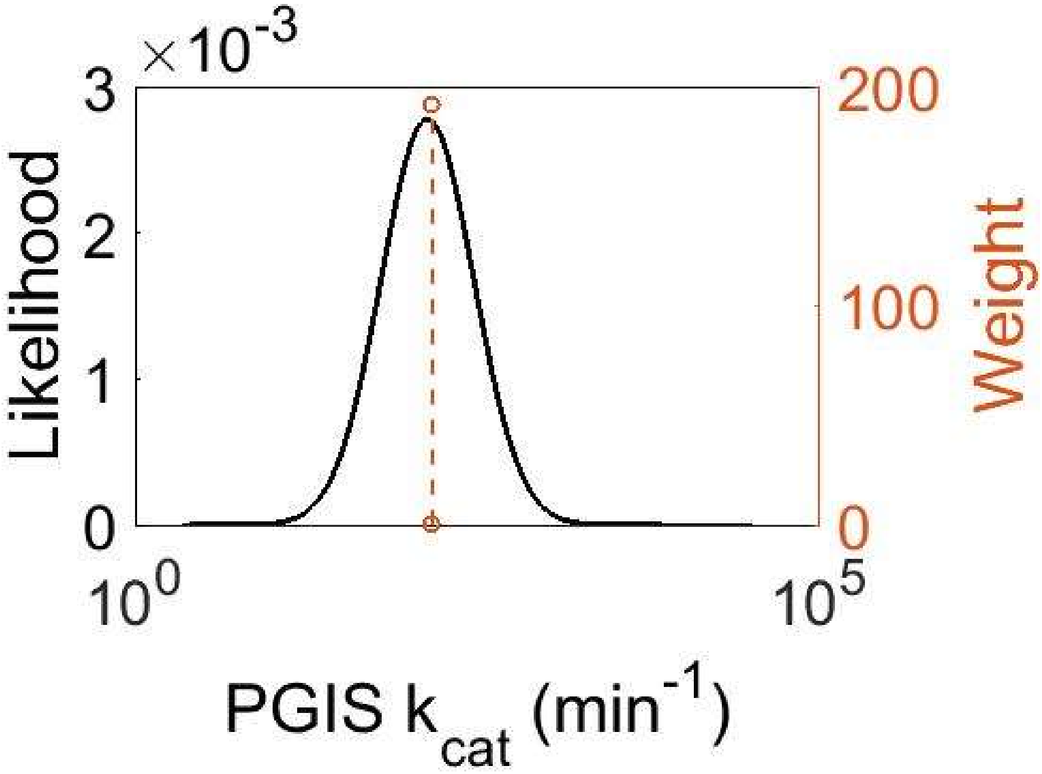


**Figure SF.7.4.1.3.1.** The estimated probability distribution for PGIS $k_{cat}$. The value and weight of the literature values used to define the distribution are indicated by an orange dashed line. The x axis is plotted on a log-scale.

### S.7.4.1.4. Parameter: PGIS $K_{eq}$

Parameter values for the $K_{eq}$ of Reaction 5 were obtained from the literature and summarised in Table ST.7.4.1.4.1. The log-normal distribution properties for the $K_{eq}$ of Reaction 5 are shown in Table ST.7.4.1.4.2 and plotted in Figure SF.7.4.1.4.1.

**Table ST.7.4.1.4.1.** Literature information used to design the PGIS $K_{eq}$ parameter distribution. Each value was assigned a weight using the protocol described in Section 2.6.2. The type of error was assigned as 0 if it was additive and 1 if it was multiplicative.

| ΔG (kcal/mol) | Keq | Experimental details | | | | | | Weight | Type of error | Reference |
|---|---|---|---|---|---|---|---|---|---|---|
| | | Species | Expression Vector | Enzyme | pH | Temperature (°C) | Other | | | |
| 4.00 x10$^{-2}$ | 9.35 x10$^{-1}$ | Human | Unknown | PGIS | 7 | Unknown | | 64 | 0 | (Caspi et al., 2018) |

**Table ST.7.4.1.4.2.** The log-normal distribution properties of the PGIS $K_{eq}$ distribution. These values were calculated using the functions described in Section 2.6.2.

| Mode | Confidence Interval | Location parameter (μ) | Scale parameter (σ) |
|---|---|---|---|
| 9.35 x10$^{-1}$ | 1.00 x10$^{1}$ | 7.30 x10$^{-1}$ | 8.90 x10$^{-1}$ |

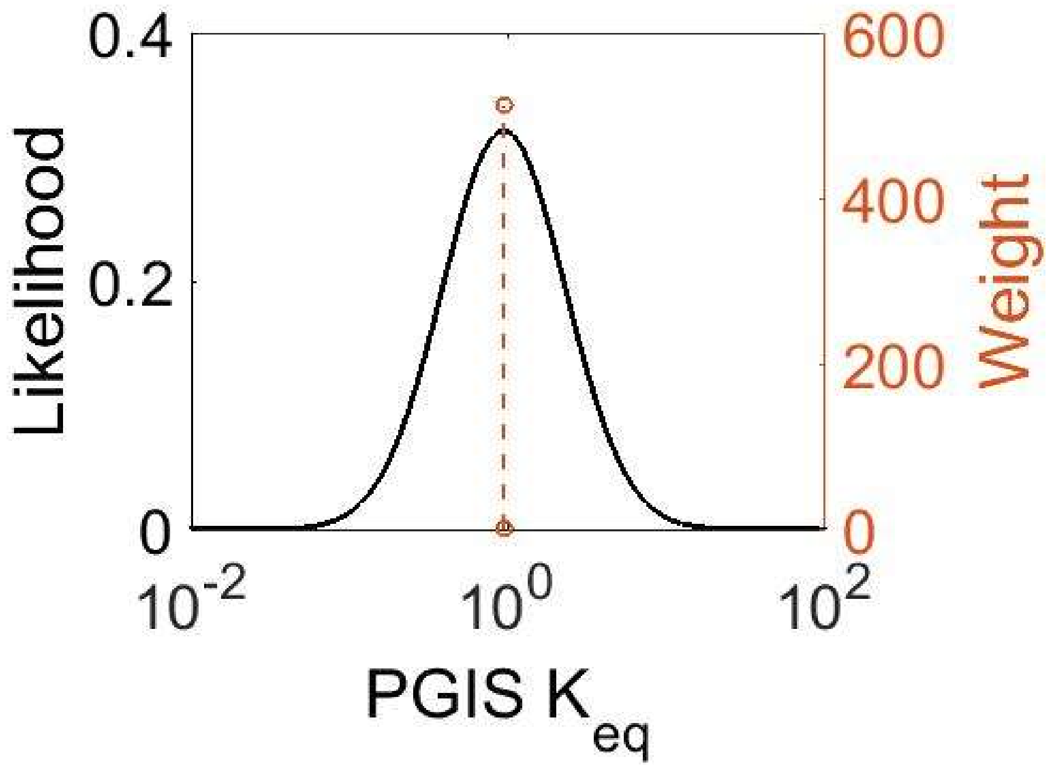


**Figure SF.7.4.1.4.1.** The estimated probability distribution for PGIS $K_{eq}$. The value and weight of the literature values used to define the distribution are indicated by an orange dashed line. The x axis is plotted on a log-scale.

### S.7.4.1.5. Parameter: PGIS concentration

Parameter values for the PGIS concentration of Reaction 5 were obtained from the literature and summarised in Table ST.7.4.1.5.1. The log-normal distribution properties for the PGIS concentration of Reaction 5 are shown in Table ST.7.4.1.5.2 and plotted in Figure SF.7.4.1.5.1. To convert the enzyme concentration from ppm to mM, **Equation S.6.2** was used.

**Table ST.7.4.1.5.1.** Literature information used to design the PGIS concentration parameter distribution. Each value was assigned a weight using the protocol described in Section 2.6.2. The type of error was assigned as 0 if it was additive and 1 if it was multiplicative.

| Value (ppm) | Error (ppm) | Experimental details | | | | | | Weight | Type of error | Reference |
|---|---|---|---|---|---|---|---|---|---|---|
| | | Species | Expression Vector | Enzyme | pH | Temperature (°C) | Other | | | |
| 9.93 | NaN | Human | Oral cavity | PGIS | 7.5 | 37 | | **1024** | 0 | (Wilhelm et al., 2014) |
| 6.01 x$10^1$ | NaN | Human | Oesophagus | PGIS | 7.5 | 37 | | **1024** | 0 | (Kim et al., 2014) |
| 2.06 x$10^2$ | NaN | Human | Lung | PGIS | 7.5 | 37 | | **1024** | 0 | (Kim et al., 2014) |
| 4.12 x$10^2$ | NaN | Human | Urinary bladder | PGIS | 7.5 | 37 | | **1024** | 0 | (Kim et al., 2014) |

**Table ST.7.4.1.5.2.** The log-normal distribution properties of the PGIS concentration distribution. These values were calculated using the functions described in Section 2.6.2.

| Mode (ppm) | Mode (mM) | Confidence Interval | Location parameter (μ) | Scale parameter (σ) |
|---|---|---|---|---|
| 1.13 x$10^2$ | 6.25 x$10^{-4}$ | 4.13 | 5.67 | 9.68 x$10^{-1}$ |

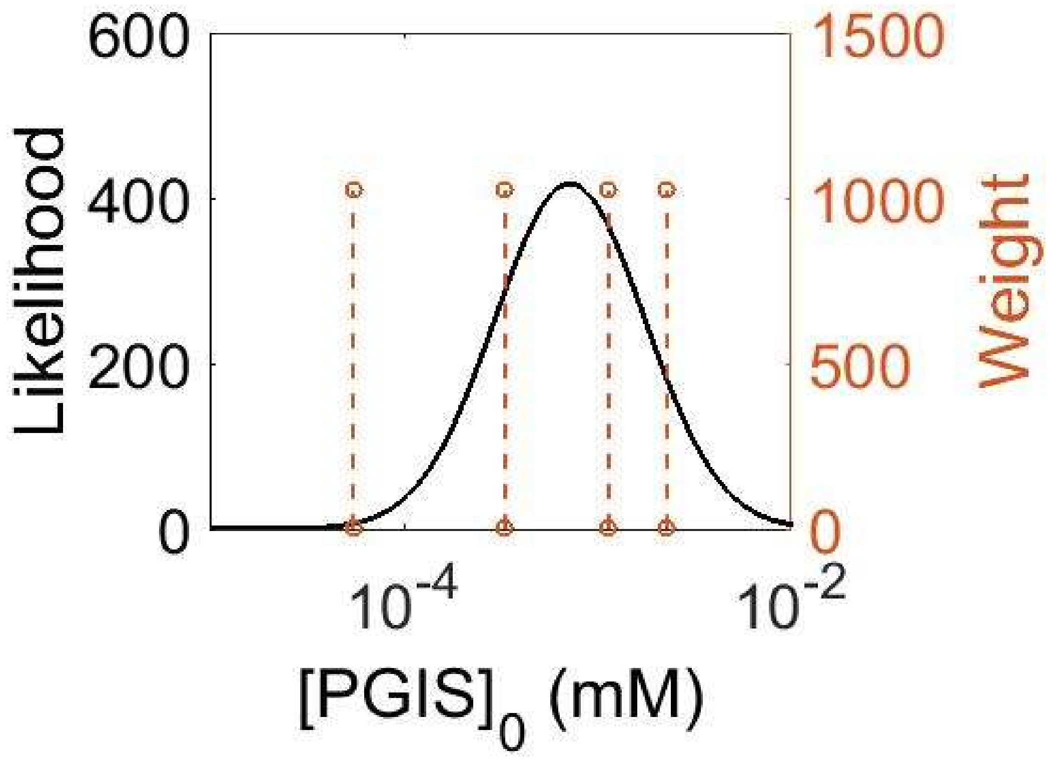


**Figure SF.7.4.1.5.1.** The estimated probability distribution for the PGIS concentration. The value and weight of the literature values used to define the distribution are indicated by an orange dashed line. The x axis is plotted on a log-scale.

### S.7.5 Reaction 10: $PGH_2 \rightleftharpoons PGE_2$

$PGE_2$ is produced by the isomerisation of the $PGH_2$ peroxide, into a ketone at C9 and an alcohol at C11 by PGES, yielding $PGE_2$.

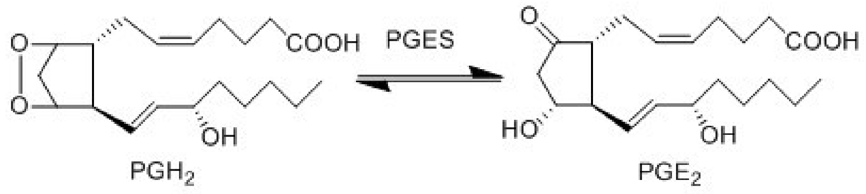


**Figure SF.7.5.** The metabolism of prostaglandin $H_2$ ($PGH_2$) into prostaglandin $E_2$ ($PGE_2$) by prostaglandin E synthase (PGES) (Reaction 10).

**SEq.7.5.** Reaction rate law for Reaction 10.

Eq.S.7.1

$$\boldsymbol{v_{10}} = \frac{K_{cat} \cdot [PGES]\left([PGH_2] - {[PGE_2]}/{K_{eq}}\right)}{K_{m_s}\left(1 + \frac{[PGE_2]}{K_{m_p}}\right) + [PGH_2]}$$

### S.7.5.1. Reaction parameters

#### S.7.5.1.1. Parameter: PGES $K_{ms}$

Parameter values for the $K_{ms}$ of Reaction 10 were obtained from the literature and summarised in Table ST.7.5.1.1.1. The log-normal distribution properties for the $K_{ms}$ of Reaction 10 are shown in Table ST.7.5.1.1.2 and plotted in Figure SF.7.5.1.1.1.

**Table ST.7.5.1.1.1.** Literature information used to design the PGES $K_{ms}$ parameter distribution. Each value was assigned a weight using the protocol described in Section 2.6.2. The type of error was assigned as 0 if it was additive and 1 if it was multiplicative.

| Value (mM) | Error (mM) | Experimental details | | | | | | Weight | Type of error | Reference |
|---|---|---|---|---|---|---|---|---|---|---|
| | | Species | Expression Vector | Enzyme | pH | Temperature (°C) | Other | | | |
| 1.49 $\times10^{-2}$ | NaN | Human | E. coli. | PGES (cPGES) | Unknown | Unknown | | 64 | 0 | (Kobayashi et al., 2004) |
| 6.66 $\times10^{-2}$ | NaN | Human | E. coli. | PGES (cPGES) | Unknown | Unknown | | 64 | 0 | (Kobayashi et al., 2004) |
| 1.60 $\times10^{-1}$ | 4.00 $\times10^{-3}$ | Human | E. coli. | PGES (mPGES-1) | 8 | 37 | | 512 | 0 | (Pettersson et al., 2005) |
| 2.15 $\times10^{-1}$ | NaN | Human | E. coli. | PGES (mPGES-1) | 7.2 | 37 | | 1024 | 0 | (Hamza et al., 2010) |

**Table ST.7.5.1.1.2.** The log-normal distribution properties of the PGES $K_{ms}$ distribution. These values were calculated using the functions described in Section 2.6.2.

| Mode (mM) | Confidence Interval | Location parameter (μ) | Scale parameter (σ) |
|---|---|---|---|
| 1.97 $\times10^{-1}$ | 1.73 | -1.39 | 4.91 $\times10^{-1}$ |

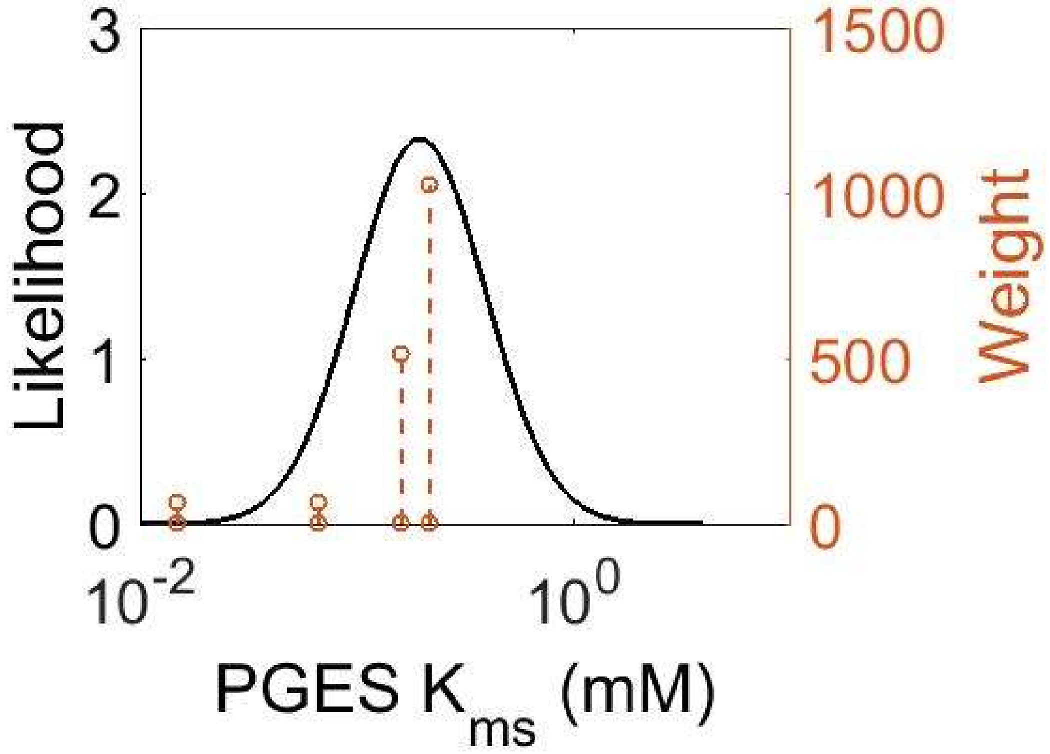


**Table SF.7.5.1.1.1.** The estimated probability distribution for PGES $K_{ms}$. The value and weight of the literature values used to define the distribution are indicated by an orange dashed line. The x axis is plotted on a log-scale.

### S.7.5.1.2. Parameter: PGES $K_{mp}$ (Dependent Parameter)

The log-normal distribution for the parameter for the $K_{mp}$ of Reaction 10 was calculated using multivariate distributions. The log-normal distribution properties for the $K_{mp}$ of Reaction 10 are shown in Table ST.7.5.1.2.1 and plotted in Figure SF.7.5.1.2.1.

**Table ST.7.5.1.2.1.** The log-normal distribution properties of the PGES $K_{mp}$ distribution. These values were calculated using the functions described in Section 2.6.2.

| Mode (mM) | Location parameter (μ) | Scale parameter (σ) |
|---|---|---|
| 1.93 $x10^{-1}$ | -1.15 | 7.02 $x10^{-1}$ |

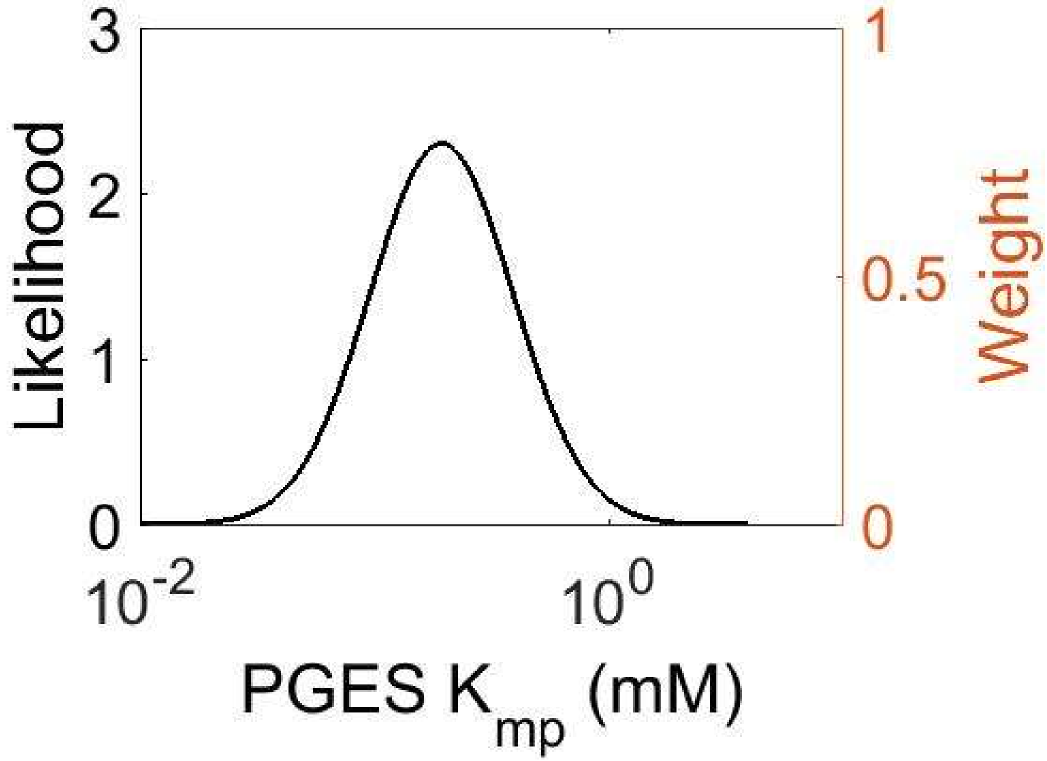


**Figure SF.7.5.1.2.1.** The estimated probability distribution for PGES $K_{mp}$. The value and weight of the literature values used to define the distribution are indicated by an orange dashed line. The x axis is plotted on a log-scale.

### S.7.5.1.3. Parameter: PGES $k_{cat}$

Parameter values for the $k_{cat}$ of Reaction 10 were obtained from the literature and summarised in Table ST.7.5.1.3.1. The log-normal distribution properties for the $k_{cat}$ of Reaction 10 are shown in Table ST.7.5.1.3.2 and plotted in Figure SF.7.5.1.3.1.

**Table ST.7.5.1.3.1.** Literature information used to design the PGES $k_{cat}$ parameter distribution. Each value was assigned a weight using the protocol described in Section 2.6.2. The type of error was assigned as 0 if it was additive and 1 if it was multiplicative.

| Value (min$^{-1}$) | Error (min$^{-1}$) | Experimental details | | | | | | Weight | Type of error | Reference |
|---|---|---|---|---|---|---|---|---|---|---|
| | | Species | Expression Vector | Enzyme | pH | Temperature (°C) | Other | | | |
| 3.00 x10$^{3}$ | 3.60 x10$^{2}$ | Human | E. coli. | PGES (mPGES-1) | 7.5 | 37 | | 1024 | 0 | (Pettersson et al., 2005) |

**Table ST.7.5.1.3.2.** The log-normal distribution properties of the PGES $k_{cat}$ distribution. These values were calculated using the functions described in Section 2.6.2.

| Mode (min$^{-1}$) | Confidence Interval | Location parameter (μ) | Scale parameter (σ) |
|---|---|---|---|
| 2.98 x10$^{3}$ | 1.13 | 8.01 | 1.19 x10$^{-1}$ |

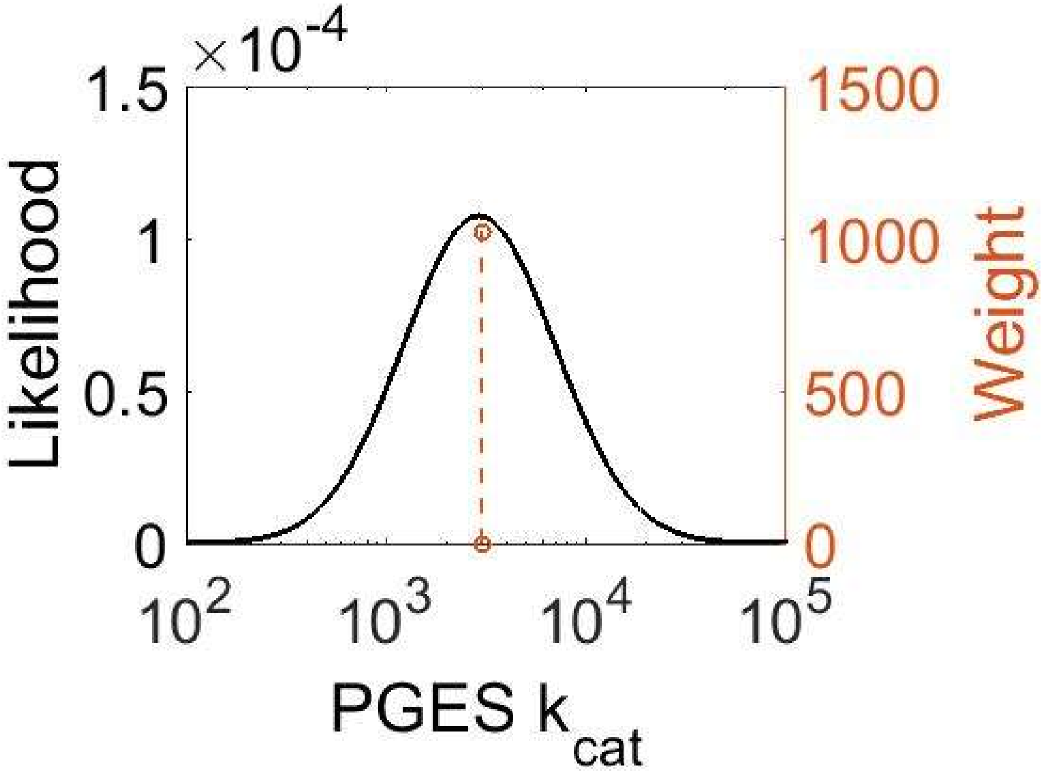


**Figure SF.7.5.1.3.1.** The estimated probability distribution for PGES $k_{cat}$. The value and weight of the literature values used to define the distribution are indicated by an orange dashed line. The x axis is plotted on a log-scale.

#### S.7.5.1.4. Parameter: PGES $K_{eq}$

Parameter values for the $K_{eq}$ of Reaction 10 were obtained from the literature and summarised in Table ST.7.5.1.4.1. The log-normal distribution properties for the $K_{eq}$ of Reaction 10 are shown in Table ST.7.5.1.4.2 and plotted in Figure SF.7.5.1.4.1.

**Table ST.7.5.1.4.1.** Literature information used to design the PGES $K_{eq}$ parameter distribution. Each value was assigned a weight using the protocol described in Section 2.6.2. The type of error was assigned as 0 if it was additive and 1 if it was multiplicative.

| ΔG (kcal/mol) | $K_{eq}$ | Experimental details | | | | | | Weight | Type of error | Reference |
|---|---|---|---|---|---|---|---|---|---|---|
| | | Species | Expression Vector | Enzyme | pH | Temperature (°C) | Other | | | |
| -6.64 | $7.46 \times 10^4$ | Human | Unknown | PGES | 7 | Unknown | | 64 | 0 | (Caspi et al., 2018) |

**Table ST.7.5.1.4.2.** The log-normal distribution properties of the PGES $K_{eq}$ distribution. These values were calculated using the functions described in Section 2.6.2.

| Mode | Confidence Interval | Location parameter (μ) | Scale parameter (σ) |
|---|---|---|---|
| $7.46 \times 10^4$ | $1.00 \times 10^1$ | $1.20 \times 10^1$ | $8.90 \times 10^{-1}$ |

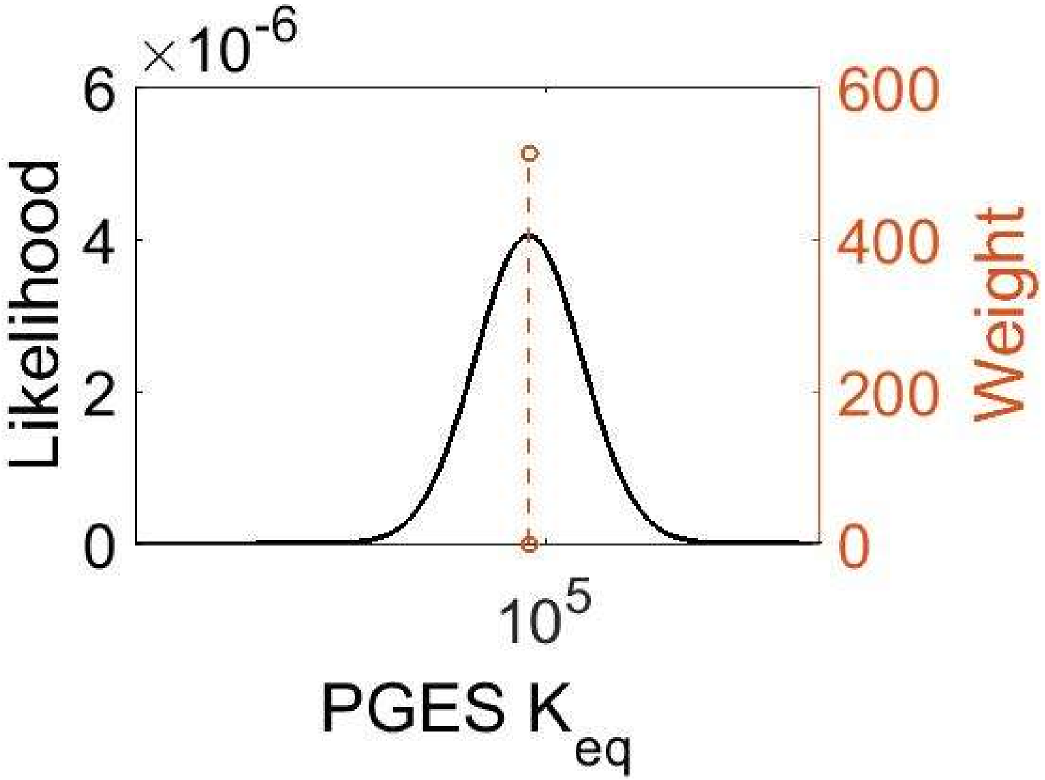


**Figure SF.7.5.1.4.1.** The estimated probability distribution for PGES $K_{eq}$. The value and weight of the literature values used to define the distribution are indicated by an orange dashed line. The x axis is plotted on a log-scale.

### S.7.5.1.5. Parameter: PGES concentration

Parameter values for the PGES concentration of Reaction 10 were obtained from the literature and summarised in Table ST.7.5.1.5.1. The log-normal distribution properties for the PGES concentration of Reaction 10 are shown in Table ST.7.5.1.5.2 and plotted in Figure SF.7.5.1.5.1. To convert the enzyme concentration from ppm to mM, **Equation S.6.2** was used.

**Table ST.7.5.1.5.1.** Literature information used to design the PGES $K_{eq}$ parameter distribution. Each value was assigned a weight using the protocol described in Section 2.6.2. The type of error was assigned as 0 if it was additive and 1 if it was multiplicative.

| Value (ppm) | Error (ppm) | Experimental details | | | | | | Weight | Type of error | Reference |
|---|---|---|---|---|---|---|---|---|---|---|
| | | Species | Expression Vector | Enzyme | pH | Temperature (°C) | Other | | | |
| $1.16 \times 10^{1}$ | NaN | Human | Colon | PGES | 7.5 | 37 | | **1024** | 0 | (Kim et al., 2014) |
| $2.81 \times 10^{1}$ | NaN | Human | Lung | PGES | 7.5 | 37 | | **1024** | 0 | (Kim et al., 2014) |
| $7.53 \times 10^{1}$ | NaN | Human | Urinary bladder | PGES | 7.5 | 37 | | **1024** | 0 | (Kim et al., 2014) |
| $2.08 \times 10^{2}$ | NaN | Human | Stomach | PGES | 7.5 | 37 | | **1024** | 0 | (Wilhelm et al., 2014) |
| $2.20 \times 10^{2}$ | NaN | Human | Placenta | PGES | 7.5 | 37 | | **1024** | 0 | (Wilhelm et al., 2014) |

**Table ST.7.5.1.5.2** The log-normal distribution properties of the PGES concentration distribution. These values were calculated using the functions described in Section 2.6.2.

| Mode (ppm) | Mode (mM) | Confidence Interval | Location parameter (μ) | Scale parameter (σ) |
|---|---|---|---|---|
| $7.49 \times 10^{1}$ | $4.15 \times 10^{-4}$ | 3.15 | 5.03 | $8.44 \times 10^{-1}$ |

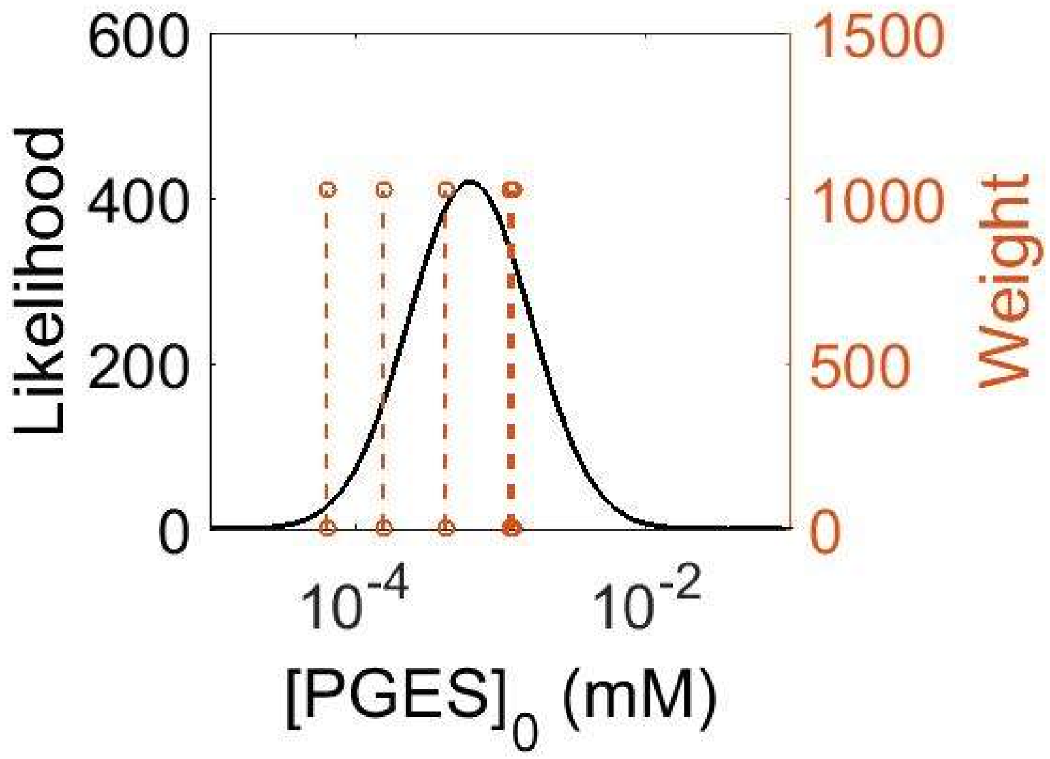


**Figure SF.7.5.1.5.1.** The estimated probability distribution for the PGES concentration. The value and weight of the literature values used to define the distribution are indicated by an orange dashed line. The x axis is plotted on a log-scale.

### S.7.6 Reaction 11: AA ⇌ 5-HPETE

The gene ALOX5 encodes the protein 5-LOX, which is responsible for the generation of 5-HPETE from AA. The formation of the hydroperoxy fatty acids (HPETE) begins with the abstraction of a hydrogen radical at the allylic position between two double bonds. The structure undergoes a rearrangement reaction which results in the formation of a conjugated diene system. The insertion of molecular oxygen and a hydrogen leads to the formation of the final structure, a hydroperoxy fatty acid.

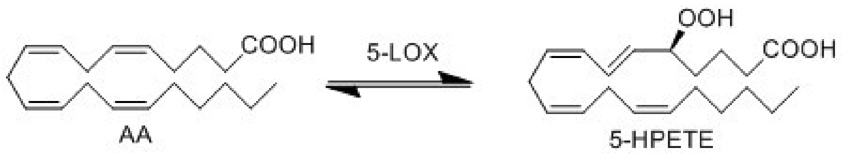


**Figure SF.7.6.** The metabolism of arachidonic acid (AA) into 5-hydroperoxyeicosatetraenoic acid (5-HPETE) by 5-lipoxygenase (5-LOX) (Reaction 11).

**SEq.7.6.** Reaction rate law for Reaction 11.

$$\boldsymbol{v_{11}} = \frac{K_{cat} \cdot [5-LOX]\left([AA] - {[5-HPETE]}/{K_{eq}}\right)}{K_{m_s}\left(1 + \frac{[5-HPETE]}{K_{m_p}}\right) + [AA]}$$

### S.7.6.1. Reaction parameters

#### S.7.6.1.1. Parameter: 5-LOX $K_{ms}$

Parameter values for the $K_{ms}$ of Reaction 11 were obtained from the literature and summarised in Table ST.7.6.1.1.1. The log-normal distribution properties for the $K_{ms}$ of Reaction 11 are shown in Table ST.7.6.1.1.2 and plotted in Figure SF.7.6.1.1.1.

**Table ST.7.6.1.1.1.** Literature information used to design the 5-LOX $K_{ms}$ parameter distribution. Each value was assigned a weight using the protocol described in Section 2.6.2. The type of error was assigned as 0 if it was additive and 1 if it was multiplicative.

| Value (mM) | Error (mM) | Experimental details | | | | | | Weight | Type of error | Reference |
|---|---|---|---|---|---|---|---|---|---|---|
| | | Species | Expression Vector | Enzyme | pH | Temperature (°C) | Other | | | |
| 5.10 $\times 10^{-3}$ | NaN | Human | Baculovirus | 5-LOX | 5.6 | 37 | | 256 | 0 | (Shirumalla et al., 2006) |
| 1.20 $\times 10^{-2}$ | NaN | Human | Polymorphonuclear Leukocytes | 5-LOX | 7.5 | 22 | | 512 | 0 | (Soberman, 1988) |
| 6.31 $\times 10^{-2}$ | NaN | Human | Polymorphonuclear Leukocytes | 5-LOX | 7.5 | 22 | | 512 | 0 | (Soberman et al., 1985) |

**Table ST.7.6.1.1.2.** The log-normal distribution properties of the 5-LOX $K_{ms}$ distribution. These values were calculated using the functions described in Section 2.6.2.

| Mode (mM) | Confidence Interval | Location parameter (μ) | Scale parameter (σ) |
|---|---|---|---|
| 1.27 $\times 10^{-2}$ | 2.74 | -3.76 | 7.73 $\times 10^{-1}$ |

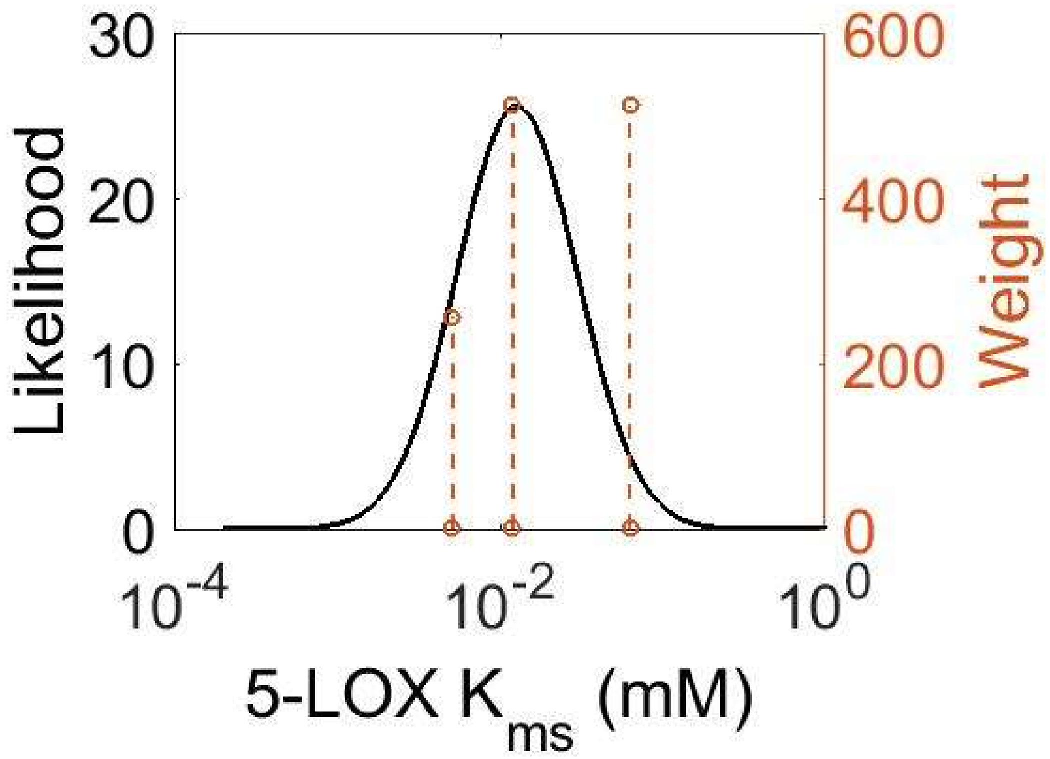


**Figure SF.7.6.1.1.1.** The estimated probability distribution for 5-LOX $K_{ms}$. The value and weight of the literature values used to define the distribution are indicated by an orange dashed line. The x axis is plotted on a log-scale.

**S.7.6.1.2. Parameter: 5-LOX $K_{mp}$ (Dependent parameter)**

The log-normal distribution for the parameter for the $K_{mp}$ of Reaction 11 was calculated using multivariate distributions. The log-normal distribution properties for the $K_{mp}$ of Reaction 11 are shown in Table ST.7.6.1.2.1 and plotted in Figure SF.7.6.1.2.1.

**Table ST.7.6.1.2.1.** The log-normal distribution properties of the 5-LOX $K_{mp}$ distribution. These values were calculated using the functions described in Section 2.6.2.

| **Mode (mM)** | **Location parameter (μ)** | **Scale parameter (σ)** |
|---|---|---|
| 1.25 $x10^{-2}$ | -3.63 | 8.68 $x10^{-1}$ |

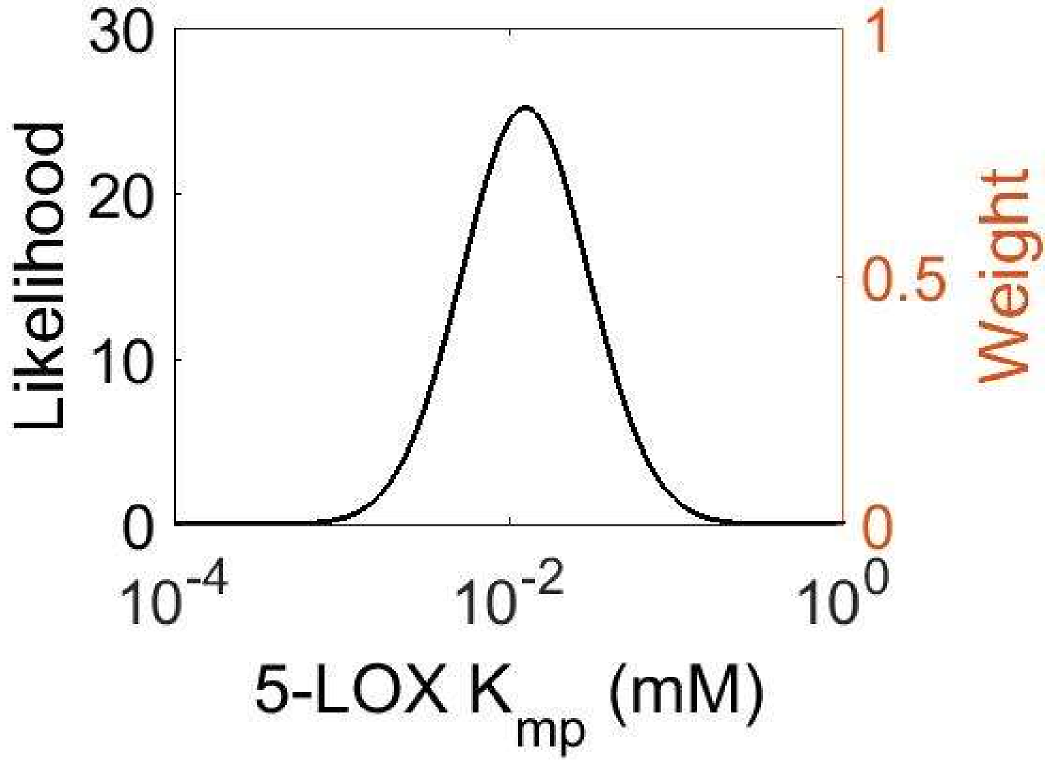


**Figure SF.7.6.1.2.1.** The estimated probability distribution for 5-LOX $K_{mp}$. The value and weight of the literature values used to define the distribution are indicated by an orange dashed line. The x axis is plotted on a log-scale.

## S.7.6.1.3. Parameter: 5-LOX $k_{cat}$

Parameter values for the $k_{cat}$ of Reaction 11 were obtained from the literature and summarised in Table ST.7.6.1.3.1. The log-normal distribution properties for the $k_{cat}$ of Reaction 11 are shown in Table ST.7.6.1.3.2 and plotted in Figure SF.7.6.1.3.1.

**Table ST.7.6.1.3.1.** Literature information used to design the 5-LOX $k_{cat}$ parameter distribution. Each value was assigned a weight using the protocol described in Section 2.6.2. The type of error was assigned as 0 if it was additive and 1 if it was multiplicative.

| Value ($min^{-1}$) | Error ($min^{-1}$) | Experimental details | | | | | | Weight | Type of error | Reference |
|---|---|---|---|---|---|---|---|---|---|---|
| | | Species | Expression Vector | Enzyme | pH | Temperature (°C) | Other | | | |
| 1.50 $x10^3$ | 7.50 $x10^1$ | Potato | Potato | 5-LOX | 5.5 | 23 | | 16 | 0 | (Mulliez et al., 1987) |

**Table ST.7.6.1.3.2.** The log-normal distribution properties of the 5-LOX $k_{cat}$ distribution. These values were calculated using the functions described in Section 2.6.2.

| Mode ($min^{-1}$) | Confidence Interval | Location parameter (μ) | Scale parameter (σ) |
|---|---|---|---|
| 1.50 $x10^3$ | 1.05 | 7.31 | 4.99 $x10^{-2}$ |

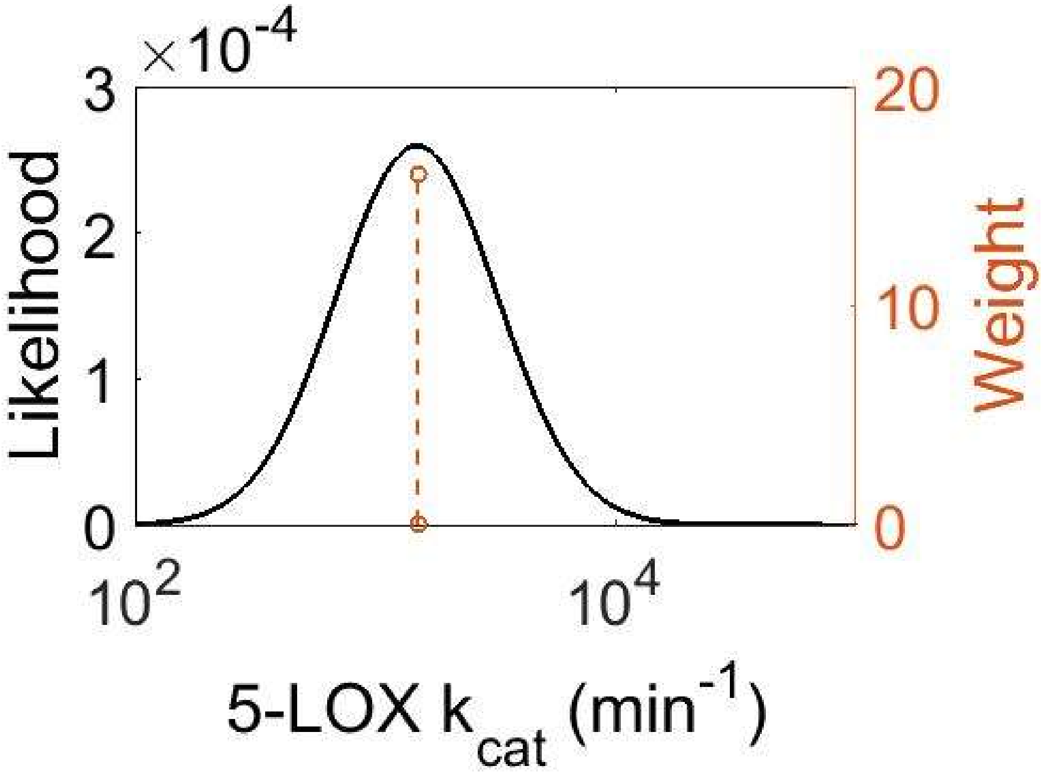


**Figure SF.7.6.1.3.1.** The estimated probability distribution for 5-LOX $k_{cat}$. The value and weight of the literature values used to define the distribution are indicated by an orange dashed line. The x axis is plotted on a log-scale.

### S.7.6.1.4. Parameter: 5-LOX $K_{eq}$

Parameter values for the $K_{eq}$ of Reaction 11 were obtained from the literature and summarised in Table ST.7.6.1.4.1. The log-normal distribution properties for the $K_{eq}$ of Reaction 11 are shown in Table ST.7.6.1.4.2 and plotted in Figure SF.7.6.1.4.1.

**Table ST.7.6.1.4.1.** Literature information used to design the 5-LOX $K_{eq}$ parameter distribution. Each value was assigned a weight using the protocol described in Section 2.6.2. The type of error was assigned as 0 if it was additive and 1 if it was multiplicative.

| ΔG (kcal/mol) | $K_{eq}$ | Experimental details | | | | | | Weight | Type of error | Reference |
|---|---|---|---|---|---|---|---|---|---|---|
| | | Species | Expression Vector | Enzyme | pH | Temperature (°C) | Other | | | |
| -7.00 x10$^{1}$ | 2.27 x10$^{51}$ | Human | Unknown | 5-LOX | 7 | Unknown | | 64 | 0 | (Caspi et al., 2018) |

**Table ST.7.6.1.4.2.** The log-normal distribution properties of the 5-LOX $K_{eq}$ distribution. These values were calculated using the functions described in Section 2.6.2.

| Mode | Confidence Interval | Location parameter (μ) | Scale parameter (σ) |
|---|---|---|---|
| 2.27 x10$^{51}$ | 1.00 x10$^{1}$ | 1.19 x10$^{2}$ | 8.90 x10$^{-1}$ |

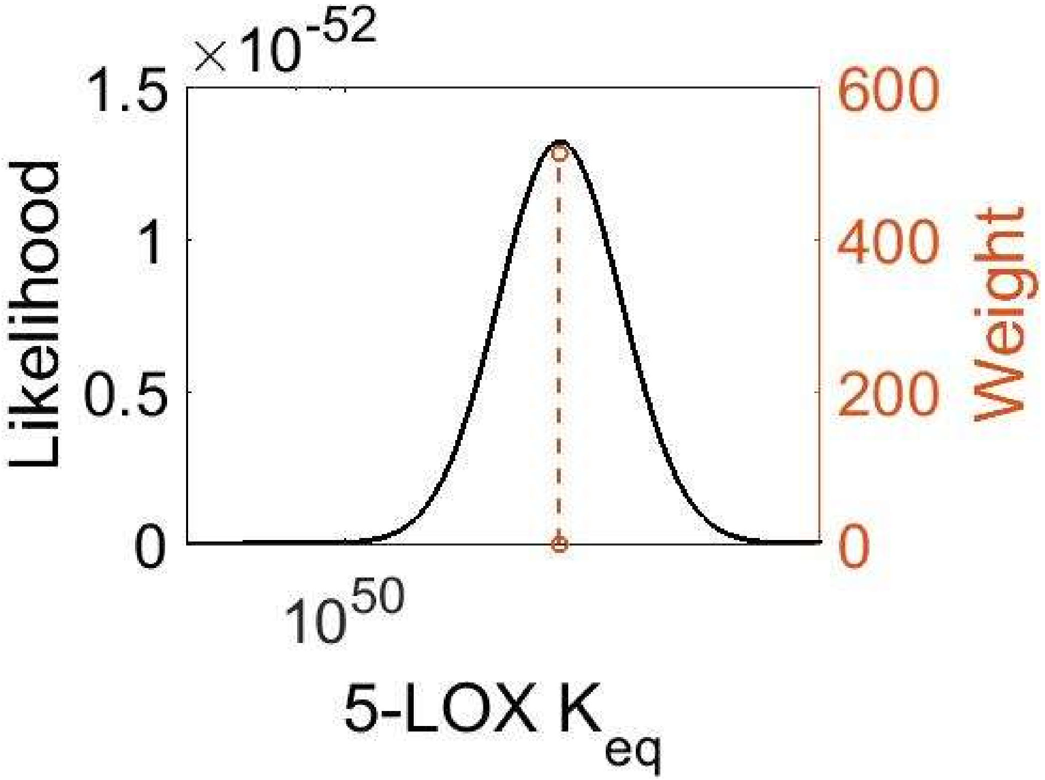


**Figure SF.7.6.1.4.1.** The estimated probability distribution for 5-LOX $K_{eq}$. The value and weight of the literature values used to define the distribution are indicated by an orange dashed line. The x axis is plotted on a log-scale.

### S.7.6.1.5. Parameter: 5-LOX concentration

Parameter values for the 5-LOX concentration of Reaction 11 were obtained from the literature and summarised in Table ST.7.6.1.5.1. The log-normal distribution properties for the 5-LOX concentration of Reaction 11 are shown in Table ST.7.6.1.5.2 and plotted in Figure SF.7.6.1.5.1. To convert the enzyme concentration from ppm to mM, **Equation S.6.2** was used.

**Table ST.7.6.1.5.1.** Literature information used to design the 5-LOX concentration parameter distribution. Each value was assigned a weight using the protocol described in Section 2.6.2. The type of error was assigned as 0 if it was additive and 1 if it was multiplicative.

| Value (ppm) | Error (ppm) | Experimental details | | | | | | Weight | Type of error | Reference |
|---|---|---|---|---|---|---|---|---|---|---|
| | | Species | Expression Vector | Enzyme | pH | Temperature (°C) | Other | | | |
| 3.19 $x10^{1}$ | NaN | Human | Oral cavity | 5-LOX | 7.5 | 37 | | **1024** | 0 | (Wilhelm et al., 2014) |
| 4.98 $x10^{1}$ | NaN | Human | Oesophagus | 5-LOX | 7.5 | 37 | | **1024** | 0 | (Wilhelm et al., 2014) |
| 9.73 $x10^{1}$ | NaN | Human | Lung | 5-LOX | 7.5 | 37 | | **1024** | 0 | (Kim et al., 2014) |

**Table ST.7.6.1.5.2.** The log-normal distribution properties of the 5-LOX concentration distribution. These values were calculated using the functions described in Section 2.6.2.

| Mode (ppm) | Mode (mM) | Confidence Interval | Location parameter (μ) | Scale parameter (σ) |
|---|---|---|---|---|
| 4.96 $x10^{1}$ | 2.74 $x10^{-4}$ | 1.60 | 4.09 | 4.28 $x10^{-1}$ |

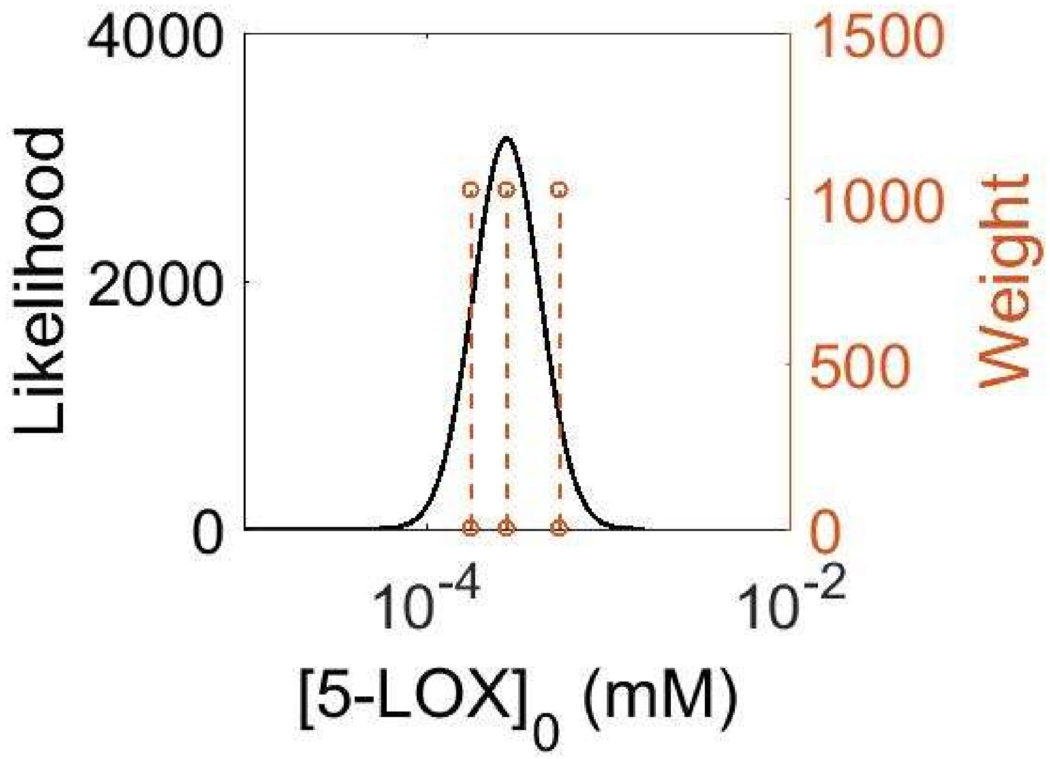


**Figure SF.7.6.1.5.1.** The estimated probability distribution for the 5-LOX concentration The value and weight of the literature values used to define the distribution are indicated by an orange dashed line. The x axis is plotted on a log-scale.

### S.7.7 Reaction 12: 5-HPETE ⇌ 5-HETE

Upon being generated, 5-HPETE is reduced by an oxidoreductase enzyme, phospholipid hydroperoxide glutathione peroxidase (PHGPx), to form 5-HETE.

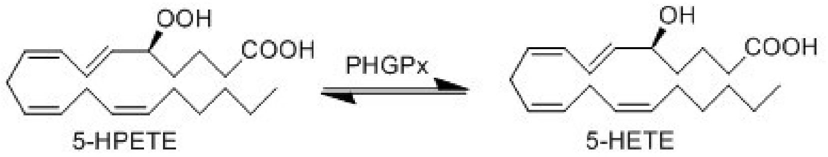


**Figure SF.7.7.** The metabolism of 5-hydroperoxyeicosatetraenoic acid (5-HPETE) into 5-hydroxyeicosatetraenoic acid (5-HETE) by phospholipid hydroperoxide glutathione peroxidase (PHGPx) (Reaction 12).

**SEq.7.7.** Reaction rate law for Reaction 12.

$$\boldsymbol{v_{12}} = \frac{K_{cat} \cdot [PHGPx]\left([5-HPETE5] - {[5-HETE]}/{K_{eq}}\right)}{K_{m_s}\left(1 + \frac{[5-HETE]}{K_{m_p}}\right) + [5-HPETE]}$$

### S.7.7.1. Reaction parameters

#### S.7.7.1.1. Parameter: PHGPx $K_{ms}$

Parameter values for the $K_{ms}$ of Reaction 12 were obtained from the literature and summarised in Table ST.7.7.1.1.1. The log-normal distribution properties for the $K_{ms}$ of Reaction 12 are shown in Table ST.7.7.1.1.2 and plotted in Figure SF.7.7.1.1.1.

**Table ST.7.7.1.1.1.** Literature information used to design the PHGPx $K_{ms}$ parameter distribution. Each value was assigned a weight using the protocol described in Section 2.6.2. The type of error was assigned as 0 if it was additive and 1 if it was multiplicative.

| Value (mM) | Error (mM) | Experimental details | | | | | | Weight | Type of error | Reference |
|---|---|---|---|---|---|---|---|---|---|---|
| | | Species | Expression Vector | Enzyme | pH | Temperature (°C) | Other | | | |
| $4.00 \times 10^{-3}$ | NaN | Rat | Liver | PHGPx | 7.4 | 37 | | 768 | 0 | (Hiratsuka et al., 1997) |
| $3.00 \times 10^{-1}$ | NaN | Human | Bio imprinted Enzyme | PHGPx | 7 | 37 | | 512 | 0 | (Liu et al., 2008) |
| $1.11 \times 10^{1}$ | $2.90 \times 10^{-1}$ | Human | E. coli. | PHGPx | 7 | 37 | | 1024 | 0 | (Zheng et al., 2008) |

**Table ST.7.7.1.1.2.** The log-normal distribution properties of the PHGPx $K_{ms}$ distribution. These values were calculated using the functions described in Section 2.6.2.

| Mode (mM) | Confidence Interval | Location parameter (μ) | Scale parameter (σ) |
|---|---|---|---|
| $3.19 \times 10^{-1}$ | $1.02 \times 10^{3}$ | 2.54 | 1.92 |

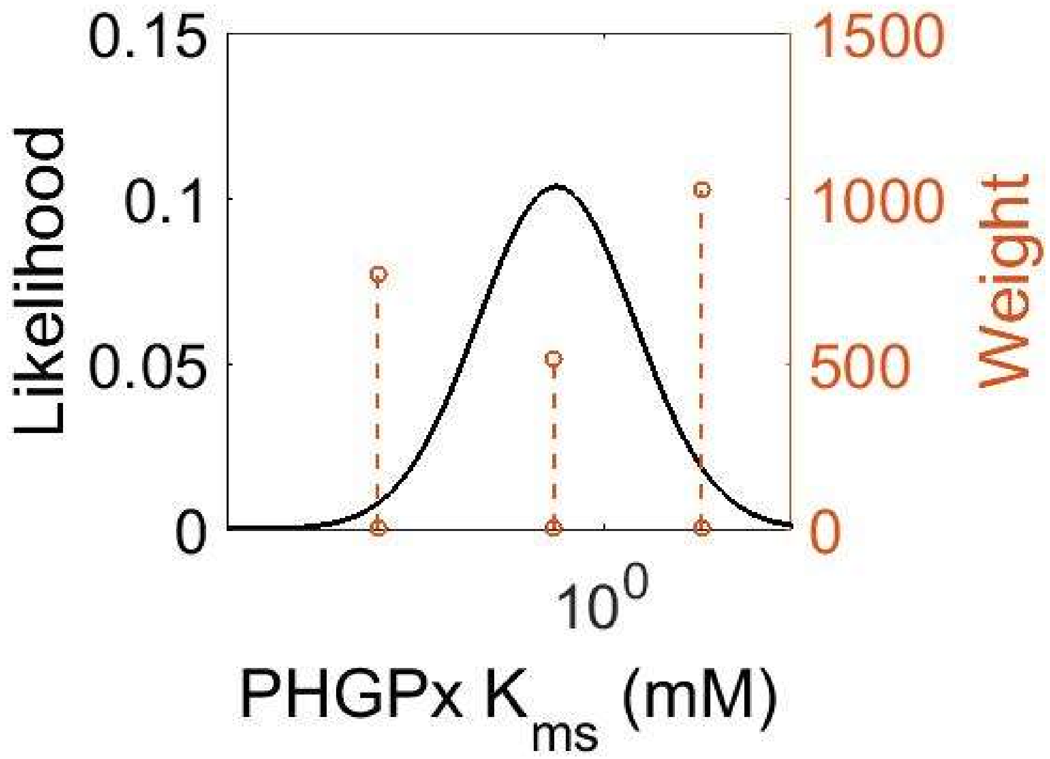


**Figure SF.7.7.1.1.1.** The estimated probability distribution for PHGPx $K_{ms}$. The value and weight of the literature values used to define the distribution are indicated by an orange dashed line. The x axis is plotted on a log-scale.

### S.7.7.1.2. Parameter: PHGPx $K_{mp}$ (Dependent parameter)

The log-normal distribution for the parameter for the $K_{mp}$ of Reaction 12 was calculated using multivariate distributions. The log-normal distribution properties for the $K_{mp}$ of Reaction 12 are shown in Table ST.7.7.1.2.1 and plotted in Figure SF.7.7.1.2.1.

**Table ST.7.7.1.2.1.** The log-normal distribution properties of the PHGPx $K_{mp}$ distribution. These values were calculated using the functions described in Section 2.6.2.

| Mode (mM) | Location parameter (μ) | Scale parameter (σ) |
|---|---|---|
| $3.15 \times 10^{-1}$ | 2.53 | 1.92 |

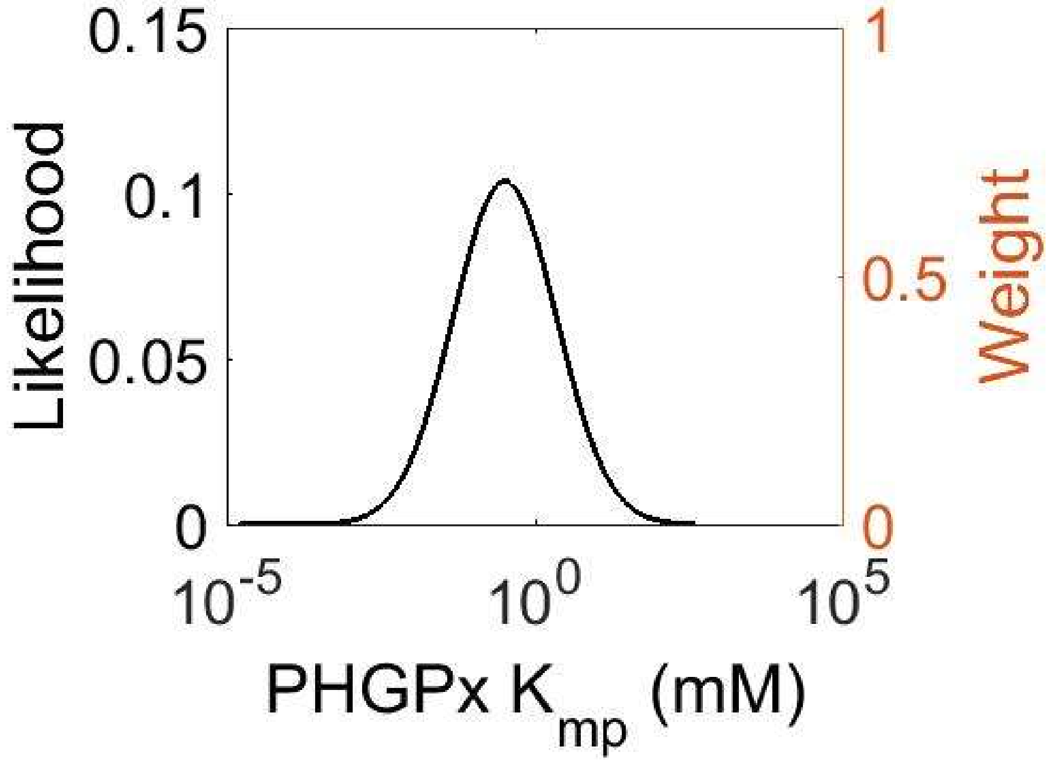


**Figure SF.7.7.1.2.1.** The estimated probability distribution for PHGPx $K_{mp}$. The value and weight of the literature values used to define the distribution are indicated by an orange dashed line. The x axis is plotted on a log-scale.

#### S.7.7.1.3. Parameter: PHGPx $k_{cat}$

Parameter values for the $k_{cat}$ of Reaction 12 were obtained from the literature and summarised in Table ST.7.7.1.3.1. The log-normal distribution properties for the $k_{cat}$ of Reaction 12 are shown in Table ST.7.7.1.3.2 and plotted in Figure SF.7.7.1.3.1.

**Table ST.7.7.1.3.1.** Literature information used to design the PHGPx $k_{cat}$ parameter distribution. Each value was assigned a weight using the protocol described in Section 2.6.2. The type of error was assigned as 0 if it was additive and 1 if it was multiplicative.

| Value ($min^{-1}$) | Error ($min^{-1}$) | Experimental details | | | | | | Weight | Type of error | Reference |
|---|---|---|---|---|---|---|---|---|---|---|
| | | Species | Expression Vector | Enzyme | pH | Temperature (°C) | Other | | | |
| 1.17 x$10^3$ | 5.00 x$10^1$ | Human | Bio imprinted Enzyme | PHGPx | 7 | 37 | | 512 | 0 | (Liu et al., 2008) |
| 2.45 x$10^4$ | 1.50 x$10^2$ | Human | E. coli. | PHGPx | 7 | 37 | | 1024 | 0 | (Zheng et al., 2008) |

**Table ST.7.7.1.3.2.** The log-normal distribution properties of the PHGPx $k_{cat}$ distribution. These values were calculated using the functions described in Section 2.6.2.

| Mode ($min^{-1}$) | Confidence Interval | Location parameter (μ) | Scale parameter (σ) |
|---|---|---|---|
| 2.44 x$10^4$ | 4.20 | 1.11 x$10^1$ | 9.75 x$10^{-1}$ |

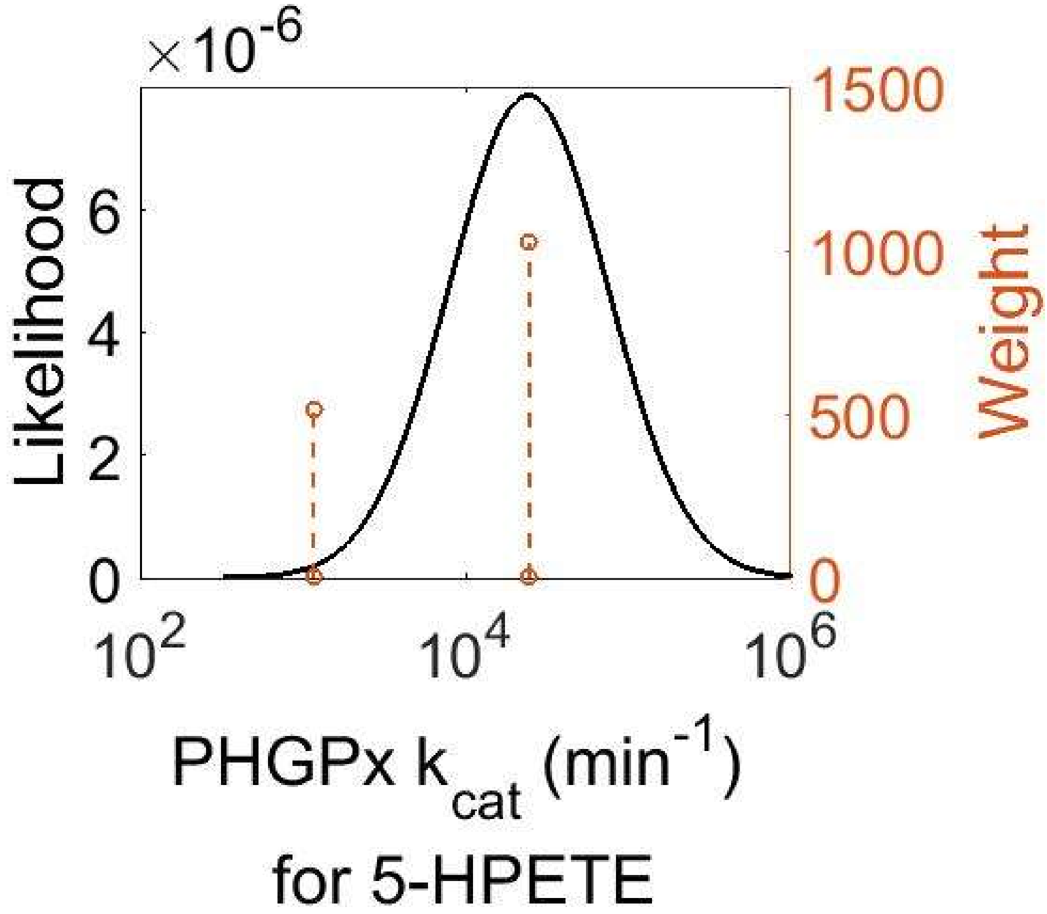


**Figure SF.7.7.1.3.1.** The estimated probability distribution for PHGPx $k_{cat}$. The value and weight of the literature values used to define the distribution are indicated by an orange dashed line. The x axis is plotted on a log-scale.

### S.7.7.1.4. Parameter: PHGPx $K_{eq}$

Parameter values for the $K_{eq}$ of Reaction 12 were obtained from the literature and summarised in Table ST.7.7.1.4.1. The log-normal distribution properties for the $K_{eq}$ of Reaction 12 are shown in Table ST.7.7.1.4.2 and plotted in Figure SF.7.7.1.4.1.

**Table ST.7.7.1.4.1.** Literature information used to design the PHGPx $K_{eq}$ parameter distribution. Each value was assigned a weight using the protocol described in Section 2.6.2. The type of error was assigned as 0 if it was additive and 1 if it was multiplicative.

| ΔG (kcal/mol) | $K_{eq}$ | Experimental details | | | | | | Weight | Type of error | Reference |
|---|---|---|---|---|---|---|---|---|---|---|
| | | Species | Expression Vector | Enzyme | pH | Temperature (°C) | Other | | | |
| $-2.69 \times 10^{1}$ | $5.90 \times 10^{19}$ | Human | Unknown | PHGPx | 7 | Unknown | | 64 | 0 | (Caspi et al., 2018) |

**Table ST.7.7.1.4.2.** The log-normal distribution properties of the PHGPx $K_{eq}$ distribution. These values were calculated using the functions described in Section 2.6.2.

| Mode | Confidence Interval | Location parameter (μ) | Scale parameter (σ) |
|---|---|---|---|
| $5.90 \times 10^{19}$ | $1.00 \times 10^{1}$ | 4.63 x101 | $8.90 \times 10^{-1}$ |

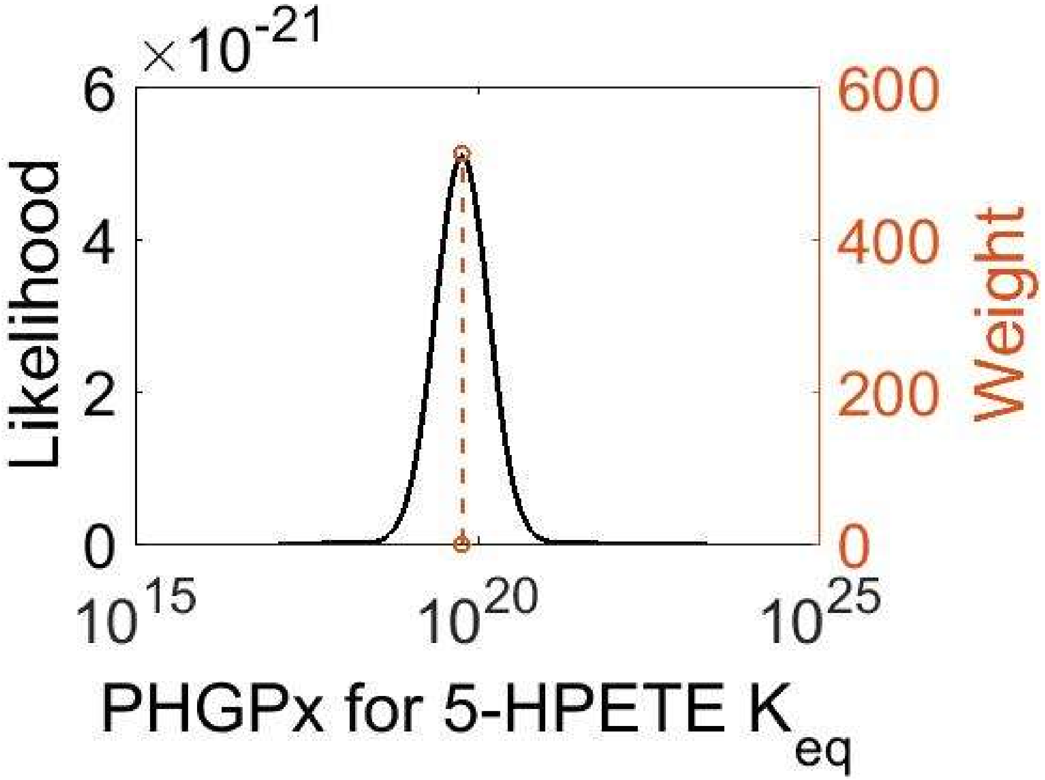


**Figure SF.7.7.1.4.1.** The estimated probability distribution for PHGPx $K_{eq}$. The value and weight of the literature values used to define the distribution are indicated by an orange dashed line. The x axis is plotted on a log-scale.

### S.7.7.1.5. Parameter: PHGPx concentration

Parameter values for the PHGPx concentration of Reaction 12 were obtained from the literature and summarised in Table ST.7.7.1.5.1. The log-normal distribution properties for the PHGPx concentration of Reaction 12 are shown in Table ST.7.7.1.5.2 and plotted in Figure SF.7.7.1.5.1. To convert the enzyme concentration from ppm to mM, **Equation S.6.2** was used.

**Table ST.7.7.1.5.1.** Literature information used to design the PHGPx concentration parameter distribution. Each value was assigned a weight using the protocol described in Section 2.6.2. The type of error was assigned as 0 if it was additive and 1 if it was multiplicative.

| Value (ppm) | Error (ppm) | Experimental details | | | | | | Weight | Type of error | Reference |
|---|---|---|---|---|---|---|---|---|---|---|
| | | Species | Expression Vector | Enzyme | pH | Temperature (°C) | Other | | | |
| 2.04 $\times 10^2$ | NaN | Human | Gut | PHGPx | 7.5 | 37 | | **1024** | 0 | (Kim et al., 2014) |
| 2.82 $\times 10^2$ | NaN | Human | Oesophagus | PHGPx | 7.5 | 37 | | **1024** | 0 | (Wilhelm et al., 2014) |
| 3.07 $\times 10^2$ | NaN | Human | Lung | PHGPx | 7.5 | 37 | | **1024** | 0 | (Wilhelm et al., 2014) |
| 4.59 $\times 10^2$ | NaN | Human | Skin | PHGPx | 7.5 | 37 | | **2048** | 0 | (Wilhelm et al., 2014) |

**Table ST.7.7.1.5.2.** The log-normal distribution properties of the PHGPx concentration distribution. These values were calculated using the functions described in Section 2.6.2.

| Mode (ppm) | Mode (mM) | Confidence Interval | Location parameter (μ) | Scale parameter (σ) |
|---|---|---|---|---|
| 3.14 $\times 10^2$ | 1.74$\times 10^{-3}$ | 1.38 | 5.85 | 3.09 $\times 10^{-1}$ |

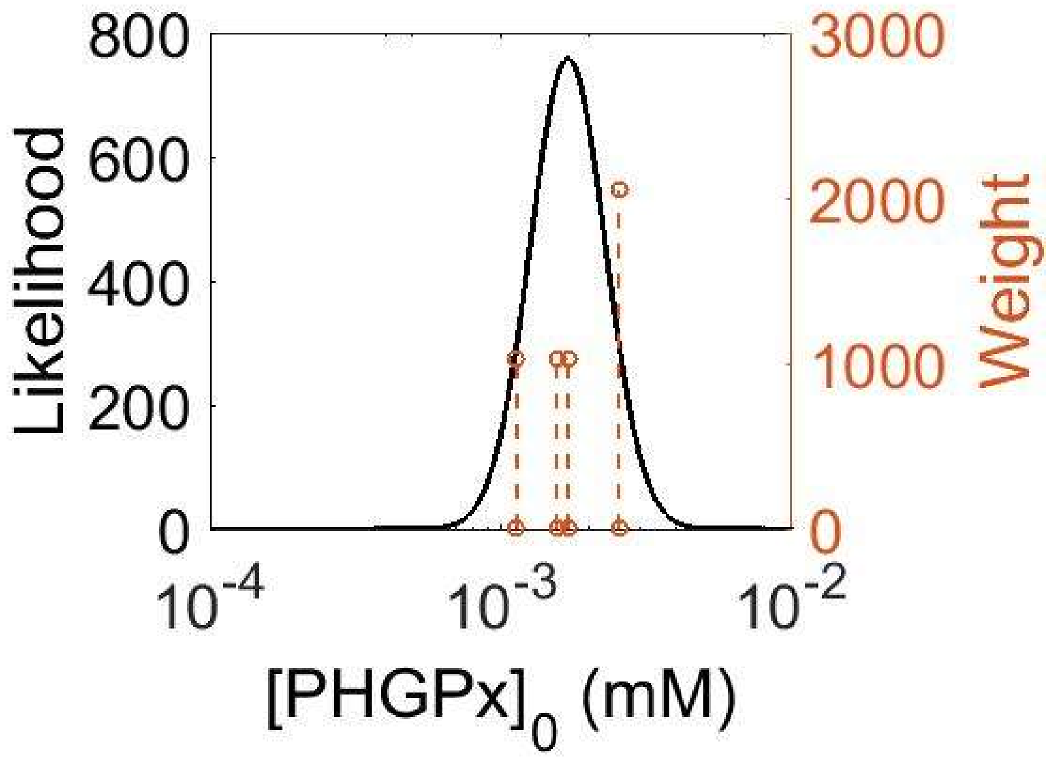


**Figure SF.7.7.1.5.1.** The estimated probability distribution for the PHGPx concentration. The value and weight of the literature values used to define the distribution are indicated by an orange dashed line. The x axis is plotted on a log-scale.

## S.7.8 Reaction 13: 5-HPETE ⇌ $LTA_4$

5-HPETE is further metabolised by 5-LOX. This dehydration reaction converts the peroxide functional group of 5(S)-HPETE to an epoxide functional group, generating $LTA_4$ (Shimizu et al., 1984). 5-LOX performs this reaction by abstracting the pro-R hydrogen at C10 and rearranging the structure so that the radical relocates to C6. The double bonds within the structure then rearrange to form a conjugated triene system and an epoxide. This reaction is promoted when 5-LOX colocalises with 5-lipoxygenase-activating protein (FLAP) on the nuclear membrane or the ER (Abramovitz et al., 1993; Brock et al., 1994).

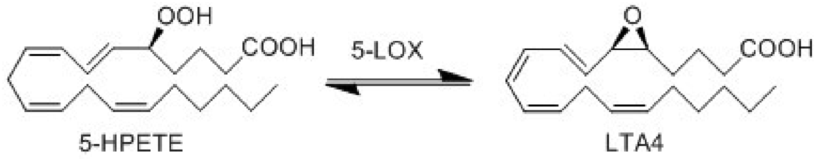


**Figure SF.7.8.** The metabolism of 5-hydroperoxyeicosatetraenoic acid (5-HPETE) into leukotriene $A_4$ ($LTA_4$) by 5-lipoxygenase/5-lipoxygenase-activating protein (5-LOX/FLAP) (Reaction 13).

**SEq.7.8.** Reaction rate law for Reaction 13.

$$v_{13} = \frac{K_{cat} \cdot [5-LOX]\left([5-HPETE] - {[LTA_4]}/{K_{eq}}\right)}{K_{m_s}\left(1 + \frac{[LTA_4]}{K_{m_p}}\right) + [5-HPETE]}$$

### S.7.8.1. Reaction parameters

#### S.7.8.1.1. Parameter: 5-LOX/FLAP $K_{ms}$

Parameter values for the $K_{ms}$ of Reaction 13 were obtained from the literature and summarised in Table ST.7.8.1.1.1. The log-normal distribution properties for the $K_{ms}$ of Reaction 13 are shown in Table ST.7.8.1.1.2 and plotted in Figure SF.7.8.1.1.1.

**Table ST.7.8.1.1.1.** Literature information used to design the 5-LOX/FLAP $K_{ms}$ parameter distribution. Each value was assigned a weight using the protocol described in Section 2.6.2. The type of error was assigned as 0 if it was additive and 1 if it was multiplicative.

| Value (mM) | Error (mM) | Experimental details | | | | | | Weight | Type of error | Reference |
|---|---|---|---|---|---|---|---|---|---|---|
| | | Species | Expression Vector | Enzyme | pH | Temperature (°C) | Other | | | |
| 5.10 $x10^{-3}$ | NaN | Human | Baculovirus | 5-LOX | 5.6 | 37 | | 256 | 0 | (Shirumalla et al., 2006) |
| 1.20 $x10^{-2}$ | NaN | Human | Polymorphonuclear Leukocytes | 5-LOX | 7.5 | 22 | | 512 | 0 | (Soberman, 1988) |
| 6.31 $x10^{-2}$ | NaN | Human | Polymorphonuclear Leukocytes | 5-LOX | 7.5 | 22 | | 512 | 0 | (Soberman et al., 1985) |

**Table ST.7.8.1.1.2.** The log-normal distribution properties of the 5-LOX/FLAP $K_{ms}$ distribution. These values were calculated using the functions described in Section 2.6.2.

| Mode (mM) | Confidence Interval | Location parameter (μ) | Scale parameter (σ) |
|---|---|---|---|
| 1.28 $x10^{-2}$ | 8.70 | -3.64 | 8.50 $x10^{-1}$ |

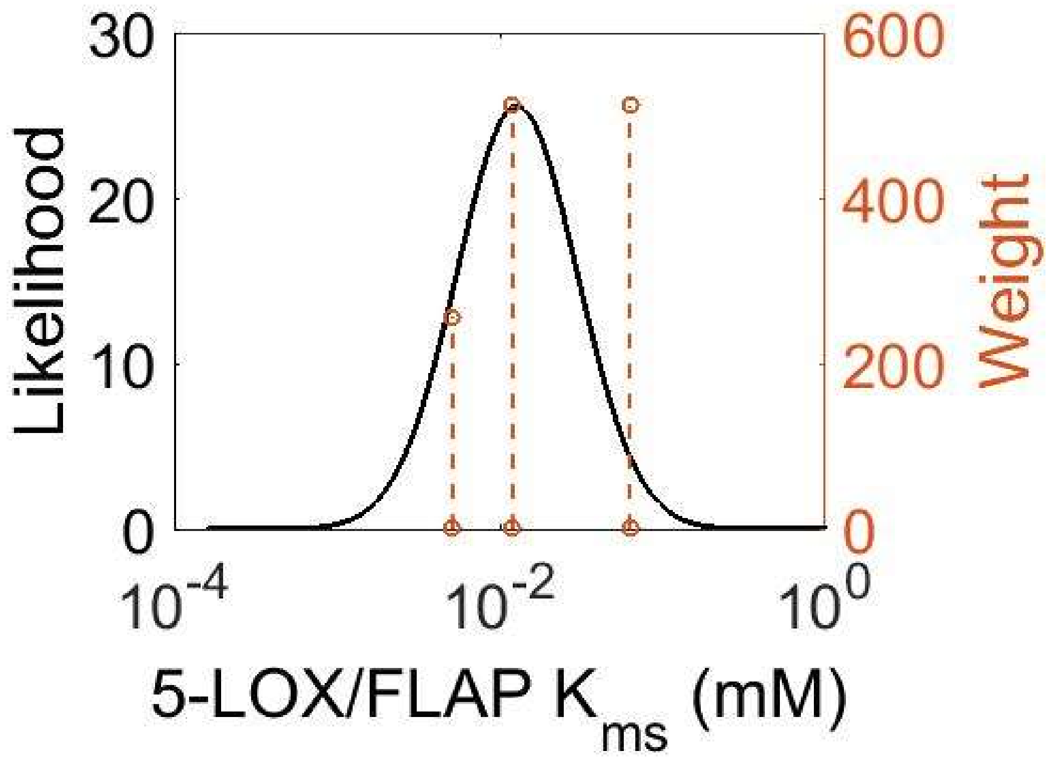


**Figure SF.7.8.1.1.1.** The estimated probability distribution for 5-LOX/FLAP $K_{ms}$. The value and weight of the literature values used to define the distribution are indicated by an orange dashed line. The x axis is plotted on a log-scale.

#### S.7.8.1.2. Parameter: 5-LOX/FLAP $K_{mp}$ (Dependent parameter)

The log-normal distribution for the parameter for the $K_{mp}$ of Reaction 13 was calculated using multivariate distributions. The log-normal distribution properties for the $K_{mp}$ of Reaction 13 are shown in Table ST.7.8.1.2.1 and plotted in Figure SF.7.8.1.2.1.

**Table ST.7.8.1.2.1.** The log-normal distribution properties of the 5-LOX/FLAP $K_{mp}$ distribution. These values were calculated using the functions described in Section 2.6.2.

| Mode (mM) | Location parameter (μ) | Scale parameter (σ) |
|---|---|---|
| 1.30 $x10^{-2}$ | -3.65 | 8.31 $x10^{-1}$ |

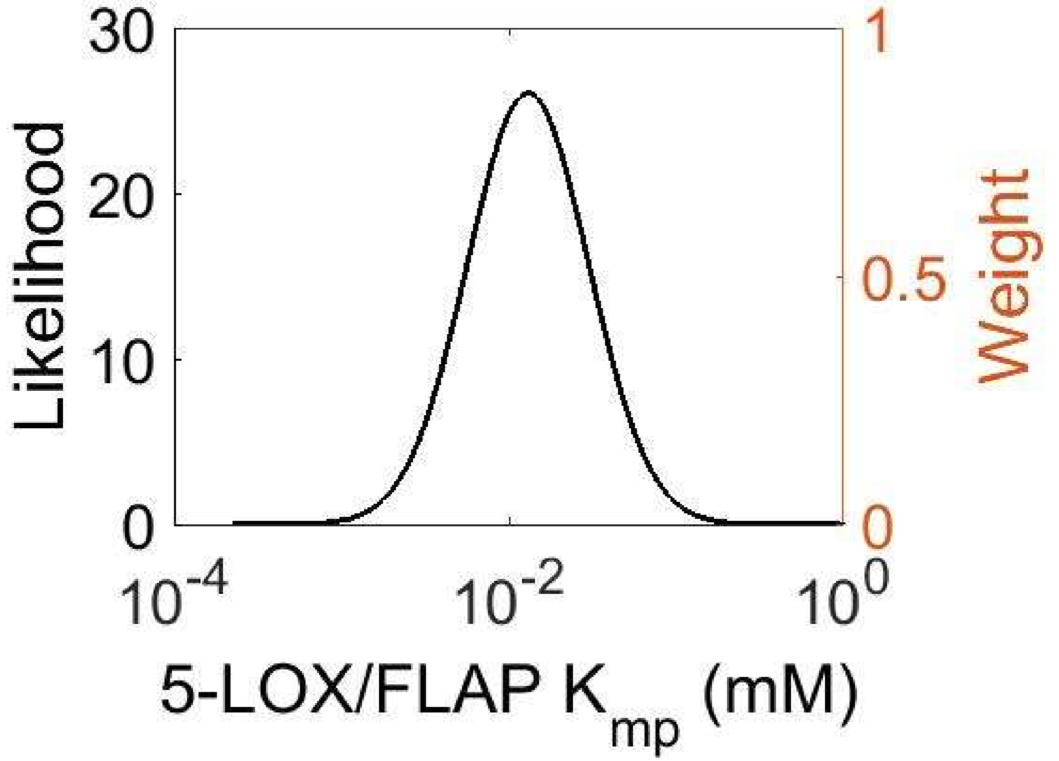


**Figure SF.7.8.1.2.1.** The estimated probability distribution for 5-LOX/FLAP $K_{mp}$. The value and weight of the literature values used to define the distribution are indicated by an orange dashed line. The x axis is plotted on a log-scale.

### S.7.8.1.3. Parameter: 5-LOX/FLAP $k_{cat}$

Parameter values for the $k_{cat}$ of Reaction 13 were obtained from the literature and summarised in Table ST.7.8.1.3.1. The log-normal distribution properties for the $k_{cat}$ of Reaction 13 are shown in Table ST.7.8.1.3.2 and plotted in Figure SF.7.8.1.3.1.

**Table ST.7.8.1.3.1.** Literature information used to design the 5-LOX/FLAP $k_{cat}$ parameter distribution. Each value was assigned a weight using the protocol described in Section 2.6.2. The type of error was assigned as 0 if it was additive and 1 if it was multiplicative.

| Value ($min^{-1}$) | Error ($min^{-1}$) | Experimental details | | | | | | Weight | Type of error | Reference |
|---|---|---|---|---|---|---|---|---|---|---|
| | | Species | Expression Vector | Enzyme | pH | Temperature (°C) | Other | | | |
| 1.50 x10$^{3}$ | 7.50 x10$^{1}$ | Potato | Potato | 5-LOX | 5.5 | 23 | | 16 | 0 | (Mulliez et al., 1987) |

**Table ST.7.8.1.3.2.** The log-normal distribution properties of the 5-LOX/FLAP $k_{cat}$ distribution. These values were calculated using the functions described in Section 2.6.2.

| Mode ($min^{-1}$) | Confidence Interval | Location parameter (μ) | Scale parameter (σ) |
|---|---|---|---|
| 1.49 x10$^{3}$ | 6.66 | 7.90 | 7.69 x10$^{-1}$ |

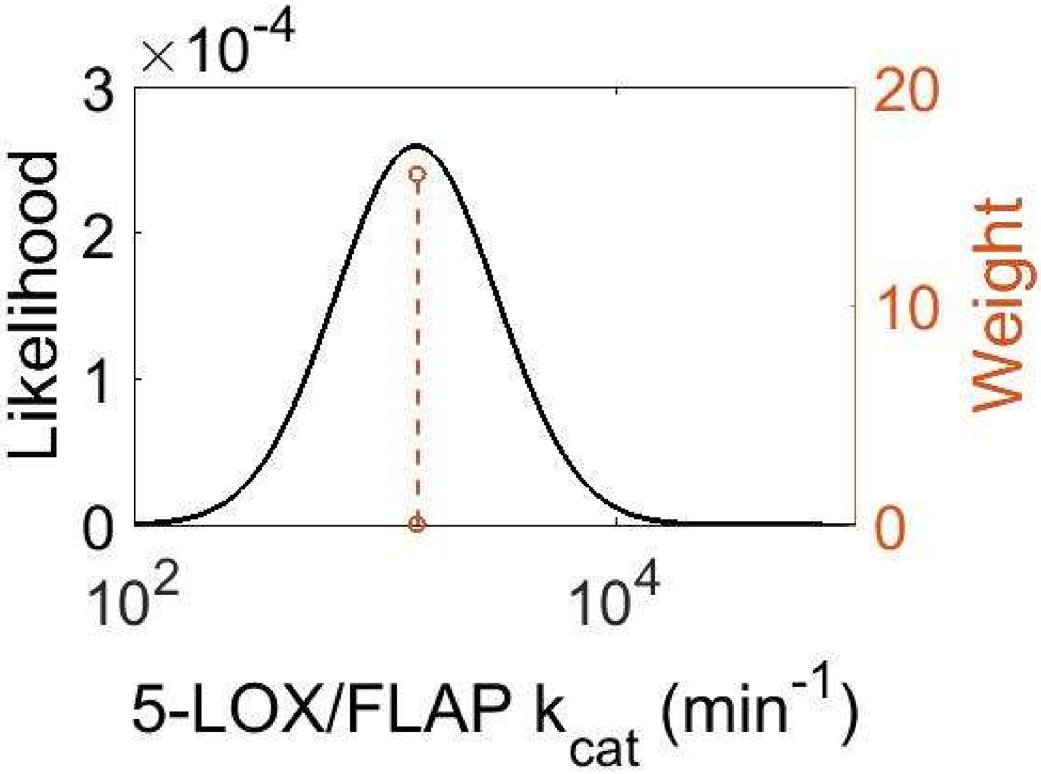


**Figure SF.7.8.1.3.1.** The estimated probability distribution for 5-LOX/FLAP $k_{cat}$. The value and weight of the literature values used to define the distribution are indicated by an orange dashed line. The x axis is plotted on a log-scale.

### S.7.8.1.4. Parameter: 5-LOX/FLAP $K_{eq}$

Parameter values for the $K_{eq}$ of Reaction 13 were obtained from the literature and summarised in Table ST.7.8.1.4.1. The log-normal distribution properties for the $K_{eq}$ of Reaction 13 are shown in Table ST.7.8.1.4.2 and plotted in Figure SF.7.8.1.4.1.

**Table ST.7.8.1.4.1.** Literature information used to design the 5-LOX/FLAP $K_{eq}$ parameter distribution. Each value was assigned a weight using the protocol described in Section 2.6.2. The type of error was assigned as 0 if it was additive and 1 if it was multiplicative.

| ΔG (kcal/mol) | $K_{eq}$ | Experimental details | | | | | | Weight | Type of error | Reference |
|---|---|---|---|---|---|---|---|---|---|---|
| | | Species | Expression Vector | Enzyme | pH | Temperature (°C) | Other | | | |
| -8.60 x10$^{1}$ | 1.31 x10$^{63}$ | Human | Unknown | 5-LOX/FLAP | 7 | Unknown | | 64 | 0 | (Caspi et al., 2018) |

**Table ST.7.8.1.4.2.** The log-normal distribution properties of the 5-LOX/FLAP $K_{eq}$ distribution. These values were calculated using the functions described in Section 2.6.2.

| Mode | Confidence Interval | Location parameter (μ) | Scale parameter (σ) |
|---|---|---|---|
| 1.31 x10$^{63}$ | 1.00 x10$^{1}$ | 1.46 x10$^{2}$ | 8.90 x10$^{-1}$ |

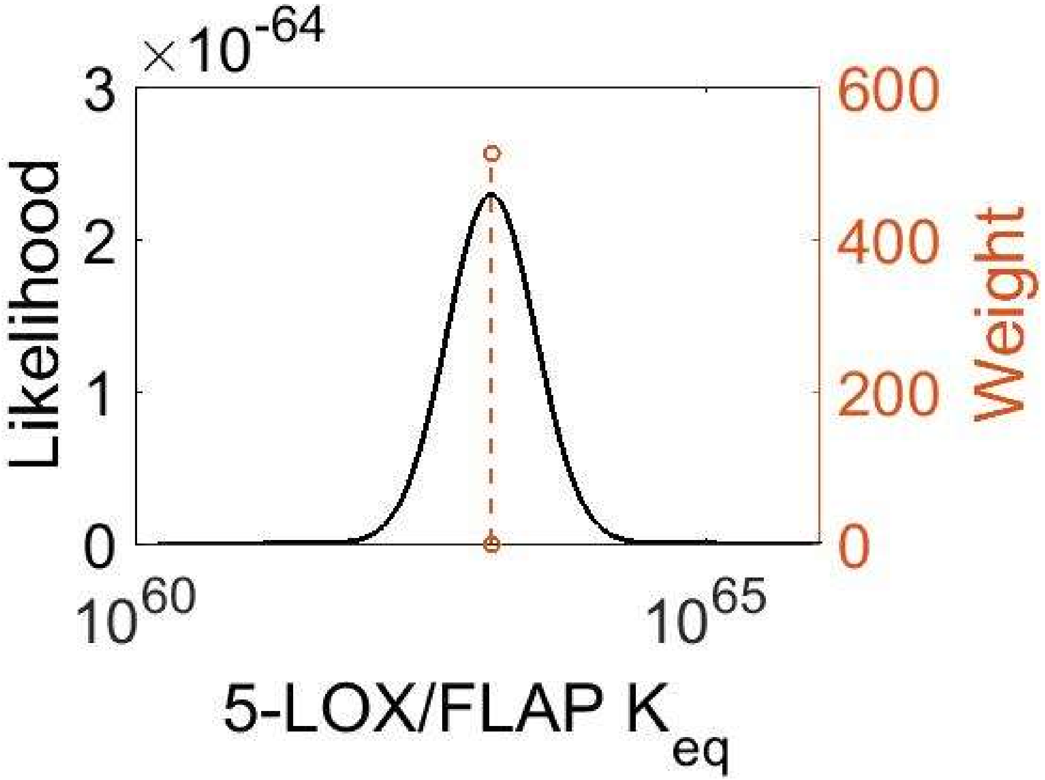


**Figure SF.7.8.1.4.1.** The estimated probability distribution for 5-LOX/FLAP $K_{eq}$. The value and weight of the literature values used to define the distribution are indicated by an orange dashed line. The x axis is plotted on a log-scale.

### S.7.8.1.5. Parameter: 5-LOX/FLAP concentration

Parameter values for the 5-LOX/FLAP concentration of Reaction 13 were obtained from the literature and summarised in Table ST.7.8.1.5.1. The log-normal distribution properties for the 5-LOX/FLAP concentration of Reaction 13 are shown in Table ST.7.8.1.5.2 and plotted in Figure SF.7.8.1.5.1. To convert the enzyme concentration from ppm to mM, **Equation S.6.2** was used.

**Table ST.7.8.1.5.1.** Literature information used to design the 5-LOX/FLAP concentration parameter distribution. Each value was assigned a weight using the protocol described in Section 2.6.2. The type of error was assigned as 0 if it was additive and 1 if it was multiplicative.

| Value (ppm) | Error (ppm) | Experimental details | | | | | | Weight | Type of error | Reference |
|---|---|---|---|---|---|---|---|---|---|---|
| | | Species | Expression Vector | Enzyme | pH | Temperature (°C) | Other | | | |
| $3.19 \times 10^{1}$ | NaN | Human | Oral cavity | 5-LOX | 7.5 | 37 | | **1024** | 0 | (Wilhelm et al., 2014) |
| $4.98 \times 10^{1}$ | NaN | Human | Oesophagus | 5-LOX | 7.5 | 37 | | **1024** | 0 | (Wilhelm et al., 2014) |
| $9.73 \times 10^{1}$ | NaN | Human | Lung | 5-LOX | 7.5 | 37 | | **1024** | 0 | (Kim et al., 2014) |

**Table ST.7.8.1.5.2.** The log-normal distribution properties of the 5-LOX/FLAP concentration distribution. These values were calculated using the functions described in Section 2.6.2.

| Mode (ppm) | Mode (mM) | Confidence Interval | Location parameter (μ) | Scale parameter (σ) |
|---|---|---|---|---|
| $4.96 \times 10^{1}$ | $2.74 \times 10^{-4}$ | 1.60 | 4.09 | $4.28 \times 10^{-1}$ |

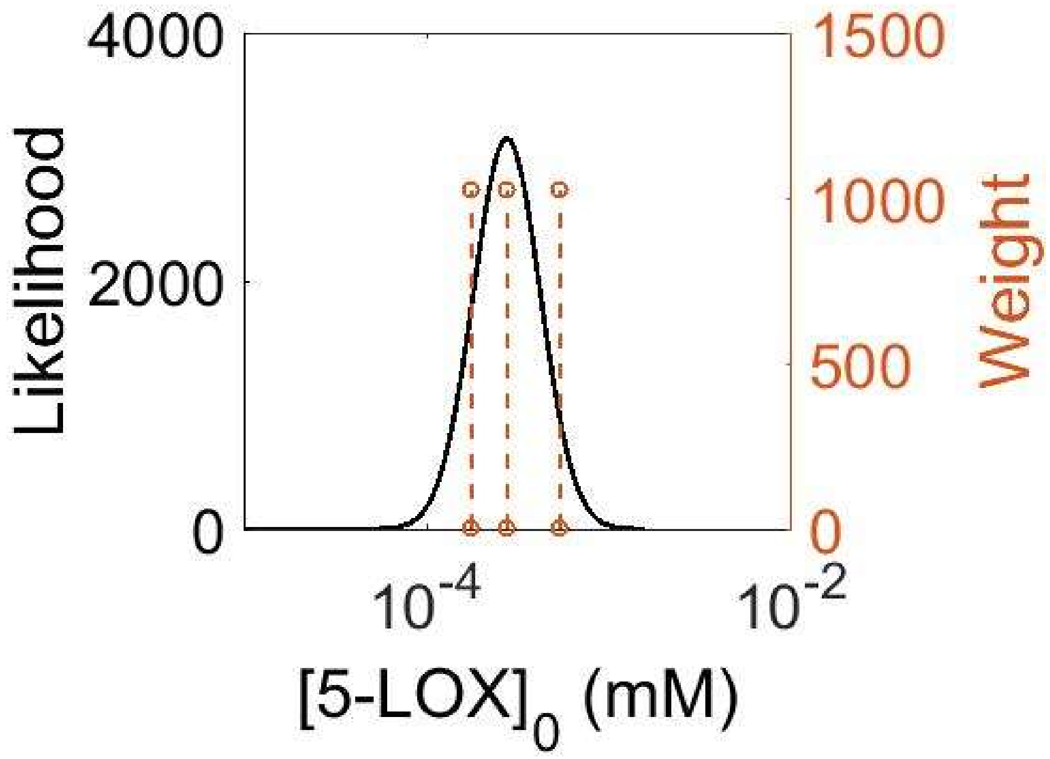


**Figure SF.7.8.1.5.1.** The estimated probability distribution for the 5-LOX concentration. The value and weight of the literature values used to define the distribution are indicated by an orange dashed line. The x axis is plotted on a log-scale.

### S.7.9 Reaction 14: 5-HETE ⇌ 5-oxo-ETE

5-HETE is then oxidised by 5-hydroxyeicosanoid dehydrogenase (5-HEDH) to 5-oxo-ETE (Powell et al., 1992). This reaction is selective for 5(S)-HETE and the cofactor is NADP$^+$ (Powell et al., 1992). 5-HEDH is a microsomal enzyme which catalyses the conversion of the C5 alcohol to a ketone by transferring the hydrogen cation to NADP$^+$ via a ping-pong mechanism (K.-R. Erlemann et al., 2007; Powell et al., 1992).

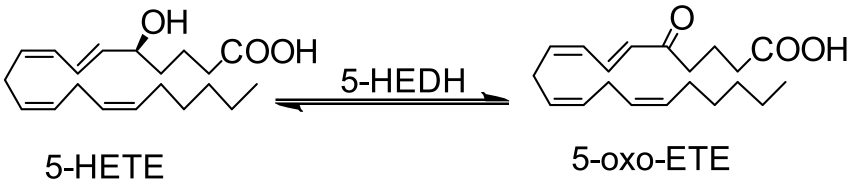


**Figure SF.7.9.** The metabolism of 5-hydroxyeicosatetraenoic acid (5-HETE) into 5-oxo-eicosatetraenoic acid (5-oxo-ETE) by 5-hydroxyeicosanoid dehydrogenase (5-HEDH) (Reaction 14).

**SEq.7.9.** Reaction rate law for Reaction 14.

$$\boldsymbol{v_{14}} = \frac{K_{cat}\cdot[5-HEDH]\left([5-HETE]-{[5-oxo-ETE]}/{K_{eq}}\right)}{K_{m_S}\left(1+\frac{[5-oxo-ETE]}{K_{m_P}}\right)+[5-HETE]}$$

### S.7.9.1. Reaction parameters

#### S.7.9.1.1. Parameter: 5-HEDH $K_{ms}$

Parameter values for the $K_{ms}$ of Reaction 14 were obtained from the literature and summarised in Table ST.7.9.1.1.1. The log-normal distribution properties for the $K_{ms}$ of Reaction 14 are shown in Table ST.7.9.1.1.2 and plotted in Figure SF.7.9.1.1.1.

**Table ST.7.9.1.1.1.** Literature information used to design the 5-HEDH $K_{ms}$ parameter distribution. Each value was assigned a weight using the protocol described in Section 2.6.2. The type of error was assigned as 0 if it was additive and 1 if it was multiplicative.

| Value (mM) | Error (mM) | Experimental details | | | | | | Weight | Type of error | Reference |
|---|---|---|---|---|---|---|---|---|---|---|
| | | Species | Expression Vector | Enzyme | pH | Temperature (°C) | Other | | | |
| 6.00 x$10^{-4}$ | | Human | Unknown | 5-HEDH | Unknown | Unknown | | 64 | | (Steinhilber, 2016) |
| 6.70 x$10^{-4}$ | | Human | Cell line | 5-HEDH | 7.4 | 37 | | 2048 | | (K. R. Erlemann et al., 2007) |
| 5.16 x$10^{-4}$ | 1.90 x$10^{-4}$ | Human | Cell line | 5-HEDH | 7.4 | 37 | | 2048 | | (Patel P., 2009) |

**Table ST.7.9.1.1.2.** The log-normal distribution properties of the 5-HEDH $K_{ms}$ distribution. These values were calculated using the functions described in Section 2.6.2.

| Mode (mM) | Confidence Interval | Location parameter (μ) | Scale parameter (σ) |
|---|---|---|---|
| 6.34 x$10^{-4}$ | 1.63 x$10^{1}$ | -6.31 | 1.03 |

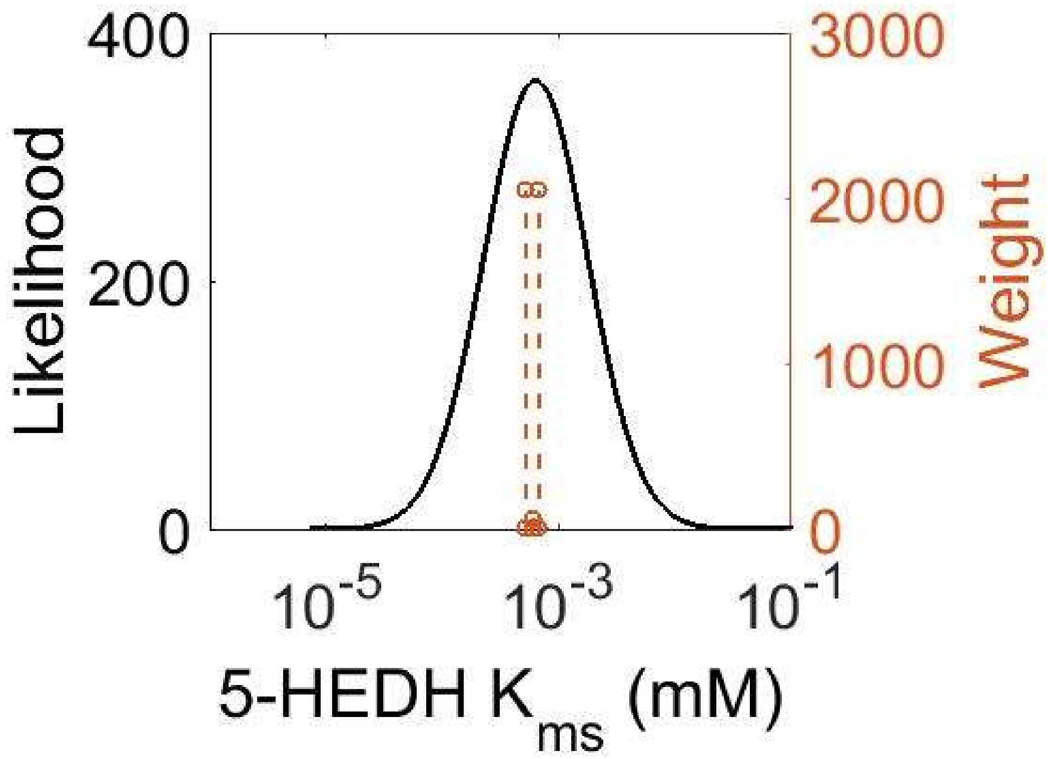


**Figure SF.7.9.1.1.1.** The estimated probability distribution for 5-HEDH $K_{ms}$. The value and weight of the literature values used to define the distribution are indicated by an orange dashed line. The x axis is plotted on a log-scale.

### S.7.9.1.2. Parameter: 5-HEDH $K_{mp}$ (Dependent parameter)

The log-normal distribution for the parameter for the $K_{mp}$ of Reaction 14 was calculated using multivariate distributions. The log-normal distribution properties for the $K_{mp}$ of Reaction 14 are shown in Table ST.7.9.1.2.1 and plotted in Figure SF.7.9.1.2.1.

**Table ST.7.9.1.2.1.** The log-normal distribution properties of the 5-HEDH $K_{mp}$ distribution. These values were calculated using the functions described in Section 2.6.2.

| Mode (mM) | Location parameter (μ) | Scale parameter (σ) |
|---|---|---|
| $1.14 \times 10^4$ | $1.09 \times 10^1$ | 1.25 |

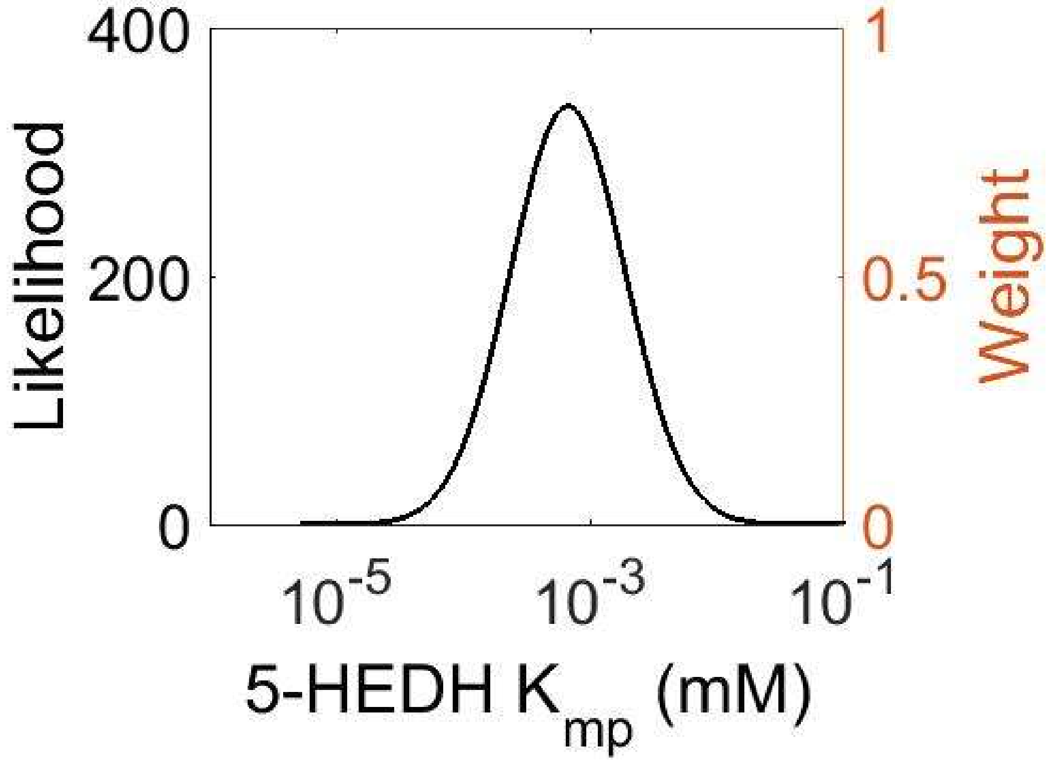


**Figure SF.7.9.1.2.1.** The estimated probability distribution for 5-HEDH $K_{mp}$. The value and weight of the literature values used to define the distribution are indicated by an orange dashed line. The x axis is plotted on a log-scale.

### S.7.9.1.3. Parameter: 5-HEDH $k_{cat}$

Parameter values for the $k_{cat}$ of Reaction 14 were obtained from the literature and summarised in Table ST.7.9.1.3.1. The log-normal distribution properties for the $k_{cat}$ of Reaction 14 are shown in Table ST.7.9.1.3.2 and plotted in Figure SF.7.9.1.3.1.

**Table ST.7.9.1.3.1.** Literature information used to design the 5-HEDH $k_{cat}$ parameter distribution. Each value was assigned a weight using the protocol described in Section 2.6.2. The type of error was assigned as 0 if it was additive and 1 if it was multiplicative.

| Value ($min^{-1}$) | Error ($min^{-1}$) | Experimental details | | | | | | Weight | Type of error | Reference |
|---|---|---|---|---|---|---|---|---|---|---|
| | | Species | Expression Vector | Enzyme | pH | Temperature (°C) | Other | | | |
| $1.16 \times 10^{4}$ | $8.40 \times 10^{2}$ | Yokenella | E. coli. | 5-HEDH | 6.5 | 65 | | 128 | | (Wei, 2012) |

**Table ST.7.9.1.3.2.** The log-normal distribution properties of the 5-HEDH $k_{cat}$ distribution. These values were calculated using the functions described in Section 2.6.2.

| Mode ($min^{-1}$) | Confidence Interval | Location parameter (μ) | Scale parameter (σ) |
|---|---|---|---|
| $1.15 \times 10^{4}$ | $4.02 \times 10^{1}$ | $1.09 \times 10^{1}$ | 1.25 |

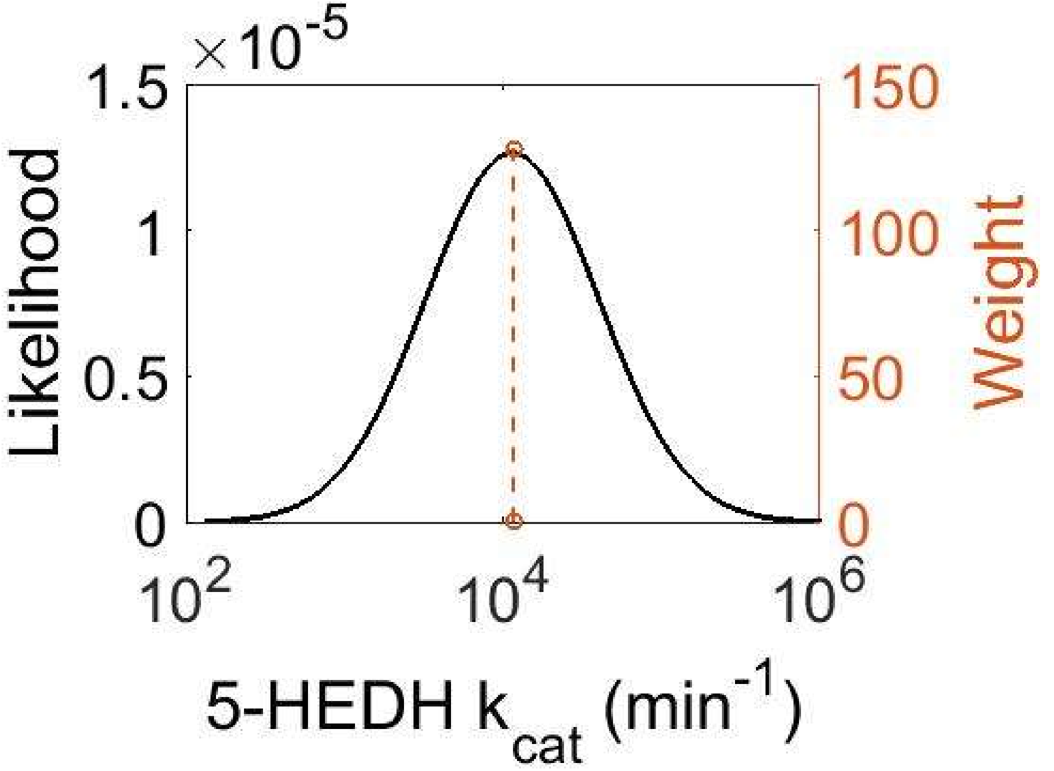


**Figure SF.7.9.1.3.1.** The estimated probability distribution for 5-HEDH $k_{cat}$. The value and weight of the literature values used to define the distribution are indicated by an orange dashed line. The x axis is plotted on a log-scale.

### S.7.9.1.4. Parameter: 5-HEDH $K_{eq}$

Parameter values for the $K_{eq}$ of Reaction 14 were obtained from the literature and summarised in Table ST.7.9.1.4.1. The log-normal distribution properties for the $K_{eq}$ of Reaction 14 are shown in Table ST.7.9.1.4.2 and plotted in Figure SF.7.9.1.4.1.

**Table ST.7.9.1.4.1.** Literature information used to design the 5-HEDH $K_{eq}$ parameter distribution. Each value was assigned a weight using the protocol described in Section 2.6.2. The type of error was assigned as 0 if it was additive and 1 if it was multiplicative.

| ΔG (kcal/mol) | $K_{eq}$ | Experimental details | | | | | | Weight | Type of error | Reference |
|---|---|---|---|---|---|---|---|---|---|---|
| | | Species | Expression Vector | Enzyme | pH | Temperature (°C) | Other | | | |
| 1.29 | 1.12 x10$^{-1}$ | Human | Unknown | 5-HEDH | 7 | Unknown | | 64 | 0 | (Caspi et al., 2018) |

**Table ST.7.9.1.4.2.** The log-normal distribution properties of the 5-HEDH $K_{eq}$ distribution. These values were calculated using the functions described in Section 2.6.2.

| Mode | Confidence Interval | Location parameter (μ) | Scale parameter (σ) |
|---|---|---|---|
| 1.12 x10$^{-1}$ | 1.00 x10$^{1}$ | -1.39 | 8.91 x10$^{-1}$ |

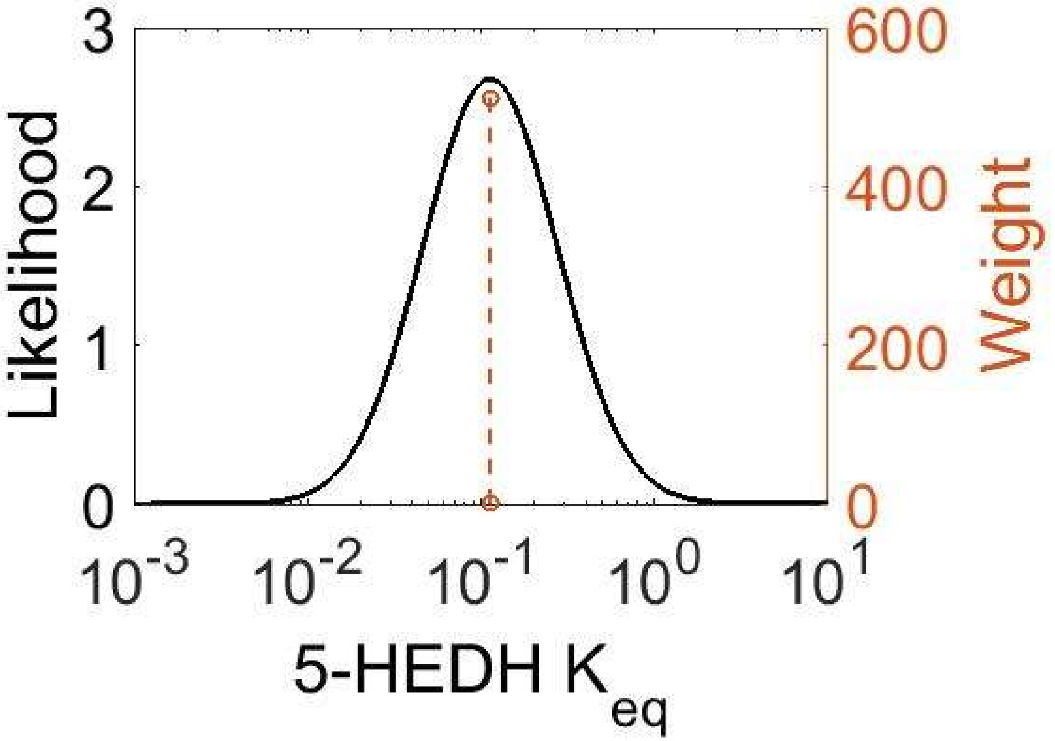


**Figure SF.7.9.1.4.1.** The estimated probability distribution for 5-HEDH $K_{eq}$. The value and weight of the literature values used to define the distribution are indicated by an orange dashed line. The x axis is plotted on a log-scale.

### S.7.9.1.5. Parameter: 5-HEDH concentration

Parameter values for the 5-HEDH concentration of Reaction 14 were obtained from the literature and summarised in Table ST.7.9.1.5.1. The log-normal distribution properties for the 5-HEDH concentration of Reaction 14 are shown in Table ST.7.9.1.5.2 and plotted in Figure SF.7.9.1.5.1. To convert the enzyme concentration from ppm to mM, **Equation S.6.2** was used.

**Table ST.7.9.1.5.1.** Literature information used to design the 5-HEDH concentration parameter distribution. Each value was assigned a weight using the protocol described in Section 2.6.2. The type of error was assigned as 0 if it was additive and 1 if it was multiplicative.

| Value (ppm) | Error (ppm) | Experimental details | | | | | | Weight | Type of error | Reference |
|---|---|---|---|---|---|---|---|---|---|---|
| | | Species | Expression Vector | Enzyme | pH | Temperature (°C) | Other | | | |
| $4.90 \times 10^2$ | | Human | Skin | 5-HEDH | 7.5 | 37 | | **2048** | | (Wilhelm et al., 2014) |
| $6.25 \times 10^2$ | | Human | Oral cavity | 5-HEDH | 7.5 | 37 | | 1024 | | (Wilhelm et al., 2014) |
| $7.28 \times 10^2$ | | Human | Oesophagus | 5-HEDH | 7.5 | 37 | | 1024 | | (Kim et al., 2014) |
| $1.15 \times 10^1$ | | Human | Skin | 5-HEDH | 7.5 | 37 | | **2048** | | (Kim et al., 2014) |

**Table ST.7.9.1.5.2.** The log-normal distribution properties of the 5-HEDH concentration distribution. These values were calculated using the functions described in Section 2.6.2.

| Mode (ppm) | Mode (mM) | Confidence Interval | Location parameter (μ) | Scale parameter (σ) |
|---|---|---|---|---|
| $4.88 \times 10^2$ | $2.70 \times 10^{-3}$ | 6.37 | 7.49 | 1.14 |

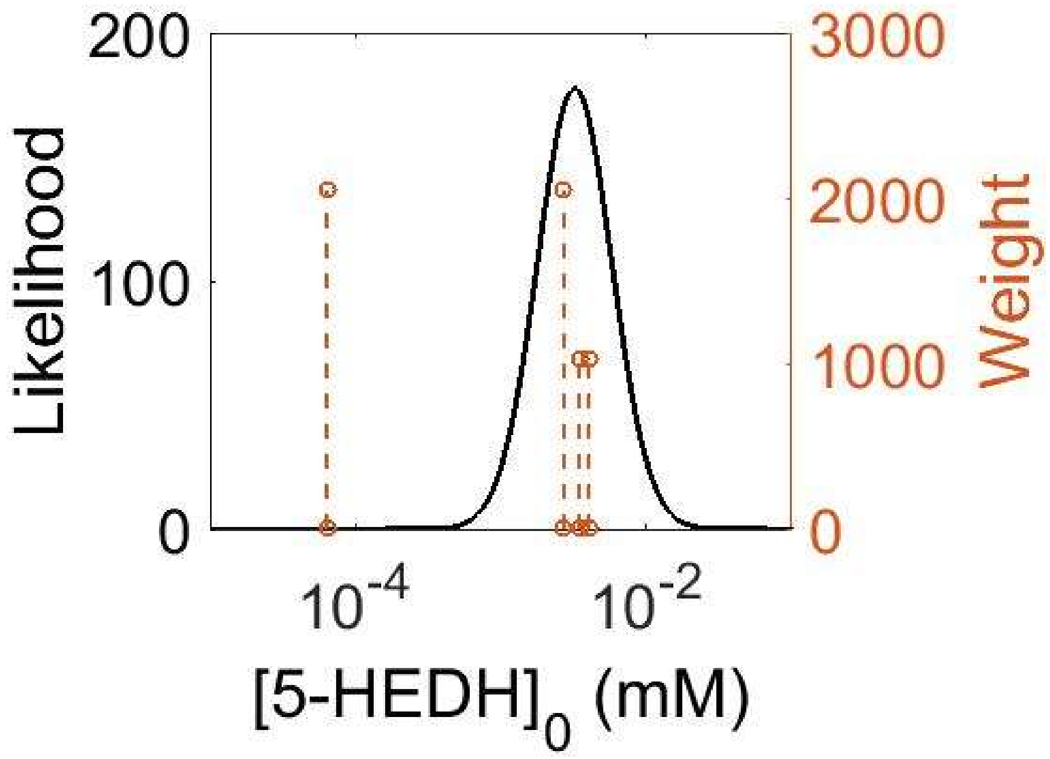


**Figure SF.7.9.1.5.1.** The estimated probability distribution for the 5-HEDH concentration. The value and weight of the literature values used to define the distribution are indicated by an orange dashed line. The x axis is plotted on a log-scale.

### S.7.10 Reaction 15: $LTA_4 \rightleftharpoons LTB_4$

$LTA_4$ is subsequently enzymatically hydrolysed to $LTB_4$ by $LTA_4$ hydrolase ($LTA_4H$). This reaction is stereospecific (Haeggstrom et al., 2007) and relies on the $Zn^{2+}$ at the active site of $LTA_4H$. . The enzyme performs this reaction by opening the epoxide and creating a carbocation intermediate. The charge of the carbocation delocalises over the triene system and is subsequently subject to nucleophilic attack by water at C12, resulting in the stereospecific addition of a hydroxyl group and the generation of $LTB_4$.

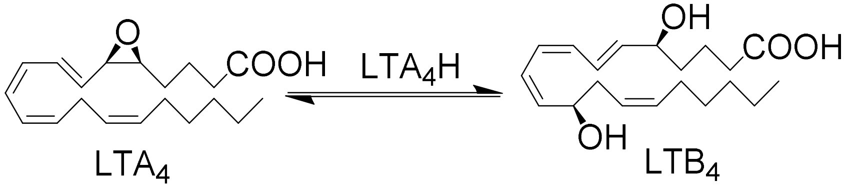


**Figure SF.7.10.** The metabolism of leukotriene $A_4$ ($LTA_4$) into leukotriene $B_4$ ($LTB_4$) by $LTA_4$ hydrolase ($LTA_4H$) (Reaction 15).

**SEq.7.10.** Reaction rate law for Reaction 15.

$$\boldsymbol{v_{15}} = \frac{K_{cat}\cdot[LTA_4H]\left([LTA_4]-{[LTB_4]}/{K_{eq}}\right)}{K_{m_s}\left(1+\frac{[LTB_4]}{K_{m_p}}\right)+[LTA_4]}$$

### S.7.10.1. Reaction parameters

#### S.7.10.1.1. Parameter: $LTA_4H$ $K_{ms}$

Parameter values for the $K_{ms}$ of Reaction 15 were obtained from the literature and summarised in Table ST.7.10.1.1.1. The log-normal distribution properties for the $K_{ms}$ of Reaction 15 are shown in Table ST.7.10.1.1.2 and plotted in Figure SF.7.10.1.1.1.

**Table ST.7.10.1.1.1.** Literature information used to design the $LTA_4H$ $K_{ms}$ parameter distribution. Each value was assigned a weight using the protocol described in Section 2.6.2. The type of error was assigned as 0 if it was additive and 1 if it was multiplicative.

| Value (mM) | Error (mM) | Experimental details | | | | | | Weight | Type of error | Reference |
|---|---|---|---|---|---|---|---|---|---|---|
| | | Species | Expression Vector | Enzyme | pH | Temperature (°C) | Other | | | |
| $2.30 \times 10^{-3}$ | NaN | Human | Leukocytes | $LTA_4H$ | 8 | 37 | | 1024 | 0 | (Radmark et al., 1984) |
| $2.70 \times 10^{-3}$ | NaN | Frog | Oocytes | $LTA_4H$ | 8 | 20 | | 16 | 0 | (Stromberg-Kull and Haeggstrom, 1998) |
| $5.80 \times 10^{-3}$ | NaN | Human | E. coli. | $LTA_4H$ | 8 | 20 | | 128 | 0 | (Mueller et al., 1996) |
| $2.20 \times 10^{-2}$ | NaN | Human | Leukocytes | $LTA_4H$ | 8 | 2 | | 256 | 0 | (Radmark et al., 1984) |

**Table ST.7.10.1.1.2.** The log-normal distribution properties of the $LTA_4H$ $K_{ms}$ distribution. These values were calculated using the functions described in Section 2.6.2.

| **Mode (mM)** | **Confidence Interval** | **Location parameter (μ)** | **Scale parameter (σ)** |
|---|---|---|---|
| 2.40 x10$^{-3}$ | 5.77 | -5.51 | 7.20 x10$^{-1}$ |

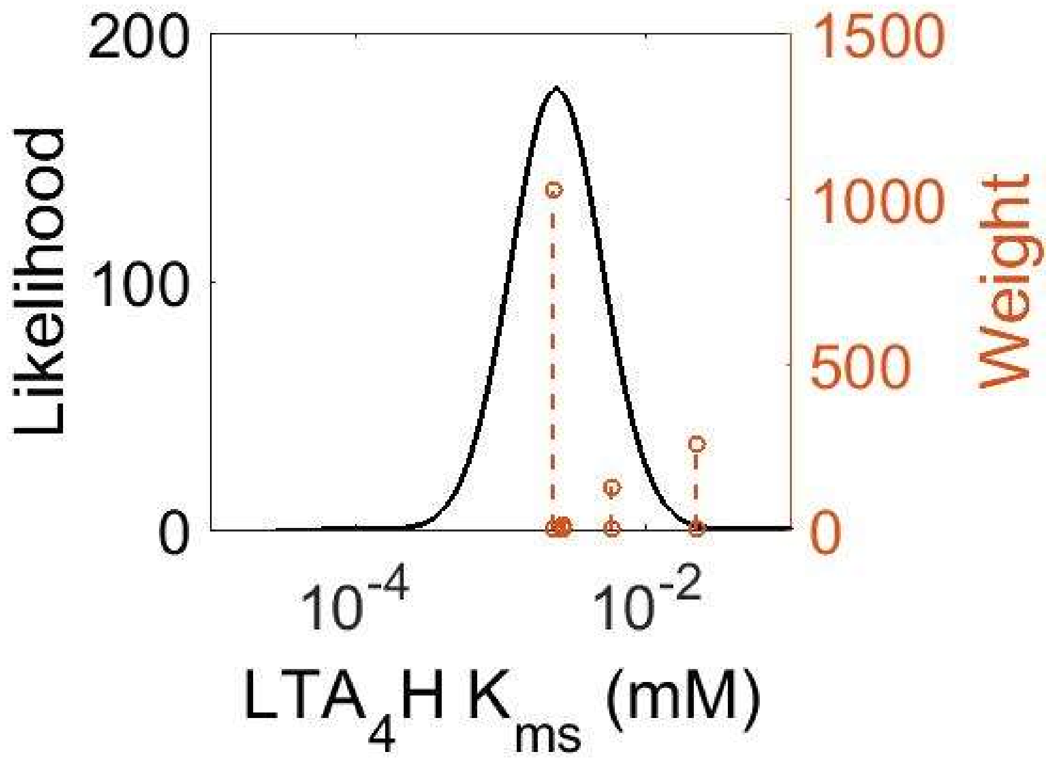


**Figure SF.7.10.1.1.1.** The estimated probability distribution for $LTA_4H$ $K_{ms}$. The value and weight of the literature values used to define the distribution are indicated by an orange dashed line. The x axis is plotted on a log-scale.

### S.7.10.1.2. Parameter: $LTA_4H$ $K_{mp}$ (Dependent parameters)

The log-normal distribution for the parameter for the $K_{mp}$ of Reaction 15 was calculated using multivariate distributions. The log-normal distribution properties for the $K_{mp}$ of Reaction 15 are shown in Table ST.7.10.1.2.1 and plotted in Figure SF.7.10.1.2.1.

**Table ST.7.10.1.2.1.** The log-normal distribution properties of the $LTA_4H$ $K_{mp}$ distribution. These values were calculated using the functions described in Section 2.6.2.

| Mode (mM) | Location parameter (μ) | Scale parameter (σ) |
|---|---|---|
| 2.40 x$10^{-3}$ | -5.51 | 7.17 x$10^{-1}$ |

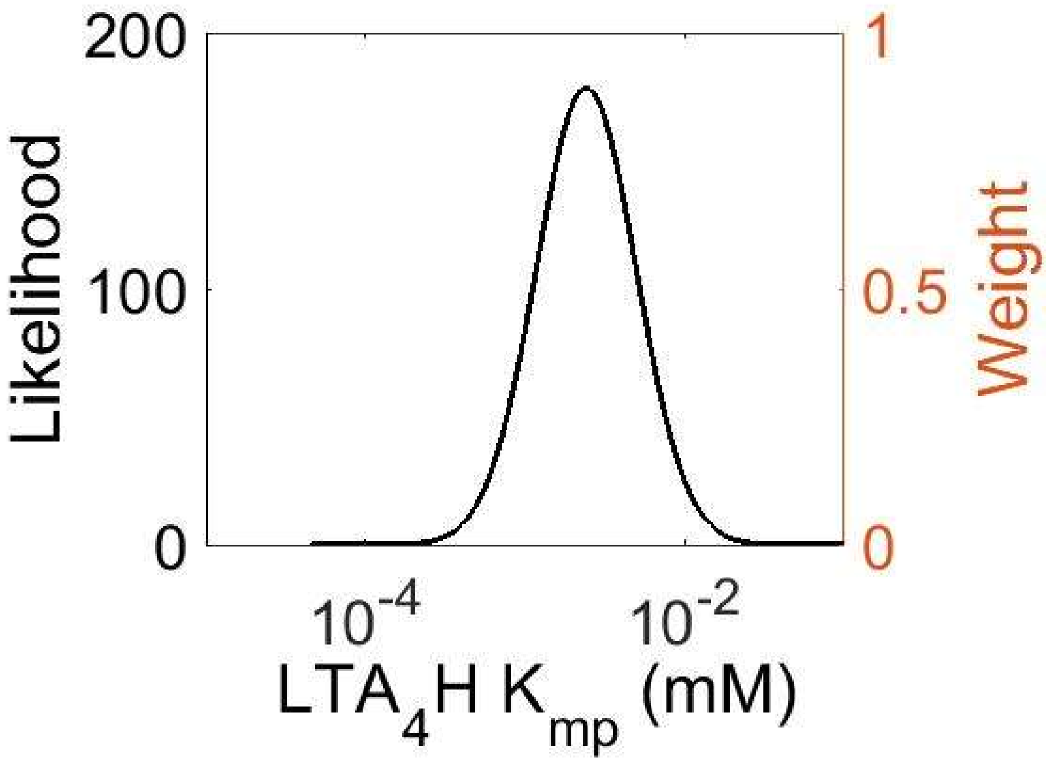


**Figure SF.7.10.1.2.1.** The estimated probability distribution for $LTA_4H$ $K_{mp}$. The value and weight of the literature values used to define the distribution are indicated by an orange dashed line. The x axis is plotted on a log-scale.

### S.7.10.1.3. Parameter: $LTA_4H$ $k_{cat}$

Parameter values for the $k_{cat}$ of Reaction 15 were obtained from the literature and summarised in Table ST.7.10.1.3.1. The log-normal distribution properties for the $k_{cat}$ of Reaction 15 are shown in Table ST.7.10.1.3.2 and plotted in Figure SF.7.10.1.3.1.

**Table ST.7.10.1.3.1.** Literature information used to design the $LTA_4H$ $k_{cat}$ parameter distribution. Each value was assigned a weight using the protocol described in Section 2.6.2. The type of error was assigned as 0 if it was additive and 1 if it was multiplicative.

| Value ($min^{-1}$) | Error ($min^{-1}$) | Experimental details | | | | | | Weight | Type of error | Reference |
|---|---|---|---|---|---|---|---|---|---|---|
| | | Species | Expression Vector | Enzyme | pH | Temperature (°C) | Other | | | |
| $1.26 \times 10^{1}$ | NaN | Human | E. coli. | LTA4H | 7.4 | 37 | | 1024 | 0 | (Rudberg et al., 2002) |
| $2.82 \times 10^{1}$ | NaN | Human | E. coli. | LTA4H | 7.4 | 37 | | 1024 | 0 | (Rudberg et al., 2002) |
| $5.10 \times 10^{1}$ | NaN | Human | E. coli. | LTA4H | 8 | 20 | | 128 | 0 | (Mueller et al., 1996) |
| $9.00 \times 10^{1}$ | NaN | Frog | Oocytes | LTA4H | 8 | 20 | | 16 | 0 | (Stromberg-Kull and Haeggstrom, 1998) |
| $1.25 \times 10^{2}$ | NaN | Human | Leukocytes | LTA4H | 8 | 37 | | 1024 | 0 | (Radmark et al., 1984) |

**Table ST.7.10.1.3.2.** The log-normal distribution properties of the $LTA_4H$ $k_{cat}$ distribution. These values were calculated using the functions described in Section 2.6.2.

| Mode ($min^{-1}$) | Confidence Interval | Location parameter (μ) | Scale parameter (σ) |
|---|---|---|---|
| $2.85 \times 10^{1}$ | 6.56 | 3.94 | $7.60 \times 10^{-1}$ |

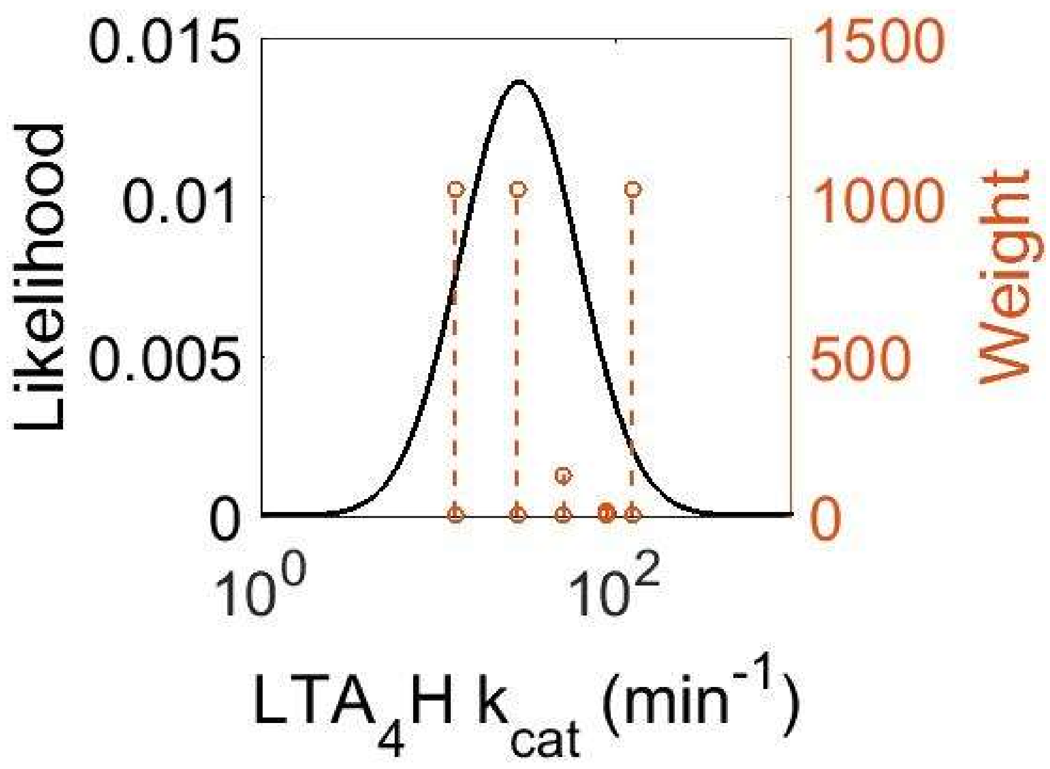


**Figure SF.7.10.1.3.1.** The estimated probability distribution for $LTA_4H$ $k_{cat}$. The value and weight of the literature values used to define the distribution are indicated by an orange dashed line. The x axis is plotted on a log-scale.

### S.7.10.1.4. Parameter: $LTA_4H$ $K_{eq}$

Parameter values for the $K_{eq}$ of Reaction 15 were obtained from the literature and summarised in Table ST.7.10.1.4.1. The log-normal distribution properties for the $K_{eq}$ of Reaction 15 are shown in Table ST.7.10.1.4.2 and plotted in Figure SF.7.10.1.4.1.

**Table ST.7.10.1.4.1.** Literature information used to design the $LTA_4H$ $K_{eq}$ parameter distribution. Each value was assigned a weight using the protocol described in Section 2.6.2. The type of error was assigned as 0 if it was additive and 1 if it was multiplicative.

| ΔG (kcal/mol) | $K_{eq}$ | Experimental details | | | | | | Weight | Type of error | Reference |
|---|---|---|---|---|---|---|---|---|---|---|
| | | Species | Expression Vector | Enzyme | pH | Temperature (°C) | Other | | | |
| -3.63 | 4.64 x10$^{2}$ | Human | Unknown | LTA4H | 7 | Unknown | | 64 | 0 | (Caspi et al., 2018) |

**Table ST.7.10.1.4.2.** The log-normal distribution properties of the $LTA_4H$ $K_{eq}$ distribution. These values were calculated using the functions described in Section 2.6.2.

| Mode | Confidence Interval | Location parameter (μ) | Scale parameter (σ) |
|---|---|---|---|
| 4.64 x10$^{2}$ | 1.00 x10$^{1}$ | 6.93 | 8.90 x10$^{-1}$ |

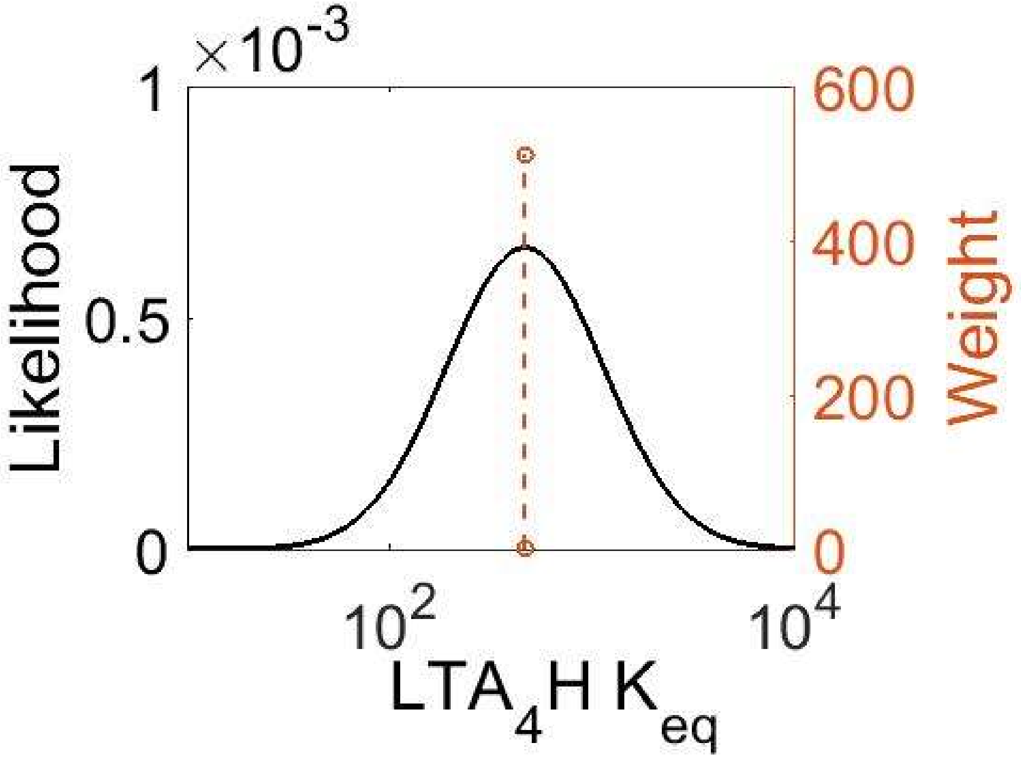


**Figure SF.7.10.1.4.1.** The estimated probability distribution for $LTA_4H$ $K_{eq}$. The value and weight of the literature values used to define the distribution are indicated by an orange dashed line. The x axis is plotted on a log-scale.

### S.7.10.1.5. Parameter: $LTA_4H$ concentration

Parameter values for the $LTA_4H$ concentration of Reaction 15 were obtained from the literature and summarised in Table ST.7.10.1.5.1. The log-normal distribution properties for the $LTA_4H$ concentration of Reaction 15 are shown in Table ST.7.10.1.5.2 and plotted in Figure SF.7.10.1.5.1. To convert the enzyme concentration from ppm to mM, **Equation S.6.2** was used.

**Table ST.7.10.1.5.1.** Literature information used to design the $LTA_4H$ concentration parameter distribution. Each value was assigned a weight using the protocol described in Section 2.6.2. The type of error was assigned as 0 if it was additive and 1 if it was multiplicative.

| Value (mM) | Error (mM) | Experimental details | | | | | | Weight | Type of error | Reference |
|---|---|---|---|---|---|---|---|---|---|---|
| | | Species | Expression Vector | Enzyme | pH | Temperature (°C) | Other | | | |
| 4.10 x10$^{2}$ | NaN | Human | Oral cavity | LTA4H | 7.5 | 37 | | **1024** | 0 | (Wilhelm et al., 2014) |
| 4.89 x10$^{2}$ | NaN | Human | Skin | LTA4H | 7.5 | 37 | | **2048** | 0 | (Wilhelm et al., 2014) |
| 7.14 x10$^{2}$ | NaN | Human | Lung | LTA4H | 7.5 | 37 | | **1024** | 0 | (Kim et al., 2014) |

**Table ST.7.10.1.5.2.** The log-normal distribution properties of the $LTA_4H$ concentration distribution. These values were calculated using the functions described in Section 2.6.2.

| Mode (ppm) | Mode (mM) | Confidence Interval | Location parameter (μ) | Scale parameter (σ) |
|---|---|---|---|---|
| 4.88 x10$^{2}$ | 2.70 x10$^{-3}$ | 1.25 | 6.24 | 2.19 x10$^{-1}$ |

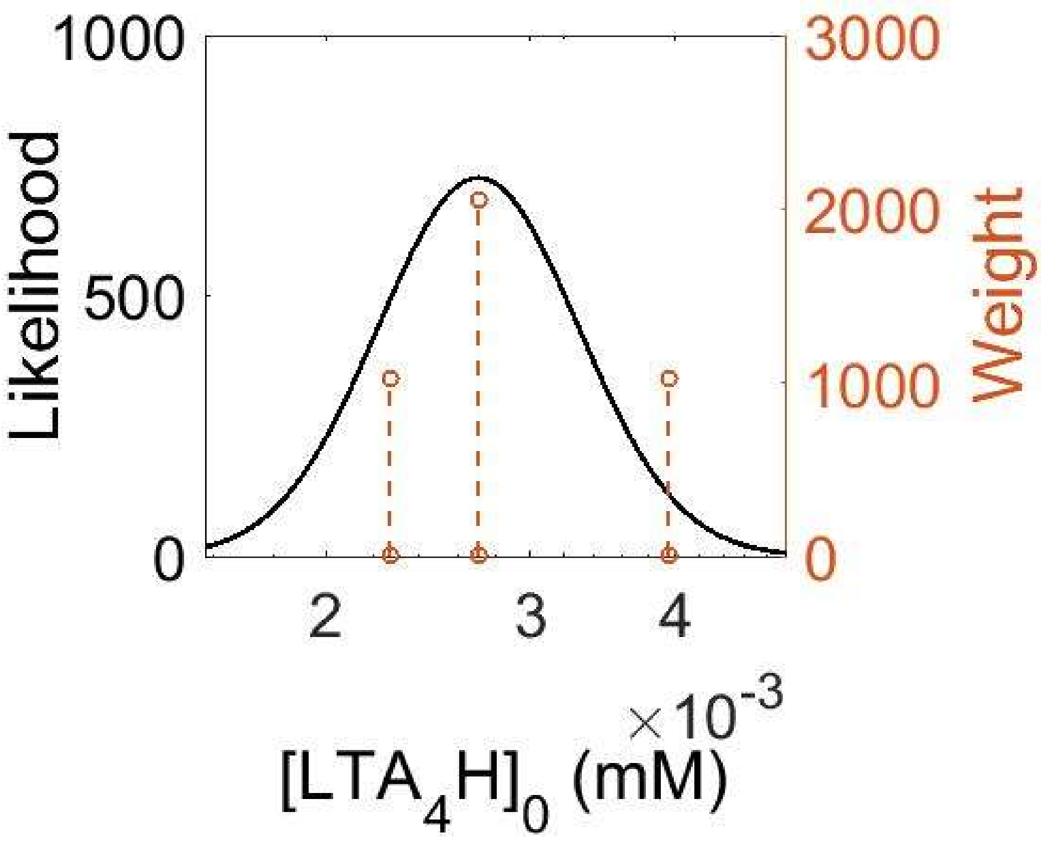


**Figure SF.7.10.1.5.1.** The estimated probability distribution for the $LTA_4H$ concentration. The value and weight of the literature values used to define the distribution are indicated by an orange dashed line. The x axis is plotted on a log-scale.

### S.7.11 Reaction 16: $LTA_4 \rightleftharpoons LTC_4$

$LTA_4$ can be metabolised by leukotriene $C_4$ synthase ($LTC_4S$), into $LTC_4$. This reaction involves the conjugation of the $LTA_4$ epoxide and glutathione.

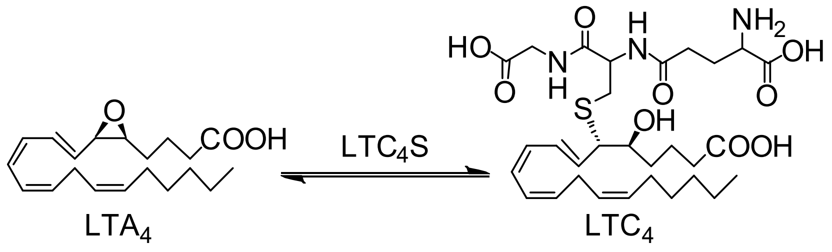


**Figure SF.7.11.** The metabolism of leukotriene $A_4$ ($LTA_4$) into leukotriene $C_4$ ($LTC_4$) by leukotriene $C_4$ synthase ($LTC_4S$) (Reaction 16).

**SEq.7.11.** Reaction rate law for Reaction 16.

$$\boldsymbol{v_{16}} = \frac{K_{cat} \cdot [LTC_4S] \left( [LTA_4] - {[LTC_4]}/{K_{eq}} \right)}{K_{m_s}\left(1 + \frac{[LTC_4]}{K_{m_p}}\right) + [LTA_4]}$$

### S.7.11.1. Reaction parameters

#### S.7.11.1.1. Parameter: LTC4S $K_{ms}$

Parameter values for the $K_{ms}$ of Reaction 16 were obtained from the literature and summarised in Table ST.7.11.1.1.1. The log-normal distribution properties for the $K_{ms}$ of Reaction 16 are shown in Table ST.7.11.1.1.2 and plotted in Figure SF.7.11.1.1.1.

**Table ST.7.11.1.1.1.** Literature information used to design the LTC4S $K_{ms}$ parameter distribution. Each value was assigned a weight using the protocol described in Section 2.6.2. The type of error was assigned as 0 if it was additive and 1 if it was multiplicative.

| Value (mM) | Error (mM) | Experimental details | | | | | | Weight | Type of error | Reference |
|---|---|---|---|---|---|---|---|---|---|---|
| | | Species | Expression Vector | Enzyme | pH | Temperature (°C) | Other | | | |
| 3.00 x10$^{-1}$ | 6.00 x10$^{-2}$ | Human | E. coli. | hLTC4S | 7.8 | 20 | | 256 | 0 | (Rinaldo-Matthis et al., 2010) |
| 3.00 x10$^{-2}$ | 1.00 x10$^{-2}$ | Human | E. coli. | LTC4S | 7.8 | 37 | | 1024 | 0 | (Niegowski et al., 2013) |

**Table ST.7.11.1.1.2.** The log-normal distribution properties of the LTC4S $K_{ms}$ distribution. These values were calculated using the functions described in Section 2.6.2.

| Mode (mM) | Confidence Interval | Location parameter (µ) | Scale parameter (σ) |
|---|---|---|---|
| 3.15 x10$^{-2}$ | 7.18 | -2.83 | 7.90 x10$^{-1}$ |

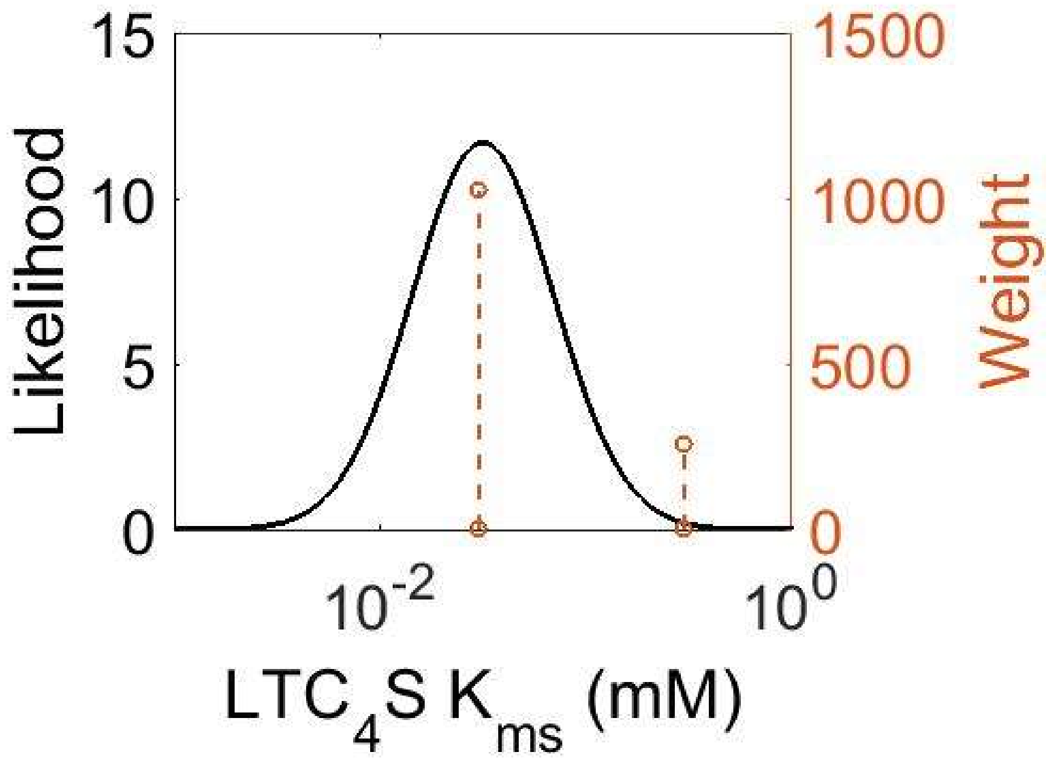


**Figure SF.7.11.1.1.1.** The estimated probability distribution for the $LTC_4S$ $K_{ms}$. The value and weight of the literature values used to define the distribution are indicated by an orange dashed line. The x axis is plotted on a log-scale.

### S.7.11.1.2. Parameter: $LTC_4S$ $K_{mp}$ (Dependent parameter)

The log-normal distribution for the parameter for the $K_{mp}$ of Reaction 16 was calculated using multivariate distributions. The log-normal distribution properties for the $K_{mp}$ of Reaction 16 are shown in Table ST.7.11.1.2.1 and plotted in Figure SF.7.11.1.2.1.

**Table ST.7.11.1.2.1.** The log-normal distribution properties of the $LTC_4S$ $K_{mp}$ distribution. These values were calculated using the functions described in Section 2.6.2.

| Mode (mM) | Location parameter (μ) | Scale parameter (σ) |
|---|---|---|
| $3.18 \times 10^{-2}$ | -2.82 | $7.92 \times 10^{-1}$ |

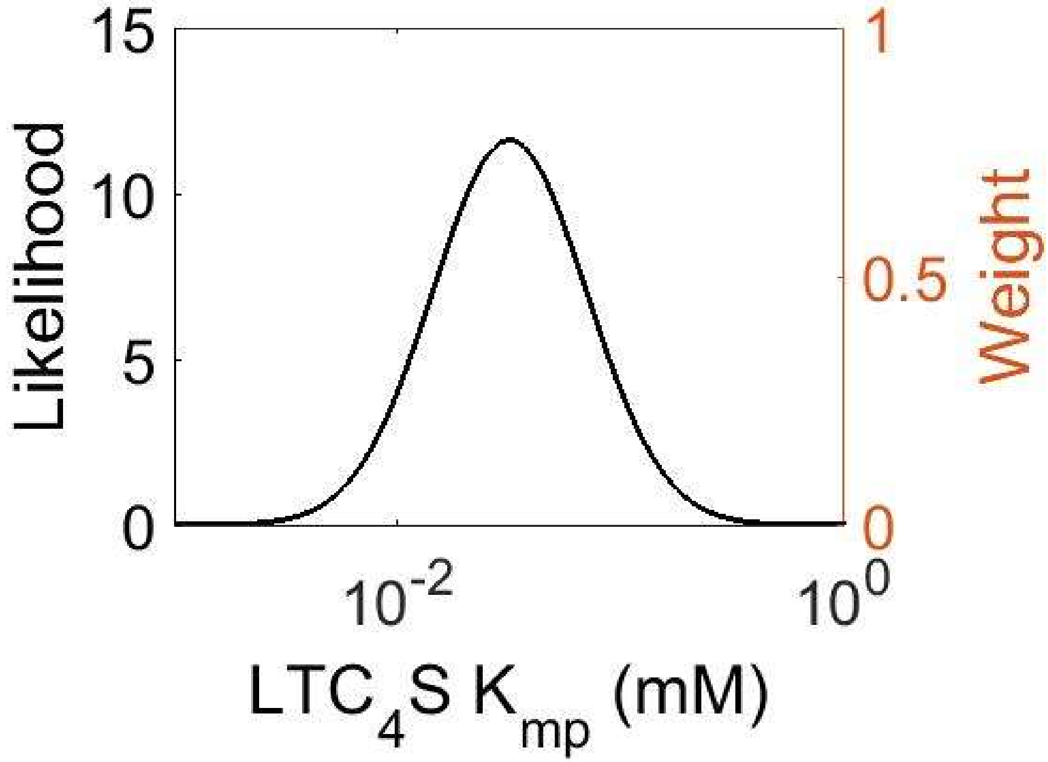


**Figure SF.7.11.1.2.1.** The estimated probability distribution for the $LTC_4S$ $K_{mp}$. The value and weight of the literature values used to define the distribution are indicated by an orange dashed line. The x axis is plotted on a log-scale.

### S.7.11.1.3. Parameter: $LTC_4S$ $k_{cat}$

Parameter values for the $k_{cat}$ of Reaction 16 were obtained from the literature and summarised in Table ST.7.11.1.3.1. The log-normal distribution properties for the $k_{cat}$ of Reaction 16 are shown in Table ST.7.11.1.3.2 and plotted in Figure SF.7.11.1.3.1.

**Table ST.7.11.1.3.1.** Literature information used to design the $LTC_4S$ $k_{cat}$ parameter distribution. Each value was assigned a weight using the protocol described in Section 2.6.2. The type of error was assigned as 0 if it was additive and 1 if it was multiplicative.

| Value ($min^{-1}$) | Error ($min^{-1}$) | Experimental details | | | | | | Weight | Type of error | Reference |
|---|---|---|---|---|---|---|---|---|---|---|
| | | Species | Expression Vector | Enzyme | pH | Temperature (°C) | Other | | | |
| 7.02 $x10^2$ | NaN | Human | E. coli. | hLTC4S | 7.8 | 20 | | 256 | 0 | (Rinaldo-Matthis et al., 2010) |
| 1.56 $x10^3$ | 2.40 $x10^2$ | Human | E. coli. | LTC4S | 7.8 | 37 | | 1024 | 0 | (Niegowski et al., 2013) |

**Table ST.7.11.1.3.2.** The log-normal distribution properties of the $LTC_4S$ $k_{cat}$ distribution. These values were calculated using the functions described in Section 2.6.2.

| Mode ($min^{-1}$) | Confidence Interval | Location parameter (μ) | Scale parameter (σ) |
|---|---|---|---|
| 1.47 $x10^3$ | 1.42 | 7.40 | 3.29 $x10^{-1}$ |

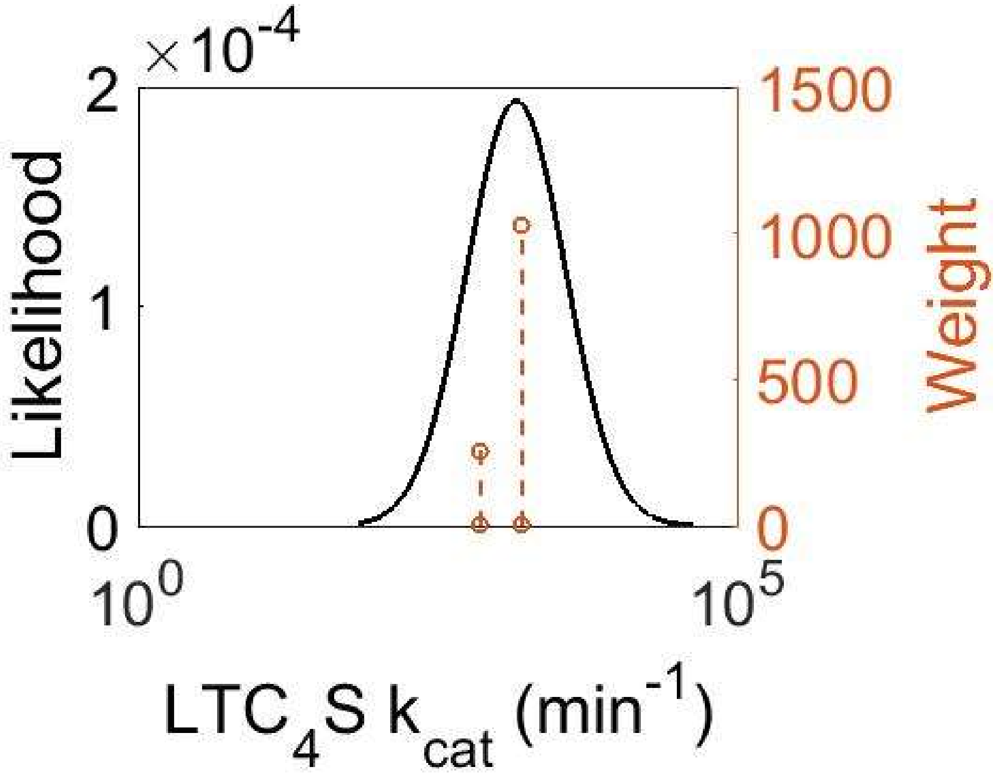


**Figure SF.7.11.1.3.1.** The estimated probability distribution for the LTC4S $k_{cat}$. The value and weight of the literature values used to define the distribution are indicated by an orange dashed line. The x axis is plotted on a log-scale.

#### S.7.11.1.4. Parameter: $LTC_4S$ $K_{eq}$

Parameter values for the $K_{eq}$ of Reaction 16 were obtained from the literature and summarised in Table ST.7.11.1.4.1. The log-normal distribution properties for the $K_{eq}$ of Reaction 16 are shown in Table ST.7.11.1.4.2 and plotted in Figure SF.7.11.1.4.1.

**Table ST.7.11.1.4.1.** Literature information used to design the $LTC_4S$ $K_{eq}$ parameter distribution. Each value was assigned a weight using the protocol described in Section 2.6.2. The type of error was assigned as 0 if it was additive and 1 if it was multiplicative.

| ΔG (kcal/mol) | $K_{eq}$ | Experimental details | | | | | | Weight | Type of error | Reference |
|---|---|---|---|---|---|---|---|---|---|---|
| | | Species | Expression Vector | Enzyme | pH | Temperature (°C) | Other | | | |
| 9.93 | 5.13 x10$^{-8}$ | Human | Unknown | $LTC_4S$ | 7 | Unknown | | 64 | 0 | (Caspi et al., 2018) |

**Table ST.7.11.1.4.2.** The log-normal distribution properties of the $LTC_4S$ $K_{eq}$ distribution. These values were calculated using the functions described in Section 2.6.2.

| Mode | Confidence Interval | Location parameter (μ) | Scale parameter (σ) |
|---|---|---|---|
| 5.13 x10$^{-8}$ | 1.00 x10$^{1}$ | -1.60 x10$^{1}$ | 8.90 x10$^{-1}$ |

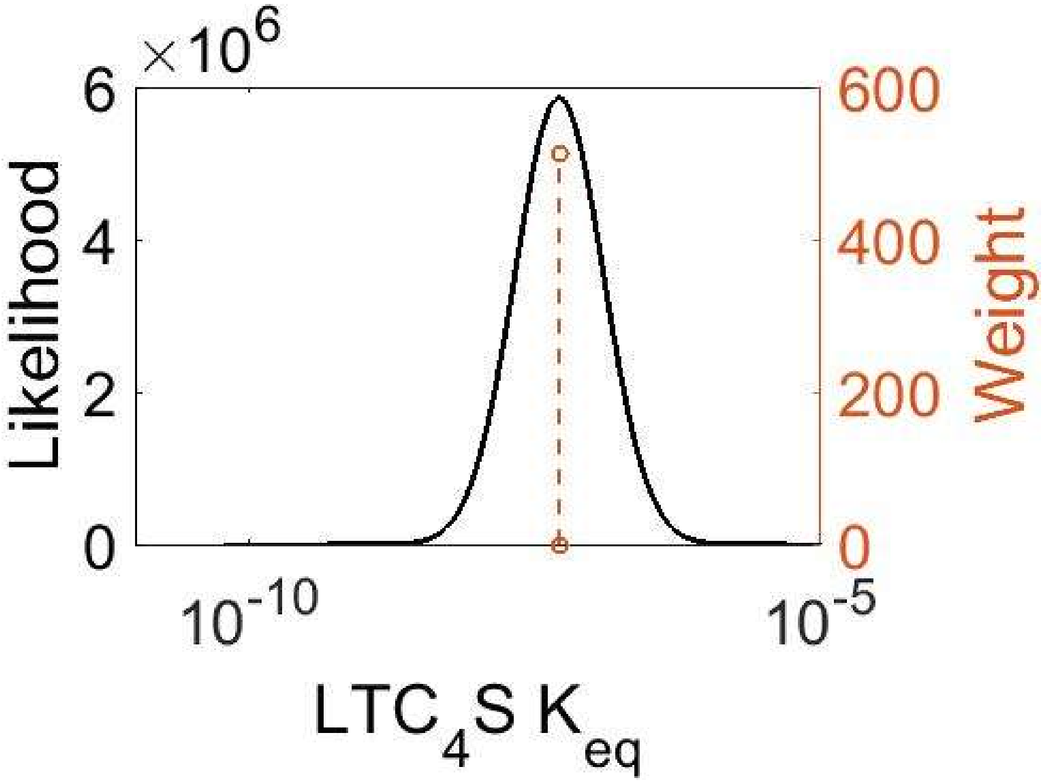


**Figure SF.7.11.1.4.1.** The estimated probability distribution for the $LTC_4S$ $K_{eq}$. The value and weight of the literature values used to define the distribution are indicated by an orange dashed line. The x axis is plotted on a log-scale.

### S.7.11.1.5. Parameter: $LTC_4S$ concentration

Parameter values for the $LTC_4S$ concentration of Reaction 16 were obtained from the literature and summarised in Table ST.7.11.1.5.1. The log-normal distribution properties for the $LTC_4S$ concentration of Reaction 16 are shown in Table ST.7.11.1.5.2 and plotted in Figure SF.7.11.1.5.1. To convert the enzyme concentration from ppm to mM, **Equation S.6.2** was used.

**Table ST.7.11.1.5.1.** Literature information used to design the $LTC_4S$ concentration parameter distribution. Each value was assigned a weight using the protocol described in Section 2.6.2. The type of error was assigned as 0 if it was additive and 1 if it was multiplicative.

| Value (ppm) | Error (ppm) | Experimental details | | | | | | Weight | Type of error | Reference |
|---|---|---|---|---|---|---|---|---|---|---|
| | | Species | Expression Vector | Enzyme | pH | Temperature (°C) | Other | | | |
| 1.39 $x10^{1}$ | NaN | Human | Adrenal gland | LTC4S | 7.5 | 37 | | **1024** | 0 | (Kim et al., 2014) |
| 2.68 $x10^{1}$ | NaN | Human | Lung | LTC4S | 7.5 | 37 | | **1024** | 0 | (Kim et al., 2014) |
| 3.30 $x10^{1}$ | NaN | Human | Oesophagus | LTC4S | 7.5 | 37 | | **2048** | 0 | (Kim et al., 2014) |

**Table ST.7.11.1.5.2.** The log-normal distribution properties of the $LTC_4S$ concentration distribution. These values were calculated using the functions described in Section 2.6.2.

| Mode (ppm) | Mode (mM) | Confidence Interval | Location parameter (μ) | Scale parameter (σ) |
|---|---|---|---|---|
| 2.90 $x10^{1}$ | 1.60 $x10^{-4}$ | 1.44 | 3.49 | 3.46 $x10^{-1}$ |

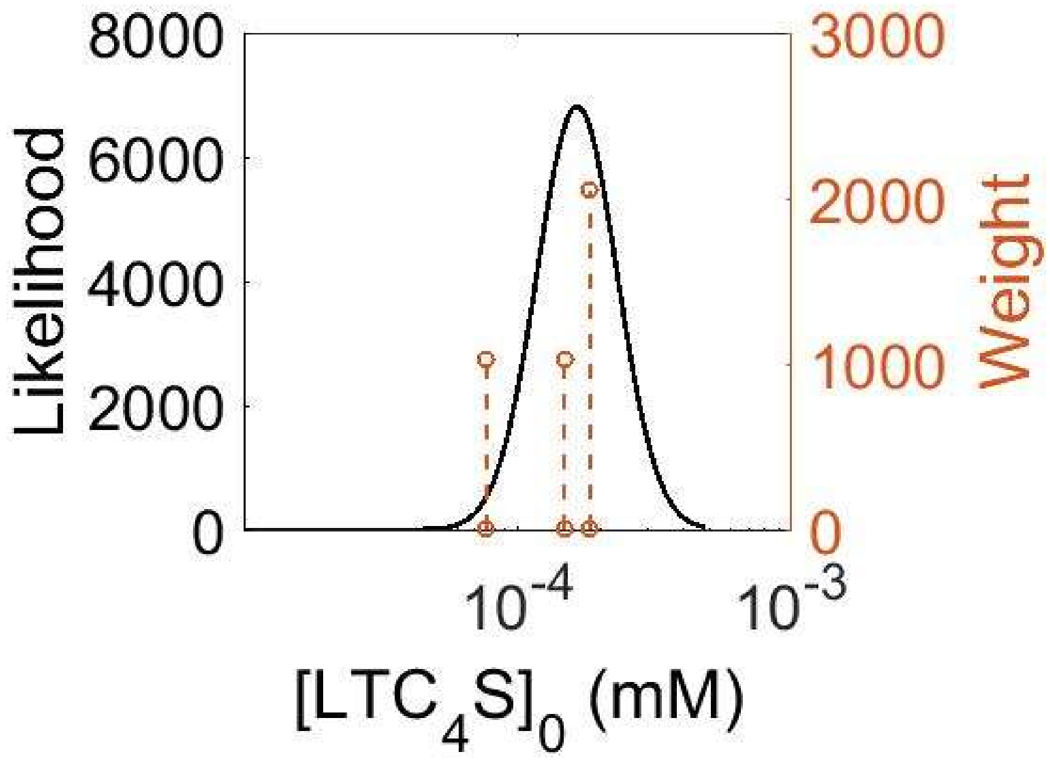


**Figure SF.7.11.1.5.1.** The estimated probability distribution for the LTC4S concentration. The value and weight of the literature values used to define the distribution are indicated by an orange dashed line. The x axis is plotted on a log-scale.

### S.7.12 Reaction 17: AA ⇌ 15-HPETE

The gene ALOX15B encodes the formation of 15-LOX-2, which converts AA exclusively into 15(S)-HPETE. Whereas the gene ALOX15 encodes the 15-LOX-1 protein, which converts AA into both 12(S)-HPETE and 15(S)-HPETE in a ratio of 1 to 16 (Bryant, Bailey et al. 1982). The formation of the hydroperoxy fatty acids (HPETE) begins with the abstraction of a hydrogen radical at the allylic position between two double bonds. The structure undergoes a rearrangement reaction which results in the formation of a conjugated diene system. The insertion of molecular oxygen and a hydrogen leads to the formation of the final structure, a hydroperoxy fatty acid.

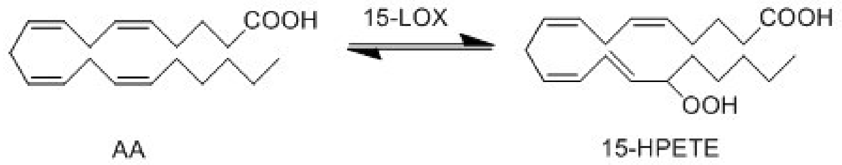


**Figure SF.7.17.** The metabolism of arachidonic acid (AA) into 15-hydroperoxyeicosatetraenoic acid (15-HPETE) by 15-lipoxygenase (15-LOX) (Reaction 17).

S.7.12.1. **Reaction rate law for Reaction 17.**

$$\boldsymbol{v_{17}} = \frac{K_{cat}\cdot[15-LOX]\left([AA]-{[15-HPETE]}/{K_{eq}}\right)}{K_{m_S}\left(1+\frac{[15-HPETE]}{K_{m_p}}\right)+[AA]}$$

### S.7.12.2. Reaction parameters

#### S.7.12.2.1. Parameter: 15-LOX $K_{ms}$

Parameter values for the $K_{ms}$ of Reaction 17 were obtained from the literature and summarised in Table ST.7.12.1.1.1. The log-normal distribution properties for the $K_{ms}$ of Reaction 17 are shown in Table ST.7.12.1.1.2 and plotted in Figure SF.7.12.1.1.1.

**Table ST.7.12.1.1.1.** Literature information used to design the 15-LOX $K_{ms}$ parameter distribution. Each value was assigned a weight using the protocol described in Section 2.6.2. The type of error was assigned as 0 if it was additive and 1 if it was multiplicative.

| Value (mM) | Error (mM) | Experimental details | | | | | | Weight | Type of error | Reference |
|---|---|---|---|---|---|---|---|---|---|---|
| | | Species | Expression Vector | Enzyme | pH | Temperature (°C) | Other | | | |
| 1.17 x$10^{-2}$ | 9.00 x$10^{-4}$ | Human | Baculovirus | 15-LOX | 6.8 | 37 | | 512 | 0 | (Sloane et al., 1995) |
| 3.70 x$10^{-3}$ | 3.00 x$10^{-4}$ | Human | Epithelium | 15-LOX | 7.5 | 25 | | 512 | 0 | (C. Jacquot et al., 2008) |
| 5.00 x$10^{-3}$ | NaN | Human | Reticulocyte | 15-LOX | 7.5 | Unknown | | 512 | 0 | (Cyril Jacquot et al., 2008) |
| 1.06 x$10^{-2}$ | NaN | Human | Keratinocyte | 15-LOX | 6.7 | 37 | | 2048 | 0 | (Burrall et al., 1988) |

**Table ST.7.12.1.1.2.** The log-normal distribution properties of the 15-LOX $K_{ms}$ distribution. These values were calculated using the functions described in Section 2.6.2.

| Mode (mM) | Confidence Interval | Location parameter (μ) | Scale parameter (σ) |
|---|---|---|---|
| 1.02 x$10^{-2}$ | 2.40 | -4.42 | 4.10 x$10^{-1}$ |

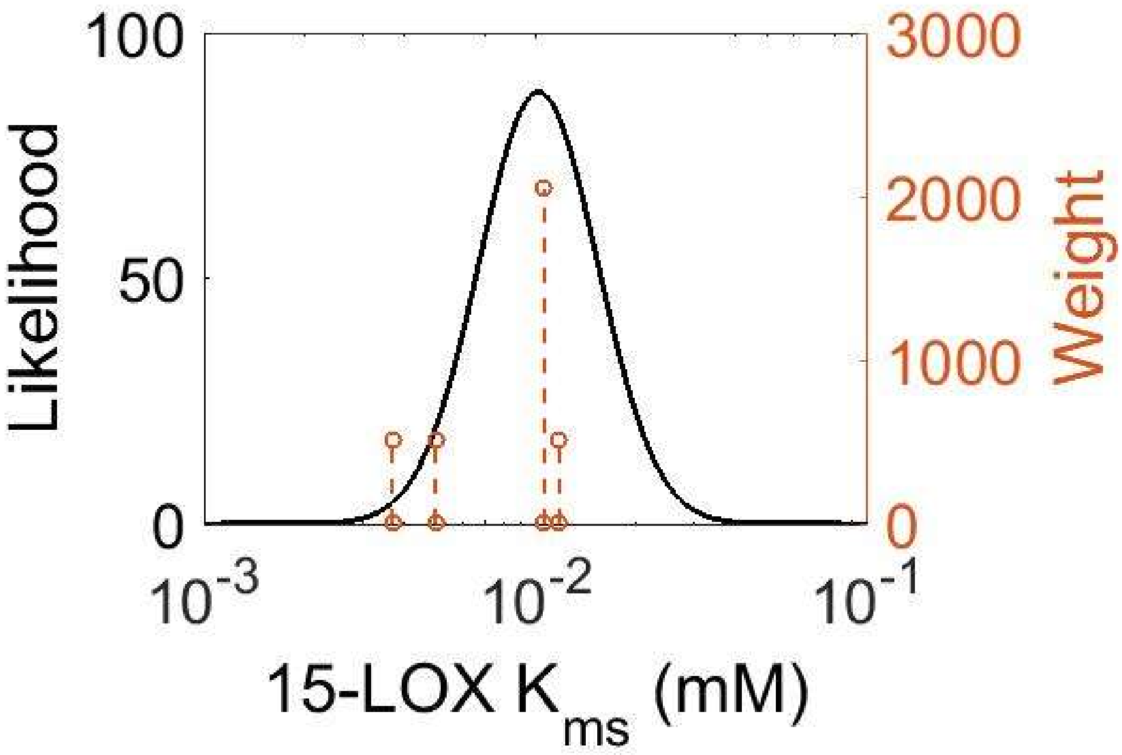


**Figure SF.7.12.1.1.1.** The estimated probability distribution for the 15-LOX $K_{ms}$. The value and weight of the literature values used to define the distribution are indicated by an orange dashed line. The x axis is plotted on a log-scale.

**S.7.12.2.2. Parameter: 15-LOX $K_{mp}$ (Dependent parameters)**

The log-normal distribution for the parameter for the $K_{mp}$ of Reaction 17 was calculated using multivariate distributions. The log-normal distribution properties for the $K_{mp}$ of Reaction 17 are shown in Table ST.7.12.1.2.1 and plotted in Figure SF.7.12.1.2.1.

**Table ST.7.12.1.2.1.** The log-normal distribution properties of the 15-LOX $K_{mp}$ distribution. These values were calculated using the functions described in Section 2.6.2.

| **Mode (mM)** | **Location parameter (μ)** | **Scale parameter (σ)** |
|---|---|---|
| 1.02 x10$^{-2}$ | -4.42 | 4.11 x10$^{-1}$ |

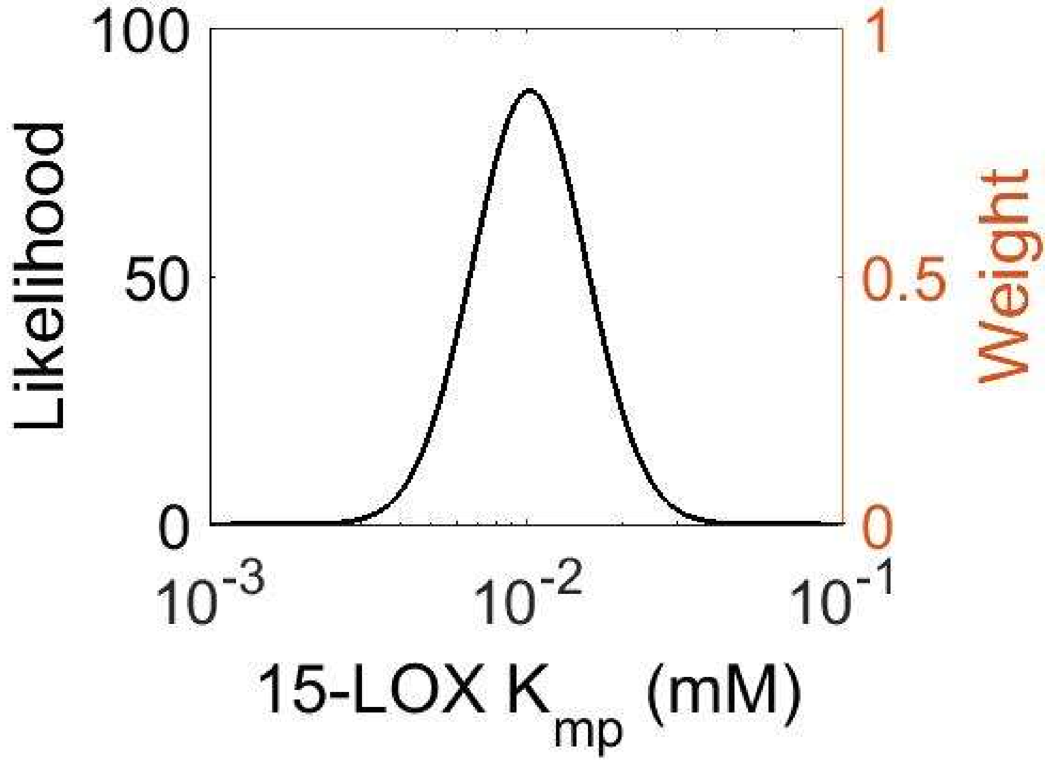


**Figure SF.7.12.1.2.1.** The estimated probability distribution for the 15-LOX $K_{mp}$. The value and weight of the literature values used to define the distribution are indicated by an orange dashed line. The x axis is plotted on a log-scale.

### S.7.12.2.3. Parameter: 15-LOX $k_{cat}$

Parameter values for the $k_{cat}$ of Reaction 17 were obtained from the literature and summarised in Table ST.7.12.1.3.1. The log-normal distribution properties for the $k_{cat}$ of Reaction 17 are shown in Table ST.7.12.1.3.2 and plotted in Figure SF.7.12.1.3.1.

**Table ST.7.12.1.3.1.** Literature information used to design the 15-LOX $k_{cat}$ parameter distribution. Each value was assigned a weight using the protocol described in Section 2.6.2. The type of error was assigned as 0 if it was additive and 1 if it was multiplicative.

| Value ($min^{-1}$) | Error ($min^{-1}$) | Experimental details | | | | | | Weight | Type of error | Reference |
|---|---|---|---|---|---|---|---|---|---|---|
| | | Species | Expression Vector | Enzyme | pH | Temperature (°C) | Other | | | |
| $3.42 \times 10^{1}$ | $6.00 \times 10^{-1}$ | Human | Epithelial | 15-LOX-2 | 7.5 | 15 | | 512 | 0 | (A. T. Wecksler et al., 2009) |
| $3.72 \times 10^{1}$ | 1.80 | Human | Epithelial | 15-LOX-2 | 7 | 22 | | 512 | 0 | (A. T. Wecksler et al., 2009) |
| $4.44 \times 10^{1}$ | 2.40 | Human | Epithelial | 15-LOX-2 | 8 | 22 | | 256 | 0 | (A. T. Wecksler et al., 2009) |
| $4.50 \times 10^{1}$ | 1.20 | Human | Epithelial | 15-LOX-2 | 7.5 | 22 | | 512 | 0 | (A. T. Wecksler et al., 2009) |
| $4.50 \times 10^{1}$ | 1.20 | Human | Epithelial | 15-LOX-2 | 7.5 | 22 | | 512 | 0 | (A. T. Wecksler et al., 2009) |
| $6.24 \times 10^{1}$ | 4.20 | Human | Epithelial | 15-LOX-2 | 7.5 | 30 | | 512 | 0 | (A. T. Wecksler et al., 2009) |
| $8.28 \times 10^{1}$ | 4.80 | Human | Epithelial | 15-LOX-2 | 7.5 | 37 | | 2048 | 0 | (A. T. Wecksler et al., 2009) |

| | | | | | | | | | | |
|---|---|---|---|---|---|---|---|---|---|---|
| $5.96 \times 10^{2}$ | $1.68 \times 10^{1}$ | Human | Epithelial | 15-LOX | 7.5 | 25 | | 512 | 0 | (C. Jacquot et al., 2008) |

**Table ST.7.12.1.3.2.** The log-normal distribution properties of the 15-LOX $k_{cat}$ distribution. These values were calculated using the functions described in Section 2.6.2.

| **Mode ($min^{-1}$)** | **Confidence Interval** | **Location parameter (μ)** | **Scale parameter (σ)** |
|---|---|---|---|
| $6.51 \times 10^{1}$ | 4.62 | 4.60 | $6.50 \times 10^{-1}$ |

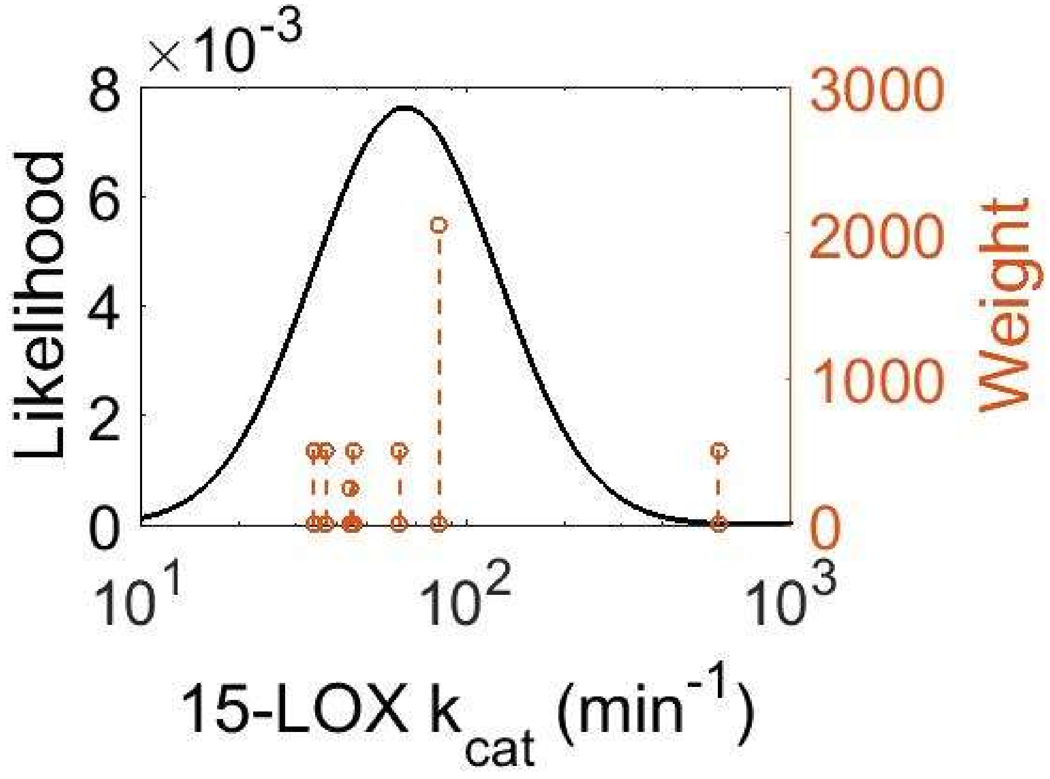


**Figure SF.7.12.1.3.1.** The estimated probability distribution for the 15-LOX $k_{cat}$. The value and weight of the literature values used to define the distribution are indicated by an orange dashed line. The x axis is plotted on a log-scale.

### S.7.12.2.4. Parameter: 15-LOX $K_{eq}$

Parameter values for the $K_{eq}$ of Reaction 17 were obtained from the literature and summarised in Table ST.7.12.1.4.1. The log-normal distribution properties for the $K_{eq}$ of Reaction 17 are shown in Table ST.7.12.1.4.2 and plotted in Figure SF.7.12.1.4.1.

**Table ST.7.12.1.4.1.** Literature information used to design the 15-LOX $K_{eq}$ parameter distribution. Each value was assigned a weight using the protocol described in Section 2.6.2. The type of error was assigned as 0 if it was additive and 1 if it was multiplicative.

| ΔG (kcal/mol) | $K_{eq}$ | Experimental details | | | | | | Weight | Type of error | Reference |
|---|---|---|---|---|---|---|---|---|---|---|
| | | Species | Expression Vector | Enzyme | pH | Temperature (°C) | Other | | | |
| -7.00 x10$^{1}$ | 2.27 x10$^{51}$ | Human | Unknown | 5-LOX | 7 | Unknown | | 64 | 0 | (Caspi et al., 2018) |

**Table ST.7.12.1.4.2.** The log-normal distribution properties of the 15-LOX $K_{eq}$ distribution. These values were calculated using the functions described in Section 2.6.2.

| Mode | Confidence Interval | Location parameter (μ) | Scale parameter (σ) |
|---|---|---|---|
| 2.27 x10$^{51}$ | 1.00 x10$^{1}$ | 1.19 x10$^{2}$ | 8.90 x10$^{-1}$ |

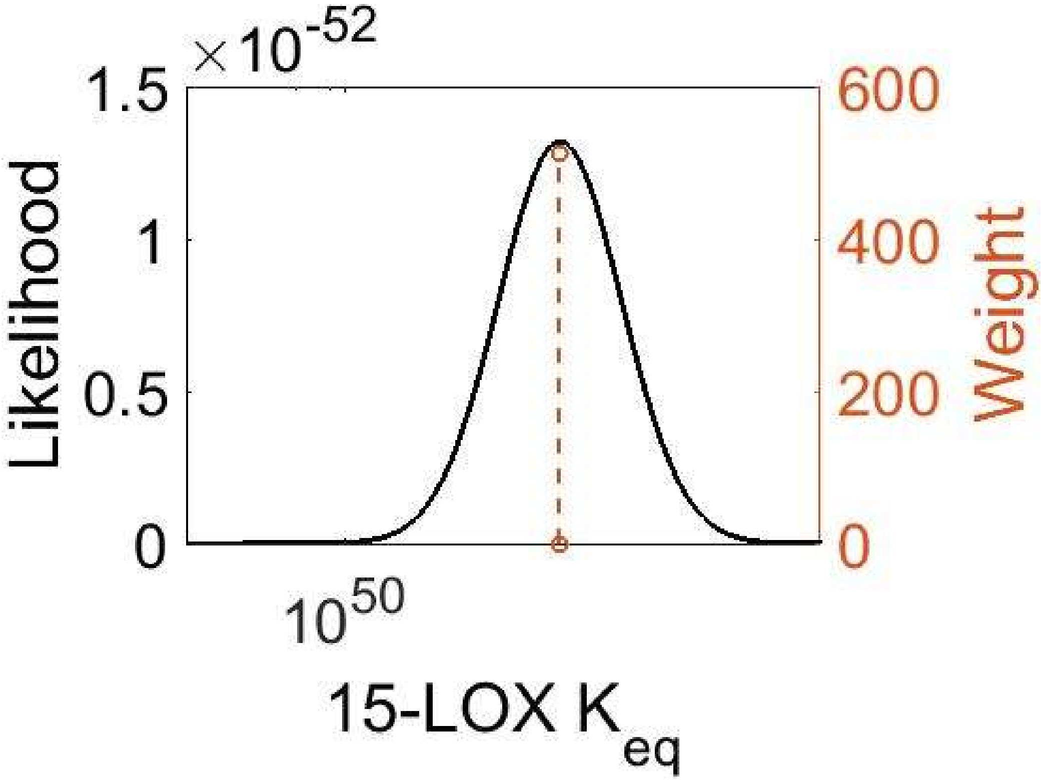


**Figure SF.7.12.1.4.1.** The estimated probability distribution for the 15-LOX $K_{eq}$. The value and weight of the literature values used to define the distribution are indicated by an orange dashed line. The x axis is plotted on a log-scale.

### S.7.12.2.5. Parameter: 15-LOX concentration

Parameter values for the 15-LOX concentration of Reaction 17 were obtained from the literature and summarised in Table ST.7.12.1.5.1. The log-normal distribution properties for the 15-LOX concentration of Reaction 17 are shown in Table ST.7.12.1.5.2 and plotted in Figure SF.7.12.1.5.1. To convert the enzyme concentration from ppm to mM, **Equation S.6.2** was used.

**Table ST.7.12.1.5.1.** Literature information used to design the 15-LOX concentration parameter distribution. Each value was assigned a weight using the protocol described in Section 2.6.2. The type of error was assigned as 0 if it was additive and 1 if it was multiplicative.

| Value (ppm) | Error (ppm) | Experimental details | | | | | | Weight | Type of error | Reference |
|---|---|---|---|---|---|---|---|---|---|---|
| | | Species | Expression Vector | Enzyme | pH | Temperature (°C) | Other | | | |
| 1.40 | NaN | Human | Gut | 15-LOX | 7.5 | 37 | | **1024** | 0 | (Kim et al., 2014) |
| 4.09 | NaN | Human | Lung | 15-LOX | 7.5 | 37 | | **1024** | 0 | (Kim et al., 2014) |
| $3.74 \times 10^{1}$ | NaN | Human | Spleen | 15-LOX | 7.5 | 37 | | **1024** | 0 | (Wilhelm et al., 2014) |

**Table ST.7.12.1.5.2.** The log-normal distribution properties of the 15-LOX concentration distribution. These values were calculated using the functions described in Section 2.6.2.

| Mode (ppm) | Mode (mM) | Confidence Interval | Location parameter (μ) | Scale parameter (σ) |
|---|---|---|---|---|
| 4.07 | 2.25E-05 | 7.23 | $7.12 \times 10^{-1}$ | 1.19 |

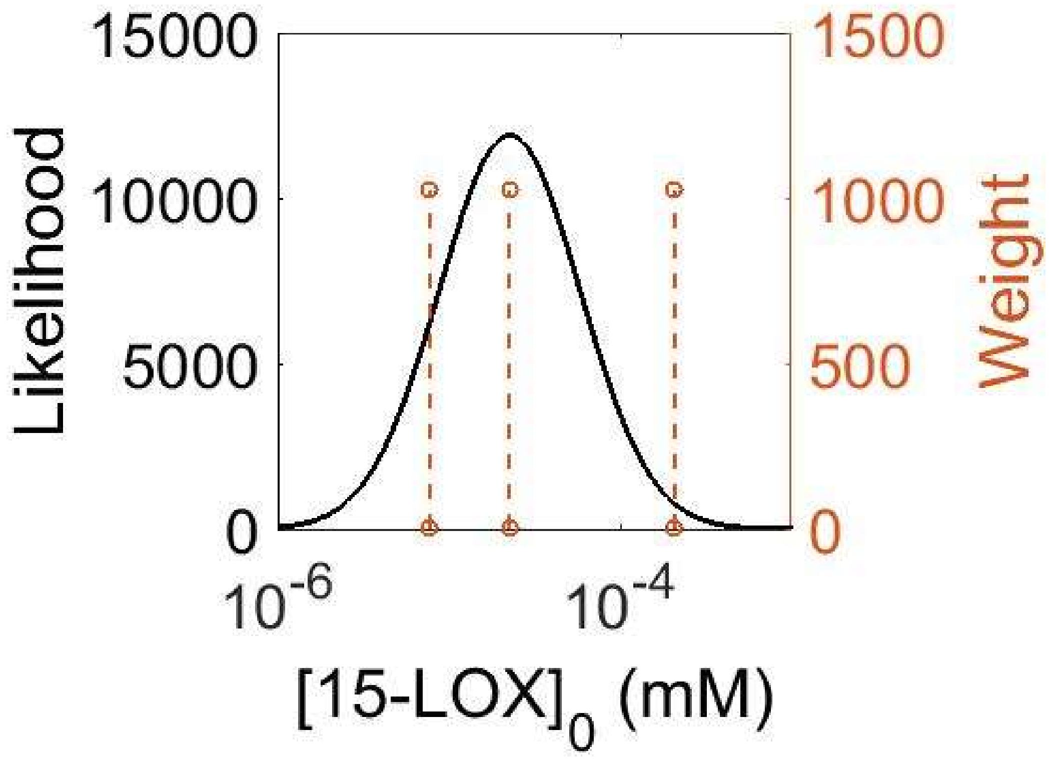


**Figure SF.7.12.1.5.1.** The estimated probability distribution for the 15-LOX concentration. The value and weight of the literature values used to define the distribution are indicated by an orange dashed line. The x axis is plotted on a log-scale.

### S.7.13 Reaction 18: 15-HPETE ⇌ 15-HETE

15-HPETE is reduced by an oxidoreductase enzyme (PHGPx) to form 15-HETE.

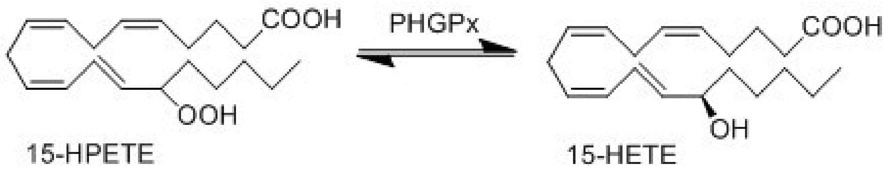


**Figure SF.7.13.** The metabolism of 15-hydroperoxyeicosatetraenoic acid (15-HPETE) into 15-hydroxyeicosatetraenoic acid (15-HETE) by hydroperoxide glutathione peroxidase (PHGPx) (Reaction 18).

**SEq.7.13.** Reaction rate law for Reaction 18.

$$\boldsymbol{v_{18}} = \frac{K_{cat} \cdot [PHGPx]\left([15-HPETE] - {[15-HETE]}/{K_{eq}}\right)}{K_{m_s}\left(1 + \frac{[15-HETE]}{K_{mp}}\right) + [15-HPETE]}$$

### S.7.13.1. Reaction parameters

#### S.7.13.1.1. Parameter: PHGPx $K_{ms}$

Parameter values for the $K_{ms}$ of Reaction 18 were obtained from the literature and summarised in Table ST.7.13.1.1.1. The log-normal distribution properties for the $K_{ms}$ of Reaction 18 are shown in Table ST.7.13.1.1.2 and plotted in Figure SF.7.13.1.1.1.

**Table ST.7.13.1.1.1.** Literature information used to design the PHGPx $K_{ms}$ parameter distribution. Each value was assigned a weight using the protocol described in Section 2.6.2. The type of error was assigned as 0 if it was additive and 1 if it was multiplicative.

| Value (mM) | Error (mM) | Experimental details | | | | | | Weight | Type of error | Reference |
|---|---|---|---|---|---|---|---|---|---|---|
| | | Species | Expression Vector | Enzyme | pH | Temperature (°C) | Other | | | |
| 4.00 x$10^{-3}$ | NaN | Rat | Liver | PHGPx | 7.4 | 37 | | 768 | 0 | (Hiratsuka et al., 1997) |
| 3.00 x$10^{-1}$ | NaN | Human | Bio imprinted Enzyme | PHGPx | 7 | 37 | | 512 | 0 | (Liu et al., 2008) |
| 1.11 x$10^{1}$ | 2.90 x$10^{-1}$ | Human | E. coli. | PHGPx | 7 | 37 | | 1024 | 0 | (Zheng et al., 2008) |

**Table ST.7.13.1.1.2.** The log-normal distribution properties of the PHGPx $K_{ms}$ distribution. These values were calculated using the functions described in Section 2.6.2.

| Mode (mM) | Confidence Interval | Location parameter (μ) | Scale parameter (σ) |
|---|---|---|---|
| 3.19 x$10^{-1}$ | 1.02 x$10^{3}$ | 2.54 | 1.92 |

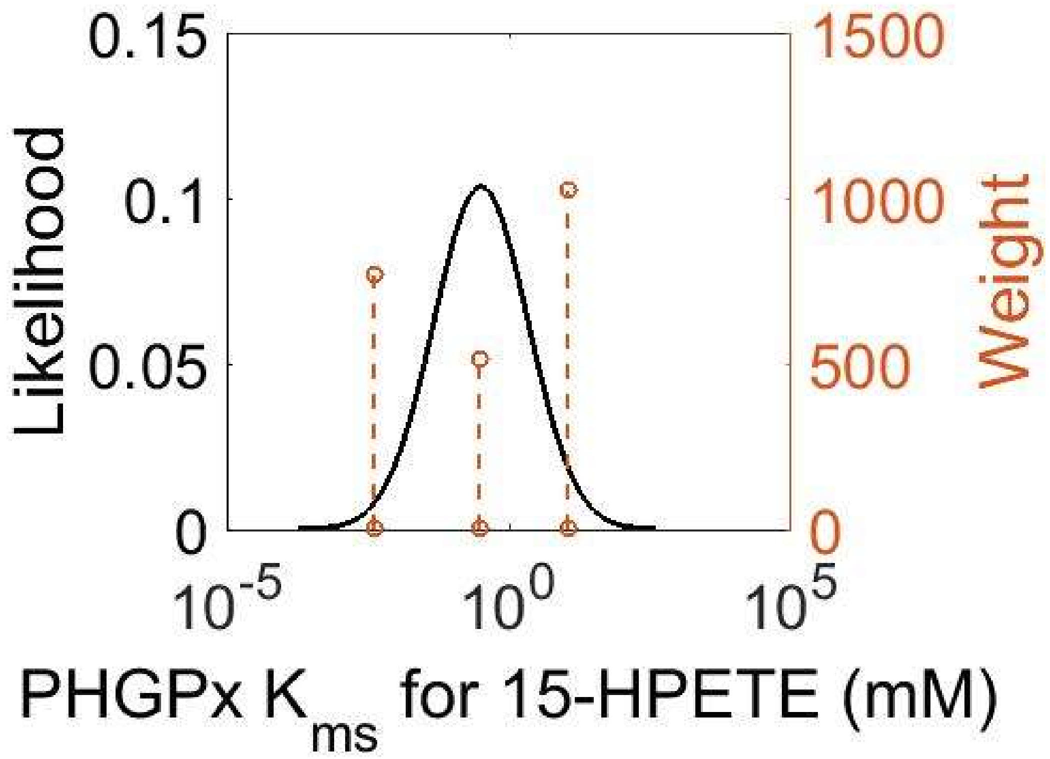


**Figure SF.7.13.1.1.1.** The estimated probability distribution for the PHGPx $K_{ms}$. The value and weight of the literature values used to define the distribution are indicated by an orange dashed line. The x axis is plotted on a log-scale.

### S.7.13.1.2. Parameter: PHGPx $K_{mp}$ (Dependent parameter)

The log-normal distribution for the parameter for the $K_{mp}$ of Reaction 18 was calculated using multivariate distributions. The log-normal distribution properties for the $K_{mp}$ of Reaction 18 are shown in Table ST.7.13.1.2.1 and plotted in Figure SF.7.13.1.2.1.

**Table ST.7.13.1.2.1.** The log-normal distribution properties of the PHGPx $K_{mp}$ distribution. These values were calculated using the functions described in Section 2.6.2.

| Mode (mM) | Location parameter (μ) | Scale parameter (σ) |
|---|---|---|
| 3.15 $x10^{-1}$ | 2.53 | 1.92 |

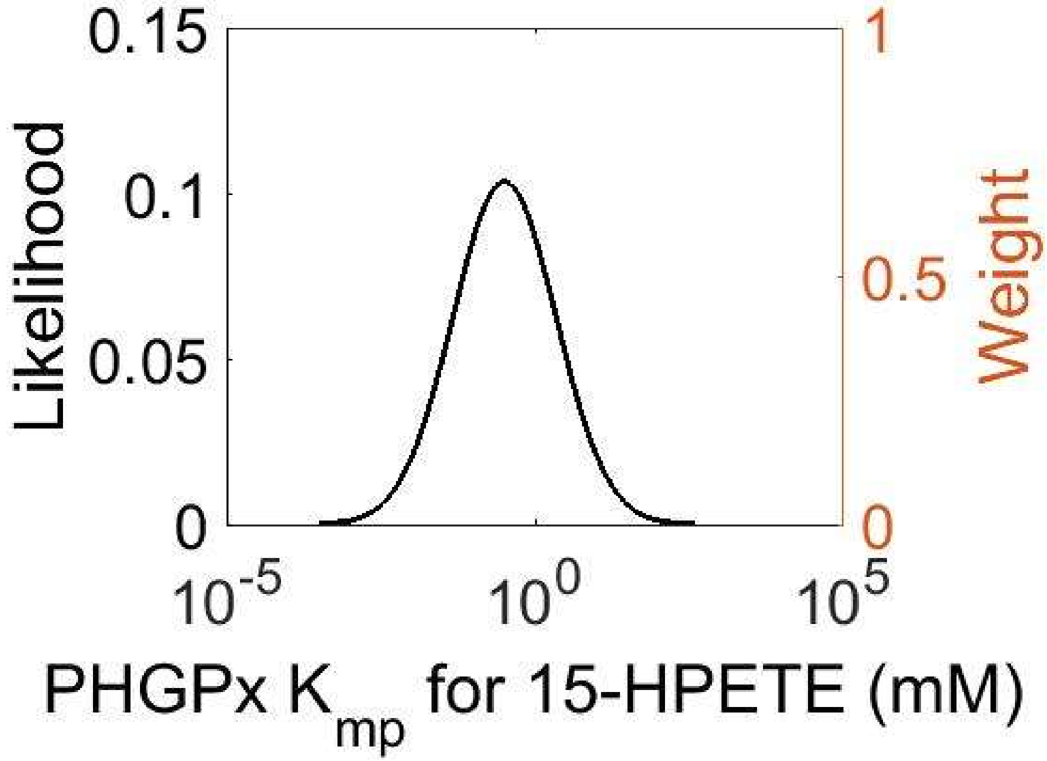


**Figure SF.7.13.1.2.1.** The estimated probability distribution for the PHGPx $K_{mp}$. The value and weight of the literature values used to define the distribution are indicated by an orange dashed line. The x axis is plotted on a log-scale.

### S.7.13.1.3. Parameter: PHGPx $k_{cat}$

Parameter values for the $k_{cat}$ of Reaction 18 were obtained from the literature and summarised in Table ST.7.13.1.3.1. The log-normal distribution properties for the $k_{cat}$ of Reaction 18 are shown in Table ST.7.13.1.3.2 and plotted in Figure SF.7.13.1.3.1.

**Table ST.7.13.1.3.1.** Literature information used to design the PHGPx $k_{cat}$ parameter distribution. Each value was assigned a weight using the protocol described in Section 2.6.2. The type of error was assigned as 0 if it was additive and 1 if it was multiplicative.

| Value ($min^{-1}$) | Error ($min^{-1}$) | Experimental details | | | | | | Weight | Type of error | Reference |
|---|---|---|---|---|---|---|---|---|---|---|
| | | Species | Expression Vector | Enzyme | pH | Temperature (°C) | Other | | | |
| 1.17 $x10^3$ | 5.00 $x10^1$ | Human | Bio imprinted Enzyme | PHGPx | 7 | 37 | | 512 | 0 | (Liu et al., 2008) |
| 2.45 $x10^4$ | 1.50 $x10^2$ | Human | E. coli. | PHGPx | 7 | 37 | | 1024 | 0 | (Zheng et al., 2008) |

**Table ST.7.13.1.3.2.** The log-normal distribution properties of the PHGPx $k_{cat}$ distribution. These values were calculated using the functions described in Section 2.6.2.

| Mode ($min^{-1}$) | Confidence Interval | Location parameter (μ) | Scale parameter (σ) |
|---|---|---|---|
| 2.44 $x10^4$ | 4.20 | 1.11 $x10^1$ | 9.75 $x10^{-1}$ |

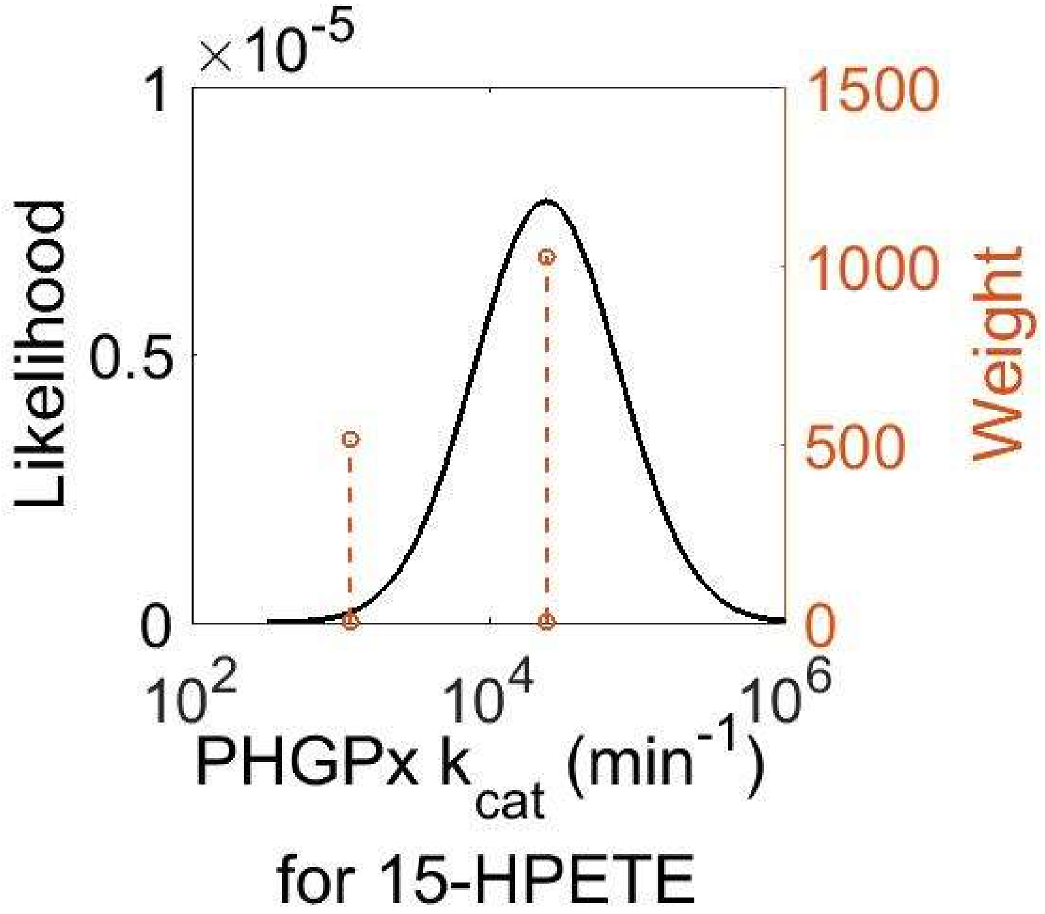


**Figure SF.7.13.1.3.1.** The estimated probability distribution for the PHGPx $k_{cat}$. The value and weight of the literature values used to define the distribution are indicated by an orange dashed line. The x axis is plotted on a log-scale.

### S.7.13.1.4. Parameter: PHGPx $K_{eq}$

Parameter values for the $K_{eq}$ of Reaction 18 were obtained from the literature and summarised in Table ST.7.13.1.4.1. The log-normal distribution properties for the $K_{eq}$ of Reaction 18 are shown in Table ST.7.13.1.4.2 and plotted in Figure SF.7.13.1.4.1.

**Table ST.7.13.1.4.1.** Literature information used to design the PHGPx $K_{eq}$ parameter distribution. Each value was assigned a weight using the protocol described in Section 2.6.2. The type of error was assigned as 0 if it was additive and 1 if it was multiplicative.

| ΔG (kcal/mol) | $K_{eq}$ | Experimental details | | | | | | Weight | Type of error | Reference |
|---|---|---|---|---|---|---|---|---|---|---|
| | | Species | Expression Vector | Enzyme | pH | Temperature (°C) | Other | | | |
| $-2.69 \times 10^{1}$ | $5.90 \times 10^{19}$ | Human | Unknown | PHGPx | 7 | Unknown | | 64 | 0 | (Caspi et al., 2018) |

**Table ST.7.13.1.4.2.** The log-normal distribution properties of the PHGPx $K_{eq}$ distribution. These values were calculated using the functions described in Section 2.6.2.

| Mode | Confidence Interval | Location parameter (μ) | Scale parameter (σ) |
|---|---|---|---|
| $5.90 \times 10^{19}$ | $1.00 \times 10^{1}$ | $4.63 \times 10^{1}$ | $8.90 \times 10^{-1}$ |

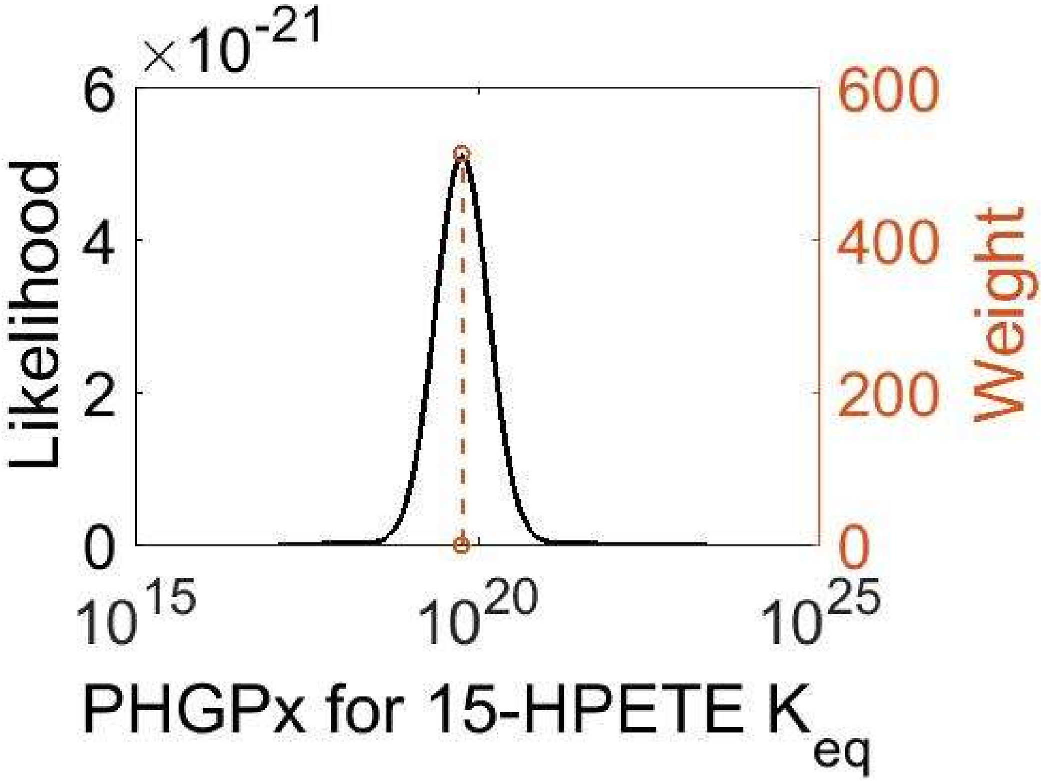


**Figure SF.7.13.1.4.1.** The estimated probability distribution for the PHGPx $K_{eq}$. The value and weight of the literature values used to define the distribution are indicated by an orange dashed line. The x axis is plotted on a log-scale.

### S.7.13.1.5. Parameter: PHGPx concentration

Parameter values for the PHGPx concentration of Reaction 18 were obtained from the literature and summarised in Table ST.7.13.1.5.1. The log-normal distribution properties for the PHGPx concentration of Reaction 18 are shown in Table ST.7.13.1.5.2 and plotted in Figure SF.7.13.1.5.1. To convert the enzyme concentration from ppm to mM, **Equation S.6.2** was used.

**Table ST.7.13.1.5.1.** Literature information used to design the PHGPx concentration parameter distribution. Each value was assigned a weight using the protocol described in Section 2.6.2. The type of error was assigned as 0 if it was additive and 1 if it was multiplicative.

| Value (ppm) | Error (ppm) | Experimental details | | | | | | Weight | Type of error | Reference |
|---|---|---|---|---|---|---|---|---|---|---|
| | | Species | Expression Vector | Enzyme | pH | Temperature (°C) | Other | | | |
| $2.04 \times 10^{2}$ | NaN | Human | Gut | PHGPx | 7.5 | 37 | | **1024** | 0 | (Kim et al., 2014) |
| $2.82 \times 10^{2}$ | NaN | Human | Oesophagus | PHGPx | 7.5 | 37 | | **1024** | 0 | (Wilhelm et al., 2014) |
| $3.07 \times 10^{2}$ | NaN | Human | Lung | PHGPx | 7.5 | 37 | | **1024** | 0 | (Wilhelm et al., 2014) |
| $4.59 \times 10^{2}$ | NaN | Human | Skin | PHGPx | 7.5 | 37 | | **2048** | 0 | (Wilhelm et al., 2014) |

**Table ST.7.13.1.5.2.** The log-normal distribution properties of the PHGPx concentration distribution. These values were calculated using the functions described in Section 2.6.2.

| Mode (ppm) | Mode (mM) | Confidence Interval | Location parameter (μ) | Scale parameter (σ) |
|---|---|---|---|---|
| $3.14 \times 10^{2}$ | $1.74 \times 10^{-3}$ | 1.38 | 5.85 | $3.09 \times 10^{-1}$ |

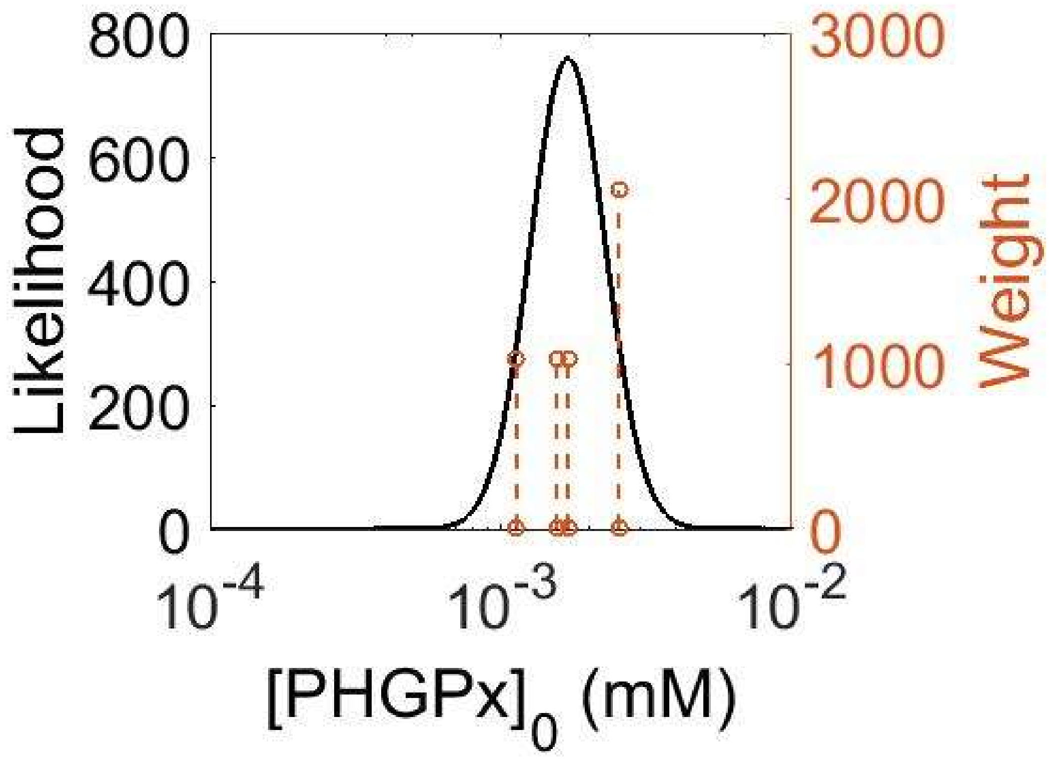


**Figure SF.7.13.1.5.1.** The estimated probability distribution for the PHGPx concentration. The value and weight of the literature values used to define the distribution are indicated by an orange dashed line. The x axis is plotted on a log-scale.

### S.7.14 Reaction 19: AA ⇌ 12-HPETE

The gene ALOX12 encodes 12-LOX, which converts AA into the S enantiomer of 12-HPETE, 12(S)-HPETE (Izumi et al., 1990). The genes ALOX12B and ALOXE3 encode the proteins 12R-LOX and eLOX3 respectively, these proteins are responsible for the generation of the R enantiomer of 12-HPETE, 12(R)-HPETE (Schwartzman et al., 1987). Interestingly, ALOX15 which encodes the 15-LOX-1 protein, is capable of producing both 12(S)-HPETE and 15(S)-HPETE in a ratio of 1 to 16 (Bryant et al., 1982). The formation of the hydroperoxy fatty acids (HPETE) begins with the abstraction of a hydrogen radical at the allylic position between two double bonds. The structure undergoes a rearrangement reaction which results in the formation of a conjugated diene system. The insertion of molecular oxygen and a hydrogen leads to the formation of the final structure, a hydroperoxy fatty acid.

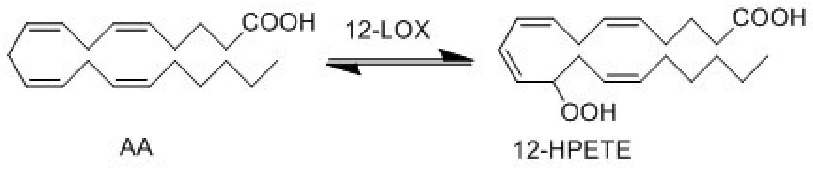


**Figure SF.7.14.** The metabolism of arachidonic acid (AA) into 12-hydroperoxyeicosatetraenoic acid (12-HPETE) by 12-lipoxygenase (12-LOX) (Reaction 19).

**SEq.7.14.** Reaction rate law for Reaction 19.

$$\boldsymbol{v_{19}} = \frac{K_{cat} \cdot [12-LOX]\left([AA] - {[12-HPETE]}/{K_{eq}}\right)}{K_{m_s}\left(1 + \frac{[12-HPETE]}{K_{m_p}}\right) + [AA]}$$

### S.7.14.1. Reaction parameters

#### S.7.14.1.1. Parameter: 12-LOX $K_{ms}$

Parameter values for the $K_{ms}$ of Reaction 18 were obtained from the literature and summarised in Table ST.7.14.1.1.1. The log-normal distribution properties for the $K_{ms}$ of Reaction 18 are shown in Table ST.7.14.1.1.2 and plotted in Figure SF.7.14.1.1.1.

**Table ST.7.14.1.1.1.** Literature information used to design the 12-LOX $K_{ms}$ parameter distribution. Each value was assigned a weight using the protocol described in Section 2.6.2. The type of error was assigned as 0 if it was additive and 1 if it was multiplicative.

| Value (mM) | Error (mM) | Experimental details | | | | | | Weight | Type of error | Reference |
|---|---|---|---|---|---|---|---|---|---|---|
| | | Species | Expression Vector | Enzyme | pH | Temperature (°C) | Other | | | |
| 7.20 x10$^{-3}$ | NaN | Human | Platelet | 12-LOX | 7.4 | 37 | | 2048 | 0 | (Lagarde et al., 1984) |
| 7.90 x10$^{-3}$ | 8.00 x10$^{-4}$ | Human | Platelet | 12-LOX | 7.4 | 37 | | 2048 | 0 | (Romano et al., 1993) |
| 1.00 x10$^{-2}$ | NaN | Human | Baculovirus | 12-LOX | 8 | 37 | | 256 | 0 | (Chen et al., 1993) |
| 8.00 x10$^{-2}$ | NaN | Human | Platelet | 12-LOX | 7.4 | 24 | | 512 | 0 | (Hada et al., 1991) |

**Table ST.7.14.1.1.2.** The log-normal distribution properties of the 12-LOX $K_{ms}$ distribution. These values were calculated using the functions described in Section 2.6.2.

| Mode (mM) | Confidence Interval | Location parameter (σ) | Scale parameter (σ) |
|---|---|---|---|
| 7.60 x10$^{-3}$ | 4.34 | -4.48 | 6.30 x10$^{-1}$ |

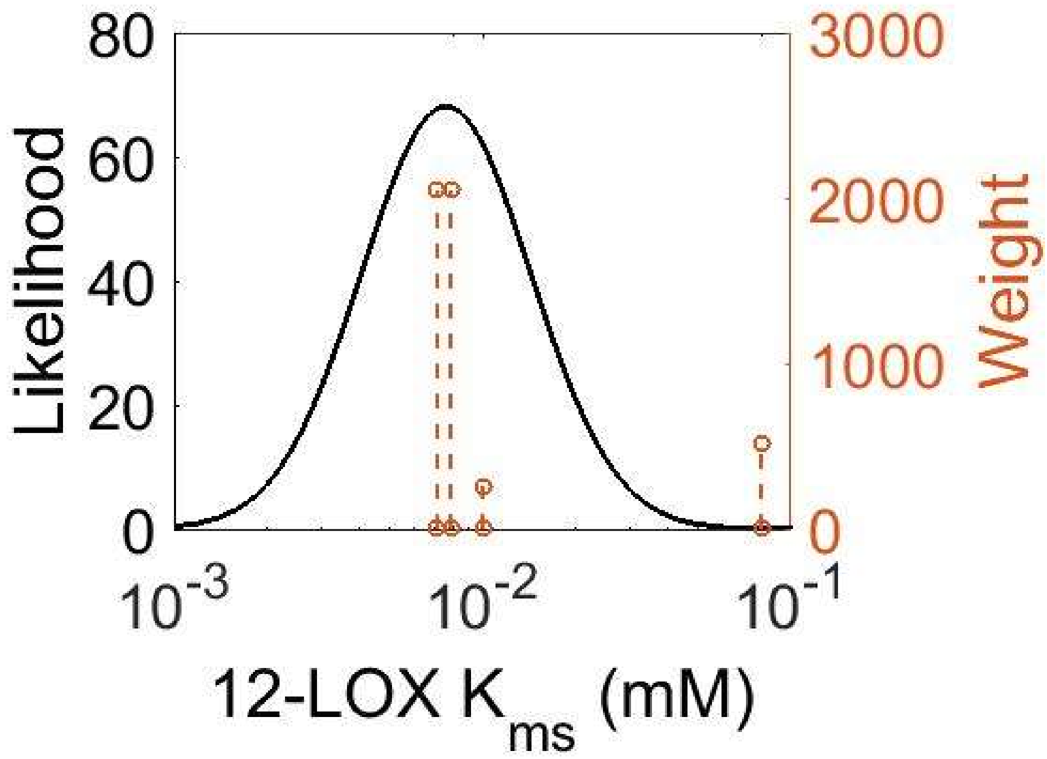


**Figure SF.7.14.1.1.1.** The estimated probability distribution for the 12-LOX $K_{ms}$. The value and weight of the literature values used to define the distribution are indicated by an orange dashed line. The x axis is plotted on a log-scale.

**S.7.14.1.2. Parameter: 12-LOX $K_{mp}$ (Dependent parameter)**

he log-normal distribution for the parameter for the $K_{mp}$ of Reaction 18 was calculated using multivariate distributions. The log-normal distribution properties for the $K_{mp}$ of Reaction 18 are shown in Table ST.7.14.1.2.1 and plotted in Figure SF.7.14.1.2.1.

**Table ST.7.14.1.2.1.** The log-normal distribution properties of the 12-LOX $K_{mp}$ distribution. These values were calculated using the functions described in Section 2.6.2.

| Mode (mM) | Location parameter (μ) | Scale parameter (σ) |
|---|---|---|
| 7.30 $x10^{-3}$ | -4.52 | 6.28 $x10^{-1}$ |

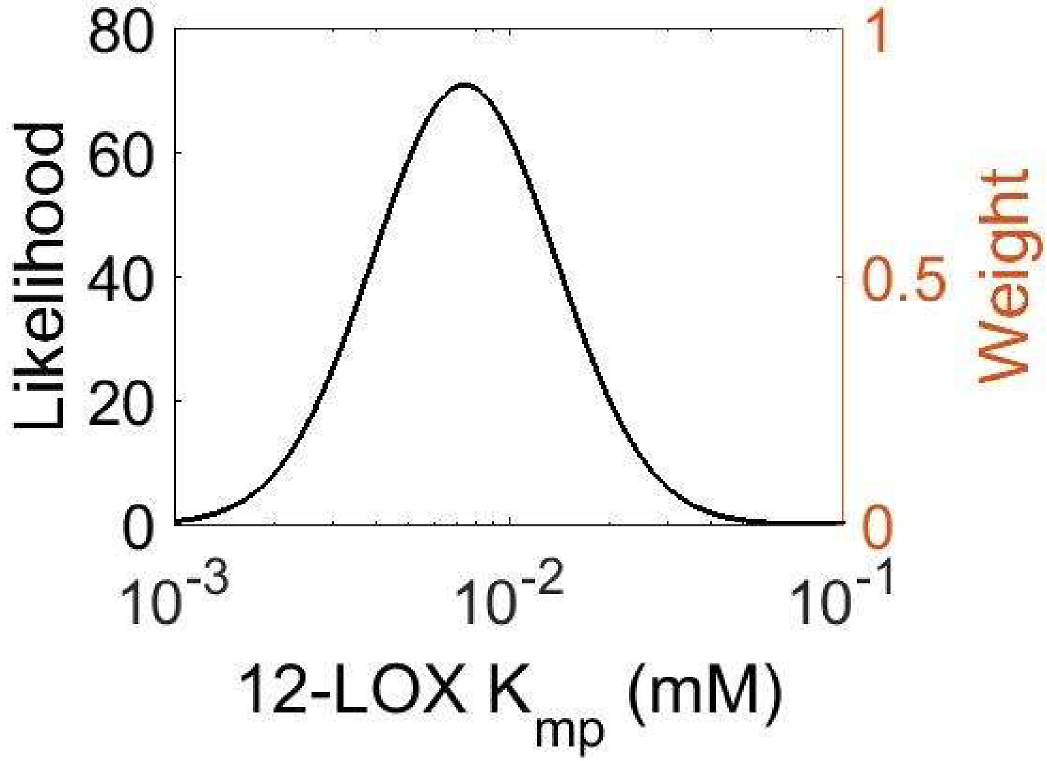


**Figure SF.7.14.1.2.1.** The estimated probability distribution for the 12-LOX $K_{mp}$. The value and weight of the literature values used to define the distribution are indicated by an orange dashed line. The x axis is plotted on a log-scale.

### S.7.14.1.3. Parameter: 12-LOX $k_{cat}$

Parameter values for the $k_{cat}$ of Reaction 18 were obtained from the literature and summarised in Table ST.7.14.1.3.1. The log-normal distribution properties for the $k_{cat}$ of Reaction 18 are shown in Table ST.7.14.1.3.2 and plotted in Figure SF.7.14.1.3.1.

**Table ST.7.14.1.3.1.** Literature information used to design the 12-LOX $k_{cat}$ parameter distribution. Each value was assigned a weight using the protocol described in Section 2.6.2. The type of error was assigned as 0 if it was additive and 1 if it was multiplicative.

| Value ($min^{-1}$) | Error ($min^{-1}$) | Experimental details | | | | | | Weight | Type of error | Reference |
|---|---|---|---|---|---|---|---|---|---|---|
| | | Species | Expression Vector | Enzyme | pH | Temperature (°C) | Other | | | |
| 5.04 $x10^2$ | NaN | Boar | E. coli. | 12-LOX | 7.4 | 37 | | 1024 | 0 | (Richards and Marnett, 1997) |
| 3.36 $x10^2$ | 1.20 $x10^1$ | Human | Reticulocyte | 12-LOX | 7.5 | 25 | | 256 | 0 | (Aaron T. Wecksler et al., 2009) |

**Table ST.7.14.1.3.2.** The log-normal distribution properties of the 12-LOX $k_{cat}$ distribution. These values were calculated using the functions described in Section 2.6.2.

| Mode ($min^{-1}$) | Confidence Interval | Location parameter (μ) | Scale parameter (σ) |
|---|---|---|---|
| 4.87 $x10^2$ | 1.20 | 6.22 | 1.80 $x10^{-1}$ |

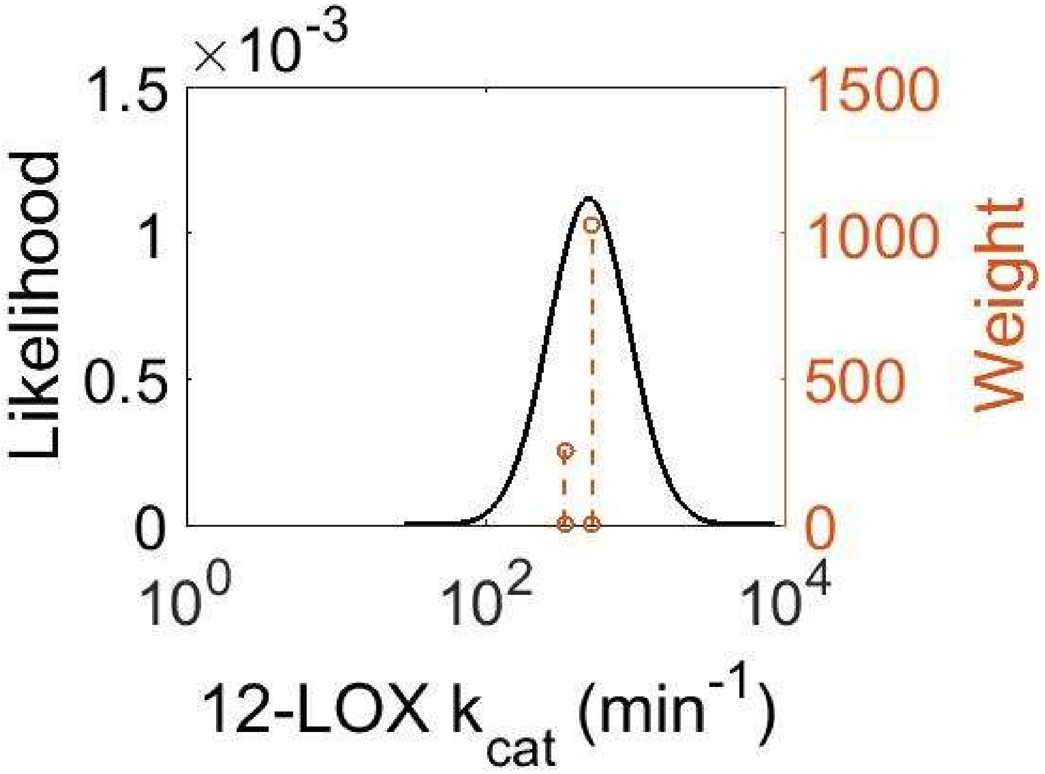


**Figure SF.7.14.1.3.1.** The estimated probability distribution for the 12-LOX $k_{cat}$. The value and weight of the literature values used to define the distribution are indicated by an orange dashed line. The x axis is plotted on a log-scale.

### S.7.14.1.4. Parameter: 12-LOX $K_{eq}$

Parameter values for the $K_{eq}$ of Reaction 18 were obtained from the literature and summarised in Table ST.7.14.1.4.1. The log-normal distribution properties for the $K_{eq}$ of Reaction 18 are shown in Table ST.7.14.1.4.2 and plotted in Figure SF.7.14.1.4.1.

**Table ST.7.14.1.4.1.** Literature information used to design the 12-LOX $K_{eq}$ parameter distribution. Each value was assigned a weight using the protocol described in Section 2.6.2. The type of error was assigned as 0 if it was additive and 1 if it was multiplicative.

| ΔG (kcal/mol) | $K_{eq}$ | Experimental details | | | | | | Weight | Type of error | Reference |
|---|---|---|---|---|---|---|---|---|---|---|
| | | Species | Expression Vector | Enzyme | pH | Temperature (°C) | Other | | | |
| -7.00 x10$^{1}$ | 2.27 x10$^{51}$ | Human | Unknown | 5-LOX | 7 | Unknown | | 64 | 0 | (Caspi et al., 2018) |

**Table ST.7.14.1.4.2.** The log-normal distribution properties of the 12-LOX $K_{eq}$ distribution. These values were calculated using the functions described in Section 2.6.2.

| Mode | Confidence Interval | Location parameter (μ) | Scale parameter (σ) |
|---|---|---|---|
| 2.27 x10$^{51}$ | 1.00 x10$^{1}$ | 1.19 x10$^{2}$ | 8.90 x10$^{-1}$ |

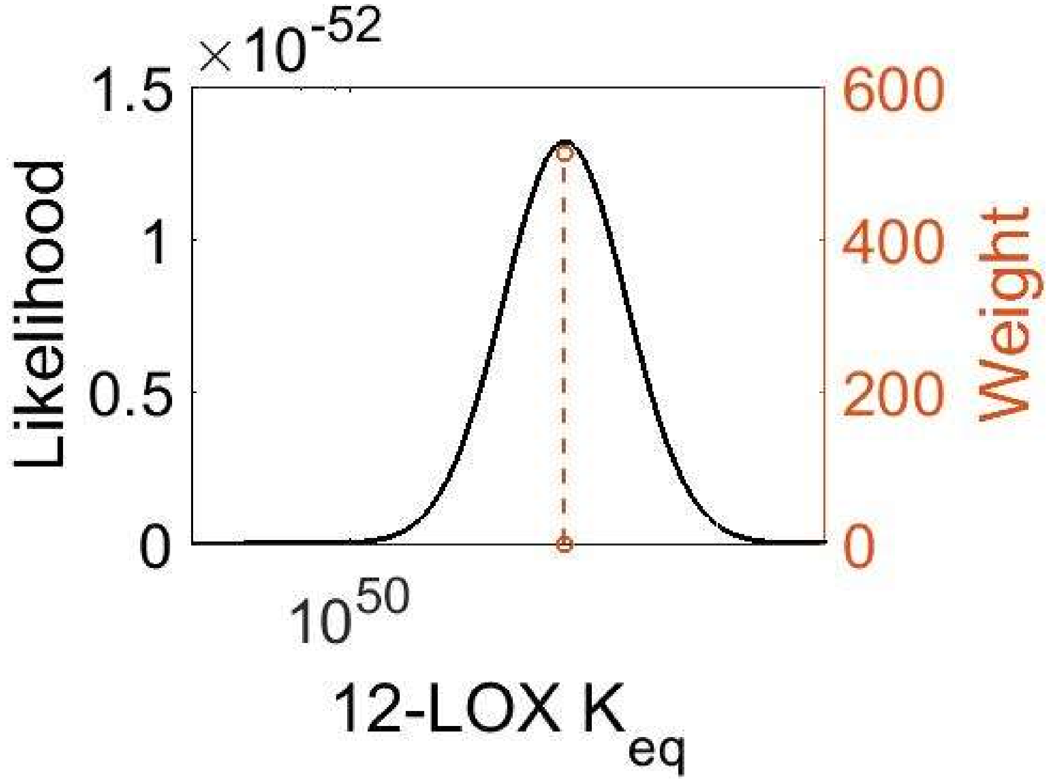


**Figure SF.7.14.1.4.1.** The estimated probability distribution for the 12-LOX $K_{eq}$. The value and weight of the literature values used to define the distribution are indicated by an orange dashed line. The x axis is plotted on a log-scale.

### S.7.14.1.5. Parameter: 12-LOX concentration

Parameter values for the 12-LOX concentration of Reaction 18 were obtained from the literature and summarised in Table ST.7.14.1.5.1. The log-normal distribution properties for the 12-LOX concentration of Reaction 18 are shown in Table ST.7.14.1.5.2 and plotted in Figure SF.7.14.1.5.1. To convert the enzyme concentration from ppm to mM, **Equation S.6.2** was used.

**Table ST.7.14.1.5.1.** Literature information used to design the 12-LOX concentration parameter distribution. Each value was assigned a weight using the protocol described in Section 2.6.2. The type of error was assigned as 0 if it was additive and 1 if it was multiplicative.

| Value (ppm) | Error (ppm) | Experimental details | | | | | | Weight | Type of error | Reference |
|---|---|---|---|---|---|---|---|---|---|---|
| | | Species | Expression Vector | Enzyme | pH | Temperature (°C) | Other | | | |
| 1.10 x$10^{-1}$ | NaN | Human | Pancreas | 12-LOX | 7.5 | 37 | | **1024** | 0 | (Kim et al., 2014) |
| 2.80 x$10^{-1}$ | NaN | Human | Gut | 12-LOX | 7.5 | 37 | | **1024** | 0 | (Kim et al., 2014) |
| 1.60 | NaN | Human | Liver | 12-LOX | 7.5 | 37 | | **1024** | 0 | (Kim et al., 2014) |
| 1.98 x$10^{1}$ | NaN | Human | Spleen | 12-LOX | 7.5 | 37 | | **1024** | 0 | (Wilhelm et al., 2014) |

**Table ST.7.14.1.5.2.** The log-normal distribution properties of the 12-LOX concentration distribution. These values were calculated using the functions described in Section 2.6.2.

| Mode (ppm) | Mode (mM) | Confidence Interval | Location parameter (μ) | Scale parameter (σ) |
|---|---|---|---|---|
| 4.98 x$10^{-1}$ | 2.76 x$10^{-6}$ | 7.23 | 7.12 x$10^{-1}$ | 1.19 |

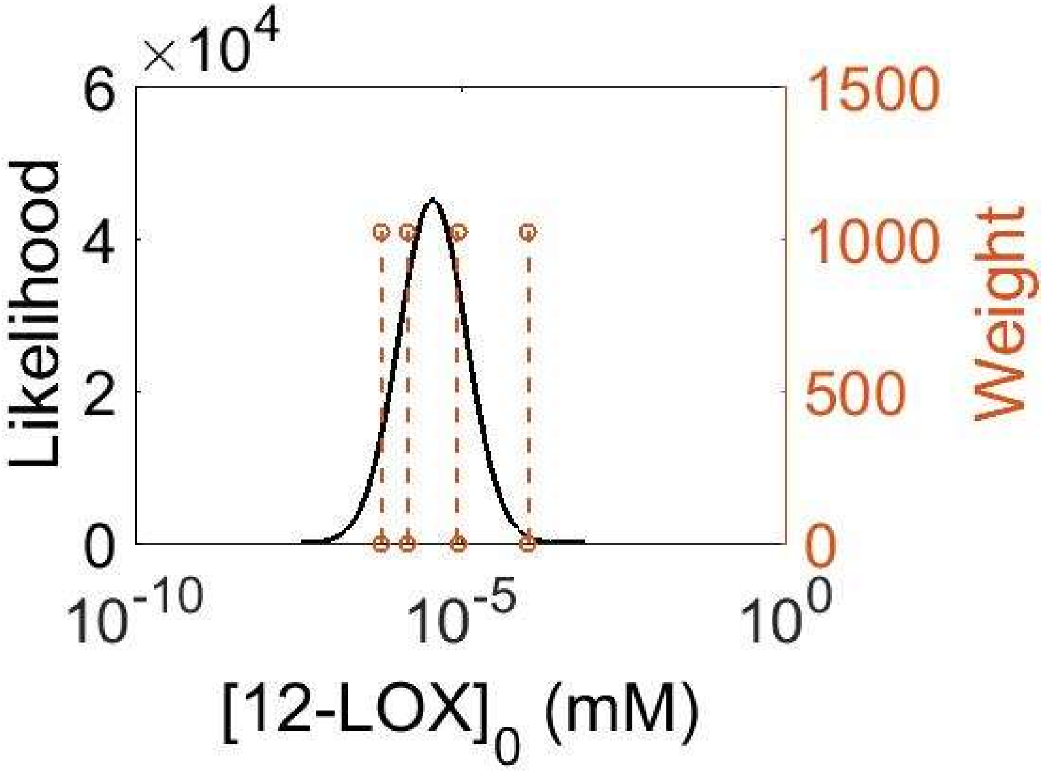


**Figure SF.7.14.1.5.1.** The estimated probability distribution for the 12-LOX concentration. The value and weight of the literature values used to define the distribution are indicated by an orange dashed line. The x axis is plotted on a log-scale.

**S.7.15 Reaction 20: 12-HPETE ⇌ 12-HETE**
Both enantiomers of 12-HPETE can be reduced by an oxidoreductase enzyme (PHGPx), to form 12(R)-HETE and 12(S)-HETE.

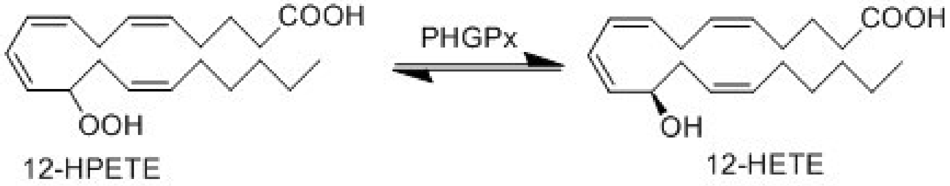


**Figure SF.7.15.** The metabolism of 12-hydroperoxyeicosatetraenoic acid (12-HPETE) to 12-hydroxyeicosatetraenoic acid (12-HETE) by phospholipid hydroperoxide glutathione peroxidase (PHGPx) (Reaction 20).

**SEq.7.15. Reaction rate law for Reaction 20.**

$$\boldsymbol{v_{20}} = \frac{K_{cat} \cdot [PHGPx]\left([12-HPETE] - {[12-HETE]}/{K_{eq}}\right)}{K_{m_s}\left(1 + \frac{[12-HPETE]}{K_{m_p}}\right) + [12-HETE]}$$

### S.7.15.1. Reaction parameters

#### S.7.15.1.1. Parameter: PHGPx $K_{ms}$

Parameter values for the $K_{ms}$ of Reaction 20 were obtained from the literature and summarised in Table ST.7.15.1.1.1. The log-normal distribution properties for the $K_{ms}$ of Reaction 20 are shown in Table ST.7.15.1.1.2 and plotted in Figure SF.7.15.1.1.1.

**Table ST.7.15.1.1.1.** Literature information used to design the PHGPx $K_{ms}$ parameter distribution. Each value was assigned a weight using the protocol described in Section 2.6.2. The type of error was assigned as 0 if it was additive and 1 if it was multiplicative.

| Value (mM) | Error (mM) | Experimental details | | | | | | Weight | Type of error | Reference |
|---|---|---|---|---|---|---|---|---|---|---|
| | | Species | Expression Vector | Enzyme | pH | Temperature (°C) | Other | | | |
| $4.00 \times 10^{-3}$ | NaN | Rat | Liver | PHGPx | 7.4 | 37 | | 768 | 0 | (Hiratsuka et al., 1997) |
| $3.00 \times 10^{-1}$ | NaN | Human | Bio imprinted Enzyme | PHGPx | 7 | 37 | | 512 | 0 | (Liu et al., 2008) |
| $1.11 \times 10^{1}$ | $2.90 \times 10^{-1}$ | Human | E. coli. | PHGPx | 7 | 37 | | 1024 | 0 | (Zheng et al., 2008) |

**Table ST.7.15.1.1.2.** The log-normal distribution properties of the PHGPx $K_{ms}$ distribution. These values were calculated using the functions described in Section 2.6.2.

| Mode (mM) | Confidence Interval | Location parameter (μ) | Scale parameter (σ) |
|---|---|---|---|
| $3.19 \times 10^{-1}$ | $1.02 \times 10^{3}$ | 2.54 | 1.92 |

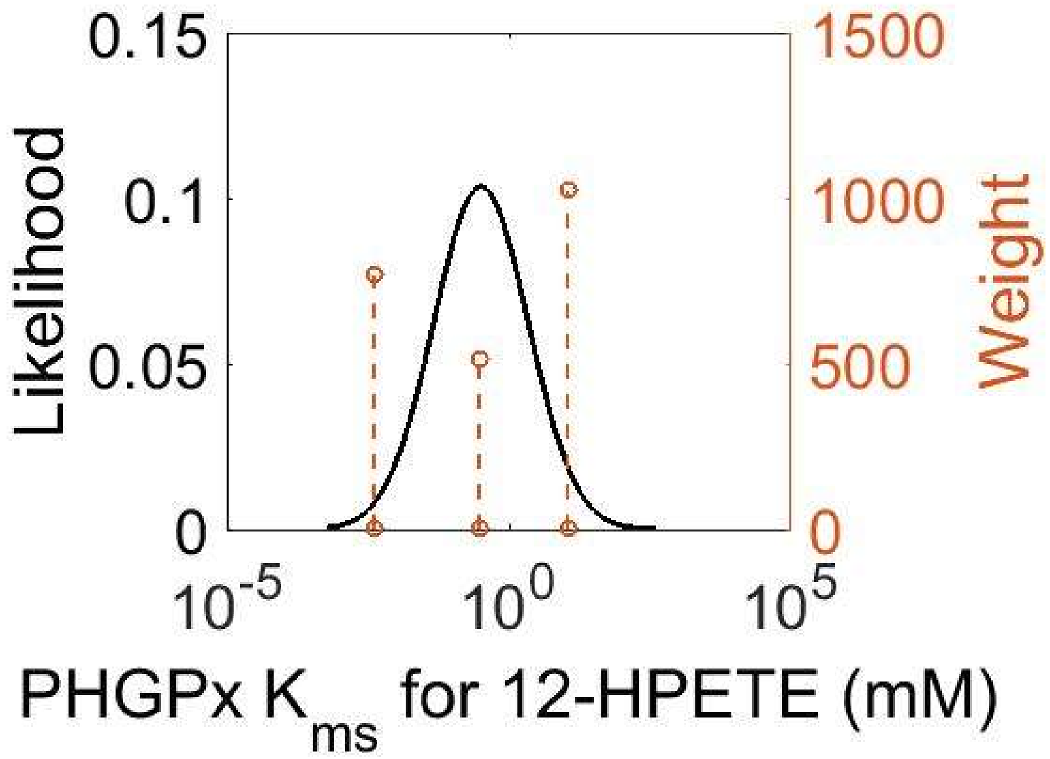


**Figure SF.7.15.1.1.1.** The estimated probability distribution for the PHGPx $K_{ms}$. The value and weight of the literature values used to define the distribution are indicated by an orange dashed line. The x axis is plotted on a log-scale.

**S.7.15.1.2. Parameter: PHGPx $K_{mp}$ (Dependent parameter)**

The log-normal distribution for the parameter for the $K_{mp}$ of Reaction 20 was calculated using multivariate distributions. The log-normal distribution properties for the $K_{mp}$ of Reaction 20 are shown in Table ST.7.15.1.2.1 and plotted in Figure SF.7.15.1.2.1.

**Table ST.7.15.1.2.1.** The log-normal distribution properties of the PHGPx $K_{mp}$ distribution. These values were calculated using the functions described in Section 2.6.2.

| Mode (mM) | Location parameter (μ) | Scale parameter (σ) |
|---|---|---|
| $3.15 \times 10^{-1}$ | 2.53 | 1.92 |

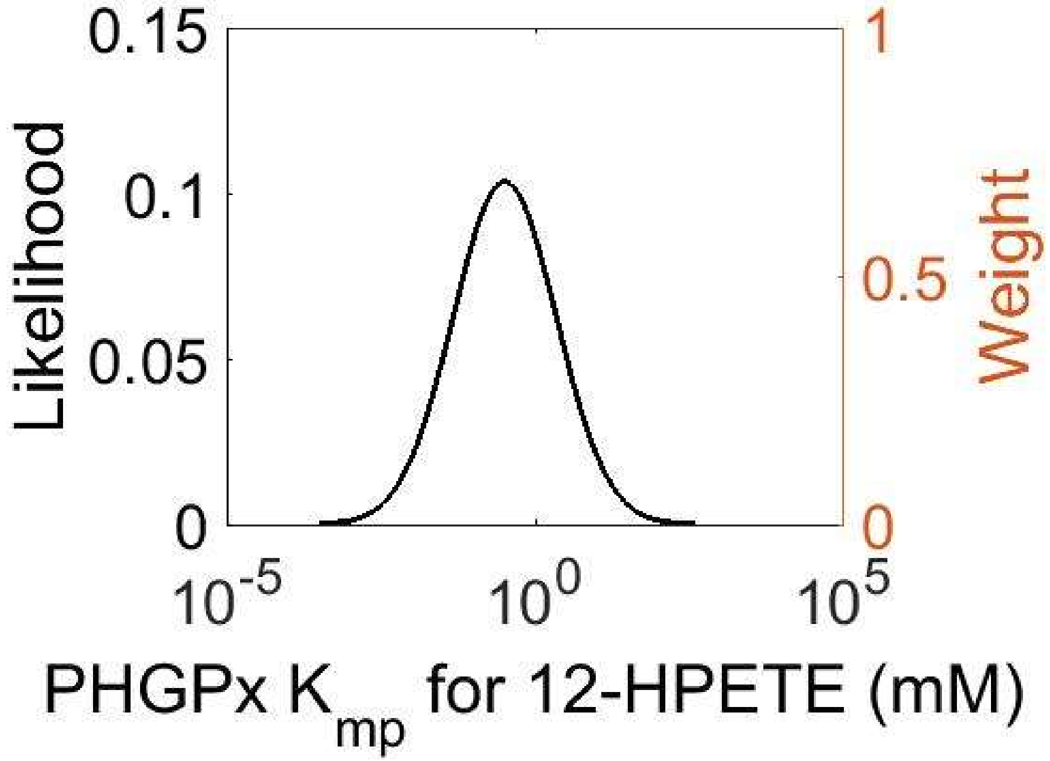


**Figure SF.7.15.1.2.1.** The estimated probability distribution for the PHGPx $K_{mp}$. The value and weight of the literature values used to define the distribution are indicated by an orange dashed line. The x axis is plotted on a log-scale.

### S.7.15.1.3. Parameter: PHGPx $k_{cat}$

Parameter values for the $k_{cat}$ of Reaction 20 were obtained from the literature and summarised in Table ST.7.15.1.3.1. The log-normal distribution properties for the $k_{cat}$ of Reaction 20 are shown in Table ST.7.15.1.3.2 and plotted in Figure SF.7.15.1.3.1.

**Table ST.7.15.1.3.1.** Literature information used to design the PHGPx $k_{cat}$ parameter distribution. Each value was assigned a weight using the protocol described in Section 2.6.2. The type of error was assigned as 0 if it was additive and 1 if it was multiplicative.

| Value ($min^{-1}$) | Error ($min^{-1}$) | Experimental details | | | | | | Weight | Type of error | Reference |
|---|---|---|---|---|---|---|---|---|---|---|
| | | Species | Expression Vector | Enzyme | pH | Temperature (°C) | Other | | | |
| 1.17 $x10^3$ | 5.00 $x10^1$ | Human | Bio imprinted Enzyme | PHGPx | 7 | 37 | | 512 | 0 | (Liu et al., 2008) |
| 2.45 $x10^4$ | 1.50 $x10^2$ | Human | E. coli. | PHGPx | 7 | 37 | | 1024 | 0 | (Zheng et al., 2008) |

**Table ST.7.15.1.3.2.** The log-normal distribution properties of the PHGPx $k_{cat}$ distribution. These values were calculated using the functions described in Section 2.6.2.

| Mode ($min^{-1}$) | Confidence Interval | Location parameter (μ) | Scale parameter (σ) |
|---|---|---|---|
| 2.44 $x10^4$ | 4.20 | 1.11 $x10^1$ | 9.75 $x10^{-1}$ |

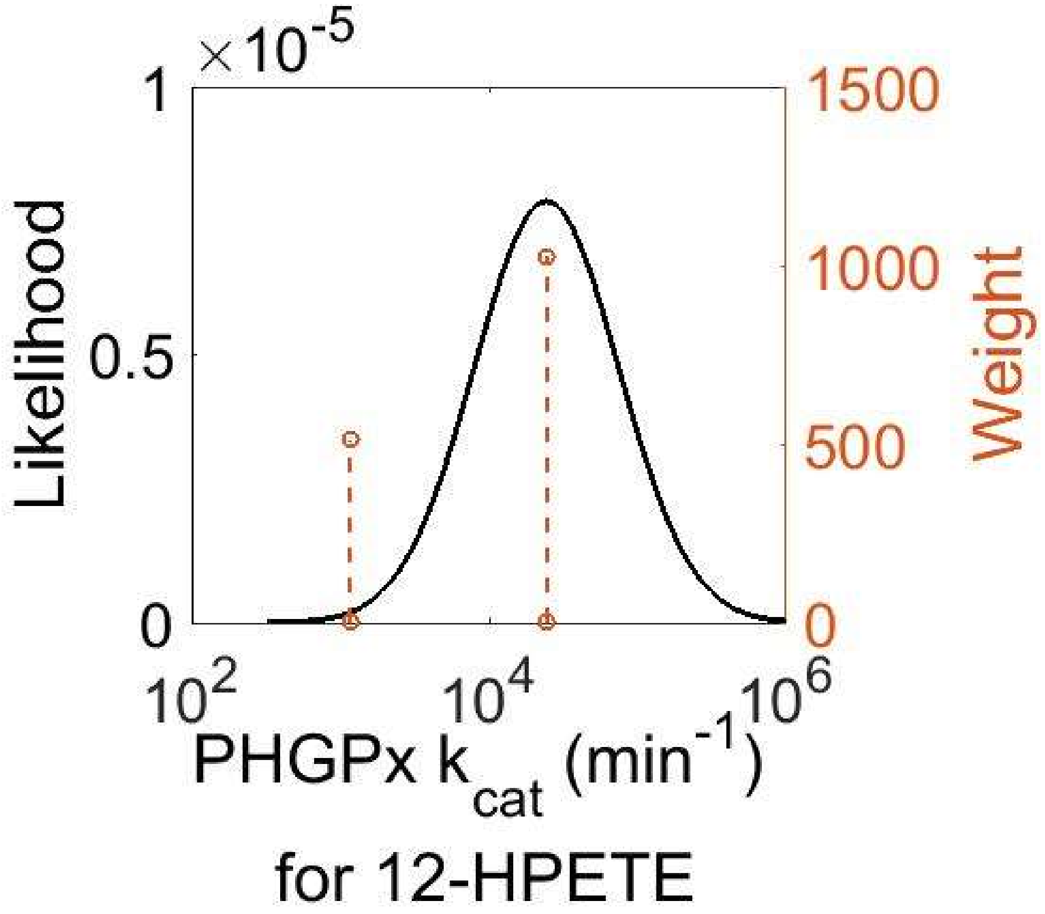


**Figure SF.7.15.1.3.1.** The estimated probability distribution for the PHGPx $k_{cat}$. The value and weight of the literature values used to define the distribution are indicated by an orange dashed line. The x axis is plotted on a log-scale.

### S.7.15.1.4. Parameter: PHGPx $K_{eq}$

Parameter values for the $K_{eq}$ of Reaction 20 were obtained from the literature and summarised in Table ST.7.15.1.4.1. The log-normal distribution properties for the $K_{eq}$ of Reaction 20 are shown in Table ST.7.15.1.4.2 and plotted in Figure SF.7.15.1.4.1.

**Table ST.7.15.1.4.1.** Literature information used to design the PHGPx $K_{eq}$ parameter distribution. Each value was assigned a weight using the protocol described in Section 2.6.2. The type of error was assigned as 0 if it was additive and 1 if it was multiplicative.

| ΔG (kcal/mol) | $K_{eq}$ | Experimental details | | | | | | Weight | Type of error | Reference |
|---|---|---|---|---|---|---|---|---|---|---|
| | | Species | Expression Vector | Enzyme | pH | Temperature (°C) | Other | | | |
| $-2.69 \times 10^{1}$ | $5.90 \times 10^{19}$ | Human | Unknown | PHGPx | 7 | Unknown | | 64 | 0 | (Caspi et al., 2018) |

**Table ST.7.15.1.4.2.** The log-normal distribution properties of the PHGPx $K_{eq}$ distribution. These values were calculated using the functions described in Section 2.6.2.

| Mode | Confidence Interval | Location parameter (μ) | Scale parameter (σ) |
|---|---|---|---|
| $5.90 \times 10^{19}$ | $1.00 \times 10^{1}$ | $4.63 \times 10^{1}$ | $8.90 \times 10^{-1}$ |

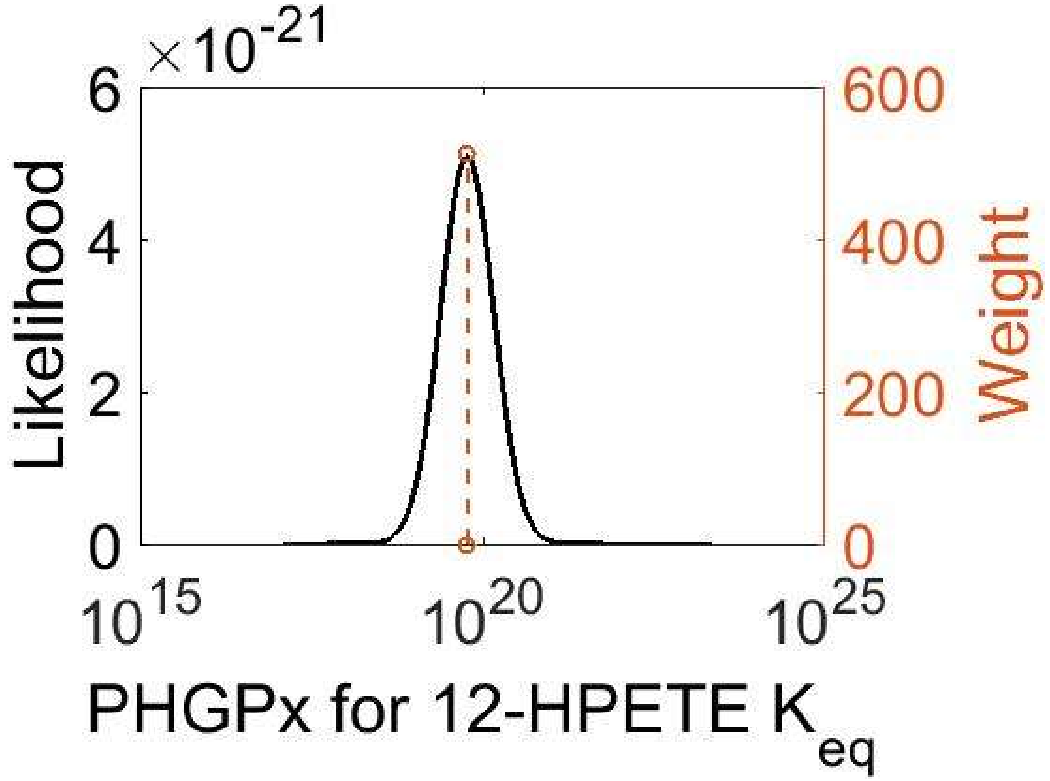


**Figure SF.7.15.1.4.1.** The estimated probability distribution for the PHGPx $K_{eq}$. The value and weight of the literature values used to define the distribution are indicated by an orange dashed line. The x axis is plotted on a log-scale.

### S.7.15.1.5. Parameter: PHGPx concentration

Parameter values for the PHGPx concentration of Reaction 20 were obtained from the literature and summarised in Table ST.7.15.1.5.1. The log-normal distribution properties for the PHGPx concentration of Reaction 20 are shown in Table ST.7.15.1.5.2 and plotted in Figure SF.7.15.1.5.1. To convert the enzyme concentration from ppm to mM, **Equation S.6.2** was used.

**Table ST.7.15.1.5.1.** Literature information used to design the PHGPx concentration parameter distribution. Each value was assigned a weight using the protocol described in Section 2.6.2. The type of error was assigned as 0 if it was additive and 1 if it was multiplicative.

| Value (mM) | Error (mM) | Experimental details | | | | | | Weight | Type of error | Reference |
|---|---|---|---|---|---|---|---|---|---|---|
| | | Species | Expression Vector | Enzyme | pH | Temperature (°C) | Other | | | |
| 2.04 $\times 10^{2}$ | NaN | Human | Gut | PHGPx | 7.5 | 37 | | **1024** | 0 | (Kim et al., 2014) |
| 2.82 $\times 10^{2}$ | NaN | Human | Oesophagus | PHGPx | 7.5 | 37 | | **1024** | 0 | (Wilhelm et al., 2014) |
| 3.07 $\times 10^{2}$ | NaN | Human | Lung | PHGPx | 7.5 | 37 | | **1024** | 0 | (Wilhelm et al., 2014) |
| 4.59 $\times 10^{2}$ | NaN | Human | Skin | PHGPx | 7.5 | 37 | | **2048** | 0 | (Wilhelm et al., 2014) |

**Table ST.7.15.1.5.2.** The log-normal distribution properties of the PHGPx concentration distribution. These values were calculated using the functions described in Section 2.6.2.

| Mode (ppm) | Mode (mM) | Confidence Interval | Location parameter (μ) | Scale parameter (σ) |
|---|---|---|---|---|
| 3.14 $\times 10^{2}$ | 1.74 $\times 10^{-3}$ | 1.38 | 5.85 | 3.09 $\times 10^{-1}$ |

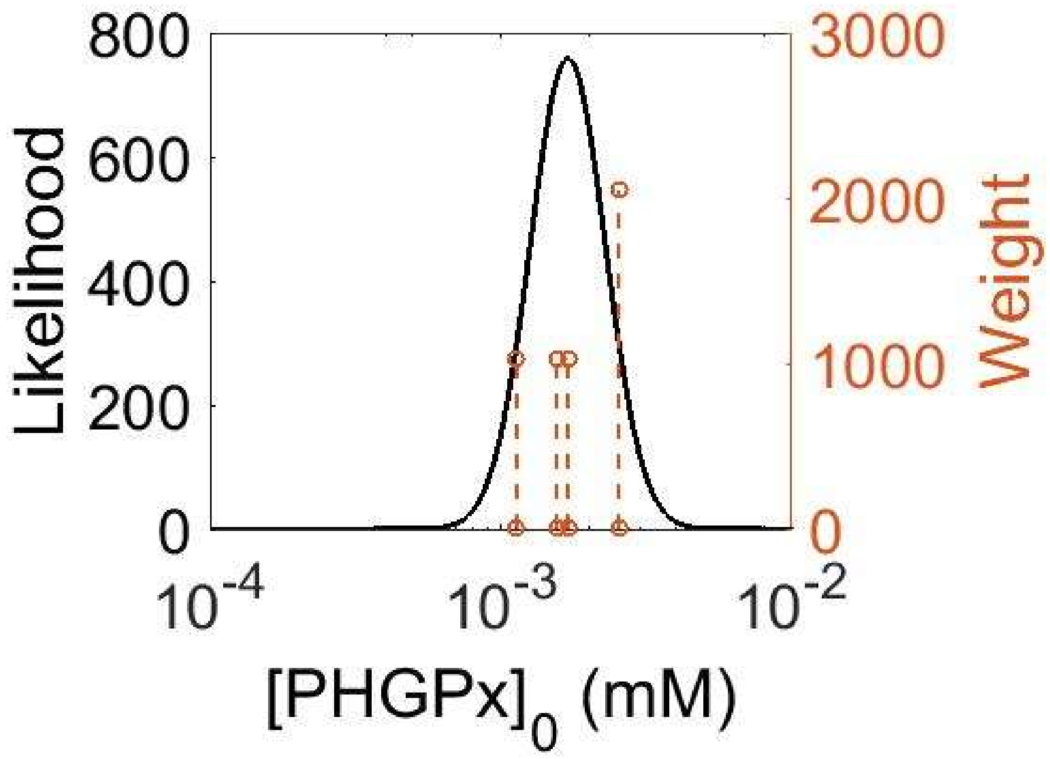


**Figure SF.7.15.1.5.1.** The estimated probability distribution for the PHGPx concentration. The value and weight of the literature values used to define the distribution are indicated by an orange dashed line. The x axis is plotted on a log-scale.

**S.7.16 Reaction 21: $PGH_2 \rightleftharpoons PGD_2$**

The isomerisation of $PGH_2$ to $PGD_2$ is performed by prostaglandin D synthase (PGDS), to yield a hydroxyl group at C9 and a ketone group at C11.

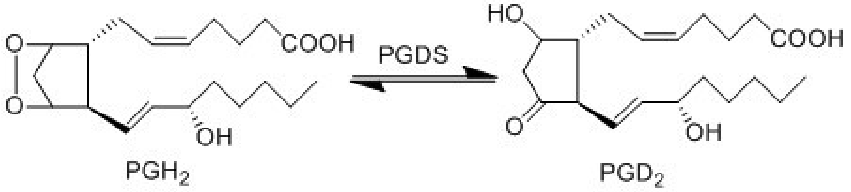


**Figure SF.7.16.** The metabolism of prostaglandin $H_2$ ($PGH_2$) into prostaglandin $D_2$ ($PGD_2$) by prostaglandin D synthase (PGDS) (Reaction 21).

**SEq.7.16.** Reaction rate law for Reaction 21.

$$\boldsymbol{v_{21}} = \frac{k_{cat} \cdot [PGDS]\left([PGH_2] - {[PGD_2]}/{K_{eq}}\right)}{K_{m_s}\left(1 + \frac{[PGD_2]}{K_{m_p}}\right) + [PGH_2]}$$

### S.7.16.1. Reaction parameters

#### S.7.16.1.1. Parameter: PGDS $K_{ms}$

Parameter values for the $K_{ms}$ of Reaction 21 were obtained from the literature and summarised in Table ST.7.16.1.1.1. The log-normal distribution properties for the $K_{ms}$ of Reaction 21 are shown in Table ST.7.16.1.1.2 and plotted in Figure SF.7.16.1.1.1.

**Table ST.7.16.1.1.1.** Literature information used to design the PGDS $K_{ms}$ parameter distribution. Each value was assigned a weight using the protocol described in Section 2.6.2. The type of error was assigned as 0 if it was additive and 1 if it was multiplicative.

| Value (mM) | Error (mM) | Experimental details | | | | | | Weight | Type of error | Reference |
|---|---|---|---|---|---|---|---|---|---|---|
| | | Species | Expression Vector | Enzyme | pH | Temperature (°C) | Other | | | |
| 4.00 x10$^{-3}$ | NaN | Human | Cerebrospinal Fluid | PGDS | 10 | Unknown | | 256 | 0 | (Watanabe et al., 1994) |
| 1.38 x10$^{-2}$ | NaN | Human | Human cell | PGDS | 8 | 25 | | 512 | 0 | (Zhou et al., 2010) |
| 5.00 x10$^{-1}$ | NaN | Human | E. coli. | PGDS | 6.5 | Unknown | | 128 | 0 | (Pinzar et al., 2000) |
| 1.40 x10$^{-2}$ | NaN | Rat | Cerebrospinal Fluid | PGDS | 7 | 25 | | 256 | 0 | (Urade et al., 1985) |

**Table ST.7.16.1.1.2.** The log-normal distribution properties of the PGDS $K_{ms}$ distribution. These values were calculated using the functions described in Section 2.6.2.

| Mode (mM) | Confidence Interval | Location parameter (μ) | Scale parameter (σ) |
|---|---|---|---|
| 1.35 x10$^{-2}$ | 3.78 | -3.44 | 9.29 x10$^{-1}$ |

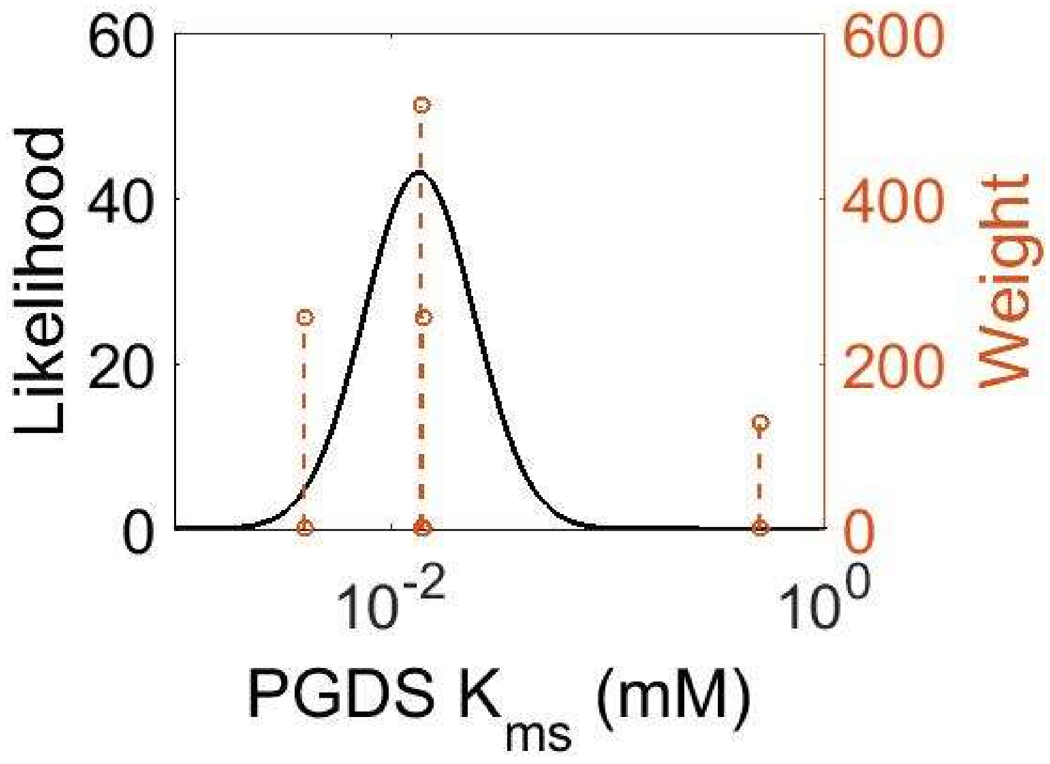


**Figure SF.7.16.1.1.1.** The estimated probability distribution for the PGDS $K_{ms}$. The value and weight of the literature values used to define the distribution are indicated by an orange dashed line. The x axis is plotted on a log-scale.

### S.7.16.1.2. Parameter: PGDS $K_{mp}$ (Dependent parameter)

The log-normal distribution for the parameter for the $K_{mp}$ of Reaction 21 was calculated using multivariate distributions. The log-normal distribution properties for the $K_{mp}$ of Reaction 21 are shown in Table ST.7.16.1.2.1 and plotted in Figure SF.7.16.1.2.1.

**Table ST.7.16.1.2.1. The log-normal distribution properties of the PGDS $K_{mp}$ distribution. These values were calculated using the functions described in Section 2.6.2.**

| Mode (mM) | Location parameter (μ) | Scale parameter (σ) |
| --- | --- | --- |
| 1.30 $x10^{-2}$ | -4.02 | 5.69 $x10^{-1}$ |

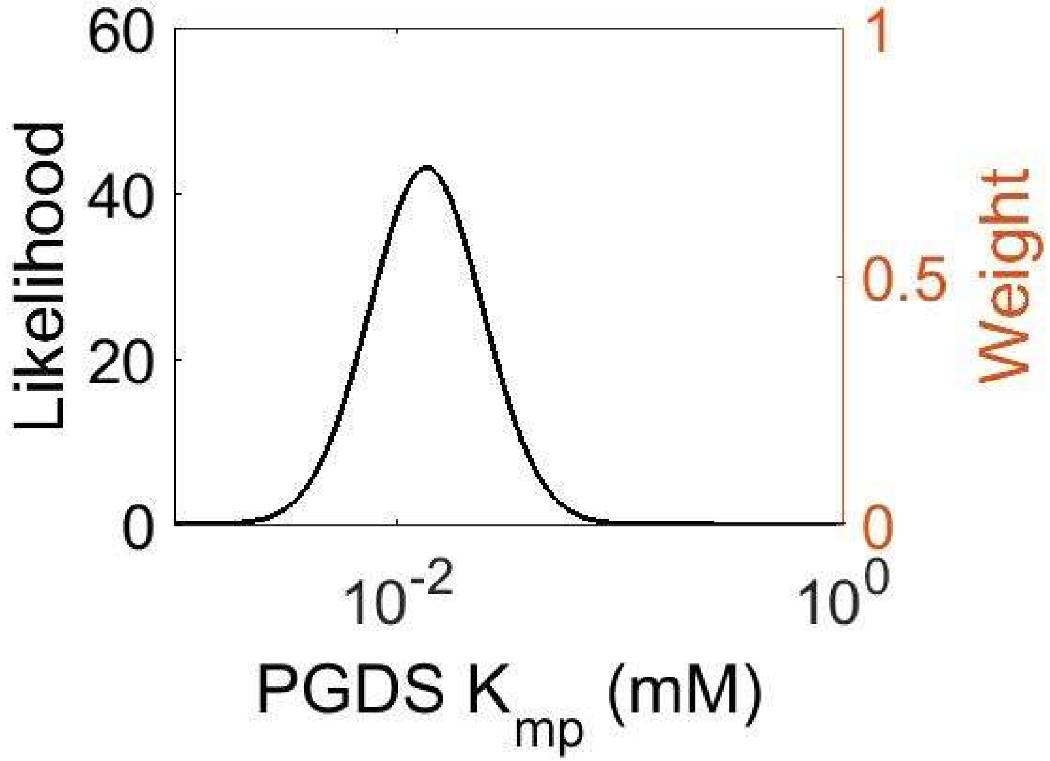


**Figure SF.7.16.1.2.1.** The estimated probability distribution for the PGDS $K_{mp}$. The value and weight of the literature values used to define the distribution are indicated by an orange dashed line. The x axis is plotted on a log-scale.

### S.7.16.1.3. Parameter: PGDS $k_{cat}$

Parameter values for the $k_{cat}$ of Reaction 21 were obtained from the literature and summarised in Table ST.7.16.1.3.1. The log-normal distribution properties for the $k_{cat}$ of Reaction 21 are shown in Table ST.7.16.1.3.2 and plotted in Figure SF.7.16.1.3.1.

**Table ST.7.16.1.3.1.** Literature information used to design the PGDS $k_{cat}$ parameter distribution. Each value was assigned a weight using the protocol described in Section 2.6.2. The type of error was assigned as 0 if it was additive and 1 if it was multiplicative.

| Value ($min^{-1}$) | Error ($min^{-1}$) | Experimental details | | | | | | Weight | Type of error | Reference |
|---|---|---|---|---|---|---|---|---|---|---|
| | | Species | Expression Vector | Enzyme | pH | Temperature (°C) | Other | | | |
| 1.58 $x10^2$ | NaN | Human | Human cell | PGDS | 8 | 25 | | 256 | 0 | (Zhou et al., 2010) |
| 1.30 $x10^3$ | | Human | E. coli. | PGDS | 6.5 | Unknown | | 128 | | (Pinzar et al., 2000) |

**Table ST.7.16.1.3.2.** The log-normal distribution properties of the PGDS $k_{cat}$ distribution. These values were calculated using the functions described in Section 2.6.2.

| Mode ($min^{-1}$) | Confidence Interval | Location parameter (μ) | Scale parameter (σ) |
|---|---|---|---|
| 1.58 $x10^2$ | 1.50 | 5.10 | 2.00 $x10^{-1}$ |

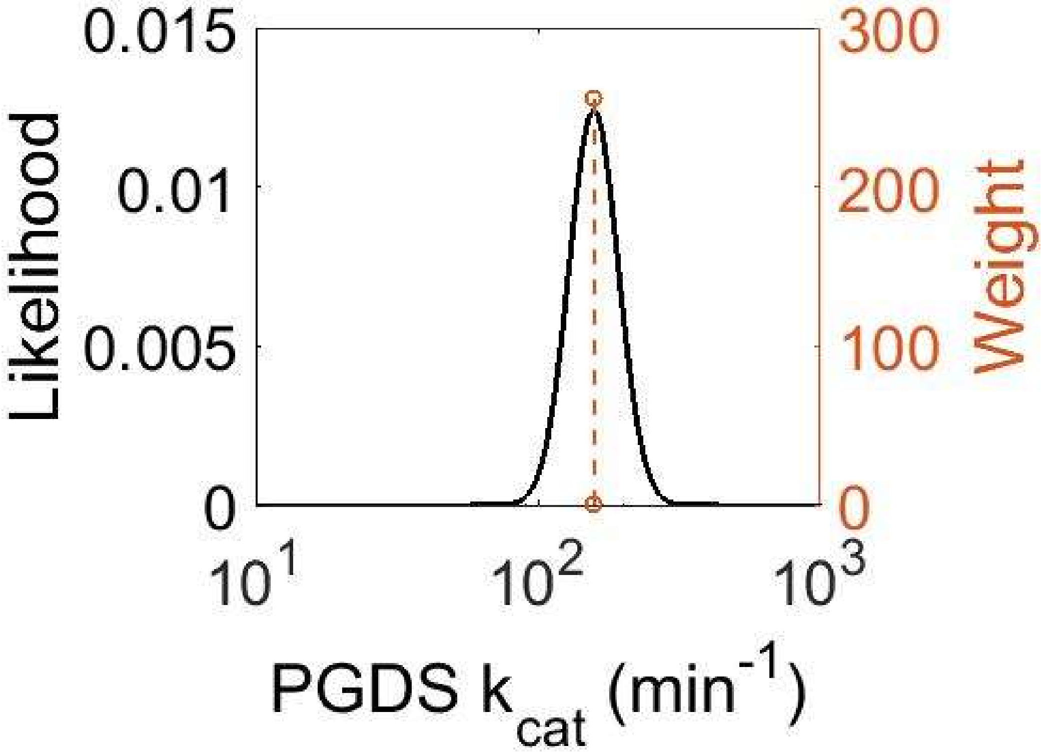


**Figure SF.7.16.1.3.1.** The estimated probability distribution for the PGDS $k_{cat}$. The value and weight of the literature values used to define the distribution are indicated by an orange dashed line. The x axis is plotted on a log-scale.

### S.7.16.1.4. Parameter: PGDS $K_{eq}$

Parameter values for the $K_{eq}$ of Reaction 21 were obtained from the literature and summarised in Table ST.7.16.1.4.1. The log-normal distribution properties for the $K_{eq}$ of Reaction 21 are shown in Table ST.7.16.1.4.2 and plotted in Figure SF.7.16.1.4.1.

**Table ST.7.16.1.4.1.** Literature information used to design the PGDS $K_{eq}$ parameter distribution. Each value was assigned a weight using the protocol described in Section 2.6.2. The type of error was assigned as 0 if it was additive and 1 if it was multiplicative.

| ΔG (kcal/mol) | $K_{eq}$ | Experimental details | | | | | | Weight | Type of error | Reference |
|---|---|---|---|---|---|---|---|---|---|---|
| | | Species | Expression Vector | Enzyme | pH | Temperature (°C) | Other | | | |
| -6.64 | 7.46 x10$^{4}$ | Human | Unknown | PGDS | 7 | Unknown | | 64 | 0 | (Caspi et al., 2018) |

**Table ST.7.16.1.4.2.** The log-normal distribution properties of the PGDS $K_{eq}$ distribution. These values were calculated using the functions described in Section 2.6.2.

| Mode | Confidence Interval | Location parameter (μ) | Scale parameter (σ) |
|---|---|---|---|
| 7.46 x10$^{4}$ | 1.00 x10$^{1}$ | 1.20 x10$^{1}$ | 8.90 x10$^{-1}$ |

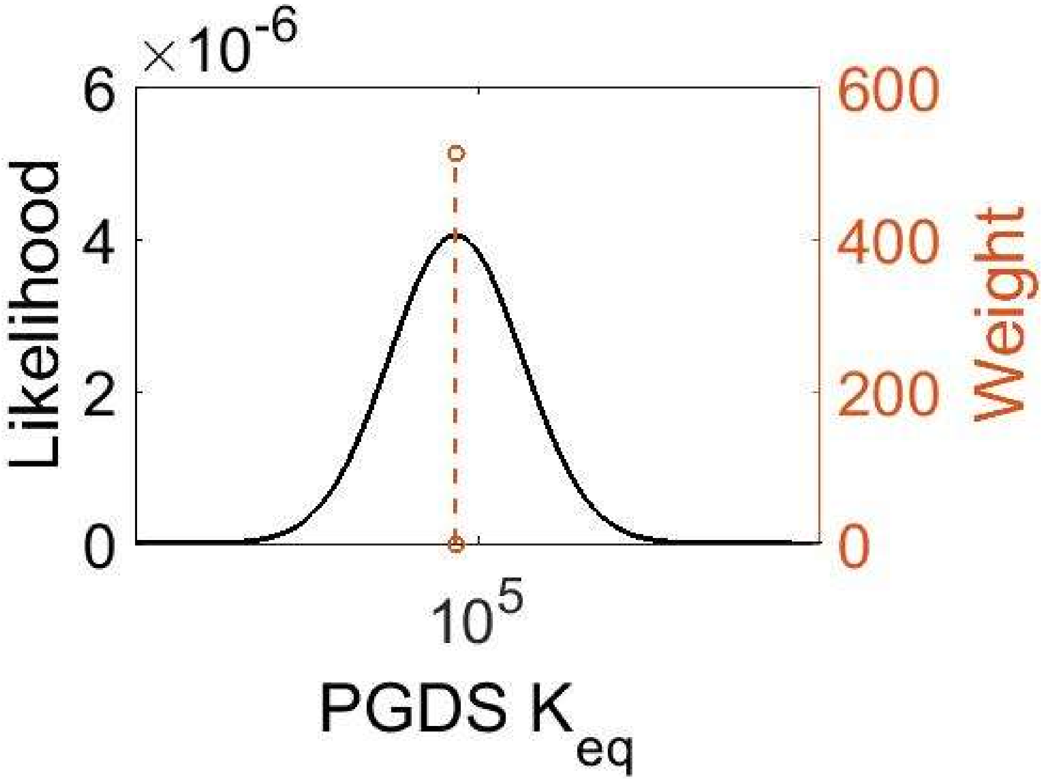


**Figure SF.7.16.1.4.1.** The estimated probability distribution for the PGDS $K_{eq}$. The value and weight of the literature values used to define the distribution are indicated by an orange dashed line. The x axis is plotted on a log-scale.

### S.7.16.1.5. Parameter: PGDS concentration

Parameter values for the PGDS concentration of Reaction 21 were obtained from the literature and summarised in Table ST.7.16.1.5.1. The log-normal distribution properties for the PGDS concentration of Reaction 21 are shown in Table ST.7.16.1.5.2 and plotted in Figure SF.7.16.1.5.1. To convert the enzyme concentration from ppm to mM, **Equation S.6.2** was used.

**Table ST.7.16.1.5.1.** Literature information used to design the PGDS concentration parameter distribution. Each value was assigned a weight using the protocol described in Section 2.6.2. The type of error was assigned as 0 if it was additive and 1 if it was multiplicative.

| Value (mM) | Error (ppm) | Experimental details | | | | | | Weight | Type of error | Reference |
|---|---|---|---|---|---|---|---|---|---|---|
| | | Species | Expression Vector | Enzyme | pH | Temperature (°C) | Other | | | |
| $4.45 \times 10^{1}$ | NaN | Human | Skin | PGDS | 7.5 | 37 | | **2048** | 0 | (Wilhelm et al., 2014) |
| $6.79 \times 10^{1}$ | NaN | Human | Oesophagus | PGDS | 7.5 | 37 | | **1024** | 0 | (Kim et al., 2014) |
| $1.01 \times 10^{2}$ | NaN | Human | Oral cavity | PGDS | 7.5 | 37 | | **1024** | 0 | (Wilhelm et al., 2014) |
| $1.56 \times 10^{2}$ | NaN | Human | Pancreas | PGDS | 7.5 | 37 | | **1024** | 0 | (Kim et al., 2014) |

**Table ST.7.16.1.5.2.** The log-normal distribution properties of the PGDS concentration distribution. These values were calculated using the functions described in Section 2.6.2.

| Mode (ppm) | Mode (mM) | Confidence Interval | Location parameter (μ) | Scale parameter (σ) |
|---|---|---|---|---|
| $6.76 \times 10^{1}$ | $3.74 \times 10^{-4}$ | 1.64 | 4.41 | $4.49 \times 10^{-1}$ |

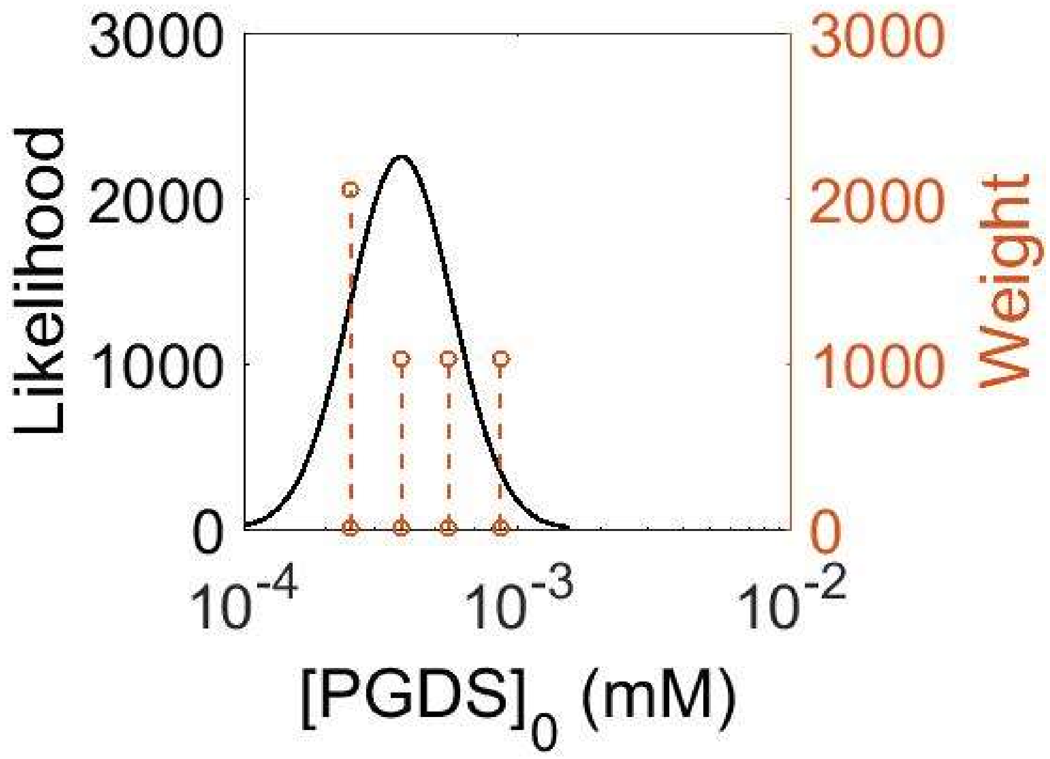


**Figure SF.7.16.1.5.1.** The estimated probability distribution for the PGDS concentration. The value and weight of the literature values used to define the distribution are indicated by an orange dashed line. The x axis is plotted on a log-scale.

### S.7.17 Reaction 65: AA = $PGH_2$

COX-1, the constitutively expressed isoform of COX, mediates the production of PGH2 from AA. This is a two-step reaction of cycloooxygenation and oxygenation, followed by a hydroperoxide reduction. The cyclooxygenase reaction occurs in the hydrophobic channel within the core of the protein and generates $PGG_2$. The subsequent peroxidase reaction produces $PGH_2$ and occurs at the heme-containing active site near the protein surface. The two step reaction results in the insertion of molecular oxygen across the C-9 and C-11 double bonds.

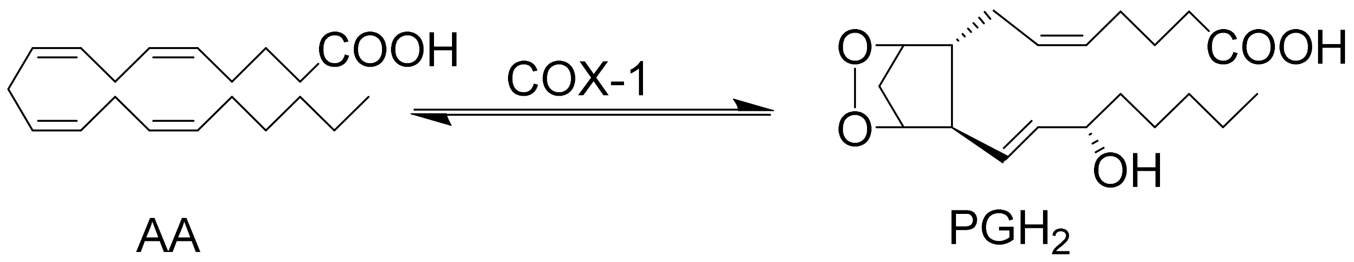


**Figure SF.7.17.** The cycloooxygenation and oxygenation reaction, followed by a hydroperoxide reduction of arachidonic acid (AA) into prostaglandin $H_2$ ($PGH_2$) by cyclooxygenase 1 (COX-1) (Reaction 65).

**SEq.7.17.** Reaction rate law for Reaction 65.

$$v_{65} = \frac{K_{cat} \cdot [COX-1]\left([AA] - {[PGH_2]}/{K_{eq}}\right)}{K_{m_S}\left(1 + \frac{[PGH_2]}{K_{m_P}}\right) + [AA]}$$

### S.7.17.1. Reaction parameters

#### S.7.17.1.1. Parameter: COX-1 $K_{ms}$

Parameter values for the $K_{ms}$ of Reaction 65 were obtained from the literature and summarised in Table ST.7.17.1.1.1. The log-normal distribution properties for the $K_{ms}$ of Reaction 65 are shown in Table ST.7.17.1.1.2 and plotted in Figure SF.7.17.1.1.1.

**Table ST.7.17.1.1.1.** Literature information used to design the COX-1 $K_{ms}$ parameter distribution. Each value was assigned a weight using the protocol described in Section 2.6.2. The type of error was assigned as 0 if it was additive and 1 if it was multiplicative.

| Value (mM) | Error (mM) | Experimental details | | | | | | Weight | Type of error | Reference |
|---|---|---|---|---|---|---|---|---|---|---|
| | | Species | Expression Vector | Enzyme | pH | Temperature (°C) | Other | | | |
| 8.80 $\times10^{-3}$ | 2.20 $\times10^{-3}$ | Human | Human cell | COX-1 | 7.6 | 37 | | 2048 | 0 | (Noreen et al., 1998) |
| 1.90 $\times10^{-3}$ | 2.00 $\times10^{-4}$ | Ram | Ram cell | COX-1 | 8 | 30 | | 192 | 0 | (Mukherjee et al., 2007) |

**Table ST.7.17.1.1.2.** The log-normal distribution properties of the COX-1 $K_{ms}$ distribution. These values were calculated using the functions described in Section 2.6.2.

| Mode (mM) | Confidence Interval | Location parameter (μ) | Scale parameter (σ) |
|---|---|---|---|
| 8.30 $\times10^{-3}$ | 1.62 | -4.60 | 4.41 $\times10^{-1}$ |

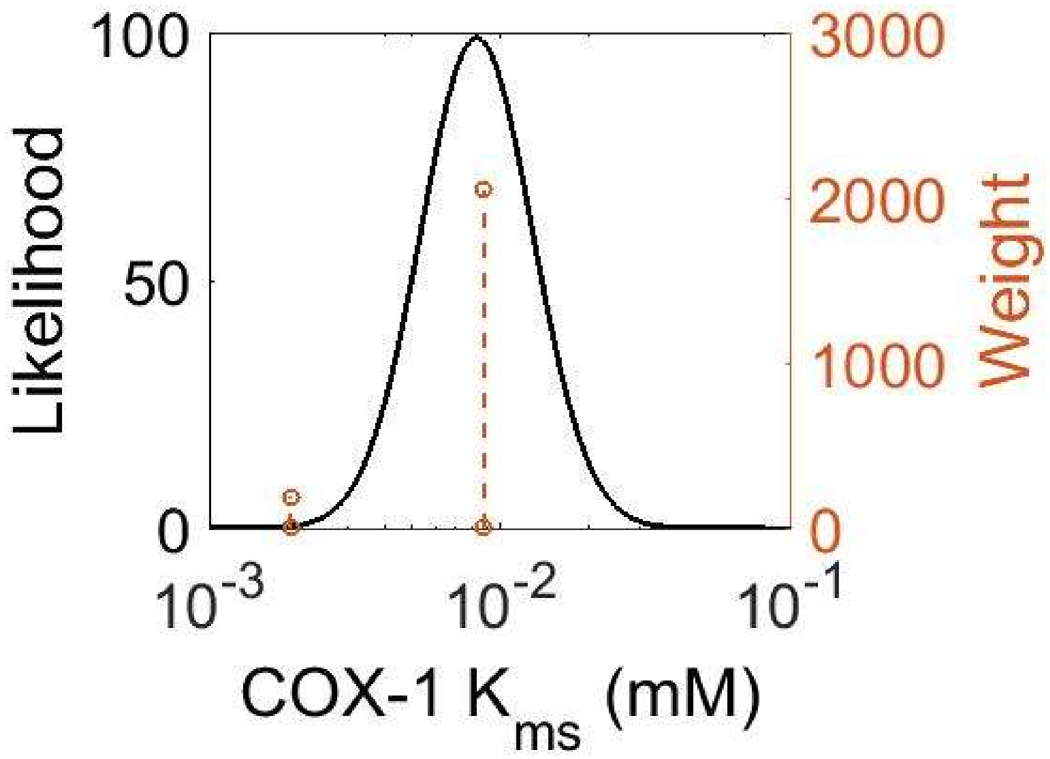


**Figure SF.7.17.1.1.1.** The estimated probability distribution for COX-1 $K_{ms}$. The value and weight of the literature values used to define the distribution are indicated by an orange dashed line. The x axis is plotted on a log-scale.

### S.7.17.1.2. Parameter: COX-1 $K_{mp}$

The log-normal distribution for the parameter for the $K_{mp}$ of Reaction 65 was calculated using multivariate distributions. The log-normal distribution properties for the $K_{mp}$ of Reaction 65 are shown in Table ST.7.17.1.2.1 and plotted in Figure SF.7.17.1.2.1.

**Table ST.7.17.1.2.1.** The log-normal distribution properties of the COX-1 $K_{mp}$ distribution. These values were calculated using the functions described in Section 2.6.2.

| Mode (mM) | Location parameter (μ) | Scale parameter (σ) |
|---|---|---|
| $8.20 \times 10^{-3}$ | -4.60 | $4.49 \times 10^{-1}$ |

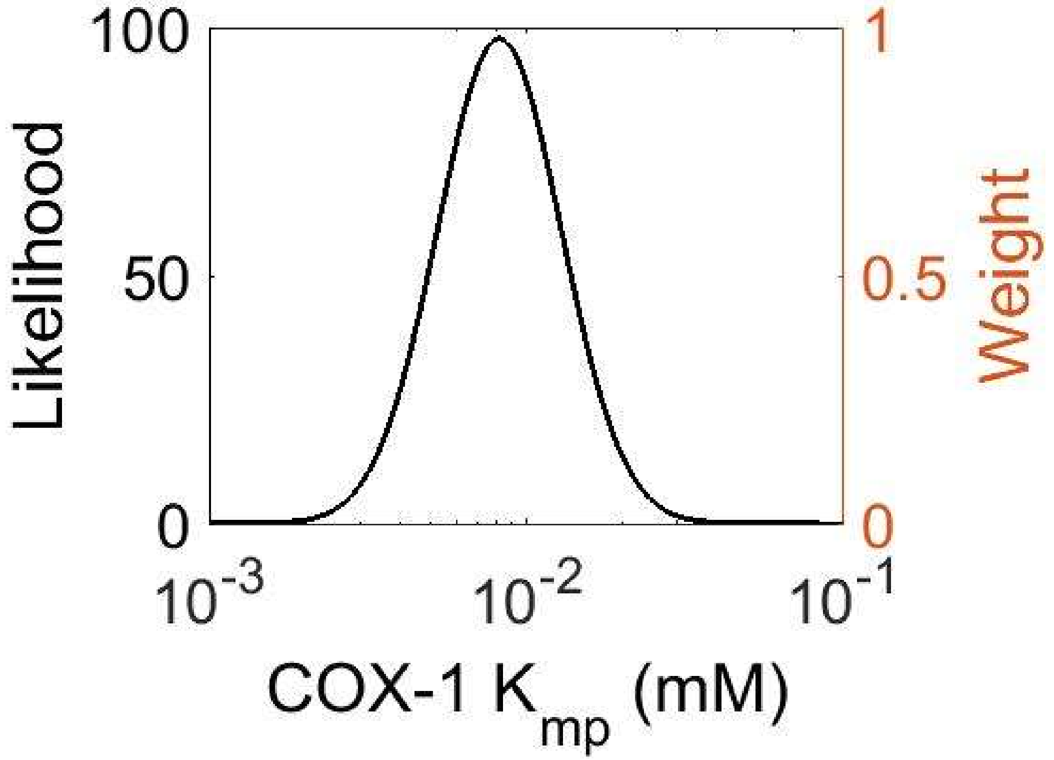


**Figure SF.7.17.1.2.1.** The estimated probability distribution for COX-1 $K_{mp}$. The value and weight of the literature values used to define the distribution are indicated by an orange dashed line. The x axis is plotted on a log-scale.

### S.7.17.1.3. Parameter: COX-1 $k_{cat}$

Parameter values for the $k_{cat}$ of Reaction 65 were obtained from the literature and summarised in Table ST.7.17.1.3.1. The log-normal distribution properties for the $k_{cat}$ of Reaction 65 are shown in Table ST.7.17.1.3.2 and plotted in Figure SF.7.17.1.3.1.

**Table ST.7.17.1.3.1.** Literature information used to design the COX-1 $k_{cat}$ parameter distribution. Each value was assigned a weight using the protocol described in Section 2.6.2. The type of error was assigned as 0 if it was additive and 1 if it was multiplicative.

| Value ($min^{-1}$) | Error ($min^{-1}$) | Experimental details | | | | | | Weight | Type of error | Reference |
|---|---|---|---|---|---|---|---|---|---|---|
| | | Species | Expression Vector | Enzyme | pH | Temperature (°C) | Other | | | |
| 8.82 $x10^3$ | 3.60 $x10^2$ | Ram | Ram cell | COX-1 | 8 | 30 | | 192 | 0 | (Mukherjee et al., 2007) |

**Table ST.7.17.1.3.2.** The log-normal distribution properties of the COX-1 $K_{ms}$ distribution. These values were calculated using the functions described in Section 2.6.2.

| Mode ($min^{-1}$) | Confidence Interval | Location parameter (μ) | Scale parameter (σ) |
|---|---|---|---|
| 8.81 $x10^3$ | 1.04 | 9.09 | 4.08 $x10^{-2}$ |

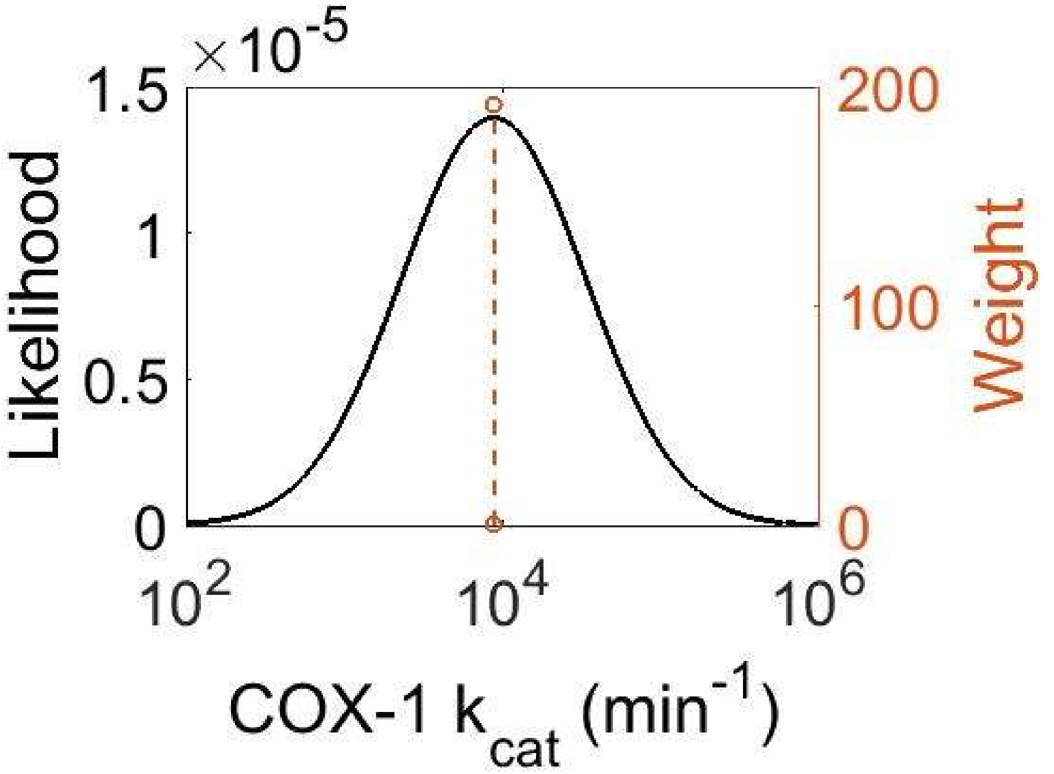


**Figure SF.7.17.1.3.1.** The estimated probability distribution for COX-1 $k_{cat}$. The value and weight of the literature values used to define the distribution are indicated by an orange dashed line. The x axis is plotted on a log-scale.

### S.7.17.1.4. Parameter: COX-1 Keq

Parameter values for the $K_{eq}$ of Reaction 65 were obtained from the literature and summarised in Table ST.7.17.1.4.1. The log-normal distribution properties for the $K_{eq}$ of Reaction 65 are shown in Table ST.7.17.1.4.2 and plotted in Figure SF.7.17.1.4.1.

**Table ST.7.17.1.4.1.** Literature information used to design the COX-1 $K_{eq}$ parameter distribution. Each value was assigned a weight using the protocol described in Section 2.6.2. The type of error was assigned as 0 if it was additive and 1 if it was multiplicative.

| ΔG (kcal/mol) | $K_{eq}$ | Experimental details | | | | | | Weight | Type of error | Reference |
|---|---|---|---|---|---|---|---|---|---|---|
| | | Species | Expression Vector | Enzyme | pH | Temperature (°C) | Other | | | |
| $-3.90 \times 10^{1}$ | $4.18 \times 10^{28}$ | Human | Unknown | COX-1 | 7 | Unknown | | 64 | 0 | (Caspi et al., 2018) |

**Table ST.7.17.1.4.2.** The log-normal distribution properties of the COX-1 $K_{eq}$ distribution. These values were calculated using the functions described in Section 2.6.2.

| Mode | Confidence Interval | Location parameter (μ) | Scale parameter (σ) |
|---|---|---|---|
| $4.18 \times 10^{28}$ | $1.00 \times 10^{1}$ | $6.67 \times 10^{1}$ | $8.90 \times 10^{-1}$ |

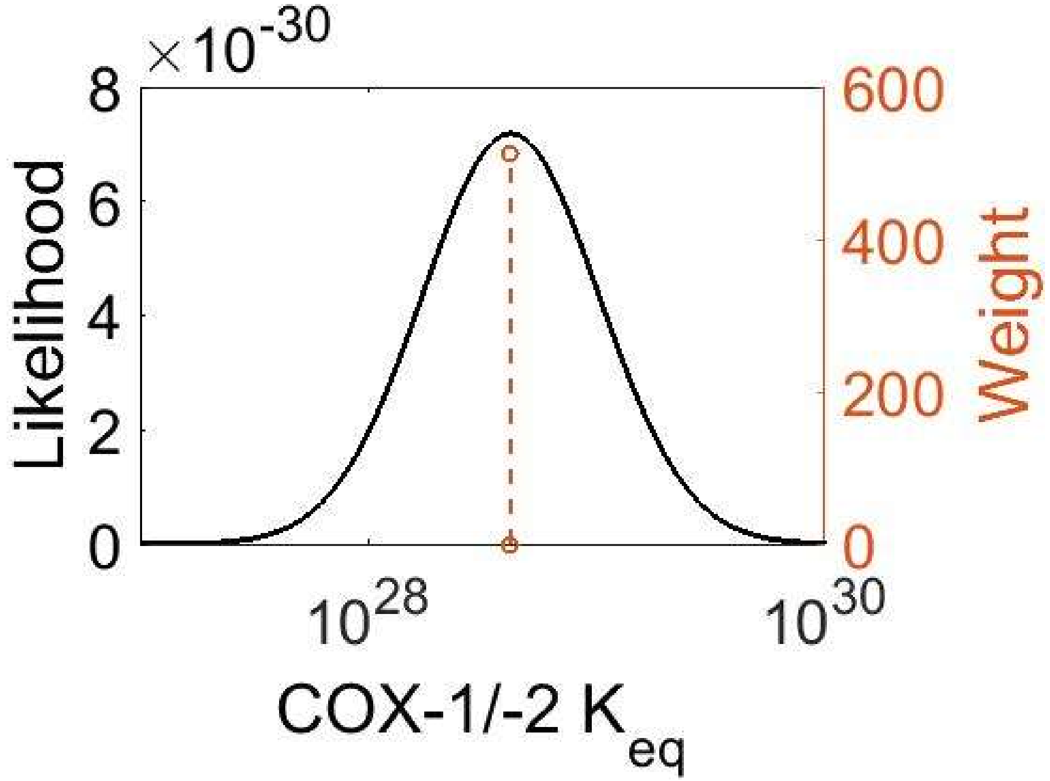


**Figure SF.7.17.1.4.1.** The estimated probability distribution for COX-1 $K_{eq}$. The value and weight of the literature values used to define the distribution are indicated by an orange dashed line. The x axis is plotted on a log-scale.

### S.7.17.1.5. Parameter: COX-1 concentration

Parameter values for the COX-1 concentration of Reaction 65 were obtained from the literature and summarised in Table ST.7.17.1.5.1. The log-normal distribution properties for the COX-1 concentration of Reaction 65 are shown in Table ST.7.17.1.5.2 and plotted in Figure SF.7.17.1.5.1. To convert the enzyme concentration from ppm to mM, **Equation S.6.2** was used.

**Table ST.7.17.1.5.1.** Literature information used to design the COX-1 concentration parameter distribution. Each value was assigned a weight using the protocol described in Section 2.6.2. The type of error was assigned as 0 if it was additive and 1 if it was multiplicative.

| Value (ppm) | Error (ppm) | Experimental details | | | | | | Weight | Type of error | Reference |
|---|---|---|---|---|---|---|---|---|---|---|
| | | Species | Expression Vector | Enzyme | pH | Temperature (°C) | Other | | | |
| 1.91 $x10^1$ | NaN | Human | Lung | COX-1 | 7.5 | 37 | | **1024** | 0 | (Kim et al., 2014) |
| 7.08 $x10^1$ | NaN | Human | Oral cavity | COX-1 | 7.5 | 37 | | **1024** | 0 | (Wilhelm et al., 2014) |
| 1.30 $x10^2$ | NaN | Human | Oesophagus | COX-1 | 7.5 | 37 | | **1024** | 0 | (Wilhelm et al., 2014) |
| 2.08 $x10^2$ | NaN | Human | Stomach | COX-1 | 7.5 | 37 | | **1024** | 0 | (Wilhelm et al., 2014) |
| 8.49 $x10^2$ | NaN | Human | Platelet | COX-1 | 7.5 | 37 | | **1024** | 0 | (Kim et al., 2014) |

**Table ST.7.17.1.5.2.** The log-normal distribution properties of the COX-1 concentration distribution. These values were calculated using the functions described in Section 2.6.2.

| Mode (ppm) | Mode (mM) | Confidence Interval | Location parameter (μ) | Scale parameter (σ) |
|---|---|---|---|---|
| 1.29 $x10^2$ | 7.14 $x10^{-4}$ | 3.49 | 5.66 | 8.94 $x10^{-1}$ |

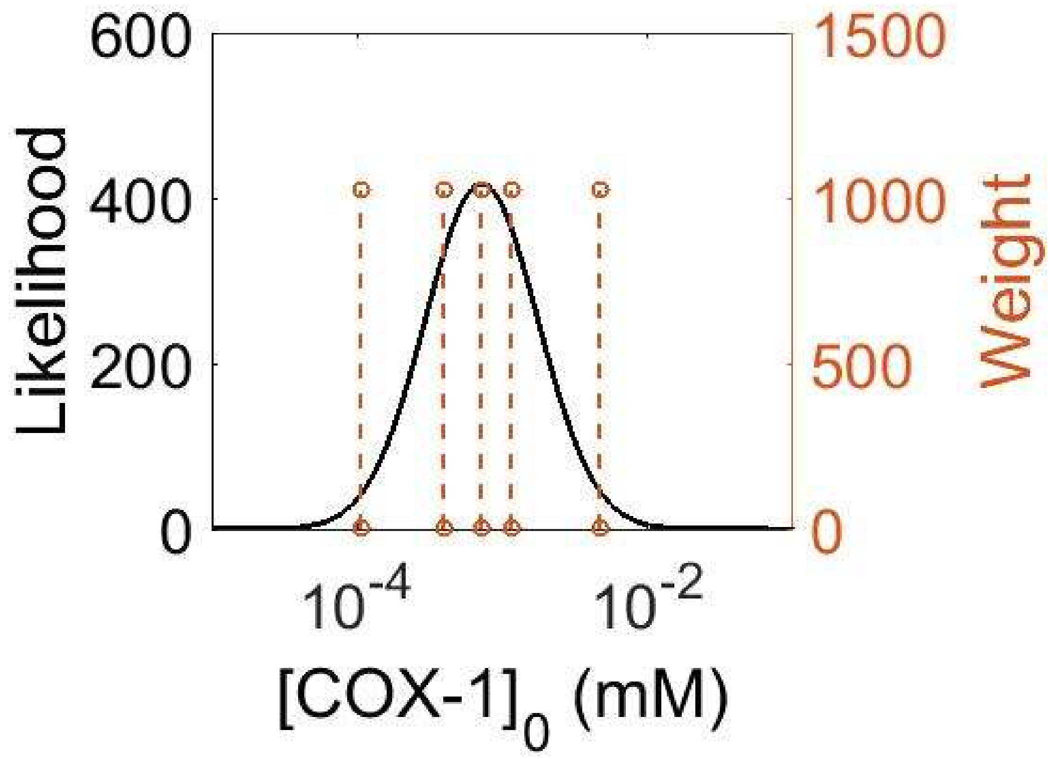


**Figure SF.7.17.1.5.1.** The estimated probability distribution for the COX-1 concentration. The value and weight of the literature values used to define the distribution are indicated by an orange dashed line. The x axis is plotted on a log-scale.

### S.7.18 Reaction 66: $PGE_2 \rightleftharpoons$ 15-keto-$PGE_2$

$PGE_2$ is metabolised by 15-prostoglandin dehydrogenase (15-PGDH), via the oxidation of the 15(S)-hydroxyl group of $PGE_2$ into a ketone (Anggard and Samuelsson, 1965). The product of this reaction, 15-keto-$PGE_2$, has less biological activity than $PGE_2$ therefore is of therapeutic interest (Lim et al., 2010; Myung et al., 2006).

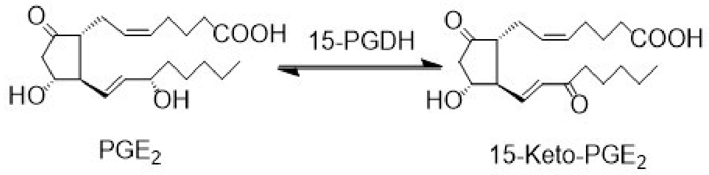


**Figure SF.7.18.** The oxidation reaction of prostaglandin $E_2$ ($PGE_2$) into 15-keto-prostaglandin $E_2$ (15-keto-$PGE_2$), by 15-prostoglandin dehydrogenase (15-PGDH) (Reaction 66).

**SEq.7.18. Reaction rate law for Reaction 66.**

$$v_{66} = \frac{K_{cat} \cdot [15-PGDH]\left([PGE_2] - {[15-\text{keto}-\text{PGE}_2]}/{K_{eq}}\right)}{K_{m_s}\left(1 + \frac{[15-\text{keto}-\text{PGE}_2]}{K_{m_p}}\right) + [PGE_2]}$$

### S.7.18.1. Reaction parameters

#### S.7.18.1.1. Parameter: 15-PGDH $K_{ms}$

Parameter values for the $K_{ms}$ of Reaction 66 were obtained from the literature and summarised in Table ST.7.18.1.1.1. The log-normal distribution properties for the $K_{ms}$ of Reaction 66 are shown in Table ST.7.18.1.1.2 and plotted in Figure SF.7.18.1.1.1.

**Table ST.7.18.1.1.1.** Literature information used to design the 15-PGDH $K_{ms}$ parameter distribution. Each value was assigned a weight using the protocol described in Section 2.6.2. The type of error was assigned as 0 if it was additive and 1 if it was multiplicative.

| Value (mM) | Error (mM) | Experimental details | | | | | | Weight | Type of error | Reference |
|---|---|---|---|---|---|---|---|---|---|---|
| | | Species | Expression Vector | Enzyme | pH | Temperature (°C) | Other | | | |
| $8.00 \times 10^{-3}$ | | Rat | Skin | 15-PGDH | 7 | 37 | | 1024 | | (Fincham and Camp, 1983) |
| $7.50 \times 10^{-3}$ | | Rat | Skin | 15-PGDH | 7 | 37 | | 1024 | | (Fincham and Camp, 1983) |
| $2.40 \times 10^{-2}$ | | Rat | Skin | 15-PGDH | 7 | 37 | | 1024 | | (Fincham and Camp, 1983) |
| $2.30 \times 10^{-2}$ | | Rat | Skin | 15-PGDH | 7 | 37 | | 1024 | | (Fincham and Camp, 1983) |
| $3.90 \times 10^{-3}$ | | Human | E. coli. | 15-PGDH | 7 | 37 | | 1024 | | (Zhou et al., 2001) |
| $9.90 \times 10^{-3}$ | | Rat | E. coli. | 15-PGDH | 7 | 37 | | 512 | | (Zhou et al., 2001) |
| $5.50 \times 10^{-3}$ | $6.00 \times 10^{-4}$ | Human | E. coli. | 15-PGDH | 7 | 37 | | 1024 | | (Niesen et al., 2010) |

**Table ST.7.18.1.1.2.** The log-normal distribution properties of the 15-PGDH $K_{ms}$ distribution. These values were calculated using the functions described in Section 2.6.2.

| Mode (mM) | Confidence Interval | Location parameter (μ) | Scale parameter (σ) |
|---|---|---|---|
| $7.64 \times 10^{-3}$ | 5.05 | -4.41 | $6.80 \times 10^{-1}$ |

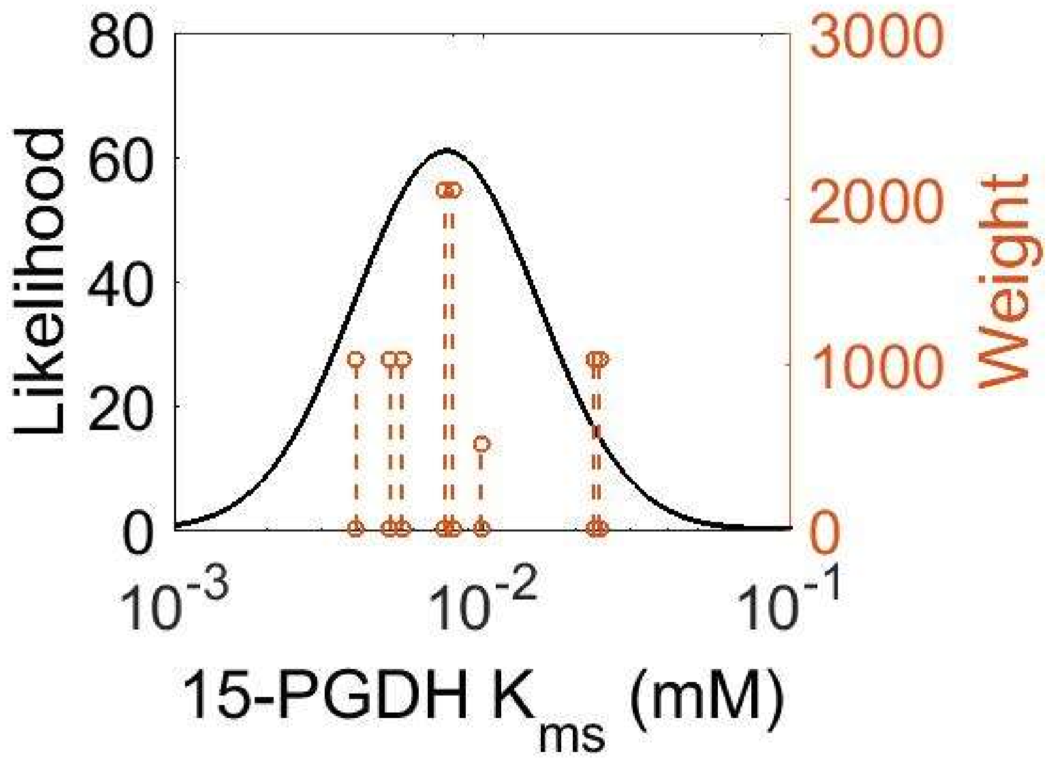


**Figure SF.7.18.1.1.1.** The estimated probability distribution for 15-PGDH $K_{ms}$. The value and weight of the literature values used to define the distribution are indicated by an orange dashed line. The x axis is plotted on a log-scale.

**S.7.18.1.2. Parameter: 15-PGDH $K_{mp}$ (Dependent parameter)**

The log-normal distribution for the parameter for the $K_{mp}$ of Reaction 66 was calculated using multivariate distributions. The log-normal distribution properties for the $K_{mp}$ of Reaction 66 are shown in Table ST.7.18.1.2.1 and plotted in Figure SF.7.18.1.2.1.

**Table ST.7.18.1.2.1.** The log-normal distribution properties of the 15-PGDH $K_{mp}$ distribution. These values were calculated using the functions described in Section 2.6.2.

| Mode (mM) | Location parameter (μ) | Scale parameter (σ) |
|---|---|---|
| $7.70 \times 10^{-3}$ | -4.41 | $6.72 \times 10^{-1}$ |

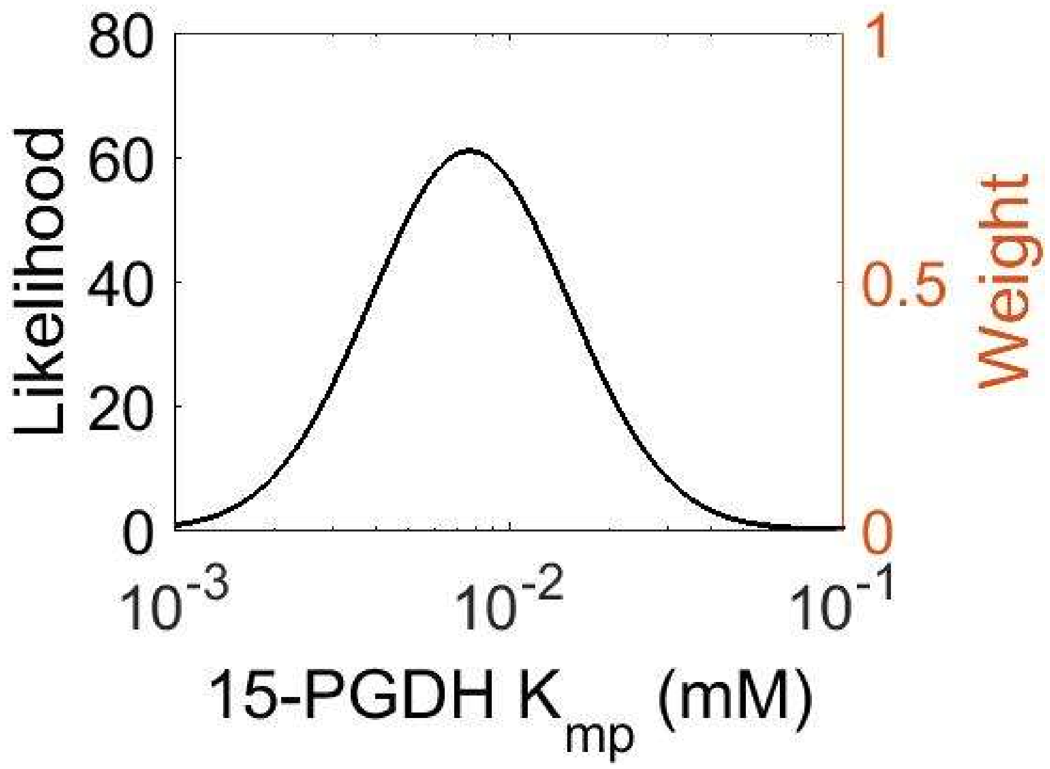


**Figure SF.7.18.1.2.1.** The estimated probability distribution for 15-PGDH $K_{mp}$. The value and weight of the literature values used to define the distribution are indicated by an orange dashed line. The x axis is plotted on a log-scale.

### S.7.18.1.3. Parameter: 15-PGDH kcat

Parameter values for the $k_{cat}$ of Reaction 66 were obtained from the literature and summarised in Table ST.7.18.1.3.1. The log-normal distribution properties for the $k_{cat}$ of Reaction 66 are shown in Table ST.7.18.1.3.2 and plotted in Figure SF.7.18.1.3.1.

**Table ST.7.18.1.3.1.** Literature information used to design the 15-PGDH $k_{cat}$ parameter distribution. Each value was assigned a weight using the protocol described in Section 2.6.2. The type of error was assigned as 0 if it was additive and 1 if it was multiplicative.

| Value ($min^{-1}$) | Error ($min^{-1}$) | Experimental details | | | | | | Weight | Type of error | Reference |
|---|---|---|---|---|---|---|---|---|---|---|
| | | Species | Expression Vector | Enzyme | pH | Temperature (°C) | Other | | | |
| $8.16 \times 10^{2}$ | $1.80 \times 10^{1}$ | Human | E. coli. | 15-PGDH | 8 | 25 | | 128 | 0 | (Niesen et al., 2010) |
| $3.66 \times 10^{2}$ | $1.20 \times 10^{1}$ | Human | E. coli. | 15-PGDH | 8 | 25 | | 128 | | (Niesen et al., 2010) |
| $8.46 \times 10^{2}$ | $1.20 \times 10^{1}$ | Human | E. coli. | 15-PGDH | 8 | 25 | | 128 | | (Niesen et al., 2010) |

**Table ST.7.18.1.3.2.** The log-normal distribution properties of the 15-PGDH $k_{cat}$ distribution. These values were calculated using the functions described in Section 2.6.2.

| Mode ($min^{-1}$) | Confidence Interval | Location parameter (μ) | Scale parameter (σ) |
|---|---|---|---|
| $8.12 \times 10^{2}$ | 5.38 | 7.19 | $7.01 \times 10^{-1}$ |

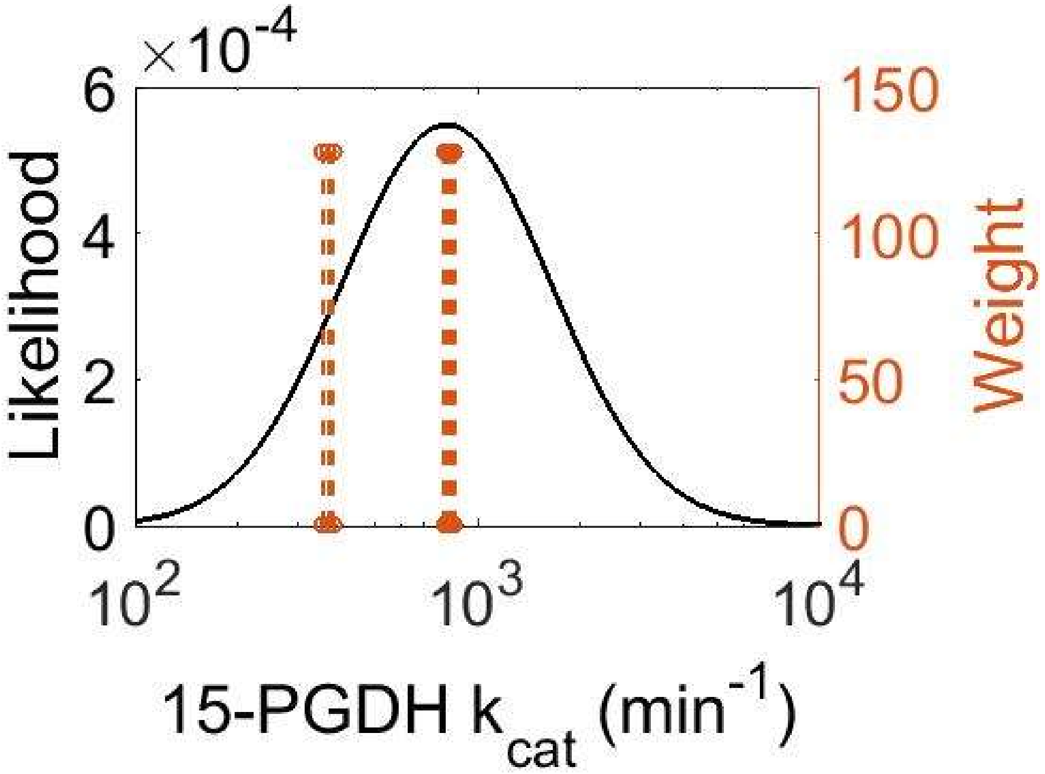


**Figure SF.7.18.1.3.1.** The estimated probability distribution for 15-PGDH $k_{cat}$. The value and weight of the literature values used to define the distribution are indicated by an orange dashed line. The x axis is plotted on a log-scale.

### S.7.18.1.4. Parameter: 15-PGDH $K_{eq}$

Parameter values for the $K_{eq}$ of Reaction 66 were obtained from the literature and summarised in Table ST.7.18.1.4.1. The log-normal distribution properties for the $K_{eq}$ of Reaction 66 are shown in Table ST.7.18.1.4.2 and plotted in Figure SF.7.18.1.4.1.

**Table ST.7.18.1.4.1.** Literature information used to design the 15-PGDH $K_{eq}$ parameter distribution. Each value was assigned a weight using the protocol described in Section 2.6.2. The type of error was assigned as 0 if it was additive and 1 if it was multiplicative.

| ΔG (kcal/mol) | $K_{eq}$ | Experimental details | | | | | | Weight | Type of error | Reference |
|---|---|---|---|---|---|---|---|---|---|---|
| | | Species | Expression Vector | Enzyme | pH | Temperature (°C) | Other | | | |
| $-4.68 \times 10^{-1}$ | 2.21 | Human | Unknown | 15-PGDH | 7 | Unknown | | 64 | 0 | (Caspi et al., 2018) |

**Table ST.7.18.1.4.2.** The log-normal distribution properties of the 15-PGDH $K_{eq}$ distribution. These values were calculated using the functions described in Section 2.6.2.

| Mode | Confidence Interval | Location parameter (μ) | Scale parameter (σ) |
|---|---|---|---|
| 2.21 | $1.00 \times 10^{1}$ | 1.58 | $8.91 \times 10^{-1}$ |

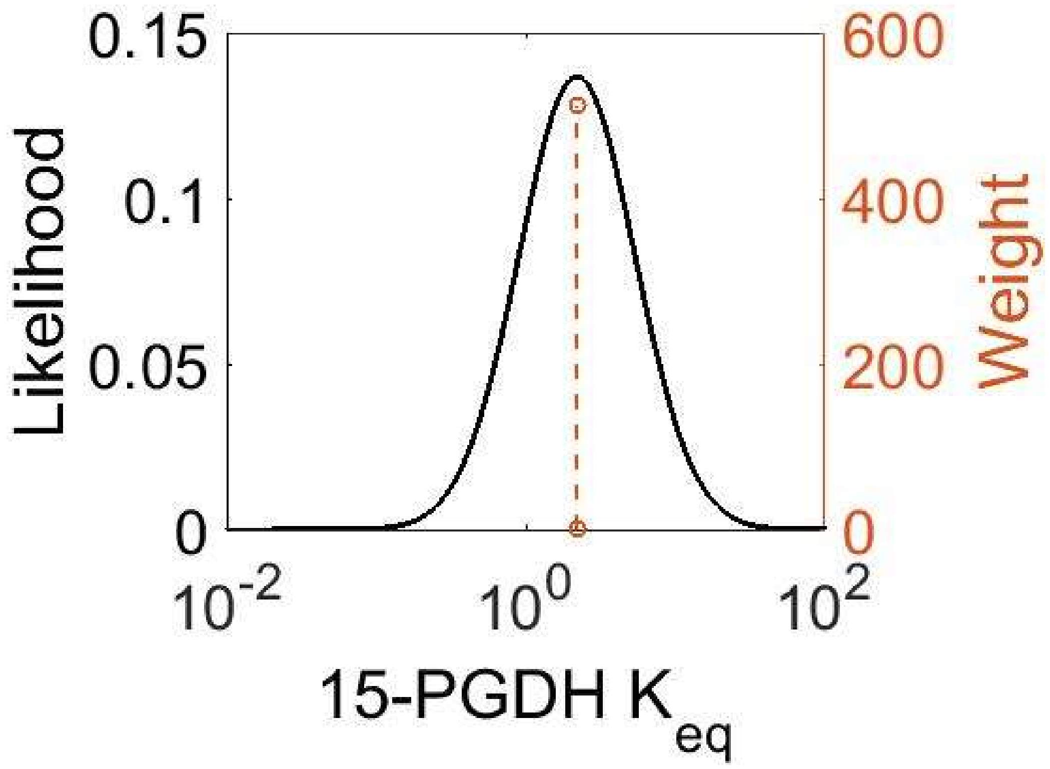


**Figure SF.7.18.1.4.1.** The estimated probability distribution for 15-PGDH $K_{eq}$. The value and weight of the literature values used to define the distribution are indicated by an orange dashed line. The x axis is plotted on a log-scale.

### S.7.18.1.5. Parameter: 15-PGDH concentration

Parameter values for the 15-PGDH concentration of Reaction 66 were obtained from the literature and summarised in Table ST.7.18.1.5.1. The log-normal distribution properties for the 15-PGDH concentration of Reaction 66 are shown in Table ST.7.18.1.5.2 and plotted in Figure SF.7.18.1.5.1. To convert the enzyme concentration from ppm to mM, **Equation S.6.2** was used.

**Table ST.7.18.1.5.1.** Literature information used to design the 15-PGDH concentration parameter distribution. Each value was assigned a weight using the protocol described in Section 2.6.2. The type of error was assigned as 0 if it was additive and 1 if it was multiplicative.

| Value (ppm) | Error (ppm) | Experimental details | | | | | | Weight | Type of error | Reference |
|---|---|---|---|---|---|---|---|---|---|---|
| | | Species | Expression Vector | Enzyme | pH | Temperature (°C) | Other | | | |
| 7.68 | NaN | Human | Skin | 15-PGDH | 7.5 | 37 | | **2048** | 0 | (Kim et al., 2014) |
| 1.12 x10$^{1}$ | NaN | Human | Oesophagus | 15-PGDH | 7.5 | 37 | | **1024** | 0 | (Kim et al., 2014) |
| 2.21 x10$^{1}$ | NaN | Human | Heart | 15-PGDH | 7.5 | 37 | | **1024** | 0 | (Kim et al., 2014) |
| 6.98 x10$^{1}$ | NaN | Human | Oesophagus | 15-PGDH | 7.5 | 37 | | **1024** | 0 | (Wilhelm et al., 2014) |

**Table ST.7.18.1.5.2.** The log-normal distribution properties of the 15-PGDH concentration distribution. These values were calculated using the functions described in Section 2.6.2.

| Mode (ppm) | Mode (mM) | Confidence Interval | Location parameter (μ) | Scale parameter (σ) |
|---|---|---|---|---|
| 1.12 x10$^{1}$ | 6.20 x10$^{-5}$ | 2.32 | 2.87 | 6.80 x10$^{-1}$ |

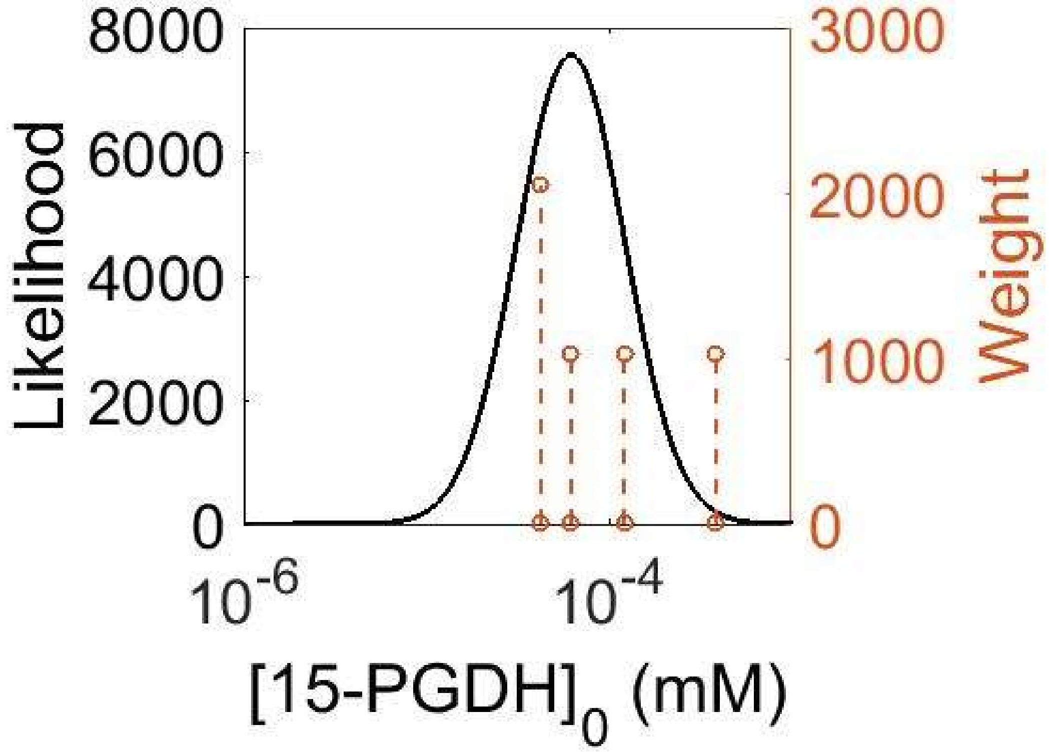


**Figure SF.7.18.1.5.1.** The estimated probability distribution for the 15-PGDH concentration. The value and weight of the literature values used to define the distribution are indicated by an orange dashed line. The x axis is plotted on a log-scale.

### S.7.19 Reaction 69: 15-keto-PGE$_2$ ⇌ 13,14-dihydro-15-keto-PGE$_2$

15-keto-PGE$_2$ is a substrate for prostaglandin reductase 2 (PTGR-2), which catalyses the formation of 13,14-dihydro-15-keto-PGE$_2$. In this reaction, the conjugated α, β-unsaturated double bond between C13 and C14 is reduced.

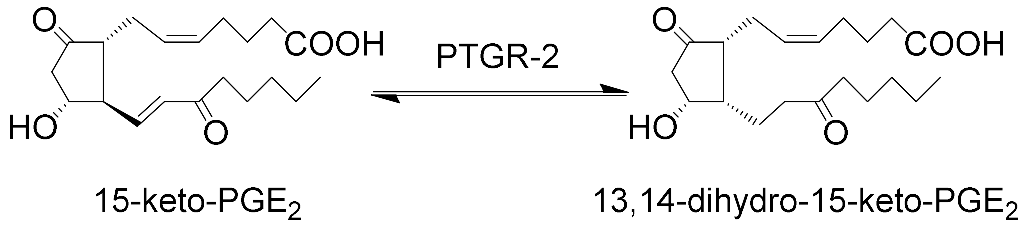


**Figure SF.7.19.** The reduction of 15-keto-prostaglandin E$_2$ (15-keto-PGE$_2$) to 13,14-dihydro-15-keto-prostaglandin E$_2$ (13,14-dihydro-15-keto-PGE$_2$), by prostaglandin reductase 2 (PTGR-2) (Reaction 69).

**SEq.7.19. Reaction rate law for Reaction 69.**

$$v_{69} = \frac{K_{cat} \cdot [\text{PTGR2}] \left( [\text{15-keto-PGE}_2] - {}^{[\text{13,14-dihydro-15-keto-PGE}_2]}\!/_{K_{eq}} \right)}{K_{m_s}\left(1 + \frac{[\text{13,14-dihydro-15-keto-PGE}_2]}{K_{m_p}}\right) + [\text{15-keto-PGE}_2]}$$

### S.7.19.1. Reaction parameters

#### S.7.19.1.1. Parameter: PTGR-2 $K_{ms}$

Parameter values for the $K_{ms}$ of Reaction 69 were obtained from the literature and summarised in Table ST.7.19.1.1.1. The log-normal distribution properties for the $K_{ms}$ of Reaction 69 are shown in Table ST.7.19.1.1.2 and plotted in Figure SF.7.19.1.1.1.

**Table ST.7.19.1.1.1.** Literature information used to design the PTGR-2 $K_{ms}$ parameter distribution. Each value was assigned a weight using the protocol described in Section 2.6.2. The type of error was assigned as 0 if it was additive and 1 if it was multiplicative.

| Value (mM) | Error (mM) | Experimental details | | | | | | Weight | Type of error | Reference |
|---|---|---|---|---|---|---|---|---|---|---|
| | | Species | Expression Vector | Enzyme | pH | Temperature (°C) | Other | | | |
| 1.12 $\times 10^{-2}$ | 1.40 $\times 10^{-4}$ | Human | E Coli | PTGR2 | 7 | 37 | | **512** | | (Wu et al., 2008) |
| 1.59 $\times 10^{-2}$ | 1.71 $\times 10^{-3}$ | Human | E Coli | PTGR2 | 7 | 37 | | **512** | | (Wu et al., 2008) |

**Table ST.7.19.1.1.2.** The log-normal distribution properties of the PTGR-2 $K_{ms}$ distribution. These values were calculated using the functions described in Section 2.6.2.

| Mode (mM) | Confidence Interval | Location parameter (μ) | Scale parameter (σ) |
|---|---|---|---|
| 1.20 $\times 10^{-2}$ | 4.92 | -3.97 | 6.71 $\times 10^{-1}$ |

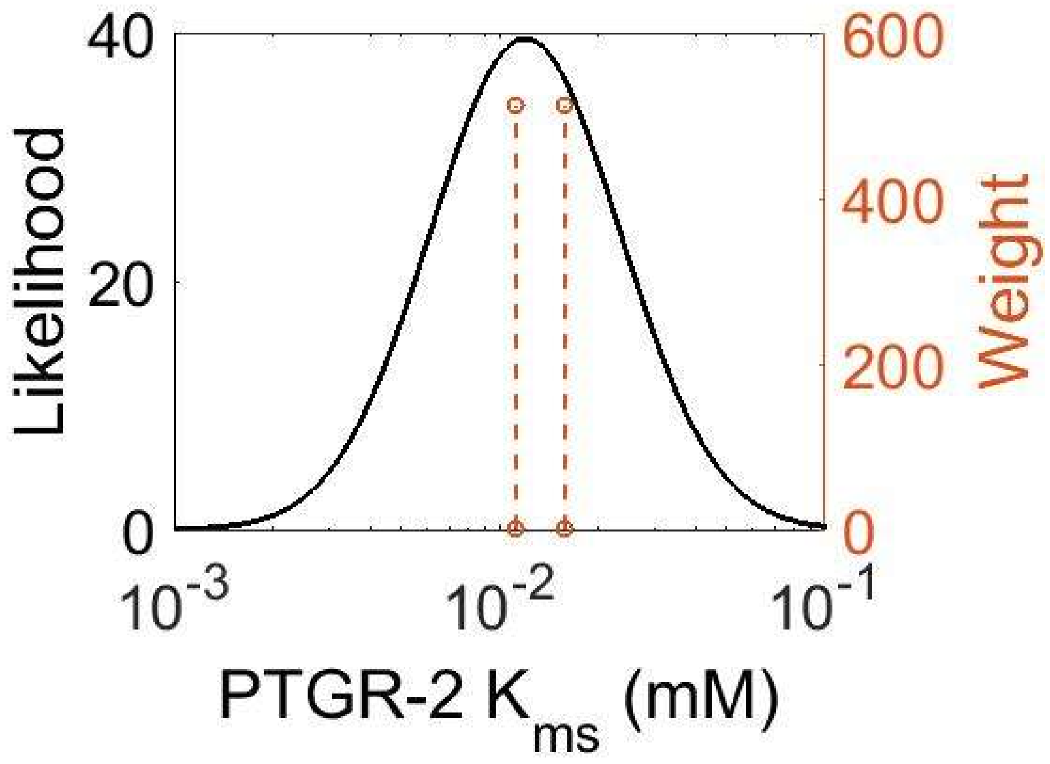


**Figure SF.7.19.1.1.1.** The estimated probability distribution for PTGR-2 $K_{ms}$. The value and weight of the literature values used to define the distribution are indicated by an orange dashed line. The x axis is plotted on a log-scale.

### S.7.19.1.2. Parameter: PTGR-2 $K_{mp}$

The log-normal distribution for the parameter for the $K_{mp}$ of Reaction 69 was calculated using multivariate distributions. The log-normal distribution properties for the $K_{mp}$ of Reaction 69 are shown in Table ST.7.19.1.2.1 and plotted in Figure SF.7.19.1.2.1.

**Table ST.7.19.1.2.1.** The log-normal distribution properties of the PTGR-2 $K_{mp}$ distribution. These values were calculated using the functions described in Section 2.6.2.

| Mode (mM) | Location parameter (μ) | Scale parameter (σ) |
|---|---|---|
| $1.19 \times 10^{-2}$ | -3.97 | $6.81 \times 10^{-1}$ |

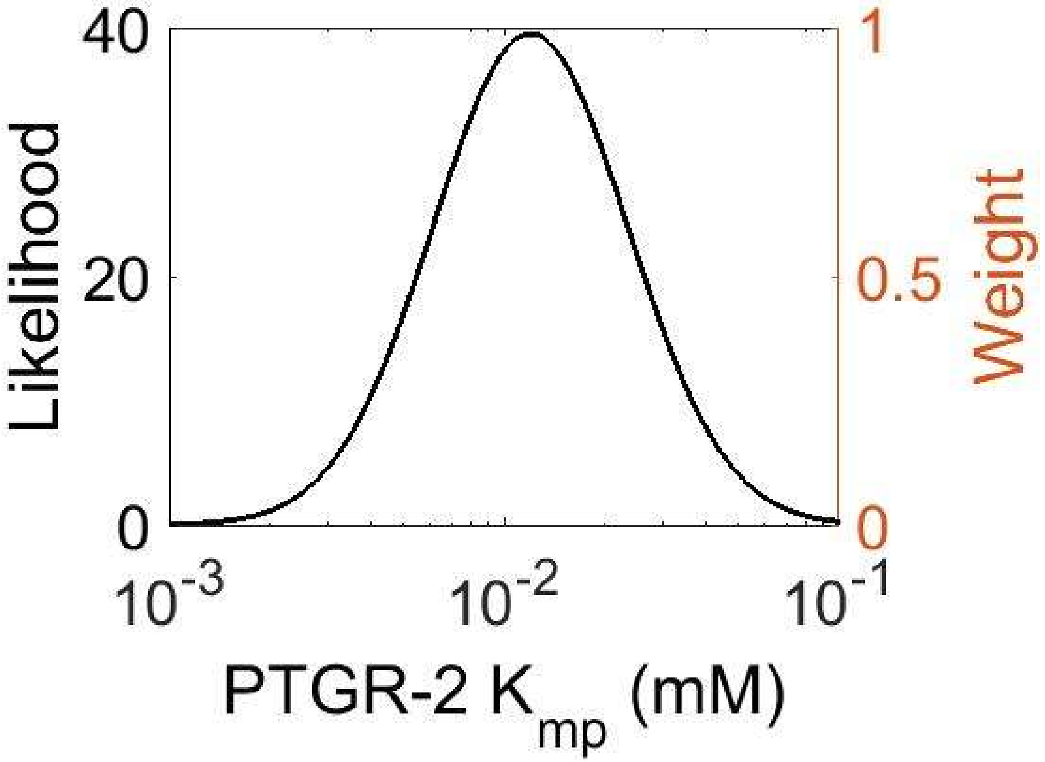


**Figure SF.7.19.1.2.1.** The estimated probability distribution for PTGR-2 $K_{mp}$. The value and weight of the literature values used to define the distribution are indicated by an orange dashed line. The x axis is plotted on a log-scale.

### S.7.19.1.3. Parameter: PTGR-2 $k_{cat}$

Parameter values for the $k_{cat}$ of Reaction 69 were obtained from the literature and summarised in Table ST.7.19.1.3.1. The log-normal distribution properties for the $k_{cat}$ of Reaction 69 are shown in Table ST.7.19.1.3.2 and plotted in Figure SF.7.19.1.3.1.

**Table ST.7.19.1.3.1.** Literature information used to design the PTGR-2 $k_{cat}$ parameter distribution. Each value was assigned a weight using the protocol described in Section 2.6.2. The type of error was assigned as 0 if it was additive and 1 if it was multiplicative.

| Value ($min^{-1}$) | Error ($min^{-1}$) | Experimental details | | | | | | Weight | Type of error | Reference |
|---|---|---|---|---|---|---|---|---|---|---|
| | | Species | Expression Vector | Enzyme | pH | Temperature (°C) | Other | | | |
| 1.14 x$10^{1}$ | 9.00 x$10^{-1}$ | Mouse | Mouse cell | 13,14-dehydro-15-oxoprostaglandin 13-reductase | 7.4 | 37 | | 384 | | (Wu et al., 2008) |

**Table ST.7.19.1.3.2.** The log-normal distribution properties of the PTGR-2 $k_{cat}$ distribution. These values were calculated using the functions described in Section 2.6.2.

| Mode ($min^{-1}$) | Confidence Interval | Location parameter (μ) | Scale parameter (σ) |
|---|---|---|---|
| 1.14 x$10^{1}$ | 9.86 | 3.22 | 8.87 x$10^{-1}$ |

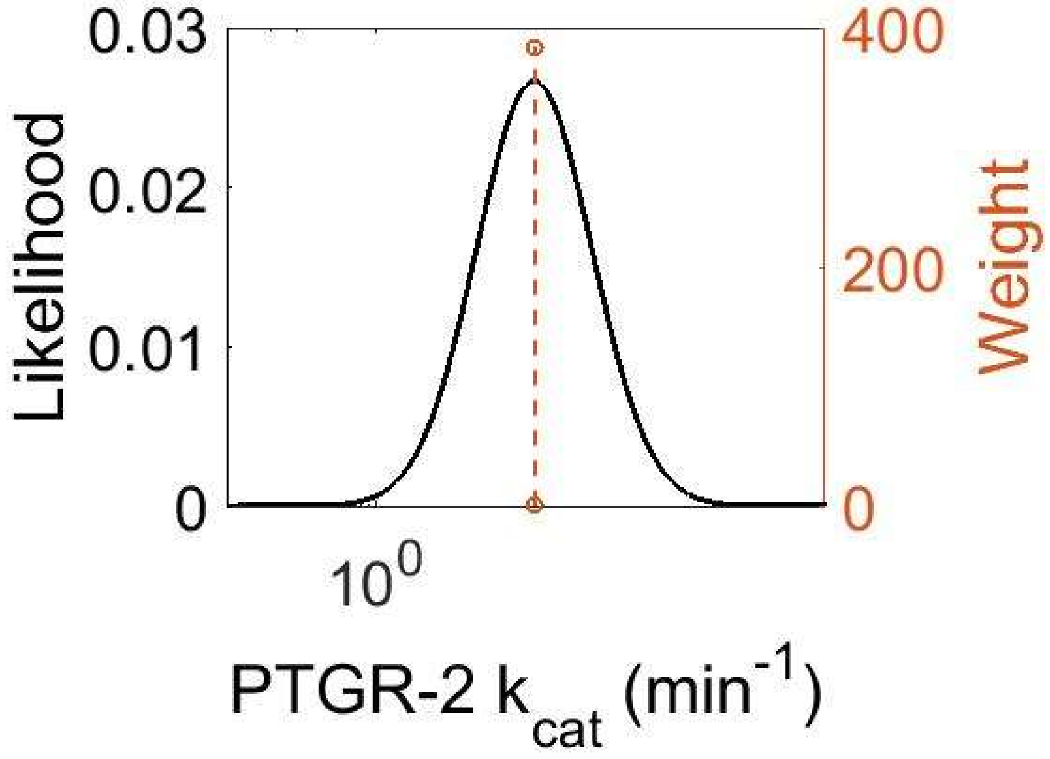


**Figure SF.7.19.1.3.1.** The estimated probability distribution for PTGR-2 $k_{cat}$. The value and weight of the literature values used to define the distribution are indicated by an orange dashed line. The x axis is plotted on a log-scale.

### S.7.19.1.4. Parameter: PTGR-2 $K_{eq}$

Parameter values for the $K_{eq}$ of Reaction 69 were obtained from the literature and summarised in Table ST.7.19.1.4.1. The log-normal distribution properties for the $K_{eq}$ of Reaction 69 are shown in Table ST.7.19.1.4.2 and plotted in Figure SF.7.19.1.4.1.

**Table ST.7.19.1.4.1.** Literature information used to design the PTGR-2 $K_{eq}$ parameter distribution. Each value was assigned a weight using the protocol described in Section 2.6.2. The type of error was assigned as 0 if it was additive and 1 if it was multiplicative.

| ΔG (kcal/mol) | $K_{eq}$ | Experimental details | | | | | | Weight | Type of error | Reference |
|---|---|---|---|---|---|---|---|---|---|---|
| | | Species | Expression Vector | Enzyme | pH | Temperature (°C) | Other | | | |
| 3.60 | 2.28 x10$^{-3}$ | Human | Unknown | 15-PGDH | 7 | Unknown | | 64 | 0 | (Caspi et al., 2018) |

**Table ST.7.19.1.4.2.** The log-normal distribution properties of the PTGR-2 $K_{eq}$ distribution. These values were calculated using the functions described in Section 2.6.2.

| Mode | Confidence Interval | Location parameter (μ) | Scale parameter (σ) |
|---|---|---|---|
| 2.28 x10$^{-3}$ | 1.00 x10$^{1}$ | -5.29 | 8.91 x10$^{-1}$ |

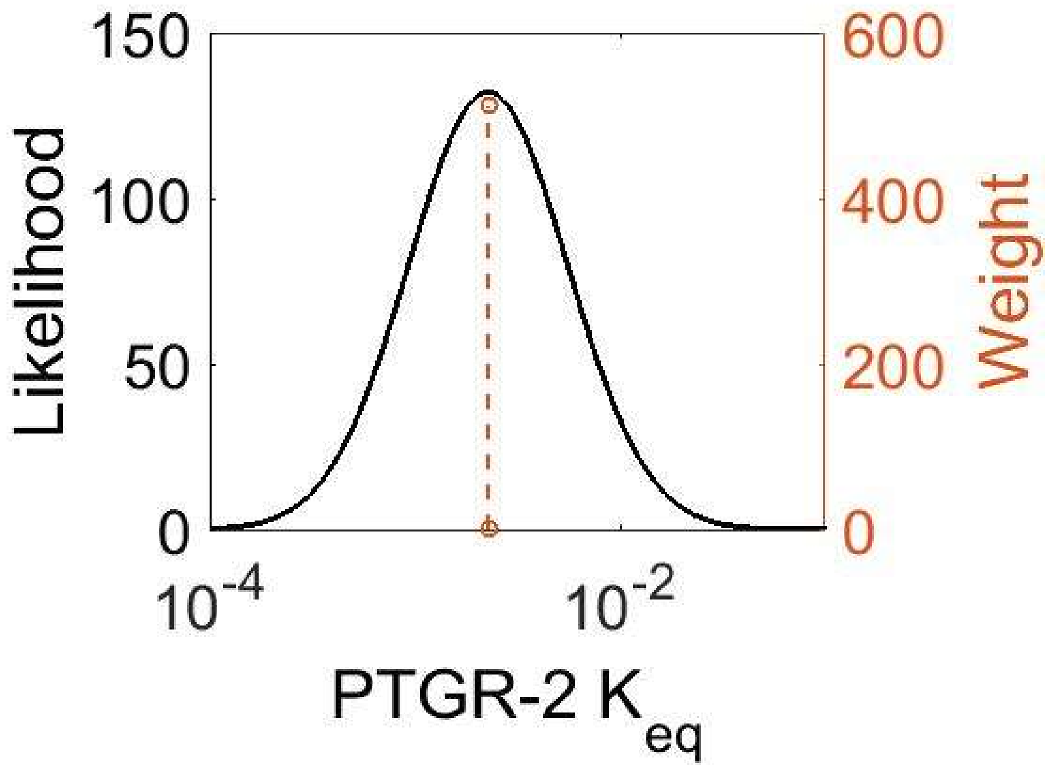


**Figure SF.7.19.1.4.1.** The estimated probability distribution for PTGR-2 $K_{eq}$. The value and weight of the literature values used to define the distribution are indicated by an orange dashed line. The x axis is plotted on a log-scale.

### S.7.19.1.5. Parameter: PTGR-2 concentration

Parameter values for the PTGR-2 concentration of Reaction 69 were obtained from the literature and summarised in Table ST.7.19.1.5.1. The log-normal distribution properties for the PTGR-2 concentration of Reaction 69 are shown in Table ST.7.19.1.5.2 and plotted in Figure SF.7.19.1.5.1. To convert the enzyme concentration from ppm to mM, **Equation S.6.2** was used.

**Table ST.7.19.1.5.1.** Literature information used to design the PTGR-2 concentration parameter distribution. Each value was assigned a weight using the protocol described in Section 2.6.2. The type of error was assigned as 0 if it was additive and 1 if it was multiplicative.

| Value (ppm) | Error (ppm) | Experimental details | | | | | | Weight | Type of error | Reference |
|---|---|---|---|---|---|---|---|---|---|---|
| | | Species | Expression Vector | Enzyme | pH | Temperature (°C) | Other | | | |
| $1.62 \times 10^{2}$ | | Human | Skin | PTGR2 | 7.5 | 37 | | 2048 | | (Wilhelm et al., 2014) |
| $8.09 \times 10^{1}$ | | Human | Skin | PTGR2 | 7.5 | 37 | | 2048 | | (Wilhelm et al., 2014) |
| $7.41 \times 10^{1}$ | | Human | Oral cavity | PTGR2 | 7.5 | 37 | | 1024 | | (Wilhelm et al., 2014) |

**Table ST.7.19.1.5.2.** The log-normal distribution properties of the PTGR-2 concentration distribution. These values were calculated using the functions described in Section 2.6.2.

| Mode (ppm) | Mode (mM) | Confidence Interval | Location parameter (μ) | Scale parameter (σ) |
|---|---|---|---|---|
| $8.65 \times 10^{1}$ | $4.79 \times 10^{-4}$ | 1.45 | 4.58 | $3.47 \times 10^{-1}$ |

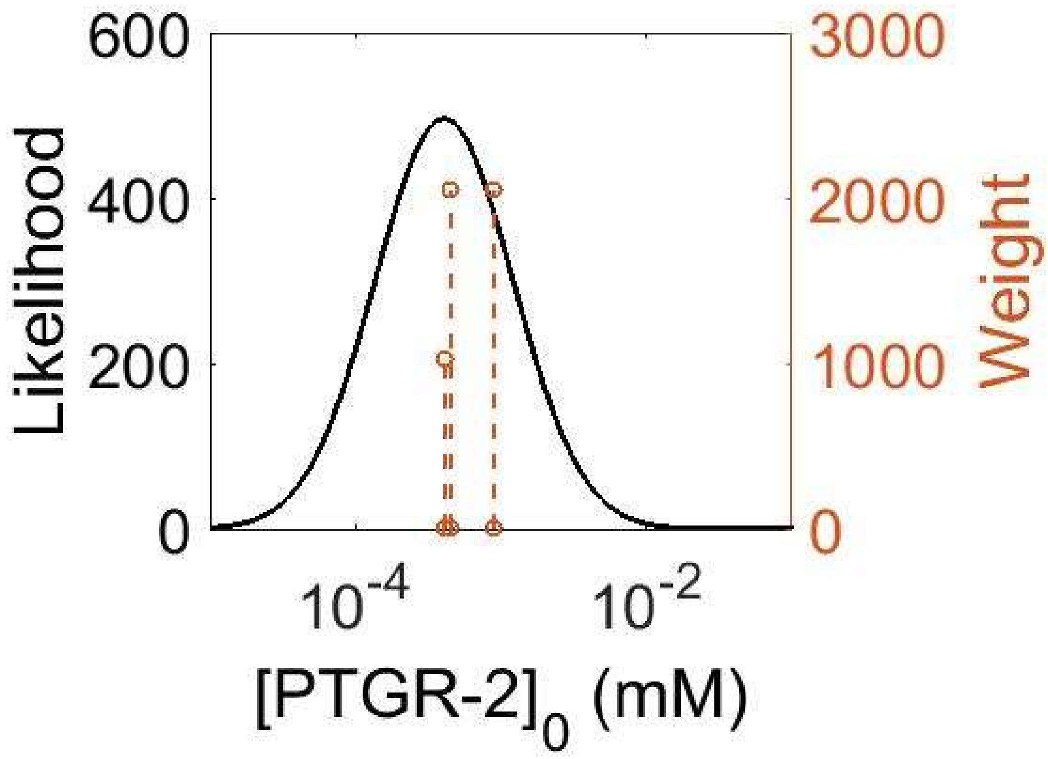


**Figure SF.7.19.1.5.1.** The estimated probability distribution for the PTGR-2 concentration. The value and weight of the literature values used to define the distribution are indicated by an orange dashed line. The x axis is plotted on a log-scale.

## References


Abramovitz, M., et al. (1993). ‘5-lipoxygenase-activating protein stimulates the utilization of arachidonic acid by 5-lipoxygenase’ *Eur J Biochem*, 215 (1), pp. 105-11.

Anggard, E. & Samuelsson, B. (1965). ‘Biosynthesis of prostaglandins from arachidonic acid in guinea pig lung. Prostaglandins and related factors. 38’ *J Biol Chem*, 240 (9), pp. 3518-21.

Bambai, B., Rogge, C. E., Stec, B. & Kulmacz, R. J. (2004). ‘Role of asn-382 and thr-383 in activation and inactivation of human prostaglandin h synthase cyclooxygenase catalysis’ *J Biol Chem*, 279 (6), pp. 4084-92. DOI: 10.1074/jbc.M304762200.

Brock, T. G., Paine, R., 3rd & Peters-Golden, M. (1994). ‘Localization of 5-lipoxygenase to the nucleus of unstimulated rat basophilic leukemia cells’ *J Biol Chem*, 269 (35), pp. 22059-66.

Bryant, R. W., Bailey, J. M., Schewe, T. & Rapoport, S. M. (1982). ‘Positional specificity of a reticulocyte lipoxygenase. Conversion of arachidonic acid to 15-s-hydroperoxy-eicosatetraenoic acid’ *J Biol Chem*, 257 (11), pp. 6050-5.

Burrall, B. A., Cheung, M., Chiu, A. & Goetzl, E. J. (1988). ‘Enzymatic properties of the 15-lipoxygenase of human cultured keratinocytes’ *J Invest Dermatol*, 91 (4), pp. 294-7.

Caspi, R., et al. (2018). ‘The metacyc database of metabolic pathways and enzymes’ *Nucleic Acids Res*, 46 (D1), pp. D633-d639. DOI: 10.1093/nar/gkx935.

Chen, X. S., Brash, A. R. & Funk, C. D. (1993). ‘Purification and characterization of recombinant histidine-tagged human platelet 12-lipoxygenase expressed in a baculovirus/insect cell system’ *Eur J Biochem*, 214 (3), pp. 845-52.

Coffey, R. J., et al. (1997). ‘Epidermal growth factor receptor activation induces nuclear targeting of cyclooxygenase-2, basolateral release of prostaglandins, and mitogenesis in polarizing colon cancer cells’ *Proc Natl Acad Sci U S A*, 94 (2), pp. 657-62.

DeWitt, D. L. & Meade, E. A. (1993). ‘Serum and glucocorticoid regulation of gene transcription and expression of the prostaglandin h synthase-1 and prostaglandin h synthase-2 isozymes’ *Arch Biochem Biophys*, 306 (1), pp. 94-102. DOI: 10.1006/abbi.1993.1485.

Erlemann, K.-R., et al. (2007). ‘Regulation of 5-hydroxyeicosanoid dehydrogenase activity in monocytic cells’ *Biochem J*, 403 (1), pp. 157-165. DOI: 10.1042/BJ20061617 Available at: https://www.ncbi.nlm.nih.gov/pubmed/17166093

https://www.ncbi.nlm.nih.gov/pmc/PMC1828885/.

Erlemann, K. R., et al. (2007). ‘Regulation of 5-hydroxyeicosanoid dehydrogenase activity in monocytic cells’ *Biochem J*, 403 (1), pp. 157-65. DOI: 10.1042/bj20061617.

Fang, L., et al. (2013). ‘Egf-like growth factors induce cox-2-derived pge2 production through erk1/2 in human granulosa cells’ *J Clin Endocrinol Metab*, 98 (12), pp. 4932-41. DOI: 10.1210/jc.2013-2662.

Fincham, N. & Camp, R. (1983). ‘Novel prostaglandin dehydrogenase in rat skin’ *Biochem J*, 212 (1), pp. 129-34.

Hada, T., Ueda, N., Takahashi, Y. & Yamamoto, S. (1991). ‘Catalytic properties of human platelet 12-lipoxygenase as compared with the enzymes of other origins’ *Biochim Biophys Acta*, 1083 (1), pp. 89-93.

Haeggstrom, J. Z., Tholander, F. & Wetterholm, A. (2007). 'Structure and catalytic mechanisms of leukotriene a4 hydrolase' *Prostaglandins Other Lipid Mediat*, 83 (3), pp. 198-202. DOI: 10.1016/j.prostaglandins.2007.01.006.

Hamza, A., et al. (2010). 'Understanding microscopic binding of human microsomal prostaglandin e synthase-1 (mpges-1) trimer with substrate pgh2 and cofactor gsh: Insights from computational alanine scanning and site-directed mutagenesis' *J Phys Chem B*, 114 (16), pp. 5605-16. DOI: 10.1021/jp100668y.

Hara, S., et al. (1994). 'Isolation and molecular cloning of prostacyclin synthase from bovine endothelial cells' *J Biol Chem*, 269 (31), pp. 19897-903.

Haurand, M. & Ullrich, V. (1985). 'Isolation and characterization of thromboxane synthase from human platelets as a cytochrome p-450 enzyme' *J Biol Chem*, 260 (28), pp. 15059-67.

Hecker, M., et al. (1987). 'Products, kinetics, and substrate specificity of homogeneous thromboxane synthase from human platelets: Development of a novel enzyme assay' *Arch Biochem Biophys*, 254 (1), pp. 124-35.

Hecker, M. & Ullrich, V. (1989). 'On the mechanism of prostacyclin and thromboxane a2 biosynthesis' *J Biol Chem*, 264 (1), pp. 141-50.

Herschman, H. R. (1994). 'Regulation of prostaglandin synthase-1 and prostaglandin synthase-2' *Cancer Metastasis Rev*, 13 (3-4), pp. 241-56.

Hiratsuka, A., et al. (1997). 'Subunit ya-specific glutathione peroxidase activity toward cholesterol 7-hydroperoxides of glutathione s-transferases in cytosols from rat liver and skin' *J Biol Chem*, 272 (8), pp. 4763-9.

Izumi, T., Hoshiko, S., Radmark, O. & Samuelsson, B. (1990). 'Cloning of the cdna for human 12-lipoxygenase' *Proc Natl Acad Sci U S A*, 87 (19), pp. 7477-81.

Jacquot, C., et al. (2008). 'Synthesis of 11-thialinoleic acid and 14-thialinoleic acid, inhibitors of soybean and human lipoxygenases' *Organic & biomolecular chemistry*, 6 (22), pp. 4242-4252. DOI: 10.1039/b808003h Available at: https://www.ncbi.nlm.nih.gov/pubmed/18972057

https://www.ncbi.nlm.nih.gov/pmc/PMC2883171/.

Jacquot, C., et al. (2008). 'Isotope sensitive branching and kinetic isotope effects in the reaction of deuterated arachidonic acids with human 12- and 15-lipoxygenases' *Biochemistry*, 47 (27), pp. 7295-303. DOI: 10.1021/bi800308q.

Kabututu, Z., et al. (2009). 'Prostaglandin f2alpha synthase activities of aldo-keto reductase 1b1, 1b3 and 1b7' *J Biochem*, 145 (2), pp. 161-8. DOI: 10.1093/jb/mvn152.

Kargman, S. L., et al. (1995). 'Expression of prostaglandin g/h synthase-1 and -2 protein in human colon cancer' *Cancer Res*, 55 (12), pp. 2556-9.

Kiezel-Tsugunova, M. (2017). *Elucidating the metabolism of n-3 polyunsaturated fatty acids and formation of bioactive lipid mediators in human skin*. Unpublished thesis, The University of Manchester, Manchester.

Kim, M.-S., et al. (2014). 'A draft map of the human proteome' *Nature*, 509 p. 575. DOI: 10.1038/nature13302

https://www.nature.com/articles/nature13302#supplementary-information Available at: https://doi.org/10.1038/nature13302.

Kim, S. F., Huri, D. A. & Snyder, S. H. (2005). 'Inducible nitric oxide synthase binds, s-nitrosylates, and activates cyclooxygenase-2' *Science*, 310 (5756), pp. 1966-70. DOI: 10.1126/science.1119407.

Kobayashi, T., et al. (2004). 'Regulation of cytosolic prostaglandin e synthase by phosphorylation' *Biochem J*, 381 (Pt 1), pp. 59-69. DOI: 10.1042/bj20040118.

Kujubu, D. A., Reddy, S. T., Fletcher, B. S. & Herschman, H. R. (1993). 'Expression of the protein product of the prostaglandin synthase-2/tis10 gene in mitogen-stimulated swiss 3t3 cells' *J Biol Chem*, 268 (8), pp. 5425-30.

Lagarde, M., Croset, M., Authi, K. S. & Crawford, N. (1984). 'Subcellular localization and some properties of lipoxygenase activity in human blood platelets' *Biochem J*, 222 (2), pp. 495-500.

Lee, S. H., et al. (1992). 'Selective expression of mitogen-inducible cyclooxygenase in macrophages stimulated with lipopolysaccharide' *J Biol Chem*, 267 (36), pp. 25934-8.

Lim, S. C., et al. (2010). 'Impacts of cytosolic phospholipase a2, 15-prostaglandin dehydrogenase, and cyclooxygenase-2 expressions on tumor progression in colorectal cancer' *Yonsei Med J*, 51 (5), pp. 692-9. DOI: 10.3349/ymj.2010.51.5.692.

Liu, L., et al. (2008). 'Functional mimicry of the active site of glutathione peroxidase by glutathione imprinted selenium-containing protein' *Biomacromolecules*, 9 (1), pp. 363-8. DOI: 10.1021/bm7008312.

Moriuchi, H., et al. (2008). 'Molecular characterization of a novel type of prostamide/prostaglandin f synthase, belonging to the thioredoxin-like superfamily' *J Biol Chem*, 283 (2), pp. 792-801. DOI: 10.1074/jbc.M705638200.

Mueller, M. J., et al. (1996). 'Leukotriene a4 hydrolase: Protection from mechanism-based inactivation by mutation of tyrosine-378' *Proc Natl Acad Sci U S A*, 93 (12), pp. 5931-5.

Mukherjee, A., Brinkley, D. W., Chang, K. M. & Roth, J. P. (2007). 'Molecular oxygen dependent steps in fatty acid oxidation by cyclooxygenase-1' *Biochemistry*, 46 (13), pp. 3975-89. DOI: 10.1021/bi602502j.

Mulliez, E., et al. (1987). '5-lipoxygenase from potato tubers. Improved purification and physicochemical characteristics' *Biochim Biophys Acta Protein Struct Molec Enzym*, 916 (1), pp. 13-23. DOI: https://doi.org/10.1016/0167-4838(87)90205-6 Available at: http://www.sciencedirect.com/science/article/pii/0167483887902056.

Myung, S. J., et al. (2006). '15-hydroxyprostaglandin dehydrogenase is an in vivo suppressor of colon tumorigenesis' *Proc Natl Acad Sci U S A*, 103 (32), pp. 12098-102. DOI: 10.1073/pnas.0603235103.

Niegowski, D., et al. (2013). 'Crystal structures of leukotriene c4 synthase in complex with product analogs, implications for the enzyme mechanism' *J Biol Chem*. DOI: 10.1074/jbc.M113.534628 Available at: http://www.jbc.org/content/early/2013/12/23/jbc.M113.534628.abstract.

Niesen, F. H., et al. (2010). 'High-affinity inhibitors of human nad+-dependent 15-hydroxyprostaglandin dehydrogenase: Mechanisms of inhibition and structure-activity relationships' *PLOS ONE*, 5 (11), p. e13719. DOI: 10.1371/journal.pone.0013719 Available at: https://doi.org/10.1371/journal.pone.0013719.

Noreen, Y., et al. (1998). 'Development of a radiochemical cyclooxygenase-1 and -2 in vitro assay for identification of natural products as inhibitors of prostaglandin biosynthesis' *J Nat Prod (Gorakhpur)*, 61 (1), pp. 2-7. DOI: 10.1021/np970343j Available at: https://doi.org/10.1021/np970343j.

Nusing, R., Schneider-Voss, S. & Ullrich, V. (1990). 'Immunoaffinity purification of human thromboxane synthase' *Arch Biochem Biophys*, 280 (2), pp. 325-30.

Orlandi, M., et al. (1994). 'Thromboxane a2 synthase activity in platelet free human monocytes' *Biochim Biophys Acta*, 1215 (3), pp. 285-90.

Patel P., C. C., Anumolu J. R., Erlemann K. R., Grant G. E., Rokach J., Powell W. S. (2009). 'Substrate selectivity of 5-hydroxyeicosanoid dehydrogenase and its inhibition by 5-hydroxy-omaga-6-long-chain fatty acids' *J Pharmacol Exp Ther*, 329 (1), pp. 335-341.

Pettersson, P. L., Thoren, S. & Jakobsson, P. J. (2005). 'Human microsomal prostaglandin e synthase 1: A member of the mapeg protein superfamily' *Methods Enzymol*, 401 pp. 147-61. DOI: 10.1016/s0076-6879(05)01009-8.

Pinzar, E., et al. (2000). 'Structural basis of hematopoietic prostaglandin d synthase activity elucidated by site-directed mutagenesis' *J Biol Chem*, 275 (40), pp. 31239-44. DOI: 10.1074/jbc.M000750200.

Powell, W. S., Gravelle, F. & Gravel, S. (1992). 'Metabolism of 5(s)-hydroxy-6,8,11,14-eicosatetraenoic acid and other 5(s)-hydroxyeicosanoids by a specific dehydrogenase in human polymorphonuclear leukocytes' *J Biol Chem*, 267 (27), pp. 19233-41.

Radmark, O., Shimizu, T., Jornvall, H. & Samuelsson, B. (1984). 'Leukotriene a4 hydrolase in human leukocytes. Purification and properties' *J Biol Chem*, 259 (20), pp. 12339-45.

Richards, K. M. & Marnett, L. J. (1997). 'Leukocyte 12-lipoxygenase: Expression, purification, and investigation of the role of methionine residues in turnover-dependent inactivation and 5,8,11,14-eicosatetraynoic acid inhibition' *Biochemistry*, 36 (22), pp. 6692-6699. DOI: 10.1021/bi963051a Available at: https://doi.org/10.1021/bi963051a.

Rinaldo-Matthis, A., et al. (2010). 'Arginine 104 is a key catalytic residue in leukotriene c4 synthase' *J Biol Chem*, 285 (52), pp. 40771-40776. DOI: 10.1074/jbc.M110.105940 Available at: http://www.jbc.org/content/285/52/40771.abstract.

Ristimaki, A., et al. (1994). 'Induction of cyclooxygenase-2 by interleukin-1 alpha. Evidence for post-transcriptional regulation' *J Biol Chem*, 269 (16), pp. 11769-75.

Rogge, C. E., et al. (2004). 'Identification of tyr504 as an alternative tyrosyl radical site in human prostaglandin h synthase-2' *Biochemistry*, 43 (6), pp. 1560-1568. DOI: 10.1021/bi035717o Available at: https://doi.org/10.1021/bi035717o.

Romano, M., et al. (1993). 'Lipoxin synthase activity of human platelet 12-lipoxygenase' *Biochem J*, 296 ( Pt 1) pp. 127-33.

Rudberg, P. C., Tholander, F., Thunnissen, M. M. & Haeggstrom, J. Z. (2002). 'Leukotriene a4 hydrolase/aminopeptidase. Glutamate 271 is a catalytic residue with specific roles in two distinct enzyme mechanisms' *J Biol Chem*, 277 (2), pp. 1398-404. DOI: 10.1074/jbc.M106577200.

Schwartzman, M. L., et al. (1987). '12(r)-hydroxyicosatetraenoic acid: A cytochrome-p450-dependent arachidonate metabolite that inhibits na+,k+-atpase in the cornea' *Proc Natl Acad Sci U S A*, 84 (22), pp. 8125-8129. DOI: 10.1073/pnas.84.22.8125 Available at: https://www.pnas.org/content/pnas/84/22/8125.full.pdf.

Shimizu, T., Radmark, O. & Samuelsson, B. (1984). 'Enzyme with dual lipoxygenase activities catalyzes leukotriene a4 synthesis from arachidonic acid' *Proc Natl Acad Sci U S A*, 81 (3), pp. 689-93.

Shirumalla, R. K., et al. (2006). 'Rbx 7,796: A novel inhibitor of 5-lipoxygenase' *Inflamm Res*, 55 (12), pp. 517-27. DOI: 10.1007/s00011-006-6032-z.

Sloane, D. L., et al. (1995). 'Conversion of human 15-lipoxygenase to an efficient 12-lipoxygenase: The side-chain geometry of amino acids 417 and 418 determine positional specificity' *Protein Eng*, 8 (3), pp. 275-82.

Soberman, R. J. (1988). '5- and 15(omega-6)-lipoxygenases from human polymorphonuclear leukocytes' *Methods Enzymol*, 163 pp. 344-9.

Soberman, R. J., et al. (1985). 'Characterization and separation of the arachidonic acid 5-lipoxygenase and linoleic acid omega-6 lipoxygenase (arachidonic acid 15-lipoxygenase) of human polymorphonuclear leukocytes' *J Biol Chem*, 260 (7), pp. 4508-15.

Steinhilber, D. (2016). *Lipoxygenases in inflammation*. Frankfurt, Germany: Springer.

Stromberg-Kull, F. & Haeggstrom, J. Z. (1998). 'Purification and characterization of leukotriene a4 hydrolase from xenopus laevis oocytes' *FEBS Lett*, 433 (3), pp. 219-22.

Urade, Y., Fujimoto, N. & Hayaishi, O. (1985). 'Purification and characterization of rat brain prostaglandin d synthetase' *J Biol Chem*, 260 (23), pp. 12410-5.

Vecchio, A. J., Simmons, D. M. & Malkowski, M. G. (2010). 'Structural basis of fatty acid substrate binding to cyclooxygenase-2' *J Biol Chem*, 285 (29), pp. 22152-63. DOI: 10.1074/jbc.M110.119867.

Watanabe, K., et al. (1994). 'Identification of beta-trace as prostaglandin d synthase' *Biochem Biophys Res Commun*, 203 (2), pp. 1110-6. DOI: 10.1006/bbrc.1994.2297.

Wecksler, A. T., Jacquot, C., van der Donk, W. A. & Holman, T. R. (2009). 'Mechanistic investigations of human reticulocyte 15- and platelet 12-lipoxygenases with arachidonic acid' *Biochemistry*, 48 (26), pp. 6259-6267. DOI: 10.1021/bi802332j Available at: https://www.ncbi.nlm.nih.gov/pubmed/19469483

https://www.ncbi.nlm.nih.gov/pmc/PMC2935259/.

Wecksler, A. T., et al. (2009). 'Kinetic and structural investigations of the allosteric site in human epithelial 15-lipoxygenase-2' *Biochemistry*, 48 (36), pp. 8721-30. DOI: 10.1021/bi9009242.

Wei, C. (2012). *The role of 15-lipoxygenase-1- and cyclooxygenase-2-derived lipid mediators in endothelial cell proliferation*. Doctor of Philosophy (PhD), University of Pennsylvania, Pennsylvania.

Wilhelm, M., et al. (2014). 'Mass-spectrometry-based draft of the human proteome' *Nature*, 509 p. 582. DOI: 10.1038/nature13319

https://www.nature.com/articles/nature13319#supplementary-information Available at: https://doi.org/10.1038/nature13319.

Wu, Y. H., et al. (2008). 'Structural basis for catalytic and inhibitory mechanisms of human prostaglandin reductase ptgr2' *Structure*, 16 (11), pp. 1714-23. DOI: 10.1016/j.str.2008.09.007.

Yeh, H. C., et al. (2005). 'Characterization of heme environment and mechanism of peroxide bond cleavage in human prostacyclin synthase' *Biochim Biophys Acta*, 1738 (1-3), pp. 121-32. DOI: 10.1016/j.bbalip.2005.11.007.

Zheng, K., et al. (2008). 'A novel selenium-containing glutathione transferase zeta1-1, the activity of which surpasses the level of some native glutathione peroxidases' *Int J Biochem Cell Biol*, 40 (10), pp. 2090-7. DOI: 10.1016/j.biocel.2008.02.006.

Zhou, H., Yan, F. & Tai, H. H. (2001). 'C-terminal region of human nad+-dependent 15-hydroxyprostaglandin dehydrogenase is involved in the interaction with prostaglandin substrates' *Eur J Biochem*, 268 (12), pp. 3368-74.

Zhou, Y., et al. (2010). 'Structure-function analysis of human l-prostaglandin d synthase bound with fatty acid molecules' *Faseb j*, 24 (12), pp. 4668-77. DOI: 10.1096/fj.10-164863.

## Supplementary Document S8. Non-enzymatic Reaction Structure and Parameterisation.

Documentation of parameter values obtained for all non-enzymatic reactions in the model (Reactions 6–9, 96–99; Supplementary Table S3) from the literature and associated uncertainty for the eicosanoid network model. Parameterisation was performed using the method of Tsigkinopoulou *et al.,* (2018). The table includes information regarding each reaction and its respective parameters are documented. This includes information such as the reaction rate law and the literature values that were used to define parameters, including experimental conditions, total weights and literature references from which the data were obtained. In this model some parameters are referred as “Dependent parameters”, meaning that the log-normal distribution for that parameter was calculated using multivariate distributions (discussed in **Section 2.6.2**). As a result, no confidence interval factor or literature values were cited for the Dependent parameters.

**Contents**

**S.8.1 Reaction 6: $TXA_2 \rightleftharpoons TXB_2$ .......... 238**

**S.8.2. Reaction 7: $PGI_2 \rightleftharpoons$ 6-keto-$PGF_{1\alpha}$ .......... 247**

**S.8.3. Reaction 8: $PGD_2 \rightleftharpoons PGJ_2$ .......... 256**

**S.8.4. Reaction 9: $PGJ_2 \rightleftharpoons$ 15-deoxy-$PGJ_2$ .......... 265**

**S.8.5. Reaction 96: ex$TXA_2 \rightleftharpoons$ ex$TXB_2$ .......... 274**

**S.8.6. Reaction 97: ex$PGI_2 \rightleftharpoons$ ex6-keto-$PGF_{1\alpha}$ .......... 283**

**S.8.7. Reaction 98: ex$PGD_2 \rightleftharpoons$ ex$PGJ_2$ .......... 292**

**S.8.8. Reaction 99: ex$PGJ_2 \rightleftharpoons$ ex15-deoxy-$PGJ_2$ .......... 301**

**References .......... 310**

**S.8.1 Reaction 6: $TXA_2 \rightleftharpoons TXB_2$**

Upon generating the unstable metabolite $TXA_2$, it is rapidly hydrolysed into $TXB_2$ via a non-enzymatic reaction. This inactive metabolite is produced by the incorporation of two hydrogens and one oxygen at C9 and C11, resulting in the opening of the trimethylene oxide ring, and the generation of two hydroxyl groups on the tetrahydropyran ring.

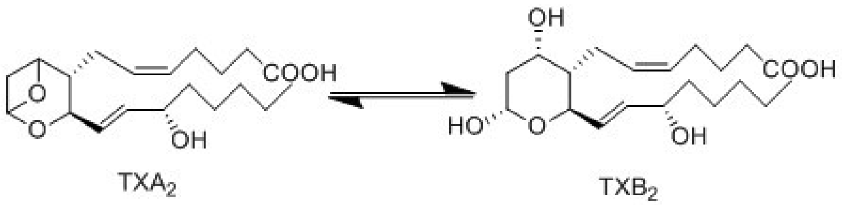


**Figure SF.8.11.** The non-enzymatic hydrolysis reaction of thromboxane $A_2$ ($TXA_2$) into thromboxane $B_2$ ($TXB_2$) (Reaction 6).

**Equation SEq.8.2.** Reaction rate law for Reaction 6.

$$v_6 = K_f[TXA_2] - K_r[TXB_2]$$

### S.8.1.1. Reaction 6 parameters

#### S.8.1.1.1. Parameter: $K_f$

Parameter values for the $K_f$ of Reaction 6 were obtained from the literature and summarised in Table ST.8.1.1.1.1. The log-normal distribution properties for the $K_f$ of Reaction 6 are shown in Table ST.8.1.1.1.2 and plotted in Figure SF.8.1.1.1.1.

**Table ST.8.1.1.1.1.** Literature information used to design the Reaction 6 $K_f$ parameter distribution. Each value was assigned a weight using the protocol described in Section 2.6.2. The type of error was assigned as 0 if it was additive and 1 if it was multiplicative.

| Value ($M^{-1}$ $s^{-1}$) | Error ($M^{-1}$ $s^{-1}$) | Experimental details | | | | Weight | Type of error | Reference |
|---|---|---|---|---|---|---|---|---|
| | | **Substrate type** | **Functional group** | **Temperature (°C)** | **Other** | | | |
| 3.70 $x10^3$ | 1.00 $x10^2$ | Different cycle | Epoxide | 25 | | **32** | | (Ross et al., 1982) |
| 8.70 $x10^3$ | | Different cycle | Epoxide | 25 | | **32** | | (Ross et al., 1982) |
| 1.10 $x10^4$ | 1.00 $x10^3$ | 6 ring cycle | Epoxide | 25 | | **48** | | (Ross et al., 1982) |
| 2.40 $x10^4$ | | 6 ring cycle | Epoxide | 25 | | **48** | | (Ross et al., 1982) |
| 3.70 $x10^3$ | 1.00 $x10^2$ | Different cycle | Epoxide | 25 | | **32** | | (Ross et al., 1982) |
| 3.60 | 2.00 $x10^{-1}$ | Different cycle | Epoxide | 25 | | **32** | | (Ross et al., 1982) |
| 1.70 | 1.00 $x10^{-1}$ | Different cycle | Epoxide | 25 | | **32** | | (Ross et al., 1982) |

| | | | | | | | | |
|---|---|---|---|---|---|---|---|---|
| 2.67 x10$^{1}$ | 9.00 x10$^{-1}$ | Different cycle | Epoxide | 25 | | **32** | | (Ross et al., 1982) |
| 3.50 x10$^{1}$ | | Different cycle | Epoxide | 25 | | **32** | | (Ross et al., 1982) |

**Table ST.8.1.1.1.2.** The log-normal distribution properties of the Reaction 6 $K_f$ distribution. These values were calculated using the functions described in Section 2.6.2.

| **Mode ($M^{-1}$ $s^{-1}$)** | **Confidence Interval** | **Location parameter (μ)** | **Scale parameter (σ)** |
|---|---|---|---|
| 3.70 x10$^{3}$ | 3.44 x10$^{1}$ | 1.10 x10$^{1}$ | 1.66 |

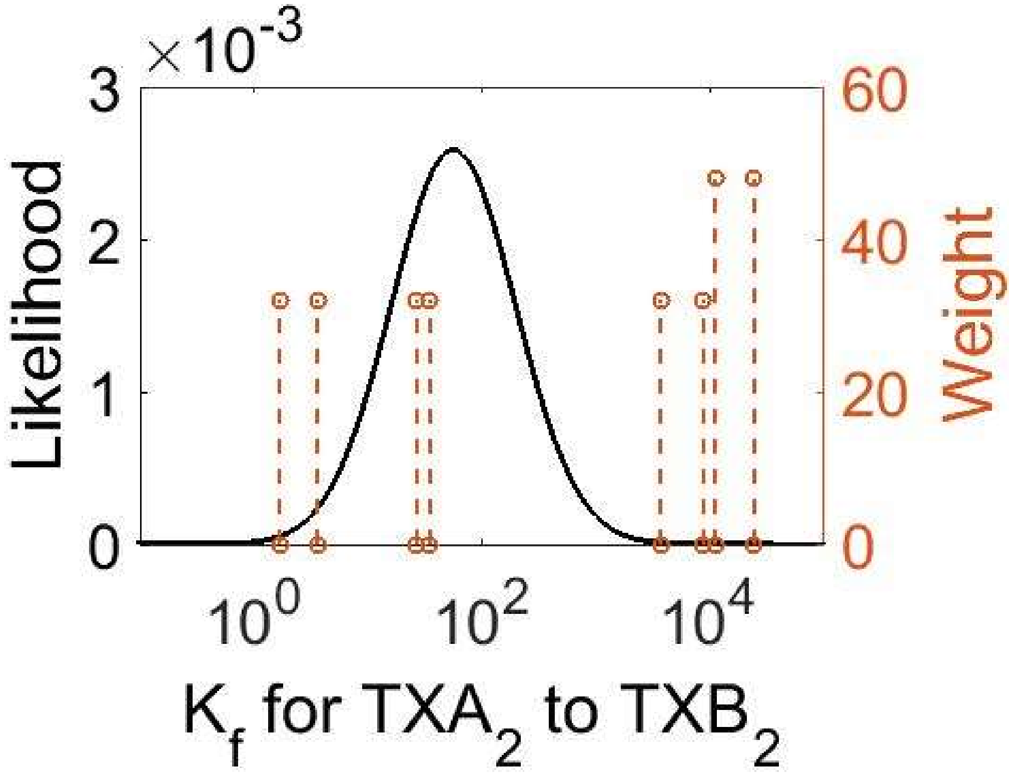


**Figure SF.8.1.1.1.1.** The estimated probability distribution for the Reaction 6 $K_f$. The value and weight of the literature values used to define the distribution are indicated by an orange dashed line. The x axis is plotted on a log-scale.

### S.8.1.1.2. Parameter: $K_D$

Parameter values for the $K_D$ of Reaction 6 were obtained from the literature and summarised in Table ST.8.1.1.2.1. The log-normal distribution properties for the $K_D$ of Reaction 6 are shown in Table ST.8.1.1.2.2 and plotted in Figure SF.8.1.1.2.1.

**Table ST.8.1.1.2.1.** Literature information used to design the Reaction 6 $K_D$ parameter distribution. Each value was assigned a weight using the protocol described in Section 2.6.2. The type of error was assigned as 0 if it was additive and 1 if it was multiplicative.

| **Value ($M^{-1}$ $min^{-1}$)** | **Error ($M^{-1}$ $min^{-1}$)** | **Experimental details** | | | | **Weight** | **Type of error** | **Reference** |
|---|---|---|---|---|---|---|---|---|
| | | **Species** | **Substrate type** | **Temperature (°C)** | **Other** | | | |
| 0.000038 | NaN | Mosquito | Carbocyclic TXA2 | 35 | | **24** | | (Alvarenga et al., 2010) |
| 0.00000023 | NaN | Rabbit | TXA2 antagonist | 35 | | **36** | | (Nakahata et al., 1992) |
| 1500 | 500 | N/A | Hydroxysulfamic acid | 25 | | **16** | | (D. LITTLEJOHN, 1988) |
| 1.75 $x10^{-12}$ | NaN | N/A | H2O2 | | | **16** | | (D. LITTLEJOHN, 1988) |
| 5.90 $x10^{-8}$ | NaN | N/A | Oxaplatin | | | **16** | | (Jerremalm et al., 2003) |

**Table ST.8.1.1.2.2.** The log-normal distribution properties of the Reaction 6 $K_D$ distribution. These values were calculated using the functions described in Section 2.6.2.

| **Mode** ($M^{-1}$ $min^{-1}$) | **Confidence Interval** | **Location parameter (μ)** | **Scale parameter (σ)** |
|---|---|---|---|
| 2.35 x$10^{-7}$ | 1.76 x$10^{4}$ | -6.86 | 2.90 |

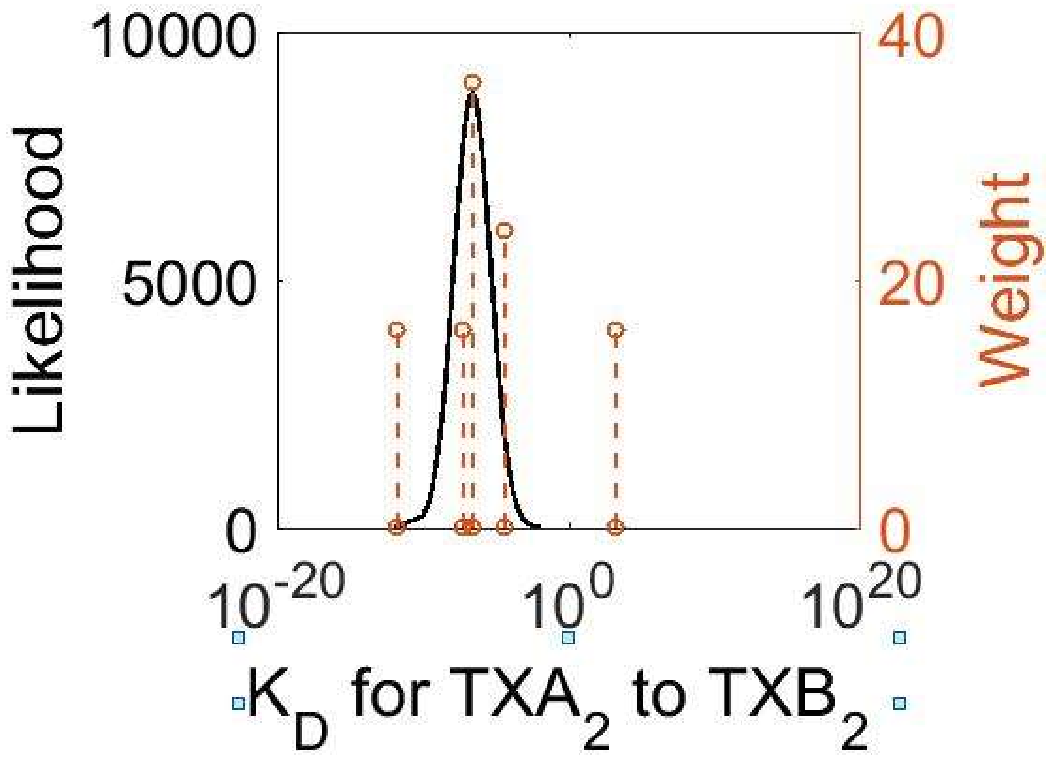


**Figure SF.8.1.1.2.1.** The estimated probability distribution for the Reaction 6 $K_D$. The value and weight of the literature values used to define the distribution are indicated by an orange dashed line. The x axis is plotted on a log-scale.

**S.8.1.1.3. Parameter: $K_r$ (Dependent parameter)**

The log-normal distribution for the parameter for the $K_r$ of Reaction 6 was calculated using multivariate distributions. The log-normal distribution properties for the $K_r$ of Reaction 6 are shown in Table ST.8.1.1.3.1 and plotted in Figure SF.8.1.1.3.1.

**Table ST.8.1.1.3.1.** The log-normal distribution properties of the Reaction 6 $K_r$ distribution. These values were calculated using the functions described in Section 2.6.2.

| **Mode ($M^{-1}$ $min^{-1}$)** | **Location parameter (μ)** | **Scale parameter (σ)** |
|---|---|---|
| $5.28 \times 10^{1}$ | 5.63 | 1.29 |

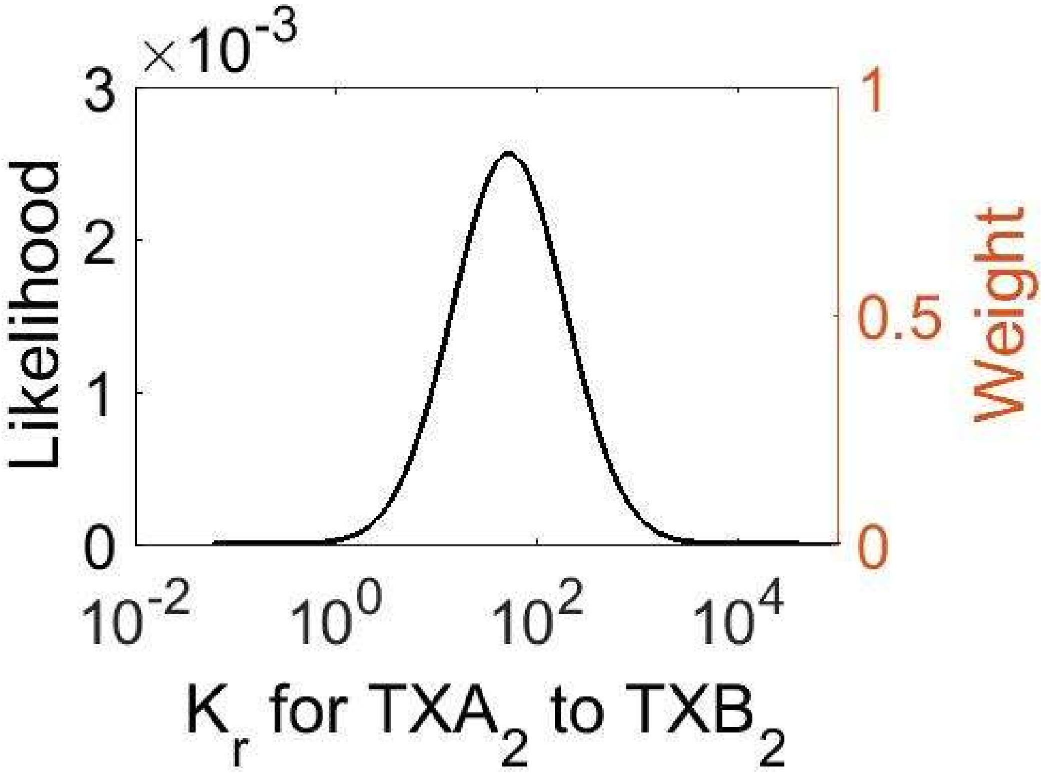


**Figure SF.8.1.1.3.1.** The estimated probability distribution Reaction 6 $K_r$. The value and weight of the literature values used to define the distribution are indicated by an orange dashed line. The x axis is plotted on a log-scale.

**S.8.2. Reaction 7: $PGI_2 \rightleftharpoons$ 6-keto-$PGF_{1\alpha}$**

Due to ring strain, $PGI_2$ is rapidly hydrolysed to 6-keto-$PGF_{1\alpha}$. This is a non-enzymatic reaction and results in the formation of a hydroxyl group at C9 and a ketone at C6, by incorporating two hydrogens and one oxygen.

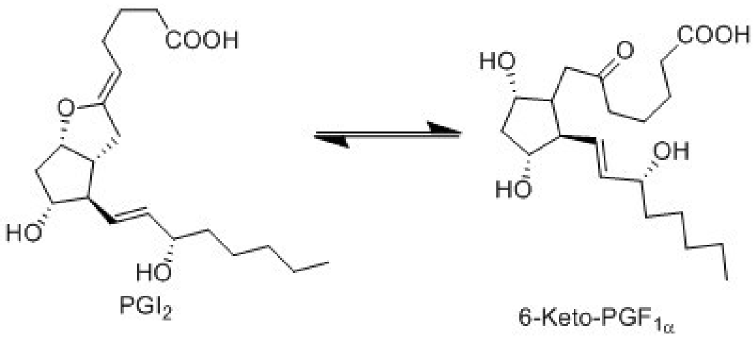


**Figure SF.8.2.** The non-enzymatic transformation of prostaglandin $I_2$ ($PGI_2$) into 6-keto-prostaglandin $F_{1\alpha}$ (6-keto-$PGF_{1\alpha}$) (Reaction 7).

**Equation Seq.8.2.** Reaction rate law for Reaction 7.

$$\mathbf{\nu_7 = K_f[PGI_2] - K_r[6 - keto - PGF_{1\alpha}]}$$

### S.8.2.1. Reaction parameters

#### S.8.2.1.1. Parameter: $K_f$

Parameter values for the $K_f$ of Reaction 7 were obtained from the literature and summarised in Table ST.8.2.1.1.1. The log-normal distribution properties for the $K_f$ of Reaction 7 are shown in Table ST.8.2.1.1.2 and plotted in Figure SF.8.2.1.1.1.

**Table ST.8.2.1.1.1.** Literature information used to design the Reaction 7 $K_f$ parameter distribution. Each value was assigned a weight using the protocol described in Section 2.6.2. The type of error was assigned as 0 if it was additive and 1 if it was multiplicative.

| Value ($M^{-1}$ $s^{-1}$) | Error ($M^{-1}$ $s^{-1}$) | Experimental details | | | | Weight | Type of error | Reference |
|---|---|---|---|---|---|---|---|---|
| | | Substrate type | Functional group | Temperature (°C) | Other | | | |
| 3.70 $x10^3$ | 1.00 $x10^2$ | Different cycle | Epoxide | 25 | | **32** | | (Ross et al., 1982) |
| 8.70 $x10^3$ | | Different cycle | Epoxide | 25 | | **32** | | (Ross et al., 1982) |
| 1.10 $x10^4$ | 1.00 $x10^3$ | 6 ring cycle | Epoxide | 25 | | **48** | | (Ross et al., 1982) |
| 2.40 $x10^4$ | | 6 ring cycle | Epoxide | 25 | | **48** | | (Ross et al., 1982) |
| 3.70 $x10^3$ | 1.00 $x10^2$ | Different cycle | Epoxide | 25 | | **32** | | (Ross et al., 1982) |
| 3.60 | 2.00 $x10^{-1}$ | Different cycle | Epoxide | 25 | | **32** | | (Ross et al., 1982) |

| | | | | | | | | |
|---|---|---|---|---|---|---|---|---|
| 1.70 | 1.00 $\times10^{-1}$ | Different cycle | Epoxide | 25 | | **32** | | (Ross et al., 1982) |
| 2.67 $\times10^{1}$ | 9.00 $\times10^{-1}$ | Different cycle | Epoxide | 25 | | **32** | | (Ross et al., 1982) |
| 3.50 $\times10^{1}$ | | Different cycle | Epoxide | 25 | | **32** | | (Ross et al., 1982) |

**Table ST.8.2.1.1.2.** The log-normal distribution properties of the Reaction 7 $K_f$ distribution. These values were calculated using the functions described in Section 2.6.2.

| **Mode ($M^{-1}$ $s^{-1}$)** | **Confidence Interval** | **Location parameter (μ)** | **Scale parameter (σ)** |
|---|---|---|---|
| 3.70 $\times10^{3}$ | 3.44 $\times10^{1}$ | 1.10 $\times10^{1}$ | 1.66 |

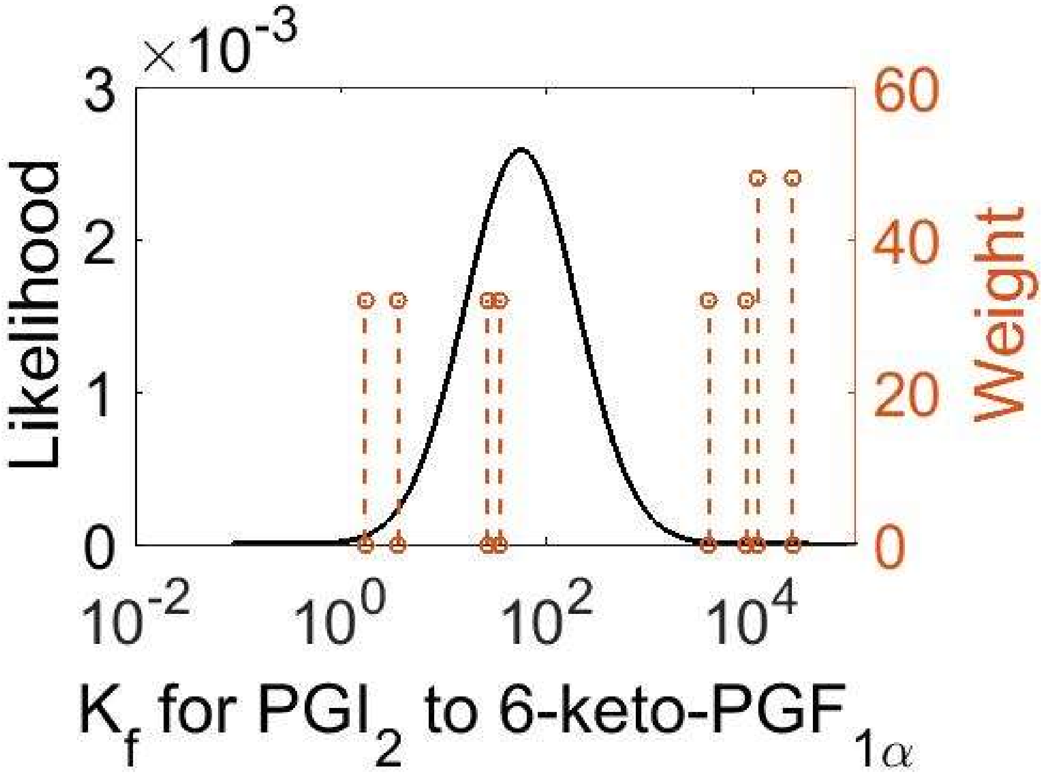


**Figure SF.8.2.1.1.1.** The estimated probability distribution for the Reaction 7 $K_f$. The value and weight of the literature values used to define the distribution are indicated by an orange dashed line. The x axis is plotted on a log-scale.

### S.8.2.1.2. Parameter: $K_D$

Parameter values for the $K_D$ of Reaction 7 were obtained from the literature and summarised in Table ST.8.2.1.2.1. The log-normal distribution properties for the $K_D$ of Reaction 7 are shown in Table ST.8.2.1.2.2 and plotted in Figure SF.8.2.1.2.1.

**Table ST.8.2.1.2.1.** Literature information used to design the Reaction 7 $K_D$ parameter distribution. Each value was assigned a weight using the protocol described in Section 2.6.2. The type of error was assigned as 0 if it was additive and 1 if it was multiplicative.

| **Value ($M^{-1}$ $min^{-1}$)** | **Error ($M^{-1}$ $min^{-1}$)** | **Experimental details** | | | | **Weight** | **Type of error** | **Reference** |
|---|---|---|---|---|---|---|---|---|
| | | **Species** | **Substrate type** | **Temperature (°C)** | **Other** | | | |
| 0.000038 | NaN | Mosquito | Carbocyclic TXA2 | 35 | | **24** | | (Alvarenga et al., 2010) |
| 0.00000023 | NaN | Rabbit | TXA2 antagonist | 35 | | **36** | | (Nakahata et al., 1992) |
| 1500 | 500 | N/A | Hydroxysulfamic acid | 25 | | **16** | | (D. LITTLEJOHN, 1988) |
| $1.75 \times 10^{-12}$ | NaN | N/A | H2O2 | | | **16** | | (D. LITTLEJOHN, 1988) |
| $5.90 \times 10^{-8}$ | NaN | N/A | Oxaplatin | | | **16** | | (Jerremalm et al., 2003) |

**Table ST.8.2.1.2.2.** The log-normal distribution properties of the Reaction 7 $K_D$ distribution. These values were calculated using the functions described in Section 2.6.2.

| **Mode ($M^{-1}$ $min^{-1}$)** | **Confidence Interval** | **Location parameter (μ)** | **Scale parameter (σ)** |
|---|---|---|---|
| 2.35 $x10^{-7}$ | 1.76 $x10^{4}$ | -6.86 | 2.90 |

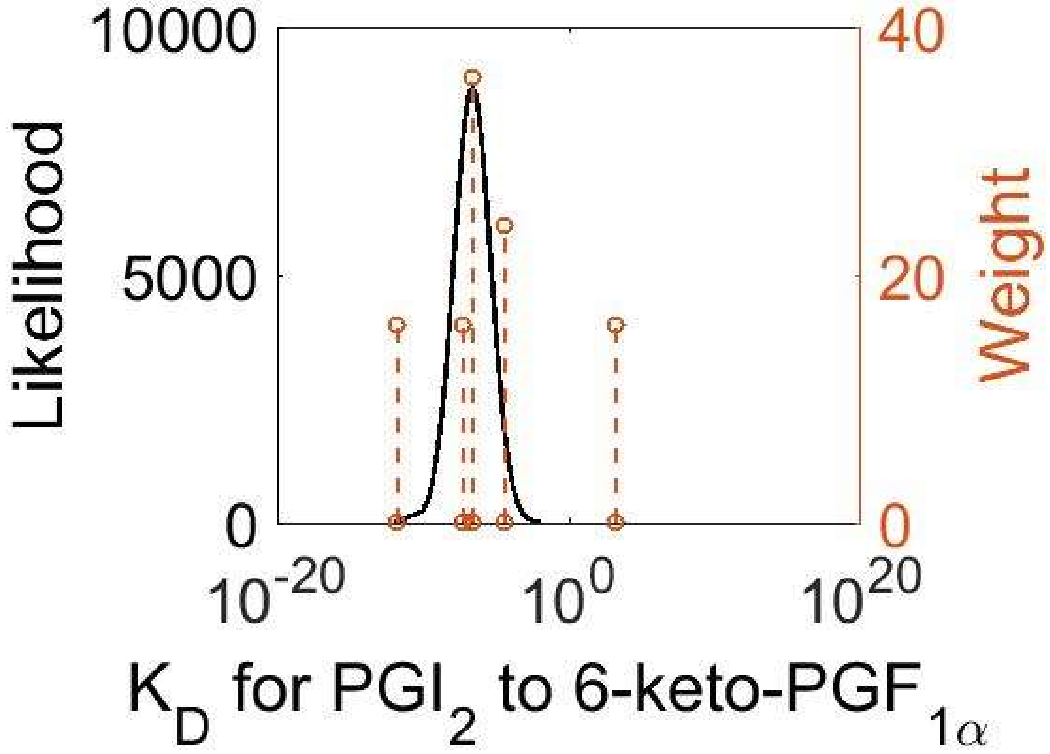


**Figure SF.8.2.1.2.1.** The estimated probability distribution for Reaction 7 $K_D$. The value and weight of the literature values used to define the distribution are indicated by an orange dashed line. The x axis is plotted on a log-scale.

**S.8.2.1.3. Parameter: $K_r$ (Dependent parameter)**

The log-normal distribution for the parameter for the $K_r$ of Reaction 7 was calculated using multivariate distributions. The log-normal distribution properties for the $K_r$ of Reaction 7 are shown in Table ST.8.2.1.3.1 and plotted in Figure SF.8.2.1.3.1.

**Table ST.8.2.1.3.1.** The log-normal distribution properties of the Reaction 7 $K_r$ distribution. These values were calculated using the functions described in Section 2.6.2.

| **Mode ($M^{-1}$ $min^{-1}$)** | **Location parameter (μ)** | **Scale parameter (σ)** |
|---|---|---|
| 5.81 $x10^1$ | 5.60 | 1.24 |

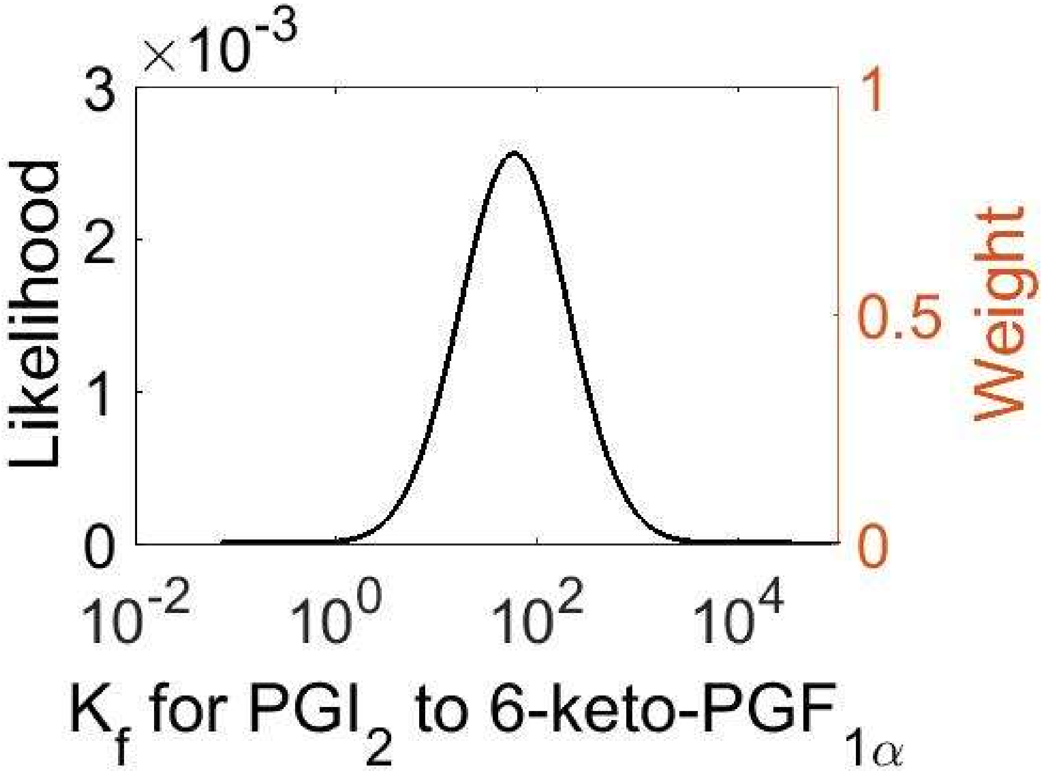


**Figure SF.8.2.1.3.1.** The estimated probability distribution for Reaction 7 $K_f$. The value and weight of the literature values used to define the distribution are indicated by an orange dashed line. The x axis is plotted on a log-scale.

### S.8.3. Reaction 8: $PGD_2 \rightleftharpoons PGJ_2$

$PGD_2$ is subsequently converted to $PGJ_2$ via a non-enzymatic reaction (Fitzpatrick and Wynalda, 1983). This dehydration reaction includes the removal of two hydrogens and one oxygen molecule, and occurs across the C9 hydroxyl and the C10 carbon axis, to form a double bond between C9 and 10.

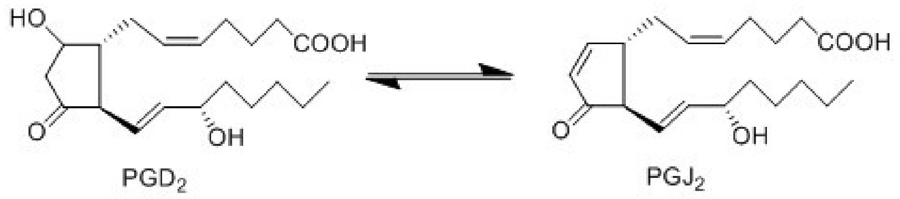


**Figure SF.8.2.** The non-enzymatic transformation of prostaglandin $D_2$ ($PGD_2$) into prostaglandin $J_2$ ($PGJ_2$) (Reaction 8).

**SEq.8.3.** Reaction rate law for Reaction 8.

$$\boldsymbol{v_8 = K_f[PGD_2] - K_r[PGJ_2]}$$

### S.8.3.1. Reaction parameters

#### S.8.3.1.1. Parameter: $K_f$

Parameter values for the $K_f$ of Reaction 8 were obtained from the literature and summarised in Table ST.8.3.1.1.1. The log-normal distribution properties for the $K_f$ of Reaction 8 are shown in Table ST.8.3.1.1.2 and plotted in Figure SF.8.3.1.1.1.

**Table ST.8.3.1.1.1.** Literature information used to design the Reaction 8 $K_f$ parameter distribution. Each value was assigned a weight using the protocol described in Section 2.6.2. The type of error was assigned as 0 if it was additive and 1 if it was multiplicative.

| Value ($M^{-1}$ $min^{-1}$) | Error ($M^{-1}$ $min^{-1}$) | Experimental details | | | | Weight | Type of error | Reference |
|---|---|---|---|---|---|---|---|---|
| | | Substrate type | Functional group | Temperature (°C) | Other | | | |
| 3.30 x$10^6$ | | Different | Carboxylic acid | 25 | | 32 | | (B. H. GIBBONS, 1963) |
| 4.50 | | Different | | 20 | | 32 | | (Tur'yan, 1998) |
| 4.20 x$10^{-1}$ | | Different | | 20 | | 32 | | (Ranney and Ziemann, 2016) |

**Table ST.8.3.1.1.2.** The log-normal distribution properties of the Reaction 8 $k_f$ distribution. These values were calculated using the functions described in Section 2.6.2.

| **Mode ($M^{-1}$ $min^{-1}$)** | **Confidence Interval** | **Location parameter (μ)** | **Scale parameter (σ)** |
|---|---|---|---|
| 4.48 | 1.09 $x10^{3}$ | 7.34 | 2.42 |

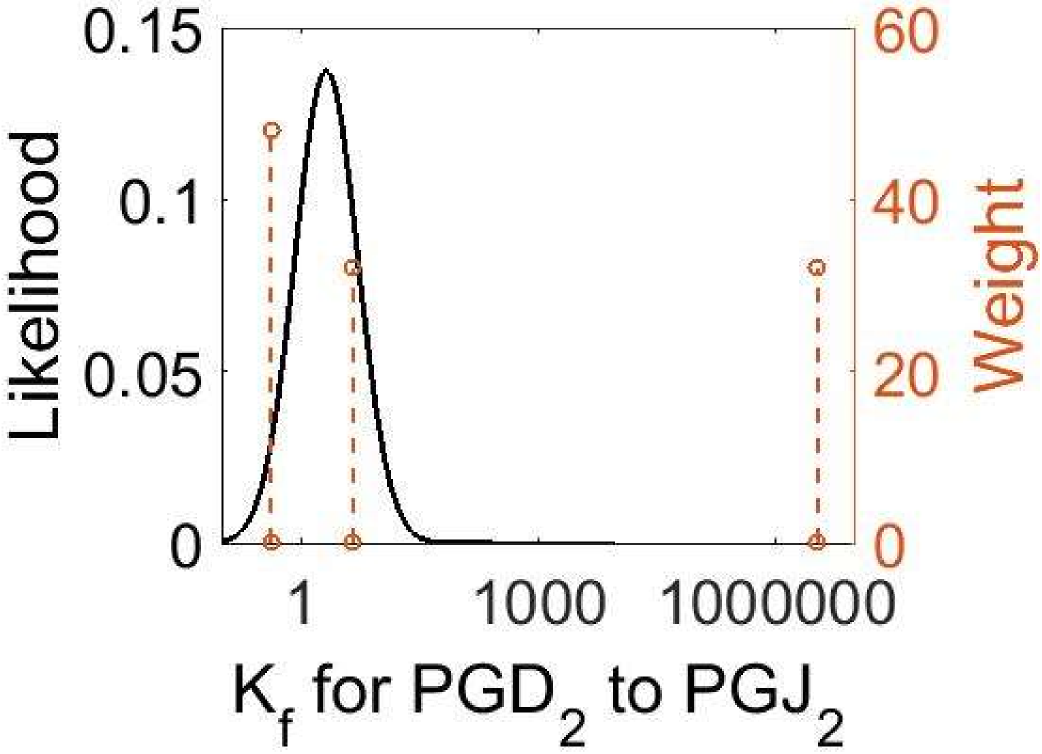


**Figure SF.8.3.1.1.1.** The estimated probability distribution for the Reaction 8 $K_f$. The value and weight of the literature values used to define the distribution are indicated by an orange dashed line. The x axis is plotted on a log-scale.

**S.8.3.1.2. Parameter: $K_D$**

Parameter values for the $K_D$ of Reaction 8 were obtained from the literature and summarised in Table ST.8.3.1.2.1. The log-normal distribution properties for the $K_D$ of Reaction 8 are shown in Table ST.8.3.1.2.2 and plotted in Figure SF.8.3.1.2.1.

**Table ST.8.3.1.2.1.** Literature information used to design the Reaction 8 $K_D$ parameter distribution. Each value was assigned a weight using the protocol described in Section 2.6.2. The type of error was assigned as 0 if it was additive and 1 if it was multiplicative.

| **Value ($M^{-1}$ $min^{-1}$)** | **Error ($M^{-1}$ $min^{-1}$)** | **Experimental details** | | | | **Weight** | **Type of error** | **Reference** |
|---|---|---|---|---|---|---|---|---|
| | | **Substrate** | **Functional group** | **Temperature (°C)** | **Other** | | | |
| 8.00 x$10^{-4}$ | NaN | OH radicals | tetrahydrofuran | Unknown | | **8** | | (Ranney and Ziemann, 2016) |
| 4.00 x$10^{-4}$ | NaN | H2CO3 | carbonate | | | **8** | | (Buytendyk et al., 1927) |
| 4.40 x$10^{-4}$ | NaN | H2CO3 | carbonate | | | **8** | | (Thiel and Strohecker, 1914) |

**Table ST.8.3.1.2.2.** The log-normal distribution properties of the Reaction 8 $K_D$ distribution. These values were calculated using the functions described in Section 2.6.2.

| **Mode** **($M^{-1}$ $min^{-1}$)** | **Confidence Interval** | **Location parameter (μ)** | **Scale parameter (σ)** |
|---|---|---|---|
| 4.49 $x10^{-4}$ | 1.38 | -7.61 | $3.07x10^{-1}$ |

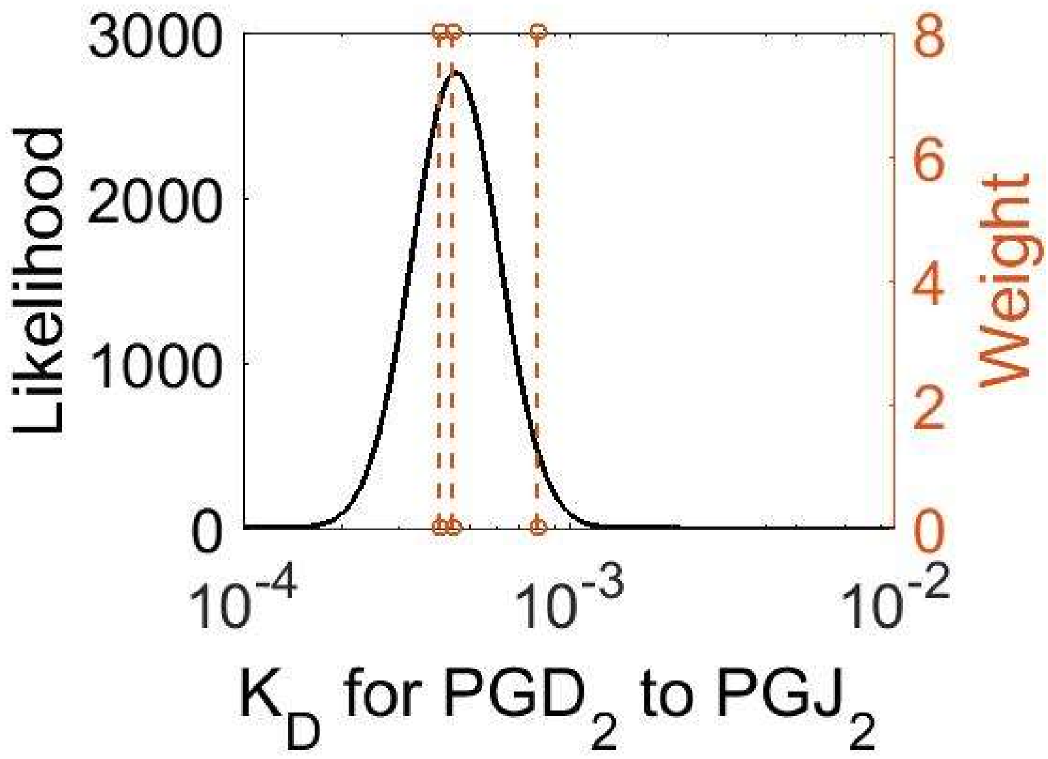


**Figure SF.8.3.1.2.1.** The estimated probability distribution for the Reaction 8 $K_D$. The value and weight of the literature values used to define the distribution are indicated by an orange dashed line. The x axis is plotted on a log-scale.

**S.8.3.1.3. Parameter: $K_r$ (Dependent parameter)**

The log-normal distribution for the parameter for the $K_r$ of Reaction 8 was calculated using multivariate distributions. The log-normal distribution properties for the $K_r$ of Reaction 6 are shown in Table ST.8.3.1.3.1 and plotted in Figure SF.8.3.1.3.1.

**Table ST.8.3.1.3.1.** The log-normal distribution properties of the Reaction 8 $K_r$ distribution. These values were calculated using the functions described in Section 2.6.2.

| Mode ($M^{-1}$ $min^{-1}$) | Location parameter (μ) | Scale parameter (σ) |
|---|---|---|
| 9.25 x$10^{-4}$ | -6.07 | 9.57 x$10^{-1}$ |

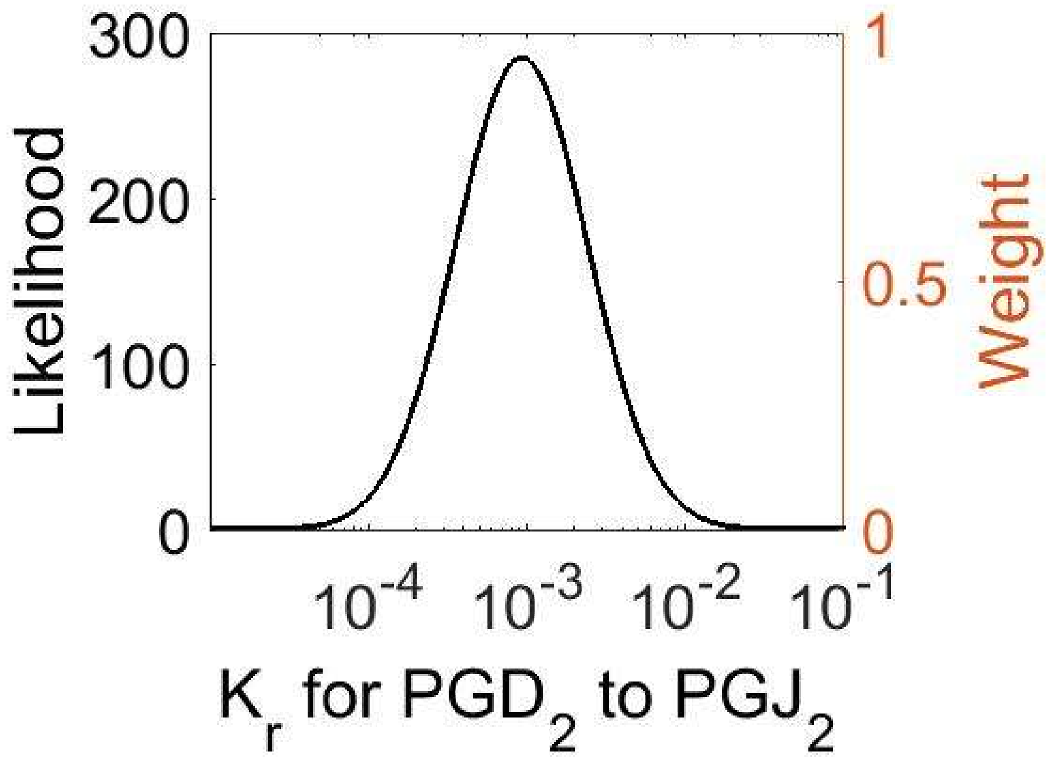


**Figure SF.8.3.1.3.1.** The estimated probability distribution for Reaction 8 $K_r$. The value and weight of the literature values used to define the distribution are indicated by an orange dashed line. The x axis is plotted on a log-scale.

### S.8.4. Reaction 9: $PGJ_2 \rightleftharpoons$ 15-deoxy-$PGJ_2$

$PGJ_2$ is subject to a non-enzymatic dehydration reaction to yield 15-deoxy-$PGJ_2$. This dehydration reaction includes the removal of two hydrogens and one oxygen molecule, and occurs across the C15 hydroxyl and the C14 carbon axis, to form a double bond between C15 and 14.

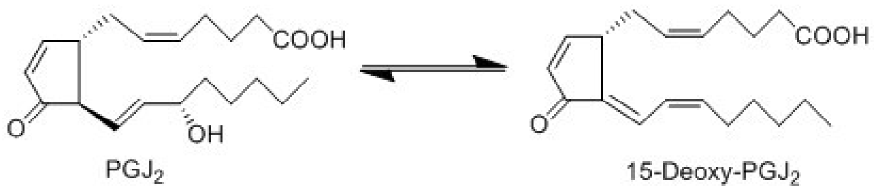


**Figure SF.8.4.** The non-enzymatic transformation of prostaglandin $J_2$ ($PGJ_2$) into 15-deoxy-prostaglandin $J_2$ (15-deoxy-$PGJ_2$) (Reaction 9).

**Equation SEq.8.4. Reaction rate law for Reaction 9.**

$$\boldsymbol{v_9 = K_f[PGJ_2] - K_r[15 - deoxy - PGJ_2]}$$

### S.8.4.1. Reaction parameters

#### S.8.4.1.1. Parameter: $K_f$

Parameter values for the $K_f$ of Reaction 9 were obtained from the literature and summarised in Table ST.8.4.1.1.1. The log-normal distribution properties for the $K_f$ of Reaction 9 are shown in Table ST.8.4.1.1.2 and plotted in Figure SF.8.4.1.1.1.

**Table ST.8.4.1.1.1.** Literature information used to design the Reaction 9 $K_f$ parameter distribution. Each value was assigned a weight using the protocol described in Section 2.6.2. The type of error was assigned as 0 if it was additive and 1 if it was multiplicative.

| Value ($M^{-1}$ $min^{-1}$) | Error ($M^{-1}$ $min^{-1}$) | Experimental details | | | | Weight | Type of error | Reference |
|---|---|---|---|---|---|---|---|---|
| | | Substrate type | Functional group | Temperature (°C) | Other | | | |
| 3.30 x$10^{6}$ | | Different | Carboxylic acid | 25 | | 32 | | (B. H. GIBBONS, 1963) |
| 4.50 | | Different | | 20 | | 32 | | (Tur'yan, 1998) |
| 4.20 x$10^{-1}$ | | Different | | 20 | | 32 | | (Ranney and Ziemann, 2016) |

**Table ST.8.4.1.1.2.** The log-normal distribution properties of the Reaction 9 $k_f$ distribution. These values were calculated using the functions described in Section 2.6.2.

| **Mode** ($M^{-1}$ $min^{-1}$) | **Confidence Interval** | **Location parameter (μ)** | **Scale parameter (σ)** |
|---|---|---|---|
| 4.48 | 1.09 $x10^3$ | 7.34 | 2.42 |

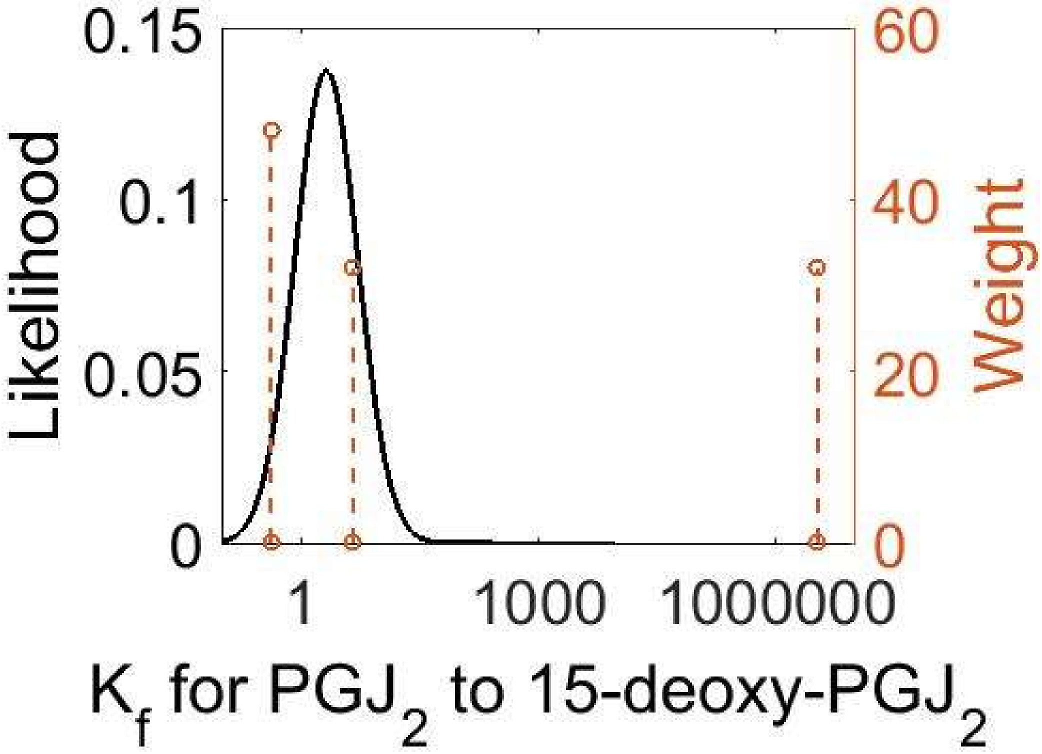


**Figure SF.8.4.1.1.1.** The estimated probability distribution for Reaction 9 $K_f$. The value and weight of the literature values used to define the distribution are indicated by an orange dashed line. The x axis is plotted on a log-scale.

### S.8.4.1.2 Parameter: $K_D$

Parameter values for the $K_D$ of Reaction 9 were obtained from the literature and summarised in Table ST.8.4.1.2.1. The log-normal distribution properties for the $K_D$ of Reaction 9 are shown in Table ST.8.4.1.2.2 and plotted in Figure SF.8.2.1.2.1.

**Table ST.8.4.1.2.1.** Literature information used to design the Reaction 9 $K_D$ parameter distribution. Each value was assigned a weight using the protocol described in Section 2.6.2. The type of error was assigned as 0 if it was additive and 1 if it was multiplicative.

| **Value ($M^{-1}$ $min^{-1}$)** | **Error ($M^{-1}$ $min^{-1}$)** | **Experimental details** | | | | **Weight** | **Type of error** | **Reference** |
|---|---|---|---|---|---|---|---|---|
| | | **Substrate** | **Functional group** | **Temperature (°C)** | **Other** | | | |
| 8.00 x$10^{-4}$ | NaN | OH radicals | tetrahydrofuran | Unknown | | **8** | | (Ranney and Ziemann, 2016) |
| 4.00 x$10^{-4}$ | NaN | H2CO3 | carbonate | | | **8** | | (Buytendyk et al., 1927) |
| 4.40 x$10^{-4}$ | NaN | H2CO3 | carbonate | | | **8** | | (Thiel and Strohecker, 1914) |

**Table ST.8.4.1.2.2.** The log-normal distribution properties of the Reaction 9 $K_D$ distribution. These values were calculated using the functions described in Section 2.6.2.

| **Mode ($M^{-1}$ $min^{-1}$)** | **Confidence Interval** | **Location parameter (μ)** | **Scale parameter (σ)** |
|---|---|---|---|
| $4.49\ x10^{-4}$ | 1.38 | -7.61 | $3.07x10^{-1}$ |

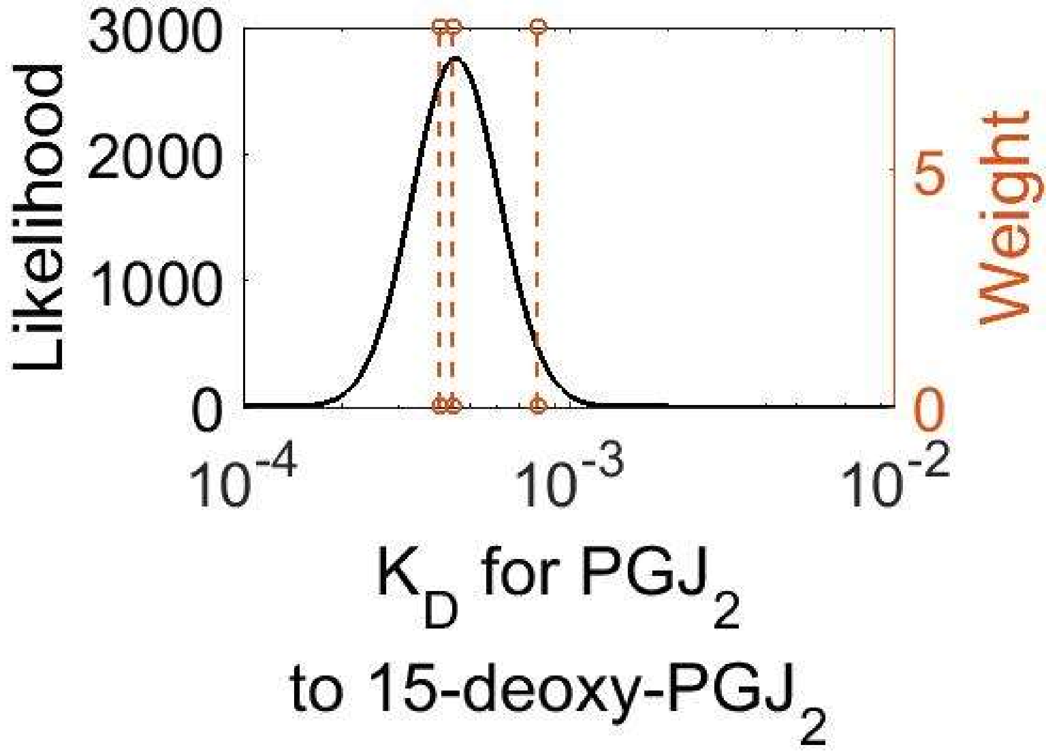


**Figure SF.8.2.1.2.1.** The estimated probability distribution for the Reaction 9 $K_D$. The value and weight of the literature values used to define the distribution are indicated by an orange dashed line. The x axis is plotted on a log-scale.

**S.8.4.1.3. Parameter: $K_r$ (Dependent parameter)**

The log-normal distribution for the parameter for the $K_r$ of Reaction 9 was calculated using multivariate distributions. The log-normal distribution properties for the $K_r$ of Reaction 9 are shown in Table ST.8.4.1.3.1 and plotted in Figure SF.8.4.1.3.1.

**Table ST.8.4.1.3.1.** The log-normal distribution properties of the Reaction 9 $k_r$ distribution. These values were calculated using the functions described in Section 2.6.2.

| **Mode ($M^{-1}$ $min^{-1}$)** | **Location parameter (μ)** | **Scale parameter (σ)** |
|---|---|---|
| 9.63 x$10^{-4}$ | -6.02 | 9.62 x$10^{-1}$ |

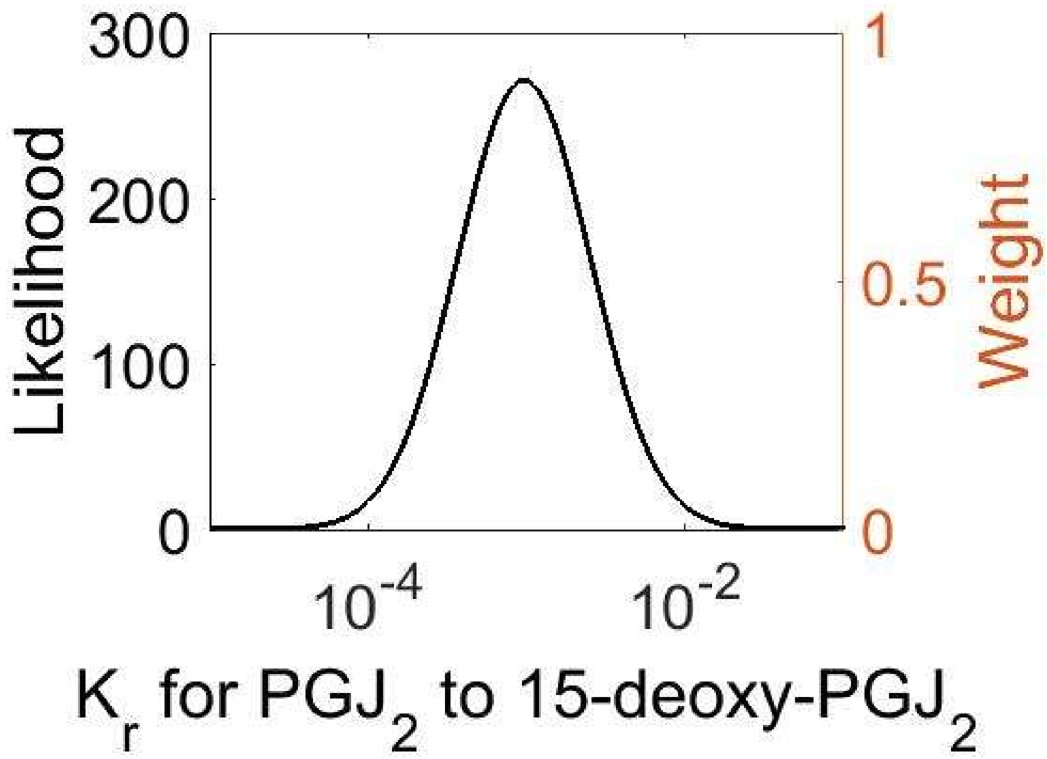


**Figure SF.8.4.1.3.1.** The estimated probability distribution for Reaction 9 $K_r$. The value and weight of the literature values used to define the distribution are indicated by an orange dashed line. The x axis is plotted on a log-scale.

**S.8.5. Reaction 96: exTXA2 ⇌ exTXB2**

$TXA_2$ is rapidly hydrolysed into $TXB_2$ via a non-enzymatic reaction. This inactive metabolite is produced by the incorporation of two hydrogens and one oxygen at C9 and C11, resulting in the opening of the trimethylene oxide ring, and the generation of two hydroxyl groups on the tetrahydropyran ring.

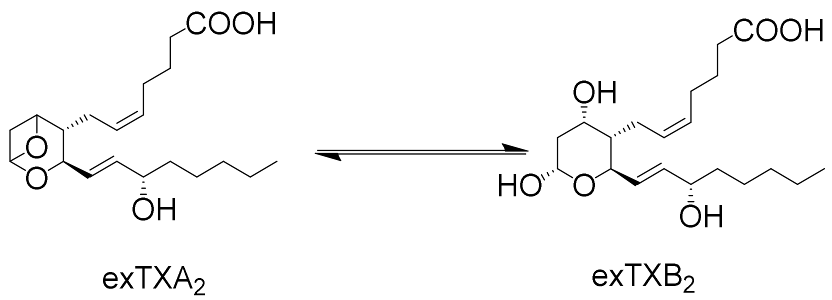


**Figure SF.8.5.** The non-enzymatic hydrolysis reaction of thromboxane $A_2$ ($TXA_2$) into thromboxane $B_2$ ($TXB_2$) in the extracellular compartment (Reaction 96).

**SEq.8.5. Reaction rate law for Reaction 96.**

$$v_{96} = K_f[exTXA_2] - K_r[exTXB_2]$$

### S.8.5.1. Reaction parameters

#### S.8.5.1.1. Parameter: $K_f$

Parameter values for the $K_f$ of Reaction 96 were obtained from the literature and summarised in Table ST.8.5.1.1.1. The log-normal distribution properties for the $K_f$ of Reaction 96 are shown in Table ST.8.5.1.1.2 and plotted in Figure SF.8.5.1.1.1.

**Table ST.8.5.1.1.1.** Literature information used to design the Reaction 96 $K_f$ parameter distribution. Each value was assigned a weight using the protocol described in Section 2.6.2. The type of error was assigned as 0 if it was additive and 1 if it was multiplicative.

| Value ($M^{-1}$ $s^{-1}$) | Error ($M^{-1}$ $s^{-1}$) | Experimental details | | | | Weight | Type of error | Reference |
|---|---|---|---|---|---|---|---|---|
| | | Substrate type | Functional group | Temperature (°C) | Other | | | |
| 3.70 x$10^3$ | 1.00 x$10^2$ | Different cycle | Epoxide | 25 | | **32** | | (Ross et al., 1982) |
| 8.70 x$10^3$ | | Different cycle | Epoxide | 25 | | **32** | | (Ross et al., 1982) |
| 1.10 x$10^4$ | 1.00 x$10^3$ | 6 ring cycle | Epoxide | 25 | | **48** | | (Ross et al., 1982) |
| 2.40 x$10^4$ | | 6 ring cycle | Epoxide | 25 | | **48** | | (Ross et al., 1982) |
| 3.70 x$10^3$ | 1.00 x$10^2$ | Different cycle | Epoxide | 25 | | **32** | | (Ross et al., 1982) |
| 3.60 | 2.00 x$10^{-1}$ | Different cycle | Epoxide | 25 | | **32** | | (Ross et al., 1982) |

| | | | | | | | | |
|---|---|---|---|---|---|---|---|---|
| 1.70 | 1.00 $x10^{-1}$ | Different cycle | Epoxide | 25 | | **32** | | (Ross et al., 1982) |
| 2.67 $x10^{1}$ | 9.00 $x10^{-1}$ | Different cycle | Epoxide | 25 | | **32** | | (Ross et al., 1982) |
| 3.50 $x10^{1}$ | | Different cycle | Epoxide | 25 | | **32** | | (Ross et al., 1982) |

**Table ST.8.5.1.1.2.** The log-normal distribution properties of the Reaction 96 $K_f$ distribution. These values were calculated using the functions described in Section 2.6.2.

| **Mode ($M^{-1}$ $s^{-1}$)** | **Confidence Interval** | **Location parameter (μ)** | **Scale parameter (σ)** |
|---|---|---|---|
| 3.70 $x10^{3}$ | 3.44 $x10^{1}$ | 1.10 $x10^{1}$ | 1.66 |

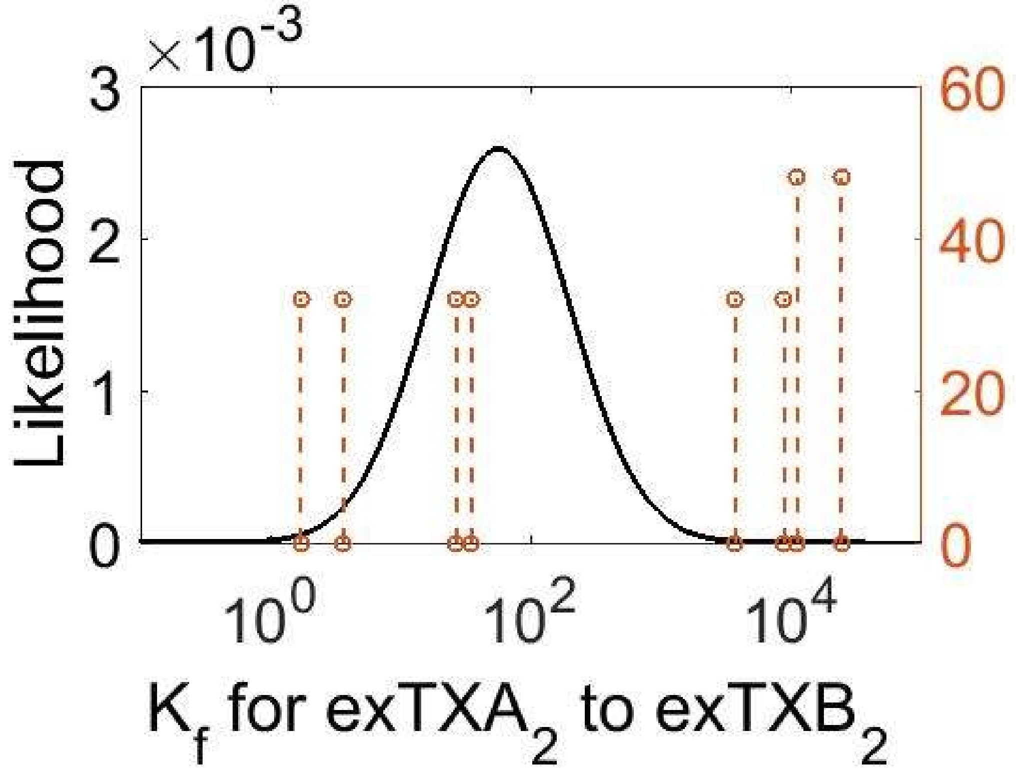


**Figure SF.8.5.1.1.1.** The estimated probability distribution for the Reaction 96 $K_f$. The value and weight of the literature values used to define the distribution are indicated by an orange dashed line. The x axis is plotted on a log-scale.

**S.8.5.1.2. Parameter: $K_D$**

Parameter values for the $K_D$ of Reaction 96 were obtained from the literature and summarised in Table ST.8.5.1.2.1. The log-normal distribution properties for the $K_D$ of Reaction 96 are shown in Table ST.8.5.1.2.2 and plotted in Figure SF.8.5.1.2.1.

**Table ST.8.5.1.2.1.** Literature information used to design the Reaction 96 $K_D$ parameter distribution. Each value was assigned a weight using the protocol described in Section 2.6.2. The type of error was assigned as 0 if it was additive and 1 if it was multiplicative.

| **Value ($M^{-1}$ $min^{-1}$)** | **Error ($M^{-1}$ $min^{-1}$)** | **Experimental details** | | | | **Weight** | **Type of error** | **Reference** |
|---|---|---|---|---|---|---|---|---|
| | | **Species** | **Substrate type** | **Temperature (°C)** | **Other** | | | |
| 0.000038 | NaN | Mosquit o | Carbocyclic $TXA_2$ | 35 | | **24** | | (Alvarenga et al., 2010) |
| 0.00000023 | NaN | Rabbit | $TXA_2$ antagonist | 35 | | **36** | | (Nakahata et al., 1992) |
| 1500 | 500 | N/A | Hydroxysulf amic acid | 25 | | **16** | | (D. LITTLEJOHN , 1988) |
| 1.75 $x10^{-12}$ | NaN | N/A | H2O2 | | | **16** | | (D. LITTLEJOHN , 1988) |
| 5.90 $x10^{-8}$ | NaN | N/A | Oxaplatin | | | **16** | | (Jerremalm et al., 2003) |

**Table ST.8.5.1.2.2. The log-normal distribution properties of the Reaction 96 $K_D$ distribution. These values were calculated using the functions described in Section 2.6.2.**

| Mode ($M^{-1}$ $min^{-1}$) | Confidence Interval | Location parameter (μ) | Scale parameter (σ) |
|---|---|---|---|
| 2.35 $x10^{-7}$ | 1.76 $x10^{4}$ | -6.86 | 2.90 |

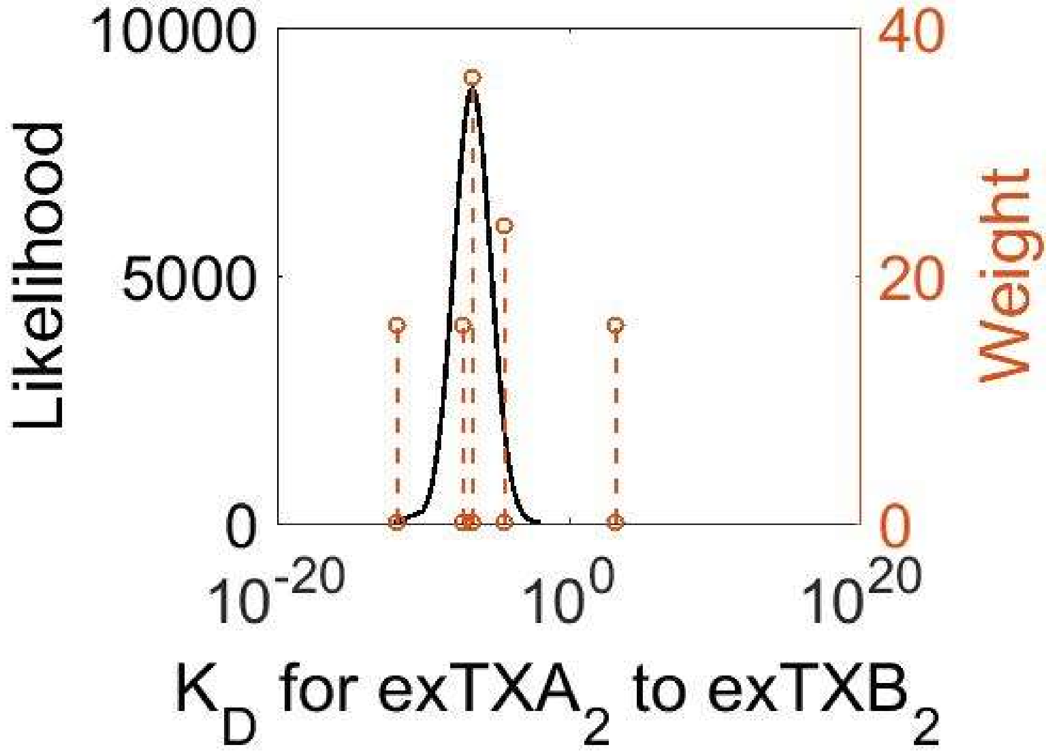


**Figure SF.8.5.1.2.1.** The estimated probability distribution for the Reaction 96 $K_D$. The value and weight of the literature values used to define the distribution are indicated by an orange dashed line. The x axis is plotted on a log-scale.

**S.8.5.1.3. Parameter: $K_r$ (Dependent parameter)**

The log-normal distribution for the parameter for the $K_r$ of Reaction 96 was calculated using multivariate distributions. The log-normal distribution properties for the $K_r$ of Reaction 96 are shown in Table ST.8.5.1.3.1 and plotted in Figure SF.8.5.1.3.1.

**Table ST.8.5.1.3.1.** The log-normal distribution properties of the Reaction 96 $K_r$ distribution. These values were calculated using the functions described in Section 2.6.2.

| Mode ($M^{-1}$ $min^{-1}$) | Location parameter (μ) | Scale parameter (σ) |
|---|---|---|
| $5.28 \times 10^{1}$ | 5.63 | 1.29 |

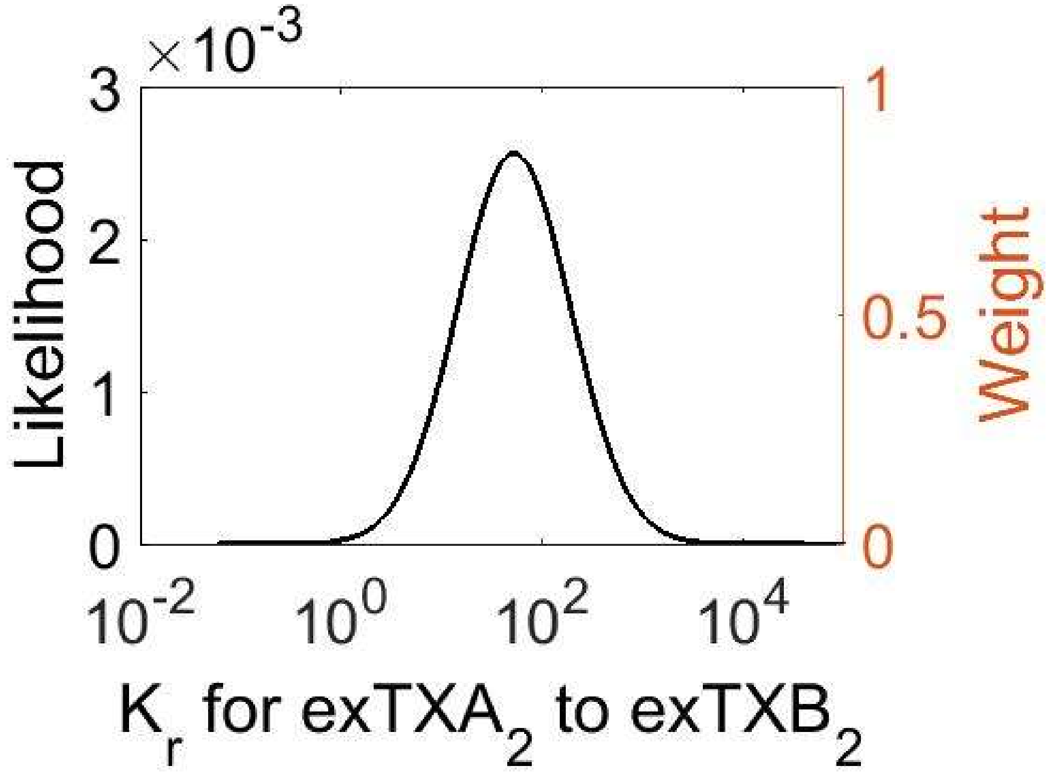


**Figure SF.8.5.1.3.1.** The estimated probability distribution for the Reaction 96 $K_r$. The value and weight of the literature values used to define the distribution are indicated by an orange dashed line. The x axis is plotted on a log-scale.

**S.8.6. Reaction 97: $exPGI_2 \rightleftharpoons ex6\text{-}keto\text{-}PGF_{1\alpha}$**

Due to ring strain, $PGI_2$ is rapidly hydrolysed to 6-keto-$PGF_{1\alpha}$. This is a non-enzymatic reaction and results in the formation of a hydroxyl group at C9 and a ketone at C6, by incorporating two hydrogens and one oxygen.

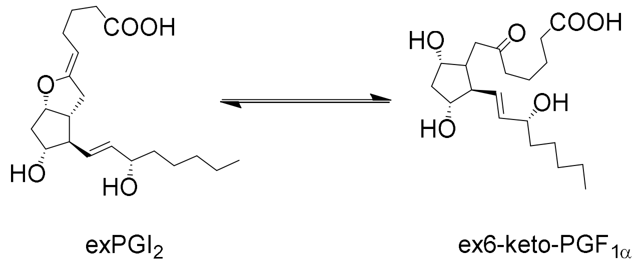


**Figure SF.8.6.** The non-enzymatic hydrolysis of prostaglandin $I_2$ ($PGI_2$) into 6-keto-prostaglandin $F_{1\alpha}$ (6-keto-$PGF_{1\alpha}$) in the extracellular compartment (Reaction 97).

**SEq.8.6.** Reaction rate law for Reaction 97.

$$v_{97} = K_f[exPGI_2] - K_r[ex6 - keto - PGF_{1\alpha}]$$

## S.8.6.1. Reaction parameters

### S.8.6.1.1. Parameter: $K_f$

Parameter values for the $K_f$ of Reaction 97 were obtained from the literature and summarised in Table ST.8.6.1.1.1. The log-normal distribution properties for the $K_f$ of Reaction 97 are shown in Table ST.8.6.1.1.2 and plotted in Figure SF.8.6.1.1.1.

**Table ST.8.6.1.1.1.** Literature information used to design the Reaction 97 $K_f$ parameter distribution. Each value was assigned a weight using the protocol described in Section 2.6.2. The type of error was assigned as 0 if it was additive and 1 if it was multiplicative.

| Value ($M^{-1}$ $s^{-1}$) | Error ($M^{-1}$ $s^{-1}$) | Experimental details | | | | Weight | Type of error | Reference |
|---|---|---|---|---|---|---|---|---|
| | | Substrate type | Functional group | Temperature (°C) | Other | | | |
| 3.70 x$10^3$ | 1.00 x$10^2$ | Different cycle | Epoxide | 25 | | **32** | | (Ross et al., 1982) |
| 8.70 x$10^3$ | | Different cycle | Epoxide | 25 | | **32** | | (Ross et al., 1982) |
| 1.10 x$10^4$ | 1.00 x$10^3$ | 6 ring cycle | Epoxide | 25 | | **48** | | (Ross et al., 1982) |
| 2.40 x$10^4$ | | 6 ring cycle | Epoxide | 25 | | **48** | | (Ross et al., 1982) |
| 3.70 x$10^3$ | 1.00 x$10^2$ | Different cycle | Epoxide | 25 | | **32** | | (Ross et al., 1982) |
| 3.60 | 2.00 x$10^{-1}$ | Different cycle | Epoxide | 25 | | **32** | | (Ross et al., 1982) |

| | | | | | | | | |
|---|---|---|---|---|---|---|---|---|
| 1.70 | 1.00 x$10^{-1}$ | Different cycle | Epoxide | 25 | | **32** | | (Ross et al., 1982) |
| 2.67 x$10^{1}$ | 9.00 x$10^{-1}$ | Different cycle | Epoxide | 25 | | **32** | | (Ross et al., 1982) |
| 3.50 x$10^{1}$ | | Different cycle | Epoxide | 25 | | **32** | | (Ross et al., 1982) |

**Table ST.8.6.1.1.2.** The log-normal distribution properties of the Reaction 97 $K_f$ distribution. These values were calculated using the functions described in Section 2.6.2.

| **Mode ($M^{-1}$ $s^{-1}$)** | **Confidence Interval** | **Location parameter (μ)** | **Scale parameter (σ)** |
|---|---|---|---|
| 3.70 x$10^{3}$ | 3.44 x$10^{1}$ | 1.10 x$10^{1}$ | 1.66 |

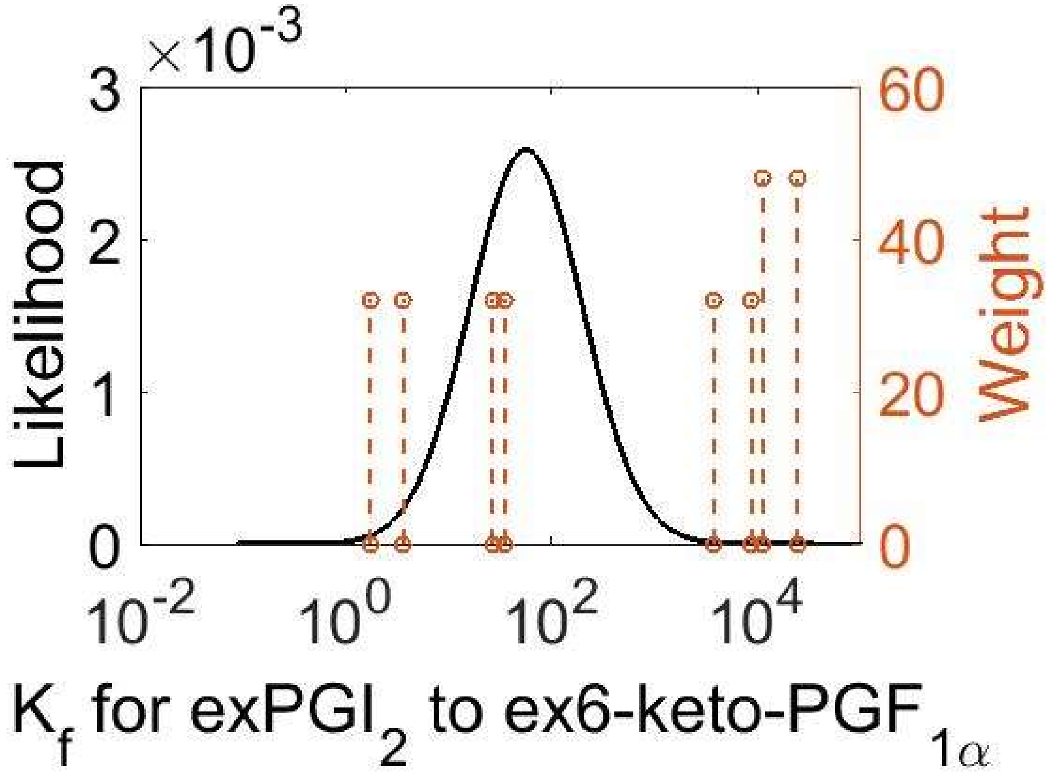


**Figure SF.8.6.1.1.1.** The estimated probability distribution for the Reaction 97 $K_f$. The value and weight of the literature values used to define the distribution are indicated by an orange dashed line. The x axis is plotted on a log-scale.

### S.8.6.1.2. Parameter: $K_D$

Parameter values for the $K_D$ of Reaction 97 were obtained from the literature and summarised in Table ST.8.6.1.2.1. The log-normal distribution properties for the $K_D$ of Reaction 97 are shown in Table ST.8.6.1.2.2 and plotted in Figure SF.8.6.1.2.1.

**Table ST.8.6.1.2.1.** Literature information used to design the Reaction 97 $K_D$ parameter distribution. Each value was assigned a weight using the protocol described in Section 2.6.2. The type of error was assigned as 0 if it was additive and 1 if it was multiplicative.

| **Value ($M^{-1}$ $min^{-1}$)** | **Error ($M^{-1}$ $min^{-1}$)** | **Experimental details** | | | | **Weight** | **Type of error** | **Reference** |
|---|---|---|---|---|---|---|---|---|
| | | **Species** | **Substrate type** | **Temperature (°C)** | **Other** | | | |
| 0.000038 | NaN | Mosquit o | Carbocyclic TXA2 | 35 | | **24** | | (Alvarenga et al., 2010) |
| 0.00000023 | NaN | Rabbit | TXA2 antagonist | 35 | | **36** | | (Nakahata et al., 1992) |
| 1500 | 500 | N/A | Hydroxysulf amic acid | 25 | | **16** | | (D. LITTLEJOH N, 1988) |
| $1.75 \times 10^{-12}$ | NaN | N/A | H2O2 | | | **16** | | (D. LITTLEJOH N, 1988) |
| $5.90 \times 10^{-8}$ | NaN | N/A | Oxaplatin | | | **16** | | (Jerremalm et al., 2003) |

**Table ST.8.6.1.2.2.** The log-normal distribution properties of the Reaction 97 $K_D$ distribution. These values were calculated using the functions described in Section 2.6.2.

| Mode ($M^{-1}$ $min^{-1}$) | Confidence Interval | Location parameter (μ) | Scale parameter (σ) |
|---|---|---|---|
| 2.35 $x10^{-7}$ | 1.76 $x10^{4}$ | -6.86 | 2.90 |

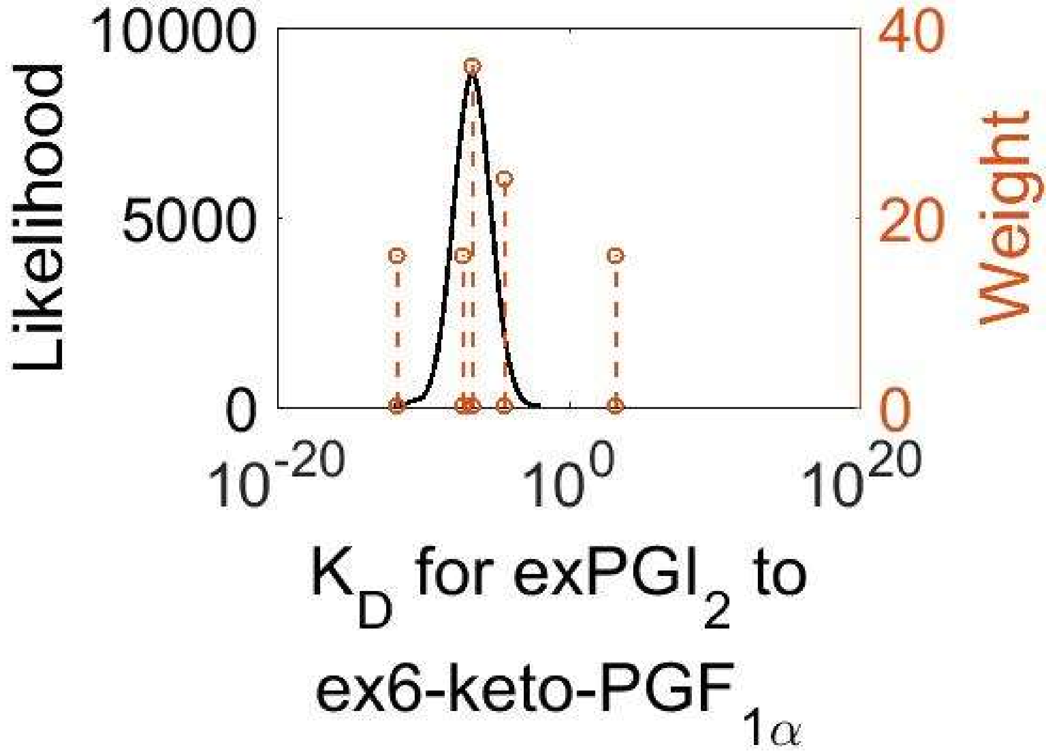


**Figure SF.8.6.1.2.1.** The estimated probability distribution for the Reaction 97 $K_D$. The value and weight of the literature values used to define the distribution are indicated by an orange dashed line. The x axis is plotted on a log-scale.

**S.8.6.1.3. Parameter: $K_r$ (Dependent parameter)**

The log-normal distribution for the parameter for the $K_r$ of Reaction 97 was calculated using multivariate distributions. The log-normal distribution properties for the $K_r$ of Reaction 97 are shown in Table ST.8.6.1.3.1 and plotted in Figure SF.8.6.1.3.1.

**Table ST.8.6.1.3.1.** The log-normal distribution properties of the Reaction 97 $K_r$ distribution. These values were calculated using the functions described in Section 2.6.2.

| Mode ($M^{-1}$ $min^{-1}$) | Location parameter (μ) | Scale parameter (σ) |
|---|---|---|
| 5.81 x$10^1$ | 5.60 | 1.24 |

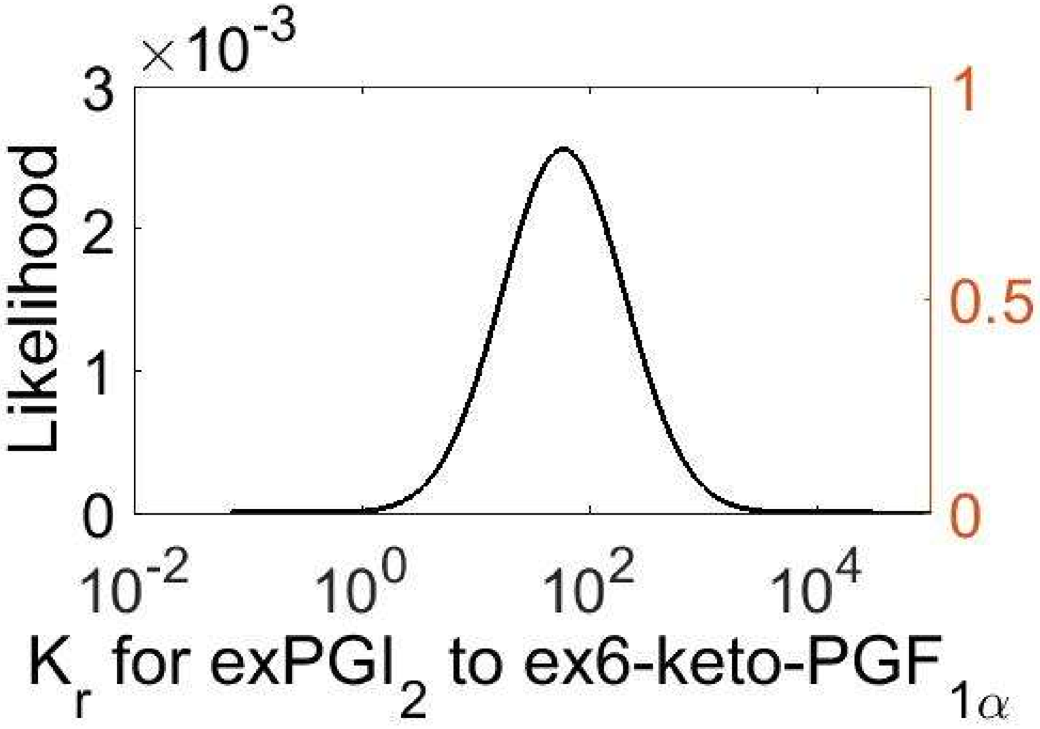


**Figure SF.8.6.1.3.1.** The estimated probability distribution for the Reaction 97 $K_r$. The value and weight of the literature values used to define the distribution are indicated by an orange dashed line. The x axis is plotted on a log-scale.

**S.8.7. Reaction 98: $exPGD_2 \rightleftharpoons exPGJ_2$**

$PGD_2$ is subsequently converted to $PGJ_2$ via a non-enzymatic reaction (Fitzpatrick and Wynalda, 1983). This dehydration reaction includes the removal of two hydrogens and one oxygen molecule, and occurs across the C9 hydroxyl and the C10 carbon axis, to form a double bond between C9 and 10.

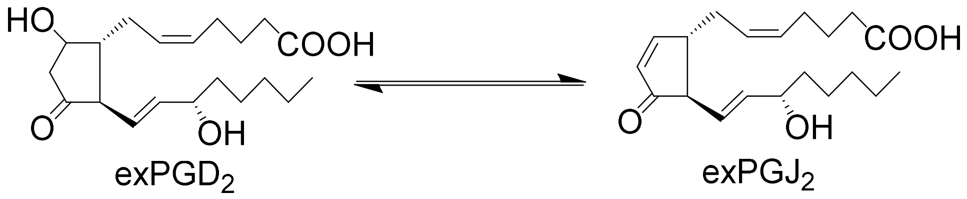


**Figure SF.8.7.** The non-enzymatic dehydration of prostaglandin $D_2$ ($PGD_2$) into prostaglandin $J_2$ ($PGJ_2$) in the extracellular compartment (Reaction 98).

**SEq.8.7. Reaction rate law for Reaction 98.**

$$\boldsymbol{v_{98} = K_f[exPGD_2] - K_r[exPGJ_2]}$$

## S.8.7.1. Reaction parameters

### S.8.7.1.1. Parameter: $K_f$

Parameter values for the $K_f$ of Reaction 98 were obtained from the literature and summarised in Table ST.8.7.1.1.1. The log-normal distribution properties for the $K_f$ of Reaction 98 are shown in Table ST.8.7.1.1.2 and plotted in Figure SF.8.7.1.1.1.

**Table ST.8.7.1.1.1.** Literature information used to design the Reaction 98 $K_f$ parameter distribution. Each value was assigned a weight using the protocol described in Section 2.6.2. The type of error was assigned as 0 if it was additive and 1 if it was multiplicative.

| Value ($M^{-1}$ $min^{-1}$) | Error ($M^{-1}$ $min^{-1}$) | Experimental details | | | | Weight | Type of error | Reference |
|---|---|---|---|---|---|---|---|---|
| | | Substrate type | Functional group | Temperature (°C) | Other | | | |
| 3.30 $x10^{6}$ | | Different | Carboxylic acid | 25 | | 32 | | (B. H. GIBBONS, 1963) |
| 4.50 | | Different | | 20 | | 32 | | (Tur'yan, 1998) |
| 4.20 $x10^{-1}$ | | Different | | 20 | | 32 | | (Ranney and Ziemann, 2016) |

**Table ST.8.7.1.1.2.** The log-normal distribution properties of the Reaction 98 $K_f$ distribution. These values were calculated using the functions described in Section 2.6.2.

| **Mode** **($M^{-1}$ $min^{-1}$)** | **Confidence Interval** | **Location parameter (μ)** | **Scale parameter (σ)** |
|---|---|---|---|
| 4.48 | $1.09 \times 10^3$ | 7.34 | 2.42 |

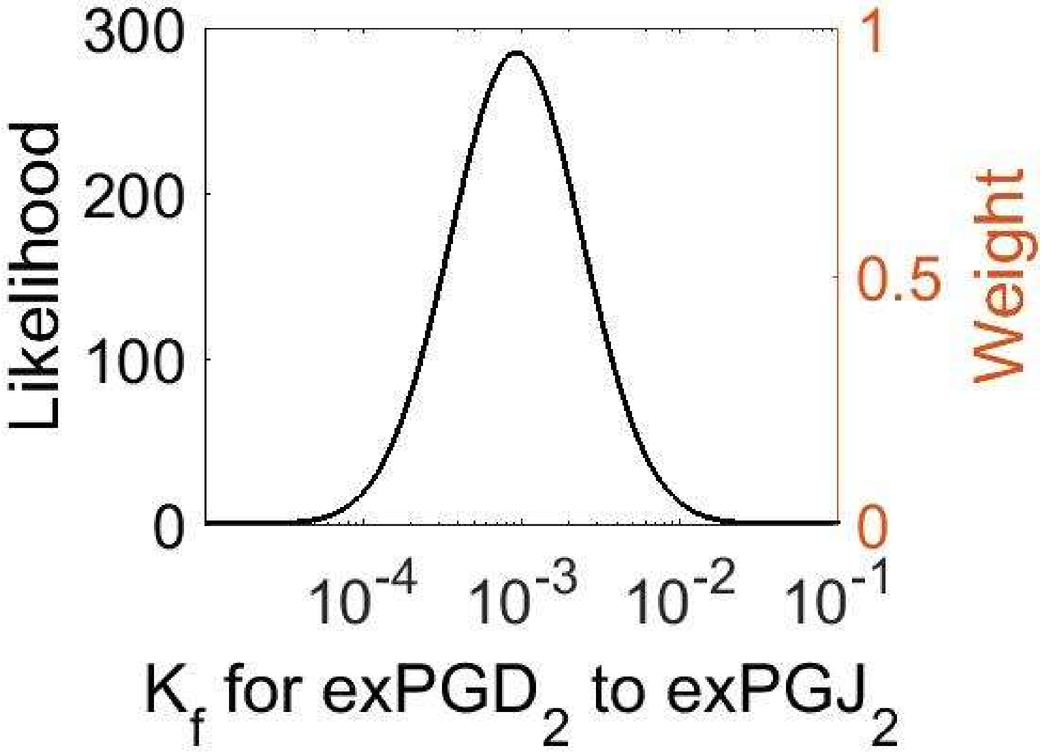


**Figure SF.8.7.1.1.1.** The estimated probability distribution for the Reaction 98 $K_f$. The value and weight of the literature values used to define the distribution are indicated by an orange dashed line. The x axis is plotted on a log-scale.

### S.8.7.1.2. Parameter: $K_D$

Parameter values for the $K_D$ of Reaction 98 were obtained from the literature and summarised in Table ST.8.7.1.2.1. The log-normal distribution properties for the $K_D$ of Reaction 98 are shown in Table ST.8.7.1.2.2 and plotted in Figure SF.8.7.1.2.1.

**Table ST.8.7.1.2.1.** Literature information used to design the Reaction 98 $K_D$ parameter distribution. Each value was assigned a weight using the protocol described in Section 2.6.2. The type of error was assigned as 0 if it was additive and 1 if it was multiplicative.

| Value ($M^{-1}$ $min^{-1}$) | Error ($M^{-1}$ $min^{-1}$) | Experimental details | | | | Weight | Type of error | Reference |
|---|---|---|---|---|---|---|---|---|
| | | Substrate | Functional group | Temperature (°C) | Other | | | |
| 8.00 x10$^{-4}$ | NaN | OH radicals | tetrahydrofuran | Unknown | | **8** | | (Ranney and Ziemann, 2016) |
| 4.00 x10$^{-4}$ | NaN | H2CO3 | carbonate | | | **8** | | (Buytendyk et al., 1927) |
| 4.40 x10$^{-4}$ | NaN | H2CO3 | carbonate | | | **8** | | (Thiel and Strohecker, 1914) |

**Table ST.8.7.1.2.2** The log-normal distribution properties of the Reaction 98 $K_D$ distribution. These values were calculated using the functions described in Section 2.6.2.

| **Mode** **($M^{-1}$ $min^{-1}$)** | **Confidence Interval** | **Location parameter (μ)** | **Scale parameter (σ)** |
|---|---|---|---|
| 4.49 x$10^{-4}$ | 1.38 | -7.61 | 3.07 x$10^{-1}$ |

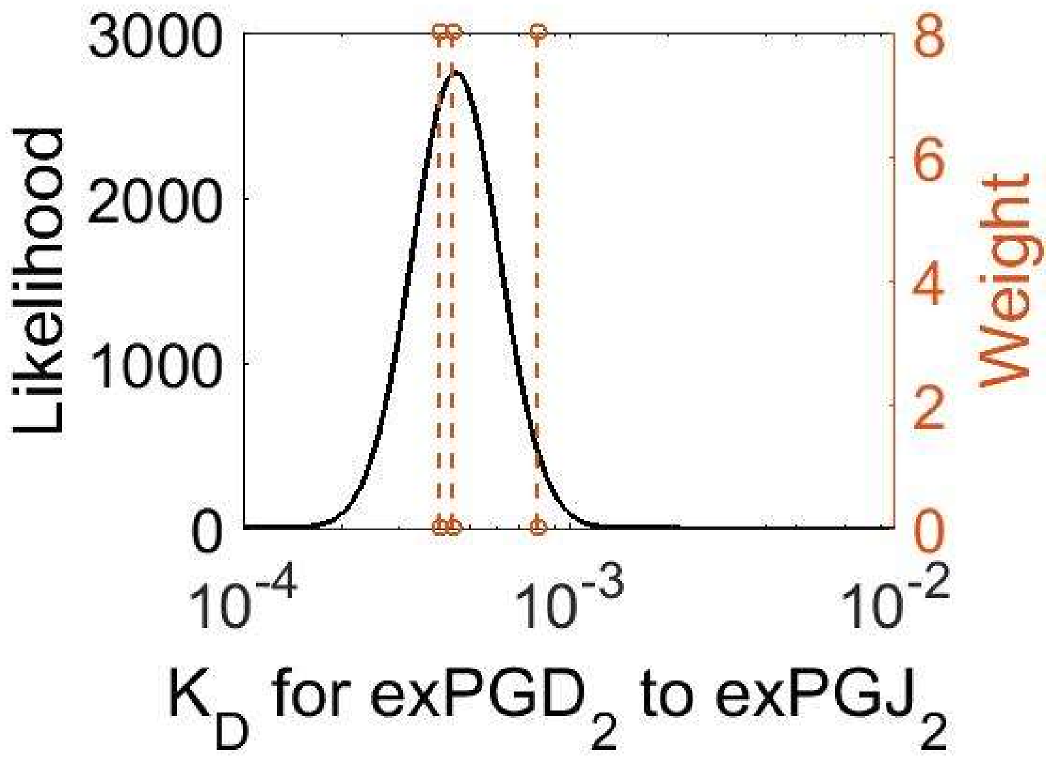


**Figure SF.8.7.1.2.1.** The estimated probability distribution for the Reaction 98 $K_D$. The value and weight of the literature values used to define the distribution are indicated by an orange dashed line. The x axis is plotted on a log-scale.

**S.8.7.1.3. Parameter: $K_r$ (Dependent parameter)**

The log-normal distribution for the parameter for the $K_r$ of Reaction 98 was calculated using multivariate distributions. The log-normal distribution properties for the $K_r$ of Reaction 98 are shown in Table ST.8.7.1.3.1 and plotted in Figure SF.8.7.1.3.1.

**Table ST.8.7.1.3.1.** The log-normal distribution properties of the Reaction 98 $K_r$ distribution. These values were calculated using the functions described in Section 2.6.2.

| **Mode ($M^{-1}$ $min^{-1}$)** | **Location parameter (μ)** | **Scale parameter (σ)** |
|---|---|---|
| $9.25 \times 10^{-4}$ | -6.07 | $9.57 \times 10^{-1}$ |

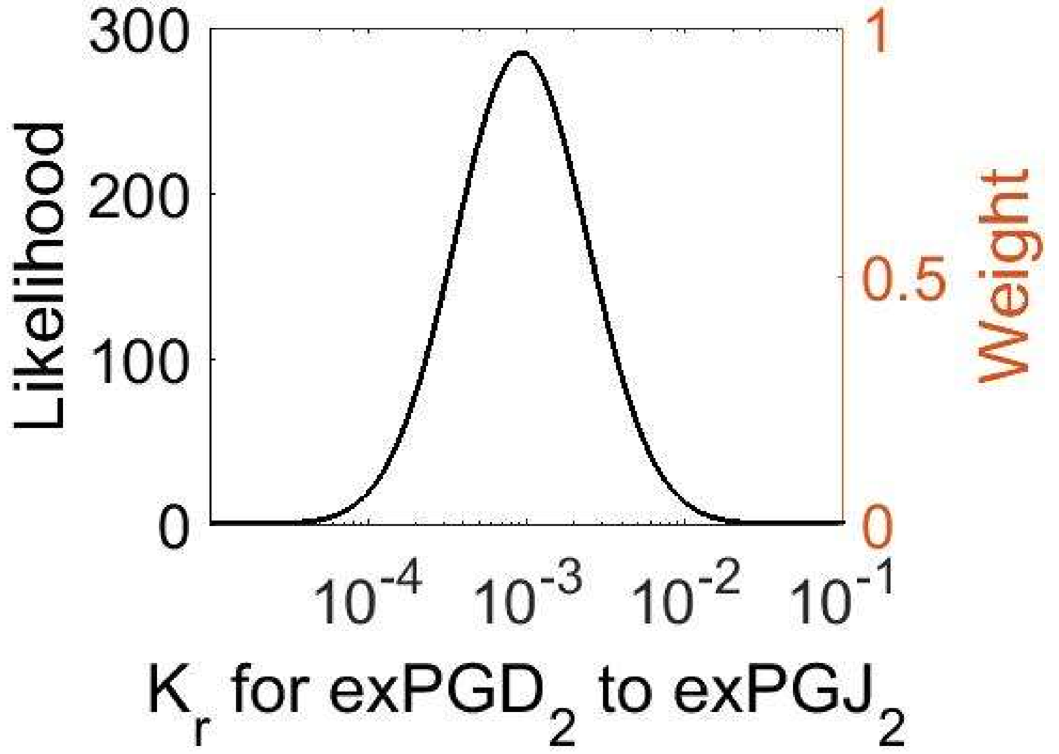


**Figure SF.8.7.1.3.1.** The estimated probability distribution for the Reaction 98 $K_r$. The value and weight of the literature values used to define the distribution are indicated by an orange dashed line. The x axis is plotted on a log-scale.

**S.8.8. Reaction 99: $exPGJ_2 \rightleftharpoons ex15\text{-}deoxy\text{-}PGJ_2$**

$PGJ_2$ is subject to a non-enzymatic dehydration reaction to yield 15-deoxy-$PGJ_2$. This dehydration reaction includes the removal of two hydrogens and one oxygen molecule, and occurs across the C15 hydroxyl and the C14 carbon axis, to form a double bond between C15 and 14.

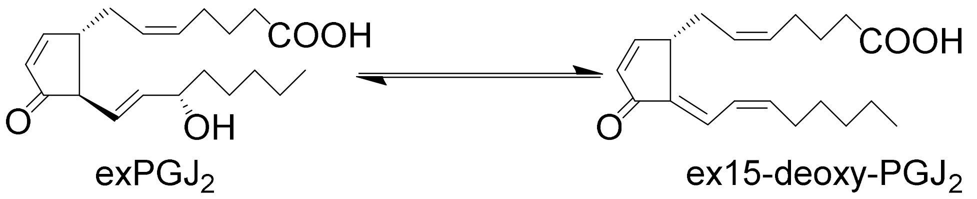


**Figure SF.8.8.** The non-enzymatic dehydration of prostaglandin $J_2$ ($PGJ_2$) into 15-deoxy-$PGJ_2$ (15-deoxy-$PGJ_2$) in the extracellular compartment (Reaction 99).

**SEq.8.8.** Reaction rate law for Reaction 99.

$$\boldsymbol{v_{99} = K_f[exPGJ_2] - K_r[ex15 - deoxy - PGJ_2]}$$

### S.8.8.1. Reaction parameters

#### S.8.8.1.1. Parameter: $K_f$

Parameter values for the $K_f$ of Reaction 99 were obtained from the literature and summarised in Table ST.8.8.1.1.1. The log-normal distribution properties for the $K_f$ of Reaction 99 are shown in Table ST.8.8.1.1.2 and plotted in Figure SF.8.8.1.1.1.

**Table ST.8.8.1.1.1.** Literature information used to design the Reaction 99 $K_f$ parameter distribution. Each value was assigned a weight using the protocol described in Section 2.6.2. The type of error was assigned as 0 if it was additive and 1 if it was multiplicative.

| Value ($M^{-1}$ $min^{-1}$) | Error ($M^{-1}$ $min^{-1}$) | Experimental details | | | | Weight | Type of error | Reference |
|---|---|---|---|---|---|---|---|---|
| | | Substrate type | Functional group | Temperature (°C) | Other | | | |
| 3.30 x$10^{6}$ | | Different | Carboxylic acid | 25 | | 32 | | (B. H. GIBBONS, 1963) |
| 4.50 | | Different | | 20 | | 32 | | (Tur'yan, 1998) |
| 4.20 x$10^{-1}$ | | Different | | 20 | | 32 | | (Ranney and Ziemann, 2016) |

**Table ST.8.8.1.1.2.** The log-normal distribution properties of the Reaction 99 $K_f$ distribution. These values were calculated using the functions described in Section 2.6.2.

| Mode ($M^{-1}$ $min^{-1}$) | Confidence Interval | Location parameter (μ) | Scale parameter (σ) |
|---|---|---|---|
| 4.48 | 1.09 x$10^3$ | 7.34 | 2.42 |

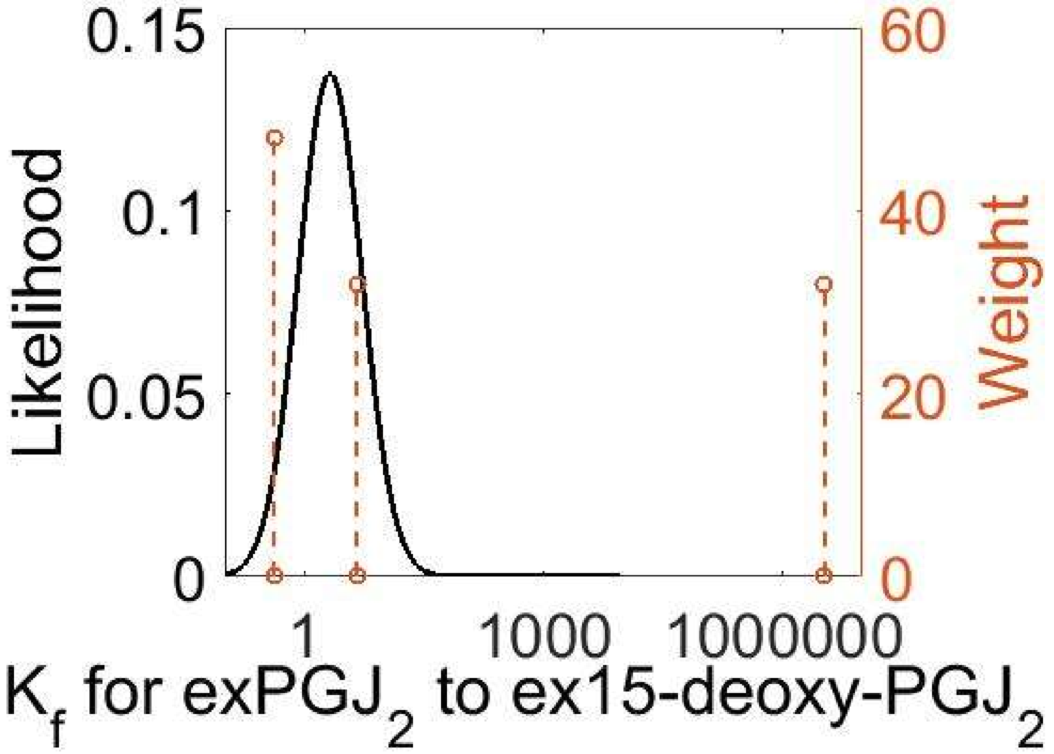


**Figure SF.8.8.1.1.1.** The estimated probability distribution for the Reaction 99 $K_f$. The value and weight of the literature values used to define the distribution are indicated by an orange dashed line. The x axis is plotted on a log-scale.

### S.8.8.1.2. Parameter: $K_D$

Parameter values for the $K_D$ of Reaction 99 were obtained from the literature and summarised in Table ST.8.8.1.2.1. The log-normal distribution properties for the $K_D$ of Reaction 99 are shown in Table ST.8.8.1.2.2 and plotted in Figure SF.8.8.1.2.1.

**Table ST.8.8.1.2.1.** Literature information used to design the Reaction 99 $K_D$ parameter distribution. Each value was assigned a weight using the protocol described in Section 2.6.2. The type of error was assigned as 0 if it was additive and 1 if it was multiplicative.

| Value ($M^{-1}$ $min^{-1}$) | Error ($M^{-1}$ $min^{-1}$) | Experimental details | | | | Weight | Type of error | Reference |
|---|---|---|---|---|---|---|---|---|
| | | Substrate | Functional group | Temperature (°C) | Other | | | |
| 8.00 x$10^{-4}$ | NaN | OH radicals | tetrahydrofuran | Unknown | | **8** | | (Ranney and Ziemann, 2016) |
| 4.00 x$10^{-4}$ | NaN | H2CO3 | carbonate | | | **8** | | (Buytendyk et al., 1927) |
| 4.40 x$10^{-4}$ | NaN | H2CO3 | carbonate | | | **8** | | (Thiel and Strohecker, 1914) |

**Table ST.8.8.1.2.2.** The log-normal distribution properties of the Reaction 99 $K_D$ distribution. These values were calculated using the functions described in Section 2.6.2.

| **Mode ($M^{-1}$ $min^{-1}$)** | **Confidence Interval** | **Location parameter (μ)** | **Scale parameter (σ)** |
|---|---|---|---|
| 4.49 $x10^{-4}$ | 1.38 | -7.61 | 3.07 $x10^{-1}$ |

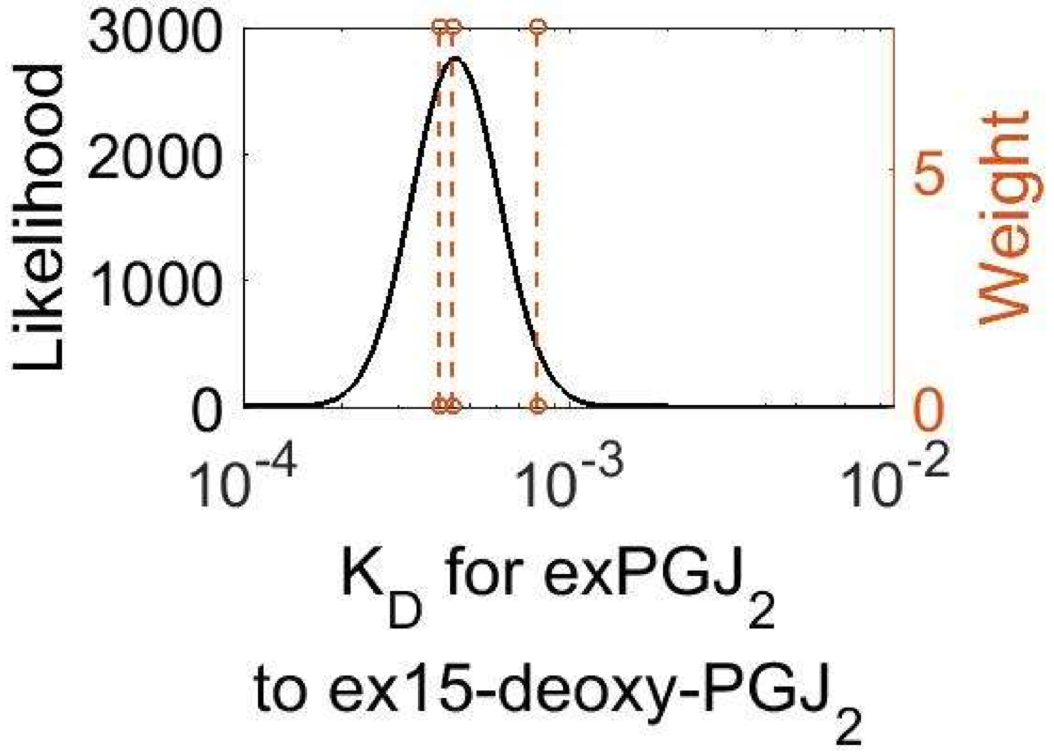


**Figure SF.8.8.1.2.1.** The estimated probability distribution for the Reaction 99 $K_D$. The value and weight of the literature values used to define the distribution are indicated by an orange dashed line. The x axis is plotted on a log-scale.

**S.8.8.1.3. Parameter: $K_r$ (Dependent parameter)**

The log-normal distribution for the parameter for the $K_r$ of Reaction 99 was calculated using multivariate distributions. The log-normal distribution properties for the $K_r$ of Reaction 99 are shown in Table ST.8.8.1.3.1 and plotted in Figure SF.8.8.1.3.1.

**Table ST.8.8.1.3.1.** The log-normal distribution properties of the Reaction 99 $K_r$ distribution. These values were calculated using the functions described in Section 2.6.2.

| Mode ($M^{-1}$ $min^{-1}$) | Location parameter (μ) | Scale parameter (σ) |
|---|---|---|
| 9.63 $x10^{-4}$ | -6.02 | 9.62 $x10^{-1}$ |

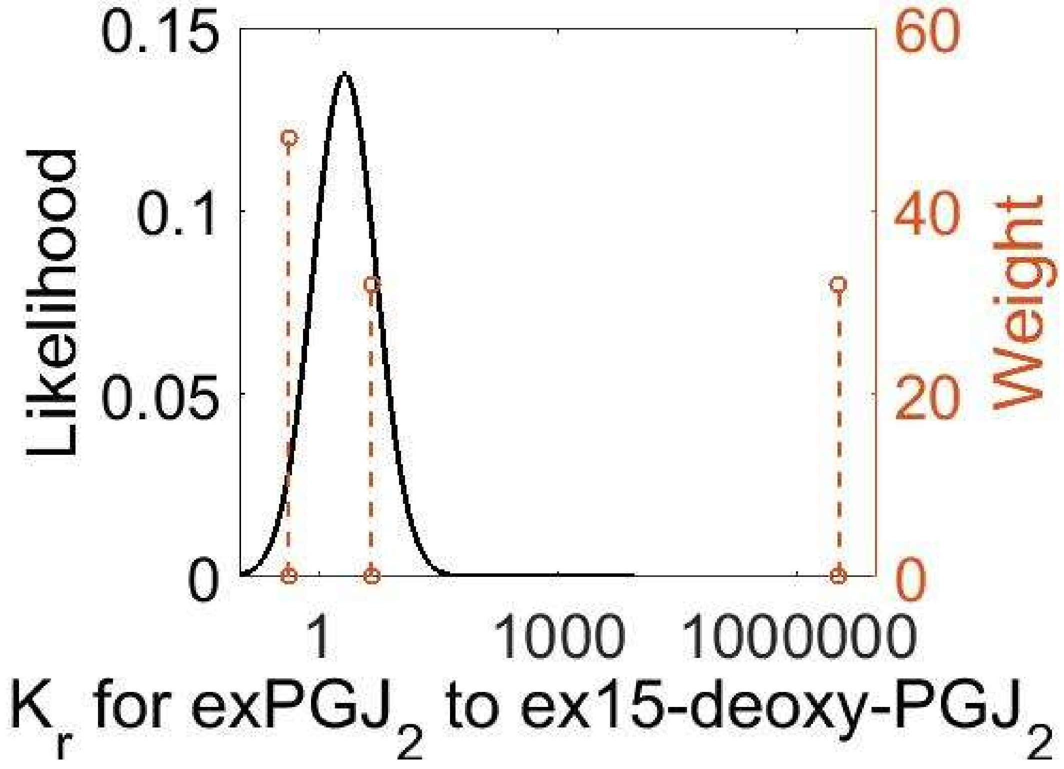


**Figure SF.8.8.1.3.1.** The estimated probability distribution for the Reaction 99 $K_r$. The value and weight of the literature values used to define the distribution are indicated by an orange dashed line. The x axis is plotted on a log-scale.

References

Alvarenga, P. H., et al. (2010). 'The function and three-dimensional structure of a thromboxane a2/cysteinyl leukotriene-binding protein from the saliva of a mosquito vector of the malaria parasite' *PLoS Biol*, 8 (11), p. e1000547. DOI: 10.1371/journal.pbio.1000547.

B. H. GIBBONS, J. T. E. (1963). 'Rate of hydration of carbon dioxide and dehydration

of carbonic acid at 25 degrees c' *J Biol Chem*, 238 pp. 3502-3507.

Buytendyk, F. J., Brinkman, R. & Mook, H. W. (1927). 'A study of the system carbonic acid, carbon dioxide and water: Determination of the true dissociation-constant of carbonic acid' *Biochem J*, 21 (3), pp. 576-584. Available at: https://www.ncbi.nlm.nih.gov/pubmed/16743873

https://www.ncbi.nlm.nih.gov/pmc/PMC1251954/.

D. LITTLEJOHN, A. R. W., and S. G. CHAN (1988). 'The dissociation constant and acid hydrolysis rate of hydroxysulfamic acid' *Can J Chem*, pp. 1596-1598.

Fitzpatrick, F. A. & Wynalda, M. A. (1983). 'Albumin-catalyzed metabolism of prostaglandin d2. Identification of products formed in vitro' *J Biol Chem*, 258 (19), pp. 11713-8.

Jerremalm, E., Eksborg, S. & Ehrsson, H. (2003). 'Hydrolysis of oxaliplatin—evaluation of the acid dissociation constant for the oxalato monodentate complex' *J Pharm Sci*, 92 (2), pp. 436-438. DOI: https://doi.org/10.1002/jps.10306 Available at: http://www.sciencedirect.com/science/article/pii/S0022354916311649.

Nakahata, N., et al. (1992). 'The presence of thromboxane a2 receptors in cultured astrocytes from rabbit brain' *Brain Res*, 583 (1), pp. 100-104. DOI: https://doi.org/10.1016/S0006-8993(10)80013-7 Available at: http://www.sciencedirect.com/science/article/pii/S0006899310800137.

Ranney, A. P. & Ziemann, P. J. (2016). 'Kinetics of acid-catalyzed dehydration of cyclic hemiacetals in organic aerosol particles in equilibrium with nitric acid vapor' *J Phys Chem A*, 120 (16), pp. 2561-2568. DOI: 10.1021/acs.jpca.6b01402 Available at: https://doi.org/10.1021/acs.jpca.6b01402.

Ross, A. M., et al. (1982). 'Vinyl epoxide hydrolysis reactions' *J Am Chem Soc*, 104 (6), pp. 1658-1665. DOI: 10.1021/ja00370a035 Available at: https://doi.org/10.1021/ja00370a035.

Thiel, A. & Strohecker, R. (1914). 'Die dynamik der zeitreaktion zwischen wäßriger kohlensäure und basen' *Ber Dtsch Chem Ges*, 47 (1), pp. 1061-1068. DOI: 10.1002/cber.191404701173 Available at: https://doi.org/10.1002/cber.191404701173.

Tur'yan, Y. I. (1998). 'Kinetics and equilibrium of the dehydration-hydration and recombination-dissociation reactions of glyoxylic acid investigated by electrochemical methods' *Croat Chem Acta*, 71 (1), pp. 727-743.

## Supplementary Document S9. Decay Reaction Structure and Parameterisation.

Documentation of parameter values obtained for all decay reactions in the model (Reactions 44–64, 68, 71–94; Supplementary Table S4) from the literature and associated uncertainty for the eicosanoid network model. Parameterisation was performed using the method of Tsigkinopoulou *et al.,* (2018). The table includes information regarding each reaction and its respective parameters are documented. This includes information such as the reaction rate law and the literature values that were used to define parameters, including experimental conditions, total weights and literature references from which the data were obtained. In this model some parameters are referred as "Dependent parameters", meaning that the log-normal distribution for that parameter was calculated using multivariate distributions (discussed in **Section 2.6.2**). As a result, no confidence interval factor or literature values were cited for the Dependent parameters.

**Contents**

S.9.1 Reaction 44: ex$PGF_{2\alpha}$ →ϕ ........ 315

S.9.2. Reaction 45: ex$TXB_2$ →ϕ ........ 318

S.9.3. Reaction 46: ex$TXA_2$ →ϕ ........ 321

S.9.4. Reaction 47: ex6-keto-$PGF_{1\alpha}$ →ϕ ........ 324

S.9.5. Reaction 48: ex$PGI_2$ →ϕ ........ 327

S.9.6. Reaction 49: ex$PGE_2$ →ϕ ........ 330

S.9.7. Reaction 50: ex15-deoxy-$PGJ_2$ →ϕ ........ 333

S.9.8. Reaction 51: ex$PGJ_2$ →ϕ ........ 336

S.9.9. Reaction 52: ex$PGD_2$ →ϕ ........ 339

S.9.10. Reaction 53: ex$PGH_2$ →ϕ ........ 342

S.9.11. Reaction 54: ex5-oxo-ETE →ϕ ........ 345

S.9.12. Reaction 55: ex5-HETE →ϕ ........ 348

S.9.12. Reaction 56: ex$LTB_4$ →ϕ ........ 351

S.9.13. Reaction 57: ex$LTC_4$ →ϕ ........ 354

S.9.14. Reaction 58: ex$LTA_4$ →ϕ ........ 357

S.9.15. Reaction 59: ex5-HPETE →ϕ ........ 360

S.9.16. Reaction 60: ex15-HETE→ϕ ........ 363

S.9.17. Reaction 61: ex15-HPETE→ϕ ........ 366

S.9.18. Reaction 62: ex12-HETE →ϕ ........ 369

S.9.19. Reaction 63: ex12-HPETE→ϕ ........ 372

S.9.20. Reaction 64: exAA →ϕ ........ 375

S.9.21. Reaction 68: ex15-keto-$PGE_2$ →ϕ ........ 378

S.9.22. Reaction 71: ex13,14-dihydro-15-keto-$PGE_2$ →ϕ ........ 381

S.9.23. Reaction 72: AA →ϕ ........ 384

S.9.24. Reaction 73: $PGH_2$ →ϕ ........ 387

S.9.25. Reaction 74: $PGF_{2\alpha}$→ϕ ........ 390

S.9.26. Reaction 75: $TXA_2$→ϕ ........ 393

S.9.27. Reaction 76: $PGE_2$→ϕ ........ 396

S.9.28. Reaction 77: $PGI_2$→ϕ ........ 399

S.9.29. Reaction 78: $PGD_2$→ϕ ........ 402

S.9.30. Reaction 79: 15-keto-$PGE_2$ →ϕ ........ 405

S.9.31. Reaction 80: 13,14-dihydro-15-keto-$PGE_2$ →ϕ ........ 408

S.9.32. Reaction 81: $TXB_2$→ϕ ........ 411

S.9.33. Reaction 82: 6-keto-$PGF_{1\alpha}$→ϕ ........ 414

S.9.34. Reaction 83: $PGJ_2$ →ϕ ........ 417

S.9.35. Reaction 84: 15-deoxy-$PGJ_2$ →ϕ ........ 420

S.9.36. Reaction 85: 15-HPETE →ϕ ........ 423

S.9.37. Reaction 86: 12-HPETE →ϕ ........ 426

S.9.38. Reaction 87: 5-HPETE →ϕ ........ 429

S.9.39. Reaction 88: 15-HETE →ϕ ........ 432

S.9.40. Reaction 89: 12-HETE →ϕ ........ 435

S.9.41. Reaction 90: 5-HETE→ϕ....438

S.9.42. Reaction 91: 5-oxo-ETE→ϕ....441

S.9.43. Reaction 92: $LTA_4$→ϕ....444

S.9.44. Reaction 93: $LTB_4$→ϕ....447

S.9.45. Reaction 94: $LTC_4$→ϕ....450

References....453

**S.9.1 Reaction 44: ex$PGF_{2\alpha}$ →ϕ**

The decay of $PGF_{2\alpha}$ in the extracellular compartment into metabolites outside of the scope of this model. An example of metabolites outside of the scope of this model includes metabolites which have been removed from the extracellular compartment into the systemic circulation or are degraded into metabolites not of interest to this model, the term decay reaction refers to the decrease in metabolite concentration by this collection of unspecified processes.

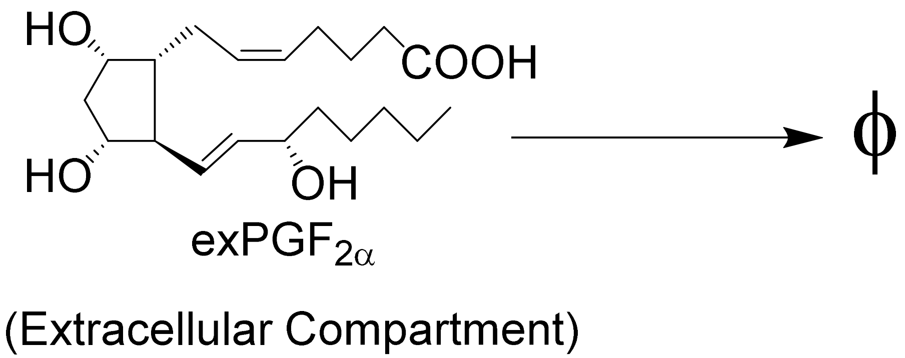


**Figure SF.9.1.** The decay of prostaglandin $F_{2\alpha}$ ($PGF_{2\alpha}$) in the extracellular compartment (Reaction 44).

**SEq. 9.1.** Reaction rate law for Reaction 44.

$$\boldsymbol{v_{44} = K[ex\mathrm{PGF}_{2\alpha}]}$$

### S.9.1.1. Reaction parameters

#### S.9.1.1.1. Parameter: K

Parameter values for the K of Reaction 44 were obtained from the literature and summarised in Table ST.9.1.1.1.1. The log-normal distribution properties for the K of Reaction 44 are shown in Table ST.9.1.1.1.2 and plotted in Figure SF.9.1.1.1.1.

**Table ST.9.1.1.1.1.** Literature information used to design the exPGF$_{2\alpha}$ decay constant parameter distribution. Each value was assigned a weight using the protocol described in Section 2.6.2. The type of error was assigned as 0 if it was additive and 1 if it was multiplicative.

| Half Life (min) | Error (min) | Rate constant (min$^{-1}$) | Error (min$^{-1}$) | Experimental details | | | | | | | Weight | Reference |
|---|---|---|---|---|---|---|---|---|---|---|---|---|
| | | | | Experiment type | Substrate | Species | Expression Vector | pH | Temperature (°C) | Other | | |
| 900 | 492 | 0.001 | 0.001 | In vivo | PGF2a | Human | Decidual stromal cells and macrophages | Unknown | Unknown | | 8 | (Ishihara et al., 1991) |

**Table ST.9.1.1.1.2.** The log-normal distribution properties of the exPGF$_{2\alpha}$ decay constant distribution. These values were calculated using the functions described in Section 2.6.2.

| Mode (min$^{-1}$) | Confidence Interval | Location parameter (μ) | Scale parameter (σ) |
|---|---|---|---|
| 7.07 x10$^{-4}$ | 2.30 | -6.80 | 6.7610$^{-1}$ |

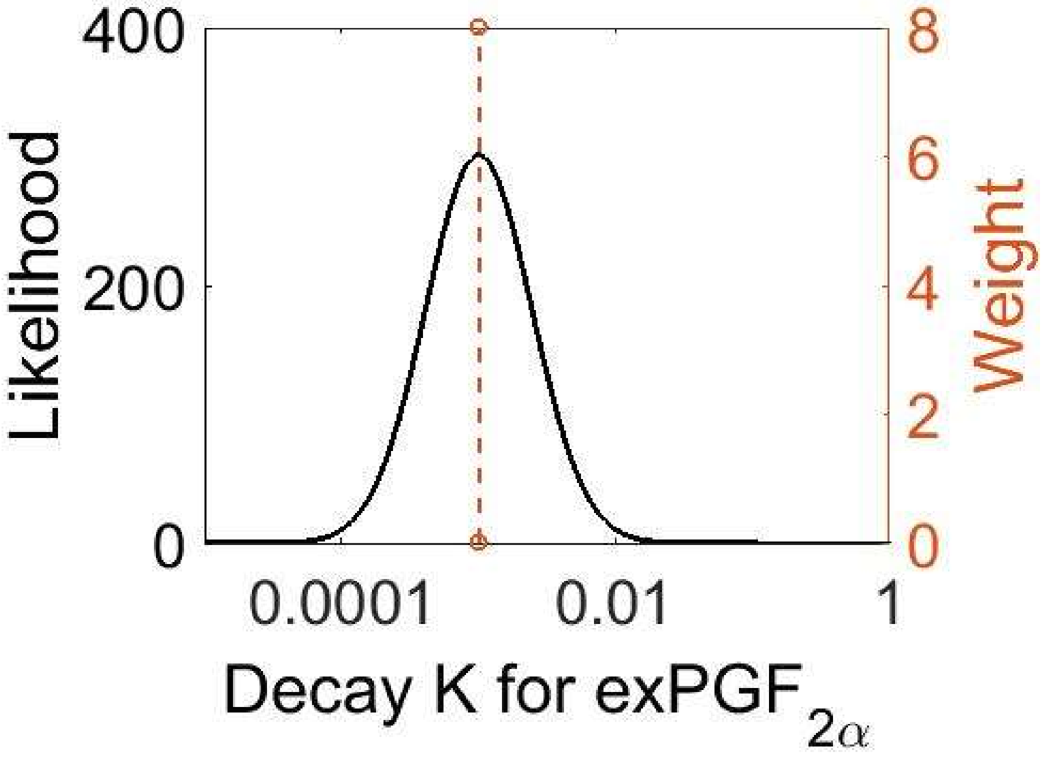


**Figure SF.9.1.1.1.1.** The estimated probability distribution for $exPGF_{2\alpha}$ decay constant. The value and weight of the literature values used to define the distribution are indicated by an orange dashed line. The x axis is plotted on a log-scale.

**S.9.2. Reaction 45: exTXB₂ →ϕ**

The decay of $TXB_2$ in the extracellular compartment into metabolites outside of the scope of this model. An example of metabolites outside of the scope of this model includes metabolites which have been removed from the extracellular compartment into the systemic circulation or are degraded into metabolites not of interest to this model, the term decay reaction refers to the decrease in metabolite concentration by this collection of unspecified processes.

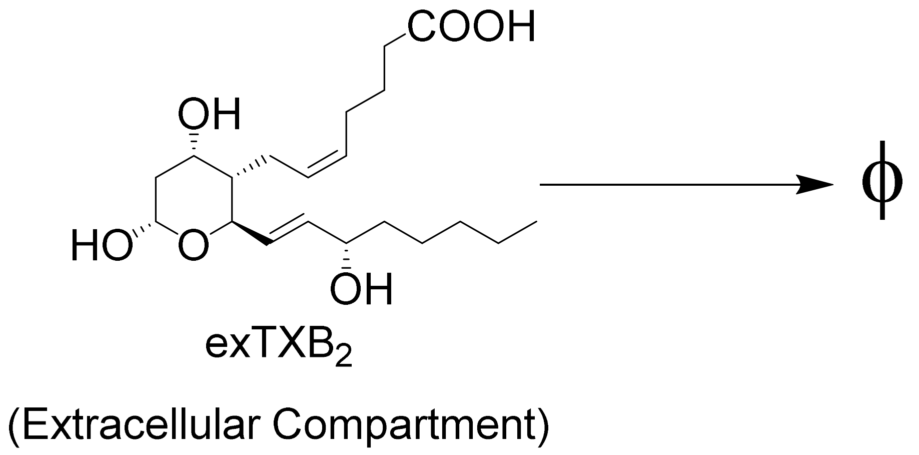


**Figure SF.9.2.** The decay of thromboxane $B_2$ ($TXB_2$) in the extracellular compartment (Reaction 45).

**SEq.9.2.** Reaction rate law for Reaction 45.

$$\boldsymbol{v_{45} = K[ex\mathrm{TXB}_2]}$$

## S.9.2.1. Reaction parameters

### S.9.2.1.1. Parameter: K

Parameter values for the K of Reaction 45 were obtained from the literature and summarised in Table ST.9.2.1.1.1. The log-normal distribution properties for the K of Reaction 45 are shown in Table ST.9.2.1.1.2 and plotted in Figure SF.9.2.1.1.1.

**Table ST.9.2.1.1.1.** Literature information used to design the $exTXB_2$ decay constant parameter distribution. Each value was assigned a weight using the protocol described in Section 2.6.2. The type of error was assigned as 0 if it was additive and 1 if it was multiplicative.

| Half Life (min) | Error (min) | Rate constant ($min^{-1}$) | Error ($min^{-1}$) | Experimental details | | | | | | | Weight | Reference |
|---|---|---|---|---|---|---|---|---|---|---|---|---|
| | | | | Experiment type | Substrate | Species | Expression Vector | pH | Temperature (°C) | Other | | |
| 20 | | 0.035 | | Unknown, assume in vitro | TXB2 | Unknown | Unknown | Unknown | Unknown | Textbook with no reference | 8 | (Wild, 2005) |

**Table ST.9.2.1.1.2.** The log-normal distribution properties of the $exTXB_2$ decay constant distribution. These values were calculated using the functions described in Section 2.6.2.

| Mode ($min^{-1}$) | Confidence Interval | Location parameter (μ) | Scale parameter (σ) |
|---|---|---|---|
| $3.48 \times 10^{-2}$ | 1.10 | -3.35 | $9.4910^{-2}$ |

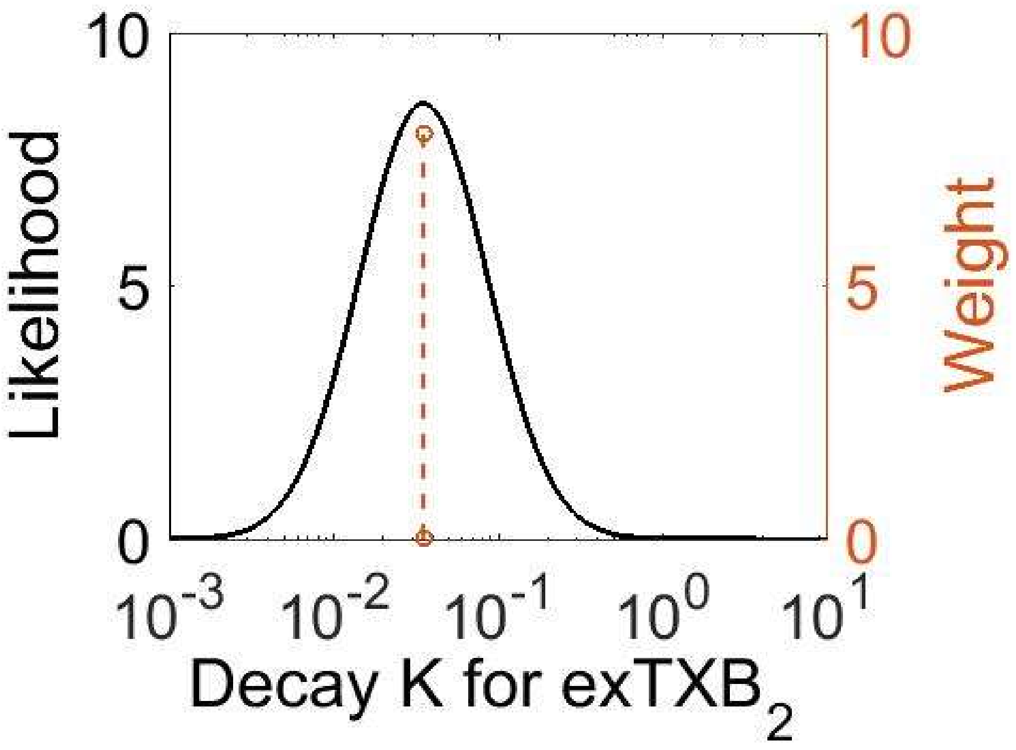


**Figure SF.9.2.1.1.1.** The estimated probability distribution for $exTXB_2$ decay constant. The value and weight of the literature values used to define the distribution are indicated by an orange dashed line. The x axis is plotted on a log-scale.

**S.9.3. Reaction 46: ex$TXA_2$ →ϕ**

The decay of $TXA_2$ in the extracellular compartment into metabolites outside of the scope of this model. An example of metabolites outside of the scope of this model includes metabolites which have been removed from the extracellular compartment into the systemic circulation or are degraded into metabolites not of interest to this model, the term decay reaction refers to the decrease in metabolite concentration by this collection of unspecified processes.

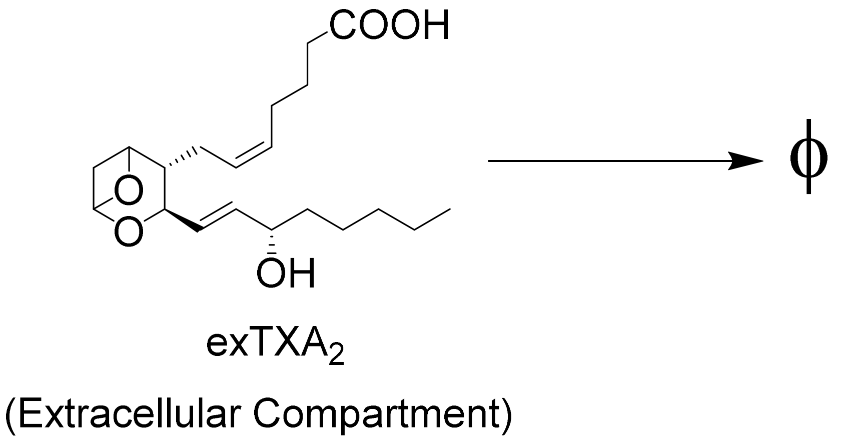


**Figure SF.9.3.** The decay of thromboxane $A_2$ ($TXA_2$) in the extracellular compartment (Reaction 46).

**SEq.9.3.** Reaction rate law for Reaction 46.

$$\boldsymbol{v_{46} = K[ex\mathrm{TXA}_2]}$$

### S.9.3.1. Reaction parameters

#### S.9.3.1.1. Parameter: K

Parameter values for the K of Reaction 46 were obtained from the literature and summarised in Table ST.9.3.1.1.1. The log-normal distribution properties for the K of Reaction 46 are shown in Table ST.9.3.1.1.2 and plotted in Figure SF.9.3.1.1.1.

**Table ST.9.3.1.1.1.** Literature information used to design the $exTXA_2$ decay constant parameter distribution. Each value was assigned a weight using the protocol described in Section 2.6.2. The type of error was assigned as 0 if it was additive and 1 if it was multiplicative.

| Half Life (min) | Error (min) | Rate constant ($min^{-1}$) | Error ($min^{-1}$) | Experimental details | | | | | | | Weight | Reference |
|---|---|---|---|---|---|---|---|---|---|---|---|---|
| | | | | Experiment type | Substrate | Species | Expression Vector | pH | Temperature (°C) | Other | | |
| 0.333 | | 2.079 | | Unknown, assume in vitro | TXA2 | Unknown | Unknown | Unknown | Unknown | Textbook with no reference | 8 | (Wild, 2005) |

**Table ST.9.3.1.1.2** The log-normal distribution properties of the $exTXA_2$ decay constant distribution. These values were calculated using the functions described in Section 2.6.2.

| Mode ($min^{-1}$) | Confidence Interval | Location parameter (μ) | Scale parameter (σ) |
|---|---|---|---|
| 2.07 | 1.10 | $7.37 \times 10^{-1}$ | $9.4910^{-2}$ |

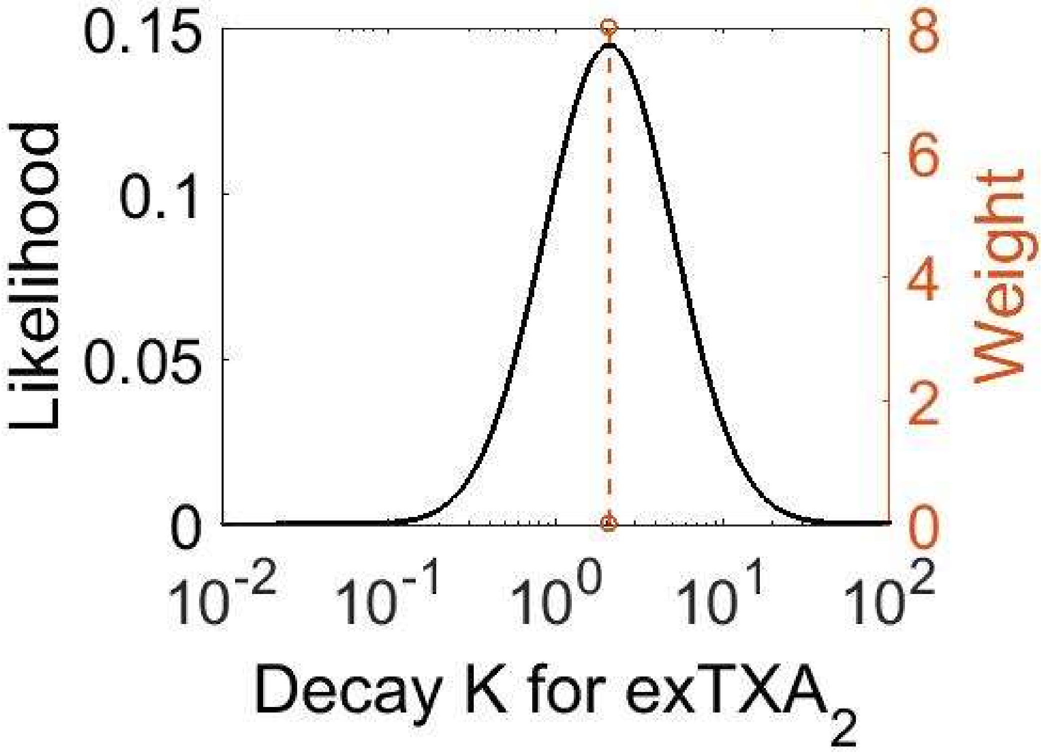


**Figure SF.9.3.1.1.1.** The estimated probability distribution for $exTXA_2$ decay constant. The value and weight of the literature values used to define the distribution are indicated by an orange dashed line. The x axis is plotted on a log-scale.

**S.9.4. Reaction 47: ex6-keto-PGF$_{1\alpha}$ →ϕ**

The decay of 6-keto-PGF$_{1\alpha}$ in the extracellular compartment into metabolites outside of the scope of this model. An example of metabolites outside of the scope of this model includes metabolites which have been removed from the extracellular compartment into the systemic circulation or are degraded into metabolites not of interest to this model, the term decay reaction refers to the decrease in metabolite concentration by this collection of unspecified processes.

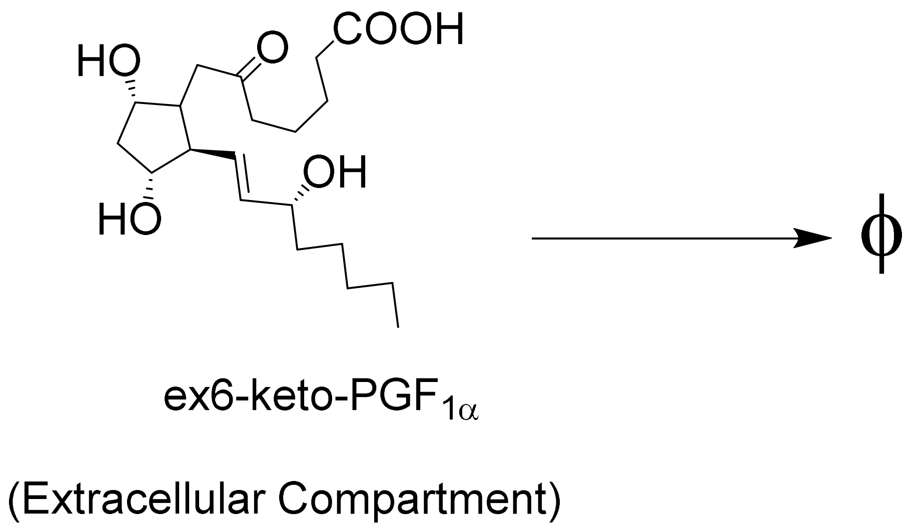


**Figure SF.9.4.** The decay of 6-keto-prostaglandin F$_{1\alpha}$ (6-keto-PGF$_{1\alpha}$) in the extracellular compartment (Reaction 47).

**SEq.9.4.** Reaction rate law for Reaction 47.

$$\boldsymbol{v_{47} = K[ex6 - keto - \mathrm{PGF}_{1\alpha}]}$$

### S.9.4.1. Reaction parameters

#### S.9.4.1.1. Parameter: K

Parameter values for the K of Reaction 47 were obtained from the literature and summarised in Table ST.9.4.1.1.1. The log-normal distribution properties for the K of Reaction 47 are shown in Table ST.9.4.1.1.2 and plotted in Figure SF.9.4.1.1.1.

**Table ST.9.4.1.1.1.** Literature information used to design the ex6-keto-$PGF_{1\alpha}$ decay constant parameter distribution. Each value was assigned a weight using the protocol described in Section 2.6.2. The type of error was assigned as 0 if it was additive and 1 if it was multiplicative.

| Half Life (min) | Error (min) | Rate constant ($min^{-1}$) | Error ($min^{-1}$) | Experimental details | | | | | | | Weight | Reference |
|---|---|---|---|---|---|---|---|---|---|---|---|---|
| | | | | Experiment type | Substrate | Species | Expression Vector | pH | Temperature (°C) | Other | | |
| 30 | | 0.023104906 | | In vivo | 6-KETO-PGF1A | Human | Plasma | Unknown | Unknown | | 8 | (Ylikorkala and Viinikka, 1981) |

**Table ST.9.4.1.1.2.** The log-normal distribution properties of the ex6-keto-$PGF_{1\alpha}$ decay constant distribution. These values were calculated using the functions described in Section 2.6.2.

| Mode ($min^{-1}$) | Confidence Interval | Location parameter (μ) | Scale parameter (σ) |
|---|---|---|---|
| 2.30 $x10^{-2}$ | 1.10 | -3.76 | 9.49 $x10^{-2}$ |

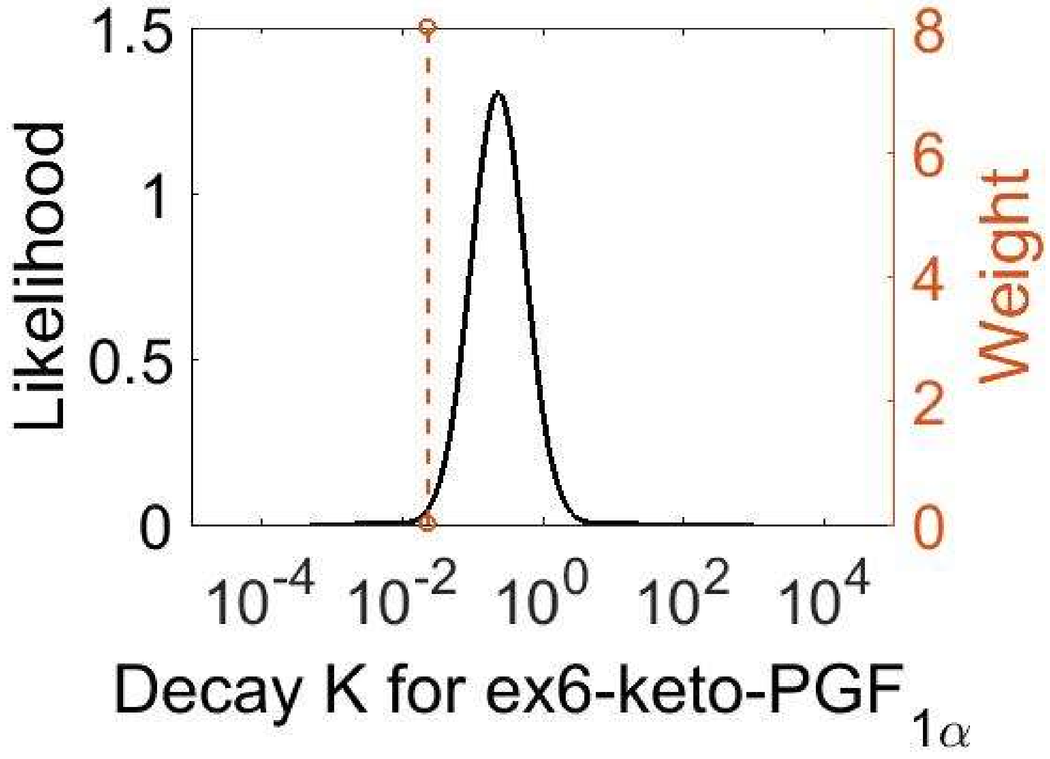


**Figure SF.9.4.1.1.1.** The estimated probability distribution for ex6-keto-$PGF_{1\alpha}$ decay constant. The value and weight of the literature values used to define the distribution are indicated by an orange dashed line. The x axis is plotted on a log-scale.

**S.9.5. Reaction 48: $exPGI_2 \rightarrow \phi$**

The decay of $PGI_2$ in the extracellular compartment into metabolites outside of the scope of this model. An example of metabolites outside of the scope of this model includes metabolites which have been removed from the extracellular compartment into the systemic circulation or are degraded into metabolites not of interest to this model, the term decay reaction refers to the decrease in metabolite concentration by this collection of unspecified processes.

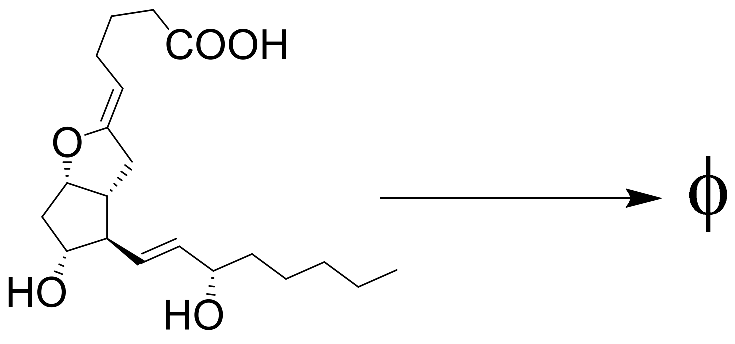


$exPGI_2$
(Extracellular Compartment)

**Figure SF.9.5.** The decay of prostaglandin $I_2$ ($PGI_2$) in the extracellular compartment (Reaction 48).

**SEq.9.5.** Reaction rate law for Reaction 48.

$$\boldsymbol{v_{48} = K[ex\mathrm{PGI}_2]}$$

### S.9.5.1. Reaction parameters

#### S.9.5.1.1. Parameter: K

Parameter values for the K of Reaction 48 were obtained from the literature and summarised in Table ST.9.5.1.1.1. The log-normal distribution properties for the K of Reaction 48 are shown in Table ST.9.5.1.1.2 and plotted in Figure SF.9.5.1.1.1.

**Table ST.9.5.1.1.1.** Literature information used to design the exPGI$_2$ decay constant parameter distribution. Each value was assigned a weight using the protocol described in Section 2.6.2. The type of error was assigned as 0 if it was additive and 1 if it was multiplicative.

| Half Life (min) | Error (min) | Rate constant (min$^{-1}$) | Error (min$^{-1}$) | Experimental details | | | | | | | Weight | Reference |
|---|---|---|---|---|---|---|---|---|---|---|---|---|
| | | | | Experiment type | Substrate | Species | Expression Vector | pH | Temperature (°C) | Other | | |
| 3 | | 0.231 | | Unknown | PGI2 | Unknown | Unknown | Unknown | Unknown | Textbook with no reference | 8 | (Cawello et al., 1994) |

**Table ST.9.5.1.1.2.** The log-normal distribution properties of the exPGI$_2$ decay constant distribution. These values were calculated using the functions described in Section 2.6.2.

| Mode (min$^{-1}$) | Confidence Interval | Location parameter (μ) | Scale parameter (σ) |
|---|---|---|---|
| 2.30 x10$^{-1}$ | 1.10 | -1.46 | 9.4910$^{-2}$ |

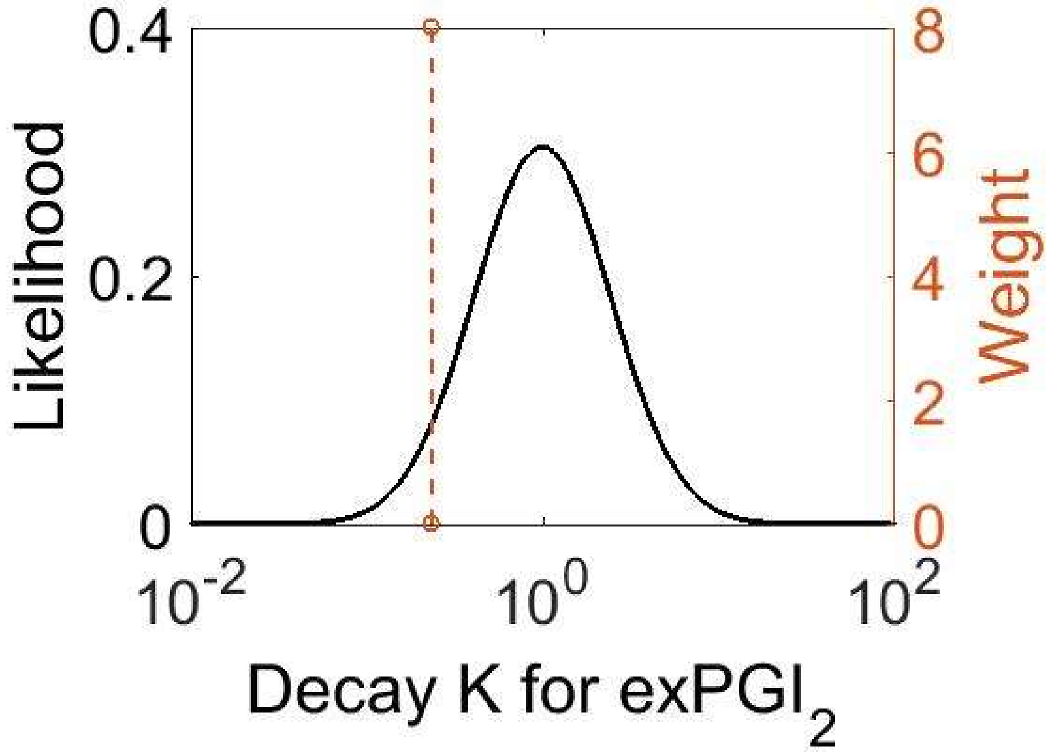


**Figure SF.9.5.1.1.1.** The estimated probability distribution for exPGI$_2$ decay constant. The value and weight of the literature values used to define the distribution are indicated by an orange dashed line. The x axis is plotted on a log-scale.

**S.9.6. Reaction 49: $exPGE_2 \rightarrow \phi$**

The decay of $PGE_2$ in the extracellular compartment into metabolites outside of the scope of this model. An example of metabolites outside of the scope of this model includes metabolites which have been removed from the extracellular compartment into the systemic circulation or are degraded into metabolites not of interest to this model, the term decay reaction refers to the decrease in metabolite concentration by this collection of unspecified processes.

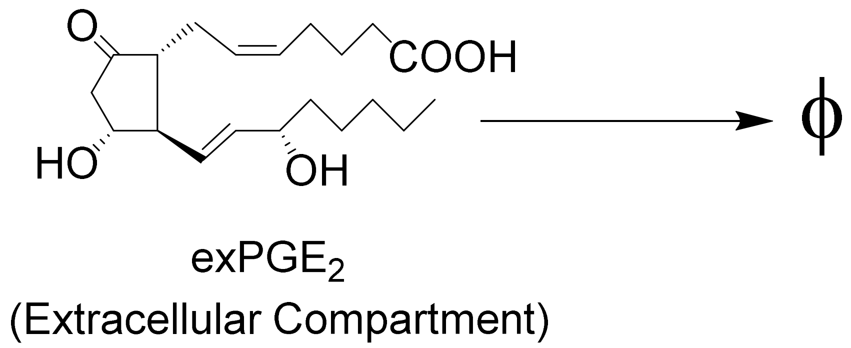


**Figure SF.9.6.** The decay of prostaglandin $E_2$ ($PGE_2$) in the extracellular compartment (Reaction 49).

**SEq.9.6.** Reaction rate law for Reaction 49.

$$\boldsymbol{v_{49} = K[exPGE_2]}$$

## S.9.6.1. Reaction parameters

### S.9.6.1.1. Parameter: K

Parameter values for the K of Reaction 49 were obtained from the literature and summarised in Table ST.9.6.1.1.1. The log-normal distribution properties for the K of Reaction 49 are shown in Table ST.9.6.1.1.2 and plotted in Figure SF.9.6.1.1.1.

**Table ST.9.6.1.1.1.** Literature information used to design the exPGE$_2$ decay constant parameter distribution. Each value was assigned a weight using the protocol described in Section 2.6.2. The type of error was assigned as 0 if it was additive and 1 if it was multiplicative.

| Half Life (min) | Error (min) | Rate constant (min$^{-1}$) | Error (min$^{-1}$) | Experimental details | | | | | | | Weight | Reference |
|---|---|---|---|---|---|---|---|---|---|---|---|---|
| | | | | Experiment type | Substrate | Species | Expression Vector | pH | Temperature (°C) | Other | | |
| 528 | 204 | 0.001 | 0.003 | In vivo | PGE2 | Human | Decidual stromal cells and macrophages | Unknown | Unknown | | 8 | (Ishihara et al., 1991) |

**Table ST.9.6.1.1.2.** The log-normal distribution properties of the exPGE$_2$ decay constant distribution. These values were calculated using the functions described in Section 2.6.2.

| Mode (min$^{-1}$) | Confidence Interval | Location parameter (μ) | Scale parameter (σ) |
|---|---|---|---|
| $3.16 \times 10^{-4}$ | 4.56 | -7.04 | 1.01 |

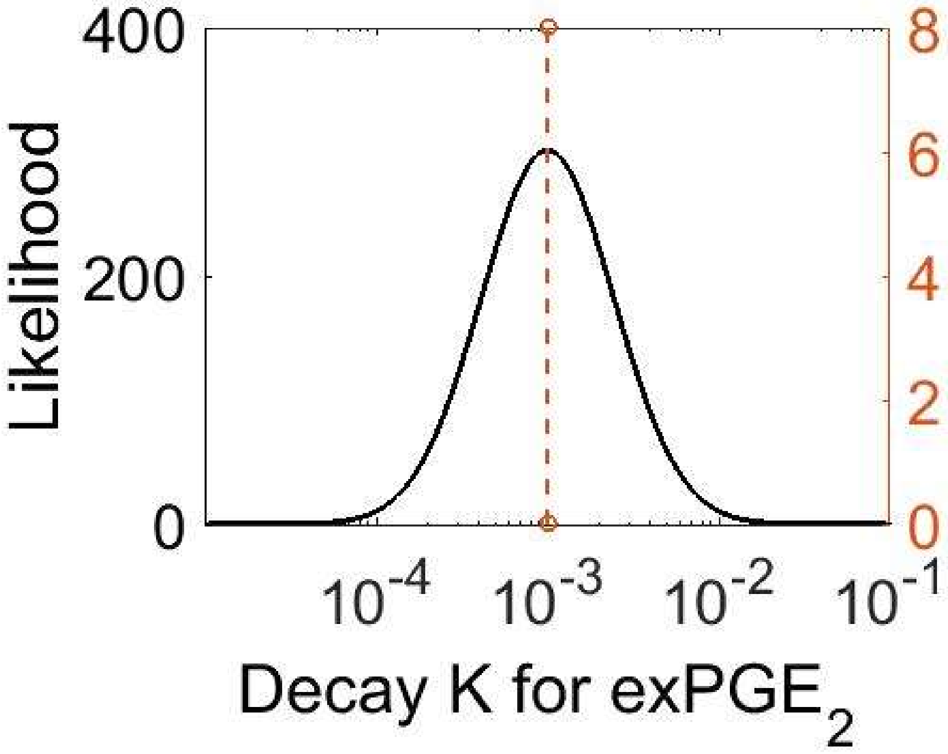


**Figure SF.9.6.1.1.1.** The estimated probability distribution for $exPGE_2$ decay constant. The value and weight of the literature values used to define the distribution are indicated by an orange dashed line. The x axis is plotted on a log-scale.

**S.9.7. Reaction 50: ex15-deoxy-PGJ$_2$ →ϕ**

The decay of 15-deoxy-PGJ$_2$ in the extracellular compartment into metabolites outside of the scope of this model. An example of metabolites outside of the scope of this model includes metabolites which have been removed from the extracellular compartment into the systemic circulation or are degraded into metabolites not of interest to this model, the term decay reaction refers to the decrease in metabolite concentration by this collection of unspecified processes.

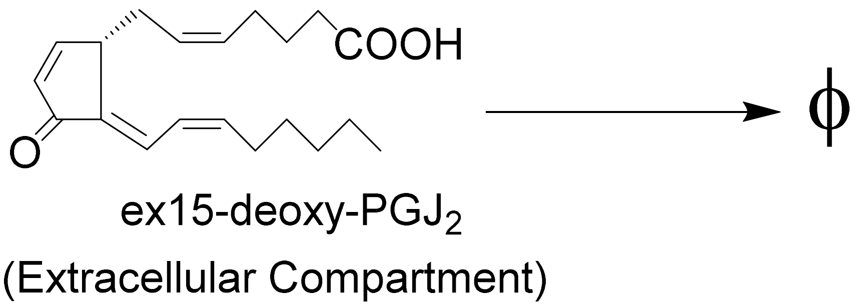


**Figure SF.9.7** The decay of 15-deoxy-prostaglandin J$_2$ (15-deoxy-PGJ$_2$) in the extracellular compartment (Reaction 50).

**SEq.9.7.** Reaction rate law for Reaction 50.

$$\boldsymbol{v_{50} = K[ex15 - \mathrm{deoxy} - \mathrm{PGJ_2}]}$$

### S.9.7.1. Reaction parameters

#### S.9.7.1.1. Parameter: K

Parameter values for the K of Reaction 50 were obtained from the literature and summarised in Table ST.9.7.1.1.1. The log-normal distribution properties for the K of Reaction 50 are shown in Table ST.9.7.1.1.2 and plotted in Figure SF.9.7.1.1.1.

**Table ST.9.7.1.1.1.** Literature information used to design the ex15-deoxy-$PGJ_2$ decay constant parameter distribution. Each value was assigned a weight using the protocol described in Section 2.6.2. The type of error was assigned as 0 if it was additive and 1 if it was multiplicative.

| Half Life (min) | Error (min) | Rate constant ($min^{-1}$) | Error ($min^{-1}$) | Experimental details | | | | | | | Weight | Reference |
|---|---|---|---|---|---|---|---|---|---|---|---|---|
| | | | | Experiment type | Substrate | Species | Expression Vector | pH | Temperature (°C) | Other | | |
| 720 | | 0.001 | | In vivo | 15-Deoxy-PGJ2 | Human | Albumin | 7.4 | 37 | | 8 | (Fitzpatrick and Wynalda, 1983) |

**Table ST.9.7.1.1.2.** The log-normal distribution properties of the ex15-deoxy-$PGJ_2$ decay constant distribution. These values were calculated using the functions described in Section 2.6.2.

| Mode ($min^{-1}$) | Confidence Interval | Location parameter (μ) | Scale parameter (σ) |
|---|---|---|---|
| $9.95 \times 10^{-4}$ | 1.10 | -6.90 | $9.4910^{-2}$ |

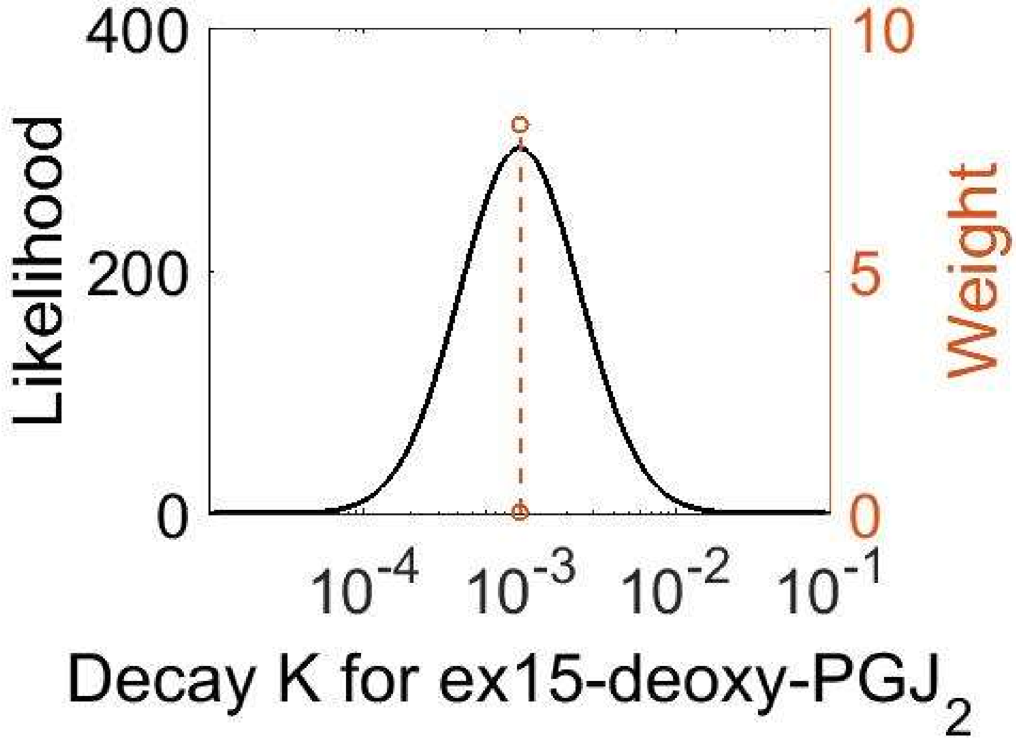


**Figure SF.9.7.1.1.1.** The estimated probability distribution for ex15-deoxy-$PGJ_2$ decay constant. The value and weight of the literature values used to define the distribution are indicated by an orange dashed line. The x axis is plotted on a log-scale.

**S.9.8. Reaction 51: exPGJ₂ →ϕ**

The decay of $PGJ_2$ in the extracellular compartment into metabolites outside of the scope of this model. An example of metabolites outside of the scope of this model includes metabolites which have been removed from the extracellular compartment into the systemic circulation or are degraded into metabolites not of interest to this model, the term decay reaction refers to the decrease in metabolite concentration by this collection of unspecified processes.

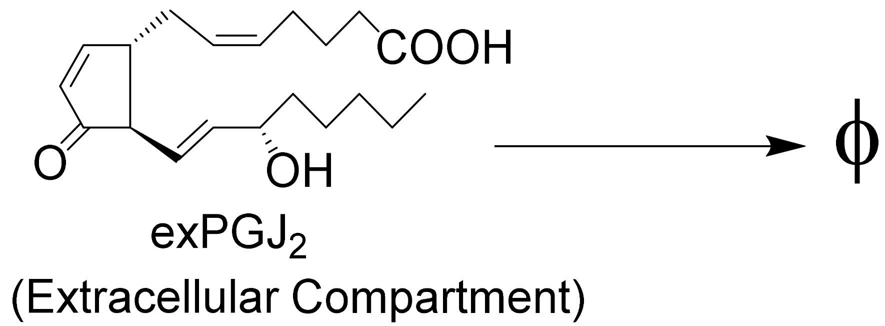


**Figure SF.9.8.** The decay of prostaglandin $J_2$ ($PGJ_2$) in the extracellular compartment (Reaction 51).

**SEq.9.8.** Reaction rate law for Reaction 51.

$$\boldsymbol{v_{51}} = \boldsymbol{K}[\boldsymbol{ex}\mathbf{PGJ_2}\ ]$$

### S.9.8.1. Reaction parameters

#### S.9.8.1.1. Parameter: K

Parameter values for the K of Reaction 51 were obtained from the literature and summarised in Table ST.9.8.1.1.1. The log-normal distribution properties for the K of Reaction 51 are shown in Table ST.9.8.1.1.2 and plotted in Figure SF.9.8.1.1.1.

**Table ST.9.8.1.1.1.** Literature information used to design the exPGJ$_2$ decay constant parameter distribution. Each value was assigned a weight using the protocol described in Section 2.6.2. The type of error was assigned as 0 if it was additive and 1 if it was multiplicative.

| Half Life (min) | Error (min) | Rate constant (min$^{-1}$) | Error (min$^{-1}$) | Experimental details | | | | | | | Weight | Reference |
|---|---|---|---|---|---|---|---|---|---|---|---|---|
| | | | | Experiment type | Substrate | Species | Expression Vector | pH | Temperature (°C) | Other | | |
| 720 | | 0.001 | | In vivo | **15-Deoxy-PGJ2** | Human | Albumin | 7.4 | 37 | | 8 | (Fitzpatrick and Wynalda, 1983) |

**Table ST.9.8.1.1.2.** The log-normal distribution properties of the exPGJ$_2$ decay constant distribution. These values were calculated using the functions described in Section 2.6.2.

| Mode (min$^{-1}$) | Confidence Interval | Location parameter (μ) | Scale parameter (σ) |
|---|---|---|---|
| 9.95 x10$^{-4}$ | 1.10 | -6.90 | 9.4910$^{-2}$ |

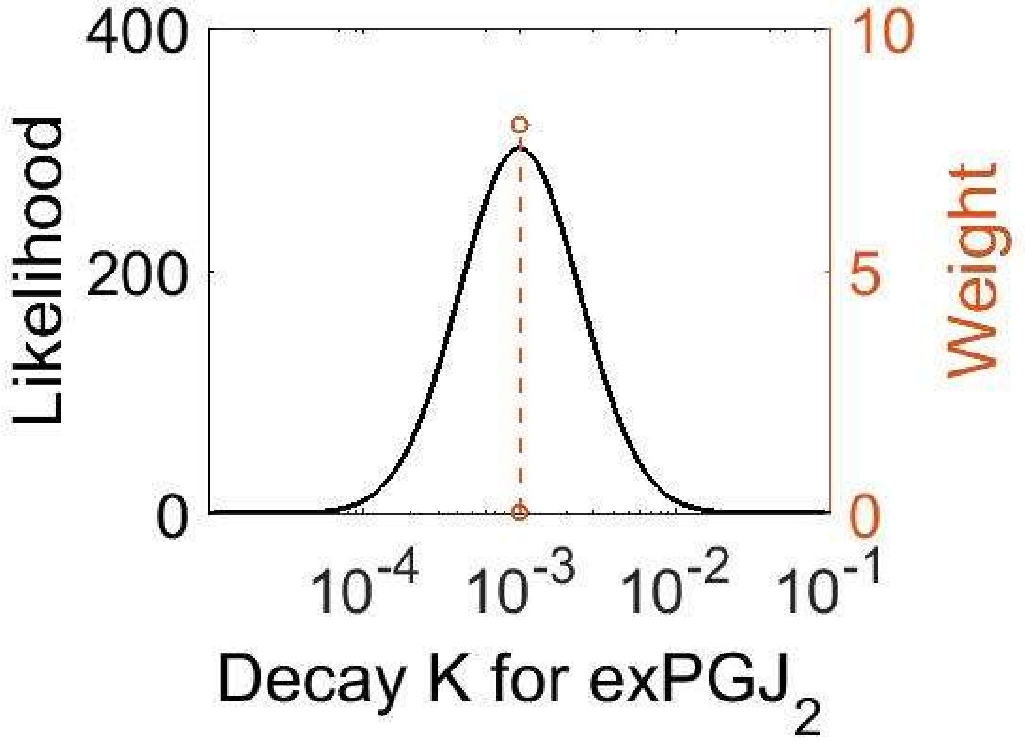


**Figure SF.9.8.1.1.1.** The estimated probability distribution for $exPGJ_2$ decay constant. The value and weight of the literature values used to define the distribution are indicated by an orange dashed line. The x axis is plotted on a log-scale.

**S.9.9. Reaction 52: exPGD$_2$ →ϕ**

The decay of PGD$_2$ in the extracellular compartment into metabolites outside of the scope of this model. An example of metabolites outside of the scope of this model includes metabolites which have been removed from the extracellular compartment into the systemic circulation or are degraded into metabolites not of interest to this model, the term decay reaction refers to the decrease in metabolite concentration by this collection of unspecified processes.

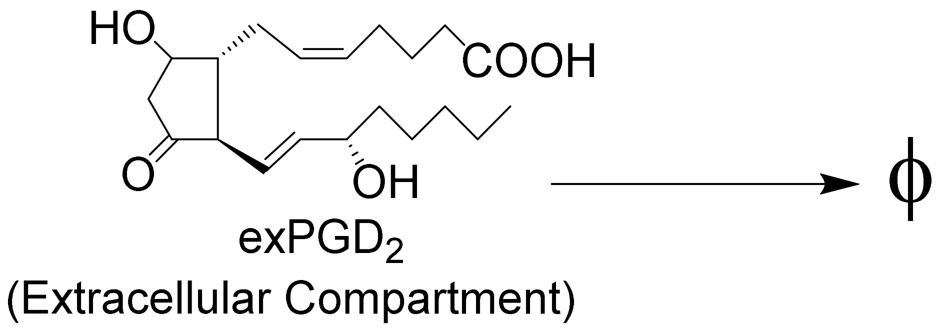


**Figure SF.9.9.** The decay of prostaglandin D$_2$ (PGD$_2$) in the extracellular compartment (Reaction 52).

**SEq.9.9.** Reaction rate law for Reaction 52.

$$\boldsymbol{v_{52} = K[ex\mathrm{PGD}_2]}$$

### S.9.9.1. Reaction parameters

#### S.9.9.1.1. Parameter: K

Parameter values for the K of Reaction 52 were obtained from the literature and summarised in Table ST.9.9.1.1.1. The log-normal distribution properties for the K of Reaction 52 are shown in Table ST.9.9.1.1.2 and plotted in Figure SF.9.9.1.1.1.

**Table ST.9.9.1.1.1.** Literature information used to design the $exPGD_2$ decay constant parameter distribution. Each value was assigned a weight using the protocol described in Section 2.6.2. The type of error was assigned as 0 if it was additive and 1 if it was multiplicative.

| Half Life (min) | Error (min) | Rate constant ($min^{-1}$) | Error ($min^{-1}$) | Experimental details | | | | | | | Weight | Reference |
|---|---|---|---|---|---|---|---|---|---|---|---|---|
| | | | | Experiment type | Substrate | Species | Expression Vector | pH | Temperature (°C) | Other | | |
| 30 | | 0.023 | | In vivo | PGD2 | Human | Plasma | 7 | 37 | | 8 | (Schuligoi et al., 2007) |
| 1.5 | | 0.462 | | In vivo | PGD2 | Human | Brain | 7.4 | Unknown | | 8 | (Suzuki et al., 1986) |

**Table ST.9.9.1.1.2.** The log-normal distribution properties of the $exPGD_2$ decay constant distribution. These values were calculated using the functions described in Section 2.6.2.

| Mode ($min^{-1}$) | Confidence Interval | Location parameter (μ) | Scale parameter (σ) |
|---|---|---|---|
| $2.30 \times 10^{-2}$ | 1.10 | -3.76 | $9.4910^{-2}$ |

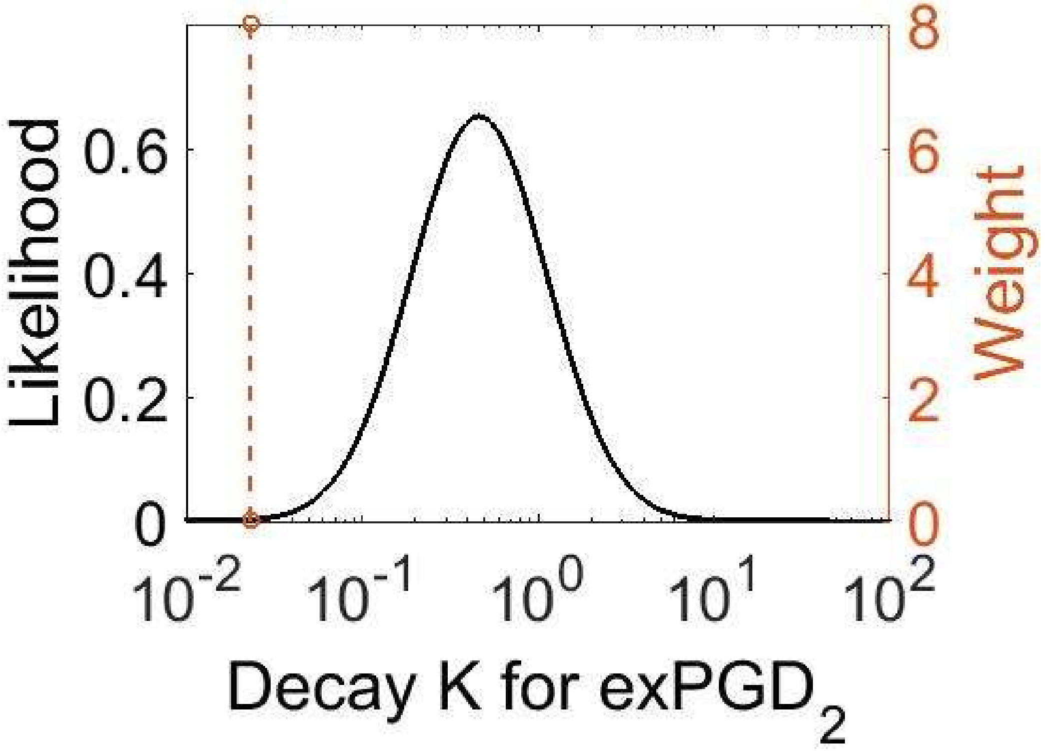


**Figure SF.9.9.1.1.1.** The estimated probability distribution for exPGD2 decay constant. The value and weight of the literature values used to define the distribution are indicated by an orange dashed line. The x axis is plotted on a log-scale.

**S.9.10. Reaction 53: $exPGH_2 \rightarrow \phi$**

The decay of $PGH_2$ in the extracellular compartment into metabolites outside of the scope of this model. An example of metabolites outside of the scope of this model includes metabolites which have been removed from the extracellular compartment into the systemic circulation or are degraded into metabolites not of interest to this model, the term decay reaction refers to the decrease in metabolite concentration by this collection of unspecified processes.

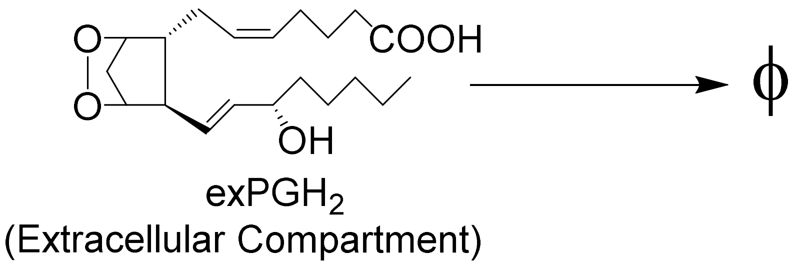


**Figure SF.9.10.** The decay of prostaglandin $H_2$ ($PGH_2$) in the extracellular compartment (Reaction 53).

**SEq.9.10.** Reaction rate law for Reaction 53.

$$\boldsymbol{v_{53} = K[exPGH_2]}$$

### S.9.10.1. Reaction parameters

#### S.9.10.1.1. Parameter: K

Parameter values for the K of Reaction 53 were obtained from the literature and summarised in Table ST.9.10.1.1.1. The log-normal distribution properties for the K of Reaction 53 are shown in Table ST.9.10.1.1.2 and plotted in Figure SF.9.10.1.1.1.

**Table ST.9.10.1.1.1.** Literature information used to design the $exPGH_2$ decay constant parameter distribution. Each value was assigned a weight using the protocol described in Section 2.6.2. The type of error was assigned as 0 if it was additive and 1 if it was multiplicative.

| Half Life (min) | Error (min) | Rate constant ($min^{-1}$) | Error ($min^{-1}$) | Experimental details | | | | | | | Weight | References |
|---|---|---|---|---|---|---|---|---|---|---|---|---|
| | | | | Experiment type | Substrate | Species | Expression Vector | pH | Temperature (°C) | Other | | |
| 5 | | 0.139 | | In vivo | PGH2 | Unknown | Unknown | Unknown | Unknown | Textbook with no reference | 8 | (Sciences, 2019) |

**Table ST.9.10.1.1.2.** The log-normal distribution properties of the $exPGH_2$ decay constant distribution. These values were calculated using the functions described in Section 2.6.2.

| Mode ($min^{-1}$) | Confidence Interval | Location parameter (μ) | Scale parameter (σ) |
|---|---|---|---|
| $1.38 \times 10^{-1}$ | 1.10 | -1.97 | $9.49 \times 10^{-2}$ |

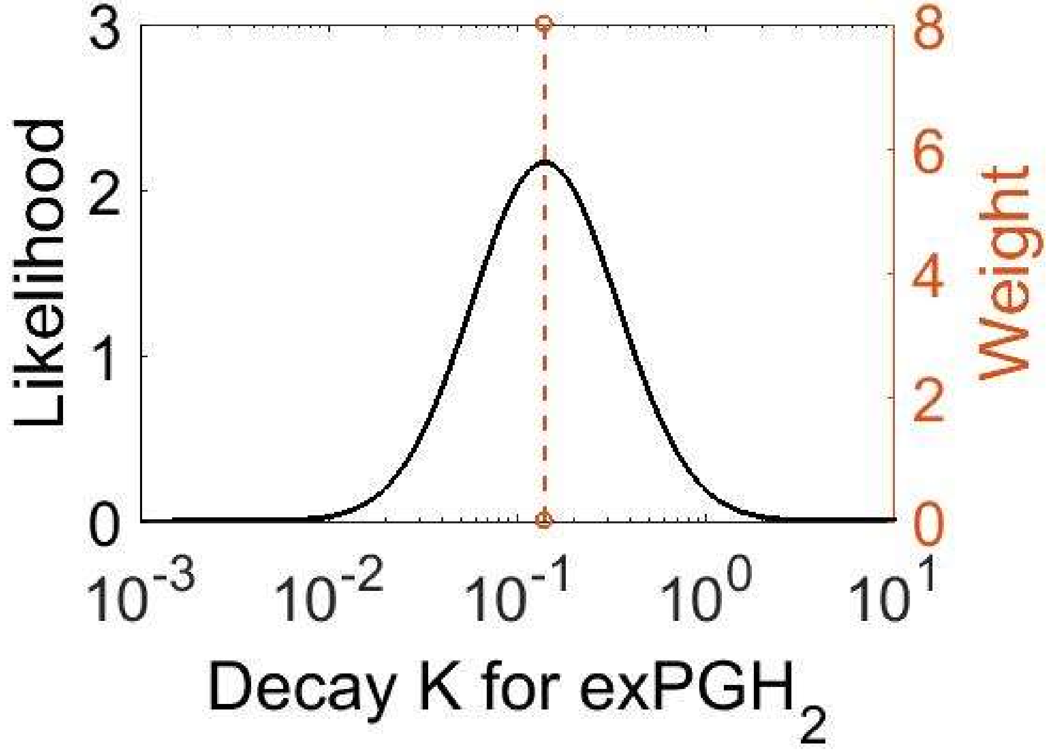


**Figure SF.9.10.1.1.1.** The estimated probability distribution for $exPGH_2$ decay constant. The value and weight of the literature values used to define the distribution are indicated by an orange dashed line. The x axis is plotted on a log-scale.

**S.9.11. Reaction 54: ex5-oxo-ETE →ϕ**

The decay of 5-oxo-ETE in the extracellular compartment into metabolites outside of the scope of this model. An example of metabolites outside of the scope of this model includes metabolites which have been removed from the extracellular compartment into the systemic circulation or are degraded into metabolites not of interest to this model, the term decay reaction refers to the decrease in metabolite concentration by this collection of unspecified processes.

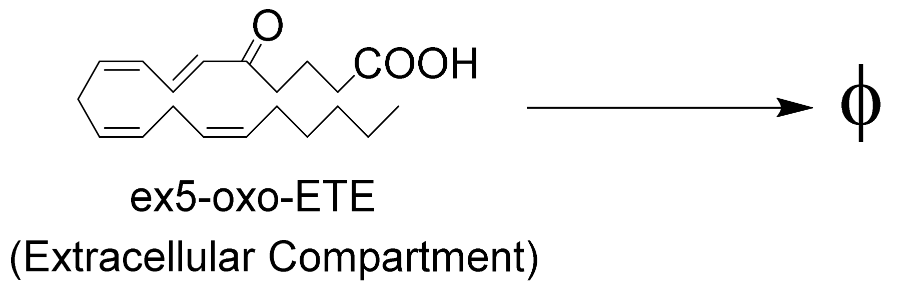


**Figure SF.9.11.** The decay of 5-oxo-eicosatetraenoic acid (5-oxo-ETE) in the extracellular compartment (Reaction 54).

**SEq.9.11.** Reaction rate law for Reaction 54.

$$\boldsymbol{v_{54}} = \boldsymbol{K}[\boldsymbol{ex}\mathbf{5} - \mathbf{oxo} - \mathbf{ETE}]$$

### S.9.11.1. Reaction parameters

### S.9.11.1. Parameter: K

Parameter values for the K of Reaction 54 were obtained from the literature and summarised in Table ST.9.11.1.1.1. The log-normal distribution properties for the K of Reaction 54 are shown in Table ST.9.11.1.1.2 and plotted in Figure SF.9.11.1.1.1.

**Table ST.9.11.1.1.1.** Literature information used to design the ex5-oxo-ETE decay constant parameter distribution. Each value was assigned a weight using the protocol described in Section 2.6.2. The type of error was assigned as 0 if it was additive and 1 if it was multiplicative.

| Half Life (min) | Error (min) | Rate constant ($min^{-1}$) | Error ($min^{-1}$) | Experimental details | | | | | | | Weight | References |
|---|---|---|---|---|---|---|---|---|---|---|---|---|
| | | | | Experiment type | Substrate | Species | Expression Vector | pH | Temperature (°C) | Other | | |
| 11 | | 0.064 | | In vivo | **15-OXO-ETE** | Human | R15L Cells | Unknown | 37 | | 8 | (Wei et al., 2009) |

**Table ST.9.11.1.1.2.** The log-normal distribution properties of the ex5-oxo-ETE decay constant distribution. These values were calculated using the functions described in Section 2.6.2.

| Mode ($min^{-1}$) | Confidence Interval | Location parameter (μ) | Scale parameter (σ) |
|---|---|---|---|
| 6.37 $x10^{-2}$ | 1.10 | -2.74 | $9.4910^{-2}$ |

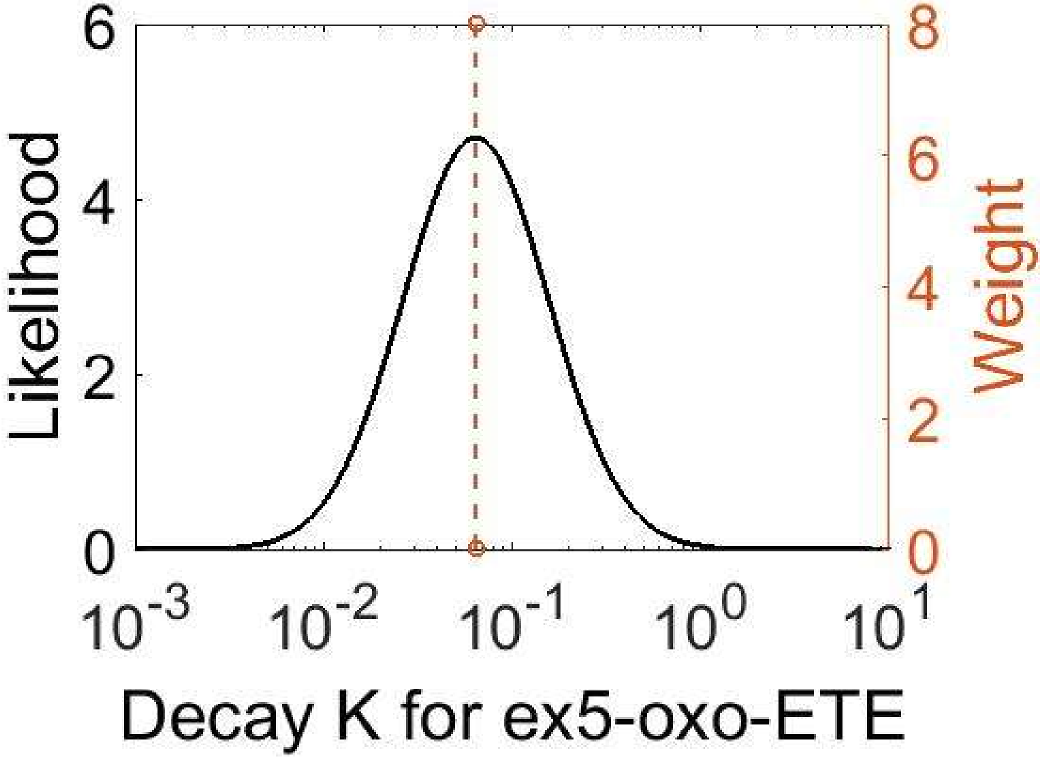


**Figure SF.9.11.1.1.1.** The estimated probability distribution for ex5-oxo-ETE decay constant. The value and weight of the literature values used to define the distribution are indicated by an orange dashed line. The x axis is plotted on a log-scale.

**S.9.12. Reaction 55: ex5-HETE →ϕ**

The decay of 5-HETE in the extracellular compartment into metabolites outside of the scope of this model. An example of metabolites outside of the scope of this model includes metabolites which have been removed from the extracellular compartment into the systemic circulation or are degraded into metabolites not of interest to this model, the term decay reaction refers to the decrease in metabolite concentration by this collection of unspecified processes.

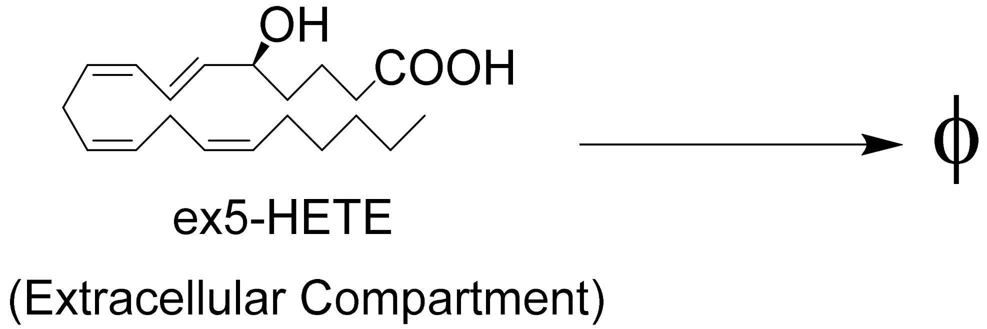


**Figure SF.9.12.** The decay of 5-hydroxyeicosatetraenoic acid (5-HETE) in the extracellular compartment (Reaction 55).

**SEq.9.12.** Reaction rate law for Reaction 55.

$\boldsymbol{v_{55} = K[ex5 - \mathrm{HETE}]}$

### S.9.11.1. Reaction parameters

#### S.9.11.1.1. Parameter: K

Parameter values for the K of Reaction 55 were obtained from the literature and summarised in Table ST.9.11.1.1.1. The log-normal distribution properties for the K of Reaction 55 are shown in Table ST.9.11.1.1.2 and plotted in Figure SF.9.11.1.1.1.

**Table ST.9.11.1.1.1.** Literature information used to design the ex5-HETE decay constant parameter distribution. Each value was assigned a weight using the protocol described in Section 2.6.2. The type of error was assigned as 0 if it was additive and 1 if it was multiplicative.

| Half Life (min) | Error (min) | Rate constant ($min^{-1}$) | Error ($min^{-1}$) | Experimental details | | | | | | | Weight | References |
|---|---|---|---|---|---|---|---|---|---|---|---|---|
| | | | | Experiment type | Substrate | Species | Expression Vector | pH | Temperature (°C) | Other | | |
| 21 | | 0.033 | | In vivo | **15-HETE** | Human | R15L Cells | Unknown | 37 | | 8 | (Wei et al., 2009) |

**Table ST.9.11.1.1.2.** The log-normal distribution properties of the ex5-HETE decay constant distribution. These values were calculated using the functions described in Section 2.6.2.

| Mode ($min^{-1}$) | Confidence Interval | Location parameter (μ) | Scale parameter (σ) |
|---|---|---|---|
| 3.30 $x10^{-2}$ | 1.10 | -3.40 | $9.4910^{-2}$ |

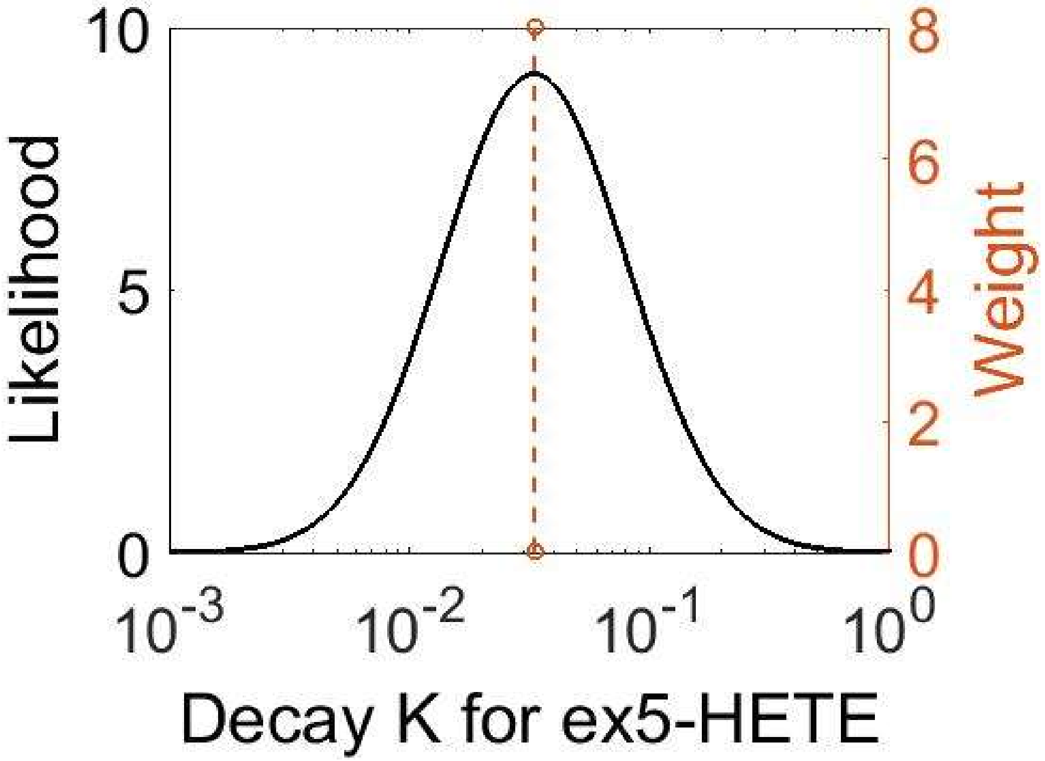


**Figure SF.9.11.1.1.1.** The estimated probability distribution for ex5-HETE decay constant. The value and weight of the literature values used to define the distribution are indicated by an orange dashed line. The x axis is plotted on a log-scale.

**S.9.12. Reaction 56: exLTB4 →ϕ**

The decay of LTB4 in the extracellular compartment into metabolites outside of the scope of this model. An example of metabolites outside of the scope of this model includes metabolites which have been removed from the extracellular compartment into the systemic circulation or are degraded into metabolites not of interest to this model, the term decay reaction refers to the decrease in metabolite concentration by this collection of unspecified processes.

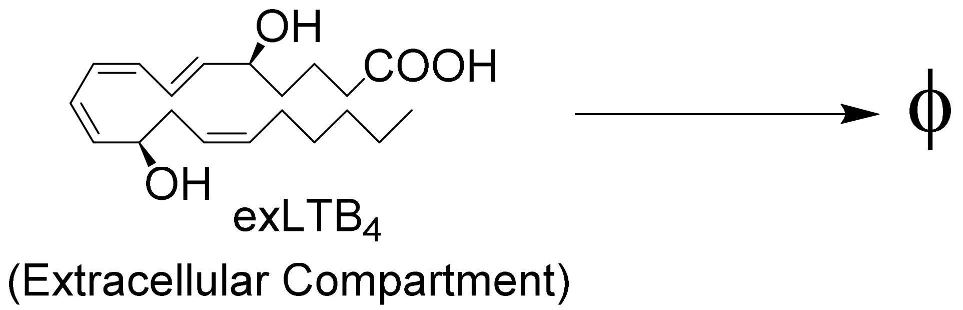


**Figure SF.9.12.** The decay of leukotriene B4 (LTB4) in the extracellular compartment (Reaction 56).

**SEq.9.12.** Reaction rate law for Reaction 56.

$\boldsymbol{v_{56} = K[ex\mathrm{LTB}_4]}$

## S.9.12.1. Reaction parameters

### S.9.12.1.1. Parameter: K

Parameter values for the K of Reaction 56 were obtained from the literature and summarised in Table ST.9.12.1.1.1. The log-normal distribution properties for the K of Reaction 56 are shown in Table ST.9.12.1.1.2 and plotted in Figure SF.9.12.1.1.1.

**Table ST.9.12.1.1.1.** Literature information used to design the exLTB4 decay constant parameter distribution. Each value was assigned a weight using the protocol described in Section 2.6.2. The type of error was assigned as 0 if it was additive and 1 if it was multiplicative.

| Half Life (min) | Error (min) | Rate constant ($min^{-1}$) | Error ($min^{-1}$) | Experimental details | | | | | | | Weight | References |
|---|---|---|---|---|---|---|---|---|---|---|---|---|
| | | | | Experiment type | Substrate | Species | Expression Vector | pH | Temperature (°C) | Other | | |
| 0.47 | | 1.475 | | In vivo | LTB4 | Rabbit | Plasma | 7.4 | Unknown | | 8 | (Marleau et al., 1994) |

**Table ST.9.12.1.1.2.** The log-normal distribution properties of the exLTB4 decay constant distribution. These values were calculated using the functions described in Section 2.6.2.

| Mode ($min^{-1}$) | Confidence Interval | Location parameter (μ) | Scale parameter (σ) |
|---|---|---|---|
| 1.47 | 1.10 | $3.93 \times 10^{-1}$ | $9.4910^{-2}$ |

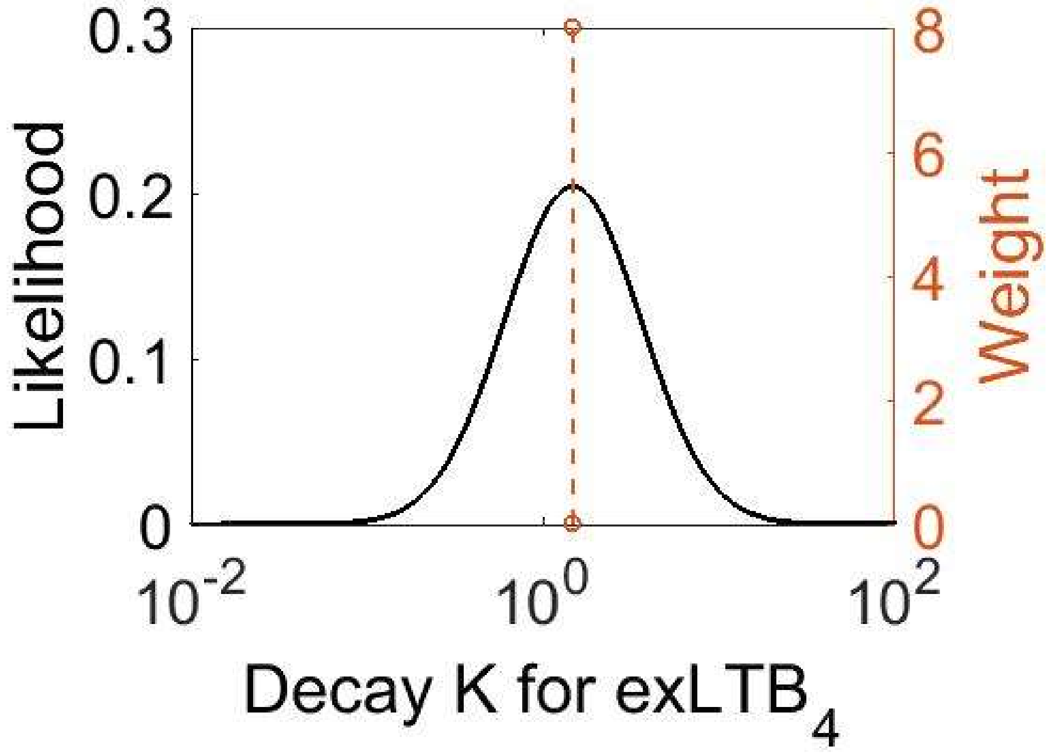


**Figure SF.9.12.1.1.1.** The estimated probability distribution for exLTB$_4$ decay constant. The value and weight of the literature values used to define the distribution are indicated by an orange dashed line. The x axis is plotted on a log-scale.

**S.9.13. Reaction 57: $exLTC_4 \rightarrow \phi$**

The decay of $LTC_4$ in the extracellular compartment into metabolites outside of the scope of this model. An example of metabolites outside of the scope of this model includes metabolites which have been removed from the extracellular compartment into the systemic circulation or are degraded into metabolites not of interest to this model, the term decay reaction refers to the decrease in metabolite concentration by this collection of unspecified processes.

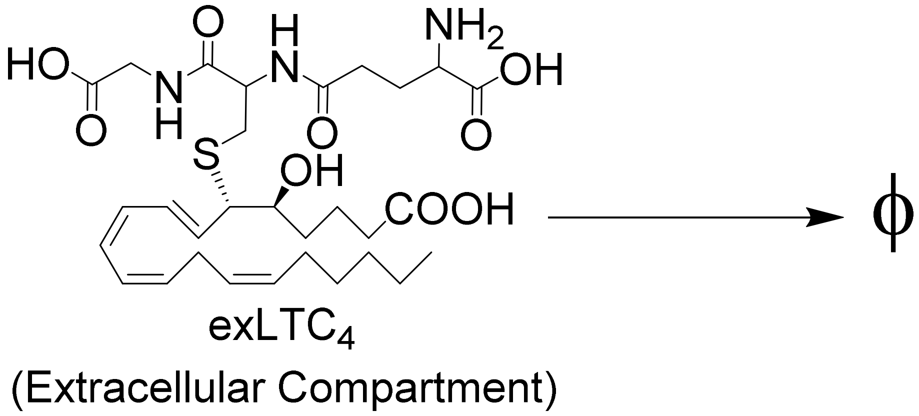


**Figure SF.9.13.** The decay of leukotriene $C_4$ ($LTC_4$) in the extracellular compartment (Reaction 57).

**SEq.9.13.** Reaction rate law for Reaction 57.

$$\boldsymbol{v_{57} = K[exLTC_4]}$$

### S.9.13.1. Reaction parameters

#### S.9.13.1.1. Parameter: K

Parameter values for the K of Reaction 57 were obtained from the literature and summarised in Table ST.9.13.1.1.1. The log-normal distribution properties for the K of Reaction 57 are shown in Table ST.9.13.1.1.2 and plotted in Figure SF.9.13.1.1.1.

**Table ST.9.13.1.1.1.** Literature information used to design the exLTC$_4$ decay constant parameter distribution. Each value was assigned a weight using the protocol described in Section 2.6.2. The type of error was assigned as 0 if it was additive and 1 if it was multiplicative.

| Half Life (min) | Error (min) | Rate constant (min$^{-1}$) | Error (min$^{-1}$) | Experimental details | | | | | | | Weight | References |
|---|---|---|---|---|---|---|---|---|---|---|---|---|
| | | | | Experiment type | Substrate | Species | Expression Vector | pH | Temperature (°C) | Other | | |
| 0.47 | | 1.475 | | In vivo | **LTB4** | Rabbit | Plasma | 7.4 | Unknown | | 8 | (Marleau et al., 1994) |

**Table ST.9.13.1.1.2.** The log-normal distribution properties of the exLTC$_4$ decay constant distribution. These values were calculated using the functions described in Section 2.6.2.

| Mode (min$^{-1}$) | Confidence Interval | Location parameter (μ) | Scale parameter (σ) |
|---|---|---|---|
| 1.47 | 1.10 | 3.93 x10$^{-1}$ | 9.4910$^{-2}$ |

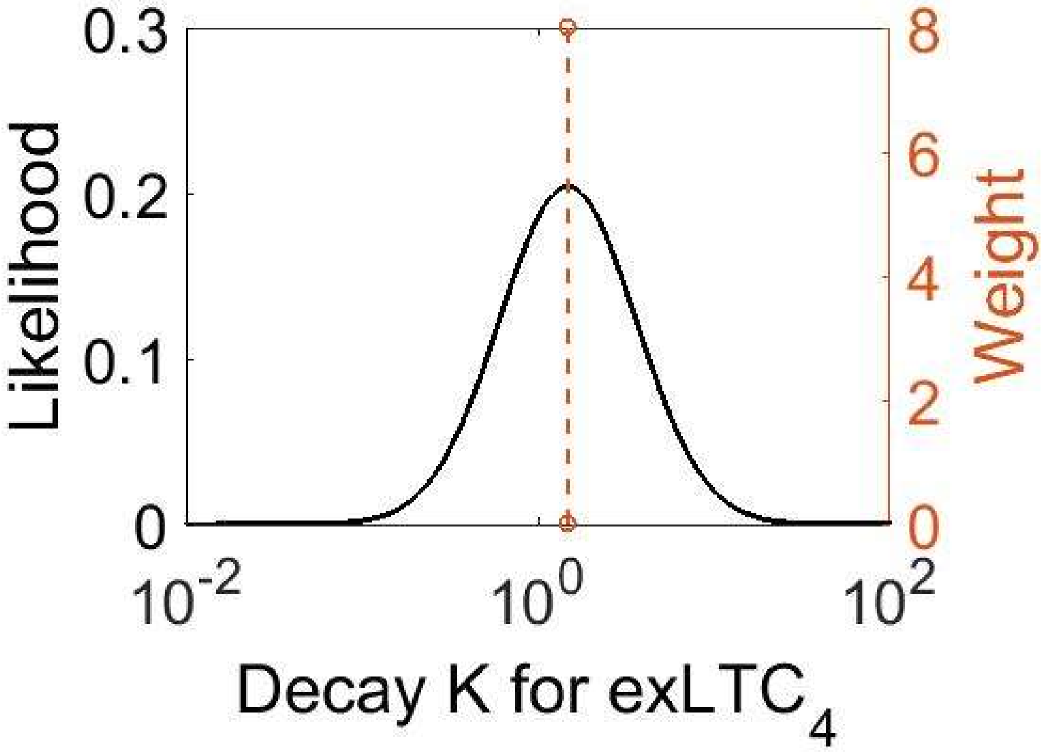


**Figure SF.9.13.1.1.1.** The estimated probability distribution for $exLTC_4$ decay constant. The value and weight of the literature values used to define the distribution are indicated by an orange dashed line. The x axis is plotted on a log-scale.

**S.9.14. Reaction 58: $exLTA_4 \rightarrow \phi$**

The decay of $LTA_4$ in the extracellular compartment into metabolites outside of the scope of this model. An example of metabolites outside of the scope of this model includes metabolites which have been removed from the extracellular compartment into the systemic circulation or are degraded into metabolites not of interest to this model, the term decay reaction refers to the decrease in metabolite concentration by this collection of unspecified processes.

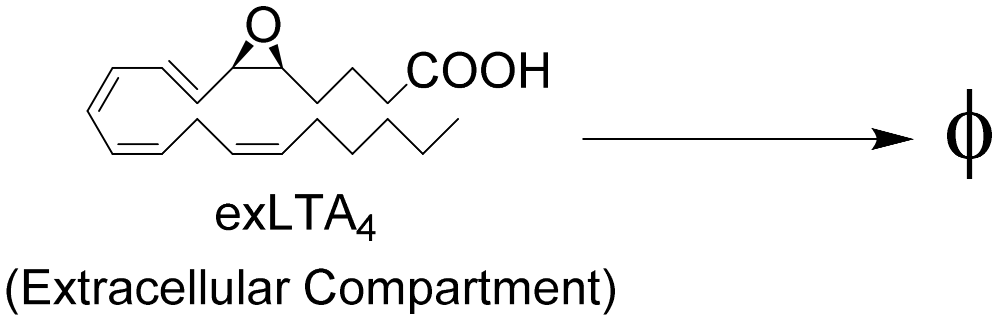


**Figure SF.9.14.** The decay of leukotriene $A_4$ ($LTA_4$) in the extracellular compartment (Reaction 58).

**SEq.9.14.** Reaction rate law for Reaction 58.

$$\boldsymbol{v_{58} = K[exLTA_4]}$$

### S.9.14.1. Reaction parameters

#### S.9.14.1.1. Parameter: K

Parameter values for the K of Reaction 58 were obtained from the literature and summarised in Table ST.9.14.1.1.1. The log-normal distribution properties for the K of Reaction 58 are shown in Table ST.9.14.1.1.2 and plotted in Figure SF.9.14.1.1.1.

**Table ST.9.14.1.1.1.** Literature information used to design the exLTA$_4$ decay constant parameter distribution. Each value was assigned a weight using the protocol described in Section 2.6.2. The type of error was assigned as 0 if it was additive and 1 if it was multiplicative.

| Half Life (min) | Error (min) | Rate constant (min$^{-1}$) | Error (min$^{-1}$) | Experimental details | | | | | | | Weight | References |
|---|---|---|---|---|---|---|---|---|---|---|---|---|
| | | | | Experiment type | Substrate | Species | Expression Vector | pH | Temperature (°C) | Other | | |
| 0.05 | | 13.863 | | In vivo | LTA4 | Unknown | Unknown | 7 | 37 | | 8 | (Dickinson Zimmer et al., 2004) |

**Table ST.9.14.1.1.2.** The log-normal distribution properties of the exLTA$_4$ decay constant distribution. These values were calculated using the functions described in Section 2.6.2.

| Mode (min$^{-1}$) | Confidence Interval | Location parameter (μ) | Scale parameter (σ) |
|---|---|---|---|
| 1.38 x10$^{1}$ | 1.10 | 2.63 | 9.4910$^{-2}$ |

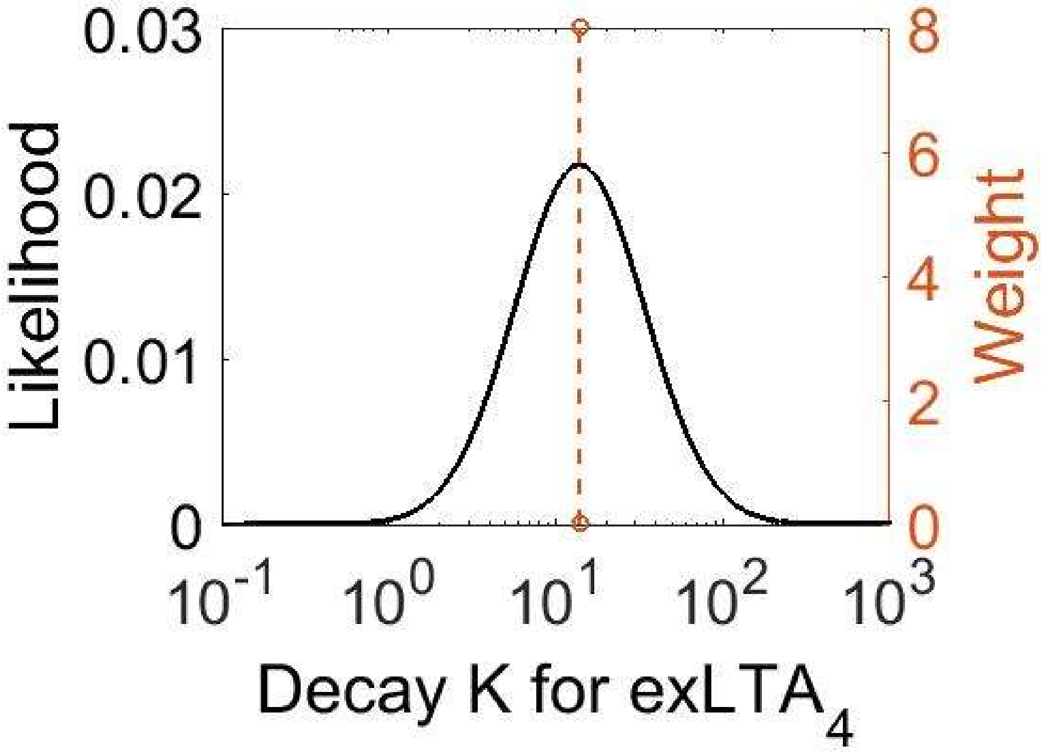


**Figure SF.9.14.1.1.1.** The estimated probability distribution for exLTA₄ decay constant. The value and weight of the literature values used to define the distribution are indicated by an orange dashed line. The x axis is plotted on a log-scale.

**S.9.15. Reaction 59: ex5-HPETE →ϕ**

The decay of 5-HPETE in the extracellular compartment into metabolites outside of the scope of this model. An example of metabolites outside of the scope of this model includes metabolites which have been removed from the extracellular compartment into the systemic circulation or are degraded into metabolites not of interest to this model, the term decay reaction refers to the decrease in metabolite concentration by this collection of unspecified processes.

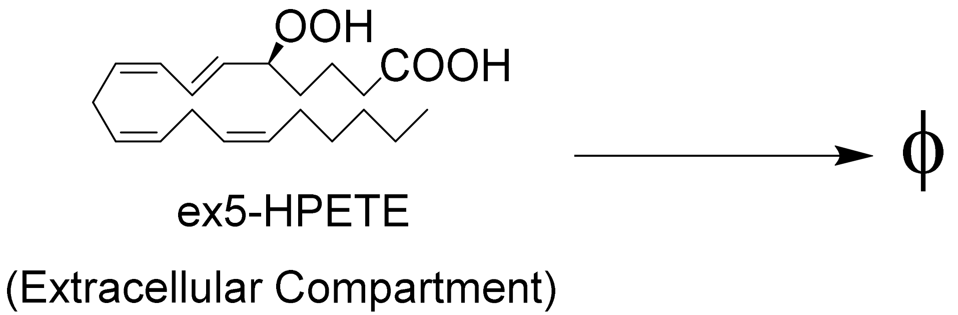


**Figure SF.9.15.** The decay of 5-hydroperoxyeicosatetraenoic acid (5-HPETE) in the extracellular compartment (Reaction 59).

**SEq.9.15.** Reaction rate law for Reaction 59.

$\boldsymbol{v_{59} = K[ex5 - \mathrm{HPETE}]}$

### S.9.15.1. Reaction parameters

#### S.9.15.1.1. Parameter: K

Parameter values for the K of Reaction 59 were obtained from the literature and summarised in Table ST.9.15.1.1.1. The log-normal distribution properties for the K of Reaction 59 are shown in Table ST.9.15.1.1.2 and plotted in Figure SF.9.15.1.1.1.

**Table ST.9.15.1.1.1.** Literature information used to design the ex5-HPETE decay constant parameter distribution. Each value was assigned a weight using the protocol described in Section 2.6.2. The type of error was assigned as 0 if it was additive and 1 if it was multiplicative.

| Half Life (min) | Error (min) | Rate constant ($min^{-1}$) | Error ($min^{-1}$) | Experimental details | | | | | | | Weight | References |
|---|---|---|---|---|---|---|---|---|---|---|---|---|
| | | | | Experiment type | Substrate | Species | Expression Vector | pH | Temperature (°C) | Other | | |
| 0.5 | | 1.386 | | In vivo | **12-HPETE** | Unknown | Unknown | Unknown | Unknown | | 8 | (Maclouf et al., 1982) |

**Table ST.9.15.1.1.2.** The log-normal distribution properties of the ex5-HPETE decay constant distribution. These values were calculated using the functions described in Section 2.6.2.

| Mode ($min^{-1}$) | Confidence Interval | Location parameter (μ) | Scale parameter (σ) |
|---|---|---|---|
| 1.38 | 1.10 | $3.31 \times 10^{-1}$ | $9.4910^{-2}$ |

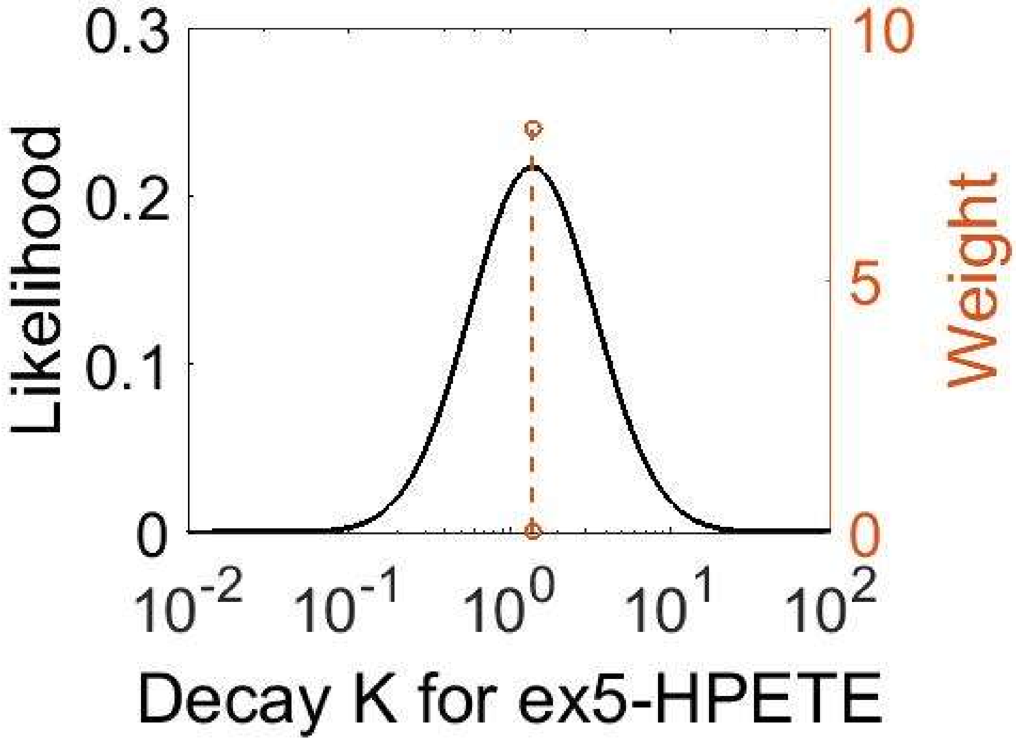


**Figure SF.9.15.1.1.1.** The estimated probability distribution for ex5-HPETE decay constant. The value and weight of the literature values used to define the distribution are indicated by an orange dashed line. The x axis is plotted on a log-scale.

**S.9.16. Reaction 60: ex15-HETE→ϕ**

The decay of 15-HETE in the extracellular compartment into metabolites outside of the scope of this model. An example of metabolites outside of the scope of this model includes metabolites which have been removed from the extracellular compartment into the systemic circulation or are degraded into metabolites not of interest to this model, the term decay reaction refers to the decrease in metabolite concentration by this collection of unspecified processes.

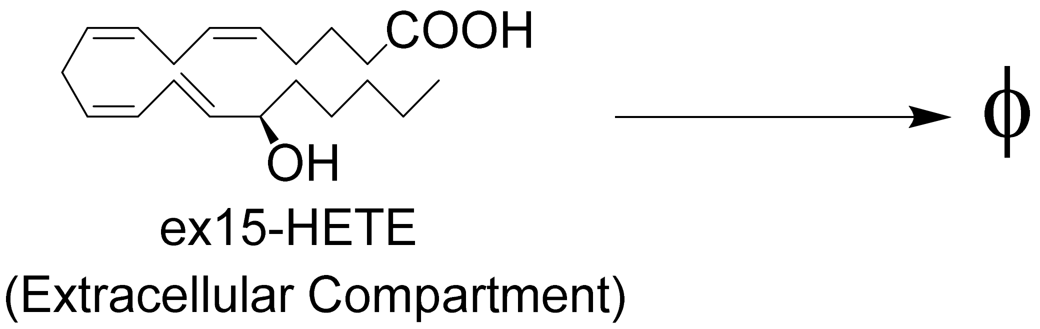


**Figure SF.9.16.** The decay of 15-hydroxyeicosatetraeonic acid (15-HETE) in the extracellular compartment (Reaction 60).

**SEq.9.16.** Reaction rate law for Reaction 60.

$$\boldsymbol{v_{60} = K[ex15 - \mathbf{HETE}\,]}$$

### S.9.16.1. Reaction parameters

### S.9.16.1.1. Parameter: K

Parameter values for the K of Reaction 60 were obtained from the literature and summarised in Table ST.9.16.1.1.1. The log-normal distribution properties for the K of Reaction 60 are shown in Table ST.9.16.1.1.2 and plotted in Figure SF.9.16.1.1.1.

**Table ST.9.16.1.1.1.** Literature information used to design the ex15-HETE decay constant parameter distribution. Each value was assigned a weight using the protocol described in Section 2.6.2. The type of error was assigned as 0 if it was additive and 1 if it was multiplicative.

| Half Life (min) | Error (min) | Rate constant ($min^{-1}$) | Error ($min^{-1}$) | Experimental details | | | | | | | Weight | References |
|---|---|---|---|---|---|---|---|---|---|---|---|---|
| | | | | Experiment type | Substrate | Species | Expression Vector | pH | Temperature (°C) | Other | | |
| 21 | | 0.033 | | In vivo | 15-HETE | Human | R15L Cells | Unknown | 37 | | 8 | (Wei et al., 2009) |

**Table ST.9.16.1.1.2.** The log-normal distribution properties of the ex15-HETE decay constant distribution. These values were calculated using the functions described in Section 2.6.2.

| Mode ($min^{-1}$) | Confidence Interval | Location parameter (μ) | Scale parameter (σ) |
|---|---|---|---|
| $3.30 \times 10^{-2}$ | 1.10 | -3.40 | $9.4910^{-2}$ |

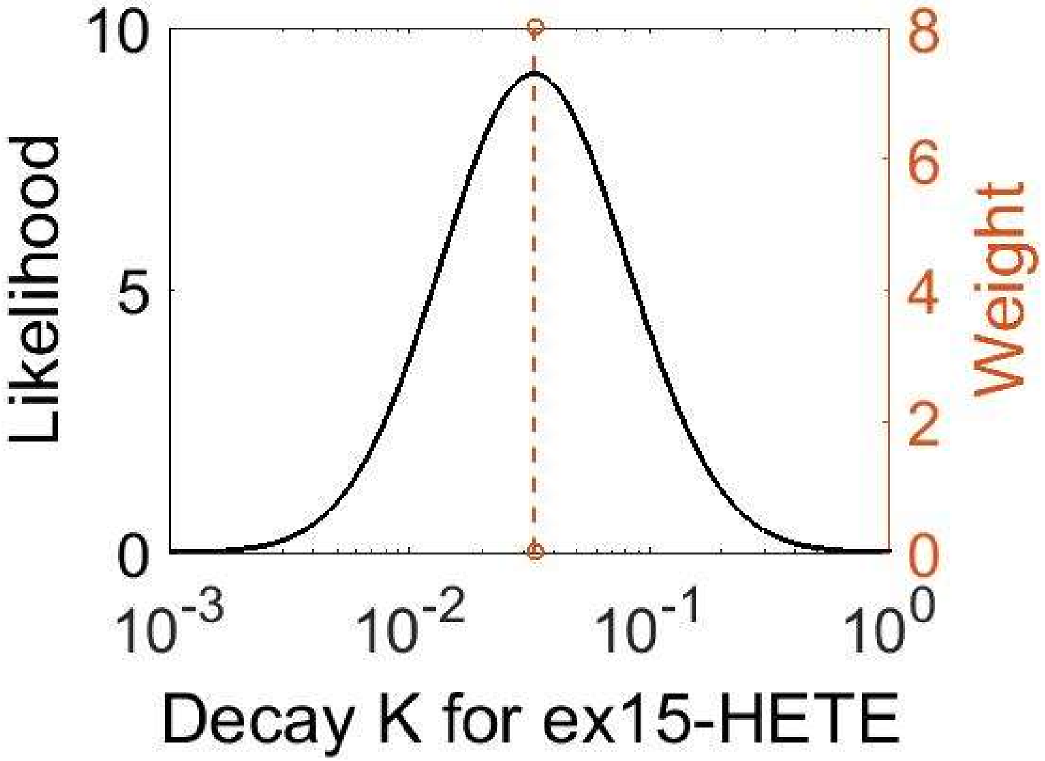


**Figure SF.9.16.1.1.1.** The estimated probability distribution for ex15-HETE decay constant. The value and weight of the literature values used to define the distribution are indicated by an orange dashed line. The x axis is plotted on a log-scale.

**S.9.17. Reaction 61: ex15-HPETE→ϕ**

The decay of 15-HPETE in the extracellular compartment into metabolites outside of the scope of this model. An example of metabolites outside of the scope of this model includes metabolites which have been removed from the extracellular compartment into the systemic circulation or are degraded into metabolites not of interest to this model, the term decay reaction refers to the decrease in metabolite concentration by this collection of unspecified processes.

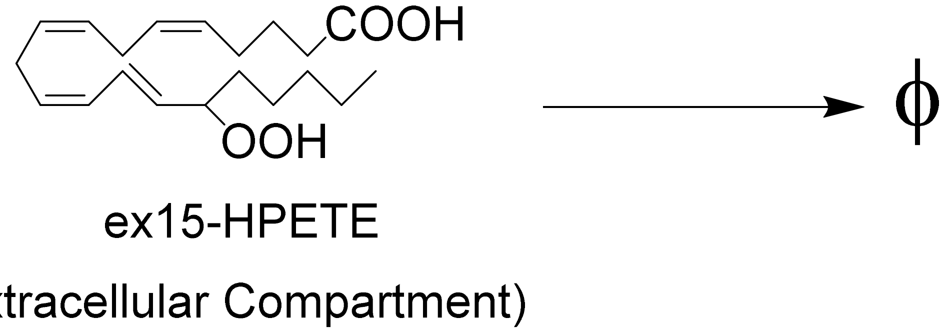


**Figure SF.9.17.** The decay of 15-hydroperoxyeicosatetraenoic acid (15-HPETE) in the extracellular compartment (Reaction 61).

**SEq.9.17.** Reaction rate law for Reaction 61.

$\boldsymbol{v_{61} = K[ex15 - \mathrm{HPETE}]}$

### S.9.17.1. Reaction parameters

#### S.9.17.1.1. Parameter: K

Parameter values for the K of Reaction 61 were obtained from the literature and summarised in Table ST.9.17.1.1.1. The log-normal distribution properties for the K of Reaction 61 are shown in Table ST.9.17.1.1.2 and plotted in Figure SF.9.17.1.1.1.

**Table ST.9.17.1.1.1.** Literature information used to design the ex15-HPETE decay constant parameter distribution. Each value was assigned a weight using the protocol described in Section 2.6.2. The type of error was assigned as 0 if it was additive and 1 if it was multiplicative.

| Half Life (min) | Error (min) | Rate constant ($min^{-1}$) | Error ($min^{-1}$) | Experimental details | | | | | | | Weight | References |
|---|---|---|---|---|---|---|---|---|---|---|---|---|
| | | | | Experiment type | Substrate | Species | Expression Vector | pH | Temperature (°C) | Other | | |
| 0.5 | | 1.386 | | In vivo | **12-HPETE** | Unknown | Unknown | Unknown | Unknown | | 8 | (Maclouf et al., 1982) |

**Table ST.9.17.1.1.2.** The log-normal distribution properties of the ex15-HPETE decay constant distribution. These values were calculated using the functions described in Section 2.6.2.

| Mode ($min^{-1}$) | Confidence Interval | Location parameter (μ) | Scale parameter (σ) |
|---|---|---|---|
| 1.38 | 1.10 | 3.31 x$10^{-1}$ | $9.4910^{-2}$ |

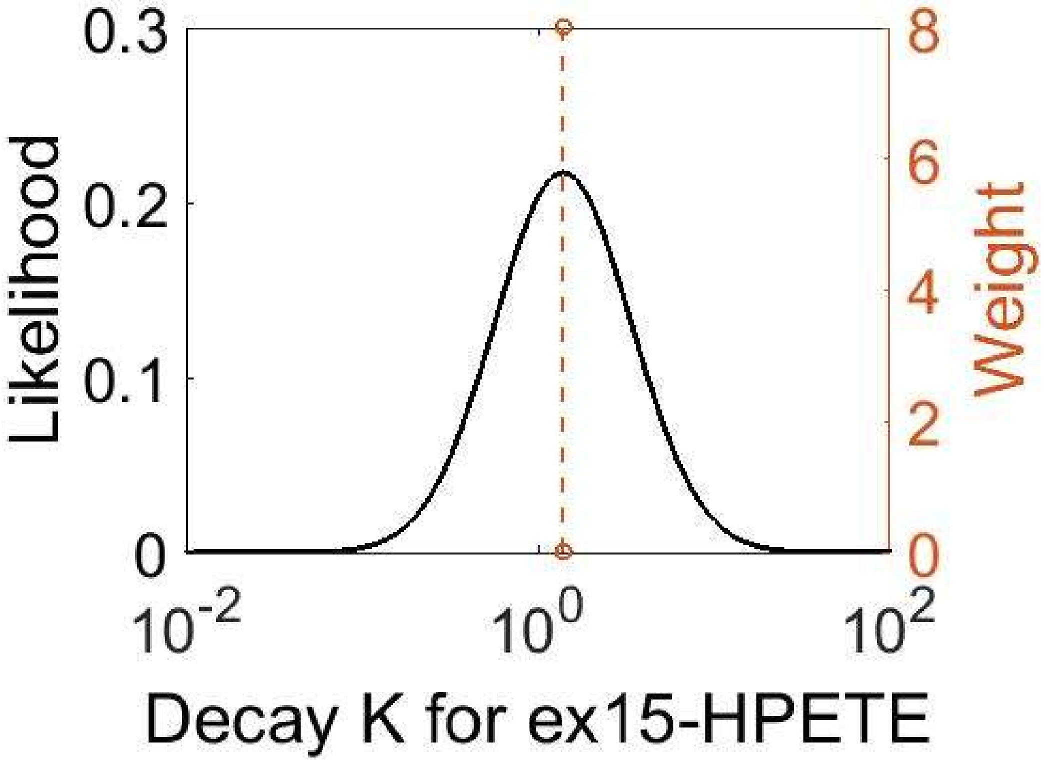


**Figure SF.9.17.1.1.1.** The estimated probability distribution for ex15-HPETE decay constant. The value and weight of the literature values used to define the distribution are indicated by an orange dashed line. The x axis is plotted on a log-scale**.**

**S.9.18. Reaction 62: ex12-HETE →ϕ**

The decay of 12-HETE in the extracellular compartment into metabolites outside of the scope of this model. An example of metabolites outside of the scope of this model includes metabolites which have been removed from the extracellular compartment into the systemic circulation or are degraded into metabolites not of interest to this model, the term decay reaction refers to the decrease in metabolite concentration by this collection of unspecified processes.

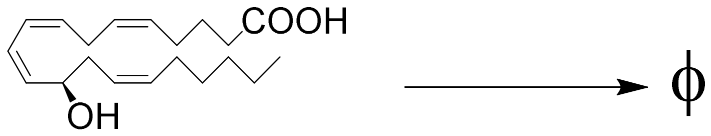


ex12-HETE
(Extracellular Compartment)

**Figure SF.9.18.** The decay of 12-hydroxyeicosatetraenoic acid (12-HETE) in the extracellular compartment (Reaction 62).

**SEq.9.18.** Reaction rate law for Reaction 62.

$$\boldsymbol{v_{62} = K[ex12 - \mathrm{HETE}]}$$

### S.9.18.1. Reaction parameters

#### S.9.18.1.1. Parameter: K

Parameter values for the K of Reaction 62 were obtained from the literature and summarised in Table ST.9.18.1.1.1. The log-normal distribution properties for the K of Reaction 62 are shown in Table ST.9.18.1.1.2 and plotted in Figure SF.9.18.1.1.1.

**Table ST.9.18.1.1.1.** Literature information used to design the ex12-HETE decay constant parameter distribution. Each value was assigned a weight using the protocol described in Section 2.6.2. The type of error was assigned as 0 if it was additive and 1 if it was multiplicative.

| Half Life (min) | Error (min) | Rate constant ($min^{-1}$) | Error ($min^{-1}$) | Experimental details | | | | | | | Weight | References |
|---|---|---|---|---|---|---|---|---|---|---|---|---|
| | | | | Experiment type | Substrate | Species | Expression Vector | pH | Temperature (°C) | Other | | |
| 180 | | 0.004 | | In vivo | 12-HETE | Rabbit | Platelets | Unknown | 37 | | 8 | (Dadaian et al., 1998) |

**Table ST.9.18.1.1.2.** The log-normal distribution properties of the ex12-HETE decay constant distribution. These values were calculated using the functions described in Section 2.6.2.

| Mode ($min^{-1}$) | Confidence Interval | Location parameter (μ) | Scale parameter (σ) |
|---|---|---|---|
| $3.83 \times 10^{-3}$ | 1.10 | -5.56 | $9.4910^{-2}$ |

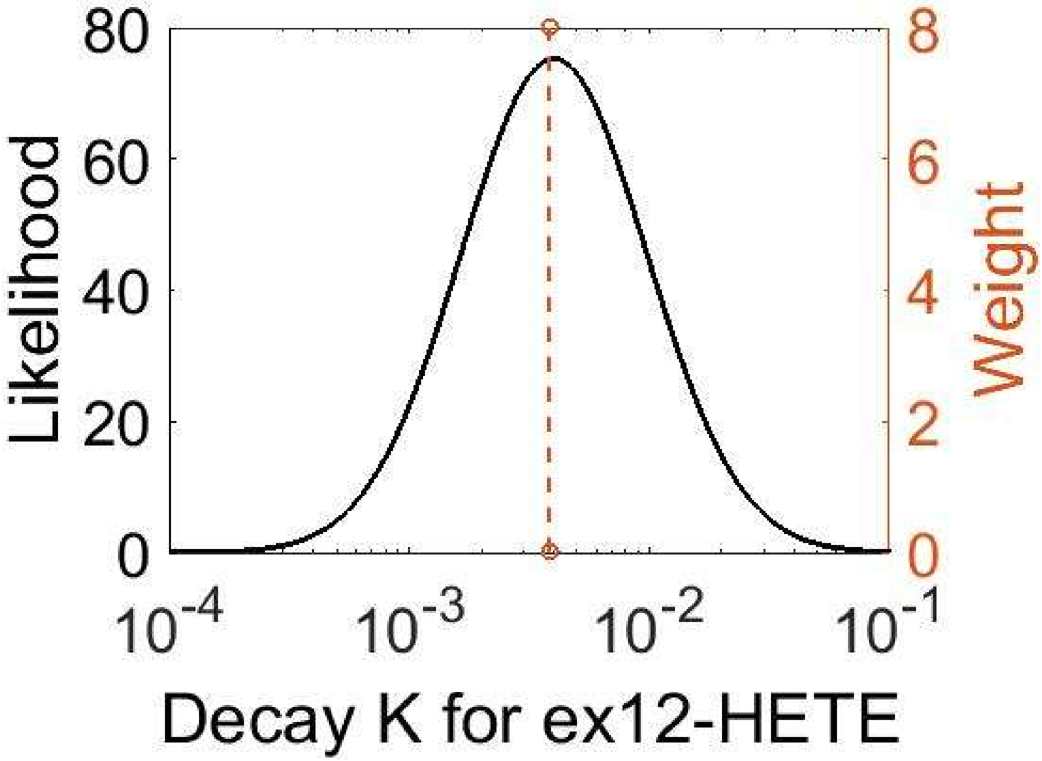


**Figure SF.9.18.1.1.1.** The estimated probability distribution for ex12-HETE decay constant. The value and weight of the literature values used to define the distribution are indicated by an orange dashed line. The x axis is plotted on a log-**scale.**

**S.9.19. Reaction 63: ex12-HPETE→ϕ**

The decay of 12-HPETE in the extracellular compartment into metabolites outside of the scope of this model. An example of metabolites outside of the scope of this model includes metabolites which have been removed from the extracellular compartment into the systemic circulation or are degraded into metabolites not of interest to this model, the term decay reaction refers to the decrease in metabolite concentration by this collection of unspecified processes.

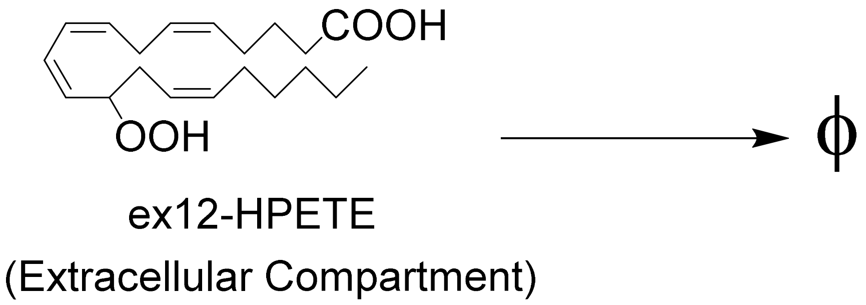


**Figure SF.9.19.** The decay of 12-hydroperoxyeicosatetraenoic acid (12-HPETE) in the extracellular compartment (Reaction 63).

**SEq.9.19.** Reaction rate law for Reaction 63.

$$\boldsymbol{v_{63} = K[ex12 - \mathrm{HPETE}]}$$

## S.9.19.1. Reaction parameters

### S.9.19.1.1. Parameter: K

Parameter values for the K of Reaction 63 were obtained from the literature and summarised in Table ST.9.19.1.1.1. The log-normal distribution properties for the K of Reaction 63 are shown in Table ST.9.19.1.1.2 and plotted in Figure SF.9.19.1.1.1.

**Table ST.9.19.1.1.1.** Literature information used to design the ex12-HPETE decay constant parameter distribution. Each value was assigned a weight using the protocol described in Section 2.6.2. The type of error was assigned as 0 if it was additive and 1 if it was multiplicative.

| Half Life (min) | Error (min) | Rate constant (min$^{-1}$) | Error (min$^{-1}$) | Experimental details | | | | | | | Weight | References |
|---|---|---|---|---|---|---|---|---|---|---|---|---|
| | | | | Experiment type | Substrate | Species | Expression Vector | pH | Temperature (°C) | Other | | |
| 0.5 | | 1.386 | | Unknown | 12-HPETE | Unknown | Unknown | Unknown | Unknown | Textbook with no reference | 8 | (Maclouf et al., 1982) |

**Table ST.9.19.1.1.2.** The log-normal distribution properties of the ex12-HPETE decay constant distribution. These values were calculated using the functions described in Section 2.6.2.

| Mode (min$^{-1}$) | Confidence Interval | Location parameter (μ) | Scale parameter (σ) |
|---|---|---|---|
| 1.85 x10$^{-1}$ | 7.46 | -2.52 x10$^{-1}$ | 1.20 |

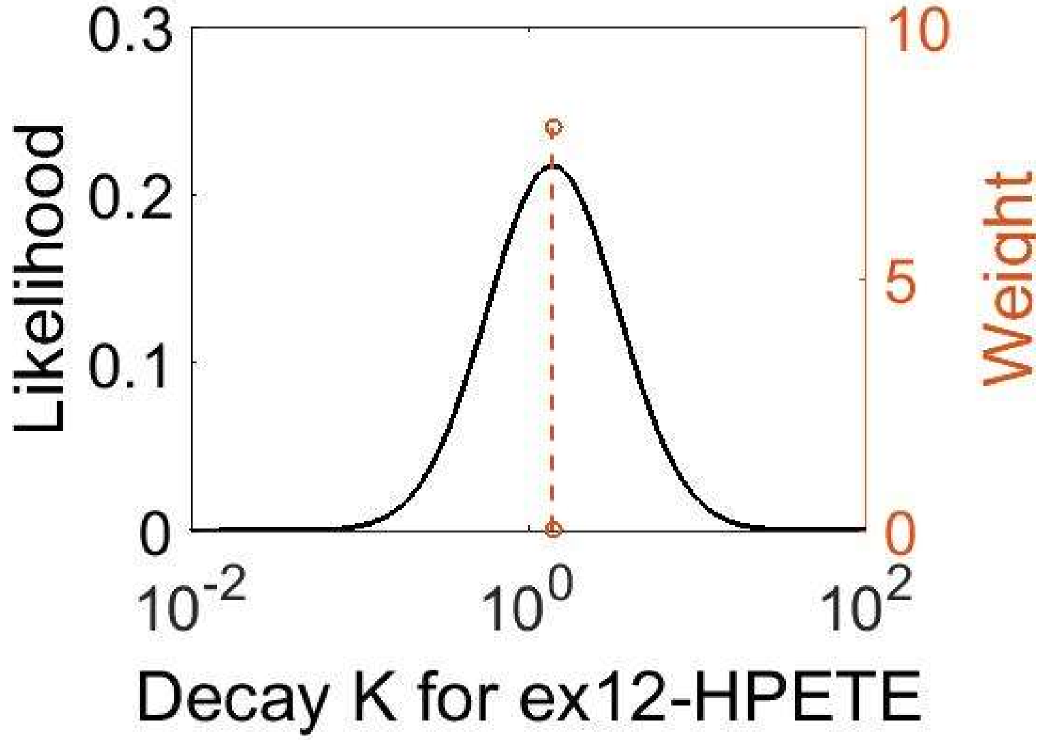


**Figure SF.9.19.1.1.1.** The estimated probability distribution for ex12-HPETE decay constant. The value and weight of the literature values used to define the distribution are indicated by an orange dashed line. The x axis is plotted on a log-scale.

**S.9.20. Reaction 64: exAA →ϕ**

The decay of AA in the extracellular compartment into metabolites outside of the scope of this model. An example of metabolites outside of the scope of this model includes metabolites which have been removed from the extracellular compartment into the systemic circulation or are degraded into metabolites not of interest to this model, the term decay reaction refers to the decrease in metabolite concentration by this collection of unspecified processes.

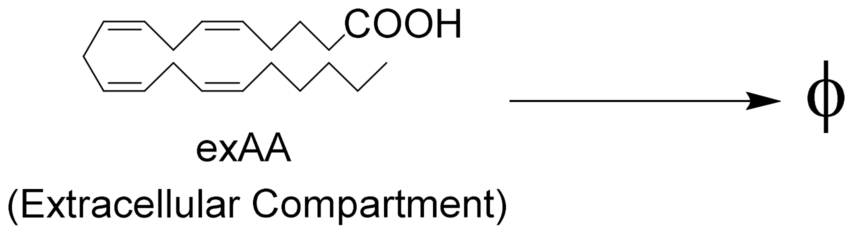


**Figure SF.9.20.** The decay of arachidonic acid (AA) in the extracellular compartment (Reaction 64).

**SEq.9.20.** Reaction rate law for Reaction 64.

$$\boldsymbol{v_{64} = K[exAA]}$$

### S.9.20.1. Reaction parameters

### S.9.20.1. Parameter: K

Parameter values for the K of Reaction 64 were obtained from the literature and summarised in Table ST.9.20.1.1.1. The log-normal distribution properties for the K of Reaction 64 are shown in Table ST.9.20.1.1.2 and plotted in Figure SF.9.20.1.1.1.

**Table ST.9.20.1.1.1.** Literature information used to design the exAA decay constant parameter distribution. Each value was assigned a weight using the protocol described in Section 2.6.2. The type of error was assigned as 0 if it was additive and 1 if it was multiplicative.

| Half Life (min) | Error (min) | Rate constant ($min^{-1}$) | Error ($min^{-1}$) | Experimental details | | | | | | | Weight | References |
|---|---|---|---|---|---|---|---|---|---|---|---|---|
| | | | | Experiment type | Substrate | Species | Expression Vector | pH | Temperature (°C) | Other | | |
| 240 | | 0.003 | | In vivo | AA | Human | Platelets | Unknown | 37 | | 8 | (Vinge, 1985) |

**Table ST.9.20.1.1.2.** The log-normal distribution properties of the exAA decay constant distribution. These values were calculated using the functions described in Section 2.6.2.

| Mode ($min^{-1}$) | Confidence Interval | Location parameter (μ) | Scale parameter (σ) |
|---|---|---|---|
| 2.88 $x10^{-3}$ | 1.10 | -5.84 | $9.4910^{-2}$ |

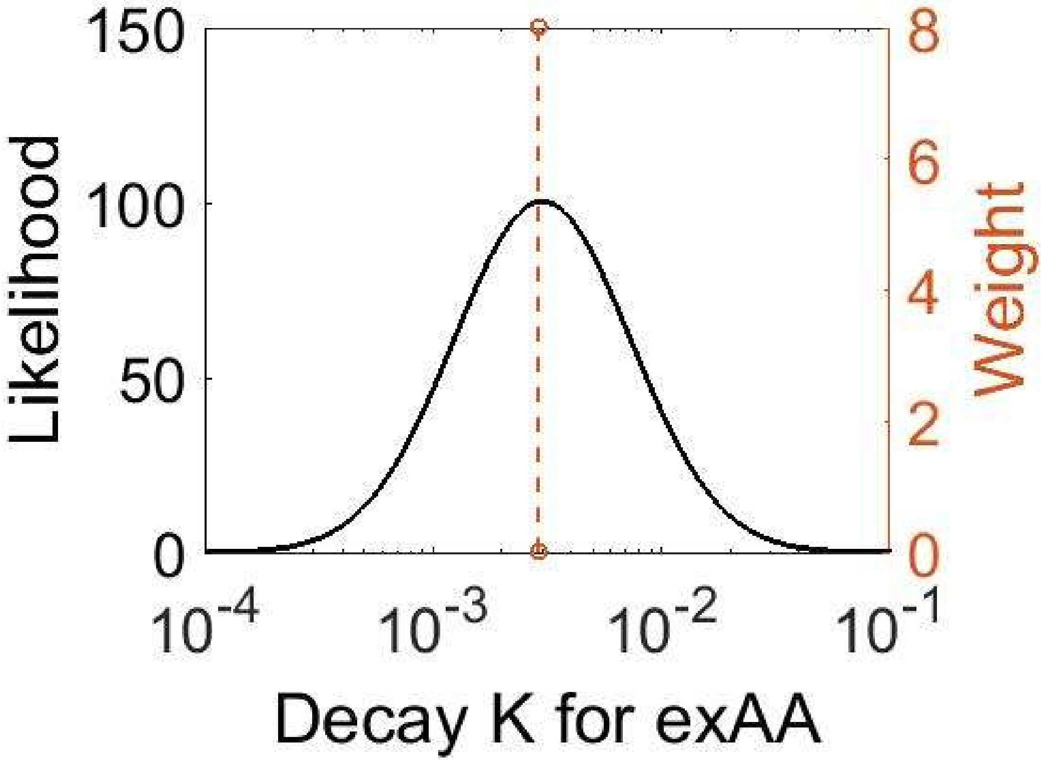


**Figure SF.9.20.1.1.1.** The estimated probability distribution for exAA decay constant. The value and weight of the literature values used to define the distribution are indicated by an orange dashed line. The x axis is plotted on a log-scale.

**S.9.21. Reaction 68: ex15-keto-PGE2 →ϕ**

The decay of 15-keto-$PGE_2$ in the extracellular compartment into metabolites outside of the scope of this model. An example of metabolites outside of the scope of this model includes metabolites which have been removed from the extracellular compartment into the systemic circulation or are degraded into metabolites not of interest to this model, the term decay reaction refers to the decrease in metabolite concentration by this collection of unspecified processes.

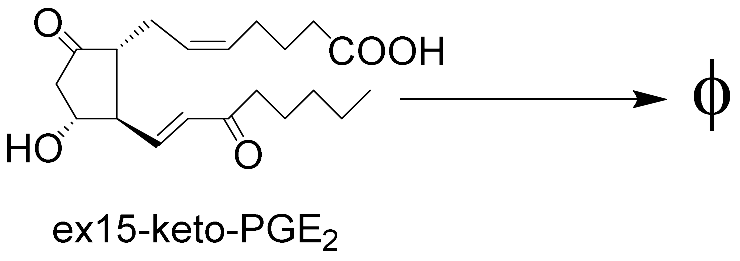


**Figure SF.9.21.** The decay of 15-keto-prostaglandin $E_2$ (15-keto-$PGE_2$) in the extracellular compartment (Reaction 68).

S.7.1.1. Reaction rate law for Reaction 68.

$$\boldsymbol{v_{68}} = \boldsymbol{K}[\mathbf{ex15-keto-PGE_2}]$$

### S.9.21.1. Reaction parameters

#### S.9.21.1.1. Parameter: K

Parameter values for the K of Reaction 68 were obtained from the literature and summarised in Table ST.9.21.1.1.1. The log-normal distribution properties for the K of Reaction 68 are shown in Table ST.9.21.1.1.2 and plotted in Figure SF.9.21.1.1.1.

**Table ST.9.21.1.1.1.** Literature information used to design the ex15-keto-$PGE_2$ decay constant parameter distribution. Each value was assigned a weight using the protocol described in Section 2.6.2. The type of error was assigned as 0 if it was additive and 1 if it was multiplicative.

| Half Life (min) | Error (min) | Rate constant ($min^{-1}$) | Error ($min^{-1}$) | Experimental details | | | | | | | Weight | Type of error | Reference |
|---|---|---|---|---|---|---|---|---|---|---|---|---|---|
| | | | | Experiment type | Substrate | Species | Expression Vector | pH | Temperature (°C) | Other | | | |
| 9.9 | | 0.07 | | In vivo | **13,14-Dihydro-15-Keto-PGE2** | Unknown | Unknown | Unknown | Unknown | | 8 | | (Bothwell et al., 1982) |

**Table ST.9.21.1.1.2.** The log-normal distribution properties of the ex15-keto-$PGE_2$ decay constant distribution. These values were calculated using the functions described in Section 2.6.2.

| Mode ($min^{-1}$) | Confidence Interval | Location parameter (μ) | Scale parameter (σ) |
|---|---|---|---|
| $6.97 \times 10^{-3}$ | 1.10 | -4.96 | $9.49 \times 10^{-2}$ |

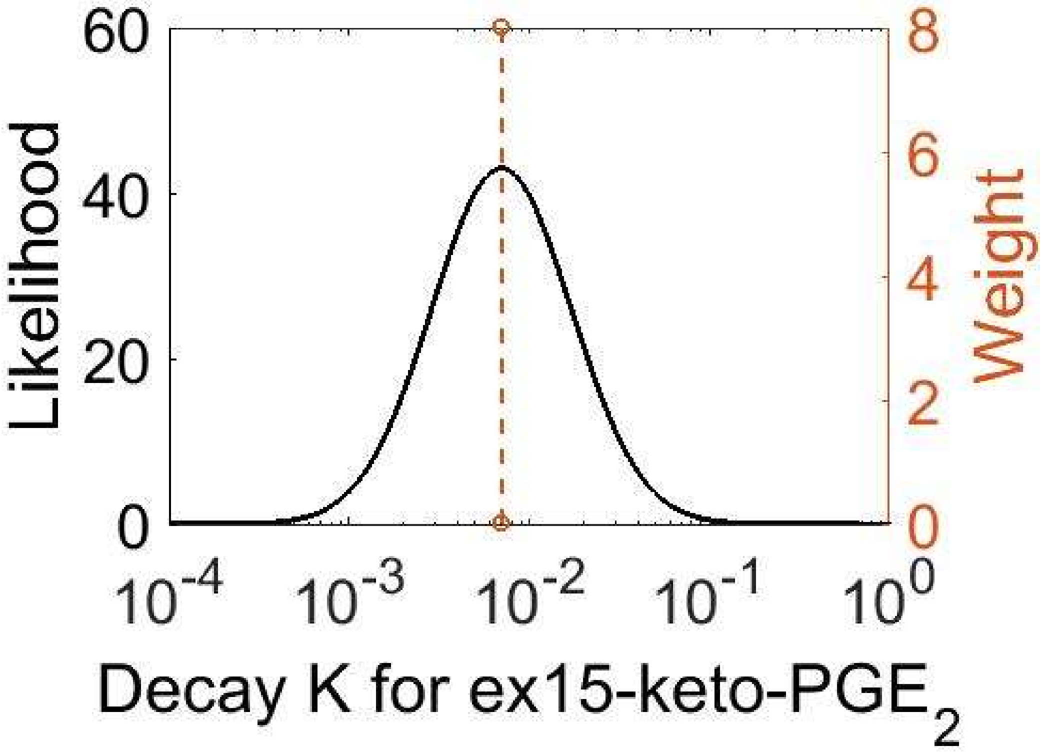


**Figure SF.9.21.1.1.1.** The estimated probability distribution for the ex15-keto-PGE$_2$ decay constant. The value and weight of the literature values used to define the distribution are indicated by an orange dashed line. The x axis is plotted on a log-scale**.**

**S.9.22. Reaction 71: ex13,14-dihydro-15-keto-PGE$_2$ →ϕ**

The decay of 13,14-dihydro-15-keto-PGE$_2$ in the extracellular compartment into metabolites outside of the scope of this model. An example of metabolites outside of the scope of this model includes metabolites which have been removed from the extracellular compartment into the systemic circulation or are degraded into metabolites not of interest to this model, the term decay reaction refers to the decrease in metabolite concentration by this collection of unspecified processes.

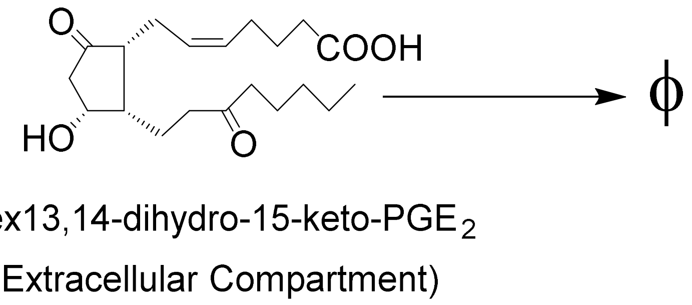


**Figure SF.9.22.** The decay of 13,14-dihydro-15-keto-prostaglandin E$_2$ (13,14-dihydro-15-keto-PGE$_2$) in the extracellular compartment (Reaction 71).

**SEq.9.22.** Reaction rate law for Reaction 71.

$$\boldsymbol{v_{64} = K[\mathbf{ex13,14-dihydro-15-keto-PGE_2}\,]}$$

### S.9.22.1. Reaction parameters

#### S.9.22.1.1. Parameter: K

Parameter values for the K of Reaction 71 were obtained from the literature and summarised in Table ST.9.22.1.1.1. The log-normal distribution properties for the K of Reaction 71 are shown in Table ST.9.22.1.1.2 and plotted in Figure SF.9.22.1.1.1.

**Table ST.9.22.1.1.1.** Literature information used to design the ex13,14-dihydro-15-keto-$PGE_2$ parameter distribution. Each value was assigned a weight using the protocol described in Section 2.6.2. The type of error was assigned as 0 if it was additive and 1 if it was multiplicative.

| Half Life (min) | Error (min) | Rate constant ($min^{-1}$) | Error ($min^{-1}$) | Experimental details | | | | | | | Weight | Type of error | Reference |
|---|---|---|---|---|---|---|---|---|---|---|---|---|---|
| | | | | Experiment type | Substrate | Species | Expression Vector | pH | Temperature (°C) | Other | | | |
| 9.9 | | 0.07 | | In vivo | **13,14-Dihydro-15-Keto-PGE2** | Unknown | Unknown | Unknown | Unknown | | 8 | | (Bothwell et al., 1982) |

**Table ST.9.22.1.1.2.** The log-normal distribution properties of the 13,14-dihydro-15-keto-$PGE_2$ decay constant distribution. These values were calculated using the functions described in Section 2.6.2.

| Mode ($min^{-1}$) | Confidence Interval | Location parameter (μ) | Scale parameter (σ) |
|---|---|---|---|
| 6.97 $x10^{-3}$ | 1.10 | -4.96 | $9.4910^{-2}$ |

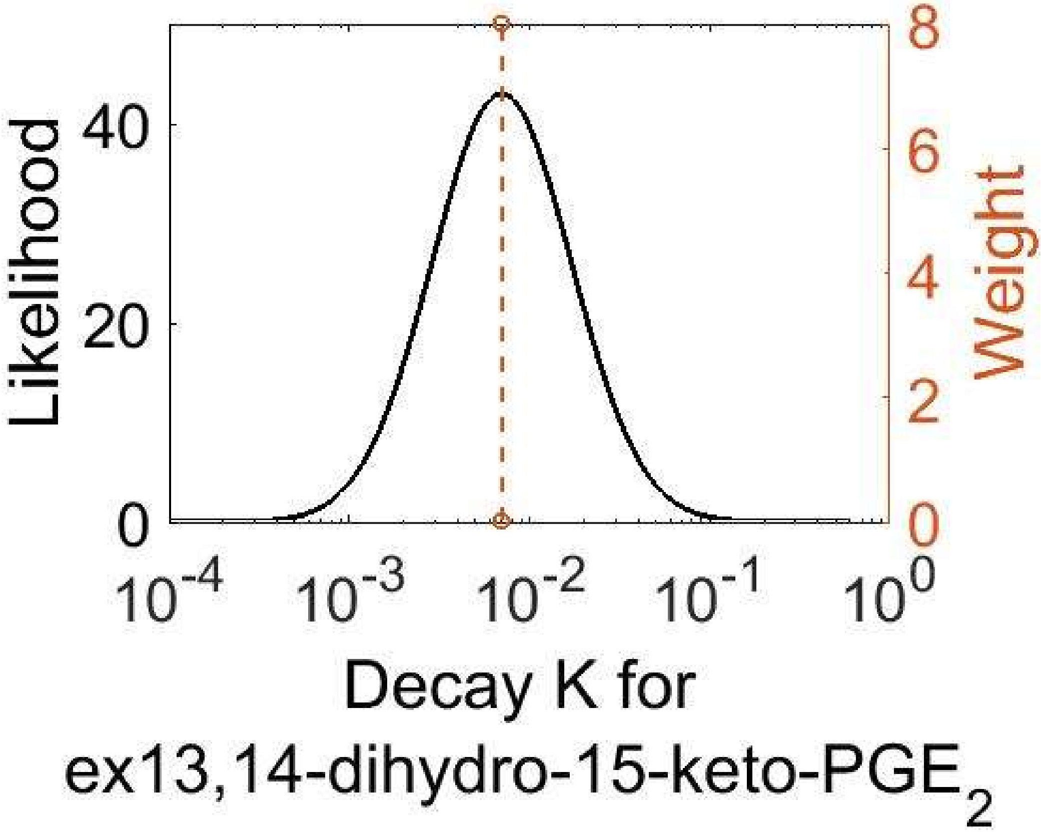


**Figure SF.9.22.1.1.1.** The estimated probability distribution for the ex13,14-dihydro-15-keto-PGE$_2$ decay constant. The value and weight of the literature values used to define the distribution are indicated by an orange dashed line. The x axis is plotted on a log-scale.

**S.9.23. Reaction 72: AA →ϕ**

The decay of AA in the intracellular compartment into metabolites outside of the scope of this model. An example of metabolites outside of the scope of this model includes metabolites which have been removed from the extracellular compartment into the systemic circulation or are degraded into metabolites not of interest to this model, the term decay reaction refers to the decrease in metabolite concentration by this collection of unspecified processes.

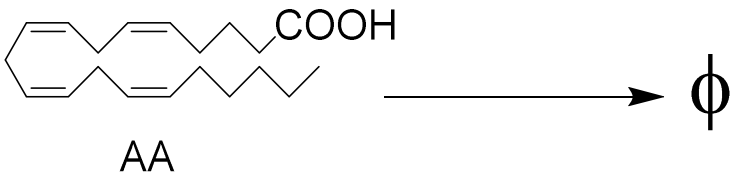


**Figure SF.9.23.** The decay of arachidonic acid (AA) in the intracellular compartment (Reaction 72).

**SEq.9.23.** Reaction rate law for Reaction 72.

$$\boldsymbol{v_{72} = K[\mathrm{AA}]}$$

### S.9.23.1. Reaction parameters

#### S.9.23.1.1. Parameter: K

Parameter values for the K of Reaction 72 were obtained from the literature and summarised in Table ST.9.23.1.1.1. The log-normal distribution properties for the K of Reaction 72 are shown in Table ST.9.23.1.1.2 and plotted in Figure SF.9.23.1.1.1.

**Table ST.9.23.1.1.1.** Literature information used to design the AA decay constant parameter distribution. Each value was assigned a weight using the protocol described in Section 2.6.2. The type of error was assigned as 0 if it was additive and 1 if it was multiplicative.

| Half Life (min) | Error (min) | Rate constant ($min^{-1}$) | Error ($min^{-1}$) | Experimental details | | | | | | | Weight | Type of error | Reference |
|---|---|---|---|---|---|---|---|---|---|---|---|---|---|
| | | | | Experiment type | Substrate | Species | Expression Vector | pH | Temperature (°C) | Other | | | |
| 240 | | 0.003 | | In vivo | AA | Human | Platelets | Unknown | 37 | | 8 | | (Vinge, 1985) |

**Table ST.9.23.1.1.2.** The log-normal distribution properties of the AA decay constant distribution. These values were calculated using the functions described in Section 2.6.2.

| Mode ($min^{-1}$) | Confidence Interval | Location parameter (μ) | Scale parameter (σ) |
|---|---|---|---|
| 2.88 $\times 10^{-3}$ | 1.10 | -5.84 | 9.49 $\times 10^{-2}$ |

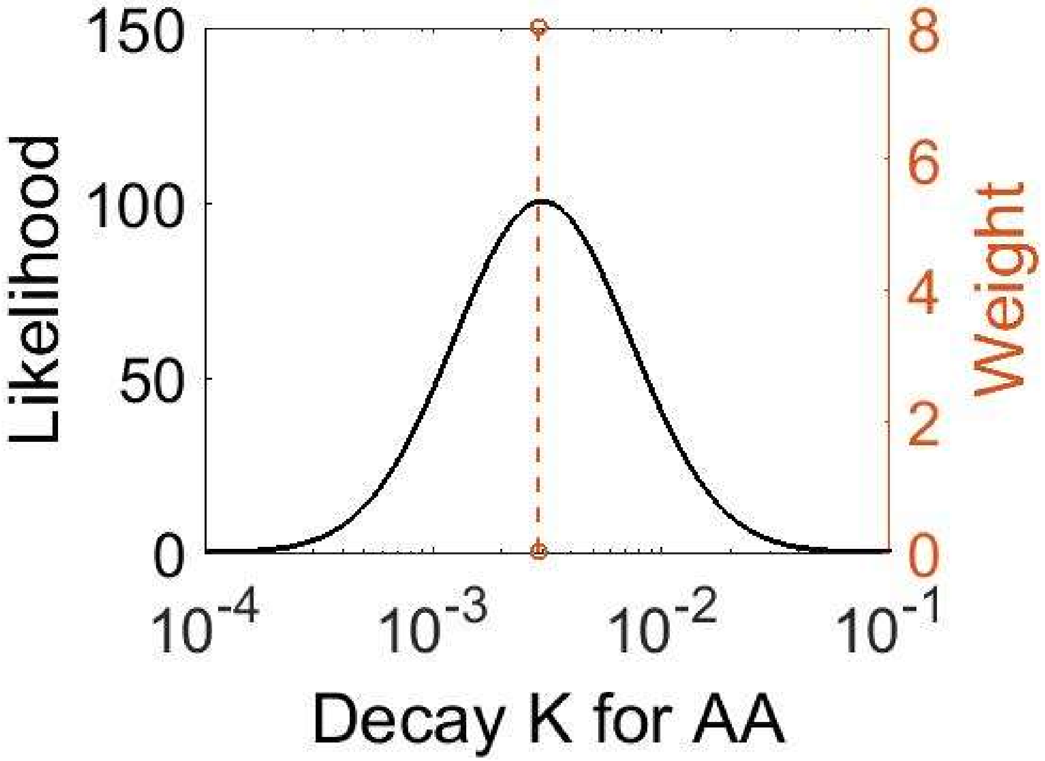


**Figure SF.9.23.1.1.1.** The estimated probability distribution for the AA decay constant. The value and weight of the literature values used to define the distribution are indicated by an orange dashed line. The x axis is plotted on a log-scale.

**S.9.24. Reaction 73: $PGH_2 \rightarrow \phi$**

The decay of $PGH_2$ in the intracellular compartment into metabolites outside of the scope of this model. An example of metabolites outside of the scope of this model includes metabolites which have been removed from the extracellular compartment into the systemic circulation or are degraded into metabolites not of interest to this model, the term decay reaction refers to the decrease in metabolite concentration by this collection of unspecified processes.

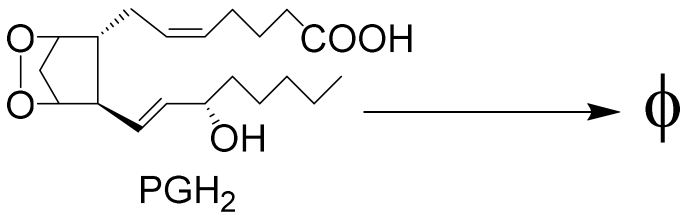


**Figure SF.9.24.** The decay of prostaglandin $H_2$ ($PGH_2$) in the intracellular compartment (Reaction 73).

**SEq.9.24.** Reaction rate law for Reaction 73.

$$\boldsymbol{v_{73} = K[\mathrm{PGH_2}]}$$

## S.9.24.1. Reaction parameters

### S.9.24.1.1. Parameter: K

Parameter values for the K of Reaction 73 were obtained from the literature and summarised in Table ST.9.24.1.1.1. The log-normal distribution properties for the K of Reaction 73 are shown in Table ST.9.24.1.1.2 and plotted in Figure SF.9.24.1.1.1.

**Table ST.9.24.1.1.1.** Literature information used to design the $PGH_2$ decay constant parameter distribution. Each value was assigned a weight using the protocol described in Section 2.6.2. The type of error was assigned as 0 if it was additive and 1 if it was multiplicative.

| Half Life (min) | Error (min) | Rate constant ($min^{-1}$) | Error ($min^{-1}$) | Experimental details | | | | | | | Weight | Type of error | Reference |
|---|---|---|---|---|---|---|---|---|---|---|---|---|---|
| | | | | Experiment type | Substrate | Species | Expression Vector | pH | Temperature (°C) | Other | | | |
| 5 | | 0.139 | | In vivo | PGH2 | Unknown | Unknown | Unknown | Unknown | Textbook with no reference | 8 | | (Sciences, 2019) |

**Table ST.9.24.1.1.2.** The log-normal distribution properties of the $PGH_2$ decay constant distribution. These values were calculated using the functions described in Section 2.6.2.

| Mode ($min^{-1}$) | Confidence Interval | Location parameter (μ) | Scale parameter (σ) |
|---|---|---|---|
| $1.38 \times 10^{-1}$ | 1.10 | -1.97 | $9.49 \times 10^{-2}$ |

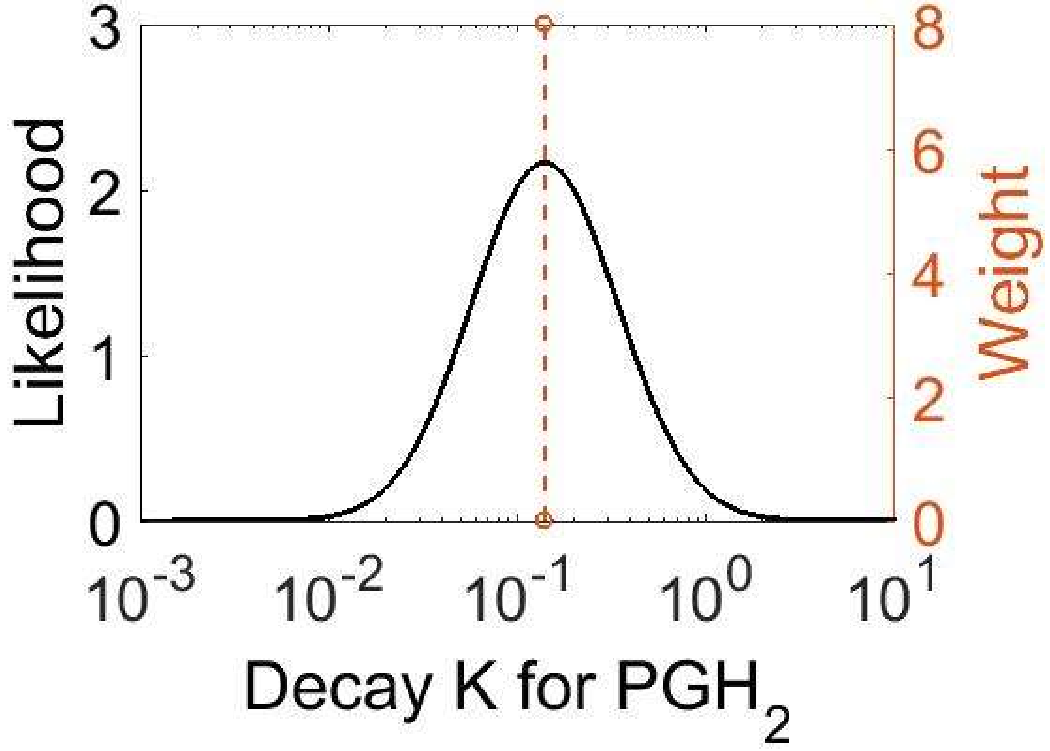


**Figure SF.9.24.1.1.1.** The estimated probability distribution for $PGH_2$ decay constant. The value and weight of the literature values used to define the distribution are indicated by an orange dashed line. The x axis is plotted on a log-scale.

**S.9.25. Reaction 74: $PGF_{2\alpha} \rightarrow \phi$**

The decay of $PGF_{2\alpha}$ in the intracellular compartment into metabolites outside of the scope of this model. An example of metabolites outside of the scope of this model includes metabolites which have been removed from the extracellular compartment into the systemic circulation or are degraded into metabolites not of interest to this model, the term decay reaction refers to the decrease in metabolite concentration by this collection of unspecified processes.

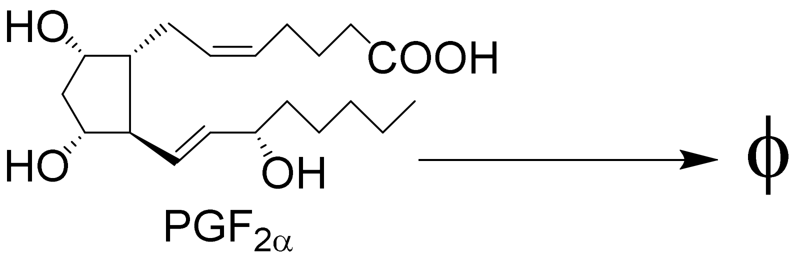


**Figure S.9.25.** The decay of prostaglandin $F_{2\alpha}$ ($PGF_{2\alpha}$) in the intracellular compartment (Reaction 74).

**SEq.9.25.** Reaction rate law for Reaction 74.

$$\boldsymbol{v_{74} = K[\mathrm{PGF_{2\alpha}}]}$$

## S.9.25.1. Reaction parameters

### S.9.25.1.1. Parameter: K

Parameter values for the K of Reaction 74 were obtained from the literature and summarised in Table ST.9.25.1.1.1. The log-normal distribution properties for the K of Reaction 74 are shown in Table ST.9.25.1.1.2 and plotted in Figure SF.9.25.1.1.1.

**Table ST.9.25.1.1.1.** Literature information used to design the $PGF_{2\alpha}$ decay constant parameter distribution. Each value was assigned a weight using the protocol described in Section 2.6.2. The type of error was assigned as 0 if it was additive and 1 if it was multiplicative.

| Half Life (min) | Error (min) | Rate constant (min$^{-1}$) | Error (min$^{-1}$) | Experimental details | | | | | | | Weight | Type of error | Reference |
|---|---|---|---|---|---|---|---|---|---|---|---|---|---|
| | | | | Experiment type | Substrate | Species | Expression Vector | pH | Temperature (°C) | Other | | | |
| 900 | 492 | 0.001 | 0.001 | In vivo | PGF2a | Human | Decidual stromal cells and macrophages | Unknown | Unknown | | 8 | | (Ishihara et al., 1991) |

**Table ST.9.25.1.1.2.** The log-normal distribution properties of the $PGH_2$ decay constant distribution. These values were calculated using the functions described in Section 2.6.2.

| Mode (min$^{-1}$) | Confidence Interval | Location parameter (μ) | Scale parameter (σ) |
|---|---|---|---|
| 7.07 x10$^{-4}$ | 2.30 | -6.80 | 6.7610$^{-1}$ |

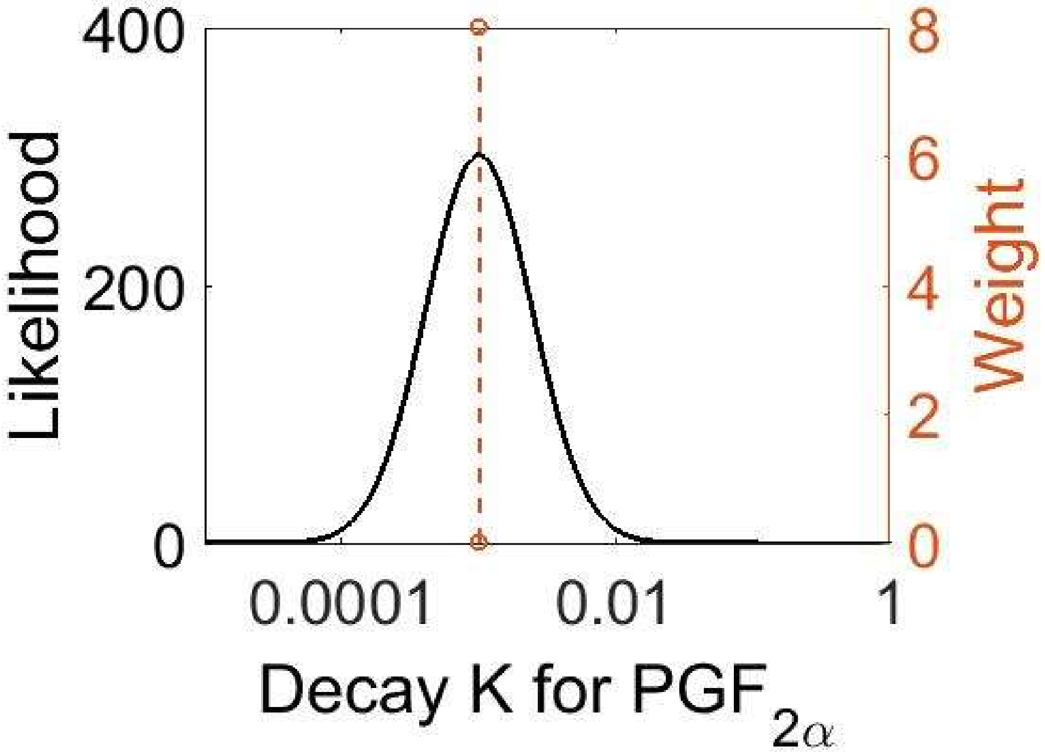


**Figure SF.9.25.1.1.1.** The estimated probability distribution for $PGF_{2\alpha}$ decay constant. The value and weight of the literature values used to define the distribution are indicated by an orange dashed line. The x axis is plotted on a log-scale.

**S.9.26. Reaction 75: $TXA_2 \rightarrow \phi$**

The decay of $TXA_2$ in the intracellular compartment into metabolites outside of the scope of this model. An example of metabolites outside of the scope of this model includes metabolites which have been removed from the extracellular compartment into the systemic circulation or are degraded into metabolites not of interest to this model, the term decay reaction refers to the decrease in metabolite concentration by this collection of unspecified processes.

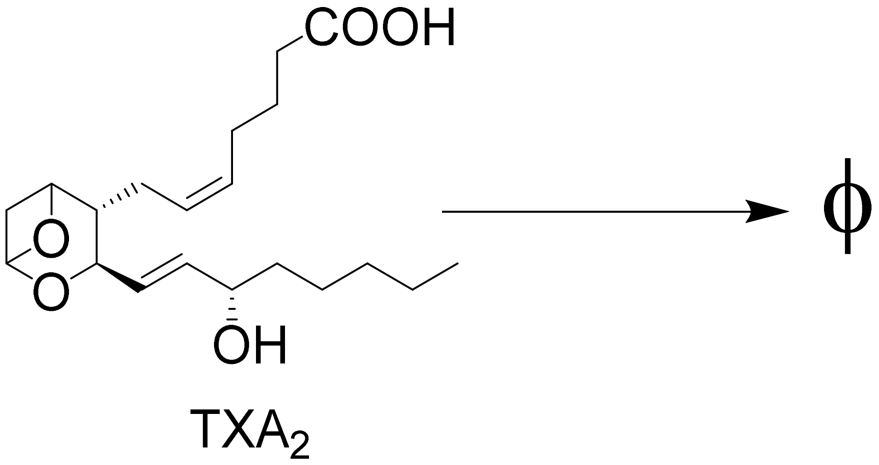


**Figure SF.9.26.** The decay of thromboxane $A_2$ ($TXA_2$) in the intracellular compartment (Reaction 75).

**SEq.9.26.** Reaction rate law for Reaction 75.

$$\boldsymbol{v_{75} = K[\mathrm{TXA_2}]}$$

### S.9.26.1. Reaction parameters

#### S.9.26.1.1. Parameter: K

Parameter values for the K of Reaction 75 were obtained from the literature and summarised in Table ST.9.26.1.1.1. The log-normal distribution properties for the K of Reaction 75 are shown in Table ST.9.26.1.1.2 and plotted in Figure SF.9.26.1.1.1.

**Table ST.9.26.1.1.1.** Literature information used to design the $TXA_2$ decay constant parameter distribution. Each value was assigned a weight using the protocol described in Section 2.6.2. The type of error was assigned as 0 if it was additive and 1 if it was multiplicative.

| Half Life (min) | Error (min) | Rate constant ($min^{-1}$) | Error ($min^{-1}$) | Experimental details | | | | | | | Weight | Type of error | Reference |
|---|---|---|---|---|---|---|---|---|---|---|---|---|---|
| | | | | Experiment type | Substrate | Species | Expression Vector | pH | Temperature (°C) | Other | | | |
| 0.333 | | 2.079 | | Unknown, assume in vitro | TXA2 | Unknown | Unknown | Unknown | Unknown | Textbook with no reference | 8 | | (Wild, 2005) |

**Table ST.9.26.1.1.2.** The log-normal distribution properties of the $TXA_2$ decay constant distribution. These values were calculated using the functions described in Section 2.6.2.

| Mode ($min^{-1}$) | Confidence Interval | Location parameter (μ) | Scale parameter (σ) |
|---|---|---|---|
| 2.07 | 1.10 | $7.37 \times 10^{-1}$ | $9.49 \times 10^{-2}$ |

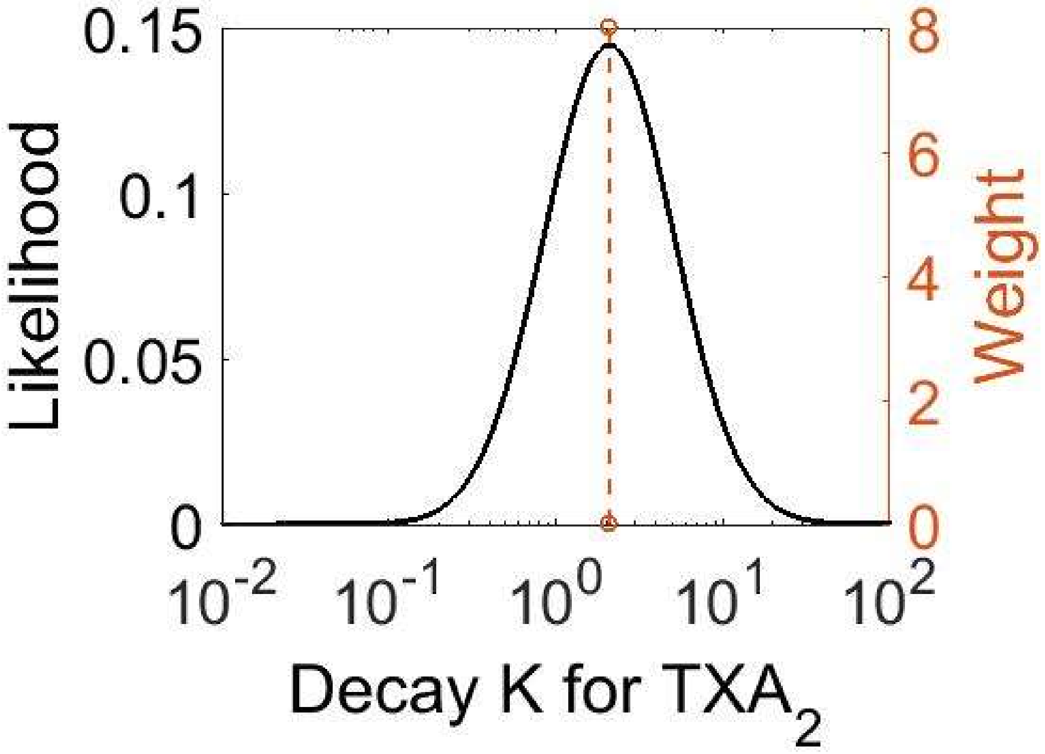


**Figure SF.9.26.1.1.1.** The estimated probability distribution for the $TXA_2$ decay constant. The value and weight of the literature values used to define the distribution are indicated by an orange dashed line. The x axis is plotted on a log-scale.

**S.9.27. Reaction 76: $PGE_2 \rightarrow \phi$**

The decay of $PGE_2$ in the intracellular compartment into metabolites outside of the scope of this model. An example of metabolites outside of the scope of this model includes metabolites which have been removed from the extracellular compartment into the systemic circulation or are degraded into metabolites not of interest to this model, the term decay reaction refers to the decrease in metabolite concentration by this collection of unspecified processes.

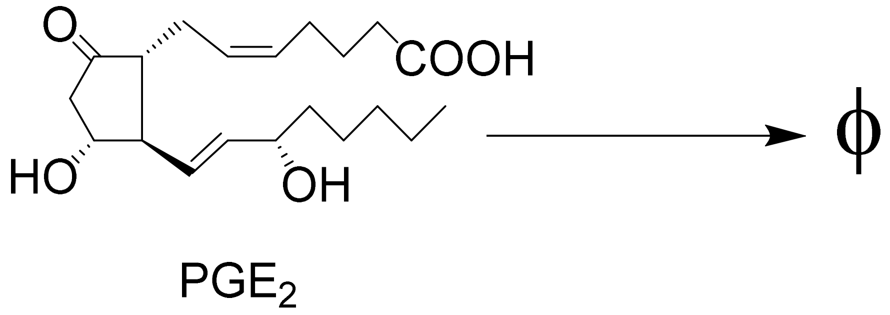


**Figure SF.9.27.** The decay of prostaglandin $E_2$ ($PGE_2$) in the intracellular compartment (Reaction 76).

**SEq.9.27.** Reaction rate law for Reaction 76.

$$\boldsymbol{v_{76} = K[\mathrm{PGE_2}]}$$

## S.9.27.1. Reaction parameters

### S.9.27.1.1 Parameter: K

Parameter values for the K of Reaction 76 were obtained from the literature and summarised in Table ST.9.27.1.1.1. The log-normal distribution properties for the K of Reaction 76 are shown in Table ST.9.27.1.1.2 and plotted in Figure SF.9.27.1.1.1.

**Table ST.9.27.1.1.1.** Literature information used to design the $PGE_2$ decay constant parameter distribution. Each value was assigned a weight using the protocol described in Section 2.6.2. The type of error was assigned as 0 if it was additive and 1 if it was multiplicative.

| Half Life (min) | Error (min) | Rate constant ($min^{-1}$) | Error ($min^{-1}$) | Experimental details | | | | | | | Weight | Type of error | Reference |
|---|---|---|---|---|---|---|---|---|---|---|---|---|---|
| | | | | Experiment type | Substrate | Species | Expression Vector | pH | Temperature (°C) | Other | | | |
| 528 | 204 | 0.001 | 0.003 | In vivo | PGE2 | Human | Decidual stromal cells and macrophages | Unknown | Unknown | | 8 | | (Ishihara et al., 1991) |

**Table ST.9.27.1.1.2.** The log-normal distribution properties of the $PGE_2$ decay constant distribution. These values were calculated using the functions described in Section 2.6.2.

| Mode ($min^{-1}$) | Confidence Interval | Location parameter (μ) | Scale parameter (σ) |
|---|---|---|---|
| $3.16 \times 10^{-4}$ | 4.56 | -7.04 | 1.01 |

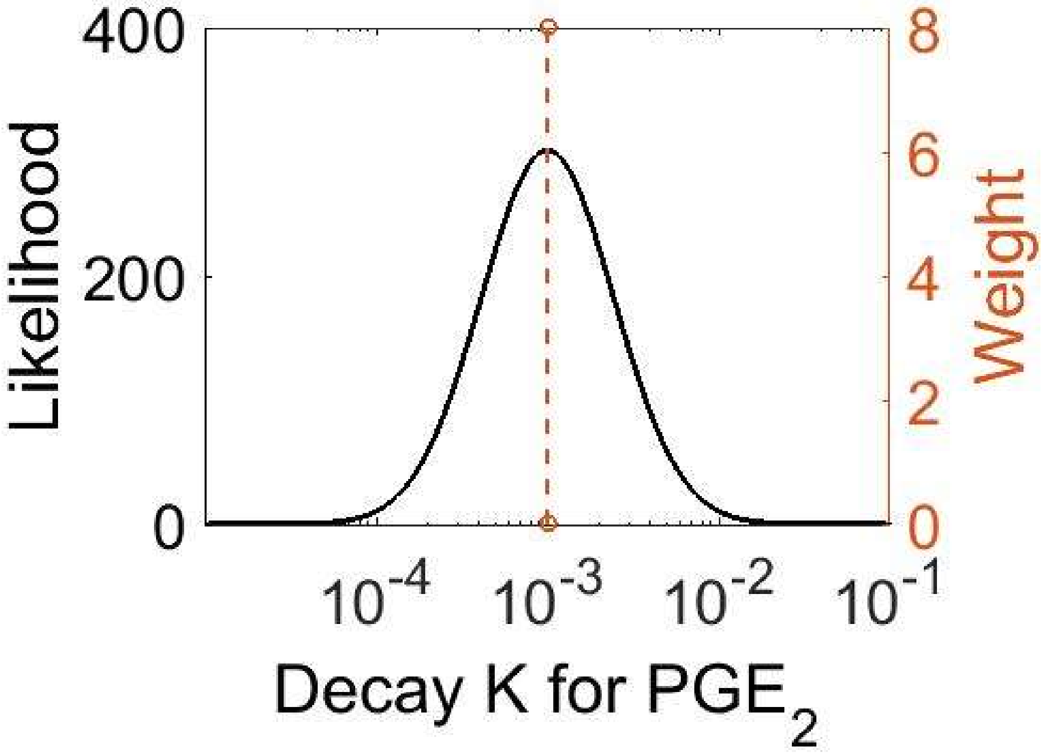


**Figure SF.9.27.1.1.1.** The estimated probability distribution for the $PGE_2$ decay constant. The value and weight of the literature values used to define the distribution are indicated by an orange dashed line. The x axis is plotted on a log-scale.

**S.9.28. Reaction 77: $PGI_2 \rightarrow \phi$**

The decay of $PGI_2$ in the intracellular compartment into metabolites outside of the scope of this model. An example of metabolites outside of the scope of this model includes metabolites which have been removed from the extracellular compartment into the systemic circulation or are degraded into metabolites not of interest to this model, the term decay reaction refers to the decrease in metabolite concentration by this collection of unspecified processes.

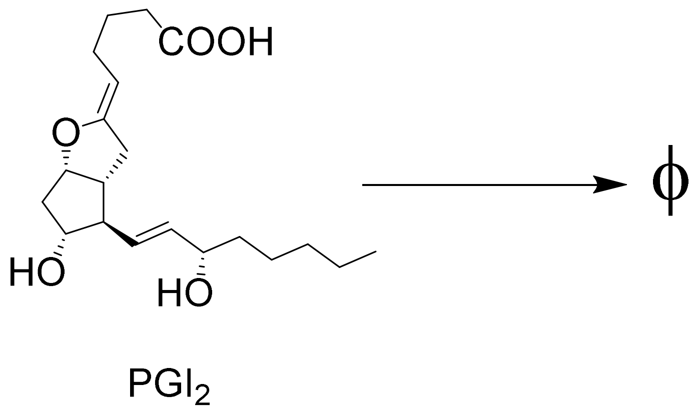


**Figure SF.9.28.** The decay of prostaglandin $I_2$ ($PGI_2$) in the intracellular compartment (Reaction 77).

**SEq.9.28.** Reaction rate law for Reaction 77.

$$\boldsymbol{v_{77} = K[\mathbf{PGI_2}]}$$

### S.9.28.1. Reaction parameters

#### S.9.28.1.1. Parameter: K

Parameter values for the K of Reaction 77 were obtained from the literature and summarised in Table ST.9.28.1.1.1. The log-normal distribution properties for the K of Reaction 77 are shown in Table ST.9.28.1.1.2 and plotted in Figure SF.9.28.1.1.1.

**Table ST.9.28.1.1.1.** Literature information used to design the $PGI_2$ decay constant parameter distribution. Each value was assigned a weight using the protocol described in Section 2.6.2. The type of error was assigned as 0 if it was additive and 1 if it was multiplicative.

| Half Life (min) | Error (min) | Rate constant ($min^{-1}$) | Error ($min^{-1}$) | Experimental details | | | | | | | Weight | Type of error | Reference |
|---|---|---|---|---|---|---|---|---|---|---|---|---|---|
| | | | | Experiment type | Substrate | Species | Expression Vector | pH | Temperature (°C) | Other | | | |
| 3 | | 0.231 | | Unknown | PGI2 | Unknown | Unknown | Unknown | Unknown | Textbook with no reference | 8 | | (Cawello et al., 1994) |

**Table ST.9.28.1.1.2.** The log-normal distribution properties of the $PGI_2$ decay constant distribution. These values were calculated using the functions described in Section 2.6.2.

| Mode ($min^{-1}$) | Confidence Interval | Location parameter (μ) | Scale parameter (σ) |
|---|---|---|---|
| $2.30 \times 10^{-1}$ | 1.10 | -1.46 | $9.49 \times 10^{-2}$ |

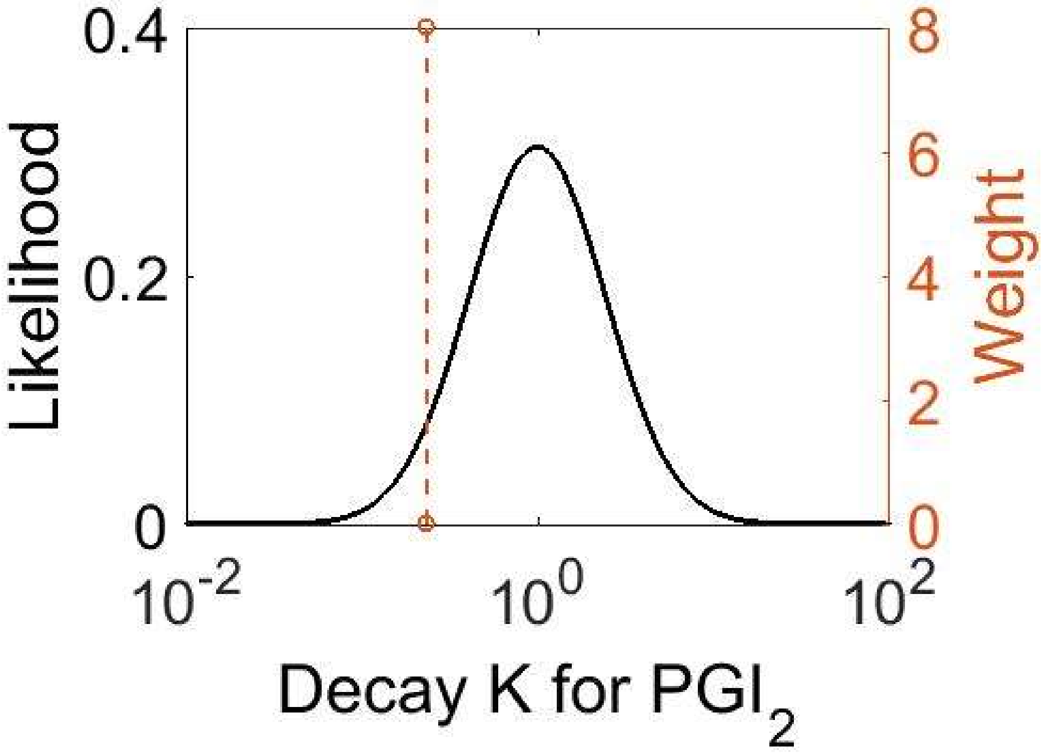


**Figure SF.9.28.1.1.1.** The estimated probability distribution for the $PGI_2$ decay constant. The value and weight of the literature values used to define the distribution are indicated by an orange dashed line. The x axis is plotted on a log-scale.

**S.9.29. Reaction 78: $PGD_2 \rightarrow \phi$**

The decay of $PGD_2$ in the intracellular compartment into metabolites outside of the scope of this model. An example of metabolites outside of the scope of this model includes metabolites which have been removed from the extracellular compartment into the systemic circulation or are degraded into metabolites not of interest to this model, the term decay reaction refers to the decrease in metabolite concentration by this collection of unspecified processes.

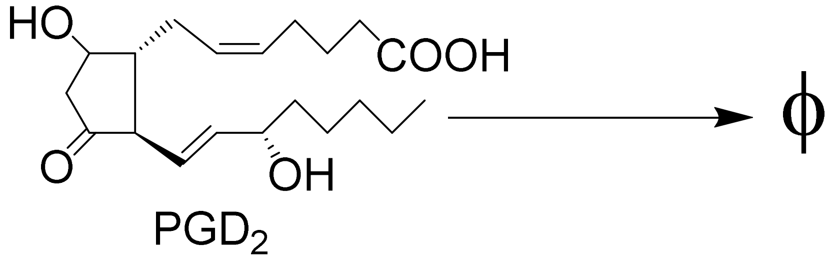


**Figure SF.9.29.** The decay of prostaglandin $D_2$ ($PGD_2$) in the intracellular compartment (Reaction 78).

**SEq.9.29.** Reaction rate law for Reaction 78.

$$\boldsymbol{v_{78} = K[\mathrm{PGD_2}]}$$

### S.9.29.1. Reaction parameters

#### S.9.29.1.1. Parameter: K

Parameter values for the K of Reaction 78 were obtained from the literature and summarised in Table ST.9.29.1.1.1. The log-normal distribution properties for the K of Reaction 78 are shown in Table ST.9.29.1.1.2 and plotted in Figure SF.9.29.1.1.1.

**Table ST.9.29.1.1.1.** Literature information used to design the $PGD_2$ decay constant parameter distribution. Each value was assigned a weight using the protocol described in Section 2.6.2. The type of error was assigned as 0 if it was additive and 1 if it was multiplicative.

| Half Life (min) | Error (min) | Rate constant ($min^{-1}$) | Error ($min^{-1}$) | Experimental details | | | | | | | Weight | Type of error | Reference |
|---|---|---|---|---|---|---|---|---|---|---|---|---|---|
| | | | | Experiment type | Substrate | Species | Expression Vector | pH | Temperature (°C) | Other | | | |
| 30 | | 0.023 | | In vivo | PGD2 | Human | Plasma | 7 | 37 | | 8 | | (Schuligoi et al., 2007) |
| 1.5 | | 0.462 | | In vivo | PGD2 | Human | Brain | 7.4 | Unknown | | 8 | | (Suzuki et al., 1986) |

**Table ST.9.29.1.1.2.** The log-normal distribution properties of the $PGD_2$ decay constant distribution. These values were calculated using the functions described in Section 2.6.2.

| Mode ($min^{-1}$) | Confidence Interval | Location parameter (μ) | Scale parameter (σ) |
|---|---|---|---|
| $2.30 \times 10^{-2}$ | 1.10 | -3.76 | $9.49 \times 10^{-2}$ |

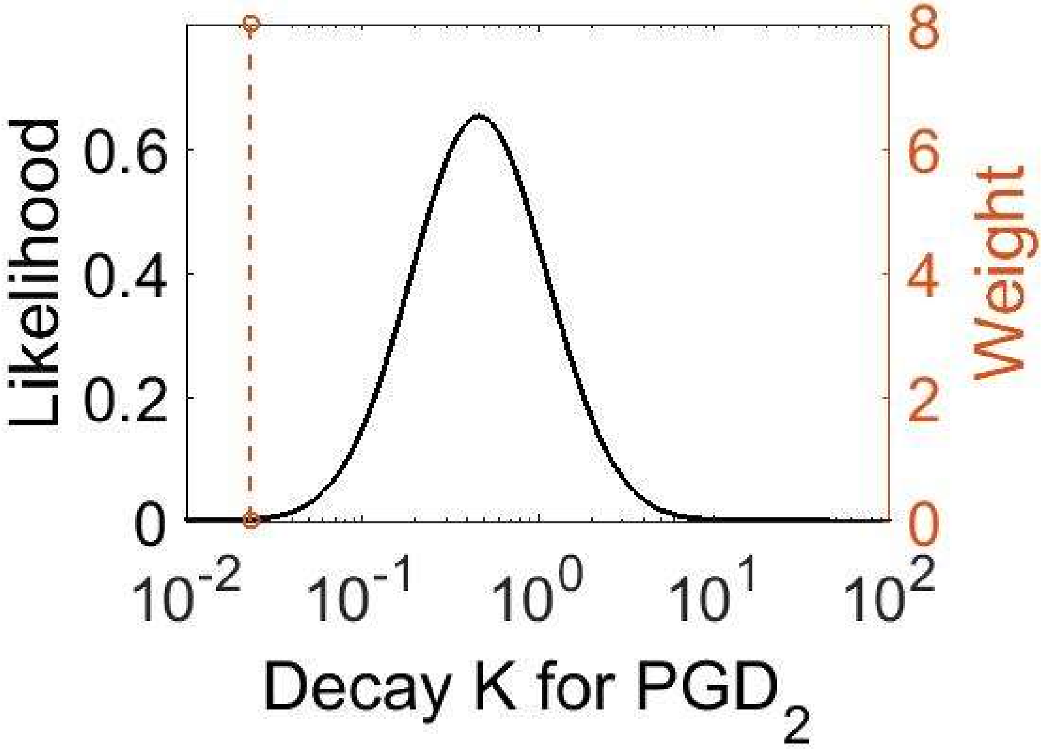


**Figure SF.9.29.1.1.1.** The estimated probability distribution for the $PGD_2$ decay constant. The value and weight of the literature values used to define the distribution are indicated by an orange dashed line. The x axis is plotted on a log-scale.

**S.9.30. Reaction 79: 15-keto-PGE$_2$ →ϕ**

The decay of 15-keto-PGE$_2$ in the intracellular compartment into metabolites outside of the scope of this model. An example of metabolites outside of the scope of this model includes metabolites which have been removed from the extracellular compartment into the systemic circulation or are degraded into metabolites not of interest to this model, the term decay reaction refers to the decrease in metabolite concentration by this collection of unspecified processes.

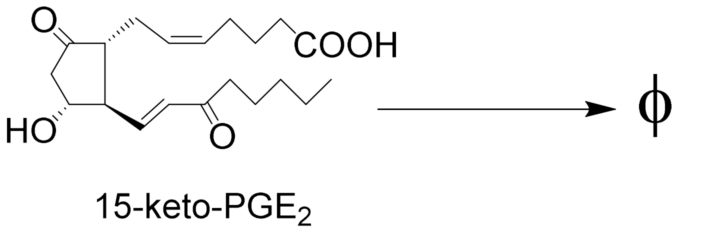


**Figure SF.9.30.** The decay of 15-keto-prostaglandin E$_2$ (15-keto-PGE$_2$) in the intracellular compartment (Reaction 79).

**SEq.9.30.** Reaction rate law for Reaction 79.

$$\boldsymbol{v_{79} = K[15 - \mathrm{keto} - \mathrm{PGE_2}]}$$

### S.9.30.1. Reaction parameters

#### S.9.30.1.1. Parameter: K

Parameter values for the K of Reaction 79 were obtained from the literature and summarised in Table ST.9.30.1.1.1. The log-normal distribution properties for the K of Reaction 79 are shown in Table ST.9.30.1.1.2 and plotted in Figure SF.9.30.1.1.1.

**Table ST.9.30.1.1.1.** Literature information used to design the 15-keto-$PGE_2$ decay constant parameter distribution. Each value was assigned a weight using the protocol described in Section 2.6.2. The type of error was assigned as 0 if it was additive and 1 if it was multiplicative.

| Half Life (min) | Error (min) | Rate constant ($min^{-1}$) | Error ($min^{-1}$) | Experimental details | | | | | | | Weight | Type of error | Reference |
|---|---|---|---|---|---|---|---|---|---|---|---|---|---|
| | | | | Experiment type | Substrate | Species | Expression Vector | pH | Temperature (°C) | Other | | | |
| 99 | | 0.007 | | In vivo | **13,14-Dihydro-15-Keto-PGE2** | Unknown | Unknown | Unknown | Unknown | | 8 | | (Bothwell et al., 1982) |

**Table ST.9.30.1.1.2** The log-normal distribution properties of the 15-keto-$PGE_2$ decay constant distribution. These values were calculated using the functions described in Section 2.6.2.

| Mode ($min^{-1}$) | Confidence Interval | Location parameter (μ) | Scale parameter (σ) |
|---|---|---|---|
| 6.97 $x10^{-3}$ | 1.10 | -4.96 | 9.49 $x10^{-2}$ |

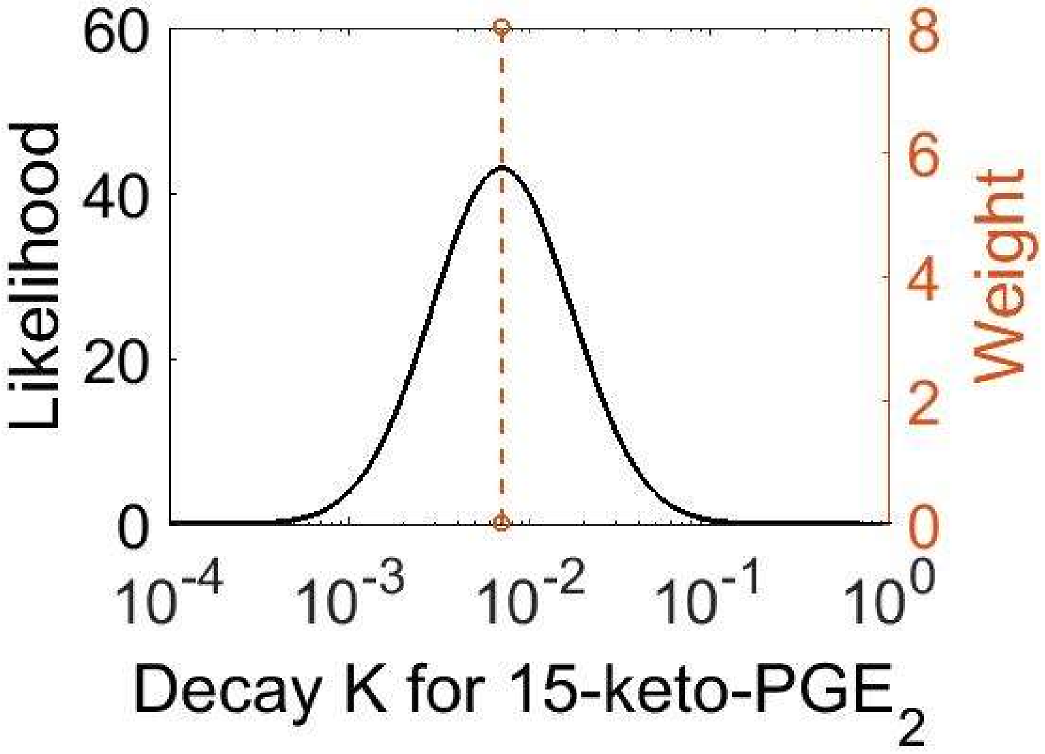


**Figure SF.9.30.1.1.1.** The estimated probability distribution for the 15-keto-$PGE_2$ decay constant. The value and weight of the literature values used to define the distribution are indicated by an orange dashed line. The x axis is plotted on a log-scale.

**S.9.31. Reaction 80: 13,14-dihydro-15-keto-PGE$_2$ →ϕ**

The decay of 13,14-dihydro-15-keto-PGE$_2$ in the intracellular compartment into metabolites outside of the scope of this model. An example of metabolites outside of the scope of this model includes metabolites which have been removed from the extracellular compartment into the systemic circulation or are degraded into metabolites not of interest to this model, the term decay reaction refers to the decrease in metabolite concentration by this collection of unspecified processes.

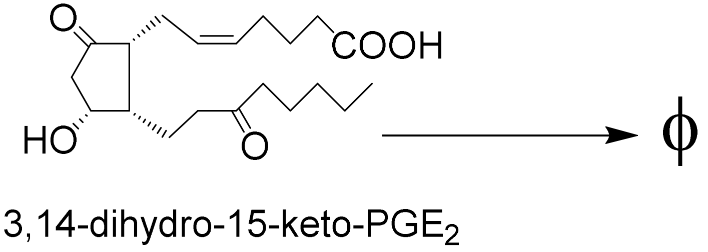


**Figure SF.9.31.** The decay of 13,14-dihydro-15-keto-prostaglandin E$_2$ (13,14-dihydro-15-keto-PGE$_2$) in the intracellular compartment (Reaction 80).

**SEq.9.31.** Reaction rate law for Reaction 80.

$$\boldsymbol{v_{80} = K[13,14 - \mathbf{dihydro} - 15 - \mathbf{keto} - \mathbf{PGE_2}\ ]}$$

### S.9.31.1. Reaction parameters

#### S.9.31.1.1. Parameter: K

Parameter values for the K of Reaction 80 were obtained from the literature and summarised in Table ST.9.31.1.1.1. The log-normal distribution properties for the K of Reaction 80 are shown in Table ST.9.31.1.1.2 and plotted in Figure SF.9.31.1.1.1.

**Table ST.9.31.1.1.1.** Literature information used to design the 13,14-dihydro-15-keto-$PGE_2$ decay constant parameter distribution. Each value was assigned a weight using the protocol described in Section 2.6.2. The type of error was assigned as 0 if it was additive and 1 if it was multiplicative.

| Half Life (min) | Error (min) | Rate constant ($min^{-1}$) | Error ($min^{-1}$) | Experimental details | | | | | | | Weight | Type of error | Reference |
|---|---|---|---|---|---|---|---|---|---|---|---|---|---|
| | | | | Experiment type | Substrate | Species | Expression Vector | pH | Temperature (°C) | Other | | | |
| 99 | | 0.007 | | In vivo | 13,14-Dihydro-15-Keto-PGE2 | Unknown | Unknown | Unknown | Unknown | | 8 | | (Bothwell et al., 1982) |

**Table ST.9.31.1.1.2.** The log-normal distribution properties of the 15-keto-$PGE_2$ decay constant distribution. These values were calculated using the functions described in Section 2.6.2.

| Mode ($min^{-1}$) | Confidence Interval | Location parameter (μ) | Scale parameter (σ) |
|---|---|---|---|
| $6.97 \times 10^{-3}$ | 1.10 | -4.96 | $9.49 \times 10^{-2}$ |

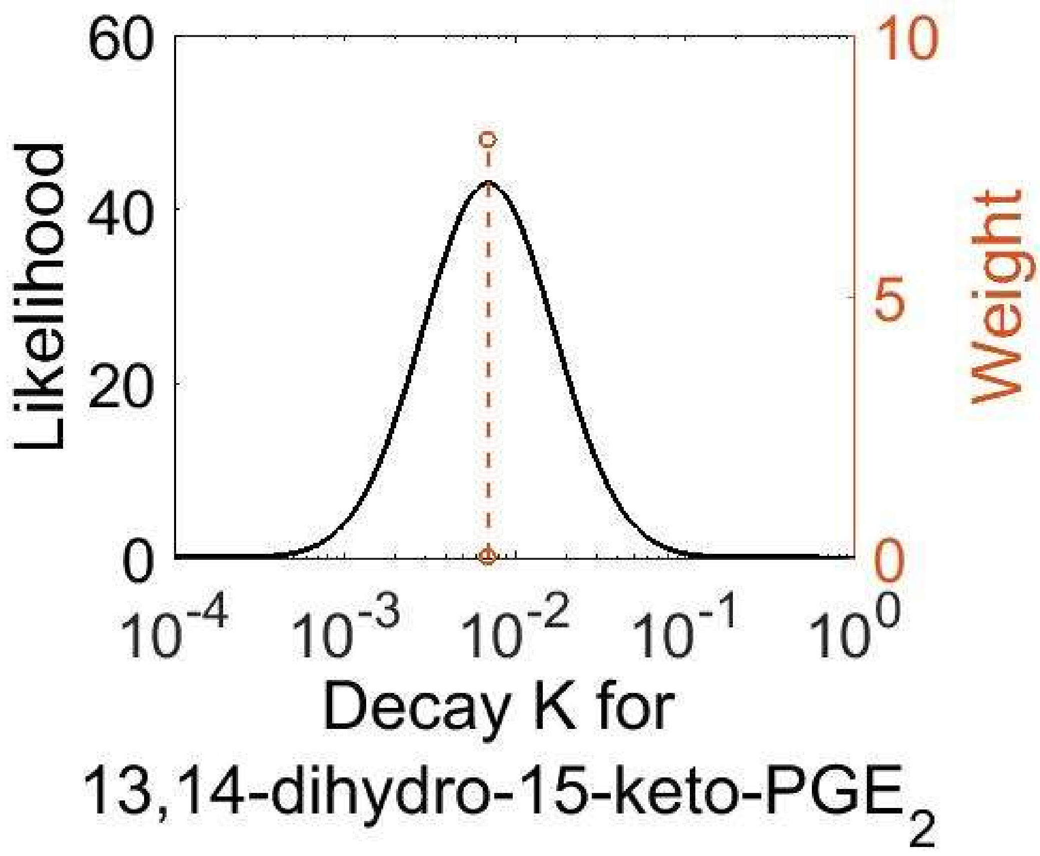


**Figure SF.9.31.1.1.1.** The estimated probability distribution for the 13,14-dihydro-15-keto-$PGE_2$ decay constant. The value and weight of the literature values used to define the distribution are indicated by an orange dashed line. The x axis is plotted on a log-scale.

**S.9.32. Reaction 81: $TXB_2 \rightarrow \phi$**

The decay of $TXB_2$ in the intracellular compartment into metabolites outside of the scope of this model. An example of metabolites outside of the scope of this model includes metabolites which have been removed from the extracellular compartment into the systemic circulation or are degraded into metabolites not of interest to this model, the term decay reaction refers to the decrease in metabolite concentration by this collection of unspecified processes.

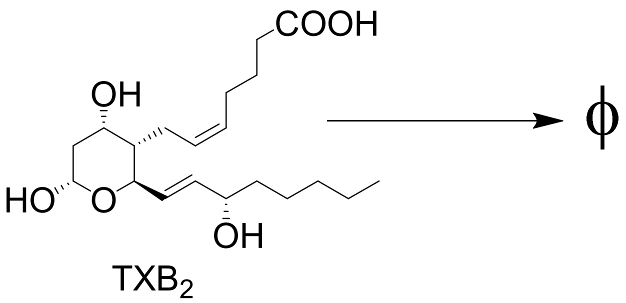


**Figure SF.9.32.** The decay of thromboxane $B_2$ ($TXB_2$) in the intracellular compartment (Reaction 81).

**SEq.9.32.** Reaction rate law for Reaction 81.

$$\boldsymbol{v_{81} = K[\mathrm{TXB_2}]}$$

### S.9.32.1. Reaction parameters

#### S.9.32.1.1. Parameter: K

Parameter values for the K of Reaction 81 were obtained from the literature and summarised in Table ST.9.32.1.1.1. The log-normal distribution properties for the K of Reaction 81 are shown in Table ST.9.32.1.1.2 and plotted in Figure SF.9.32.1.1.1.

**Table ST.9.32.1.1.1.** Literature information used to design the $TXB_2$ decay constant parameter distribution. Each value was assigned a weight using the protocol described in Section 2.6.2. The type of error was assigned as 0 if it was additive and 1 if it was multiplicative.

| Half Life (min) | Error (min) | Rate constant ($min^{-1}$) | Error ($min^{-1}$) | Experimental details | | | | | | | Weight | Type of error | Reference |
|---|---|---|---|---|---|---|---|---|---|---|---|---|---|
| | | | | Experiment type | Substrate | Species | Expression Vector | pH | Temperature (°C) | Other | | | |
| 20 | | 0.035 | | Unknown, assume in vitro | TXB2 | Unknown | Unknown | Unknown | Unknown | Textbook with no reference | 8 | | (Wild, 2005) |

**Table ST.9.32.1.1.2.** The log-normal distribution properties of the $TXB_2$ decay constant distribution. These values were calculated using the functions described in Section 2.6.2.

| Mode ($min^{-1}$) | Confidence Interval | Location parameter (μ) | Scale parameter (σ) |
|---|---|---|---|
| $3.48 \times 10^{-2}$ | 1.10 | -3.35 | $9.49 \times 10^{-2}$ |

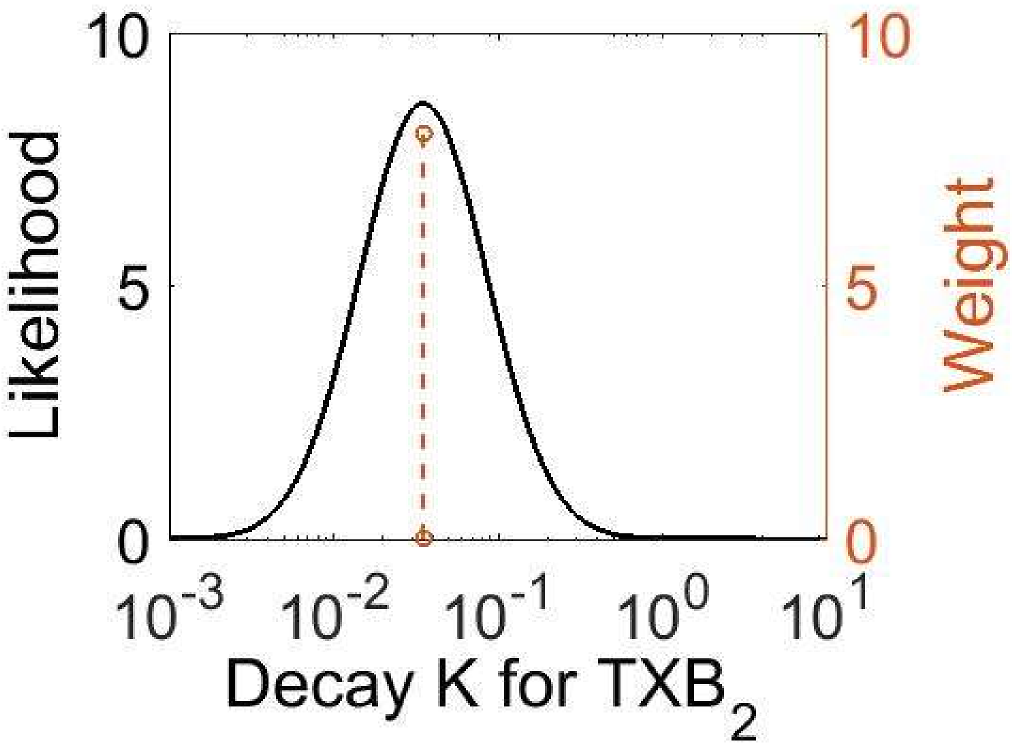


**Figure SF.9.32.1.1.1.** The estimated probability distribution for the $TXB_2$ decay constant. The value and weight of the literature values used to define the distribution are indicated by an orange dashed line. The x axis is plotted on a log-scale**.**

**S.9.33. Reaction 82: 6-keto-$PGF_{1\alpha}$→ϕ**

The decay of 6-keto-$PGF_{1\alpha}$ in the intracellular compartment into metabolites outside of the scope of this model. An example of metabolites outside of the scope of this model includes metabolites which have been removed from the extracellular compartment into the systemic circulation or are degraded into metabolites not of interest to this model, the term decay reaction refers to the decrease in metabolite concentration by this collection of unspecified processes.

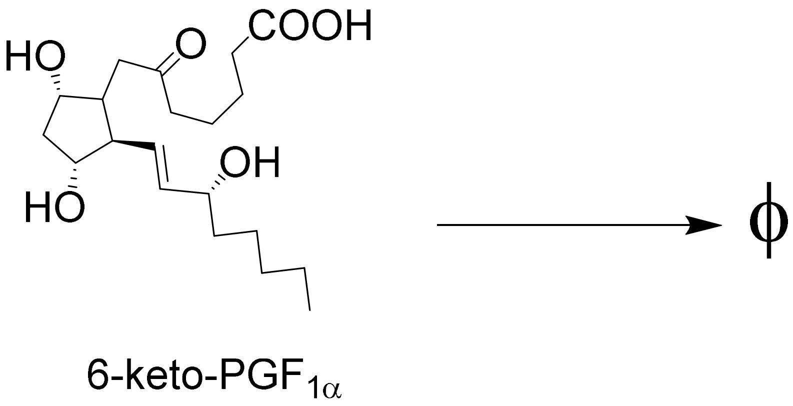


**Figure SF.9.33.** The decay of 6-keto-prostaglandin $F_{1\alpha}$ (6-keto-$PGF_{1\alpha}$) in the intracellular compartment (Reaction 82).

**SEq.9.33.** Reaction rate law for Reaction 82.

$$\boldsymbol{v_{82} = K[6 - \mathrm{keto} - \mathrm{PGF}_{1\alpha}]}$$

### S.9.33.1. Reaction parameters

#### S.9.33.1.1. Parameter: K

Parameter values for the K of Reaction 82 were obtained from the literature and summarised in Table ST.9.33.1.1.1. The log-normal distribution properties for the K of Reaction 82 are shown in Table ST.9.33.1.1.2 and plotted in Figure SF.9.33.1.1.1.

**Table ST.9.33.1.1.1.** Literature information used to design the 6-keto-PGF$_{1\alpha}$ decay constant parameter distribution. Each value was assigned a weight using the protocol described in Section 2.6.2. The type of error was assigned as 0 if it was additive and 1 if it was multiplicative.

| Half Life (min) | Error (min) | Rate constant (min$^{-1}$) | Error (min$^{-1}$) | Experimental details | | | | | | | Weight | Type of error | Reference |
|---|---|---|---|---|---|---|---|---|---|---|---|---|---|
| | | | | Experiment type | Substrate | Species | Expression Vector | pH | Temperature (°C) | Other | | | |
| 30 | | 0.023104906 | | In vivo | 6-KETO-PGF1A | Human | Plasma | Unknown | Unknown | | 8 | | (Ylikorkala and Viinikka, 1981) |

**Table ST.9.33.1.1.2.** The log-normal distribution properties of the 6-keto-PGF$_{1\alpha}$ decay constant distribution. These values were calculated using the functions described in Section 2.6.2.

| Mode (min$^{-1}$) | Confidence Interval | Location parameter (μ) | Scale parameter (σ) |
|---|---|---|---|
| 2.30 x10$^{-2}$ | 1.10 | -3.76 | 9.4910$^{-2}$ |

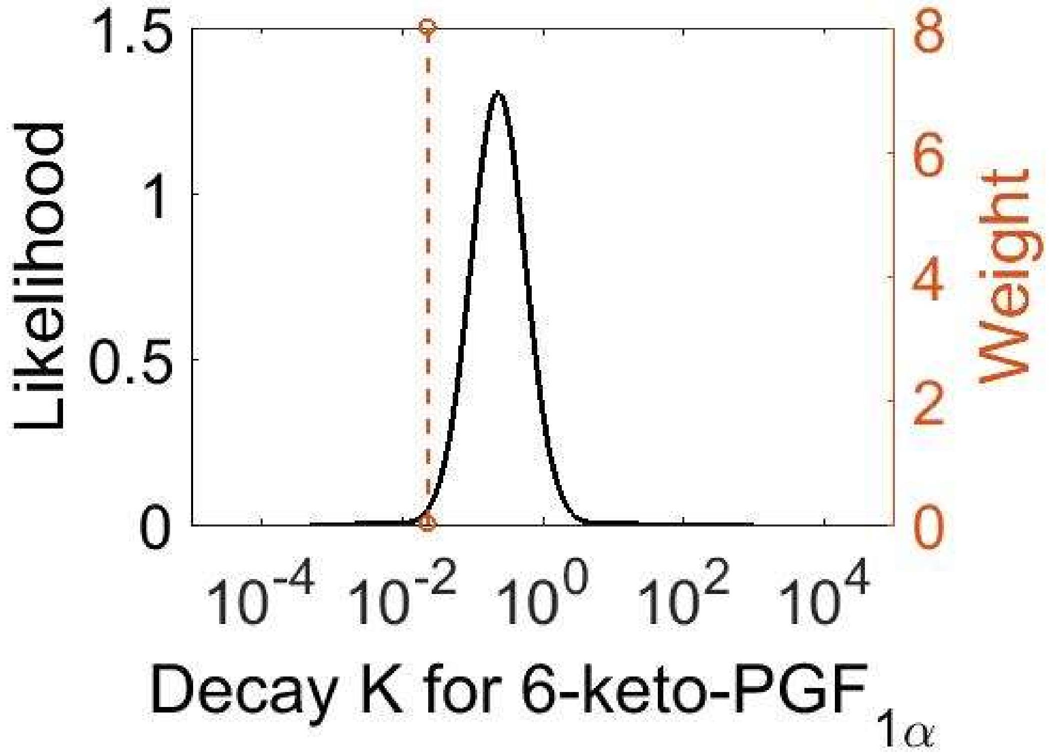


**Figure SF.9.33.1.1.1.** The estimated probability distribution for the 6-keto-$PGF_{1\alpha}$ decay constant. The value and weight of the literature values used to define the distribution are indicated by an orange dashed line. The x axis is plotted on a log-scale.

**S.9.34. Reaction 83: $PGJ_2 \rightarrow \phi$**

The decay of $PGJ_2$ in the intracellular compartment into metabolites outside of the scope of this model. An example of metabolites outside of the scope of this model includes metabolites which have been removed from the extracellular compartment into the systemic circulation or are degraded into metabolites not of interest to this model, the term decay reaction refers to the decrease in metabolite concentration by this collection of unspecified processes.

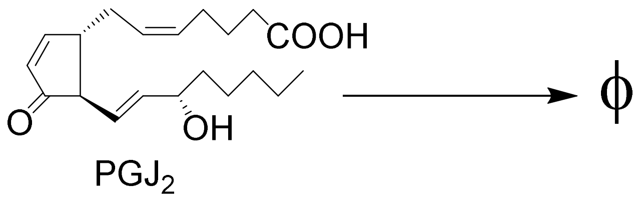


**Figure S.9.34. The decay of prostaglandin $J_2$ ($PGJ_2$) in the intracellular compartment (Reaction 83).**

**SEq.9.34.** Reaction rate law for Reaction 83.

$$\boldsymbol{v_{83} = K[\mathrm{PGJ_2}]}$$

### S.9.34.1. Reaction parameters

### S.9.34.1.1. Parameter: K

Parameter values for the K of Reaction 83 were obtained from the literature and summarised in Table ST.9.34.1.1.1. The log-normal distribution properties for the K of Reaction 83 are shown in Table ST.9.34.1.1.2 and plotted in Figure SF.9.34.1.1.1.

**Table ST.9.34.1.1.1.** Literature information used to design the $PGJ_2$ decay constant parameter distribution. Each value was assigned a weight using the protocol described in Section 2.6.2. The type of error was assigned as 0 if it was additive and 1 if it was multiplicative.

| Half Life (min) | Error (min) | Rate constant ($min^{-1}$) | Error ($min^{-1}$) | Experimental details | | | | | | | Weight | Type of error | Reference |
|---|---|---|---|---|---|---|---|---|---|---|---|---|---|
| | | | | Experiment type | Substrate | Species | Expression Vector | pH | Temperature (°C) | Other | | | |
| 720 | | 0.001 | | In vivo | 15-Deoxy-PGJ2 | Human | Albumin | 7.4 | 37 | | 8 | | (Fitzpatrick and Wynalda, 1983) |

**Table ST.9.34.1.1.2.** The log-normal distribution properties of the $PGJ_2$ decay constant distribution. These values were calculated using the functions described in Section 2.6.2.

| Mode ($min^{-1}$) | Confidence Interval | Location parameter (μ) | Scale parameter (σ) |
|---|---|---|---|
| $9.95 \times 10^{-4}$ | 1.10 | -6.90 | $9.4910^{-2}$ |

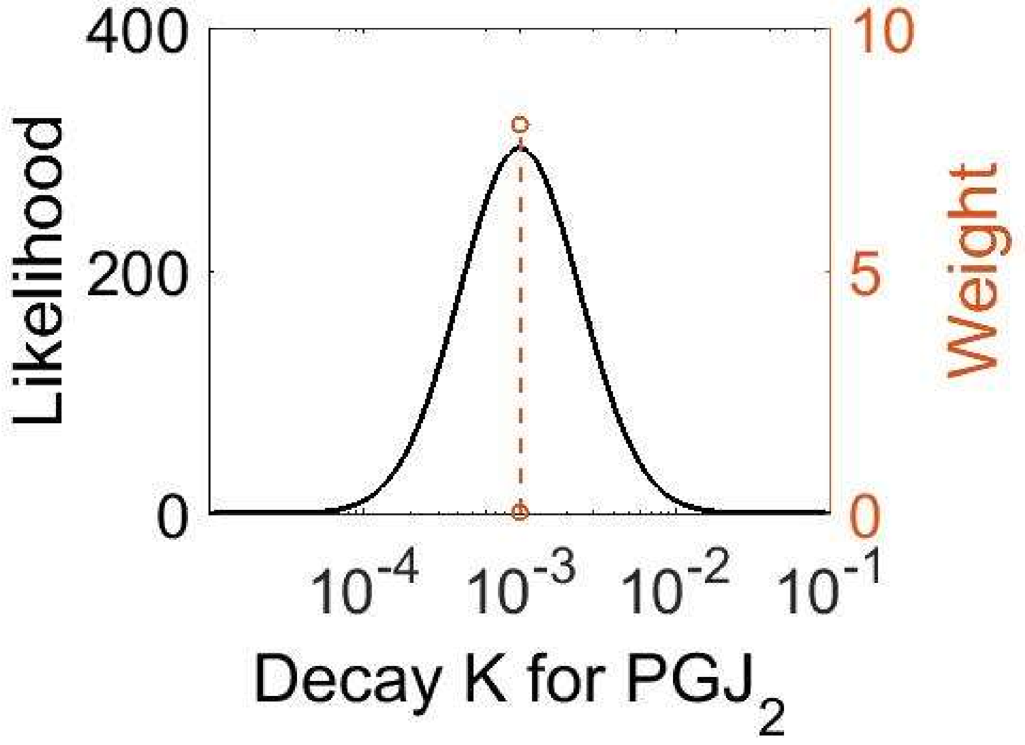


**Figure SF.9.34.1.1.1.** The estimated probability distribution for the $PGJ_2$ decay constant. The value and weight of the literature values used to define the distribution are indicated by an orange dashed line. The x axis is plotted on a log-scale.

**S.9.35. Reaction 84: 15-deoxy-PGJ$_2$ →ϕ**

The decay of 15-deoxy-PGJ$_2$ in the intracellular compartment into metabolites outside of the scope of this model. An example of metabolites outside of the scope of this model includes metabolites which have been removed from the extracellular compartment into the systemic circulation or are degraded into metabolites not of interest to this model, the term decay reaction refers to the decrease in metabolite concentration by this collection of unspecified processes.

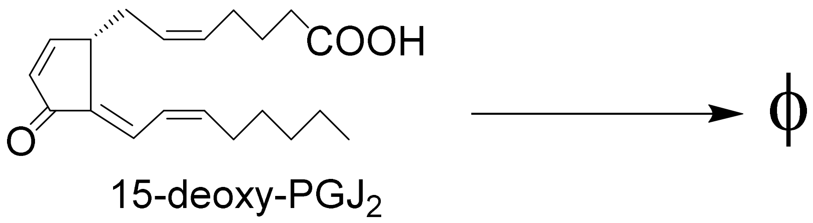


**Figure SF.9.35.** The decay of 15-deoxy-prostaglandin J$_2$ (15-deoxy-PGJ$_2$) in the intracellular compartment (Reaction 84).

**SEq.9.35.** Reaction rate law for Reaction 84.

$$\boldsymbol{v_{84} = K[15 - \mathbf{deoxy} - \mathbf{PGJ_2}\,]}$$

### S.9.35.1. Reaction parameters

**S.9.35.1.1 Parameter: K**

Parameter values for the K of Reaction 84 were obtained from the literature and summarised in Table ST.9.35.1.1.1. The log-normal distribution properties for the K of Reaction 84 are shown in Table ST.9.35.1.1.2 and plotted in Figure SF.9.35.1.1.1.

**Table ST.9.35.1.1.1.** Literature information used to design the 15-deoxy-$PGJ_2$ decay constant parameter distribution. Each value was assigned a weight using the protocol described in Section 2.6.2. The type of error was assigned as 0 if it was additive and 1 if it was multiplicative.

| Half Life (min) | Error (min) | Rate constant ($min^{-1}$) | Error ($min^{-1}$) | Experimental details | | | | | | | Weight | Type of error | Reference |
|---|---|---|---|---|---|---|---|---|---|---|---|---|---|
| | | | | Experiment type | Substrate | Species | Expression Vector | pH | Temperature (°C) | Other | | | |
| 720 | | 0.001 | | In vivo | 15-Deoxy-$PGJ_2$ | Human | Albumin | 7.4 | 37 | | 8 | | (Fitzpatrick and Wynalda, 1983) |

**Table ST.9.35.1.1.2** The log-normal distribution properties of the 15-deoxy-$PGJ_2$ decay constant distribution. These values were calculated using the functions described in Section 2.6.2.

| Mode ($min^{-1}$) | Confidence Interval | Location parameter (μ) | Scale parameter (σ) |
|---|---|---|---|
| $9.95 \times 10^{-4}$ | 1.10 | -6.90 | $9.4910^{-2}$ |

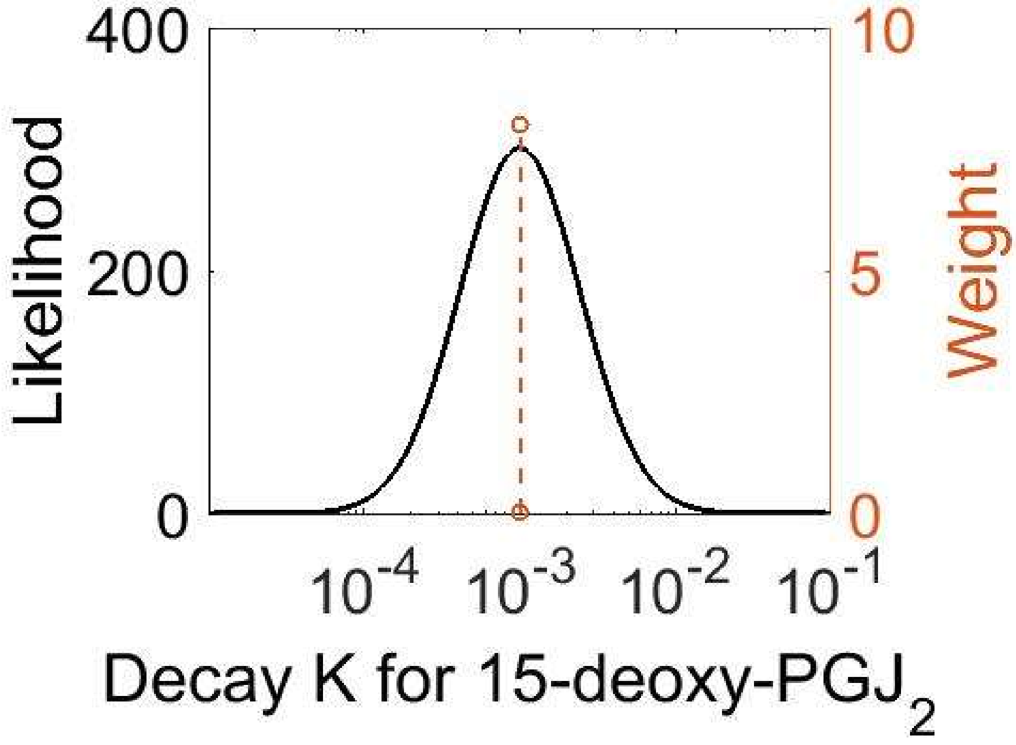


**Figure SF.9.35.1.1.1.** The estimated probability distribution for the 15-deoxy-$PGJ_2$ decay constant. The value and weight of the literature values used to define the distribution are indicated by an orange dashed line. The x axis is plotted on a log-scale.

**S.9.36. Reaction 85: 15-HPETE →ϕ**

The decay of 15-HPETE in the intracellular compartment into metabolites outside of the scope of this model. An example of metabolites outside of the scope of this model includes metabolites which have been removed from the extracellular compartment into the systemic circulation or are degraded into metabolites not of interest to this model, the term decay reaction refers to the decrease in metabolite concentration by this collection of unspecified processes.

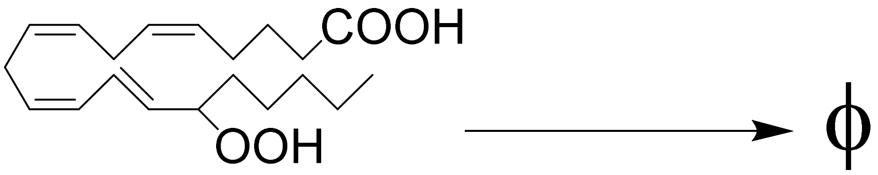


15-HPETE

**Figure SF.9.36.** The decay of 15-hydroperoxyeicosatetraenoic acid (15-HPETE) in the intracellular compartment (Reaction 85).

**SEq.9.36.** Reaction rate law for Reaction 85.

$\boldsymbol{v_{85} = K[15 - \mathrm{HPETE}\,]}$

### S.9.36.1. Reaction parameters

#### S.9.36.1.1. Parameter: K

Parameter values for the K of Reaction 85 were obtained from the literature and summarised in Table ST.9.36.1.1.1. The log-normal distribution properties for the K of Reaction 85 are shown in Table ST.9.36.1.1.2 and plotted in Figure SF.9.36.1.1.1.

**Table ST.9.36.1.1.1.** Literature information used to design the 15-HPETE decay constant parameter distribution. Each value was assigned a weight using the protocol described in Section 2.6.2. The type of error was assigned as 0 if it was additive and 1 if it was multiplicative.

| Half Life (min) | Error (min) | Rate constant ($min^{-1}$) | Error ($min^{-1}$) | Experimental details | | | | | | | Weight | Type of error | Reference |
|---|---|---|---|---|---|---|---|---|---|---|---|---|---|
| | | | | Experiment type | Substrate | Species | Expression Vector | pH | Temperature (°C) | Other | | | |
| 0.5 | | 1.386 | | In vivo | 12-HPETE | Unknown | Unknown | Unknown | Unknown | | 8 | | (Maclouf et al., 1982) |

**Table ST.9.36.1.1.2.** The log-normal distribution properties of the 15-HPETE decay constant distribution. These values were calculated using the functions described in Section 2.6.2.

| Mode ($min^{-1}$) | Confidence Interval | Location parameter (μ) | Scale parameter (σ) |
|---|---|---|---|
| 1.38 | 1.10 | 3.31 x$10^{-1}$ | 9.49$10^{-2}$ |

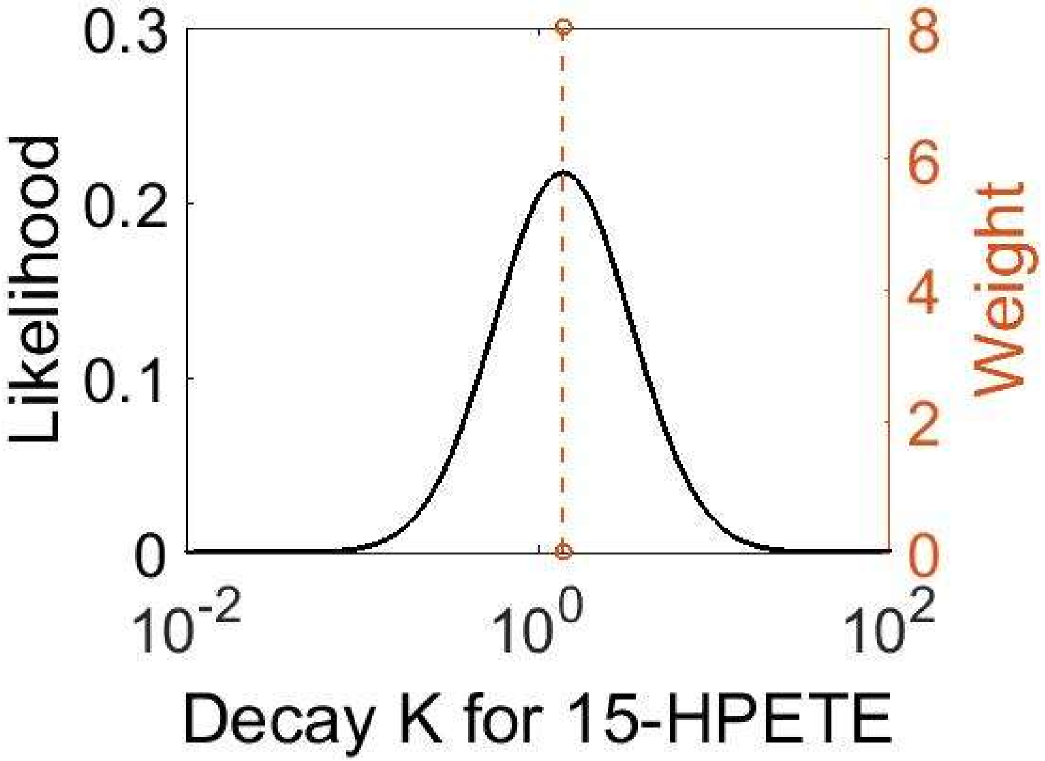


**Figure SF.9.36.1.1.1.** The estimated probability distribution for the 15-HPETE decay constant. The value and weight of the literature values used to define the distribution are indicated by an orange dashed line. The x axis is plotted on a log-scale.

**S.9.37. Reaction 86: 12-HPETE →ϕ**

The decay of 12-HPETE in the intracellular compartment into metabolites outside of the scope of this model. An example of metabolites outside of the scope of this model includes metabolites which have been removed from the extracellular compartment into the systemic circulation or are degraded into metabolites not of interest to this model, the term decay reaction refers to the decrease in metabolite concentration by this collection of unspecified processes.

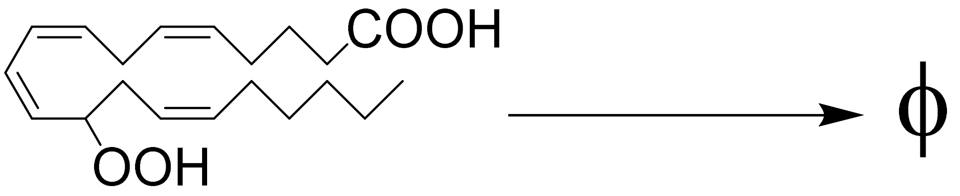


12-HPETE

**Figure SF.9.37.** The decay of 12-hydroperoxyeicosatetraenoic acid (12-HPETE) in the intracellular compartment (Reaction 86).

**SEq.9.37.** Reaction rate law for Reaction 86.

$$\boldsymbol{v_{86} = K[12 - \mathrm{HPETE}]}$$

### S.9.37.1. Reaction parameters

#### S.9.37.1.1. Parameter: K

Parameter values for the K of Reaction 86 were obtained from the literature and summarised in Table ST.9.37.1.1.1. The log-normal distribution properties for the K of Reaction 86 are shown in Table ST.9.37.1.1.2 and plotted in Figure SF.9.37.1.1.1.

**Table ST.9.37.1.1.1.** Literature information used to design the 12-HPETE decay constant parameter distribution. Each value was assigned a weight using the protocol described in Section 2.6.2. The type of error was assigned as 0 if it was additive and 1 if it was multiplicative.

| Half Life (min) | Error (min) | Rate constant ($min^{-1}$) | Error ($min^{-1}$) | Experimental details | | | | | | | Weight | Type of error | Reference |
|---|---|---|---|---|---|---|---|---|---|---|---|---|---|
| | | | | Experiment type | Substrate | Species | Expression Vector | pH | Temperature (°C) | Other | | | |
| 0.5 | | 1.386 | | Unknown | 12-HPETE | Unknown | Unknown | Unknown | Unknown | Textbook with no reference | 8 | | (Maclouf et al., 1982) |

**Table ST.9.37.1.1.2.** The log-normal distribution properties of the 12-HPETE decay constant distribution. These values were calculated using the functions described in Section 2.6.2.

| Mode ($min^{-1}$) | Confidence Interval | Location parameter (μ) | Scale parameter (σ) |
|---|---|---|---|
| $1.85 \times 10^{-1}$ | 7.46 | $-2.52 \times 10^{-1}$ | 1.20 |

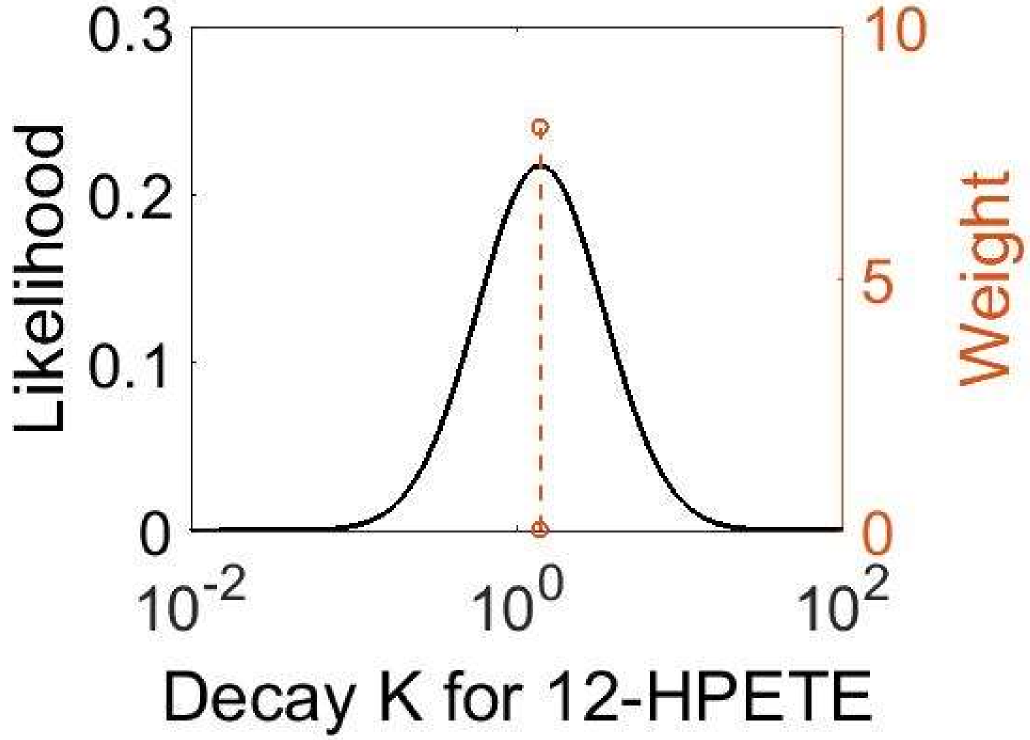


**Figure SF.9.37.1.1.1.** The estimated probability distribution for the 12-HPETE decay constant. The value and weight of the literature values used to define the distribution are indicated by an orange dashed line. The x axis is plotted on a log-scale.

**S.9.38. Reaction 87: 5-HPETE →ϕ**

The decay of 5-HPETE in the intracellular compartment into metabolites outside of the scope of this model. An example of metabolites outside of the scope of this model includes metabolites which have been removed from the extracellular compartment into the systemic circulation or are degraded into metabolites not of interest to this model, the term decay reaction refers to the decrease in metabolite concentration by this collection of unspecified processes.

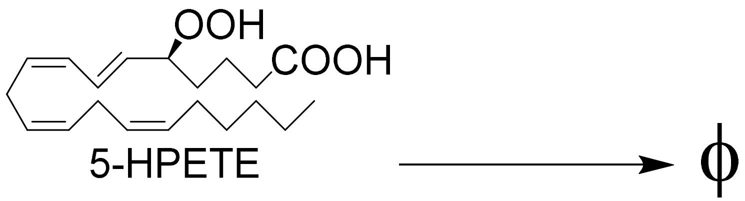


**Figure SF.9.38.** The decay of 5-hydroperoxyeicosatetraenoic acid (5-HPETE) in the intracellular compartment (Reaction 87).

**SEq.9.38.** Reaction rate law for Reaction 87.

$$\boldsymbol{v_{87} = K[5 - \mathrm{HPETE}]}$$

### S.9.38.1. Reaction parameters

#### S.9.38.1.1. Parameter: K

Parameter values for the K of Reaction 87 were obtained from the literature and summarised in Table ST.9.38.1.1.1. The log-normal distribution properties for the K of Reaction 87 are shown in Table ST.9.38.1.1.2 and plotted in Figure SF.9.38.1.1.1.

**Table ST.9.38.1.1.1.** Literature information used to design the 5-HPETE decay constant parameter distribution. Each value was assigned a weight using the protocol described in Section 2.6.2. The type of error was assigned as 0 if it was additive and 1 if it was multiplicative.

| Half Life (min) | Error (min) | Rate constant ($min^{-1}$) | Error ($min^{-1}$) | Experimental details | | | | | | | Weight | Type of error | Reference |
|---|---|---|---|---|---|---|---|---|---|---|---|---|---|
| | | | | Experiment type | Substrate | Species | Expression Vector | pH | Temperature (°C) | Other | | | |
| 0.5 | | 1.386 | | In vivo | 12-HPETE | Unknown | Unknown | Unknown | Unknown | | 8 | | (Maclouf et al., 1982) |

**Table ST.9.38.1.1.2.** The log-normal distribution properties of the 5-HPETE decay constant distribution. These values were calculated using the functions described in Section 2.6.2.

| Mode ($min^{-1}$) | Confidence Interval | Location parameter (μ) | Scale parameter (σ) |
|---|---|---|---|
| 1.38 | 1.10 | $3.31 \times 10^{-1}$ | $9.4910^{-2}$ |

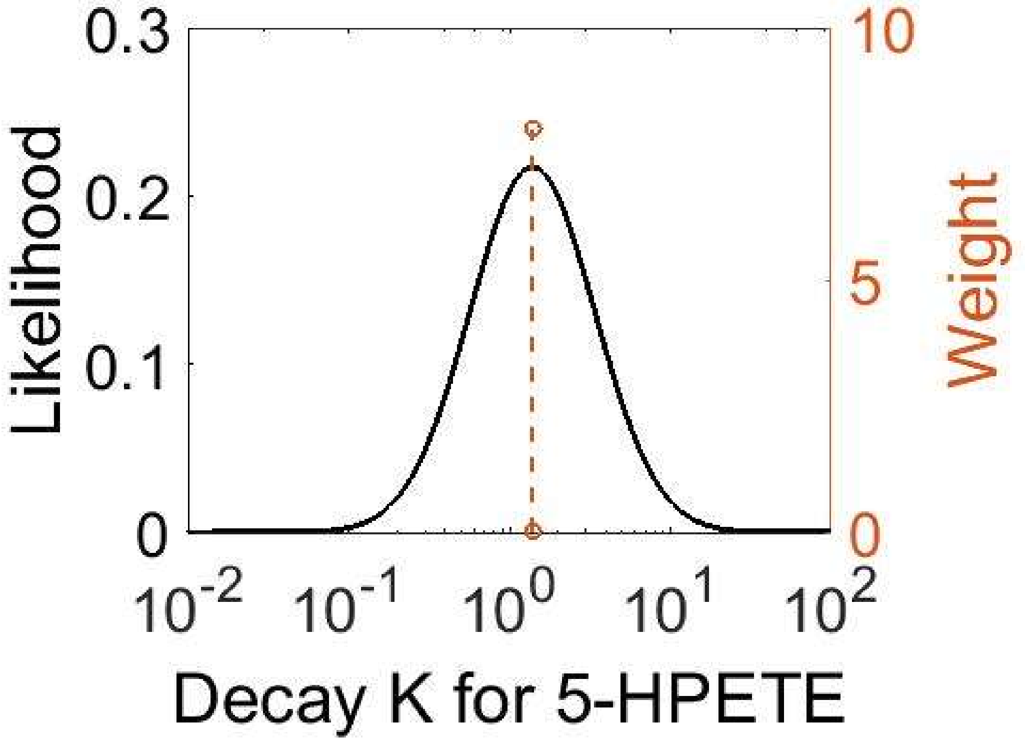


**Figure SF.9.38.1.1.1.** The estimated probability distribution for the 5-HPETE decay constant. The value and weight of the literature values used to define the distribution are indicated by an orange dashed line. The x axis is plotted on a log-scale.

**S.9.39. Reaction 88: 15-HETE →ϕ**

The decay of 15-HETE in the intracellular compartment into metabolites outside of the scope of this model. An example of metabolites outside of the scope of this model includes metabolites which have been removed from the extracellular compartment into the systemic circulation or are degraded into metabolites not of interest to this model, the term decay reaction refers to the decrease in metabolite concentration by this collection of unspecified processes.

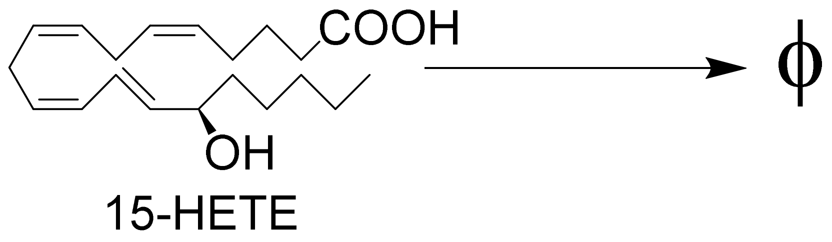


**Figure SF.9.39.** The decay of 15-hydroxyeicosatetraenoic acid (15-HETE) in the intracellular compartment (Reaction 88).

**SEq.9.39.** Reaction rate law for Reaction 88.

$$\boldsymbol{v_{88} = K[15 - \mathrm{HETE}]}$$

### S.9.39.1. Reaction parameters

#### S.9.39.1.1. Parameter: K

Parameter values for the K of Reaction 88 were obtained from the literature and summarised in Table ST.9.39.1.1.1. The log-normal distribution properties for the K of Reaction 88 are shown in Table ST.9.39.1.1.2 and plotted in Figure SF.9.39.1.1.1.

**Table ST.9.39.1.1.1.** Literature information used to design the 15-HETE decay constant parameter distribution. Each value was assigned a weight using the protocol described in Section 2.6.2. The type of error was assigned as 0 if it was additive and 1 if it was multiplicative.

| Half Life (min) | Error (min) | Rate constant ($min^{-1}$) | Error ($min^{-1}$) | Experimental details | | | | | | | Weight | Type of error | Reference |
|---|---|---|---|---|---|---|---|---|---|---|---|---|---|
| | | | | Experiment type | Substrate | Species | Expression Vector | pH | Temperature (°C) | Other | | | |
| 21 | | 0.033 | | In vivo | 15-HETE | Human | R15L Cells | Unknown | 37 | | 8 | | (Wei et al., 2009) |

**Table ST.9.39.1.1.2.** The log-normal distribution properties of the 15-HETE decay constant distribution. These values were calculated using the functions described in Section 2.6.2.

| Mode ($min^{-1}$) | Confidence Interval | Location parameter (μ) | Scale parameter (σ) |
|---|---|---|---|
| $3.30 \times 10^{-2}$ | 1.10 | -3.40 | $9.4910^{-2}$ |

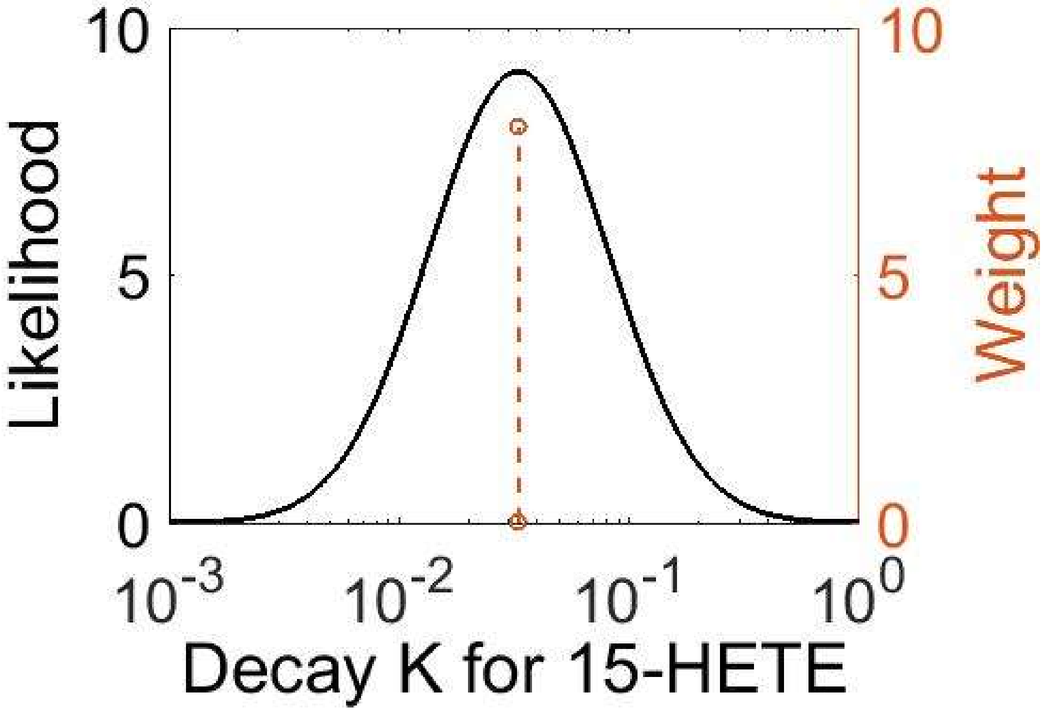


**Figure SF.9.39.1.1.1.** The estimated probability distribution for the 15-HETE decay constant. The value and weight of the literature values used to define the distribution are indicated by an orange dashed line. The x axis is plotted on a log-scale.

**S.9.40. Reaction 89: 12-HETE →ϕ**

The decay of 12-HETE in the intracellular compartment into metabolites outside of the scope of this model. An example of metabolites outside of the scope of this model includes metabolites which have been removed from the extracellular compartment into the systemic circulation or are degraded into metabolites not of interest to this model, the term decay reaction refers to the decrease in metabolite concentration by this collection of unspecified processes.

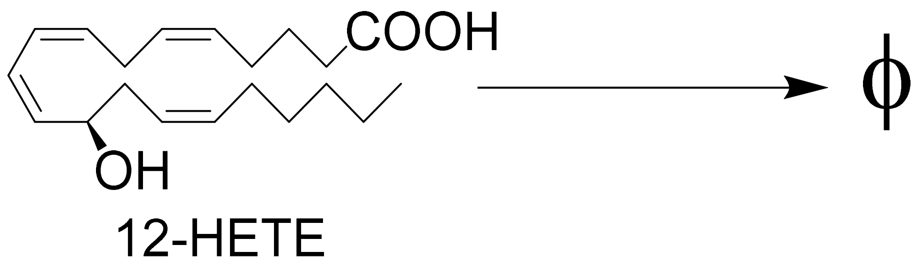


**Figure SF.9.40.** The decay of 12-hydroxyeicosatetraenoic acid (12-HETE) in the intracellular compartment (Reaction 89).

**SEq.9.40.** Reaction rate law for Reaction 89.

$$\boldsymbol{v_{89} = K[12 - \mathrm{HETE}]}$$

### S.9.40.1. Reaction parameters

#### S.9.40.1.1. Parameter: K

Parameter values for the K of Reaction 89 were obtained from the literature and summarised in Table ST.9.40.1.1.1. The log-normal distribution properties for the K of Reaction 89 are shown in Table ST.9.40.1.1.2 and plotted in Figure SF.9.40.1.1.1.

**Table ST.9.40.1.1.1.** Literature information used to design the 12-HETE decay constant parameter distribution. Each value was assigned a weight using the protocol described in Section 2.6.2. The type of error was assigned as 0 if it was additive and 1 if it was multiplicative.

| Half Life (min) | Error (min) | Rate constant ($min^{-1}$) | Error ($min^{-1}$) | Experimental details | | | | | | | Weight | Type of error | Reference |
|---|---|---|---|---|---|---|---|---|---|---|---|---|---|
| | | | | Experiment type | Substrate | Species | Expression Vector | pH | Temperature (°C) | Other | | | |
| 180 | | 0.004 | | In vivo | 12-HETE | Rabbit | Platelets | Unknown | 37 | | 8 | | (Dadaian et al., 1998) |

**Table ST.9.40.1.1.2.** The log-normal distribution properties of the 12-HETE decay constant distribution. These values were calculated using the functions described in Section 2.6.2.

| Mode ($min^{-1}$) | Confidence Interval | Location parameter (μ) | Scale parameter (σ) |
|---|---|---|---|
| $3.83 \times 10^{-3}$ | 1.10 | -5.56 | $9.49 \times 10^{-2}$ |

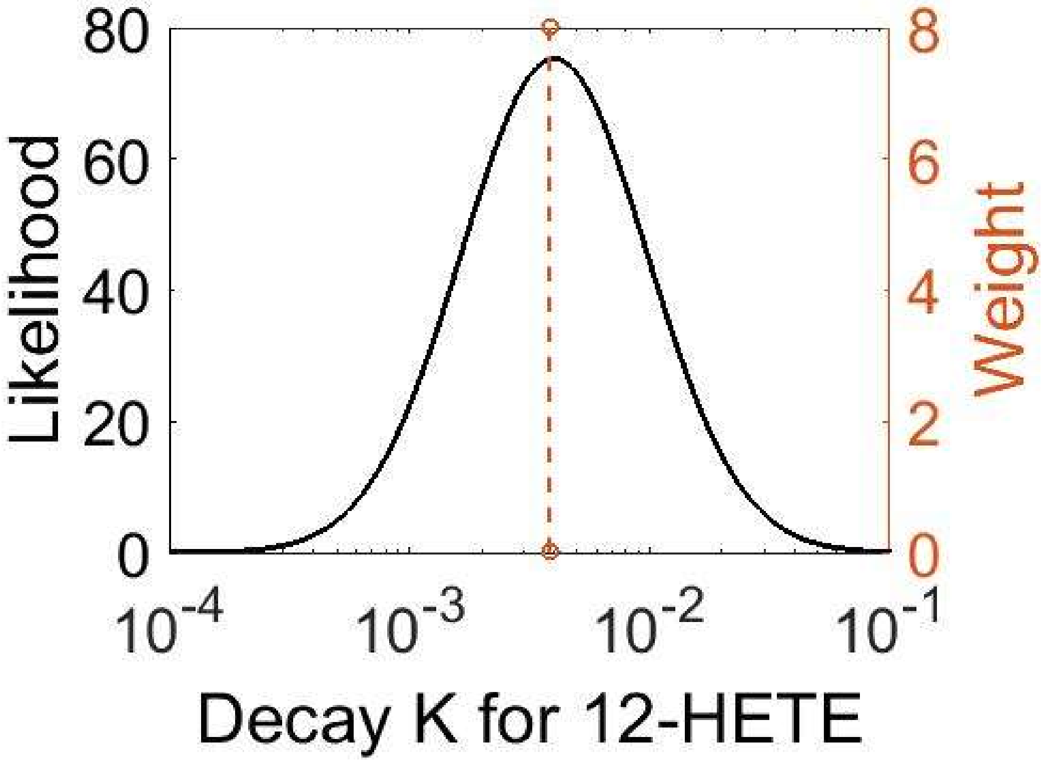


**Figure SF.9.40.1.1.1.** The estimated probability distribution for the 12-HETE decay constant. The value and weight of the literature values used to define the distribution are indicated by an orange dashed line. The x axis is plotted on a log-scale.

**S.9.41. Reaction 90: 5-HETE→ϕ**
The decay of 5-HETE in the intracellular compartment into metabolites outside of the scope of this model. An example of metabolites outside of the scope of this model includes metabolites which have been removed from the extracellular compartment into the systemic circulation or are degraded into metabolites not of interest to this model, the term decay reaction refers to the decrease in metabolite concentration by this collection of unspecified processes.

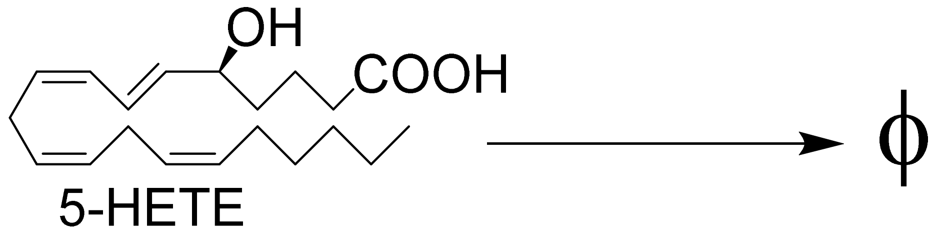


**Figure SF.9.41.** The decay of 5-hydroxyeicosatetraenoic acid (5-HETE) in the intracellular compartment (Reaction 90).

**SEq.9.41.** Reaction rate law for Reaction 90.
$$\boldsymbol{v_{90} = K[5 - \mathrm{HETE}]}$$

### S.9.41.1. Reaction parameters

#### S.9.41.1.1. Parameter: K

Parameter values for the K of Reaction 90 were obtained from the literature and summarised in Table ST.9.41.1.1.1. The log-normal distribution properties for the K of Reaction 90 are shown in Table ST.9.41.1.1.2 and plotted in Figure SF.9.41.1.1.1.

**Table ST.9.41.1.1.1.** Literature information used to design the 5-HETE decay constant parameter distribution. Each value was assigned a weight using the protocol described in Section 2.6.2. The type of error was assigned as 0 if it was additive and 1 if it was multiplicative.

| Half Life (min) | Error (min) | Rate constant ($min^{-1}$) | Error ($min^{-1}$) | Experimental details | | | | | | | Weight | Type of error | Reference |
|---|---|---|---|---|---|---|---|---|---|---|---|---|---|
| | | | | Experiment type | Substrate | Species | Expression Vector | pH | Temperature (°C) | Other | | | |
| 21 | | 0.033 | | In vivo | 15-HETE | Human | R15L Cells | Unknown | 37 | | 8 | | (Wei et al., 2009) |

**Table ST.9.41.1.1.2.** The log-normal distribution properties of the 5-HETE decay constant distribution. These values were calculated using the functions described in Section 2.6.2.

| Mode ($min^{-1}$) | Confidence Interval | Location parameter (μ) | Scale parameter (σ) |
|---|---|---|---|
| $3.30 \times 10^{-2}$ | 1.10 | -3.40 | $9.4910^{-2}$ |

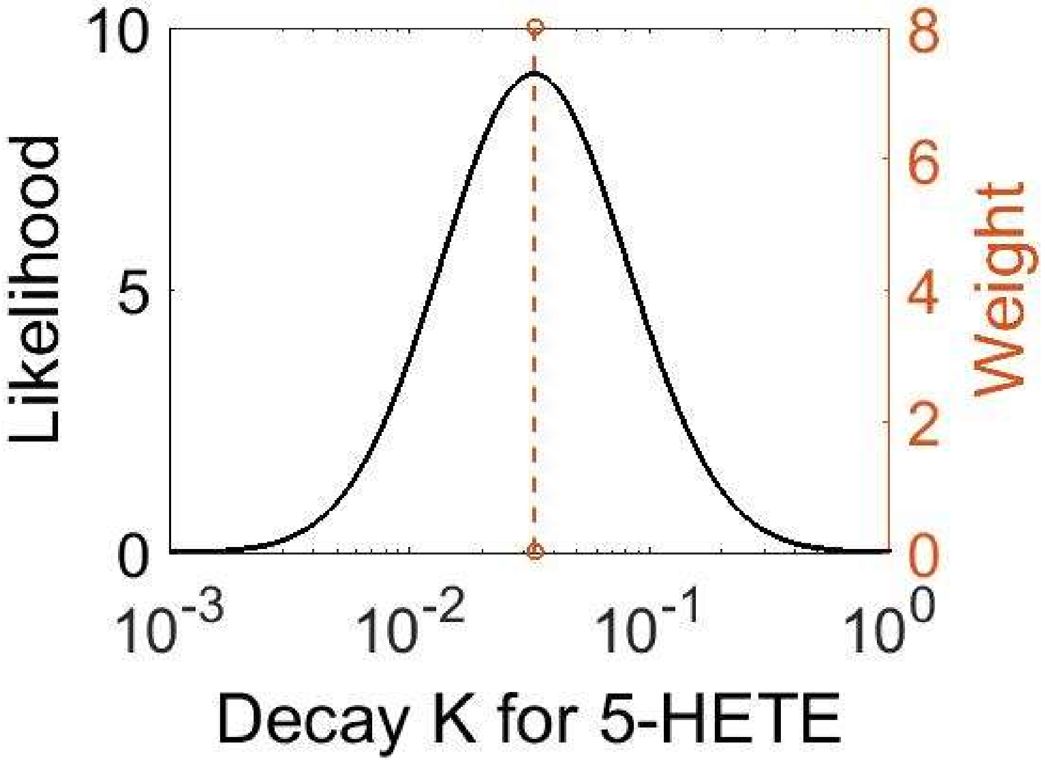


**Figure SF.9.41.1.1.1.** The estimated probability distribution for the 5-HETE decay constant. The value and weight of the literature values used to define the distribution are indicated by an orange dashed line. The x axis is plotted on a log-scale.

**S.9.42. Reaction 91: 5-oxo-ETE→ϕ**
The decay of 5-oxo-ETE in the intracellular compartment into metabolites outside of the scope of this model. An example of metabolites outside of the scope of this model includes metabolites which have been removed from the extracellular compartment into the systemic circulation or are degraded into metabolites not of interest to this model, the term decay reaction refers to the decrease in metabolite concentration by this collection of unspecified processes.

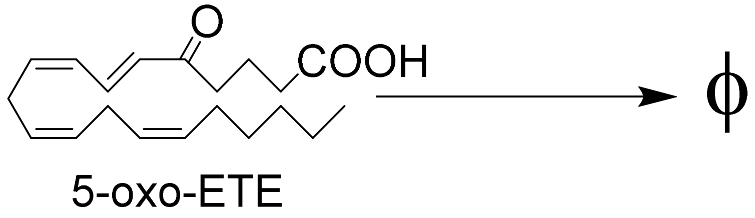


**Figure SF.9.42.** The decay of 5-oxo-eicosatetraenoic acid (5-oxo-ETE) in the intracellular compartment (Reaction 91).

**SEq.9.42.** Reaction rate law for Reaction 91.
$\boldsymbol{v_{91} = K[5 - \mathbf{oxo} - \mathbf{ETE}]}$

### S.9.42.1. Reaction parameters

#### S.9.42.1.1. Parameter: K

Parameter values for the K of Reaction 91 were obtained from the literature and summarised in Table ST.9.42.1.1.1. The log-normal distribution properties for the K of Reaction 91 are shown in Table ST.9.42.1.1.2 and plotted in Figure SF.9.42.1.1.1.

**Table ST.9.42.1.1.1.** Literature information used to design the 5-oxo-ETE decay constant parameter distribution. Each value was assigned a weight using the protocol described in Section 2.6.2. The type of error was assigned as 0 if it was additive and 1 if it was multiplicative.

| Half Life (min) | Error (min) | Rate constant ($min^{-1}$) | Error ($min^{-1}$) | Experimental details | | | | | | | Weight | Type of error | Reference |
|---|---|---|---|---|---|---|---|---|---|---|---|---|---|
| | | | | Experiment type | Substrate | Species | Expression Vector | pH | Temperature (°C) | Other | | | |
| 11 | | 0.064 | | In vivo | 15-OXO-ETE | Human | R15L Cells | Unknown | 37 | | 8 | | (Wei et al., 2009) |

**Table ST.9.42.1.1.2.** The log-normal distribution properties of the 5-oxo-ETE decay constant distribution. These values were calculated using the functions described in Section 2.6.2.

| Mode ($min^{-1}$) | Confidence Interval | Location parameter (μ) | Scale parameter (σ) |
|---|---|---|---|
| $6.37 \times 10^{-2}$ | 1.10 | -2.74 | $9.4910^{-2}$ |

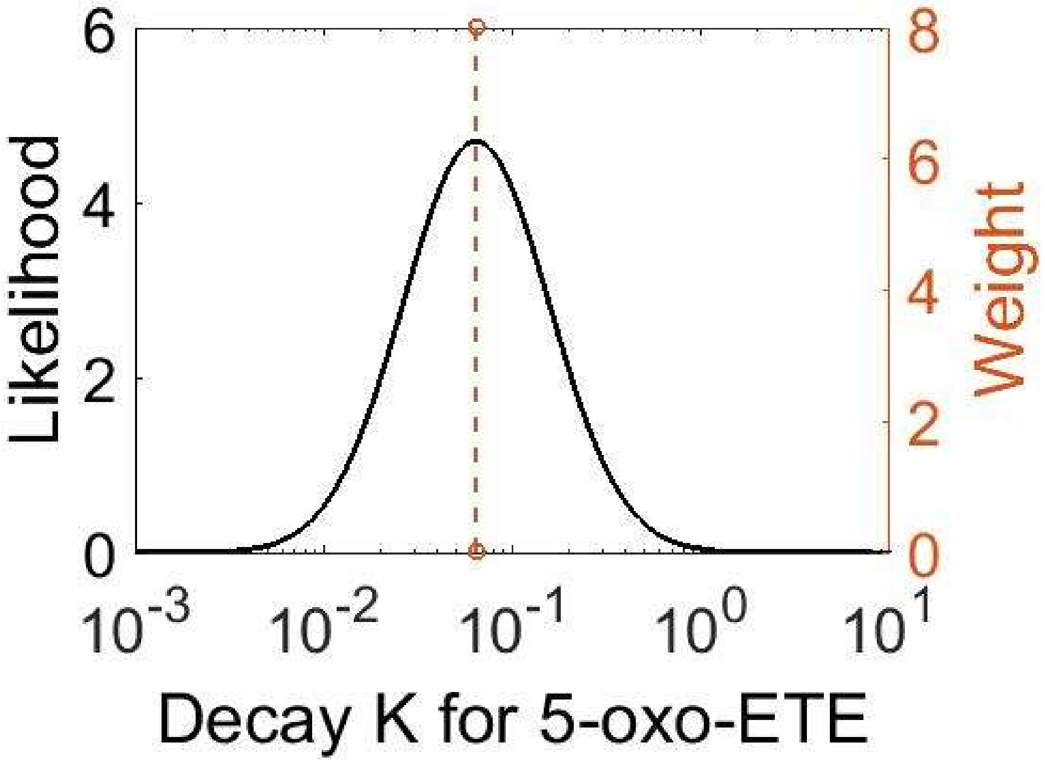


**Figure SF.9.42.1.1.1.** The estimated probability distribution for the 5-oxo-ETE decay constant. The value and weight of the literature values used to define the distribution are indicated by an orange dashed line. The x axis is plotted on a log-scale.

**S.9.43. Reaction 92: $LTA_4 \rightarrow \phi$**

The decay of $LTA_4$ in the intracellular compartment into metabolites outside of the scope of this model. An example of metabolites outside of the scope of this model includes metabolites which have been removed from the extracellular compartment into the systemic circulation or are degraded into metabolites not of interest to this model, the term decay reaction refers to the decrease in metabolite concentration by this collection of unspecified processes.

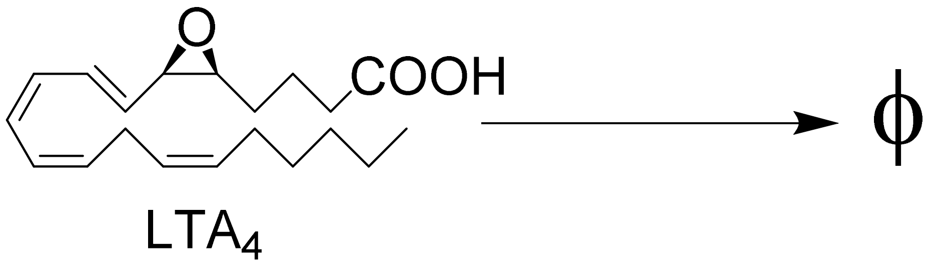


**Figure SF.9.43.** The decay of leukotriene $A_4$ ($LTA_4$) in the intracellular compartment (Reaction 92).

**SEq.9.43.** Reaction rate law for Reaction 92.

$$\boldsymbol{\nu_{92} = K[\mathrm{LTA_4}]}$$

### S.9.43.1. Reaction parameters

### S.9.43.1.1. Parameter: K

Parameter values for the K of Reaction 92 were obtained from the literature and summarised in Table ST.9.43.1.1.1. The log-normal distribution properties for the K of Reaction 92 are shown in Table ST.9.43.1.1.2 and plotted in Figure SF.9.43.1.1.1.

**Table ST.9.43.1.1.1.** Literature information used to design the $LTA_4$ decay constant parameter distribution. Each value was assigned a weight using the protocol described in Section 2.6.2. The type of error was assigned as 0 if it was additive and 1 if it was multiplicative.

| Half Life (min) | Error (min) | Rate constant ($min^{-1}$) | Error ($min^{-1}$) | Experimental details | | | | | | | Weight | Type of error | Reference |
|---|---|---|---|---|---|---|---|---|---|---|---|---|---|
| | | | | Experiment type | Substrate | Species | Expression Vector | pH | Temperature (°C) | Other | | | |
| 0.05 | | 13.863 | | In vivo | $LTA_4$ | Unknown | Unknown | 7 | 37 | | 8 | | (Dickinson Zimmer et al., 2004) |

**Table ST.9.43.1.1.2.** The log-normal distribution properties of the $LTA_4$ decay constant distribution. These values were calculated using the functions described in Section 2.6.2.

| Mode ($min^{-1}$) | Confidence Interval | Location parameter (μ) | Scale parameter (σ) |
|---|---|---|---|
| $1.38 \times 10^{1}$ | 1.10 | 2.63 | $9.4910^{-2}$ |

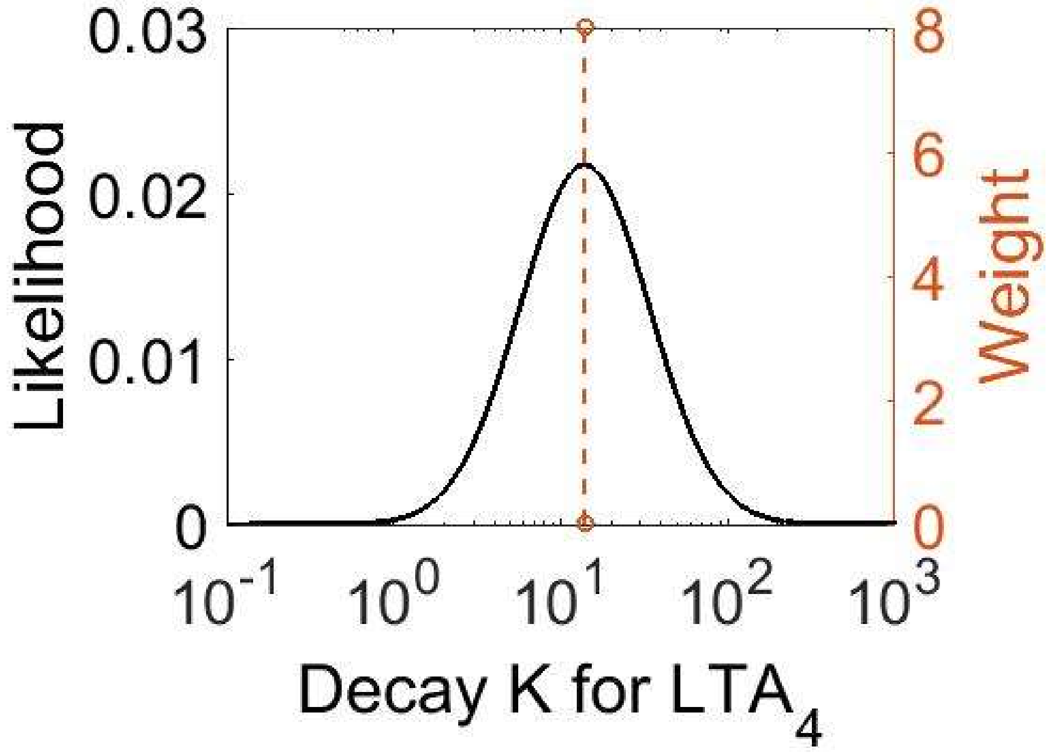


**Figure SF.9.43.1.1.1.** The estimated probability distribution for the $LTA_4$ decay constant. The value and weight of the literature values used to define the distribution are indicated by an orange dashed line. The x axis is plotted on a log-scale.

**S.9.44. Reaction 93: $LTB_4 \rightarrow \phi$**

The decay of $LTB_4$ in the intracellular compartment into metabolites outside of the scope of this model. An example of metabolites outside of the scope of this model includes metabolites which have been removed from the extracellular compartment into the systemic circulation or are degraded into metabolites not of interest to this model, the term decay reaction refers to the decrease in metabolite concentration by this collection of unspecified processes.

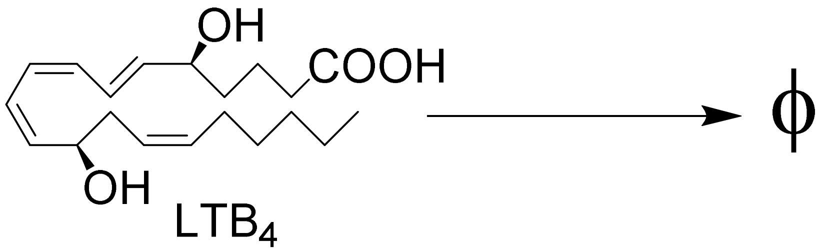


**Figure SF.9.44.** The decay of leukotriene $B_4$ ($LTB_4$) in the intracellular compartment (Reaction 93).

**SEq.9.44.** Reaction rate law for Reaction 93.

$$\boldsymbol{v_{93} = K[\mathrm{LTB_4}]}$$

### S.9.44.1. Reaction parameters

#### S.9.44.1.1. Parameter: K

Parameter values for the K of Reaction 93 were obtained from the literature and summarised in Table ST.9.44.1.1.1. The log-normal distribution properties for the K of Reaction 93 are shown in Table ST.9.44.1.1.2 and plotted in Figure SF.9.44.1.1.1.

**Table ST.9.44.1.1.1.** Literature information used to design the $LTB_4$ decay constant parameter distribution. Each value was assigned a weight using the protocol described in Section 2.6.2. The type of error was assigned as 0 if it was additive and 1 if it was multiplicative.

| Half Life (min) | Error (min) | Rate constant ($min^{-1}$) | Error ($min^{-1}$) | Experimental details | | | | | | | Weight | Type of error | Reference |
|---|---|---|---|---|---|---|---|---|---|---|---|---|---|
| | | | | Experiment type | Substrate | Species | Expression Vector | pH | Temperature (°C) | Other | | | |
| 0.47 | | 1.475 | | In vivo | $LTB_4$ | Rabbit | Plasma | 7.4 | Unknown | | 8 | | (Marleau et al., 1994) |

**Table ST.9.44.1.1.2.** The log-normal distribution properties of the $LTB_4$ decay constant distribution. These values were calculated using the functions described in Section 2.6.2.

| Mode ($min^{-1}$) | Confidence Interval | Location parameter (μ) | Scale parameter (σ) |
|---|---|---|---|
| 1.47 | 1.10 | $3.93 \times 10^{-1}$ | $9.4910^{-2}$ |

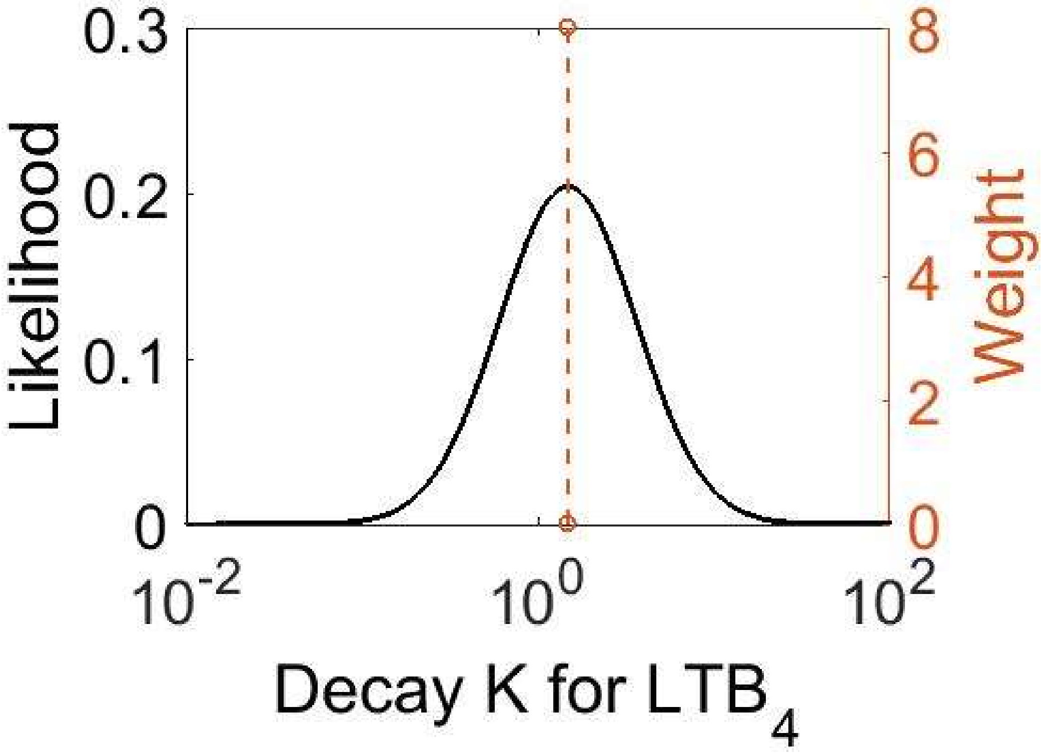


**Figure SF.9.44.1.1.1.** The estimated probability distribution for the $LTB_4$ decay constant. The value and weight of the literature values used to define the distribution are indicated by an orange dashed line. The x axis is plotted on a log-scale**.**

**S.9.45. Reaction 94: $LTC_4 \rightarrow \phi$**

The decay of $LTC_4$ in the intracellular compartment into metabolites outside of the scope of this model. An example of metabolites outside of the scope of this model includes metabolites which have been removed from the extracellular compartment into the systemic circulation or are degraded into metabolites not of interest to this model, the term decay reaction refers to the decrease in metabolite concentration by this collection of unspecified processes.

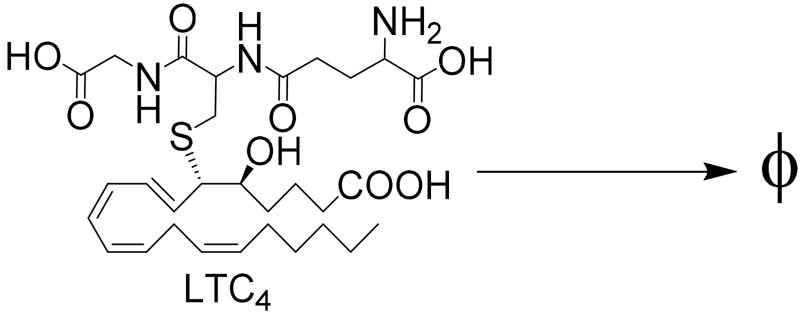


**Figure S.9.45** The decay of leukotriene $C_4$ ($LTC_4$) in the intracellular compartment (Reaction 94).

**SEq.9.45.** Reaction rate law for Reaction 94.

$$\boldsymbol{v_{94} = K[\mathrm{LTC_4}]}$$

### S.9.45.1. Reaction parameters

#### S.9.45.1.1. Parameter: K

Parameter values for the K of Reaction 94 were obtained from the literature and summarised in Table ST.9.45.1.1.1. The log-normal distribution properties for the K of Reaction 94 are shown in Table ST.9.45.1.1.2 and plotted in Figure SF.9.45.1.1.1.

**Table ST.9.45.1.1.1.** Literature information used to design the $LTC_4$ decay constant parameter distribution. Each value was assigned a weight using the protocol described in Section 2.6.2. The type of error was assigned as 0 if it was additive and 1 if it was multiplicative.

| Half Life (min) | Error (min) | Rate constant ($min^{-1}$) | Error ($min^{-1}$) | Experimental details | | | | | | | Weight | Type of error | Reference |
|---|---|---|---|---|---|---|---|---|---|---|---|---|---|
| | | | | Experiment type | Substrate | Species | Expression Vector | pH | Temperature (°C) | Other | | | |
| 0.47 | | 1.475 | | In vivo | $LTB_4$ | Rabbit | Plasma | 7.4 | Unknown | | 8 | | (Marleau et al., 1994) |

**Table ST.9.45.1.1.2.** The log-normal distribution properties of the $LTC_4$ decay constant distribution. These values were calculated using the functions described in Section 2.6.2.

| Mode ($min^{-1}$) | Confidence Interval | Location parameter (μ) | Scale parameter (σ) |
|---|---|---|---|
| 1.47 | 1.10 | $3.93 \times 10^{-1}$ | $9.49 \times 10^{-2}$ |

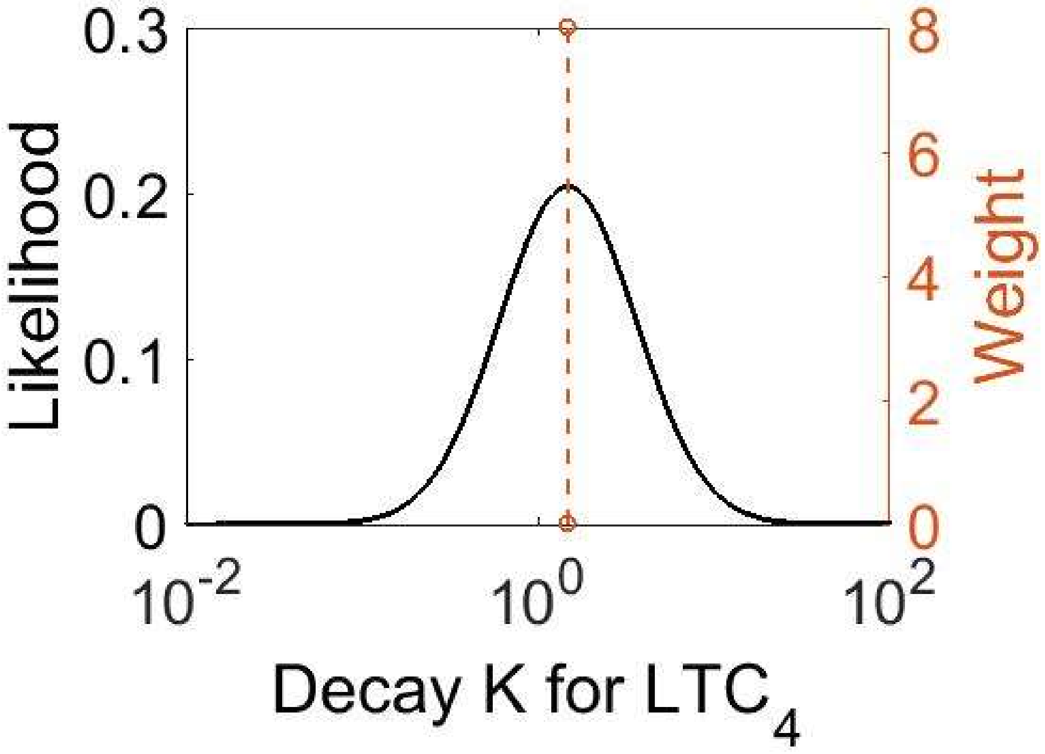


**Figure SF.9.45.1.1.1.** The estimated probability distribution for the $LTC_4$ decay constant. The value and weight of the literature values used to define the distribution are indicated by an orange dashed line. The x axis is plotted on a log-scale.

References


Bothwell, W., et al. (1982). ‘A radioimmunoassay for the unstable pulmonary metabolites of prostaglandin e1 and e2: An indirect index of their in vivo disposition and pharmacokinetics’ *J Pharmacol Exp Ther*, 220 (2), p. 229. Available at: http://jpet.aspetjournals.org/content/220/2/229.abstract.

Cawello, W., et al. (1994). ‘Metabolism and pharmacokinetics of prostaglandin e1 administered by intravenous infusion in human subjects’ *Eur J Clin Pharmacol*, 46 (3), pp. 275-7.

Dadaian, M., Granstrom, E. & Westlund, P. (1998). ‘12-hydroxyeicosatetraenoic acid is a long-lived substance in the rabbit circulation’ *Prostaglandins Other Lipid Mediat*, 55 (1), pp. 3-25.

Dickinson Zimmer, J. S., Dyckes, D. F., Bernlohr, D. A. & Murphy, R. C. (2004). ‘Fatty acid binding proteins stabilize leukotriene a4: Competition with arachidonic acid but not other lipoxygenase products’ *J Lipid Res*, 45 (11), pp. 2138-2144. DOI: 10.1194/jlr.M400240-JLR200 Available at: http://www.jlr.org/content/45/11/2138.abstract.

Fitzpatrick, F. A. & Wynalda, M. A. (1983). ‘Albumin-catalyzed metabolism of prostaglandin d2. Identification of products formed in vitro’ *J Biol Chem*, 258 (19), pp. 11713-8.

Ishihara, O., Sullivan, M. H. & Elder, M. G. (1991). ‘Differences of metabolism of prostaglandin e2 and f2 alpha by decidual stromal cells and macrophages in culture’ *Eicosanoids*, 4 (4), pp. 203-7.

Maclouf, J., de Laclos, B. F. & Borgeat, P. (1982). ‘Stimulation of leukotriene biosynthesis in human blood leukocytes by platelet-derived 12-hydroperoxy-icosatetraenoic acid’ *Proc Natl Acad Sci U S A*, 79 (19), pp. 6042-6046. DOI: 10.1073/pnas.79.19.6042 Available at: https://www.pnas.org/content/pnas/79/19/6042.full.pdf.

Marleau, S., Dallaire, N., Poubelle, P. E. & Borgeat, P. (1994). ‘Metabolic disposition of leukotriene b4 (ltb4) and oxidation-resistant analogues of ltb4 in conscious rabbits’ *Br J Pharmacol*, 112 (2), pp. 654-8.

Schuligoi, R., et al. (2007). ‘Pgd2 metabolism in plasma: Kinetics and relationship with bioactivity on dp1 and crth2 receptors’ *Biochemical Pharmacology*, 74 (1), pp. 107-117. DOI: https://doi.org/10.1016/j.bcp.2007.03.023 Available at: http://www.sciencedirect.com/science/article/pii/S0006295207001918.

Sciences, E. L. (2019). *Prostaglandin h2 datasheet* http://www.enzolifesciences.com/BML-PH002/prostaglandin-h2/ (Accessed: 26.03.19).

Suzuki, F., Hayashi, H. & Hayaishi, O. (1986). ‘Transport of prostaglandin d2 into brain’ *Brain Res*, 385 (2), pp. 321-8.

Vinge, E. (1985). ‘Arachidonic acid-induced platelet aggregation and prostanoid formation in whole blood in relation to plasma concentration of indomethacin’ *Eur J Clin Pharmacol*, 28 (2), pp. 163-9.

Wei, C., Zhu, P., Shah, S. J. & Blair, I. A. (2009). ‘15-oxo-eicosatetraenoic acid, a metabolite of macrophage 15-hydroxyprostaglandin dehydrogenase that inhibits endothelial cell proliferation’ *Mol Pharmacol*, 76 (3), pp. 516-525. DOI: 10.1124/mol.109.057489 Available at: https://www.ncbi.nlm.nih.gov/pubmed/19535459

https://www.ncbi.nlm.nih.gov/pmc/PMC2730384/.
Wild, D. (2005). *The immunoassay handbook*. Third Edition edn. Amsterdam, Netherlands: Elsevier, p. 864.
Ylikorkala, D. & Viinikka, L. (1981). 'Measurement of 6-keto-prostaglandin e1 alpha in human plasma with radioimmunoassay: Effect of prostacyclin infusion' *Prostaglandins Med*, 6 (4), pp. 427-36.

**Supplementary Document S10. Transporter-mediated Reaction Structure and Parameterisation.**

Documentation of parameter values obtained for all transporter-mediated reactions in the model (Reactions 22–42, 67, 70, 101–111; Supplementary Table S5) from the literature and associated uncertainty for the eicosanoid network model. Parameterisation was performed using the method of Tsigkinopoulou *et al.,* (2018). The table includes information regarding each reaction and its respective parameters are documented. This includes information such as the reaction rate law and the literature values that were used to define parameters, including experimental conditions, total weights and literature references from which the data were obtained. In this model some parameters are referred as "Dependent parameters", meaning that the log-normal distribution for that parameter was calculated using multivariate distributions (discussed in **Section 2.6.2**). As a result, no confidence interval factor or literature values were cited for the Dependent parameters.

**Contents**

**S.10.1. Reaction 22: $PGF_{2\alpha} \rightleftharpoons exPGF_{2\alpha}$ ....458**

**S.10.2. Reaction 23: $TXB_2 \rightleftharpoons exTXB_2$ ....467**

**S.10.3. Reaction 24: 6-keto-$PGF_{1\alpha} \rightleftharpoons$ ex6-keto-$PGF_{1\alpha}$ ....476**

**S.10.4. Reaction 25: $PGE_2 \rightleftharpoons exPGE_2$ ....485**

**S.10.5. Reaction 26: 15-deoxy-$PGJ_2 \rightleftharpoons$ ex15-deoxy-$PGJ_2$ ....494**

**S.10.6. Reaction 27: 5-oxo-ETE ⇌ ex5-oxo-ETE ....503**

**S.10.7. Reaction 28: 15-HETE⇌ ex15-HETE ....512**

**S.10.8. Reaction 29: $LTB_4 \rightleftharpoons exLTB_4$ ....521**

**S.10.9. Reaction 30: $LTC_4 \rightleftharpoons exLTC_4$ ....530**

**S.10.10. Reaction 31: 12-HETE ⇌ ex12-HETE ....539**

**S.10.11. Reaction 32: $TXA_2 \rightleftharpoons exTXA_2$ ....548**

**S.10.12. Reaction 33: $PGI_2 \rightleftharpoons exPGI_2$ ....557**

**S.10.13. Reaction 34: $PGH_2 \rightleftharpoons exPGH_2$ ....566**

**S.10.14. Reaction 35: $PGD_2 \rightleftharpoons exPGD_2$ ....574**

**S.10.15. Reaction 36: $PGJ_2 \rightleftharpoons exPGJ_2$ ....583**

**S.10.16. Reaction 37: 12-HPETE ⇌ ex12-HPETE ....592**

**S.10.17. Reaction 38: 15-HPETE ⇌ ex15-HPETE ....601**

**S.10.18. Reaction 39: 5-HPETE ⇌ ex5-HPETE ....610**

**S.10.19. Reaction 40: 5-HETE ⇌ ex5-HETE ....619**

**S.10.20. Reaction 41: $LTA_4 \rightleftharpoons exLTA_4$ ....628**

**S.10.21. Reaction 42: AA ⇌ exAA ........................................................................ 637**

**S.10.22. Reaction 67: 15-keto-$PGE_2$ ⇌ ex15-keto-$PGE_2$ ............................................... 645**

**S.10.23. Reaction 70: 13,14-dihydro-15-keto-$PGE_2$ ⇌ ex13,14-dihydro-15-keto-$PGE_2$ ......................................................................................................................... 654**

**S.10.24. Reaction 101: $PGF_{2\alpha}$ ⇌ ex$PGF_{2\alpha}$ (PGT) ........................................................... 663**

**S.10.25. Reaction 102: $PGE_2$ ⇌ ex$PGE_2$ (PGT) ............................................................ 672**

**S.10.26. Reaction 103: $PGI_2$ ⇌ ex$PGI_2$ (PGT) .............................................................. 681**

**S.10.27. Reaction 104: $PGD_2$ ⇌ ex$PGD_2$ (PGT) ............................................................ 690**

**S.10.28. Reaction 105: $PGJ_2$ ⇌ ex$PGJ_2$ (PGT) ............................................................. 699**

**S.10.29. Reaction 106: $TXB_2$ ⇌ ex$TXB_2$ (PGT) ............................................................ 708**

**S.10.30. Reaction 107: 13,14-dihydro-15-keto-$PGE_2$ ⇌ ex13,14-dihydro-15-keto-$PGE_2$ (PGT) ................................................................................................................ 717**

**S.10.31. Reaction 108: 15-keto-$PGE_2$ ⇌ ex15-keto-$PGE_2$ (PGT) ................................. 726**

**S.10.32. Reaction 109: 6-keto-$PGF_{1\alpha}$ ⇌ ex6-keto-$PGF_{1\alpha}$ (PGT) ................................... 735**

**S.10.33. Reaction 110: $TXA_2$ ⇌ ex$TXA_2$ (PGT) ............................................................ 744**

**S.10.34. Reaction 111: 15-deoxy-$PGJ_2$ ⇌ ex15-deoxy-$PGJ_2$ (PGT) ............................. 753**

**References ................................................................................................................ 762**

### S.10.1. Reaction 22: $PGF_{2\alpha} \rightleftharpoons exPGF_{2\alpha}$

$PGF_{2\alpha}$ is relocated from the intracellular compartment to the extracellular compartment via the ATP-binding cassette (ABC) transporter.

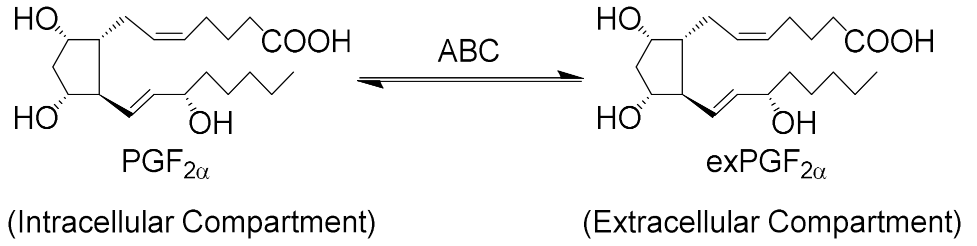


**Figure SF.10.1.** The transport of prostaglandin $F_{2\alpha}$ ($PGF_{2\alpha}$) from the intracellular compartment to the extracellular compartment by ATP Binding Cassette protein (ABC) (Reaction 22).

**SEq.10.1.** Reaction rate law for Reaction 22.

$$\boldsymbol{v_{22}} = [\boldsymbol{ABC}] \cdot \boldsymbol{k_{cat}} \cdot \frac{{}^{PGF_{2\alpha}}/_{K_m} \cdot \left( 1 - \frac{exPGF_{2\alpha}}{PGF2\alpha \cdot e^{-0.5 \cdot \left(G + R \cdot T \cdot \ln\left(\frac{ATP}{ADP}\right)\right) / R \cdot T}} \right)}{1 + \frac{PGF2\alpha}{K_m} + \frac{exPGF2\alpha}{K_m} + ABC\_CI}$$

### S.10.1.1. Reaction parameters

#### S.10.1.1.1. Parameter: ABC $k_{cat}$

Parameter values for the ABC $k_{cat}$ of Reaction 22 were obtained from the literature and summarised in Table ST.10.1.1.1.1. The log-normal distribution properties for the ABC $k_{cat}$ of Reaction 22 are shown in Table ST.10.1.1.1.2 and plotted in Figure SF.10.1.1.1.1.

**Table ST.10.1.1.1.1.** Literature information used to design the ABC $k_{cat}$ parameter distribution. Each value was assigned a weight using the protocol described in Section 2.6.2. The type of error was assigned as 0 if it was additive and 1 if it was multiplicative.

| Value ($min^{-1}$) | Error ($min^{-1}$) | Experimental details | | | | | | Weight | Type of error | Reference |
|---|---|---|---|---|---|---|---|---|---|---|
| | | Species | Expression Vector | Enzyme | pH | Temperature (°C) | Other | | | |
| 3.50 | NaN | Bacteria | E. coli. | Glucose transporter | Unknown | Unknown | Bionumbers calculation | **8** | 0 | (Waygood and Steeves, 1980) |
| 3.30 $x10^{-1}$ | NaN | Bacteria | E. coli. | Lactose transporter | Unknown | Unknown | Bionumbers calculation | **8** | 0 | (Naftalin et al., 2007) |
| 8.30 $x10^{-1}$ | NaN | Bacteria | E. coli. | Lactose transporter | Unknown | Unknown | Bionumbers calculation | **8** | 0 | (Naftalin et al., 2007) |
| 3.28 | NaN | Yeast | Saccharomyces cerevisiae | HXT7 transporter | Unknown | Unknown | Bionumbers calculation | **8** | 0 | (Ye et al., 2001) |
| 1.13 $x10^{1}$ | | Primate | Baculovirus | MRP2 | 7 | 37 | | 512 | | (Yasunaga et al., 2008) |

**Table ST.10.1.1.1.2** The log-normal distribution properties of the ABC $k_{cat}$ distribution. These values were calculated using the functions described in Section 2.6.2.

| **Mode ($min^{-1}$)** | **Confidence Interval** | **Location parameter (μ)** | **Scale parameter (σ)** |
|---|---|---|---|
| 1.13 $x10^{1}$ | 1.00 $x10^{1}$ | 3.22 | 8.91 $x10^{-1}$ |

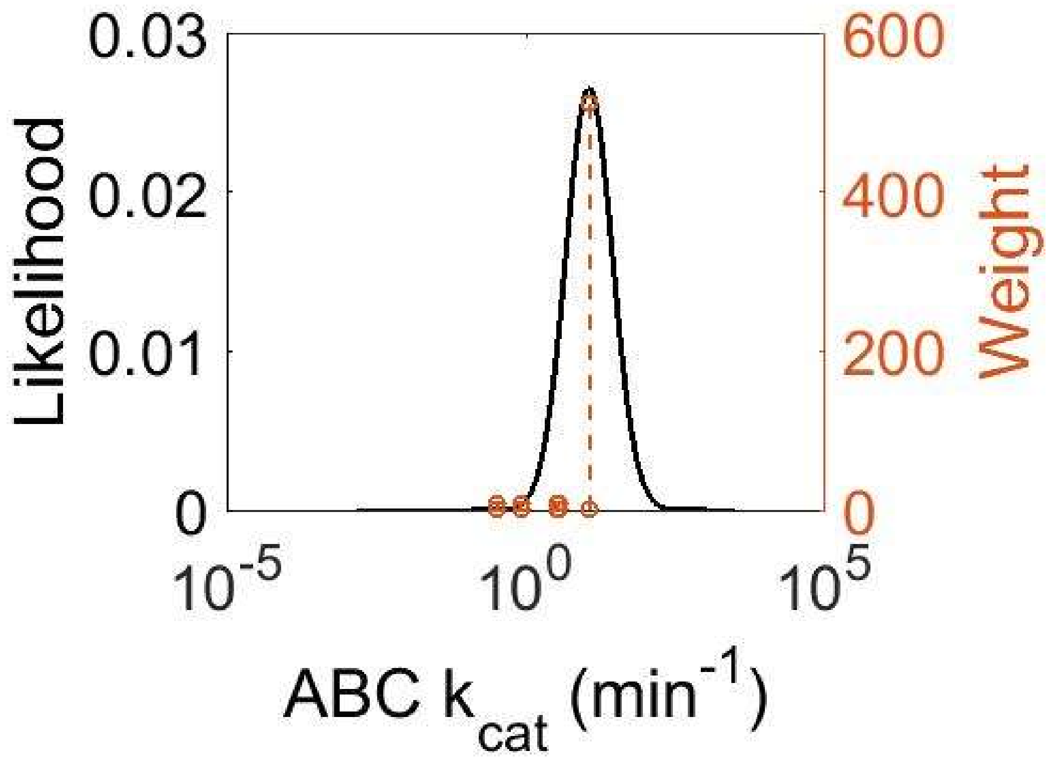


**Figure SF.10.1.1.1.1.** The estimated probability distribution for ABC $k_{cat}$. The value and weight of the literature values used to define the distribution are indicated by an orange dashed line. The x axis is plotted on a log-scale.

### S.10.1.1.2. Parameter: ABC $K_{ms}$

Parameter values for the ABC $K_{ms}$ of Reaction 22 were obtained from the literature and summarised in Table ST.10.1.1.2.1. The log-normal distribution properties for the ABC $K_{ms}$ of Reaction 22 are shown in Table ST.10.1.1.2.2 and plotted in Figure SF.10.1.1.2.1.

**Table ST.10.1.1.2.1.** Literature information used to design the ABC $K_{ms}$ parameter distribution. Each value was assigned a weight using the protocol described in Section 2.6.2. The type of error was assigned as 0 if it was additive and 1 if it was multiplicative.

| Value (mM) | Error (mM) | Experimental details | | | | | | Weight | Type of error | Reference |
|---|---|---|---|---|---|---|---|---|---|---|
| | | Species | Expression Vector | Enzyme | pH | Temperature (°C) | Other | | | |
| $1.09 \times 10^{-2}$ | $4.10 \times 10^{-3}$ | Human | Primate | MRP2 | 7 | 37 | | **512** | 0 | (Yasunaga et al., 2008) |
| $3.66 \times 10^{-5}$ | $3.80 \times 10^{-6}$ | Unknown | H69AR plasma membrane | MRP1 | 7.4 | 37 | | **128** | 0 | (Mao et al., 2000) |
| $5.30 \times 10^{-3}$ | $2.60 \times 10^{-3}$ | Human | HEK293 cells | MRP3 | 7.4 | 37 | | **128** | 0 | (Zeng et al., 2000) |
| $1.95 \times 10^{-1}$ | $6.12 \times 10^{-2}$ | Human | Primate | MRP2 | 7 | 37 | | **512** | 0 | (Yasunaga et al., 2008) |
| $1.10 \times 10^{-1}$ | $3.91 \times 10^{-2}$ | Human | Primate | MRP2 | 7 | 37 | | **512** | 0 | (Yasunaga et al., 2008) |
| $2.07 \times 10^{-2}$ | NaN | Human | HepG2 hepatoma | ABCB1 | 7.4 | 37 | | **2048** | 0 | (Fong et al., 2007) |

**Table ST.10.1.1.2.2** The log-normal distribution properties of the ABC $K_{ms}$ distribution. These values were calculated using the functions described in Section 2.6.2.

| **Mode (mM)** | **Confidence Interval** | **Location parameter (μ)** | **Scale parameter (σ)** |
|---|---|---|---|
| $2.10 \times 10^{-2}$ | $2.37 \times 10^{1}$ | -2.60 | 1.12 |

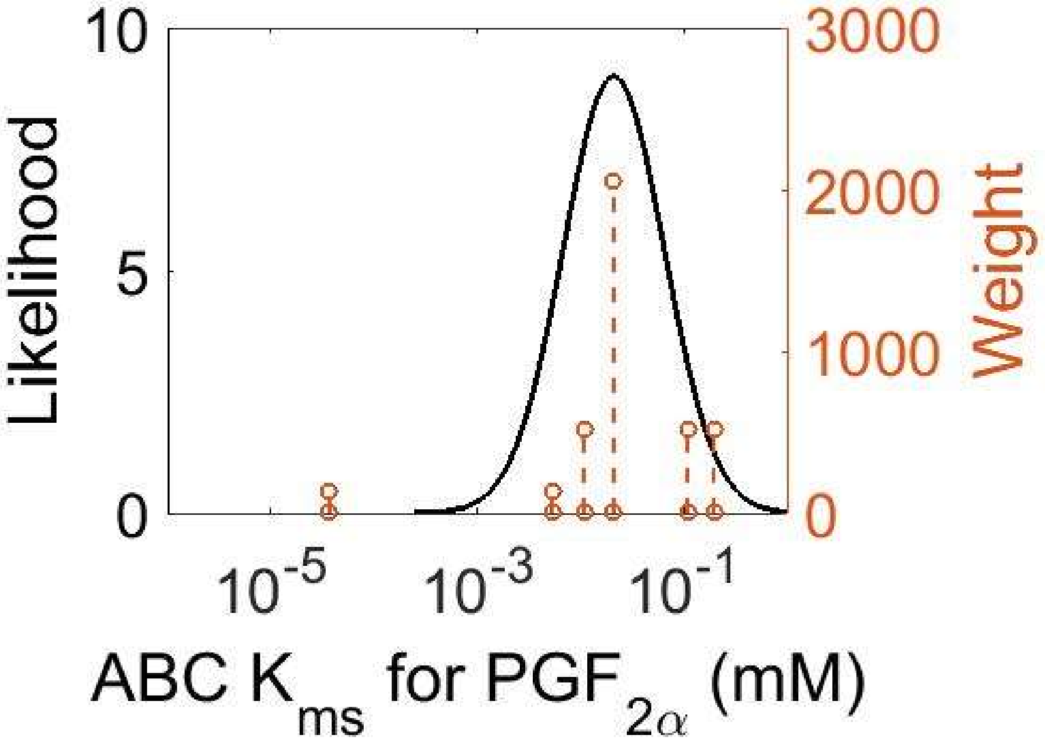


**Figure SF.10.1.1.2.1.** The estimated probability distribution for ABC $K_{ms}$. The value and weight of the literature values used to define the distribution are indicated by an orange dashed line. The x axis is plotted on a log-scale.

**S.10.1.1.3. Parameter: ABC concentration**

Parameter values for the ABC concentration of Reaction 22 were obtained from the literature and summarised in Table ST.10.1.1.3.1. The log-normal distribution properties for the ABC concentration of Reaction 22 are shown in Table ST.10.1.1.3.2 and plotted in Figure SF.10.1.1.3.1. To convert the enzyme concentration from ppm to mM, **Equation S.6.2** was used.

**Table ST.10.1.1.3.1.** Literature information used to design the ABC concentration parameter distribution. Each value was assigned a weight using the protocol described in Section 2.6.2. The type of error was assigned as 0 if it was additive and 1 if it was multiplicative.

| Value (ppm) | Error (mM) | Experimental details | | | | | | Weight | Type of error | Reference |
|---|---|---|---|---|---|---|---|---|---|---|
| | | Species | Expression Vector | Enzyme | pH | Temperature (°C) | Other | | | |
| 5.15 | NaN | Human | Stomach | ABC | 7.5 | 37 | | 2048 | 0 | (Wilhelm et al., 2014) |
| 5.94 | NaN | Human | Lung | ABC | 7.5 | 37 | | 2048 | 0 | (Kim et al., 2014) |
| 2.66 | NaN | Human | Gut | ABC | 7.5 | 37 | | 2048 | 0 | (Kim et al., 2014) |

**Table ST.10.1.1.3.2.** The log-normal distribution properties of the ABC concentration distribution. These values were calculated using the functions described in Section 2.6.2.

| Mode (ppm) | Mode (mM) | Confidence Interval | Location parameter (μ) | Scale parameter (σ) |
|---|---|---|---|---|
| 5.06 | $2.80 \times 10^{-5}$ | 1.44 | 1.74 | $3.42 \times 10^{-1}$ |

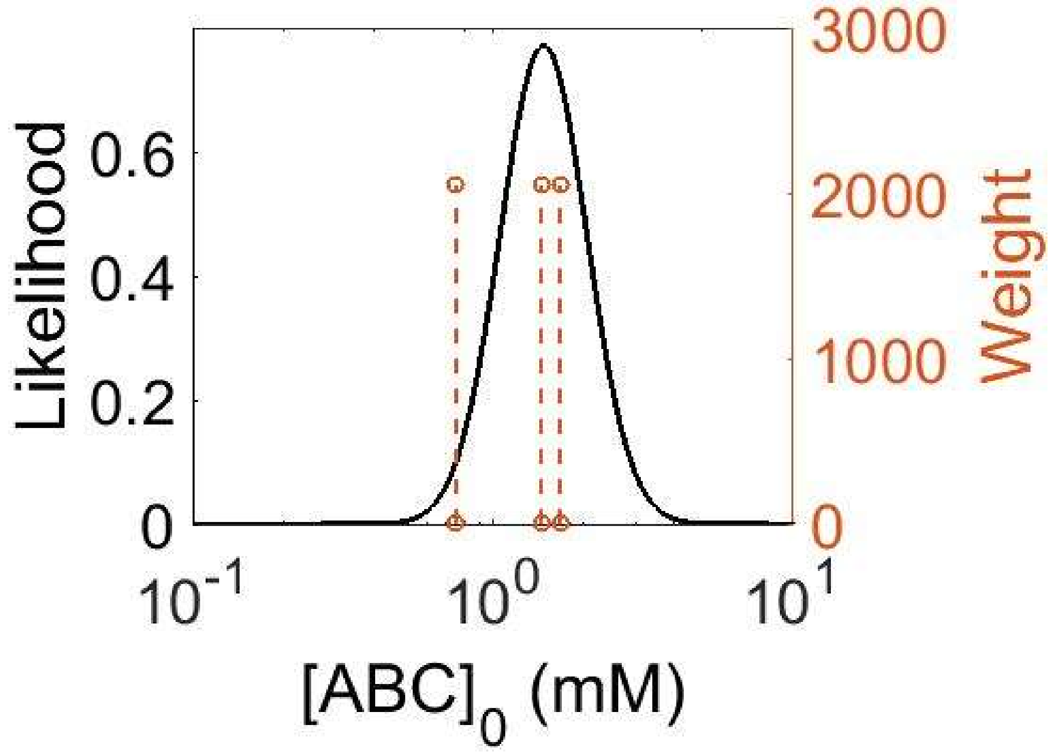


**Figure SF.10.1.1.3.1.** The estimated probability distribution for the ABC concentration. The value and weight of the literature values used to define the distribution are indicated by an orange dashed line. The x axis is plotted on a log-scale.

### S.10.2. Reaction 23: $TXB_2 \rightleftharpoons exTXB_2$

$TXB_2$ is relocated from the intracellular compartment to the extracellular compartment via the ATP-binding cassette (ABC) transporter.

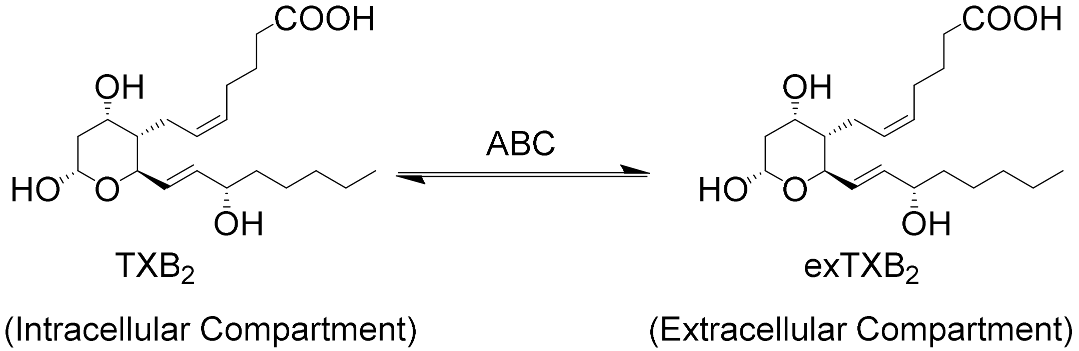


**Figure SF.10.2.** The transport of thromboxane ($TXB_2$) from the intracellular compartment to the extracellular compartment by ATP Binding Cassette protein (ABC) (Reaction 23).

**SEq.10.2.** Reaction rate law for Reaction 23.

$$v_{23} = [ABC] \cdot k_{cat} \cdot \frac{{}^{\mathrm{TXB_2}}\!/_{K_m} \cdot \left(1 - \dfrac{\mathrm{exTXB_2}}{\mathrm{TXB2} \cdot e^{-0.5 \cdot \left(G + R \cdot T \cdot \ln\left(\frac{ATP}{ADP}\right)\right) / R \cdot T}}\right)}{1 + \dfrac{\mathrm{TXB_2}}{K_m} + \dfrac{\mathrm{exTXB_2}}{K_m} + \mathrm{ABC\_CI}}$$

### S.10.2.1. Reaction parameters

### S.10.2.1.1. Parameter: ABC $k_{cat}$

Parameter values for the ABC $k_{cat}$ of Reaction 23 were obtained from the literature and summarised in Table ST.10.2.1.1.1. The log-normal distribution properties for the ABC $k_{cat}$ of Reaction 23 are shown in Table ST.10.2.1.1.2 and plotted in Figure SF.10.2.1.1.1.

**Table ST.10.2.1.1.1.** Literature information used to design the ABC $k_{cat}$ parameter distribution. Each value was assigned a weight using the protocol described in Section 2.6.2. The type of error was assigned as 0 if it was additive and 1 if it was multiplicative.

| Value ($min^{-1}$) | Error ($min^{-1}$) | Experimental details | | | | | | Weight | Type of error | Reference |
|---|---|---|---|---|---|---|---|---|---|---|
| | | Species | Expression Vector | Enzyme | pH | Temperature (°C) | Other | | | |
| 3.50 | NaN | Bacteria | E. coli. | Glucose transporter | Unknown | Unknown | Bionumbers calculation | **8** | 0 | (Waygood and Steeves, 1980) |
| 3.30 $x10^{-1}$ | NaN | Bacteria | E. coli. | Lactose transporter | Unknown | Unknown | Bionumbers calculation | **8** | 0 | (Naftalin et al., 2007) |
| 8.30 $x10^{-1}$ | NaN | Bacteria | E. coli. | Lactose transporter | Unknown | Unknown | Bionumbers calculation | **8** | 0 | (Naftalin et al., 2007) |
| 3.28 | NaN | Yeast | Saccharomyces cerevisiae | HXT7 transporter | Unknown | Unknown | Bionumbers calculation | **8** | 0 | (Ye et al., 2001) |
| 1.13 $x10^{1}$ | | Primate | Baculovirus | MRP2 | 7 | 37 | | 512 | | (Yasunaga et al., 2008) |

**Table ST.10.2.1.1.2.** The log-normal distribution properties of the ABC $k_{cat}$ distribution. These values were calculated using the functions described in Section 2.6.2.

| Mode ($min^{-1}$) | Confidence Interval | Location parameter (μ) | Scale parameter (σ) |
|---|---|---|---|
| 1.13 $x10^{1}$ | 1.00 $x10^{1}$ | 3.22 | $8.9110^{-1}$ |

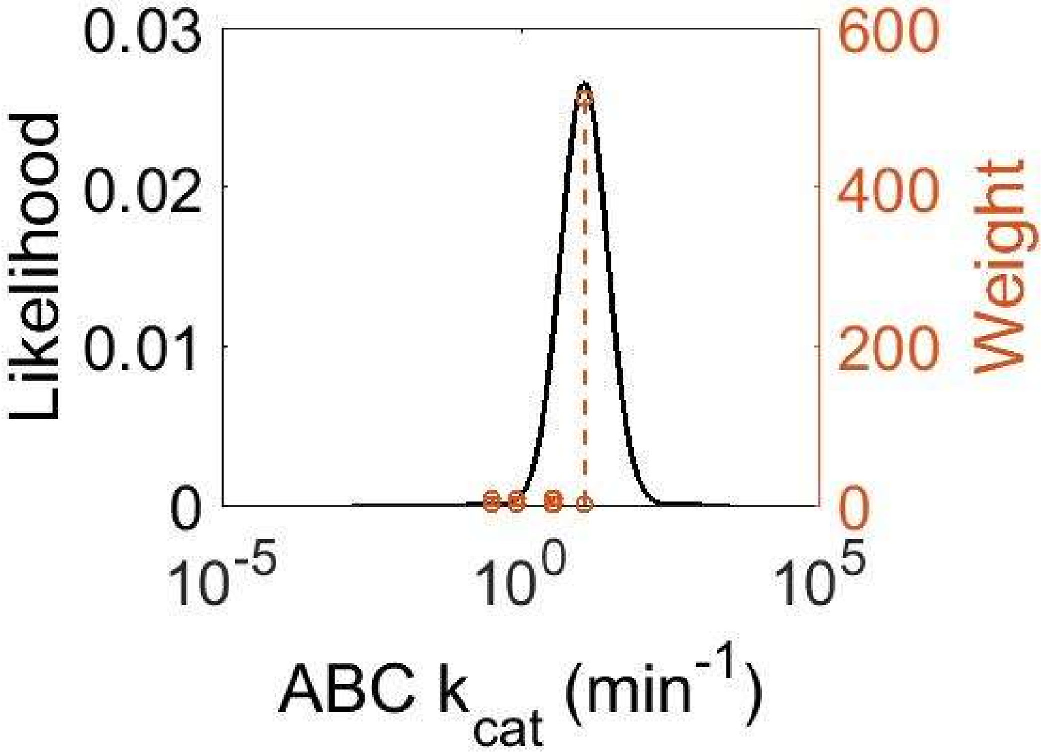


**Figure SF.10.2.1.1.1.** The estimated probability distribution for ABC $k_{cat}$. The value and weight of the literature values used to define the distribution are indicated by an orange dashed line. The x axis is plotted on a log-scale.

### S.10.2.1.2. Parameter: ABC $K_{ms}$

Parameter values for the ABC $K_{ms}$ of Reaction 23 were obtained from the literature and summarised in Table ST.10.2.1.2.1. The log-normal distribution properties for the ABC $K_{ms}$ of Reaction 23 are shown in Table ST.10.2.1.2.2 and plotted in Figure SF.10.2.1.2.1.

**Table ST.10.2.1.2.1.** Literature information used to design the ABC $K_{ms}$ parameter distribution. Each value was assigned a weight using the protocol described in Section 2.6.2. The type of error was assigned as 0 if it was additive and 1 if it was multiplicative.

| Value (mM) | Error (mM) | Experimental details | | | | | | Weight | Type of error | Reference |
|---|---|---|---|---|---|---|---|---|---|---|
| | | Species | Expression Vector | Enzyme | pH | Temperature (°C) | Other | | | |
| $1.09 \times 10^{-2}$ | $4.10 \times 10^{-3}$ | Human | Primate | MRP2 | 7 | 37 | | **512** | 0 | (Yasunaga et al., 2008) |
| $3.66 \times 10^{-5}$ | $3.80 \times 10^{-6}$ | Unknown | H69AR plasma membrane | MRP1 | 7.4 | 37 | | **128** | 0 | (Mao et al., 2000) |
| $5.30 \times 10^{-3}$ | $2.60 \times 10^{-3}$ | Human | HEK293 cells | MRP3 | 7.4 | 37 | | **128** | 0 | (Zeng et al., 2000) |
| $1.95 \times 10^{-1}$ | $6.12 \times 10^{-2}$ | Human | Primate | MRP2 | 7 | 37 | | **512** | 0 | (Yasunaga et al., 2008) |
| $1.10 \times 10^{-1}$ | $3.91 \times 10^{-2}$ | Human | Primate | MRP2 | 7 | 37 | | **512** | 0 | (Yasunaga et al., 2008) |
| $2.07 \times 10^{-2}$ | NaN | Human | HepG2 hepatoma | ABCB1 | 7.4 | 37 | | **2048** | 0 | (Fong et al., 2007) |

**Table ST.10.2.1.2.2.** The log-normal distribution properties of the ABC $K_{ms}$ distribution. These values were calculated using the functions described in Section 2.6.2.

| Mode (mM) | Confidence Interval | Location parameter (μ) | Scale parameter (σ) |
|---|---|---|---|
| $2.10 \times 10^{-2}$ | $2.37 \times 10^{1}$ | -2.60 | 1.12 |

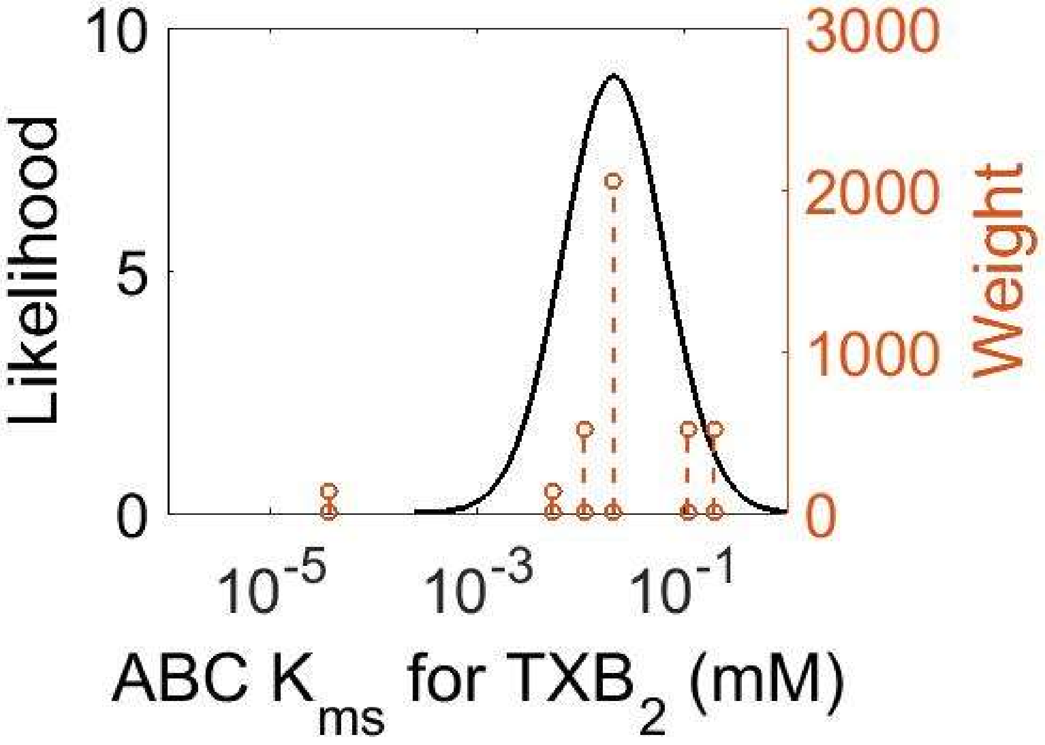


**Figure SF.10.2.1.2.1.** The estimated probability distribution for ABC $K_{ms}$. The value and weight of the literature values used to define the distribution are indicated by an orange dashed line. The x axis is plotted on a log-scale.

**S10.2.1.3. Parameter: ABC concentration**

Parameter values for the ABC concentration of Reaction 23 were obtained from the literature and summarised in Table ST.10.2.1.3.1. The log-normal distribution properties for the ABC concentration of Reaction 23 are shown in Table ST.10.2.1.3.2 and plotted in Figure SF.10.2.1.3.1. To convert the enzyme concentration from ppm to mM, **Equation S.6.2** was used.

**Table ST.10.2.1.3.1.** Literature information used to design the ABC concentration parameter distribution. Each value was assigned a weight using the protocol described in Section 2.6.2. The type of error was assigned as 0 if it was additive and 1 if it was multiplicative.

| Value (ppm) | Error (ppm) | Experimental details | | | | | | Weight | Type of error | Reference |
|---|---|---|---|---|---|---|---|---|---|---|
| | | Species | Expression Vector | Enzyme | pH | Temperature (°C) | Other | | | |
| 5.15 | NaN | Human | Stomach | ABC | 7.5 | 37 | | 2048 | 0 | (Wilhelm et al., 2014) |
| 5.94 | NaN | Human | Lung | ABC | 7.5 | 37 | | 2048 | 0 | (Kim et al., 2014) |
| 2.66 | NaN | Human | Gut | ABC | 7.5 | 37 | | 2048 | 0 | (Kim et al., 2014) |

**Table ST.10.2.1.3.2.** The log-normal distribution properties of the ABC concentration distribution. These values were calculated using the functions described in Section 2.6.2.

| Mode (ppm) | Mode (mM) | Confidence Interval | Location parameter (μ) | Scale parameter (σ) |
|---|---|---|---|---|
| 5.06 | $2.80 \times 10^{-5}$ | 1.44 | 1.74 | $3.4210^{-1}$ |

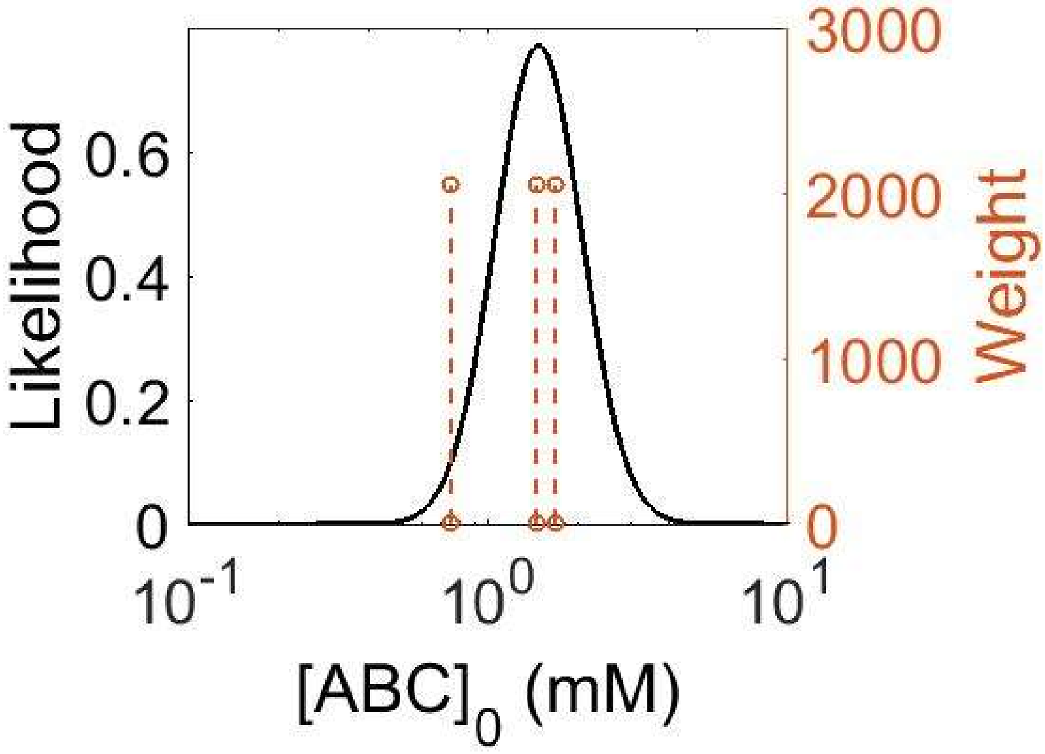


**Figure SF.10.2.1.3.1.** The estimated probability distribution for the ABC concentration. The value and weight of the literature values used to define the distribution are indicated by an orange dashed line. The x axis is plotted on a log-scale.

### S.10.3. Reaction 24: 6-keto-PGF$_{1\alpha}$ ⇌ ex6-keto-PGF$_{1\alpha}$

6-keto-PGF$_{1\alpha}$ is relocated from the intracellular compartment to the extracellular compartment via the ATP-binding cassette (ABC) transporter.

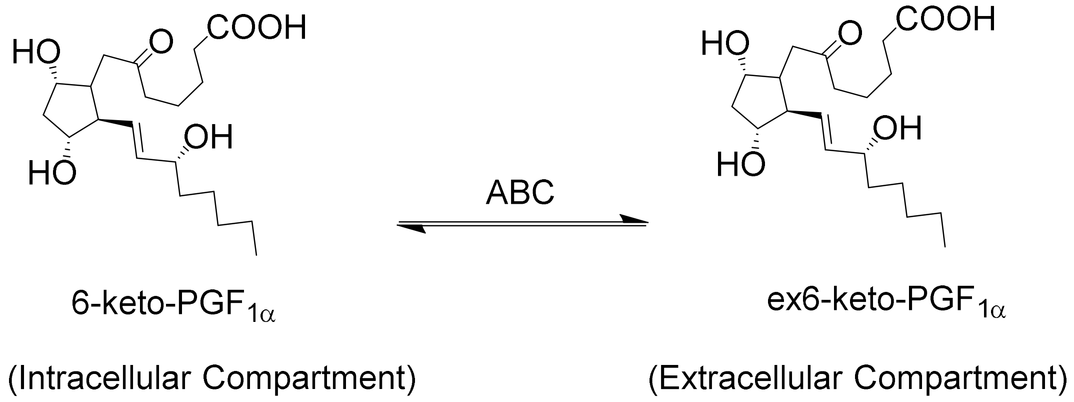


**Figure SF.10.3.** The transport of 6-keto-prostaglandin F$_{1\alpha}$ (6-keto-PGF$_{1\alpha}$) from the intracellular compartment to the extracellular compartment by ATP Binding Cassette protein (ABC) (Reaction 24).

**SEq.10.3. Reaction rate law for Reaction 24.**

$$\nu_{24} = [ABC] \cdot k_{cat} \cdot \frac{\text{6-keto-PGF}_{1\alpha}/K_m \cdot \left( 1 - \frac{\text{ex6-keto-PGF}_{1\alpha}}{\text{6-keto-PGF1}\alpha \cdot e^{-0.5 \cdot \left(G + R \cdot T \cdot \ln\left(\frac{ATP}{ADP}\right)\right) / R \cdot T}} \right)}{1 + \frac{\text{6-keto-PGF}_{1\alpha}}{K_m} + \frac{\text{ex6-keto-PGF}_{1\alpha}}{K_m} + \text{ABC\_CI}}$$

## S.10.3.1. Reaction parameters

### S.10.3.1.1. Parameter: ABC $k_{cat}$

Parameter values for the ABC $k_{cat}$ of Reaction 24 were obtained from the literature and summarised in Table ST.10.3.1.1.1. The log-normal distribution properties for the ABC $k_{cat}$ of Reaction 24 are shown in Table ST.10.3.1.1.2 and plotted in Figure SF.10.3.1.1.1.

**Table ST.10.3.1.1.1.** Literature information used to design the ABC $k_{cat}$ parameter distribution. Each value was assigned a weight using the protocol described in Section 2.6.2. The type of error was assigned as 0 if it was additive and 1 if it was multiplicative.

| Value ($min^{-1}$) | Error ($min^{-1}$) | Experimental details | | | | | | Weight | Type of error | Reference |
|---|---|---|---|---|---|---|---|---|---|---|
| | | Species | Expression Vector | Enzyme | pH | Temperature (°C) | Other | | | |
| 3.50 | NaN | Bacteria | E. coli. | Glucose transporter | Unknown | Unknown | Bionumbers calculation | **8** | 0 | (Waygood and Steeves, 1980) |
| 3.30 x$10^{-1}$ | NaN | Bacteria | E. coli. | Lactose transporter | Unknown | Unknown | Bionumbers calculation | **8** | 0 | (Naftalin et al., 2007) |
| 8.30 x$10^{-1}$ | NaN | Bacteria | E. coli. | Lactose transporter | Unknown | Unknown | Bionumbers calculation | **8** | 0 | (Naftalin et al., 2007) |
| 3.28 | NaN | Yeast | Saccharomyces cerevisiae | HXT7 transporter | Unknown | Unknown | Bionumbers calculation | **8** | 0 | (Ye et al., 2001) |
| 1.13 x$10^{1}$ | | Primate | Baculovirus | MRP2 | 7 | 37 | | 512 | | (Yasunaga et al., 2008) |

**Table ST.10.3.1.1.2.** The log-normal distribution properties of the ABC $k_{cat}$ distribution. These values were calculated using the functions described in Section 2.6.2.

| Mode ($min^{-1}$) | Confidence Interval | Location parameter (μ) | Scale parameter (σ) |
|---|---|---|---|
| 1.13 $x10^1$ | 1.00 x101 | 3.22 | $8.9110^{-1}$ |

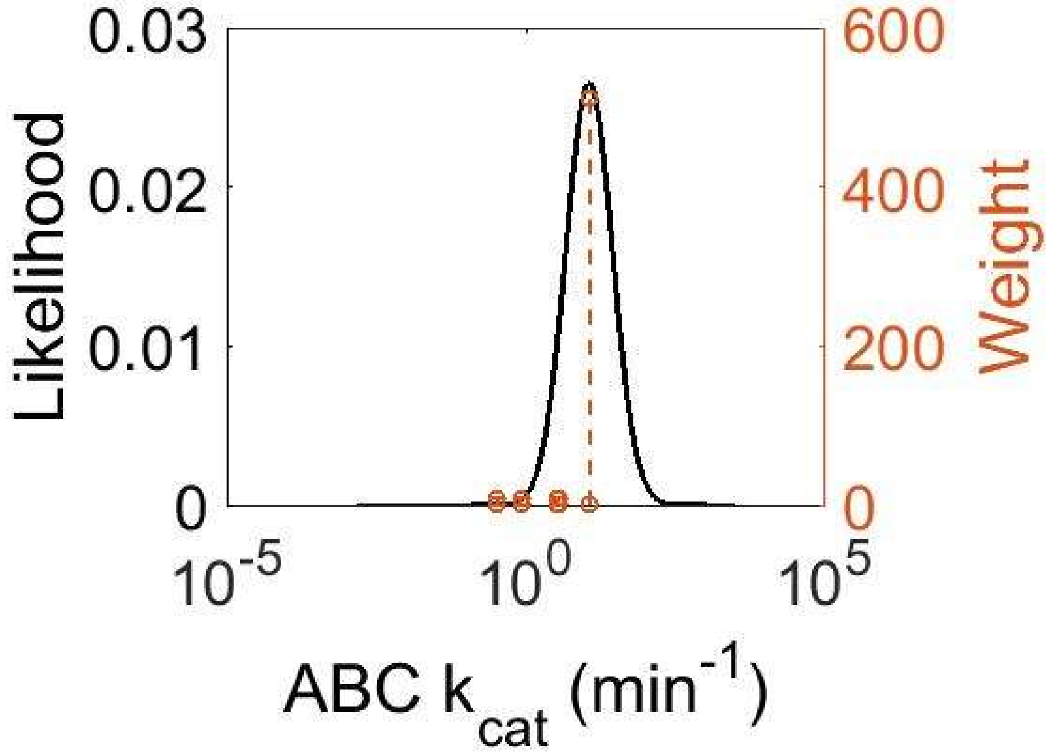


**Figure SF.10.3.1.1.1.** The estimated probability distribution for ABC $k_{cat}$. The value and weight of the literature values used to define the distribution are indicated by an orange dashed line. The x axis is plotted on a log-scale.

**S.10.3.1.2. Parameter: ABC $K_{ms}$**

Parameter values for the ABC $K_{ms}$ of Reaction 24 were obtained from the literature and summarised in Table ST.10.3.1.2.1. The log-normal distribution properties for the ABC $K_{ms}$ of Reaction 24 are shown in Table ST.10.3.1.2.2 and plotted in Figure SF.10.3.1.2.1.

**Table ST.10.3.1.2.1.** Literature information used to design the ABC $K_{ms}$ parameter distribution. Each value was assigned a weight using the protocol described in Section 2.6.2. The type of error was assigned as 0 if it was additive and 1 if it was multiplicative.

| Value (mM) | Error (mM) | Experimental details | | | | | | Weight | Type of error | Reference |
|---|---|---|---|---|---|---|---|---|---|---|
| | | Species | Expression Vector | Enzyme | pH | Temperature (°C) | Other | | | |
| $1.09 \times 10^{-2}$ | $4.10 \times 10^{-3}$ | Human | Primate | MRP2 | 7 | 37 | | **512** | 0 | (Yasunaga et al., 2008) |
| $3.66 \times 10^{-5}$ | $3.80 \times 10^{-6}$ | Unknown | H69AR plasma membrane | MRP1 | 7.4 | 37 | | **128** | 0 | (Mao et al., 2000) |
| $5.30 \times 10^{-3}$ | $2.60 \times 10^{-3}$ | Human | HEK293 cells | MRP3 | 7.4 | 37 | | **128** | 0 | (Zeng et al., 2000) |
| $1.95 \times 10^{-1}$ | $6.12 \times 10^{-2}$ | Human | Primate | MRP2 | 7 | 37 | | **512** | 0 | (Yasunaga et al., 2008) |
| $1.10 \times 10^{-1}$ | $3.91 \times 10^{-2}$ | Human | Primate | MRP2 | 7 | 37 | | **512** | 0 | (Yasunaga et al., 2008) |
| $2.07 \times 10^{-2}$ | NaN | Human | HepG2 hepatoma | ABCB1 | 7.4 | 37 | | **2048** | 0 | (Fong et al., 2007) |

**Table ST.10.3.1.2.2.** The log-normal distribution properties of the ABC $K_{ms}$ distribution. These values were calculated using the functions described in Section 2.6.2.

| Mode (mM) | Confidence Interval | Location parameter (μ) | Scale parameter (σ) |
|---|---|---|---|
| $2.10 \times 10^{-2}$ | $2.37 \times 10^{1}$ | -2.60 | 1.12 |

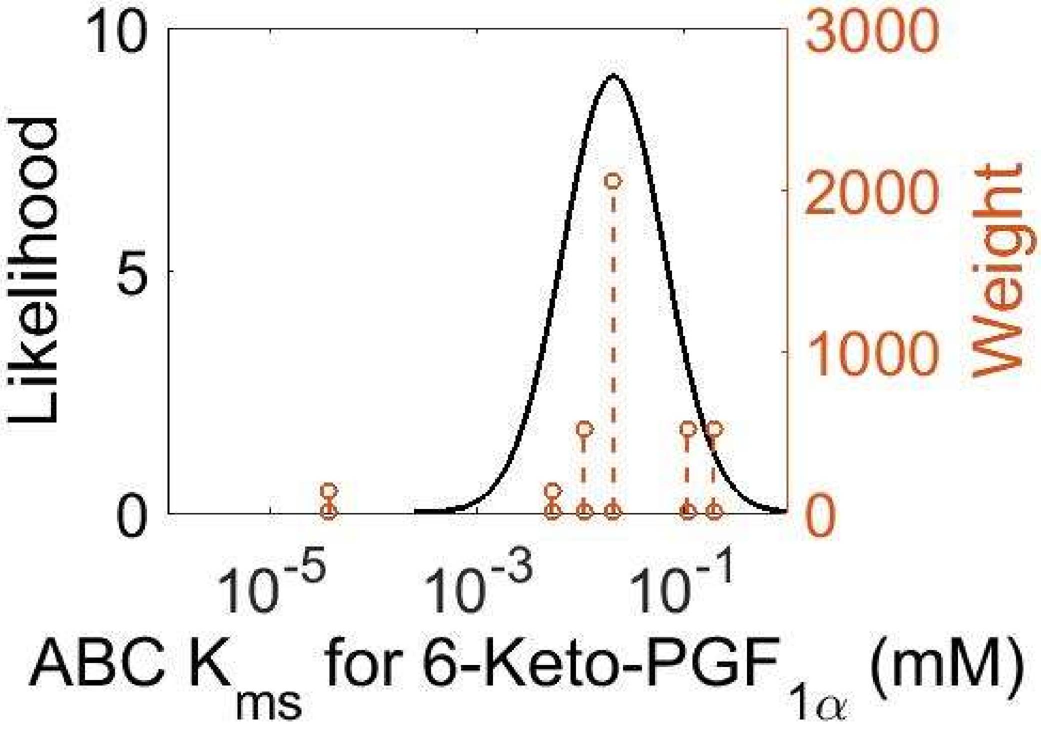


**Figure SF.10.3.1.2.1.** The estimated probability distribution for ABC $K_{ms}$. The value and weight of the literature values used to define the distribution are indicated by an orange dashed line. The x axis is plotted on a log-scale.

**S.10.3.1.3. Parameter: ABC concentration**

Parameter values for the ABC concentration of Reaction 24 were obtained from the literature and summarised in Table ST.10.3.1.3.1. The log-normal distribution properties for the ABC concentration of Reaction 24 are shown in Table ST.10.3.1.3.2 and plotted in Figure SF.10.3.1.3.1. To convert the enzyme concentration from ppm to mM, **Equation S.6.2** was used.

**Table ST.10.3.1.3.1.** Literature information used to design the ABC concentration parameter distribution. Each value was assigned a weight using the protocol described in Section 2.6.2. The type of error was assigned as 0 if it was additive and 1 if it was multiplicative.

| Value (ppm) | Error (ppm) | Experimental details | | | | | | Weight | Type of error | Reference |
|---|---|---|---|---|---|---|---|---|---|---|
| | | Species | Expression Vector | Enzyme | pH | Temperature (°C) | Other | | | |
| 5.15 | NaN | Human | Stomach | ABC | 7.5 | 37 | | 2048 | 0 | (Wilhelm et al., 2014) |
| 5.94 | NaN | Human | Lung | ABC | 7.5 | 37 | | 2048 | 0 | (Kim et al., 2014) |
| 2.66 | NaN | Human | Gut | ABC | 7.5 | 37 | | 2048 | 0 | (Kim et al., 2014) |

**Table ST.10.3.1.3.2.** The log-normal distribution properties of the ABC concentration distribution. These values were calculated using the functions described in Section 2.6.2.

| Mode (ppm) | Mode (mM) | Confidence Interval | Location parameter (μ) | Scale parameter (σ) |
|---|---|---|---|---|
| 5.06 | $2.80 \times 10^{-5}$ | 1.44 | 1.74 | $3.4210^{-1}$ |

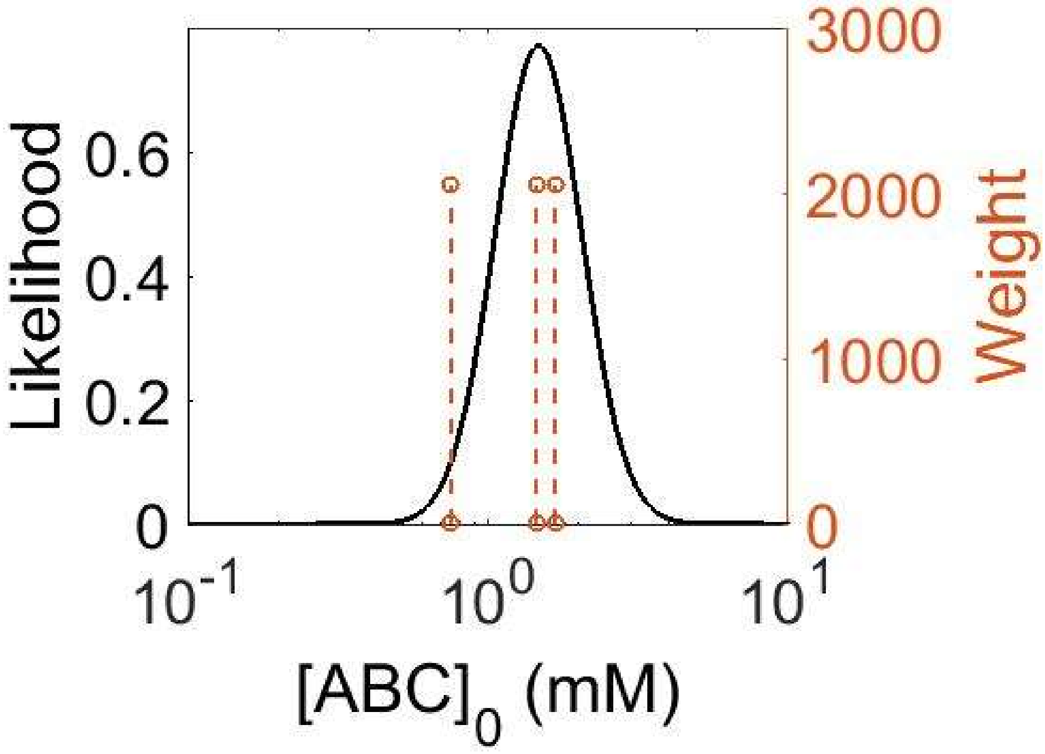


**Figure SF.10.3.1.3.1.** The estimated probability distribution for the ABC concentration. The value and weight of the literature values used to define the distribution are indicated by an orange dashed line. The x axis is plotted on a log-scale.

### S.10.4. Reaction 25: $PGE_2 \rightleftharpoons exPGE_2$

$PGE_2$ is relocated from the intracellular compartment to the extracellular compartment via the ATP-binding cassette (ABC) transporter.

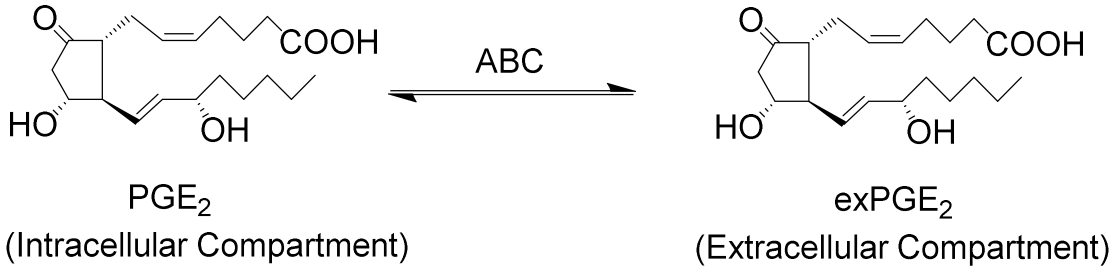


**Figure SF.10.4.** The transport of prostaglandin $E_2$ ($PGE_2$) from the intracellular compartment to the extracellular compartment by ATP Binding Cassette protein (ABC) (Reaction 25).

**SEq.10.4. Reaction rate law for Reaction 25.**

$$v_{25} = [ABC] \cdot k_{cat} \cdot \frac{\mathrm{PGE_2}/K_m \cdot \left(1 - \frac{\mathrm{exPGE_2}}{\mathrm{PGE2} \cdot e^{-0.5 \cdot \left(G + R \cdot T \cdot \ln\left(\frac{ATP}{ADP}\right)\right)/R \cdot T}}\right)}{1 + \frac{\mathrm{PGE_2}}{K_m} + \frac{\mathrm{exPGE_2}}{K_m} + \mathrm{ABC\_CI}}$$

### S.10.4.1. Reaction parameters

#### S.10.4.1.1. Parameter: ABC $k_{cat}$

Parameter values for the ABC $k_{cat}$ of Reaction 25 were obtained from the literature and summarised in Table ST.10.4.1.1.1. The log-normal distribution properties for the ABC $k_{cat}$ of Reaction 25 are shown in Table ST.10.4.1.1.2 and plotted in Figure SF.10.4.1.1.1.

**Table ST.10.4.1.1.1.** Literature information used to design the ABC $k_{cat}$ parameter distribution. Each value was assigned a weight using the protocol described in Section 2.6.2. The type of error was assigned as 0 if it was additive and 1 if it was multiplicative.

| Value ($min^{-1}$) | Error ($min^{-1}$) | Experimental details | | | | | | Weight | Type of error | Reference |
|---|---|---|---|---|---|---|---|---|---|---|
| | | Species | Expression Vector | Enzyme | pH | Temperature (°C) | Other | | | |
| 3.50 | NaN | Bacteria | E. coli. | Glucose transporter | Unknown | Unknown | Bionumbers calculation | **8** | 0 | (Waygood and Steeves, 1980) |
| $3.30 \times 10^{-1}$ | NaN | Bacteria | E. coli. | Lactose transporter | Unknown | Unknown | Bionumbers calculation | **8** | 0 | (Naftalin et al., 2007) |
| $8.30 \times 10^{-1}$ | NaN | Bacteria | E. coli. | Lactose transporter | Unknown | Unknown | Bionumbers calculation | **8** | 0 | (Naftalin et al., 2007) |
| 3.28 | NaN | Yeast | Saccharomyces cerevisiae | HXT7 transporter | Unknown | Unknown | Bionumbers calculation | **8** | 0 | (Ye et al., 2001) |
| $1.13 \times 10^{1}$ | | Primate | Baculovirus | MRP2 | 7 | 37 | | 512 | | (Yasunaga et al., 2008) |

**Table ST.10.4.1.1.2.** The log-normal distribution properties of the ABC $k_{cat}$ distribution. These values were calculated using the functions described in Section 2.6.2.

| Mode ($min^{-1}$) | Confidence Interval | Location parameter (μ) | Scale parameter (σ) |
|---|---|---|---|
| 1.13 $x10^{1}$ | 1.00 $x10^{1}$ | 3.22 | $8.9110^{-1}$ |

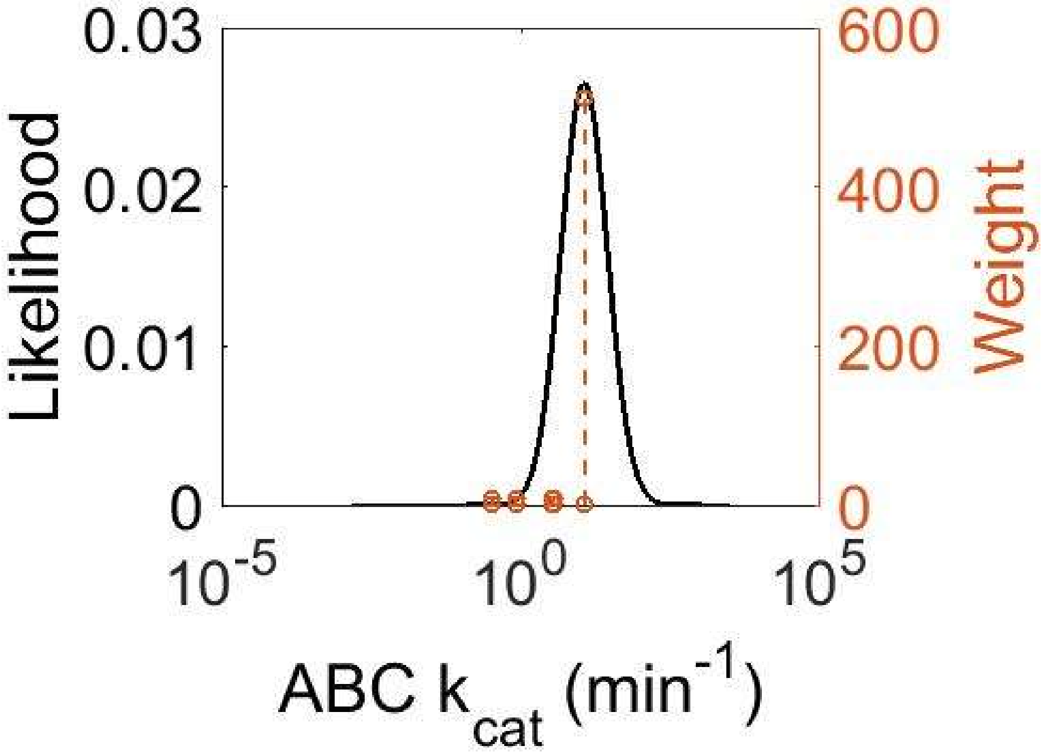


**Figure SF.10.4.1.1.1.** The estimated probability distribution for ABC $k_{cat}$. The value and weight of the literature values used to define the distribution are indicated by an orange dashed line. The x axis is plotted on a log-scale.

### S.10.4.1.2. Parameter: ABC $K_{ms}$

Parameter values for the ABC $K_{ms}$ of Reaction 25 were obtained from the literature and summarised in Table ST.10.4.1.2.1. The log-normal distribution properties for the ABC $K_{ms}$ of Reaction 25 are shown in Table ST.10.4.1.2.2 and plotted in Figure SF.10.4.1.2.1.

**Table ST.10.4.1.2.1.** Literature information used to design the ABC $K_{ms}$ parameter distribution. Each value was assigned a weight using the protocol described in Section 2.6.2. The type of error was assigned as 0 if it was additive and 1 if it was multiplicative.

| Value (mM) | Error (mM) | Experimental details | | | | | | Weight | Type of error | Reference |
|---|---|---|---|---|---|---|---|---|---|---|
| | | Species | Expression Vector | Enzyme | pH | Temperature (°C) | Other | | | |
| $1.09 \times 10^{-2}$ | $4.10 \times 10^{-3}$ | Human | Primate | MRP2 | 7 | 37 | | **512** | 0 | (Yasunaga et al., 2008) |
| $3.66 \times 10^{-5}$ | $3.80 \times 10^{-6}$ | Unknown | H69AR plasma membrane | MRP1 | 7.4 | 37 | | **128** | 0 | (Mao et al., 2000) |
| $5.30 \times 10^{-3}$ | $2.60 \times 10^{-3}$ | Human | HEK293 cells | MRP3 | 7.4 | 37 | | **128** | 0 | (Zeng et al., 2000) |
| $1.95 \times 10^{-1}$ | $6.12 \times 10^{-2}$ | Human | Primate | MRP2 | 7 | 37 | | **512** | 0 | (Yasunaga et al., 2008) |
| $1.10 \times 10^{-1}$ | $3.91 \times 10^{-2}$ | Human | Primate | MRP2 | 7 | 37 | | **512** | 0 | (Yasunaga et al., 2008) |
| $2.07 \times 10^{-2}$ | NaN | Human | HepG2 hepatoma | ABCB1 | 7.4 | 37 | | **2048** | 0 | (Fong et al., 2007) |

**Table ST.10.4.1.2.2.** The log-normal distribution properties of the ABC $K_{ms}$ distribution. These values were calculated using the functions described in Section 2.6.2.

| Mode (mM) | Confidence Interval | Location parameter (μ) | Scale parameter (σ) |
|---|---|---|---|
| 2.10 $x10^{-2}$ | 2.37 $x10^{1}$ | -2.60 | 1.12 |

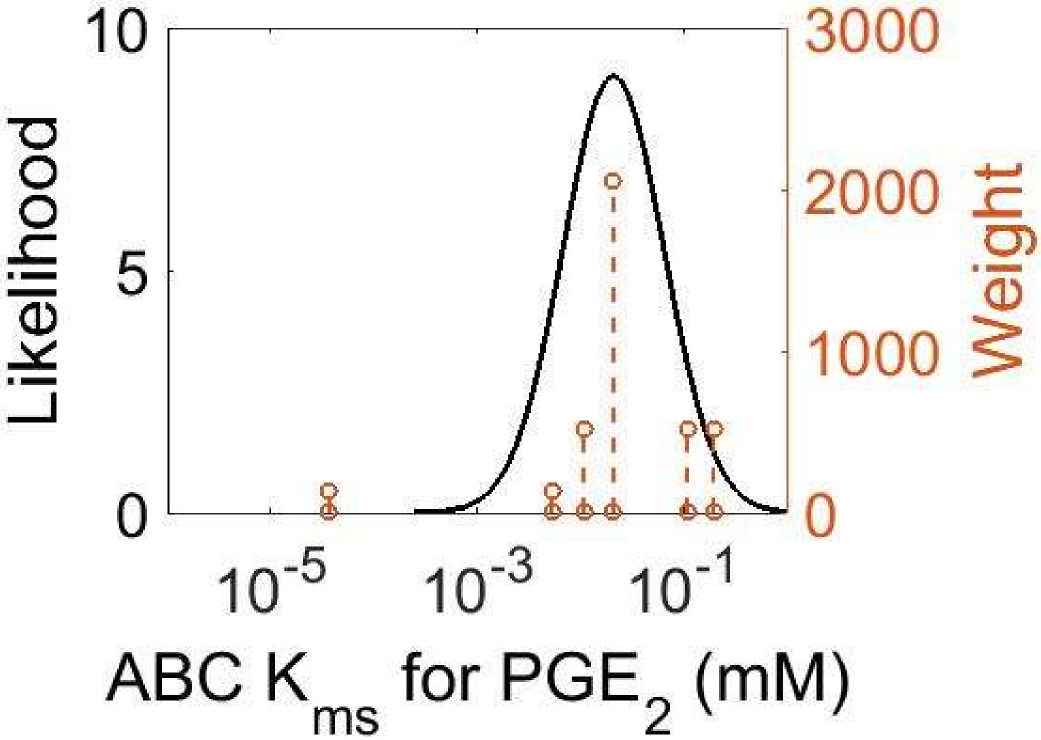


**Figure SF.10.4.1.2.1.** The estimated probability distribution for ABC $K_{ms}$. The value and weight of the literature values used to define the distribution are indicated by an orange dashed line. The x axis is plotted on a log-scale.

**S.10.4.1.3. Parameter: ABC concentration**

Parameter values for the ABC concentration of Reaction 25 were obtained from the literature and summarised in Table ST.10.4.1.3.1. The log-normal distribution properties for the ABC concentration of Reaction 25 are shown in Table ST.10.4.1.3.2 and plotted in Figure SF.10.4.1.3.1. To convert the enzyme concentration from ppm to mM, **Equation S.6.2** was used.

**Table ST.10.4.1.3.1.** Literature information used to design the ABC concentration parameter distribution. Each value was assigned a weight using the protocol described in Section 2.6.2. The type of error was assigned as 0 if it was additive and 1 if it was multiplicative.

| Value (ppm) | Error (ppm) | Experimental details | | | | | | Weight | Type of error | Reference |
|---|---|---|---|---|---|---|---|---|---|---|
| | | Species | Expression Vector | Enzyme | pH | Temperature (°C) | Other | | | |
| 5.15 | NaN | Human | Stomach | ABC | 7.5 | 37 | | 2048 | 0 | (Wilhelm et al., 2014) |
| 5.94 | NaN | Human | Lung | ABC | 7.5 | 37 | | 2048 | 0 | (Kim et al., 2014) |
| 2.66 | NaN | Human | Gut | ABC | 7.5 | 37 | | 2048 | 0 | (Kim et al., 2014) |

**Table ST.10.4.1.3.2.** The log-normal distribution properties of the ABC concentration distribution. These values were calculated using the functions described in Section 2.6.2.

| Mode (ppm) | Mode (mM) | Confidence Interval | Location parameter (μ) | Scale parameter (σ) |
|---|---|---|---|---|
| 5.06 | $2.80 \times 10^{-5}$ | 1.44 | 1.74 | $3.42 \times 10^{-1}$ |

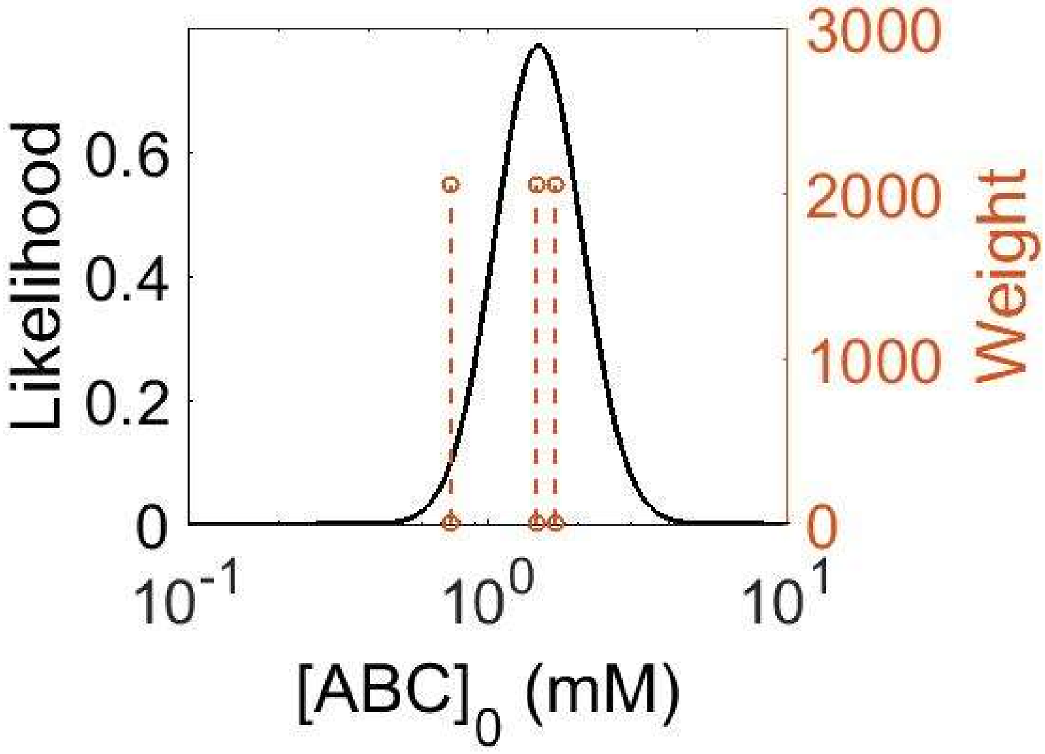


**Figure SF.10.4.1.3.1.** The estimated probability distribution for the ABC concentration. The value and weight of the literature values used to define the distribution are indicated by an orange dashed line. The x axis is plotted on a log-scale.

**S.10.5. Reaction 26: 15-deoxy-$PGJ_2$ ⇌ ex15-deoxy-$PGJ_2$**

15-deoxy-$PGJ_2$ is relocated from the intracellular compartment to the extracellular compartment via the ATP-binding cassette (ABC) transporter.

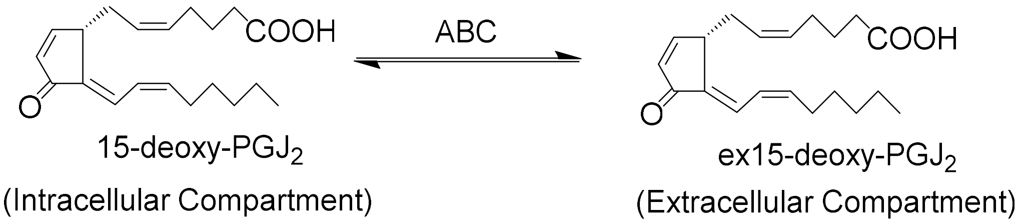


**Figure SF.10.5.** The transport of 15-deoxy-prostaglandin $J_2$ (15-deoxy-$PGJ_2$) from the intracellular compartment to the extracellular compartment by ATP Binding Cassette protein (ABC) (Reaction 26).

**SEq.10.5. Reaction rate law for Reaction 26.**

$$\boldsymbol{v_{26} = [ABC] \cdot k_{cat} \cdot \frac{{15-deoxy-PGJ_2}/{K_m} \cdot \left(1 - \frac{ex15-deoxy-PGJ_2}{15-deoxy-PGJ_2 \cdot e^{{-0.5\cdot\left(G+R\cdot T\cdot ln\left(\frac{ATP}{ADP}\right)\right)}/{R\cdot T}}}\right)}{1 + \frac{15-deoxy-PGJ_2}{K_m} + \frac{ex15-deoxy-PGJ_2}{K_m} + ABC\_CI}}$$

### S.10.5.1. Reaction parameters

#### S.10.5.1.1. Parameter: ABC $k_{cat}$

Parameter values for the ABC $k_{cat}$ of Reaction 26 were obtained from the literature and summarised in Table ST.10.5.1.1.1. The log-normal distribution properties for the ABC $k_{cat}$ of Reaction 26 are shown in Table ST.10.5.1.1.2 and plotted in Figure SF.10.5.1.1.1.

**Table ST.10.5.1.1.1.** Literature information used to design the ABC $k_{cat}$ parameter distribution. Each value was assigned a weight using the protocol described in Section 2.6.2. The type of error was assigned as 0 if it was additive and 1 if it was multiplicative.

| Value ($min^{-1}$) | Error ($min^{-1}$) | Experimental details | | | | | | Weight | Type of error | Reference |
|---|---|---|---|---|---|---|---|---|---|---|
| | | Species | Expression Vector | Enzyme | pH | Temperature (°C) | Other | | | |
| 3.50 | NaN | Bacteria | E. coli. | Glucose transporter | Unknown | Unknown | Bionumbers calculation | **8** | 0 | (Waygood and Steeves, 1980) |
| $3.30 \times 10^{-1}$ | NaN | Bacteria | E. coli. | Lactose transporter | Unknown | Unknown | Bionumbers calculation | **8** | 0 | (Naftalin et al., 2007) |
| $8.30 \times 10^{-1}$ | NaN | Bacteria | E. coli. | Lactose transporter | Unknown | Unknown | Bionumbers calculation | **8** | 0 | (Naftalin et al., 2007) |
| 3.28 | NaN | Yeast | Saccharomyces cerevisiae | HXT7 transporter | Unknown | Unknown | Bionumbers calculation | **8** | 0 | (Ye et al., 2001) |
| $1.13 \times 10^{1}$ | | Primate | Baculovirus | MRP2 | 7 | 37 | | 512 | | (Yasunaga et al., 2008) |

**Table ST.10.5.1.1.2.** The log-normal distribution properties of the ABC $k_{cat}$ distribution. These values were calculated using the functions described in Section 2.6.2.

| **Mode ($min^{-1}$)** | **Confidence Interval** | **Location parameter (μ)** | **Scale parameter (σ)** |
|---|---|---|---|
| 1.13 $x10^{1}$ | 1.00 $x10^{1}$ | 3.22 | $8.9110^{-1}$ |

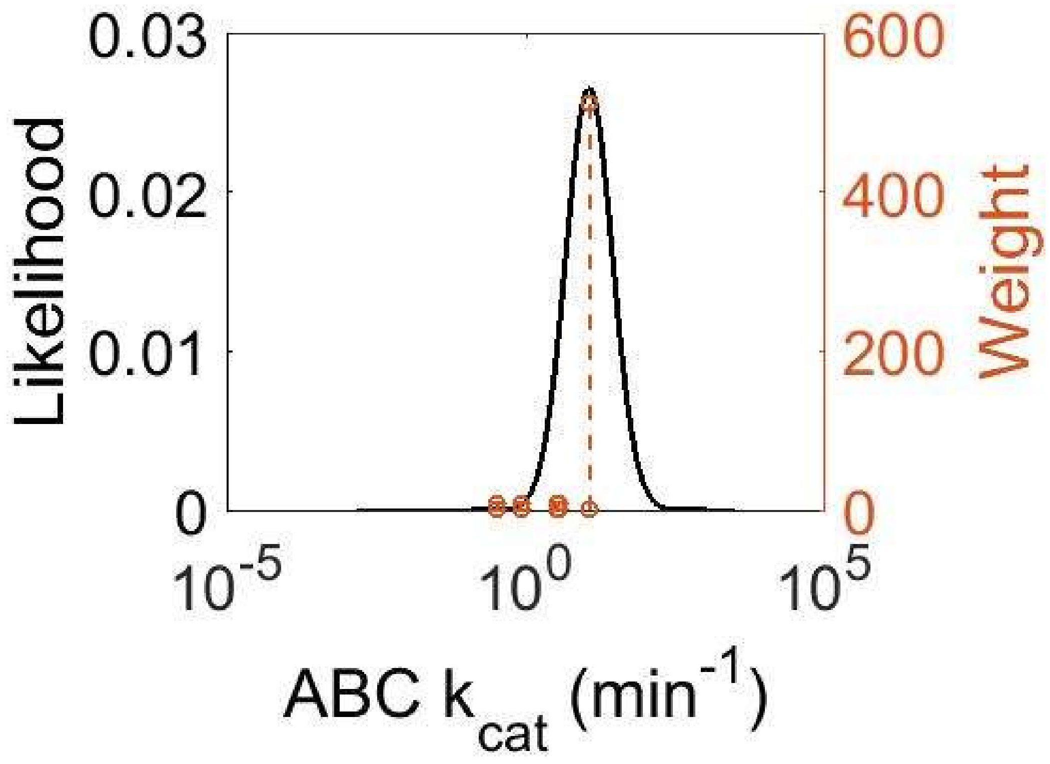


**Figure SF.10.5.1.1.1.** The estimated probability distribution for ABC $k_{cat}$. The value and weight of the literature values used to define the distribution are indicated by an orange dashed line. The x axis is plotted on a log-scale.

### S.10.5.1.2. Parameter: ABC $K_{ms}$

Parameter values for the ABC $K_{ms}$ of Reaction 26 were obtained from the literature and summarised in Table ST.10.5.1.2.1. The log-normal distribution properties for the ABC $K_{ms}$ of Reaction 26 are shown in Table ST.10.5.1.2.2 and plotted in Figure SF.10.5.1.2.1.

**Table ST.10.5.1.2.1.** Literature information used to design the ABC $K_{ms}$ parameter distribution. Each value was assigned a weight using the protocol described in Section 2.6.2. The type of error was assigned as 0 if it was additive and 1 if it was multiplicative.

| Value (mM) | Error (mM) | Experimental details | | | | | | Weight | Type of error | Reference |
|---|---|---|---|---|---|---|---|---|---|---|
| | | Species | Expression Vector | Enzyme | pH | Temperature (°C) | Other | | | |
| $1.09 \times 10^{-2}$ | $4.10 \times 10^{-3}$ | Human | Primate | MRP2 | 7 | 37 | | **512** | 0 | (Yasunaga et al., 2008) |
| $3.66 \times 10^{-5}$ | $3.80 \times 10^{-6}$ | Unknown | H69AR plasma membrane | MRP1 | 7.4 | 37 | | **128** | 0 | (Mao et al., 2000) |
| $5.30 \times 10^{-3}$ | $2.60 \times 10^{-3}$ | Human | HEK293 cells | MRP3 | 7.4 | 37 | | **128** | 0 | (Zeng et al., 2000) |
| $1.95 \times 10^{-1}$ | $6.12 \times 10^{-2}$ | Human | Primate | MRP2 | 7 | 37 | | **512** | 0 | (Yasunaga et al., 2008) |
| $1.10 \times 10^{-1}$ | $3.91 \times 10^{-2}$ | Human | Primate | MRP2 | 7 | 37 | | **512** | 0 | (Yasunaga et al., 2008) |
| $2.07 \times 10^{-2}$ | NaN | Human | HepG2 hepatoma | ABCB1 | 7.4 | 37 | | **2048** | 0 | (Fong et al., 2007) |

**Table ST.10.5.1.2.2.** The log-normal distribution properties of the ABC $K_{ms}$ distribution. These values were calculated using the functions described in Section 2.6.2.

| Mode (mM) | Confidence Interval | Location parameter (μ) | Scale parameter (σ) |
|---|---|---|---|
| $2.10 \times 10^{-2}$ | $2.37 \times 10^{1}$ | -2.60 | 1.12 |

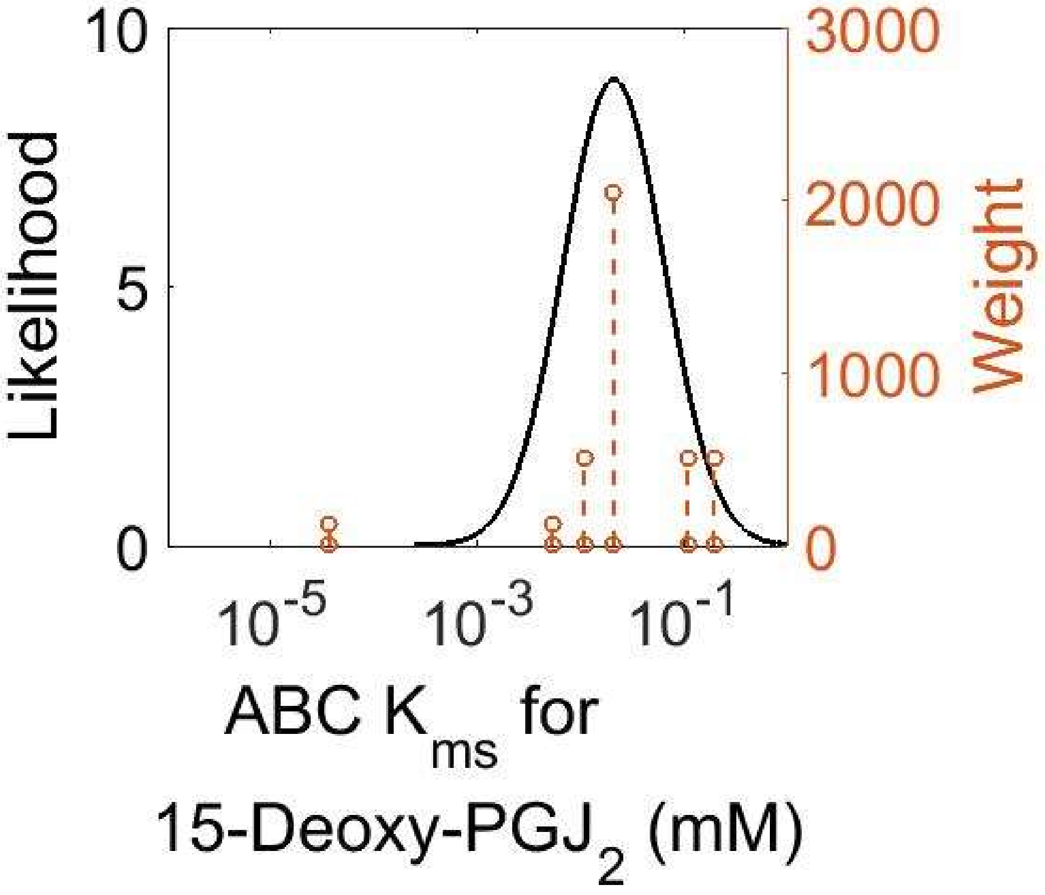


**Figure SF.10.5.1.2.1.** The estimated probability distribution for ABC $K_{ms}$. The value and weight of the literature values used to define the distribution are indicated by an orange dashed line. The x axis is plotted on a log-scale.

**S.10.5.1.3. Parameter: ABC concentration**

Parameter values for the ABC concentration of Reaction 26 were obtained from the literature and summarised in Table ST.10.5.1.3.1. The log-normal distribution properties for the ABC concentration of Reaction 26 are shown in Table ST.10.5.1.3.2 and plotted in Figure SF.10.5.1.3.1. To convert the enzyme concentration from ppm to mM, **Equation S.6.2** was used.

**Table ST.10.5.1.3.1.** Literature information used to design the ABC concentration parameter distribution. Each value was assigned a weight using the protocol described in Section 2.6.2. The type of error was assigned as 0 if it was additive and 1 if it was multiplicative.

| Value (ppm) | Error (ppm) | Experimental details | | | | | | Weight | Type of error | Reference |
|---|---|---|---|---|---|---|---|---|---|---|
| | | Species | Expression Vector | Enzyme | pH | Temperature (°C) | Other | | | |
| 5.15 | NaN | Human | Stomach | ABC | 7.5 | 37 | | 2048 | 0 | (Wilhelm et al., 2014) |
| 5.94 | NaN | Human | Lung | ABC | 7.5 | 37 | | 2048 | 0 | (Kim et al., 2014) |
| 2.66 | NaN | Human | Gut | ABC | 7.5 | 37 | | 2048 | 0 | (Kim et al., 2014) |

**Table ST.10.5.1.3.2.** The log-normal distribution properties of the ABC concentration distribution. These values were calculated using the functions described in Section 2.6.2.

| Mode (ppm) | Mode (mM) | Confidence Interval | Location parameter (μ) | Scale parameter (σ) |
|---|---|---|---|---|
| 5.06 | $2.80 \times 10^{-5}$ | 1.44 | 1.74 | $3.42 \times 10^{-1}$ |

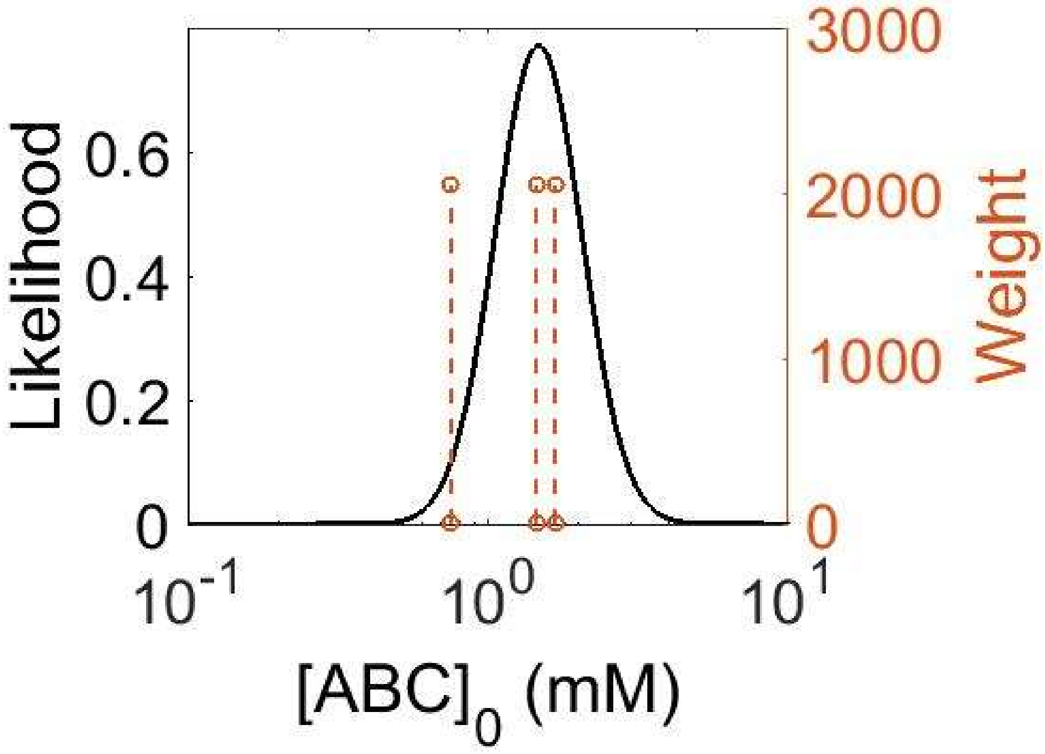


**Figure SF.10.5.1.3.1.** The estimated probability distribution for the ABC concentration. The value and weight of the literature values used to define the distribution are indicated by an orange dashed line. The x axis is plotted on a log-scale.

**S.10.6. Reaction 27: 5-oxo-ETE ⇌ ex5-oxo-ETE**

5-oxo-ETE is relocated from the intracellular compartment to the extracellular compartment via the ATP-binding cassette (ABC) transporter.

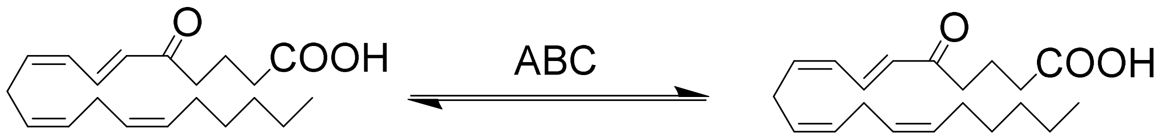


**Figure SF.10.6.** The transport of 5-oxo-eicosatetraenoic acid (5-oxo-ETE) from the intracellular compartment to the extracellular compartment by ATP Binding Cassette protein (ABC) (Reaction 27).

**SEq.10.6. Reaction rate law for Reaction 27.**

$$\boldsymbol{v_{27}} = [\boldsymbol{ABC}] \cdot \boldsymbol{k_{cat}} \cdot \frac{\text{5−oxo−ETE}/K_m \cdot \left(1 - \frac{\text{ex5−oxo−ETE}}{\text{5−oxo−ETE} \cdot e^{-0.5 \cdot \left(G + R \cdot T \cdot \ln\left(\frac{ATP}{ADP}\right)\right)/R \cdot T}}\right)}{1 + \frac{\text{5−oxo−ETE}}{K_m} + \frac{\text{ex5−oxo−ETE}}{K_m} + \text{ABC\_CI}}$$

### S.10.6.1. Reaction parameters

#### S.10.6.1.1. Parameter: ABC $k_{cat}$

Parameter values for the ABC $k_{cat}$ of Reaction 27 were obtained from the literature and summarised in Table ST.10.6.1.1.1. The log-normal distribution properties for the ABC $k_{cat}$ of Reaction 27 are shown in Table ST.10.6.1.1.2 and plotted in Figure SF.10.6.1.1.1.

**Table ST.10.6.1.1.1.** Literature information used to design the ABC $k_{cat}$ parameter distribution. Each value was assigned a weight using the protocol described in Section 2.6.2. The type of error was assigned as 0 if it was additive and 1 if it was multiplicative.

| Value ($min^{-1}$) | Error ($min^{-1}$) | Experimental details | | | | | | Weight | Type of error | Reference |
|---|---|---|---|---|---|---|---|---|---|---|
| | | Species | Expression Vector | Enzyme | pH | Temperature (°C) | Other | | | |
| 3.50 | NaN | Bacteria | E. coli. | Glucose transporter | Unknown | Unknown | Bionumbers calculation | 8 | 0 | (Waygood and Steeves, 1980) |
| $3.30 \times 10^{-1}$ | NaN | Bacteria | E. coli. | Lactose transporter | Unknown | Unknown | Bionumbers calculation | 8 | 0 | (Naftalin et al., 2007) |
| $8.30 \times 10^{-1}$ | NaN | Bacteria | E. coli. | Lactose transporter | Unknown | Unknown | Bionumbers calculation | 8 | 0 | (Naftalin et al., 2007) |
| 3.28 | NaN | Yeast | Saccharomyces cerevisiae | HXT7 transporter | Unknown | Unknown | Bionumbers calculation | 8 | 0 | (Ye et al., 2001) |
| $1.13 \times 10^{1}$ | | Primate | Baculovirus | MRP2 | 7 | 37 | | 512 | | (Yasunaga et al., 2008) |

**Table ST.10.6.1.1.2.** The log-normal distribution properties of the ABC $k_{cat}$ distribution. These values were calculated using the functions described in Section 2.6.2.

| Mode ($min^{-1}$) | Confidence Interval | Location parameter (μ) | Scale parameter (σ) |
|---|---|---|---|
| 1.13 $x10^{1}$ | 1.00 $x10^{1}$ | 3.22 | $8.9110^{-1}$ |

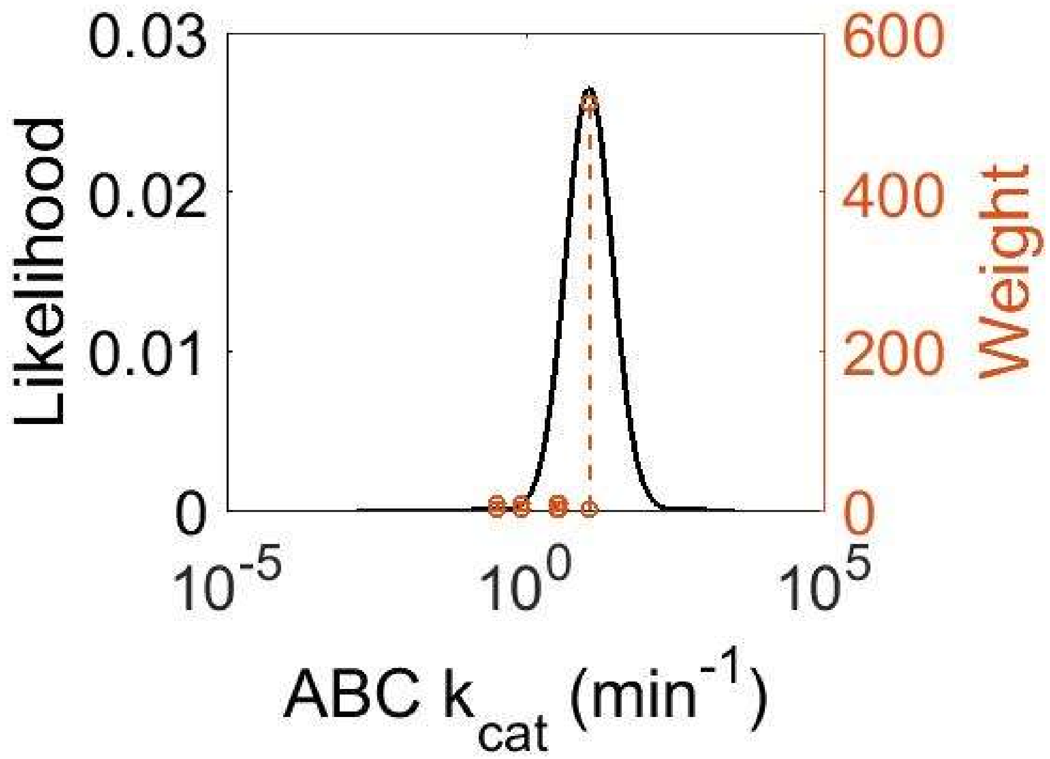


**Figure SF.10.6.1.1.1.** The estimated probability distribution for ABC $k_{cat}$. The value and weight of the literature values used to define the distribution are indicated by an orange dashed line. The x axis is plotted on a log-scale.

**S.10.6.1.2. Parameter: ABC $K_{ms}$**

Parameter values for the ABC $K_{ms}$ of Reaction 27 were obtained from the literature and summarised in Table ST.10.6.1.2.1. The log-normal distribution properties for the ABC $K_{ms}$ of Reaction 27 are shown in Table ST.10.6.1.2.2 and plotted in Figure SF.10.6.1.2.1.

**Table ST.10.6.1.2.1.** Literature information used to design the ABC $K_{ms}$ parameter distribution. Each value was assigned a weight using the protocol described in Section 2.6.2. The type of error was assigned as 0 if it was additive and 1 if it was multiplicative.

| Value (mM) | Error (mM) | Experimental details | | | | | | Weight | Type of error | Reference |
|---|---|---|---|---|---|---|---|---|---|---|
| | | Species | Expression Vector | Enzyme | pH | Temperature (°C) | Other | | | |
| $1.09 \times 10^{-2}$ | $4.10 \times 10^{-3}$ | Human | Primate | MRP2 | 7 | 37 | | **512** | 0 | (Yasunaga et al., 2008) |
| $3.66 \times 10^{-5}$ | $3.80 \times 10^{-6}$ | Unknown | H69AR plasma membrane | MRP1 | 7.4 | 37 | | **128** | 0 | (Mao et al., 2000) |
| $5.30 \times 10^{-3}$ | $2.60 \times 10^{-3}$ | Human | HEK293 cells | MRP3 | 7.4 | 37 | | **128** | 0 | (Zeng et al., 2000) |
| $1.95 \times 10^{-1}$ | $6.12 \times 10^{-2}$ | Human | Primate | MRP2 | 7 | 37 | | **512** | 0 | (Yasunaga et al., 2008) |
| $1.10 \times 10^{-1}$ | $3.91 \times 10^{-2}$ | Human | Primate | MRP2 | 7 | 37 | | **512** | 0 | (Yasunaga et al., 2008) |
| $2.07 \times 10^{-2}$ | NaN | Human | HepG2 hepatoma | ABCB1 | 7.4 | 37 | | **2048** | 0 | (Fong et al., 2007) |

**Table ST.10.6.1.2.2.** The log-normal distribution properties of the ABC $K_{ms}$ distribution. These values were calculated using the functions described in Section 2.6.2.

| **Mode (mM)** | **Confidence Interval** | **Location parameter (μ)** | **Scale parameter (σ)** |
|---|---|---|---|
| $2.10 \times 10^{-2}$ | $2.37 \times 10^{1}$ | -2.60 | 1.12 |

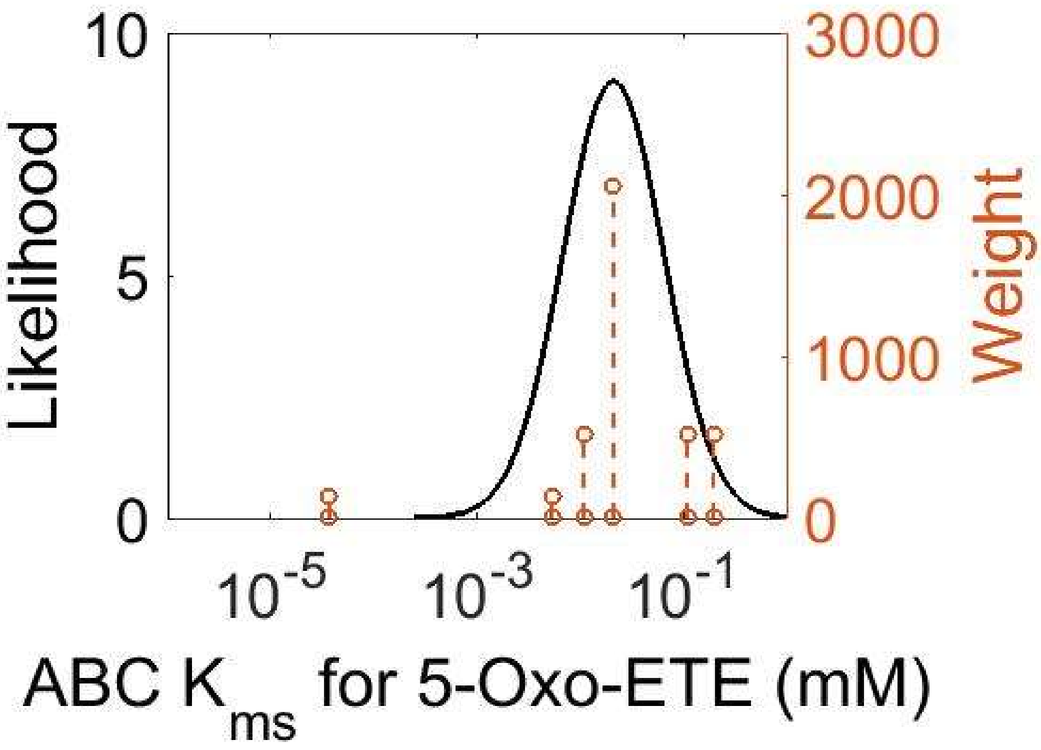


**Figure SF.10.6.1.2.1.** The estimated probability distribution for ABC $K_{ms}$. The value and weight of the literature values used to define the distribution are indicated by an orange dashed line. The x axis is plotted on a log-scale.

**S.10.6.1.3. Parameter: ABC concentration**

Parameter values for the ABC concentration of Reaction 27 were obtained from the literature and summarised in Table ST.10.6.1.3.1. The log-normal distribution properties for the ABC concentration of Reaction 27 are shown in Table ST.10.6.1.3.2 and plotted in Figure SF.10.6.1.3.1. To convert the enzyme concentration from ppm to mM, **Equation S.6.2** was used.

**Table ST.10.6.1.3.1.** Literature information used to design the ABC concentration parameter distribution. Each value was assigned a weight using the protocol described in Section 2.6.2. The type of error was assigned as 0 if it was additive and 1 if it was multiplicative.

| Value (ppm) | Error (ppm) | Experimental details | | | | | | Weight | Type of error | Reference |
|---|---|---|---|---|---|---|---|---|---|---|
| | | Species | Expression Vector | Enzyme | pH | Temperature (°C) | Other | | | |
| 5.15 | NaN | Human | Stomach | ABC | 7.5 | 37 | | 2048 | 0 | (Wilhelm et al., 2014) |
| 5.94 | NaN | Human | Lung | ABC | 7.5 | 37 | | 2048 | 0 | (Kim et al., 2014) |
| 2.66 | NaN | Human | Gut | ABC | 7.5 | 37 | | 2048 | 0 | (Kim et al., 2014) |

**Table ST.10.6.1.3.2.** The log-normal distribution properties of the ABC concentration distribution. These values were calculated using the functions described in Section 2.6.2.

| Mode (ppm) | Mode (mM) | Confidence Interval | Location parameter (μ) | Scale parameter (σ) |
|---|---|---|---|---|
| 5.06 | $2.80 \times 10^{-5}$ | 1.44 | 1.74 | $3.42 \times 10^{-1}$ |

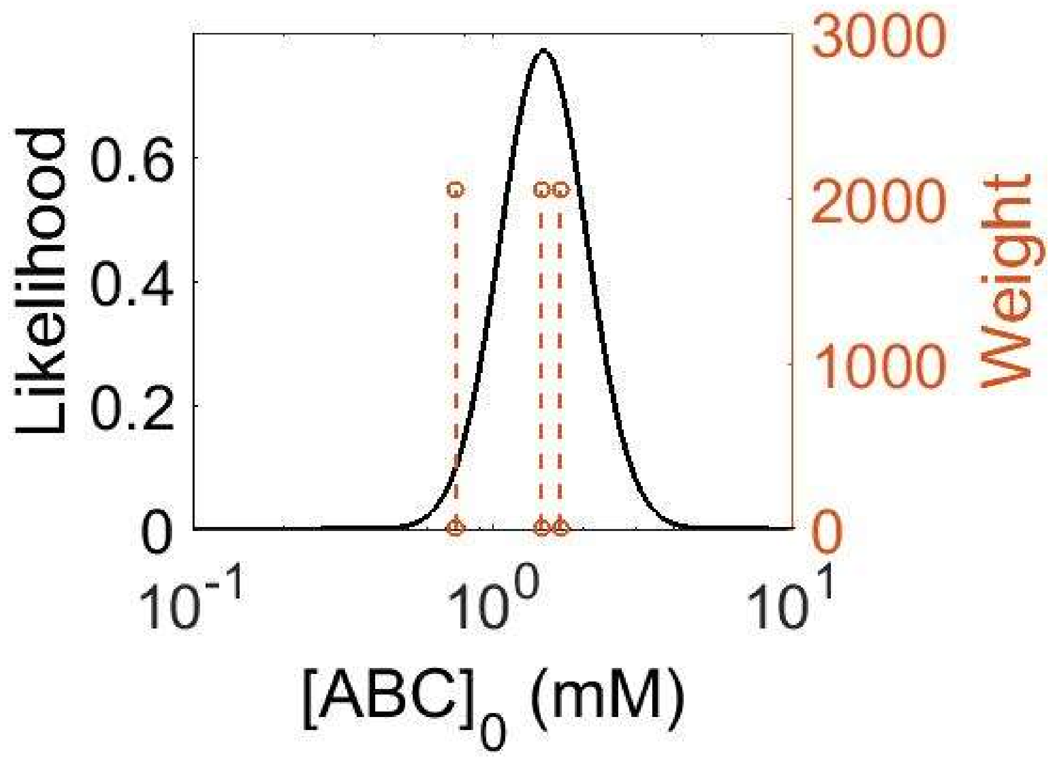


**Figure SF.10.6.1.3.1.** The estimated probability distribution for the ABC concentration. The value and weight of the literature values used to define the distribution are indicated by an orange dashed line. The x axis is plotted on a log-scale.

### S.10.7. Reaction 28: 15-HETE⇌ ex15-HETE

15-HETE is relocated from the intracellular compartment to the extracellular compartment via the ATP-binding cassette (ABC) transporter.

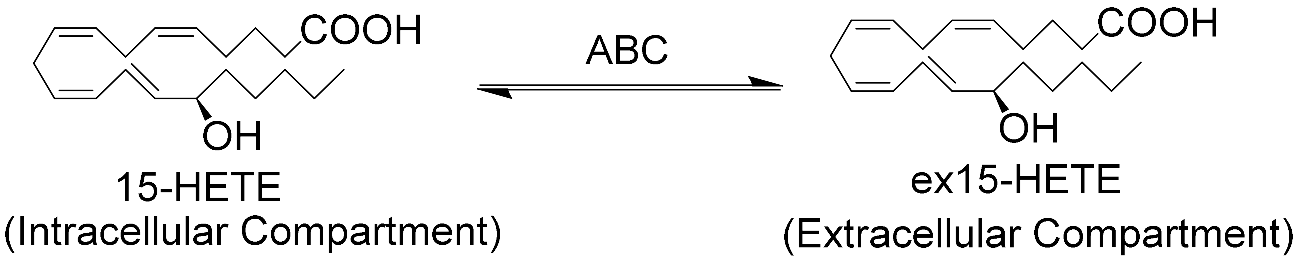


**Figure SF.10.7.** The transport of 15-hydroxyeicosatetraenoic acid (15-HETE) from the intracellular compartment to the extracellular compartment by ATP Binding Cassette protein (ABC) (Reaction 28).

**SEq.10.7.** Reaction rate law for Reaction 28.

$$v_{28} = [ABC] \cdot k_{cat} \cdot \frac{\mathbf{15-HETE}/K_m \cdot \left(1 - \frac{\mathbf{ex15-HETE}}{\mathbf{15-HETE} \cdot e^{-0.5\cdot\left(G+R\cdot T\cdot\ln\left(\frac{ATP}{ADP}\right)\right)/R\cdot T}}\right)}{1 + \frac{\mathbf{15-HETE}}{K_m} + \frac{\mathbf{ex15-HETE}}{K_m} + \mathbf{ABC\_CI}}$$

### S.10.7.1. Reaction parameters

### S.10.7.1.1. Parameter: ABC $k_{cat}$

Parameter values for the ABC $k_{cat}$ of Reaction 28 were obtained from the literature and summarised in Table ST.10.7.1.1.1. The log-normal distribution properties for the ABC $k_{cat}$ of Reaction 28 are shown in Table ST.10.7.1.1.2 and plotted in Figure SF.10.7.1.1.1.

**Table ST.10.7.1.1.1.** Literature information used to design the ABC $k_{cat}$ parameter distribution. Each value was assigned a weight using the protocol described in Section 2.6.2. The type of error was assigned as 0 if it was additive and 1 if it was multiplicative.

| Value ($min^{-1}$) | Error ($min^{-1}$) | Experimental details | | | | | | Weight | Type of error | Reference |
|---|---|---|---|---|---|---|---|---|---|---|
| | | Species | Expression Vector | Enzyme | pH | Temperature (°C) | Other | | | |
| 3.50 | NaN | Bacteria | E. coli. | Glucose transporter | Unknown | Unknown | Bionumbers calculation | **8** | 0 | (Waygood and Steeves, 1980) |
| 3.30 x$10^{-1}$ | NaN | Bacteria | E. coli. | Lactose transporter | Unknown | Unknown | Bionumbers calculation | **8** | 0 | (Naftalin et al., 2007) |
| 8.30 x$10^{-1}$ | NaN | Bacteria | E. coli. | Lactose transporter | Unknown | Unknown | Bionumbers calculation | **8** | 0 | (Naftalin et al., 2007) |
| 3.28 | NaN | Yeast | Saccharomyces cerevisiae | HXT7 transporter | Unknown | Unknown | Bionumbers calculation | **8** | 0 | (Ye et al., 2001) |
| 1.13 x$10^{1}$ | | Primate | Baculovirus | MRP2 | 7 | 37 | | 512 | | (Yasunaga et al., 2008) |

**Table ST.10.7.1.1.2.** The log-normal distribution properties of the ABC $k_{cat}$ distribution. These values were calculated using the functions described in Section 2.6.2.

| **Mode ($min^{-1}$)** | **Confidence Interval** | **Location parameter (μ)** | **Scale parameter (σ)** |
|---|---|---|---|
| 1.13 $x10^{1}$ | 1.00 $x10^{1}$ | 3.22 | $8.9110^{-1}$ |

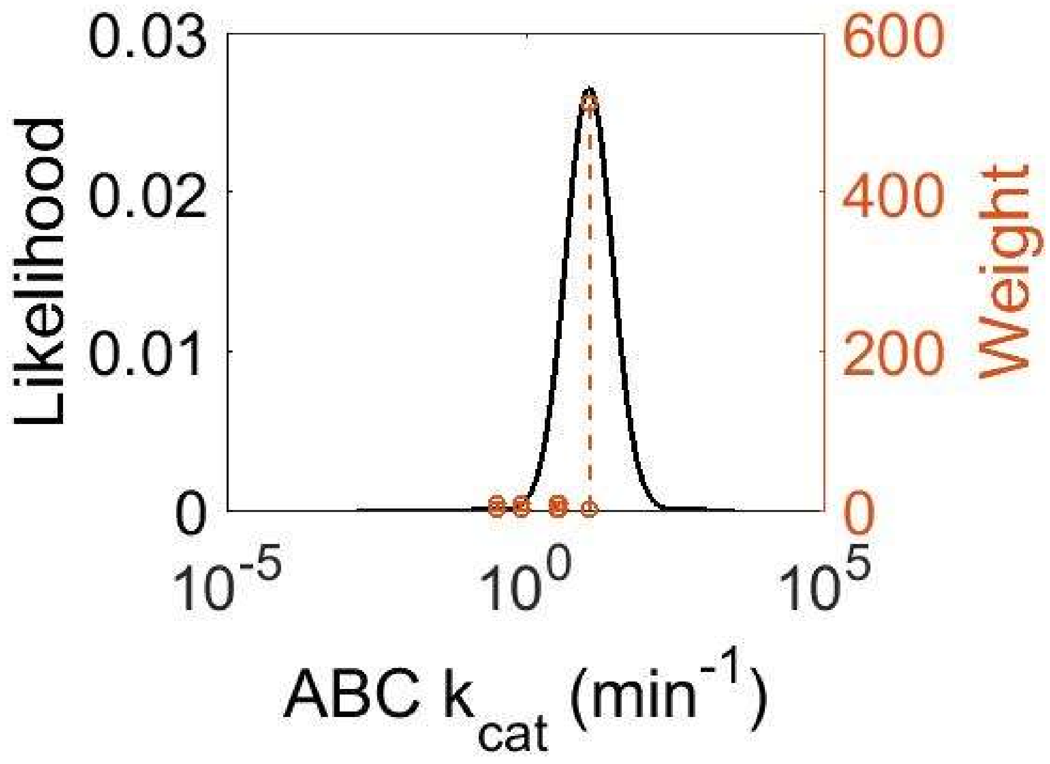


**Figure SF.10.7.1.1.1.** The estimated probability distribution for ABC $k_{cat}$. The value and weight of the literature values used to define the distribution are indicated by an orange dashed line. The x axis is plotted on a log-scale.

### S.10.7.1.2. Parameter: ABC $K_{ms}$

Parameter values for the ABC $K_{ms}$ of Reaction 28 were obtained from the literature and summarised in Table ST.10.7.1.2.1. The log-normal distribution properties for the ABC $K_{ms}$ of Reaction 28 are shown in Table ST.10.7.1.2.2 and plotted in Figure SF.10.7.1.2.1.

**Table ST.10.7.1.2.1.** Literature information used to design the ABC $K_{ms}$ parameter distribution. Each value was assigned a weight using the protocol described in Section 2.6.2. The type of error was assigned as 0 if it was additive and 1 if it was multiplicative.

| Value (mM) | Error (mM) | Experimental details | | | | | | Weight | Type of error | Reference |
|---|---|---|---|---|---|---|---|---|---|---|
| | | Species | Expression Vector | Enzyme | pH | Temperature (°C) | Other | | | |
| $1.09 \times 10^{-2}$ | $4.10 \times 10^{-3}$ | Human | Primate | MRP2 | 7 | 37 | | 512 | 0 | (Yasunaga et al., 2008) |
| $3.66 \times 10^{-5}$ | $3.80 \times 10^{-6}$ | Unknown | H69AR plasma membrane | MRP1 | 7.4 | 37 | | 128 | 0 | (Mao et al., 2000) |
| $5.30 \times 10^{-3}$ | $2.60 \times 10^{-3}$ | Human | HEK293 cells | MRP3 | 7.4 | 37 | | 128 | 0 | (Zeng et al., 2000) |
| $1.95 \times 10^{-1}$ | $6.12 \times 10^{-2}$ | Human | Primate | MRP2 | 7 | 37 | | 512 | 0 | (Yasunaga et al., 2008) |
| $1.10 \times 10^{-1}$ | $3.91 \times 10^{-2}$ | Human | Primate | MRP2 | 7 | 37 | | 512 | 0 | (Yasunaga et al., 2008) |
| $2.07 \times 10^{-2}$ | NaN | Human | HepG2 hepatoma | ABCB1 | 7.4 | 37 | | 2048 | 0 | (Fong et al., 2007) |

**Table ST.10.7.1.2.2.** The log-normal distribution properties of the ABC $K_{ms}$ distribution. These values were calculated using the functions described in Section 2.6.2.

| Mode (mM) | Confidence Interval | Location parameter (μ) | Scale parameter (σ) |
|---|---|---|---|
| $2.10 \times 10^{-2}$ | $2.37 \times 10^{1}$ | -2.60 | 1.12 |

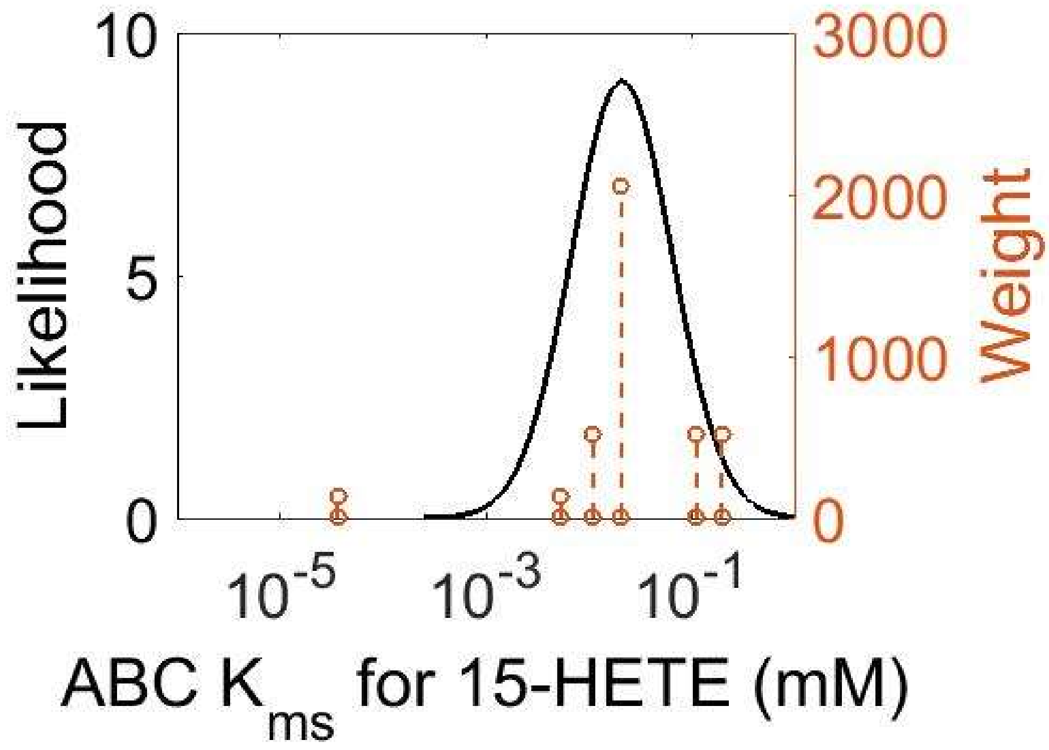


**Figure SF.10.7.1.2.1.** The estimated probability distribution for ABC $K_{ms}$. The value and weight of the literature values used to define the distribution are indicated by an orange dashed line. The x axis is plotted on a log-scale.

**S.10.7.1.3. Parameter: ABC concentration**

Parameter values for the ABC concentration of Reaction 28 were obtained from the literature and summarised in Table ST.10.7.1.3.1. The log-normal distribution properties for the ABC concentration of Reaction 28 are shown in Table ST.10.7.1.3.2 and plotted in Figure SF.10.7.1.3.1. To convert the enzyme concentration from ppm to mM, **Equation S.6.2** was used.

**Table ST.10.7.1.3.1.** Literature information used to design the ABC concentration parameter distribution. Each value was assigned a weight using the protocol described in Section 2.6.2. The type of error was assigned as 0 if it was additive and 1 if it was multiplicative.

| Value (mM) | Error (ppm) | Experimental details | | | | | | Weight | Type of error | Reference |
|---|---|---|---|---|---|---|---|---|---|---|
| | | Species | Expression Vector | Enzyme | pH | Temperature (°C) | Other | | | |
| 5.15 | NaN | Human | Stomach | ABC | 7.5 | 37 | | 2048 | 0 | (Wilhelm et al., 2014) |
| 5.94 | NaN | Human | Lung | ABC | 7.5 | 37 | | 2048 | 0 | (Kim et al., 2014) |
| 2.66 | NaN | Human | Gut | ABC | 7.5 | 37 | | 2048 | 0 | (Kim et al., 2014) |

**Table ST.10.7.1.3.2.** The log-normal distribution properties of the ABC concentration distribution. These values were calculated using the functions described in Section 2.6.2.

| Mode (ppm) | Mode (mM) | Confidence Interval | Location parameter (μ) | Scale parameter (σ) |
|---|---|---|---|---|
| 5.06 | $2.80 \times 10^{-5}$ | 1.44 | 1.74 | $3.42 \times 10^{-1}$ |

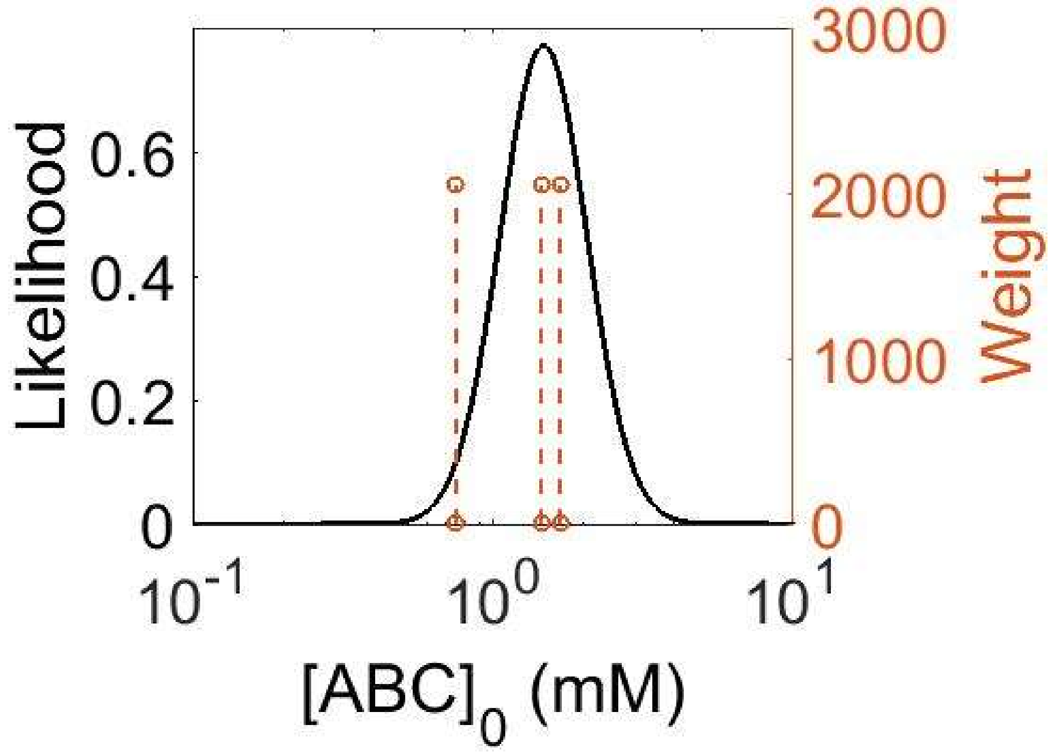


**Figure SF.10.7.1.3.1.** The estimated probability distribution for the ABC concentration. The value and weight of the literature values used to define the distribution are indicated by an orange dashed line. The x axis is plotted on a log-scale.

**S.10.8. Reaction 29: $LTB_4 \rightleftharpoons exLTB_4$**

$LTB_4$ is relocated from the intracellular compartment to the extracellular compartment via the ATP-binding cassette (ABC) transporter.

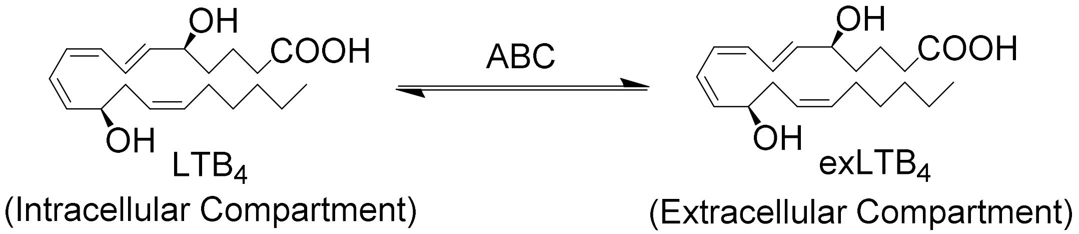


**Figure SF.10.8.** The transport of leukotriene $B_4$ ($LTB_4$) from the intracellular compartment to the extracellular compartment by ATP Binding Cassette protein (ABC) (Reaction 29).

**SEq.10.8.** Reaction rate law for Reaction 29.

$$v_{29} = [ABC] \cdot k_{cat} \cdot \frac{{LTB_4}/{K_m} \cdot \left(1 - \frac{exLTB_4}{LTB_4 \cdot e^{-0.5 \cdot \left(G + R \cdot T \cdot \ln\left(\frac{ATP}{ADP}\right)\right)/R \cdot T}}\right)}{1 + \frac{LTB_4}{K_m} + \frac{exLTB_4}{K_m} + ABC\_CI}$$

### S.10.8.1. Reaction parameters

### S.10.8.1.1. Parameter: ABC $k_{cat}$

Parameter values for the ABC $k_{cat}$ of Reaction 29 were obtained from the literature and summarised in Table ST.10.8.1.1.1. The log-normal distribution properties for the ABC $k_{cat}$ of Reaction 29 are shown in Table ST.10.8.1.1.2 and plotted in Figure SF.10.8.1.1.1.

**Table ST.10.8.1.1.1.** Literature information used to design the ABC $k_{cat}$ parameter distribution. Each value was assigned a weight using the protocol described in Section 2.6.2. The type of error was assigned as 0 if it was additive and 1 if it was multiplicative.

| Value ($min^{-1}$) | Error ($min^{-1}$) | Experimental details | | | | | | Weight | Type of error | Reference |
|---|---|---|---|---|---|---|---|---|---|---|
| | | Species | Expression Vector | Enzyme | pH | Temperature (°C) | Other | | | |
| 3.50 | NaN | Bacteria | E. coli. | Glucose transporter | Unknown | Unknown | Bionumbers calculation | **8** | 0 | (Waygood and Steeves, 1980) |
| 3.30 x10$^{-1}$ | NaN | Bacteria | E. coli. | Lactose transporter | Unknown | Unknown | Bionumbers calculation | **8** | 0 | (Naftalin et al., 2007) |
| 8.30 x10$^{-1}$ | NaN | Bacteria | E. coli. | Lactose transporter | Unknown | Unknown | Bionumbers calculation | **8** | 0 | (Naftalin et al., 2007) |
| 3.28 | NaN | Yeast | Saccharomyces cerevisiae | HXT7 transporter | Unknown | Unknown | Bionumbers calculation | **8** | 0 | (Ye et al., 2001) |
| 1.13 x10$^{1}$ | | Primate | Baculovirus | MRP2 | 7 | 37 | | 512 | | (Yasunaga et al., 2008) |

**Table ST.10.8.1.1.2.** The log-normal distribution properties of the ABC $k_{cat}$ distribution. These values were calculated using the functions described in Section 2.6.2.

| **Mode ($min^{-1}$)** | **Confidence Interval** | **Location parameter (μ)** | **Scale parameter (σ)** |
|---|---|---|---|
| $1.13 \times 10^{1}$ | $1.00 \times 10^{1}$ | 3.22 | $8.91 \times 10^{-1}$ |

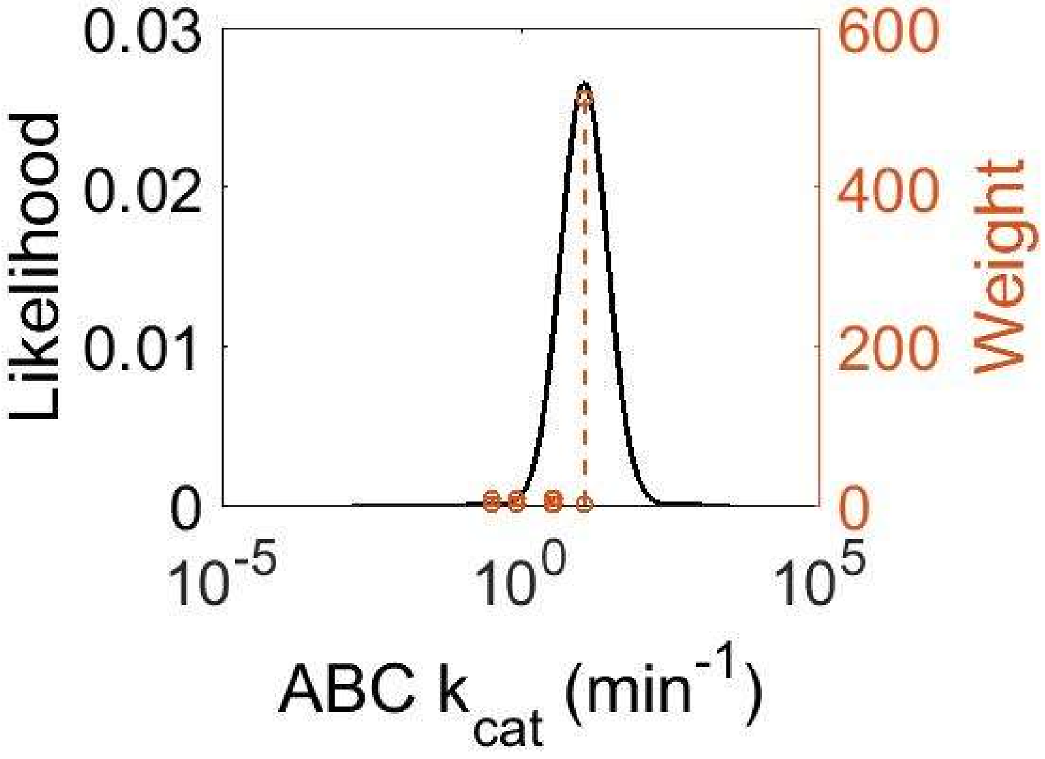


**Figure SF.10.8.1.1.1.** The estimated probability distribution for ABC $k_{cat}$. The value and weight of the literature values used to define the distribution are indicated by an orange dashed line. The x axis is plotted on a log-scale.

### S.10.8.1.2. Parameter: ABC $K_{ms}$

Parameter values for the ABC $K_{ms}$ of Reaction 29 were obtained from the literature and summarised in Table ST.10.8.1.2.1. The log-normal distribution properties for the ABC $K_{ms}$ of Reaction 29 are shown in Table ST.10.8.1.2.2 and plotted in Figure SF.10.8.1.2.1.

**Table ST.10.8.1.2.1.** Literature information used to design the ABC $K_{ms}$ parameter distribution. Each value was assigned a weight using the protocol described in Section 2.6.2. The type of error was assigned as 0 if it was additive and 1 if it was multiplicative.

| Value (mM) | Error (mM) | Experimental details | | | | | | Weight | Type of error | Reference |
|---|---|---|---|---|---|---|---|---|---|---|
| | | Species | Expression Vector | Enzyme | pH | Temperature (°C) | Other | | | |
| $1.09 \times 10^{-2}$ | $4.10 \times 10^{-3}$ | Human | Primate | MRP2 | 7 | 37 | | **512** | 0 | (Yasunaga et al., 2008) |
| $3.66 \times 10^{-5}$ | $3.80 \times 10^{-6}$ | Unknown | H69AR plasma membrane | MRP1 | 7.4 | 37 | | **128** | 0 | (Mao et al., 2000) |
| $5.30 \times 10^{-3}$ | $2.60 \times 10^{-3}$ | Human | HEK293 cells | MRP3 | 7.4 | 37 | | **128** | 0 | (Zeng et al., 2000) |
| $1.95 \times 10^{-1}$ | $6.12 \times 10^{-2}$ | Human | Primate | MRP2 | 7 | 37 | | **512** | 0 | (Yasunaga et al., 2008) |
| $1.10 \times 10^{-1}$ | $3.91 \times 10^{-2}$ | Human | Primate | MRP2 | 7 | 37 | | **512** | 0 | (Yasunaga et al., 2008) |
| $2.07 \times 10^{-2}$ | NaN | Human | HepG2 hepatoma | ABCB1 | 7.4 | 37 | | **2048** | 0 | (Fong et al., 2007) |

**Table ST.10.8.1.2.2.** The log-normal distribution properties of the ABC $K_{ms}$ distribution. These values were calculated using the functions described in Section 2.6.2.

| Mode (mM) | Confidence Interval | Location parameter (μ) | Scale parameter (σ) |
|---|---|---|---|
| $2.10 \times 10^{-2}$ | $2.37 \times 10^{1}$ | -2.60 | 1.12 |

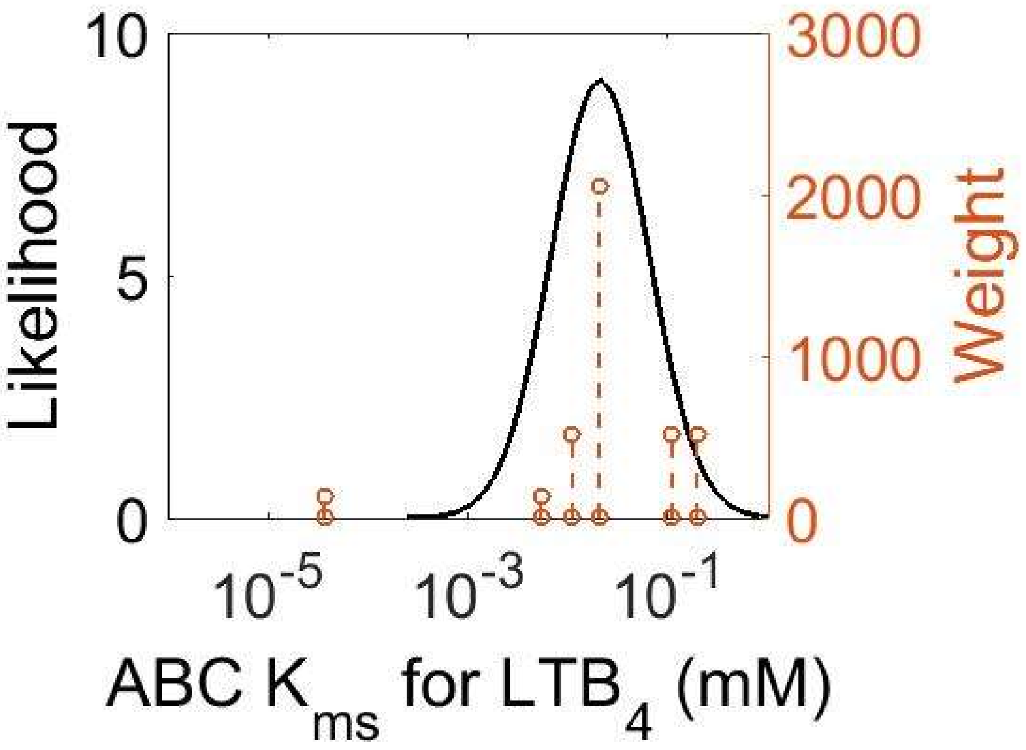


**Figure SF.10.8.1.2.1.** The estimated probability distribution for ABC $K_{ms}$. The value and weight of the literature values used to define the distribution are indicated by an orange dashed line. The x axis is plotted on a log-scale.

### S.10.8.1.3. Parameter: ABC concentration

Parameter values for the ABC concentration of Reaction 29 were obtained from the literature and summarised in Table ST.10.8.1.3.1. The log-normal distribution properties for the ABC concentration of Reaction 29 are shown in Table ST.10.8.1.3.2 and plotted in Figure SF.10.8.1.3.1. To convert the enzyme concentration from ppm to mM, **Equation S.6.2** was used.

**Table ST.10.8.1.3.1.** Literature information used to design the ABC concentration parameter distribution. Each value was assigned a weight using the protocol described in Section 2.6.2. The type of error was assigned as 0 if it was additive and 1 if it was multiplicative.

| Value (ppm) | Error (ppm) | Experimental details | | | | | | Weight | Type of error | Reference |
|---|---|---|---|---|---|---|---|---|---|---|
| | | Species | Expression Vector | Enzyme | pH | Temperature (°C) | Other | | | |
| 5.15 | NaN | Human | Stomach | ABC | 7.5 | 37 | | 2048 | 0 | (Wilhelm et al., 2014) |
| 5.94 | NaN | Human | Lung | ABC | 7.5 | 37 | | 2048 | 0 | (Kim et al., 2014) |
| 2.66 | NaN | Human | Gut | ABC | 7.5 | 37 | | 2048 | 0 | (Kim et al., 2014) |

**Table ST.10.8.1.3.2.** The log-normal distribution properties of the ABC concentration distribution. These values were calculated using the functions described in Section 2.6.2.

| Mode (ppm) | Mode (mM) | Confidence Interval | Location parameter (μ) | Scale parameter (σ) |
|---|---|---|---|---|
| 5.06 | $2.80 \times 10^{-5}$ | 1.44 | 1.74 | 3.4210-1 |

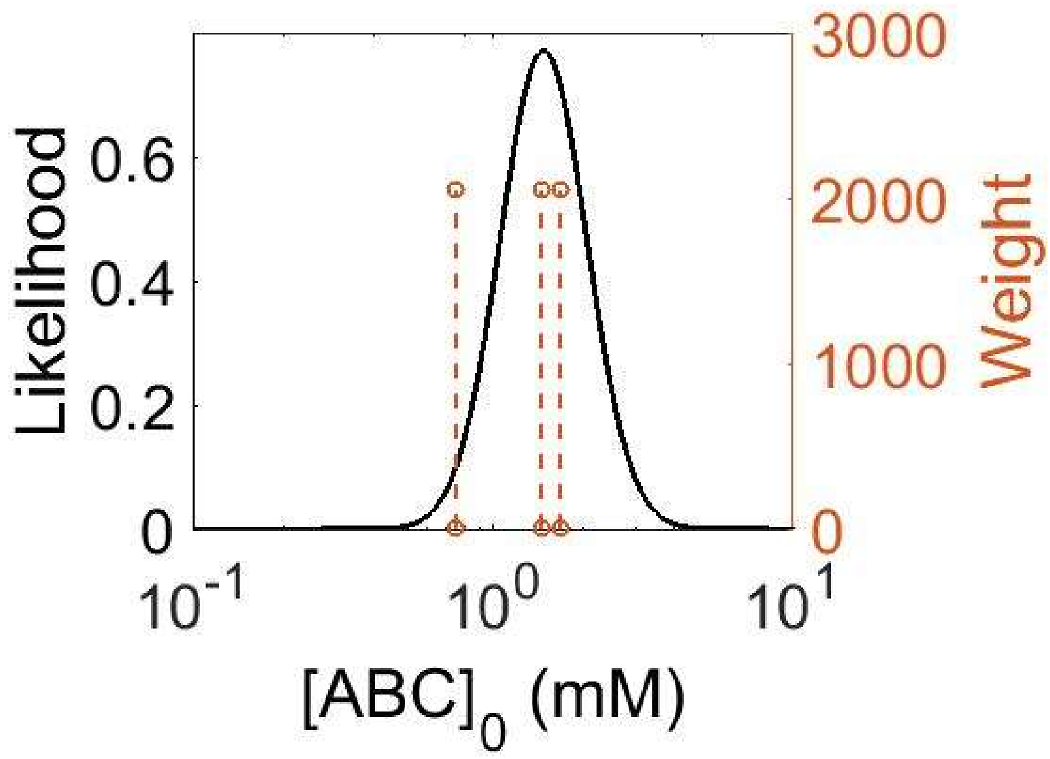


**Figure SF.10.8.1.3.1.** The estimated probability distribution for the ABC concentration. The value and weight of the literature values used to define the distribution are indicated by an orange dashed line. The x axis is plotted on a log-scale.

**S.10.9. Reaction 30: $LTC_4 \rightleftharpoons exLTC_4$**

$LTC_4$ is relocated from the intracellular compartment to the extracellular compartment via the ATP-binding cassette (ABC) transporter.

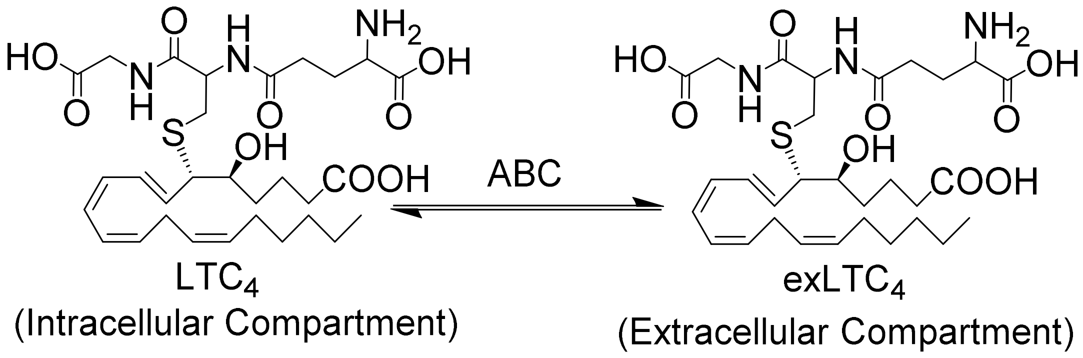


**Figure SF.10.9.** The transport of leukotriene $C_4$ ($LTC_4$) from the intracellular compartment to the extracellular compartment by ATP Binding Cassette protein (ABC) (Reaction 30).

**SEq.10.9.** Reaction rate law Reaction 30.

$$v_{30} = [ABC] \cdot k_{cat} \cdot \frac{\mathrm{LTC_4}/K_m \cdot \left(1 - \frac{\mathrm{exLTC_4}}{\mathrm{LTC_4} \cdot e^{-0.5 \cdot \left(G + R \cdot T \cdot \ln\left(\frac{ATP}{ADP}\right)\right)/R \cdot T}}\right)}{1 + \frac{\mathrm{LTC_4}}{K_m} + \frac{\mathrm{exLTC_4}}{K_m} + \mathrm{ABC\_CI}}$$

### S.10.9.1. Reaction parameters

#### S.10.9.1.1. Parameter: ABC $k_{cat}$

Parameter values for the ABC $k_{cat}$ of Reaction 30 were obtained from the literature and summarised in Table ST.10.9.1.1.1. The log-normal distribution properties for the ABC $k_{cat}$ of Reaction 30 are shown in Table ST.10.9.1.1.2 and plotted in Figure SF.10.9.1.1.1.

**Table ST.10.9.1.1.1.** Literature information used to design the ABC $k_{cat}$ parameter distribution. Each value was assigned a weight using the protocol described in Section 2.6.2. The type of error was assigned as 0 if it was additive and 1 if it was multiplicative.

| Value (min-1) | Error (min-1) | Experimental details | | | | | | Weight | Type of error | Reference |
|---|---|---|---|---|---|---|---|---|---|---|
| | | Species | Expression Vector | Enzyme | pH | Temperature (°C) | Other | | | |
| 3.50 | NaN | Bacteria | E. coli. | Glucose transporter | Unknown | Unknown | Bionumbers calculation | **8** | 0 | (Waygood and Steeves, 1980) |
| $3.30 \times 10^{-1}$ | NaN | Bacteria | E. coli. | Lactose transporter | Unknown | Unknown | Bionumbers calculation | **8** | 0 | (Naftalin et al., 2007) |
| $8.30 \times 10^{-1}$ | NaN | Bacteria | E. coli. | Lactose transporter | Unknown | Unknown | Bionumbers calculation | **8** | 0 | (Naftalin et al., 2007) |
| 3.28 | NaN | Yeast | Saccharomyces cerevisiae | HXT7 transporter | Unknown | Unknown | Bionumbers calculation | **8** | 0 | (Ye et al., 2001) |
| $1.13 \times 10^{1}$ | | Primate | Baculovirus | MRP2 | 7 | 37 | | 512 | | (Yasunaga et al., 2008) |

**Table ST.10.9.1.1.2.** The log-normal distribution properties of the ABC $k_{cat}$ distribution. These values were calculated using the functions described in Section 2.6.2.

| **Mode ($min^{-1}$)** | **Confidence Interval** | **Location parameter (μ)** | **Scale parameter (σ)** |
|---|---|---|---|
| 1.13 $x10^{1}$ | 1.00 $x10^{1}$ | 3.22 | $8.9110^{-1}$ |

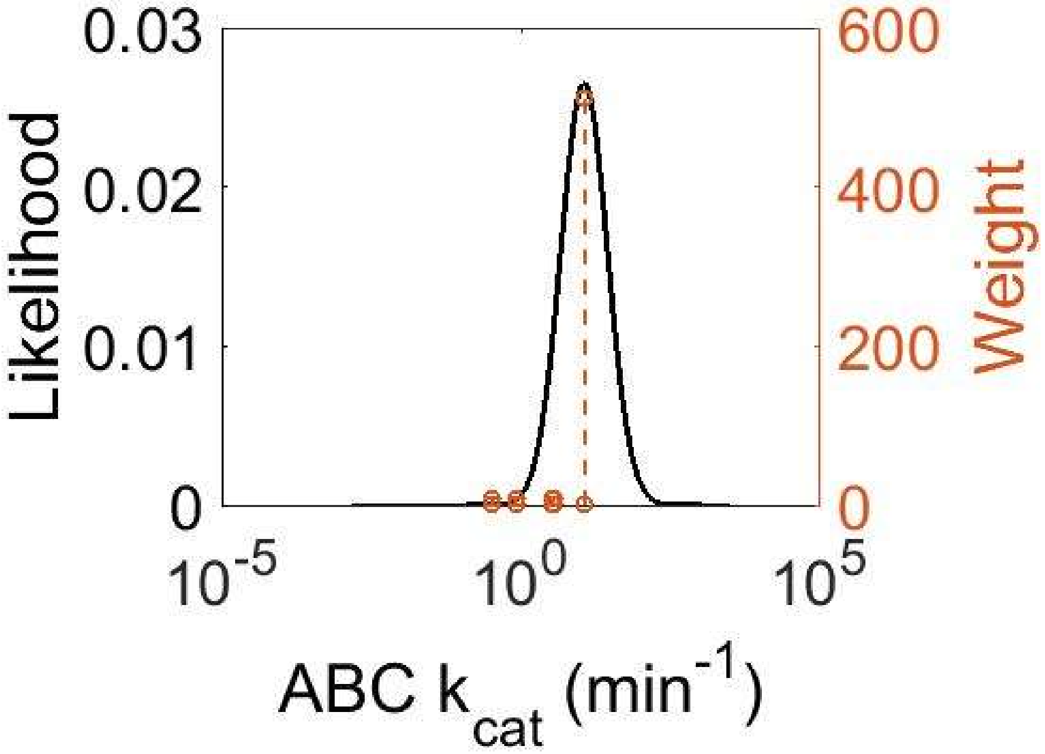


**Figure SF.10.9.1.1.1.** The estimated probability distribution for ABC $k_{cat}$. The value and weight of the literature values used to define the distribution are indicated by an orange dashed line. The x axis is plotted on a log-scale.

### S.10.9.1.2. Parameter: ABC $K_{ms}$

Parameter values for the ABC $K_{ms}$ of Reaction 30 were obtained from the literature and summarised in Table ST.10.9.1.2.1. The log-normal distribution properties for the ABC $K_{ms}$ of Reaction 30 are shown in Table ST.10.9.1.2.2 and plotted in Figure SF.10.9.1.2.1.

**Table ST.10.9.1.2.1.** Literature information used to design the ABC $K_{ms}$ parameter distribution. Each value was assigned a weight using the protocol described in Section 2.6.2. The type of error was assigned as 0 if it was additive and 1 if it was multiplicative.

| Value (mM) | Error (mM) | Experimental details | | | | | | Weight | Type of error | Reference |
|---|---|---|---|---|---|---|---|---|---|---|
| | | Species | Expression Vector | Enzyme | pH | Temperature (°C) | Other | | | |
| $1.09 \times 10^{-2}$ | $4.10 \times 10^{-3}$ | Human | Primate | MRP2 | 7 | 37 | | **512** | 0 | (Yasunaga et al., 2008) |
| $3.66 \times 10^{-5}$ | $3.80 \times 10^{-6}$ | Unknown | H69AR plasma membrane | MRP1 | 7.4 | 37 | | **128** | 0 | (Mao et al., 2000) |
| $5.30 \times 10^{-3}$ | $2.60 \times 10^{-3}$ | Human | HEK293 cells | MRP3 | 7.4 | 37 | | **128** | 0 | (Zeng et al., 2000) |
| $1.95 \times 10^{-1}$ | $6.12 \times 10^{-2}$ | Human | Primate | MRP2 | 7 | 37 | | **512** | 0 | (Yasunaga et al., 2008) |
| $1.10 \times 10^{-1}$ | $3.91 \times 10^{-2}$ | Human | Primate | MRP2 | 7 | 37 | | **512** | 0 | (Yasunaga et al., 2008) |
| $2.07 \times 10^{-2}$ | NaN | Human | HepG2 hepatoma | ABCB1 | 7.4 | 37 | | **2048** | 0 | (Fong et al., 2007) |

**Table ST.10.9.1.2.2.** The log-normal distribution properties of the ABC $K_{ms}$ distribution. These values were calculated using the functions described in Section 2.6.2.

| Mode (mM) | Confidence Interval | Location parameter (μ) | Scale parameter (σ) |
|---|---|---|---|
| $2.10 \times 10^{-2}$ | $2.37 \times 10^{1}$ | -2.60 | 1.12 |

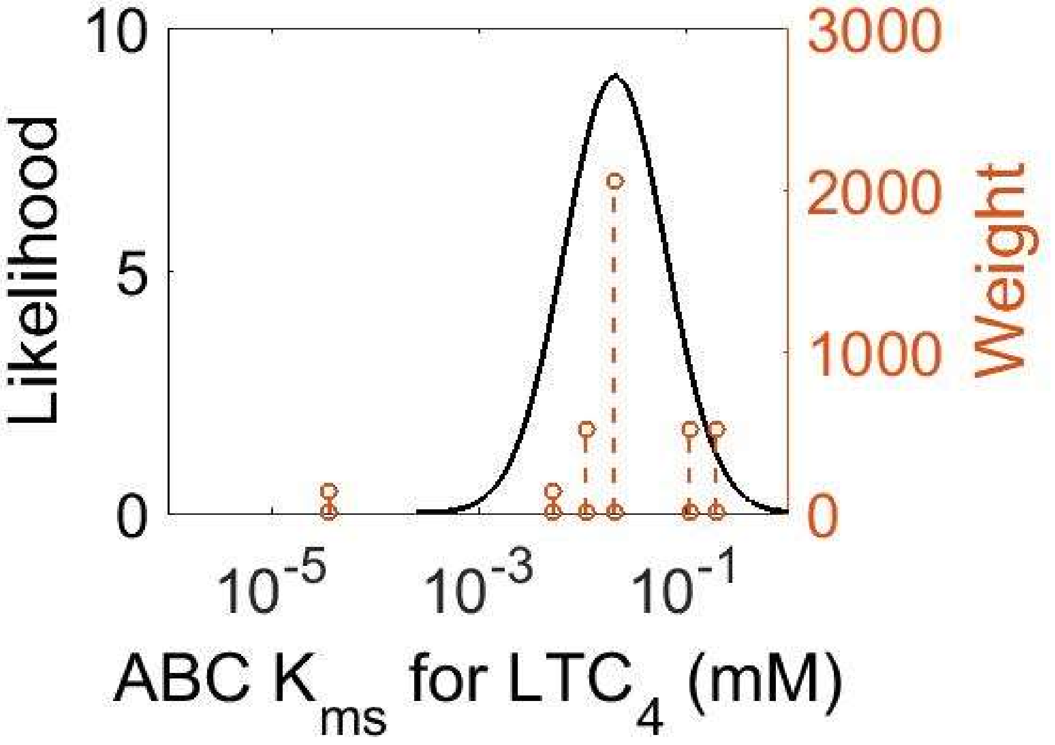


**Figure SF.10.9.1.2.1.** The estimated probability distribution for ABC $K_{ms}$. The value and weight of the literature values used to define the distribution are indicated by an orange dashed line. The x axis is plotted on a log-scale.

**S.10.9.1.3. Parameter: ABC concentration**

Parameter values for the ABC concentration of Reaction 30 were obtained from the literature and summarised in Table ST.10.9.1.3.1. The log-normal distribution properties for the ABC concentration of Reaction 30 are shown in Table ST.10.9.1.3.2 and plotted in Figure SF.10.9.1.3.1. To convert the enzyme concentration from ppm to mM, **Equation S.6.2** was used.

**Table ST.10.9.1.3.1.** Literature information used to design the ABC concentration parameter distribution. Each value was assigned a weight using the protocol described in Section 2.6.2. The type of error was assigned as 0 if it was additive and 1 if it was multiplicative.

| Value (ppm) | Error (ppm) | Experimental details | | | | | | Weight | Type of error | Reference |
|---|---|---|---|---|---|---|---|---|---|---|
| | | Species | Expression Vector | Enzyme | pH | Temperature (°C) | Other | | | |
| 5.15 | NaN | Human | Stomach | ABC | 7.5 | 37 | | 2048 | 0 | (Wilhelm et al., 2014) |
| 5.94 | NaN | Human | Lung | ABC | 7.5 | 37 | | 2048 | 0 | (Kim et al., 2014) |
| 2.66 | NaN | Human | Gut | ABC | 7.5 | 37 | | 2048 | 0 | (Kim et al., 2014) |

**Table ST.10.9.1.3.2.** The log-normal distribution properties of the ABC concentration distribution. These values were calculated using the functions described in Section 2.6.2.

| Mode (ppm) | Mode (mM) | Confidence Interval | Location parameter (μ) | Scale parameter (σ) |
|---|---|---|---|---|
| 5.06 | $2.80 \times 10^{-5}$ | 1.44 | 1.74 | $3.4210^{-1}$ |

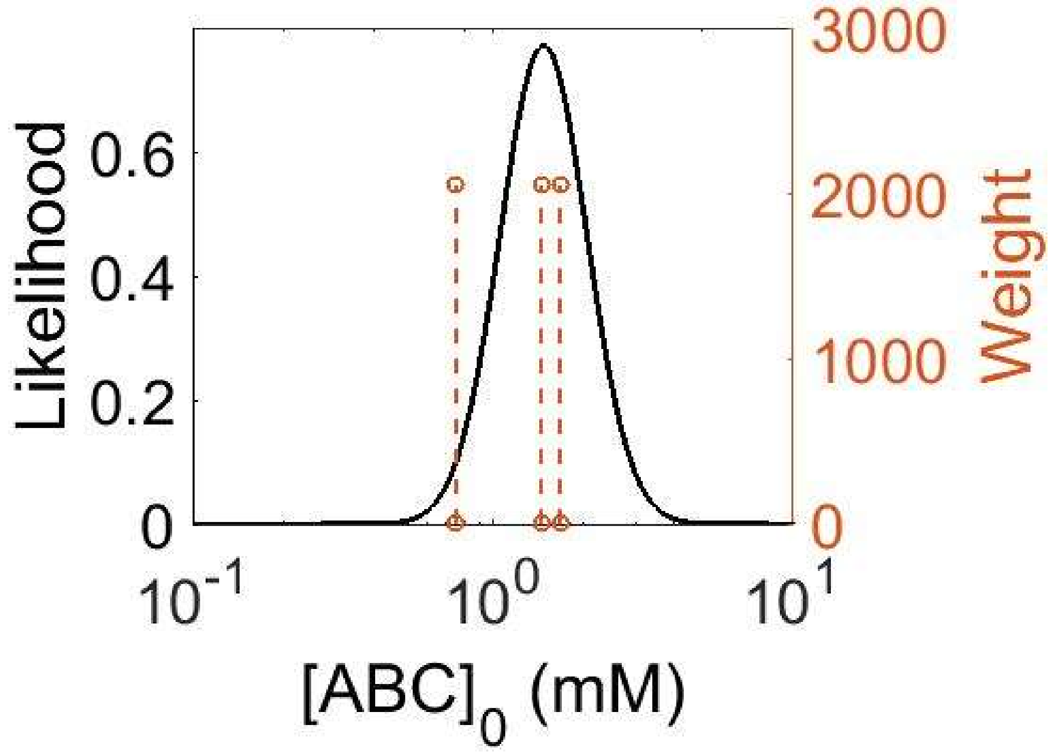


**Figure SF.10.9.1.3.1.** The estimated probability distribution for the ABC concentration. The value and weight of the literature values used to define the distribution are indicated by an orange dashed line. The x axis is plotted on a log-scale.

**S.10.10. Reaction 31: 12-HETE ⇌ ex12-HETE**

12-HETE is relocated from the intracellular compartment to the extracellular compartment via the ATP-binding cassette (ABC) transporter.

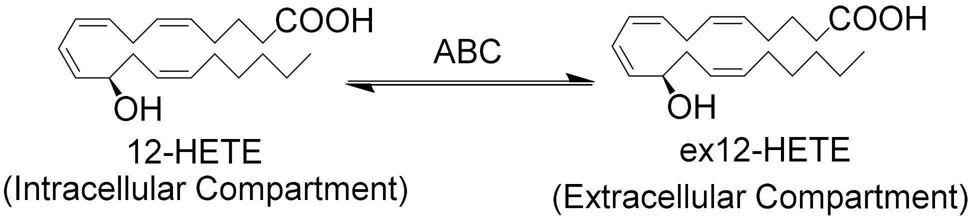


**Figure SF.10.10.** The transport of 12-hydroxyeicosatetraenoic acid (12-HETE) from the intracellular compartment to the extracellular compartment by ATP Binding Cassette protein (ABC) (Reaction 31).

**SEq.10.10.** Reaction rate law for Reaction 31.

$$\boldsymbol{v_{31}} = [\boldsymbol{ABC}] \cdot \boldsymbol{k_{cat}} \cdot \frac{\mathbf{12-HETE}/\boldsymbol{K_m} \cdot \left(\mathbf{1} - \frac{\mathbf{ex12-HETE}}{\mathbf{12-HETE} \cdot \boldsymbol{e}^{-0.5\cdot\left(\boldsymbol{G}+\boldsymbol{R}\cdot\boldsymbol{T}\cdot\ln\left(\frac{\boldsymbol{ATP}}{\boldsymbol{ADP}}\right)\right)/\boldsymbol{R}\cdot\boldsymbol{T}}}\right)}{\mathbf{1} + \frac{\mathbf{12-HETE}}{\boldsymbol{K_m}} + \frac{\mathbf{ex12-HETE}}{\boldsymbol{K_m}} + \mathbf{ABC\_CI}}$$

## S.10.10.1. Reaction parameters

### S.10.10.1.1. Parameter: ABC $k_{cat}$

Parameter values for the ABC $k_{cat}$ of Reaction 31 were obtained from the literature and summarised in Table ST.10.10.1.1.1. The log-normal distribution properties for the ABC $k_{cat}$ of Reaction 31 are shown in Table ST.10.10.1.1.2 and plotted in Figure SF.10.10.1.1.1.

**Table ST.10.10.1.1.1.** Literature information used to design the ABC $k_{cat}$ parameter distribution. Each value was assigned a weight using the protocol described in Section 2.6.2. The type of error was assigned as 0 if it was additive and 1 if it was multiplicative.

| Value ($min^{-1}$) | Error ($min^{-1}$) | Experimental details | | | | | | Weight | Type of error | Reference |
|---|---|---|---|---|---|---|---|---|---|---|
| | | Species | Expression Vector | Enzyme | pH | Temperature (°C) | Other | | | |
| 3.50 | NaN | Bacteria | E. coli. | Glucose transporter | Unknown | Unknown | Bionumbers calculation | **8** | 0 | (Waygood and Steeves, 1980) |
| 3.30 x$10^{-1}$ | NaN | Bacteria | E. coli. | Lactose transporter | Unknown | Unknown | Bionumbers calculation | **8** | 0 | (Naftalin et al., 2007) |
| 8.30 x$10^{-1}$ | NaN | Bacteria | E. coli. | Lactose transporter | Unknown | Unknown | Bionumbers calculation | **8** | 0 | (Naftalin et al., 2007) |
| 3.28 | NaN | Yeast | Saccharomyces cerevisiae | HXT7 transporter | Unknown | Unknown | Bionumbers calculation | **8** | 0 | (Ye et al., 2001) |
| 1.13 x$10^{1}$ | | Primate | Baculovirus | MRP2 | 7 | 37 | | 512 | | (Yasunaga et al., 2008) |

**Table ST.10.10.1.1.2.** The log-normal distribution properties of the ABC $k_{cat}$ distribution. These values were calculated using the functions described in Section 2.6.2.

| Mode ($min^{-1}$) | Confidence Interval | Location parameter (μ) | Scale parameter (σ) |
|---|---|---|---|
| 1.13 $x10^{1}$ | 1.00 $x10^{1}$ | 3.22 | $8.9110^{-1}$ |

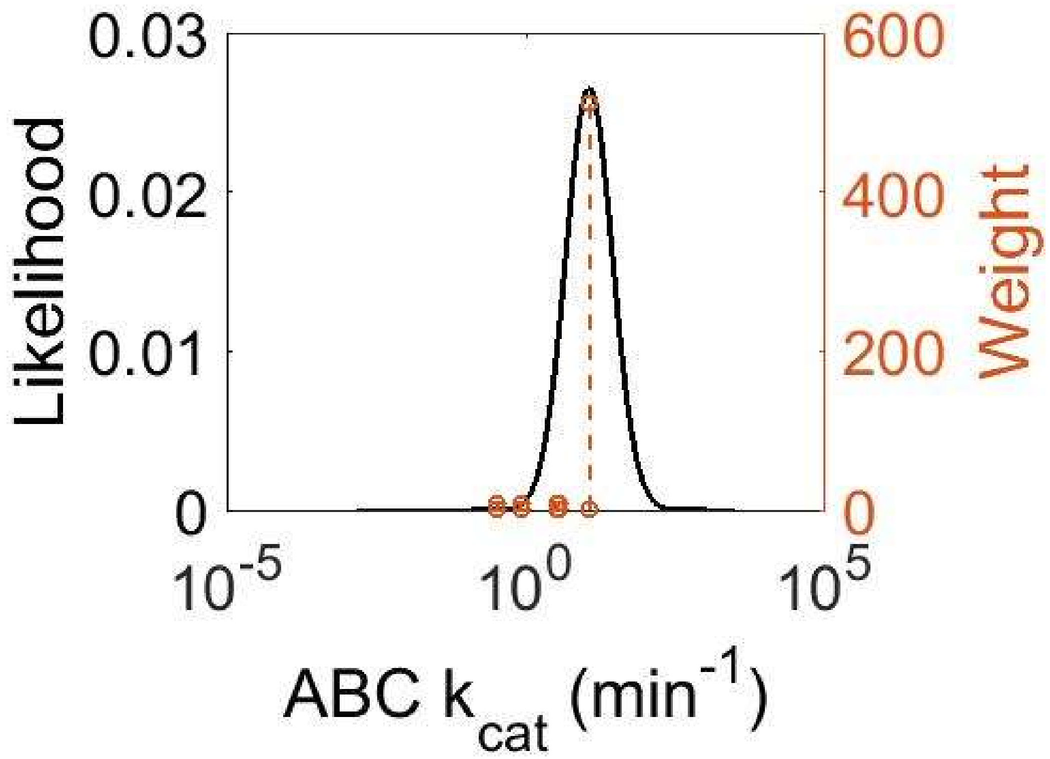


**Figure SF.10.10.1.1.1.** The estimated probability distribution for ABC $k_{cat}$. The value and weight of the literature values used to define the distribution are indicated by an orange dashed line. The x axis is plotted on a log-scale.

### S.10.10.1.2. Parameter: ABC $K_{ms}$

Parameter values for the ABC $K_{ms}$ of Reaction 31 were obtained from the literature and summarised in Table ST.10.10.1.2.1. The log-normal distribution properties for the ABC $K_{ms}$ of Reaction 31 are shown in Table ST.10.10.1.2.2 and plotted in Figure SF.10.10.1.2.1.

**Table ST.10.10.1.2.1.** Literature information used to design the ABC $K_{ms}$ parameter distribution. Each value was assigned a weight using the protocol described in Section 2.6.2. The type of error was assigned as 0 if it was additive and 1 if it was multiplicative.

| **Value (mM)** | **Error (mM)** | **Experimental details** | | | | | | **Weight** | **Type of error** | **Reference** |
|---|---|---|---|---|---|---|---|---|---|---|
| | | **Species** | **Expression Vector** | **Enzyme** | **pH** | **Temperature (°C)** | **Other** | | | |
| $1.09 \times 10^{-2}$ | $4.10 \times 10^{-3}$ | Human | Primate | MRP2 | 7 | 37 | | **512** | 0 | (Yasunaga et al., 2008) |
| $3.66 \times 10^{-5}$ | $3.80 \times 10^{-6}$ | Unknown | H69AR plasma membrane | MRP1 | 7.4 | 37 | | **128** | 0 | (Mao et al., 2000) |
| $5.30 \times 10^{-3}$ | $2.60 \times 10^{-3}$ | Human | HEK293 cells | MRP3 | 7.4 | 37 | | **128** | 0 | (Zeng et al., 2000) |
| $1.95 \times 10^{-1}$ | $6.12 \times 10^{-2}$ | Human | Primate | MRP2 | 7 | 37 | | **512** | 0 | (Yasunaga et al., 2008) |
| $1.10 \times 10^{-1}$ | $3.91 \times 10^{-2}$ | Human | Primate | MRP2 | 7 | 37 | | **512** | 0 | (Yasunaga et al., 2008) |
| $2.07 \times 10^{-2}$ | NaN | Human | HepG2 hepatoma | ABCB1 | 7.4 | 37 | | **2048** | 0 | (Fong et al., 2007) |

**Table ST.10.10.1.2.2.** The log-normal distribution properties of the ABC $K_{ms}$ distribution. These values were calculated using the functions described in Section 2.6.2.

| **Mode (mM)** | **Confidence Interval** | **Location parameter (μ)** | **Scale parameter (σ)** |
|---|---|---|---|
| $2.10 \times 10^{-2}$ | $2.37 \times 10^{1}$ | -2.60 | 1.12 |

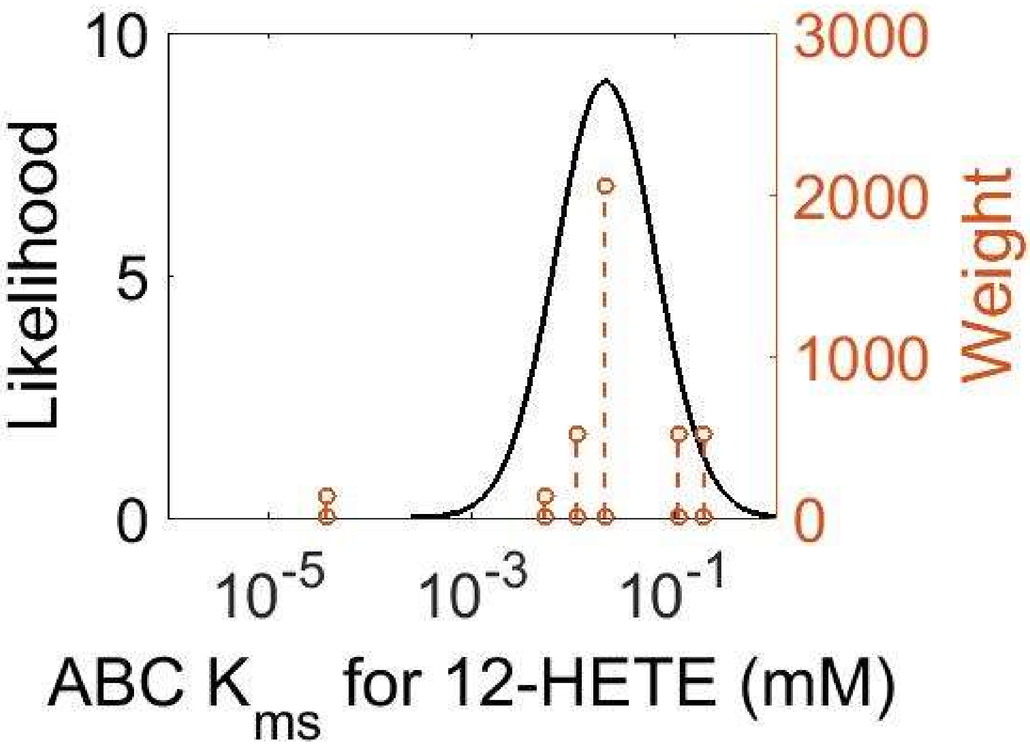


**Figure SF.10.10.1.2.1.** The estimated probability distribution for ABC $K_{ms}$. The value and weight of the literature values used to define the distribution are indicated by an orange dashed line. The x axis is plotted on a log-scale.

**S.10.10.1.3. Parameter: ABC concentration**

Parameter values for the ABC concentration of Reaction 31 were obtained from the literature and summarised in Table ST.10.10.1.3.1. The log-normal distribution properties for the ABC concentration of Reaction 31 are shown in Table ST.10.10.1.3.2 and plotted in Figure SF.10.10.1.3.1. To convert the enzyme concentration from ppm to mM, **Equation S.6.2** was used.

**Table ST.10.10.1.3.1.** Literature information used to design the ABC concentration parameter distribution. Each value was assigned a weight using the protocol described in Section 2.6.2. The type of error was assigned as 0 if it was additive and 1 if it was multiplicative.

| Value (ppm) | Error (ppm) | Experimental details | | | | | | Weight | Type of error | Reference |
|---|---|---|---|---|---|---|---|---|---|---|
| | | Species | Expression Vector | Enzyme | pH | Temperature (°C) | Other | | | |
| 5.15 | NaN | Human | Stomach | ABC | 7.5 | 37 | | 2048 | 0 | (Wilhelm et al., 2014) |
| 5.94 | NaN | Human | Lung | ABC | 7.5 | 37 | | 2048 | 0 | (Kim et al., 2014) |
| 2.66 | NaN | Human | Gut | ABC | 7.5 | 37 | | 2048 | 0 | (Kim et al., 2014) |

**Table ST.10.10.1.3.2.** The log-normal distribution properties of the ABC concentration distribution. These values were calculated using the functions described in Section 2.6.2.

| Mode (ppm) | Mode (mM) | Confidence Interval | Location parameter (μ) | Scale parameter (σ) |
|---|---|---|---|---|
| 5.06 | $2.80 \times 10^{-5}$ | 1.44 | 1.74 | $3.4210^{-1}$ |

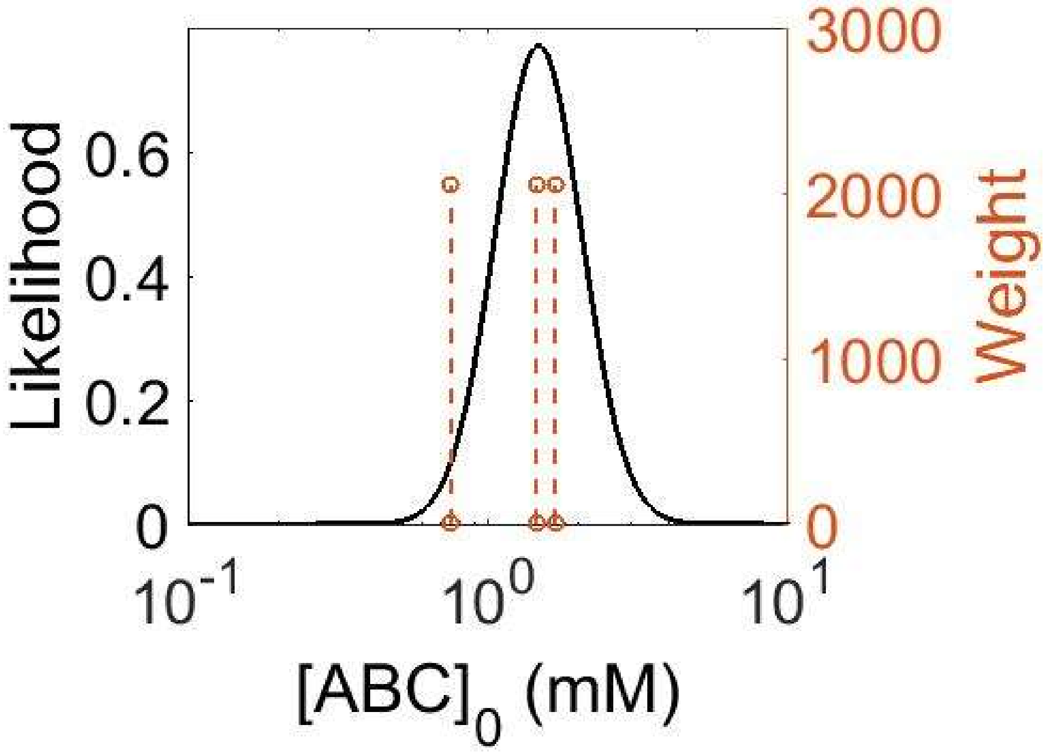


**Figure SF.10.10.1.3.1.** The estimated probability distribution for the ABC concentration. The value and weight of the literature values used to define the distribution are indicated by an orange dashed line. The x axis is plotted on a log-scale.

**S.10.11. Reaction 32: $TXA_2 \rightleftharpoons exTXA_2$**

$TXA_2$ is relocated from the intracellular compartment to the extracellular compartment via the ATP-binding cassette (ABC) transporter.

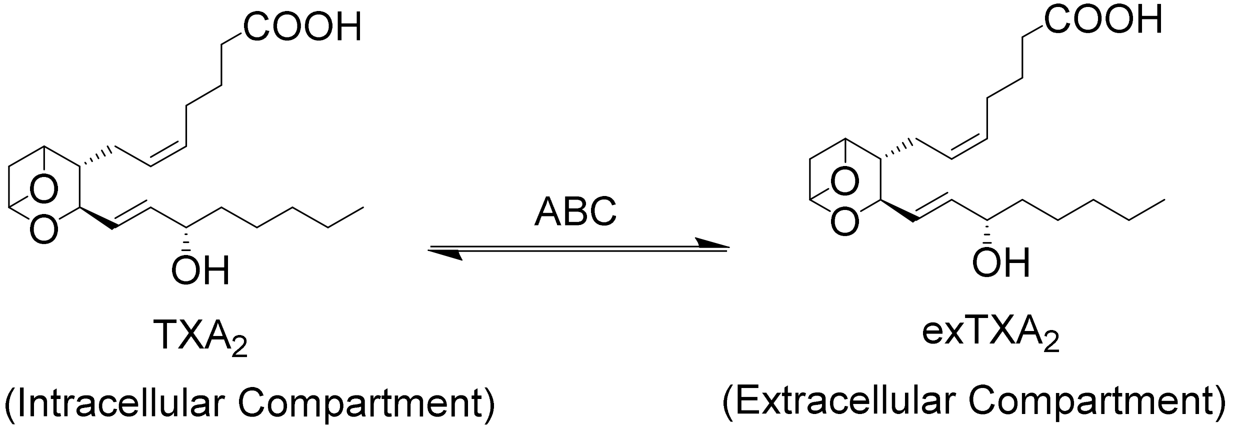


**Figure SF.10.11.** The transport of thromboxane $A_2$ ($TXA_2$) from the intracellular compartment to the extracellular compartment by ATP Binding Cassette protein (ABC) (Reaction 32).

**SEq.10.11.** Reaction rate law for Reaction 32.

$$v_{32} = [ABC] \cdot k_{cat} \cdot \frac{\mathrm{TXA_2}/K_m \cdot \left(1 - \frac{\mathrm{exTXA_2}}{\mathrm{TXA_2} \cdot e^{-0.5 \cdot \left(G + R \cdot T \cdot \ln\left(\frac{ATP}{ADP}\right)\right)/R \cdot T}}\right)}{1 + \frac{\mathrm{TXA_2}}{K_m} + \frac{\mathrm{exTXA_2}}{K_m} + \mathrm{ABC\_CI}}$$

## S.10.11.1. Reaction parameters

### S.10.11.1.1 Parameter: ABC $k_{cat}$

Parameter values for the ABC $k_{cat}$ of Reaction 32 were obtained from the literature and summarised in Table ST.10.11.1.1.1. The log-normal distribution properties for the ABC $k_{cat}$ of Reaction 32 are shown in Table ST.10.11.1.1.2 and plotted in Figure SF.10.11.1.1.1.

**Table ST.10.11.1.1.1.** Literature information used to design the ABC $k_{cat}$ parameter distribution. Each value was assigned a weight using the protocol described in Section 2.6.2. The type of error was assigned as 0 if it was additive and 1 if it was multiplicative.

| Value ($min^{-1}$) | Error ($min^{-1}$) | Experimental details | | | | | | Weight | Type of error | Reference |
|---|---|---|---|---|---|---|---|---|---|---|
| | | Species | Expression Vector | Enzyme | pH | Temperature (°C) | Other | | | |
| 3.50 | NaN | Bacteria | E. coli. | Glucose transporter | Unknown | Unknown | Bionumbers calculation | 8 | 0 | (Waygood and Steeves, 1980) |
| $3.30 \times 10^{-1}$ | NaN | Bacteria | E. coli. | Lactose transporter | Unknown | Unknown | Bionumbers calculation | 8 | 0 | (Naftalin et al., 2007) |
| $8.30 \times 10^{-1}$ | NaN | Bacteria | E. coli. | Lactose transporter | Unknown | Unknown | Bionumbers calculation | 8 | 0 | (Naftalin et al., 2007) |
| 3.28 | NaN | Yeast | Saccharomyces cerevisiae | HXT7 transporter | Unknown | Unknown | Bionumbers calculation | 8 | 0 | (Ye et al., 2001) |
| $1.13 \times 10^{1}$ | | Primate | Baculovirus | MRP2 | 7 | 37 | | 512 | | (Yasunaga et al., 2008) |

**Table ST.10.11.1.1.2.** The log-normal distribution properties of the ABC $k_{cat}$ distribution. These values were calculated using the functions described in Section 2.6.2.

| Mode ($min^{-1}$) | Confidence Interval | Location parameter (μ) | Scale parameter (σ) |
|---|---|---|---|
| 1.13 $x10^{1}$ | 1.00 $x10^{1}$ | 3.22 | $8.9110^{-1}$ |

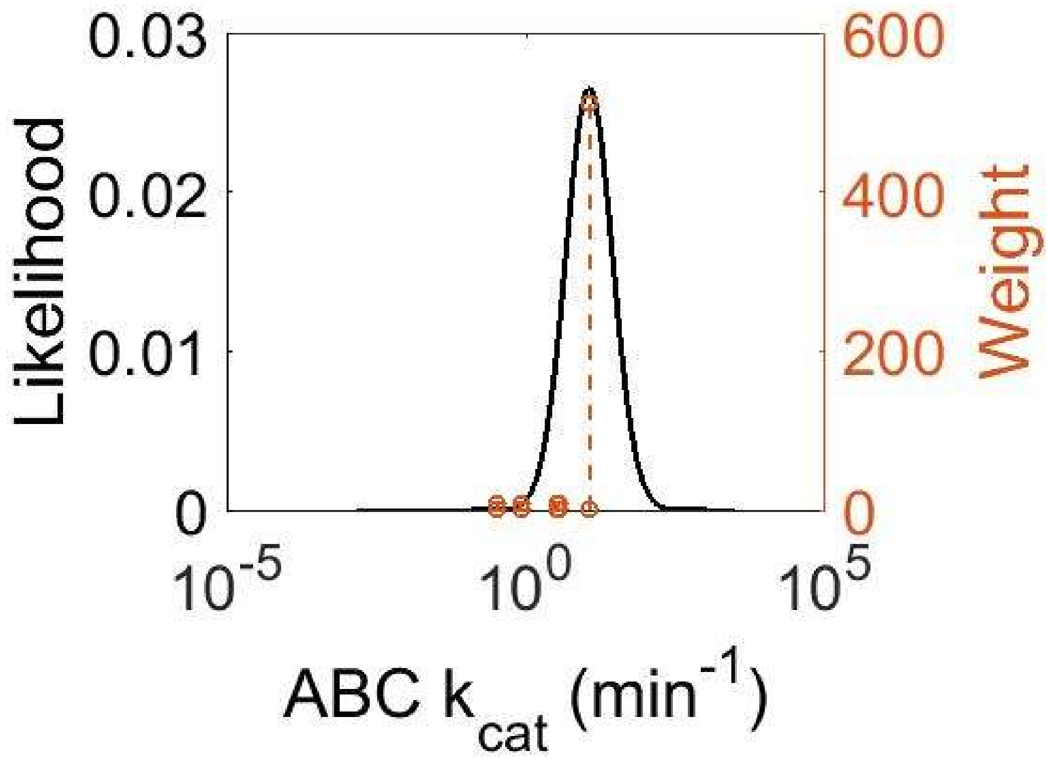


**Figure SF.10.11.1.1.1.** The estimated probability distribution for ABC $k_{cat}$. The value and weight of the literature values used to define the distribution are indicated by an orange dashed line. The x axis is plotted on a log-scale.

**S.10.11.1.2. Parameter: ABC $K_{ms}$**

Parameter values for the ABC $K_{ms}$ of Reaction 32 were obtained from the literature and summarised in Table ST.10.11.1.2.1. The log-normal distribution properties for the ABC $K_{ms}$ of Reaction 32 are shown in Table ST.10.11.1.2.2 and plotted in Figure SF.10.11.1.2.1.

**Table ST.10.11.1.2.1.** Literature information used to design the ABC $K_{ms}$ parameter distribution. Each value was assigned a weight using the protocol described in Section 2.6.2. The type of error was assigned as 0 if it was additive and 1 if it was multiplicative.

| Value (mM) | Error (mM) | Experimental details | | | | | | Weight | Type of error | Reference |
|---|---|---|---|---|---|---|---|---|---|---|
| | | Species | Expression Vector | Enzyme | pH | Temperature (°C) | Other | | | |
| 1.09 x10$^{-2}$ | 4.10 x10$^{-3}$ | Human | Primate | MRP2 | 7 | 37 | | **512** | 0 | (Yasunaga et al., 2008) |
| 3.66 x10$^{-5}$ | 3.80 x10$^{-6}$ | Unknown | H69AR plasma membrane | MRP1 | 7.4 | 37 | | **128** | 0 | (Mao et al., 2000) |
| 5.30 x10$^{-3}$ | 2.60 x10$^{-3}$ | Human | HEK293 cells | MRP3 | 7.4 | 37 | | **128** | 0 | (Zeng et al., 2000) |
| 1.95 x10$^{-1}$ | 6.12 x10$^{-2}$ | Human | Primate | MRP2 | 7 | 37 | | **512** | 0 | (Yasunaga et al., 2008) |
| 1.10 x10$^{-1}$ | 3.91 x10$^{-2}$ | Human | Primate | MRP2 | 7 | 37 | | **512** | 0 | (Yasunaga et al., 2008) |
| 2.07 x10$^{-2}$ | NaN | Human | HepG2 hepatoma | ABCB1 | 7.4 | 37 | | **2048** | 0 | (Fong et al., 2007) |

**Table ST.10.11.1.2.2.** The log-normal distribution properties of the ABC $K_{ms}$ distribution. These values were calculated using the functions described in Section 2.6.2.

| **Mode (mM)** | **Confidence Interval** | **Location parameter (μ)** | **Scale parameter (σ)** |
|---|---|---|---|
| $2.10 \times 10^{-2}$ | $2.37 \times 10^{1}$ | -2.60 | 1.12 |

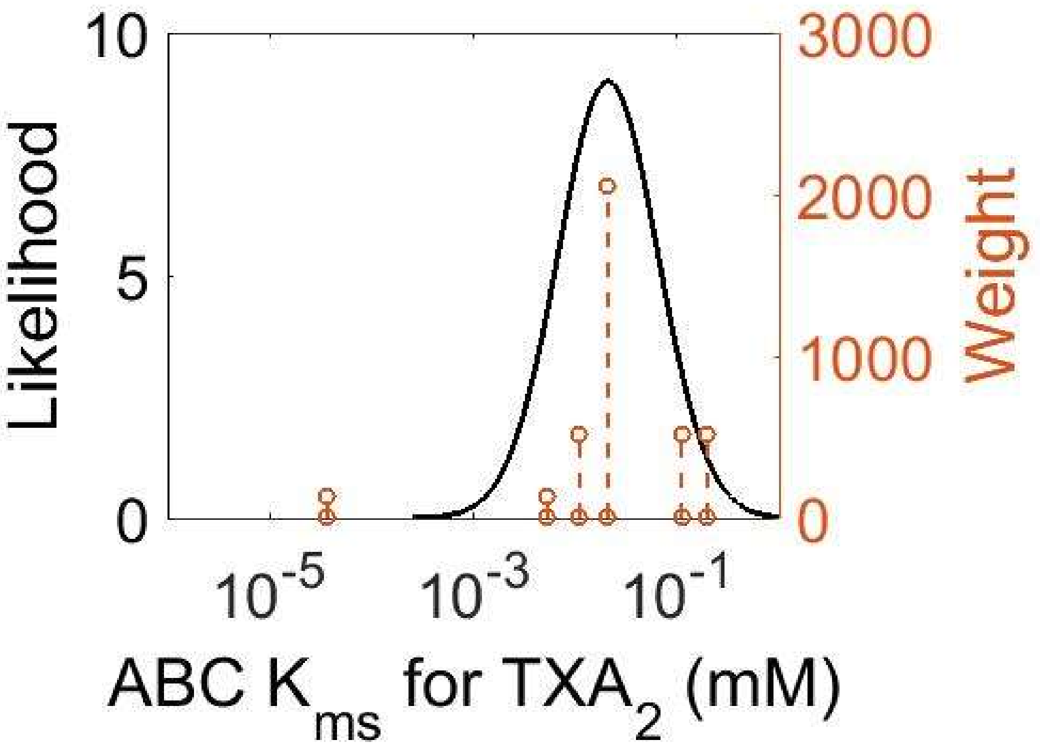


**Figure SF.10.11.1.2.1.** The estimated probability distribution for ABC $K_{ms}$. The value and weight of the literature values used to define the distribution are indicated by an orange dashed line. The x axis is plotted on a log-scale.

**S.10.11.1.3. Parameter: ABC concentration**

Parameter values for the ABC concentration of Reaction 32 were obtained from the literature and summarised in Table ST.10.11.1.3.1. The log-normal distribution properties for the ABC concentration of Reaction 32 are shown in Table ST.10.11.1.3.2 and plotted in Figure SF.10.11.1.3.1. To convert the enzyme concentration from ppm to mM, **Equation S.6.2** was used.

**Table ST.10.11.1.3.1.** Literature information used to design the ABC concentration parameter distribution. Each value was assigned a weight using the protocol described in Section 2.6.2. The type of error was assigned as 0 if it was additive and 1 if it was multiplicative.

| Value (ppm) | Error (ppm) | Experimental details | | | | | | Weight | Type of error | Reference |
|---|---|---|---|---|---|---|---|---|---|---|
| | | Species | Expression Vector | Enzyme | pH | Temperature (°C) | Other | | | |
| 5.15 | NaN | Human | Stomach | ABC | 7.5 | 37 | | 2048 | 0 | (Wilhelm et al., 2014) |
| 5.94 | NaN | Human | Lung | ABC | 7.5 | 37 | | 2048 | 0 | (Kim et al., 2014) |
| 2.66 | NaN | Human | Gut | ABC | 7.5 | 37 | | 2048 | 0 | (Kim et al., 2014) |

**Table ST.10.11.1.3.2.** The log-normal distribution properties of the ABC concentration distribution. These values were calculated using the functions described in Section 2.6.2.

| Mode (ppm) | Mode (mM) | Confidence Interval | Location parameter (μ) | Scale parameter (σ) |
|---|---|---|---|---|
| 5.06 | $2.80 \times 10^{-5}$ | 1.44 | 1.74 | $3.42 \times 10^{-1}$ |

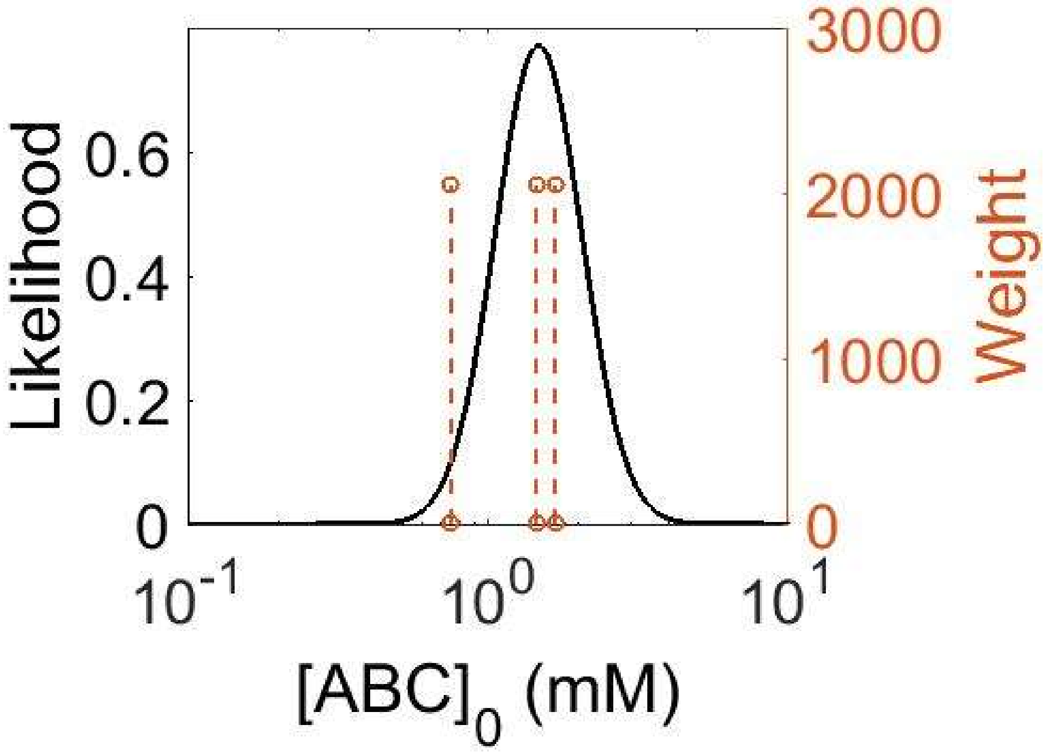


**Figure SF.10.11.1.3.1.** The estimated probability distribution for the ABC concentration. The value and weight of the literature values used to define the distribution are indicated by an orange dashed line. The x axis is plotted on a log-scale.

### S.10.12. Reaction 33: $PGI_2 \rightleftharpoons exPGI_2$

$PGI_2$ is relocated from the intracellular compartment to the extracellular compartment via the ATP-binding cassette (ABC) transporter.

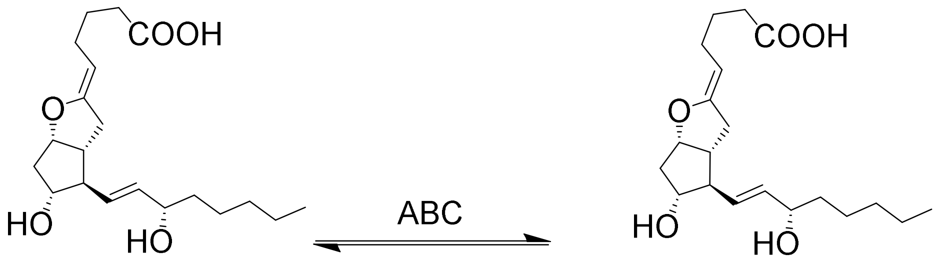


$PGI_2$ (Intracellular Compartment) — $exPGI_2$ (Extracellular Compartment)

**Figure SF.10.12.** The transport of prostaglandin $I_2$ ($PGI_2$) from the intracellular compartment to the extracellular compartment by ATP Binding Cassette protein (ABC) (Reaction 33).

**SEq.10.12.** Reaction rate law for Reaction 33.

$$v_{33} = [ABC] \cdot k_{cat} \cdot \frac{{PGI_2}/{K_m} \cdot \left(1 - \frac{exPGI_2}{PGI_2 \cdot e^{-0.5 \cdot \left(G + R \cdot T \cdot \ln\left(\frac{ATP}{ADP}\right)\right)/R \cdot T}}\right)}{1 + \frac{PGI_2}{K_m} + \frac{exPGI_2}{K_m} + ABC\_CI}$$

## S.10.12.1. Reaction parameters

### S.10.12.1.1. Parameter: ABC $k_{cat}$

Parameter values for the ABC $k_{cat}$ of Reaction 33 were obtained from the literature and summarised in Table ST.10.12.1.1.1. The log-normal distribution properties for the ABC $k_{cat}$ of Reaction 33 are shown in Table ST.10.12.1.1.2 and plotted in Figure SF.10.12.1.1.1.

**Table ST.10.12.1.1.1.** Literature information used to design the ABC $k_{cat}$ parameter distribution. Each value was assigned a weight using the protocol described in Section 2.6.2. The type of error was assigned as 0 if it was additive and 1 if it was multiplicative.

| Value ($min^{-1}$) | Error ($min^{-1}$) | Experimental details | | | | | | Weight | Type of error | Reference |
|---|---|---|---|---|---|---|---|---|---|---|
| | | Species | Expression Vector | Enzyme | pH | Temperature (°C) | Other | | | |
| 3.50 | NaN | Bacteria | E. coli. | Glucose transporter | Unknown | Unknown | Bionumbers calculation | **8** | 0 | (Waygood and Steeves, 1980) |
| 3.30 $x10^{-1}$ | NaN | Bacteria | E. coli. | Lactose transporter | Unknown | Unknown | Bionumbers calculation | **8** | 0 | (Naftalin et al., 2007) |
| 8.30 $x10^{-1}$ | NaN | Bacteria | E. coli. | Lactose transporter | Unknown | Unknown | Bionumbers calculation | **8** | 0 | (Naftalin et al., 2007) |
| 3.28 | NaN | Yeast | Saccharomyces cerevisiae | HXT7 transporter | Unknown | Unknown | Bionumbers calculation | **8** | 0 | (Ye et al., 2001) |
| 1.13 $x10^{1}$ | | Primate | Baculovirus | MRP2 | 7 | 37 | | 512 | | (Yasunaga et al., 2008) |

**Table ST.10.12.1.1.2.** The log-normal distribution properties of the ABC $k_{cat}$ distribution. These values were calculated using the functions described in Section 2.6.2.

| Mode ($min^{-1}$) | Confidence Interval | Location parameter (μ) | Scale parameter (σ) |
|---|---|---|---|
| 1.13 $x10^{1}$ | 1.00 $x10^{1}$ | 3.22 | $8.9110^{-1}$ |

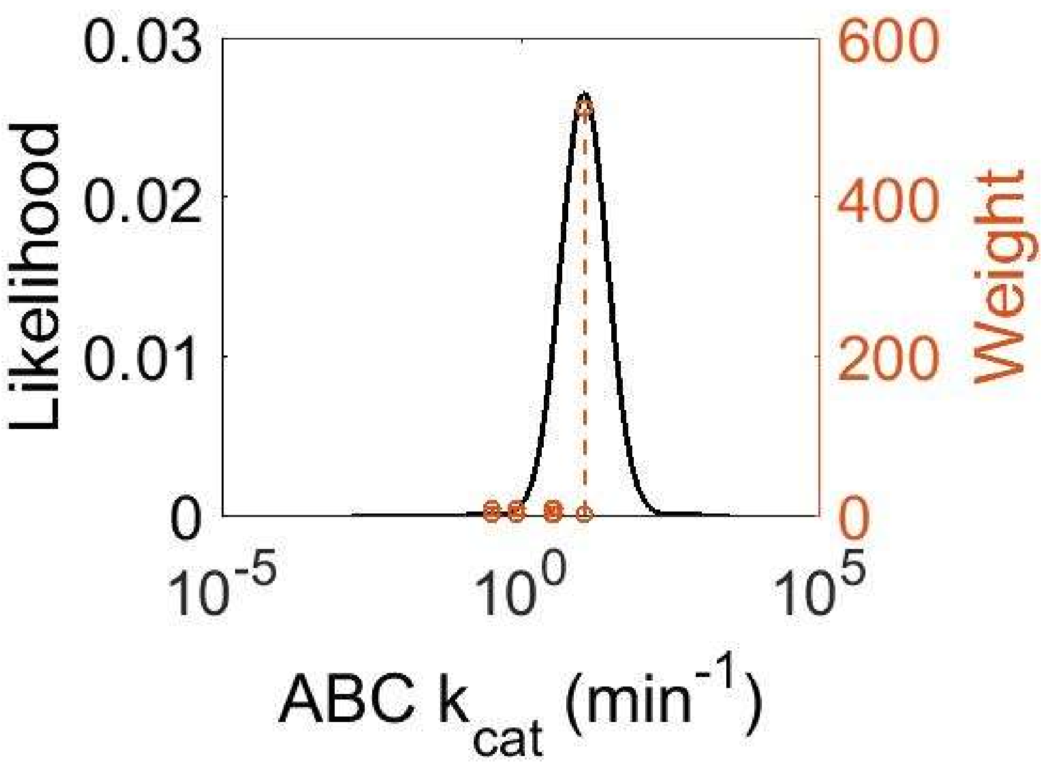


**Figure SF.10.12.1.1.1.** The estimated probability distribution for ABC $k_{cat}$. The value and weight of the literature values used to define the distribution are indicated by an orange dashed line. The x axis is plotted on a log-scale.

**S.10.12.1.2. Parameter: ABC $K_{ms}$**

Parameter values for the ABC $K_{ms}$ of Reaction 33 were obtained from the literature and summarised in Table ST.10.12.1.2.1. The log-normal distribution properties for the ABC $K_{ms}$ of Reaction 33 are shown in Table ST.10.12.1.2.2 and plotted in Figure SF.10.12.1.2.1.

**Table ST.10.12.1.2.1.** Literature information used to design the ABC $K_{ms}$ parameter distribution. Each value was assigned a weight using the protocol described in Section 2.6.2. The type of error was assigned as 0 if it was additive and 1 if it was multiplicative.

| Value (mM) | Error (mM) | Experimental details | | | | | | Weight | Type of error | Reference |
|---|---|---|---|---|---|---|---|---|---|---|
| | | Species | Expression Vector | Enzyme | pH | Temperature (°C) | Other | | | |
| $1.09 \times 10^{-2}$ | $4.10 \times 10^{-3}$ | Human | Primate | MRP2 | 7 | 37 | | **512** | 0 | (Yasunaga et al., 2008) |
| $3.66 \times 10^{-5}$ | $3.80 \times 10^{-6}$ | Unknown | H69AR plasma membrane | MRP1 | 7.4 | 37 | | **128** | 0 | (Mao et al., 2000) |
| $5.30 \times 10^{-3}$ | $2.60 \times 10^{-3}$ | Human | HEK293 cells | MRP3 | 7.4 | 37 | | **128** | 0 | (Zeng et al., 2000) |
| $1.95 \times 10^{-1}$ | $6.12 \times 10^{-2}$ | Human | Primate | MRP2 | 7 | 37 | | **512** | 0 | (Yasunaga et al., 2008) |
| $1.10 \times 10^{-1}$ | $3.91 \times 10^{-2}$ | Human | Primate | MRP2 | 7 | 37 | | **512** | 0 | (Yasunaga et al., 2008) |
| $2.07 \times 10^{-2}$ | NaN | Human | HepG2 hepatoma | ABCB1 | 7.4 | 37 | | **2048** | 0 | (Fong et al., 2007) |

**Table ST.10.12.1.2.2.** The log-normal distribution properties of the ABC $K_{ms}$ distribution. These values were calculated using the functions described in Section 2.6.2.

| Mode (mM) | Confidence Interval | Location parameter (μ) | Scale parameter (σ) |
|---|---|---|---|
| $2.10 \times 10^{-2}$ | $2.37 \times 10^{1}$ | -2.60 | 1.12 |

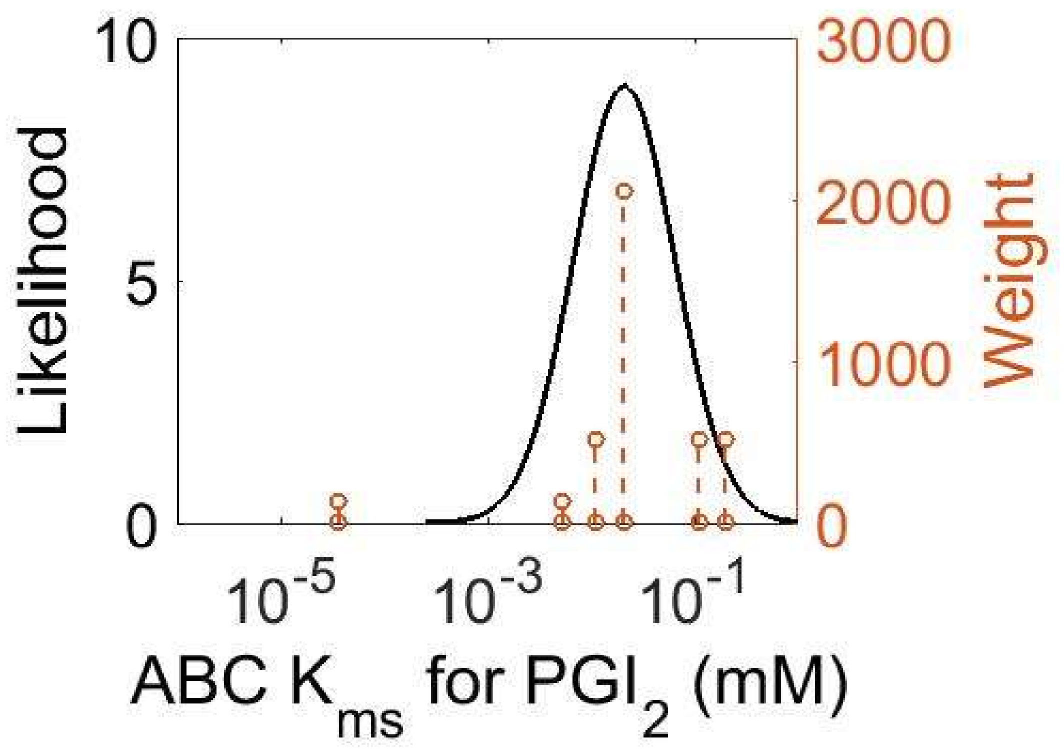


**Figure SF.10.12.1.2.1.** The estimated probability distribution for ABC $K_{ms}$. The value and weight of the literature values used to define the distribution are indicated by an orange dashed line. The x axis is plotted on a log-scale.

**S.10.12.1.3. Parameter: ABC concentration**

Parameter values for the ABC concentration of Reaction 33 were obtained from the literature and summarised in Table ST.10.12.1.3.1. The log-normal distribution properties for the ABC concentration of Reaction 33 are shown in Table ST.10.12.1.3.2 and plotted in Figure SF.10.12.1.3.1. To convert the enzyme concentration from ppm to mM, **Equation S.6.2** was used.

**Table ST.10.12.1.3.1.** Literature information used to design the ABC concentration parameter distribution. Each value was assigned a weight using the protocol described in Section 2.6.2. The type of error was assigned as 0 if it was additive and 1 if it was multiplicative.

| Value (mM) | Error (ppm) | Experimental details | | | | | | Weight | Type of error | Reference |
|---|---|---|---|---|---|---|---|---|---|---|
| | | Species | Expression Vector | Enzyme | pH | Temperature (°C) | Other | | | |
| 5.15 | NaN | Human | Stomach | ABC | 7.5 | 37 | | 2048 | 0 | (Wilhelm et al., 2014) |
| 5.94 | NaN | Human | Lung | ABC | 7.5 | 37 | | 2048 | 0 | (Kim et al., 2014) |
| 2.66 | NaN | Human | Gut | ABC | 7.5 | 37 | | 2048 | 0 | (Kim et al., 2014) |

**Table ST.10.12.1.3.2.** The log-normal distribution properties of the ABC concentration distribution. These values were calculated using the functions described in Section 2.6.2.

| Mode (mM) | Confidence Interval | Location parameter (μ) | Scale parameter (σ) |
|---|---|---|---|
| 5.06 | 1.44 | 1.74 | $3.42 \times 10^{-1}$ |

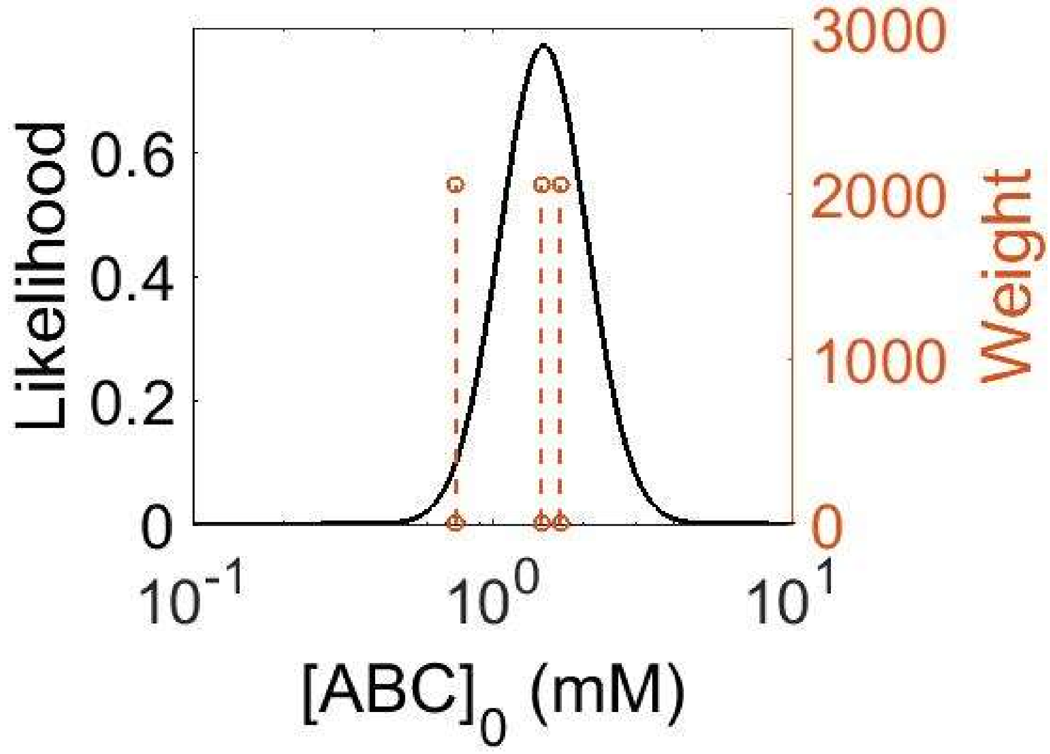


**Figure SF.10.12.1.3.1.** The estimated probability distribution for the ABC concentration. The value and weight of the literature values used to define the distribution are indicated by an orange dashed line. The x axis is plotted on a log-scale.

**S.10.13. Reaction 34: $PGH_2 \rightleftharpoons exPGH_2$**

$PGH_2$ is relocated from the intracellular compartment to the extracellular compartment via the ATP-binding cassette (ABC) transporter.

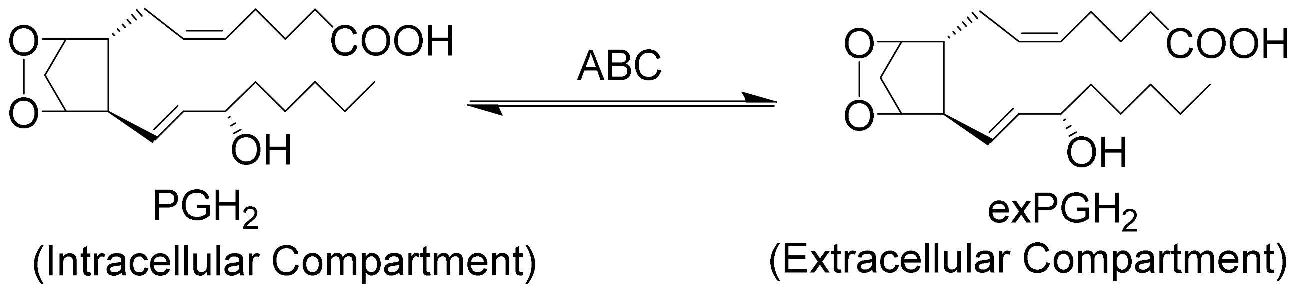


**Figure SF.10.13.** The transport of prostaglandin $H_2$ ($PGH_2$) from the intracellular compartment to the extracellular compartment by ATP Binding Cassette protein (ABC) (Reaction 34).

**SEq.10.13.** Reaction rate law for Reaction 34.

$$\boldsymbol{v_{34}} = [\boldsymbol{ABC}] \cdot \boldsymbol{k_{cat}} \cdot \frac{\mathbf{PGH_2}/\boldsymbol{K_m} \cdot \left(\mathbf{1} - \frac{\mathbf{exPGH_2}}{\mathbf{PGH_2} \cdot \boldsymbol{e}^{-0.5\cdot\left(\boldsymbol{G}+\boldsymbol{R}\cdot\boldsymbol{T}\cdot\ln\left(\frac{\boldsymbol{ATP}}{\boldsymbol{ADP}}\right)\right)/\boldsymbol{R}\cdot\boldsymbol{T}}}\right)}{\mathbf{1} + \frac{\mathbf{PGH_2}}{\boldsymbol{K_m}} + \frac{\mathbf{exPGH_2}}{\boldsymbol{K_m}} + \mathbf{ABC\_CI}}$$

### S.7.1.1. Reaction parameters

**Parameter: ABC $k_{cat}$**

Parameter values for the ABC $k_{cat}$ of Reaction 34 were obtained from the literature and summarised in Table ST.10.13.1.1.1. The log-normal distribution properties for the ABC $k_{cat}$ of Reaction 34 are shown in Table ST.10.13.1.1.2 and plotted in Figure SF.10.13.1.1.1.

**Table ST.10.13.1.1.1.** Literature information used to design the ABC $k_{cat}$ parameter distribution. Each value was assigned a weight using the protocol described in Section 2.6.2. The type of error was assigned as 0 if it was additive and 1 if it was multiplicative.

| Value ($min^{-1}$) | Error ($min^{-1}$) | Experimental details | | | | | | Weight | Type of error | Reference |
|---|---|---|---|---|---|---|---|---|---|---|
| | | Species | Expression Vector | Enzyme | pH | Temperature (°C) | Other | | | |
| 3.50 | NaN | Bacteria | E. coli. | Glucose transporter | Unknown | Unknown | Bionumbers calculation | 8 | 0 | (Waygood and Steeves, 1980) |
| $3.30 \times 10^{-1}$ | NaN | Bacteria | E. coli. | Lactose transporter | Unknown | Unknown | Bionumbers calculation | 8 | 0 | (Naftalin et al., 2007) |
| $8.30 \times 10^{-1}$ | NaN | Bacteria | E. coli. | Lactose transporter | Unknown | Unknown | Bionumbers calculation | 8 | 0 | (Naftalin et al., 2007) |
| 3.28 | NaN | Yeast | Saccharomyces cerevisiae | HXT7 transporter | Unknown | Unknown | Bionumbers calculation | 8 | 0 | (Ye et al., 2001) |
| $1.13 \times 10^{1}$ | | Primate | Baculovirus | MRP2 | 7 | 37 | | 512 | | (Yasunaga et al., 2008) |

**Table ST.10.13.1.1.2.** The log-normal distribution properties of the ABC $k_{cat}$ distribution. These values were calculated using the functions described in Section 2.6.2.

| Mode ($min^{-1}$) | Confidence Interval | Location parameter (μ) | Scale parameter (σ) |
|---|---|---|---|
| 1.13 $x10^{1}$ | 1.00 $x10^{1}$ | 3.22 | 8.91 $x10^{-1}$ |

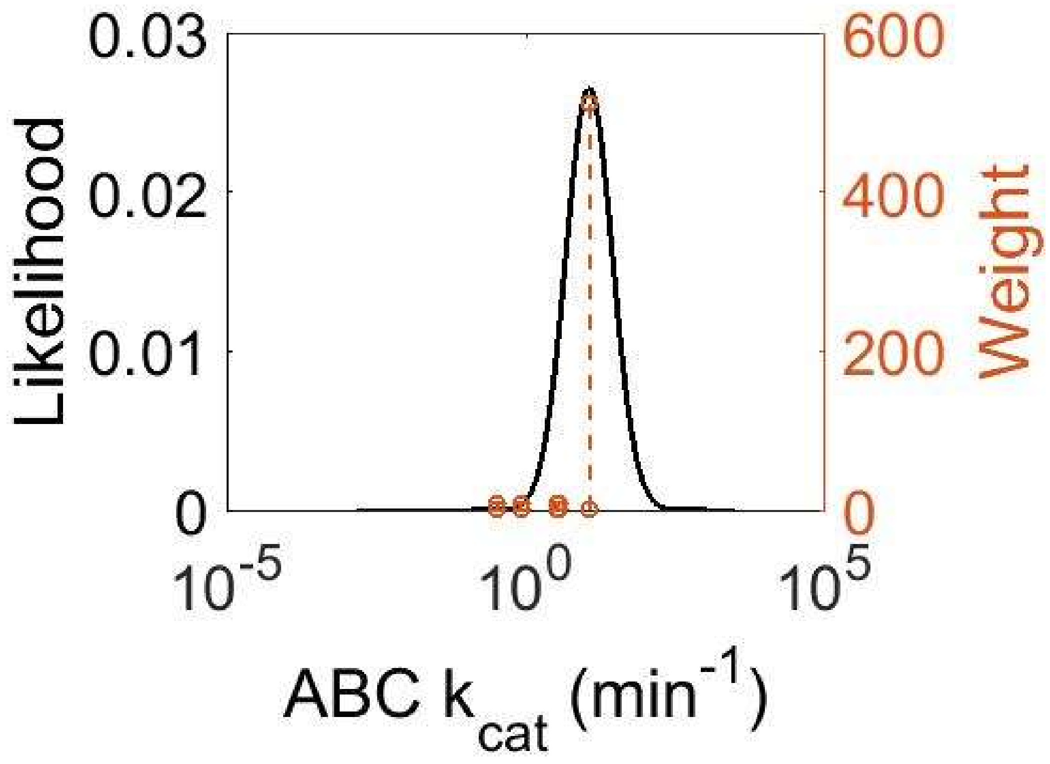


**Figure SF.10.13.1.1.1.** The estimated probability distribution for ABC $k_{cat}$. The value and weight of the literature values used to define the distribution are indicated by an orange dashed line. The x axis is plotted on a log-scale.

**S.10.13.1.2. Parameter: ABC $K_{ms}$**

In this model numerous simplifications have been made and as a result, intermediate metabolites, such as $PGH_2$, occur in higher concentrations than are found in reality. To prevent a large percentage of $PGH_2$ being exported before it is metabolised, the $K_{ms}$ of the ABC transporter has been increased (mode= 25 mM, CIF=10, $\mu$ = 4.01, $\sigma$ = 0.89) (Table ST.10.13.1.2.1.). This is plotted in Figure SF.10.13.1.2.1.

**Table ST.10.13.1.2.1.** The log-normal distribution properties of the ABC $K_{ms}$ distribution. These values were calculated using the functions described in Section 2.6.2.

| Mode (mM) | Confidence Interval | Location parameter ($\mu$) | Scale parameter ($\sigma$) |
|---|---|---|---|
| 25 | 10 | 4.01 | $8.9010^{-1}$ |

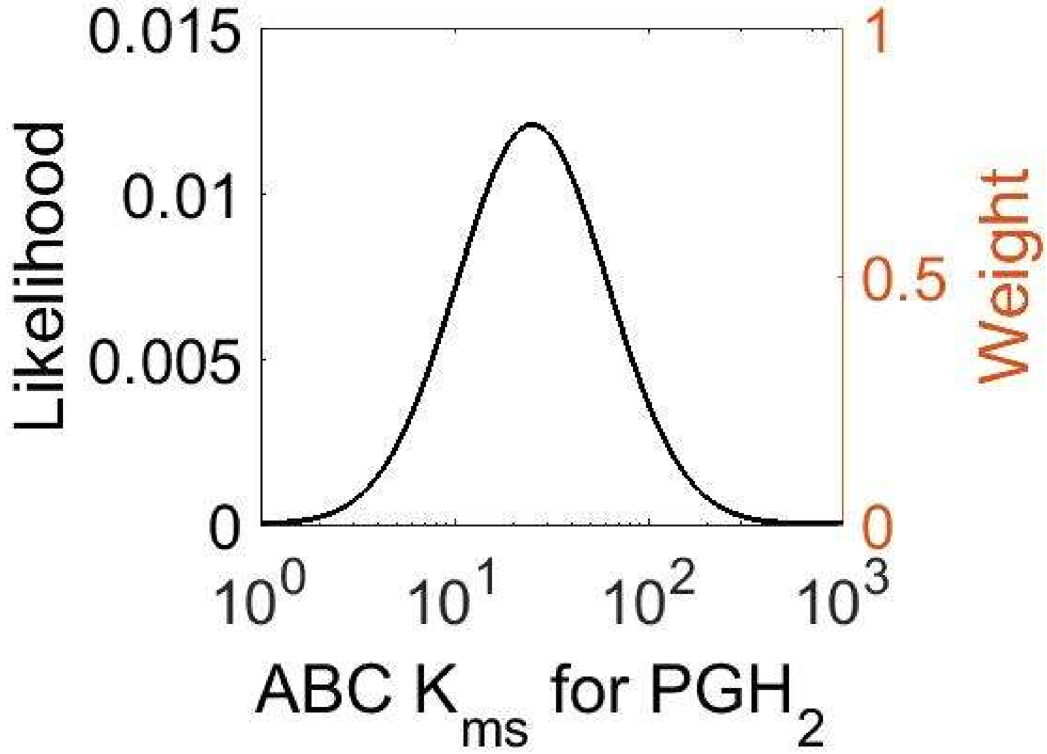


**Figure SF.10.13.1.2.1.** The estimated probability distribution for ABC $K_{ms}$. The value and weight of the literature values used to define the distribution are indicated by an orange dashed line. The x axis is plotted on a log-scale.

### S.10.13.1.3. Parameter: ABC concentration

Parameter values for the ABC concentration of Reaction 34 were obtained from the literature and summarised in Table ST.10.13.1.3.1. The log-normal distribution properties for the ABC concentration of Reaction 34 are shown in Table ST.10.13.1.3.2 and plotted in Figure SF.10.13.1.3.1. To convert the enzyme concentration from ppm to mM, **Equation S.6.2** was used.

**Table ST.10.13.1.3.1.** Literature information used to design the ABC concentration parameter distribution. Each value was assigned a weight using the protocol described in Section 2.6.2. The type of error was assigned as 0 if it was additive and 1 if it was multiplicative.

| Value (ppm) | Error (ppm) | Experimental details | | | | | | Weight | Type of error | Reference |
|---|---|---|---|---|---|---|---|---|---|---|
| | | Species | Expression Vector | Enzyme | pH | Temperature (°C) | Other | | | |
| 5.15 | NaN | Human | Stomach | ABC | 7.5 | 37 | | 2048 | 0 | (Wilhelm et al., 2014) |
| 5.94 | NaN | Human | Lung | ABC | 7.5 | 37 | | 2048 | 0 | (Kim et al., 2014) |
| 2.66 | NaN | Human | Gut | ABC | 7.5 | 37 | | 2048 | 0 | (Kim et al., 2014) |

**Table ST.10.13.1.3.2.** The log-normal distribution properties of the ABC concentration distribution. These values were calculated using the functions described in Section 2.6.2.

| Mode (ppm) | Mode (mM) | Confidence Interval | Location parameter (μ) | Scale parameter (σ) |
|---|---|---|---|---|
| 5.06 | $2.80 \times 10^{-5}$ | 1.44 | 1.74 | $3.4210^{-1}$ |

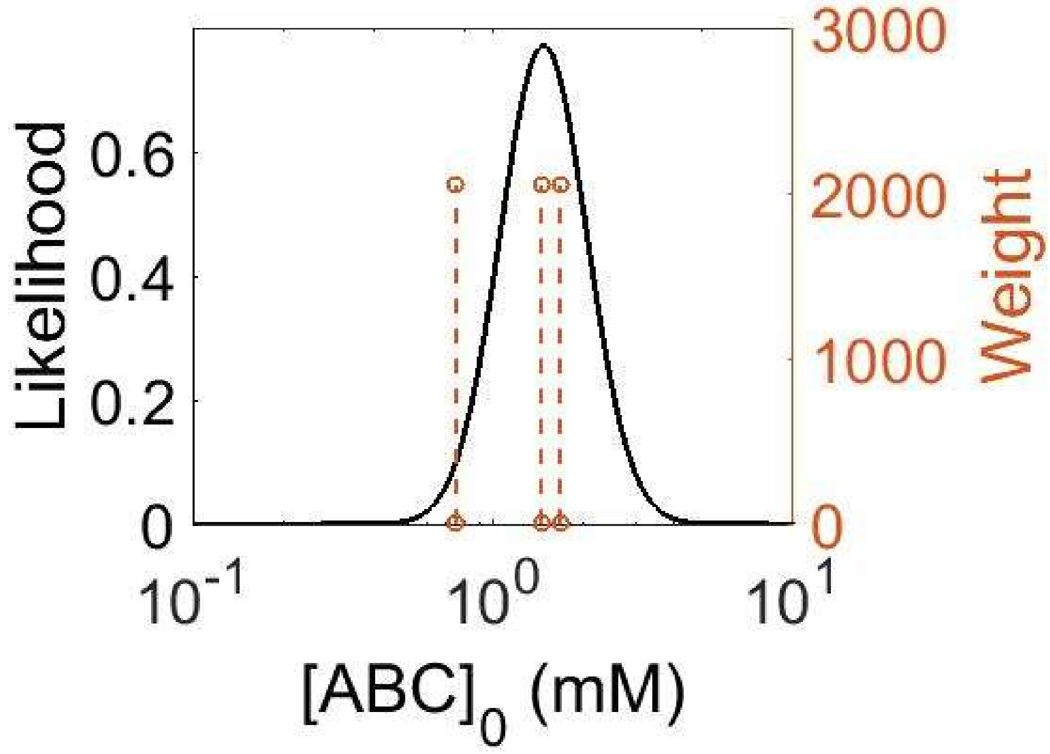


**Figure SF.10.13.1.3.1.** The estimated probability distribution for the ABC concentration. The value and weight of the literature values used to define the distribution are indicated by an orange dashed line. The x axis is plotted on a log-scale.

**S.10.14. Reaction 35: $PGD_2 \rightleftharpoons exPGD_2$**

$PGD_2$ is relocated from the intracellular compartment to the extracellular compartment via the ATP-binding cassette (ABC) transporter.

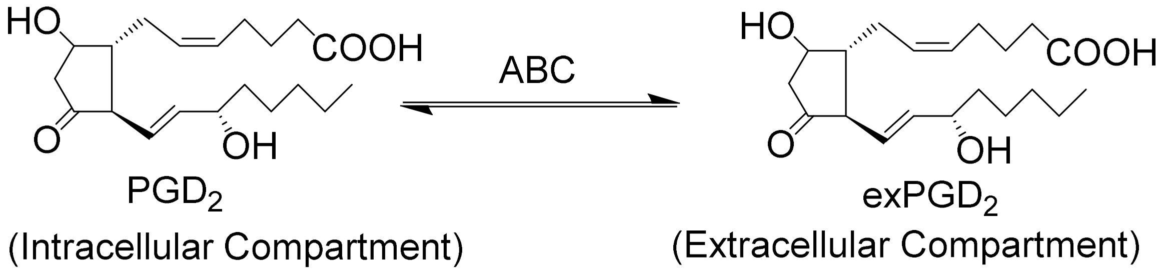


**Figure SF.10.14.** The transport of prostaglandin $D_2$ ($PGD_2$) from the intracellular compartment to the extracellular compartment by ATP Binding Cassette protein (ABC) (Reaction 35).

**SEq.10.14.** Reaction rate law for Reaction 35.

$$v_{35} = [ABC] \cdot k_{cat} \cdot \frac{\mathrm{PGD_2}/K_m \cdot \left(1 - \frac{\mathrm{exPGD_2}}{\mathrm{PGD_2} \cdot e^{-0.5 \cdot \left(G + R \cdot T \cdot \ln\left(\frac{ATP}{ADP}\right)\right)/R \cdot T}}\right)}{1 + \frac{\mathrm{PGD_2}}{K_m} + \frac{\mathrm{exPGD_2}}{K_m} + \mathrm{ABC\_CI}}$$

### S.10.14.1. Reaction parameters

#### S.10.14.1.1. Parameter: ABC $k_{cat}$

Parameter values for the ABC $k_{cat}$ of Reaction 35 were obtained from the literature and summarised in Table ST.10.14.1.1.1. The log-normal distribution properties for the ABC $k_{cat}$ of Reaction 35 are shown in Table ST.10.14.1.1.2 and plotted in Figure SF.10.14.1.1.1.

**Table ST.10.14.1.1.1.** Literature information used to design the ABC $k_{cat}$ parameter distribution. Each value was assigned a weight using the protocol described in Section 2.6.2. The type of error was assigned as 0 if it was additive and 1 if it was multiplicative.

| Value ($min^{-1}$) | Error ($min^{-1}$) | Experimental details | | | | | | Weight | Type of error | Reference |
|---|---|---|---|---|---|---|---|---|---|---|
| | | Species | Expression Vector | Enzyme | pH | Temperature (°C) | Other | | | |
| 3.50 | NaN | Bacteria | E. coli. | Glucose transporter | Unknown | Unknown | Bionumbers calculation | 8 | 0 | (Waygood and Steeves, 1980) |
| $3.30 \times 10^{-1}$ | NaN | Bacteria | E. coli. | Lactose transporter | Unknown | Unknown | Bionumbers calculation | 8 | 0 | (Naftalin et al., 2007) |
| $8.30 \times 10^{-1}$ | NaN | Bacteria | E. coli. | Lactose transporter | Unknown | Unknown | Bionumbers calculation | 8 | 0 | (Naftalin et al., 2007) |
| 3.28 | NaN | Yeast | Saccharomyces cerevisiae | HXT7 transporter | Unknown | Unknown | Bionumbers calculation | 8 | 0 | (Ye et al., 2001) |
| $1.13 \times 10^{1}$ | | Primate | Baculovirus | MRP2 | 7 | 37 | | 512 | | (Yasunaga et al., 2008) |

**Table ST.10.14.1.1.2.** The log-normal distribution properties of the ABC $k_{cat}$ distribution. These values were calculated using the functions described in Section 2.6.2.

| **Mode ($min^{-1}$)** | **Confidence Interval** | **Location parameter (μ)** | **Scale parameter (σ)** |
|---|---|---|---|
| $1.13 \times 10^{1}$ | $1.00 \times 10^{1}$ | 3.22 | $8.9110^{-1}$ |

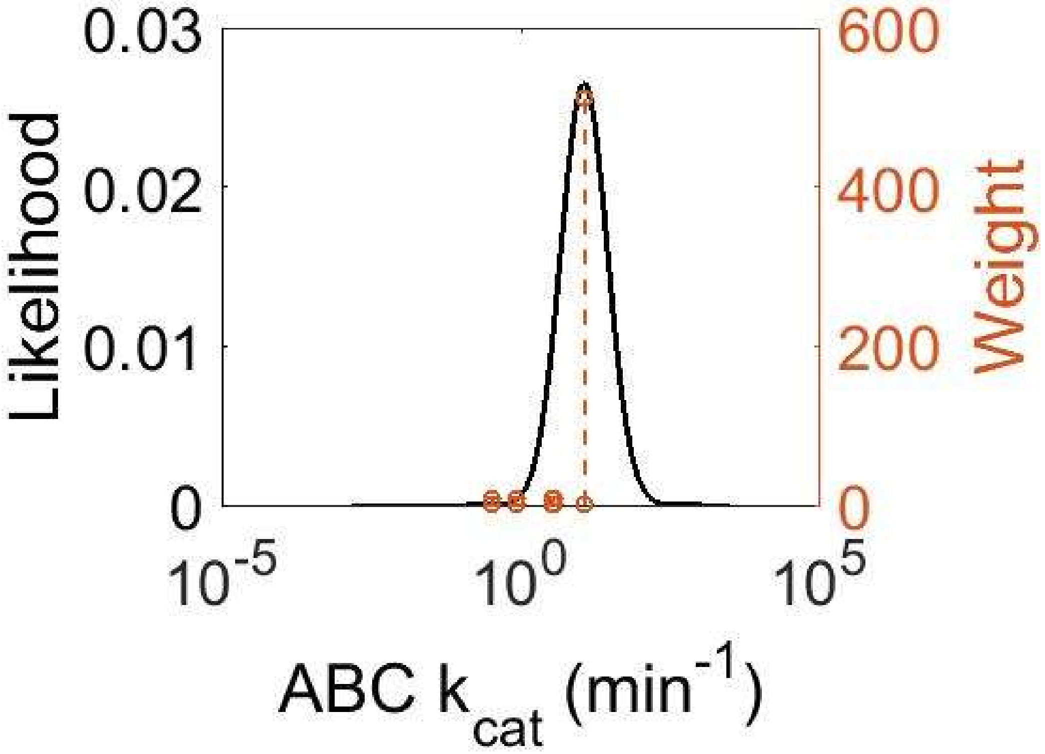


**Figure SF.10.14.1.1.1.** The estimated probability distribution for ABC $k_{cat}$. The value and weight of the literature values used to define the distribution are indicated by an orange dashed line. The x axis is plotted on a log-scale.

### S.10.14.1.2. Parameter: ABC $K_{ms}$

Parameter values for the ABC $K_{ms}$ of Reaction 35 were obtained from the literature and summarised in Table ST.10.14.1.2.1. The log-normal distribution properties for the ABC $K_{ms}$ of Reaction 35 are shown in Table ST.10.14.1.2.2 and plotted in Figure SF.10.14.1.2.1.

**Table ST.10.14.1.2.1.** Literature information used to design the ABC $K_{ms}$ parameter distribution. Each value was assigned a weight using the protocol described in Section 2.6.2. The type of error was assigned as 0 if it was additive and 1 if it was multiplicative.

| Value (mM) | Error (mM) | Experimental details | | | | | | Weight | Type of error | Reference |
|---|---|---|---|---|---|---|---|---|---|---|
| | | Species | Expression Vector | Enzyme | pH | Temperature (°C) | Other | | | |
| $1.09 \times 10^{-2}$ | $4.10 \times 10^{-3}$ | Human | Primate | MRP2 | 7 | 37 | | **512** | 0 | (Yasunaga et al., 2008) |
| $3.66 \times 10^{-5}$ | $3.80 \times 10^{-6}$ | Unknown | H69AR plasma membrane | MRP1 | 7.4 | 37 | | **128** | 0 | (Mao et al., 2000) |
| $5.30 \times 10^{-3}$ | $2.60 \times 10^{-3}$ | Human | HEK293 cells | MRP3 | 7.4 | 37 | | **128** | 0 | (Zeng et al., 2000) |
| $1.95 \times 10^{-1}$ | $6.12 \times 10^{-2}$ | Human | Primate | MRP2 | 7 | 37 | | **512** | 0 | (Yasunaga et al., 2008) |
| $1.10 \times 10^{-1}$ | $3.91 \times 10^{-2}$ | Human | Primate | MRP2 | 7 | 37 | | **512** | 0 | (Yasunaga et al., 2008) |
| $2.07 \times 10^{-2}$ | NaN | Human | HepG2 hepatoma | ABCB1 | 7.4 | 37 | | **2048** | 0 | (Fong et al., 2007) |

**Table ST.10.14.1.2.2.** The log-normal distribution properties of the ABC $K_{ms}$ distribution. These values were calculated using the functions described in Section 2.6.2.

| Mode (mM) | Confidence Interval | Location parameter (μ) | Scale parameter (σ) |
|---|---|---|---|
| 2.10 $\times 10^{-2}$ | 2.37 $\times 10^{1}$ | -2.60 | 1.12 |

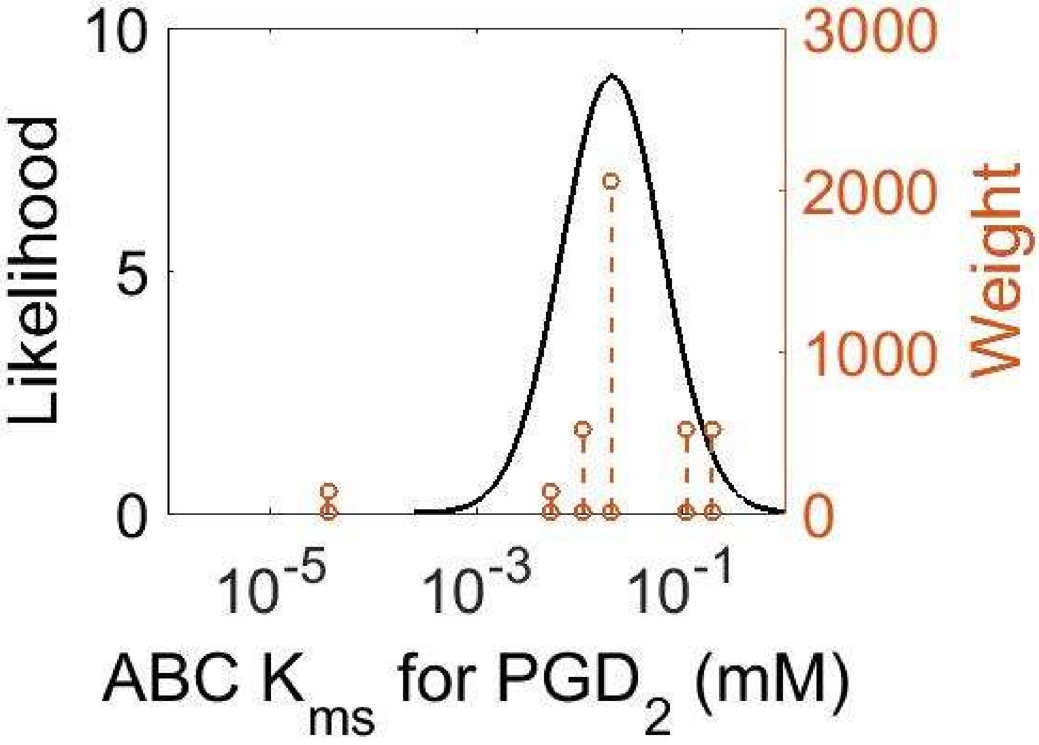


**Figure SF.10.14.1.2.1.** The estimated probability distribution for ABC $K_{ms}$. The value and weight of the literature values used to define the distribution are indicated by an orange dashed line. The x axis is plotted on a log-scale.

### S.10.14.1.3. Parameter: ABC concentration

Parameter values for the ABC concentration of Reaction 35 were obtained from the literature and summarised in Table ST.10.14.1.3.1. The log-normal distribution properties for the ABC concentration of Reaction 35 are shown in Table ST.10.14.1.3.2 and plotted in Figure SF.10.14.1.3.1. To convert the enzyme concentration from ppm to mM, **Equation S.6.2** was used.

**Table ST.10.14.1.3.1.** Literature information used to design the ABC concentration parameter distribution. Each value was assigned a weight using the protocol described in Section 2.6.2. The type of error was assigned as 0 if it was additive and 1 if it was multiplicative.

| Value (ppm) | Error (ppm) | Experimental details | | | | | | Weight | Type of error | Reference |
|---|---|---|---|---|---|---|---|---|---|---|
| | | Species | Expression Vector | Enzyme | pH | Temperature (°C) | Other | | | |
| 5.15 | NaN | Human | Stomach | ABC | 7.5 | 37 | | 2048 | 0 | (Wilhelm et al., 2014) |
| 5.94 | NaN | Human | Lung | ABC | 7.5 | 37 | | 2048 | 0 | (Kim et al., 2014) |
| 2.66 | NaN | Human | Gut | ABC | 7.5 | 37 | | 2048 | 0 | (Kim et al., 2014) |

**Table ST.10.14.1.3.2.** The log-normal distribution properties of the ABC concentration distribution. These values were calculated using the functions described in Section 2.6.2.

| Mode (ppm) | Mode (mM) | Confidence Interval | Location parameter (μ) | Scale parameter (σ) |
|---|---|---|---|---|
| 5.06 | $2.80 \times 10^{-5}$ | 1.44 | 1.74 | $3.42 \times 10^{-1}$ |

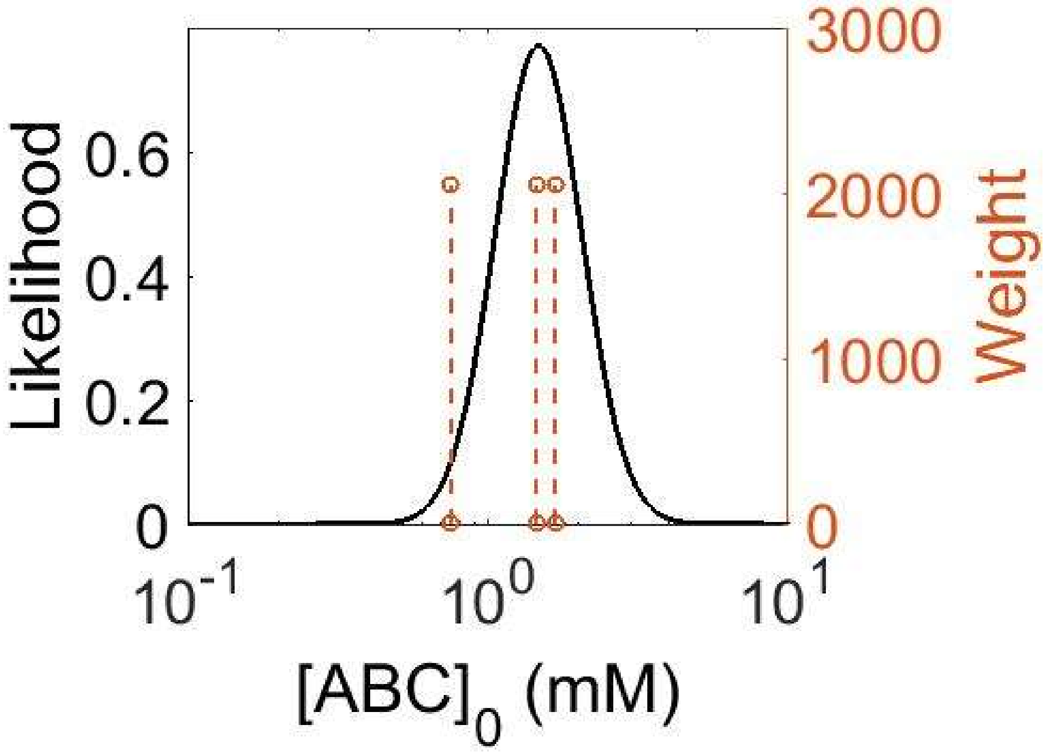


**Figure SF.10.14.1.3.1.** The estimated probability distribution for the ABC concentration. The value and weight of the literature values used to define the distribution are indicated by an orange dashed line. The x axis is plotted on a log-scale.

**S.10.15. Reaction 36: $PGJ_2 \rightleftharpoons exPGJ_2$**

$PGJ_2$ is relocated from the intracellular compartment to the extracellular compartment via the ATP-binding cassette (ABC) transporter.

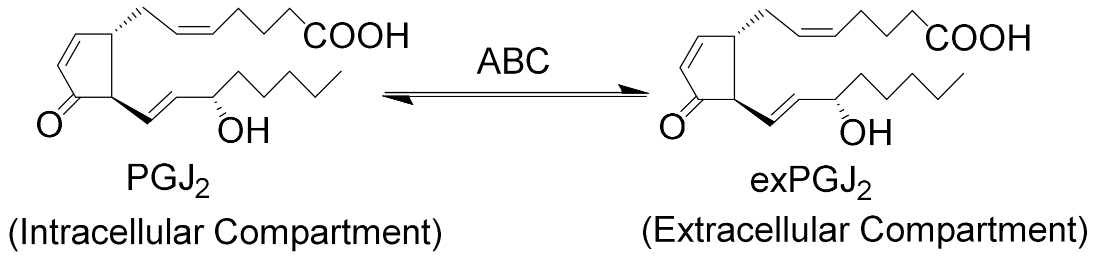


**Figure SF.10.15.** The transport of prostaglandin $J_2$ ($PGJ_2$) from the intracellular compartment to the extracellular compartment by ATP Binding Cassette protein (ABC) (Reaction 36).

**SEq.10.15.** Reaction rate law for Reaction 36.

$$v_{36} = [ABC] \cdot k_{cat} \cdot \frac{{}^{\mathrm{PGJ_2}}/_{K_m} \cdot \left( 1 - \frac{\mathrm{exPGJ_2}}{\mathrm{PGJ_2} \cdot e^{-0.5 \cdot \left(G + R \cdot T \cdot \ln\left(\frac{ATP}{ADP}\right)\right) / R \cdot T}} \right)}{1 + \frac{\mathrm{PGJ_2}}{K_m} + \frac{\mathrm{exPGJ_2}}{K_m} + \mathrm{ABC\_CI}}$$

### S.10.15.1 Reaction parameters

#### S.10.15.1.1 Parameter: ABC $k_{cat}$

Parameter values for the ABC $k_{cat}$ of Reaction 36 were obtained from the literature and summarised in Table ST.10.15.1.1.1. The log-normal distribution properties for the ABC $k_{cat}$ of Reaction 36 are shown in Table ST.10.15.1.1.2 and plotted in Figure SF.10.15.1.1.1.

**Table ST.10.15.1.1.1.** Literature information used to design the ABC $k_{cat}$ parameter distribution. Each value was assigned a weight using the protocol described in Section 2.6.2. The type of error was assigned as 0 if it was additive and 1 if it was multiplicative.

| Value ($min^{-1}$) | Error ($min^{-1}$) | Experimental details | | | | | | Weight | Type of error | Reference |
|---|---|---|---|---|---|---|---|---|---|---|
| | | Species | Expression Vector | Enzyme | pH | Temperature (°C) | Other | | | |
| 3.50 | NaN | Bacteria | E. coli. | Glucose transporter | Unknown | Unknown | Bionumbers calculation | **8** | 0 | (Waygood and Steeves, 1980) |
| 3.30 $x10^{-1}$ | NaN | Bacteria | E. coli. | Lactose transporter | Unknown | Unknown | Bionumbers calculation | **8** | 0 | (Naftalin et al., 2007) |
| 8.30 $x10^{-1}$ | NaN | Bacteria | E. coli. | Lactose transporter | Unknown | Unknown | Bionumbers calculation | **8** | 0 | (Naftalin et al., 2007) |
| 3.28 | NaN | Yeast | Saccharomyces cerevisiae | HXT7 transporter | Unknown | Unknown | Bionumbers calculation | **8** | 0 | (Ye et al., 2001) |
| 1.13 $x10^{1}$ | | Primate | Baculovirus | MRP2 | 7 | 37 | | 512 | | (Yasunaga et al., 2008) |

**Table ST.10.15.1.1.2.** The log-normal distribution properties of the ABC $k_{cat}$ distribution. These values were calculated using the functions described in Section 2.6.2.

| Mode ($min^{-1}$) | Confidence Interval | Location parameter ($\mu$) | Scale parameter ($\sigma$) |
|---|---|---|---|
| 1.13 $x10^{1}$ | 1.00 $x10^{1}$ | 3.22 | 8.91 $x10^{-1}$ |

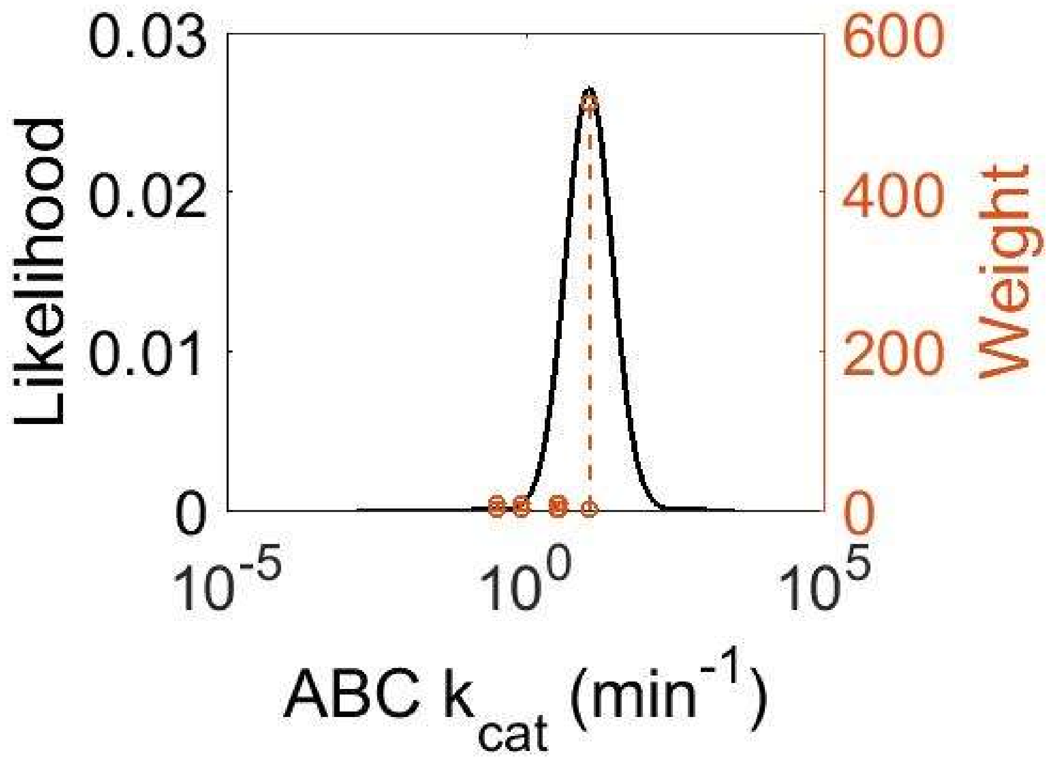


**Figure SF.10.15.1.1.1.** The estimated probability distribution for ABC $k_{cat}$. The value and weight of the literature values used to define the distribution are indicated by an orange dashed line. The x axis is plotted on a log-scale.

### S.10.15.1.2. Parameter: ABC $K_{ms}$

Parameter values for the ABC $K_{ms}$ of Reaction 36 were obtained from the literature and summarised in Table ST.10.15.1.2.1. The log-normal distribution properties for the ABC $K_{ms}$ of Reaction 36 are shown in Table ST.10.15.1.2.2 and plotted in Figure SF.10.15.1.2.1.

**Table ST.10.15.1.2.1.** Literature information used to design the ABC $K_{ms}$ parameter distribution. Each value was assigned a weight using the protocol described in Section 2.6.2. The type of error was assigned as 0 if it was additive and 1 if it was multiplicative.

| Value (mM) | Error (mM) | Experimental details | | | | | | Weight | Type of error | Reference |
|---|---|---|---|---|---|---|---|---|---|---|
| | | Species | Expression Vector | Enzyme | pH | Temperature (°C) | Other | | | |
| $1.09 \times 10^{-2}$ | $4.10 \times 10^{-3}$ | Human | Primate | MRP2 | 7 | 37 | | **512** | 0 | (Yasunaga et al., 2008) |
| $3.66 \times 10^{-5}$ | $3.80 \times 10^{-6}$ | Unknown | H69AR plasma membrane | MRP1 | 7.4 | 37 | | **128** | 0 | (Mao et al., 2000) |
| $5.30 \times 10^{-3}$ | $2.60 \times 10^{-3}$ | Human | HEK293 cells | MRP3 | 7.4 | 37 | | **128** | 0 | (Zeng et al., 2000) |
| $1.95 \times 10^{-1}$ | $6.12 \times 10^{-2}$ | Human | Primate | MRP2 | 7 | 37 | | **512** | 0 | (Yasunaga et al., 2008) |
| $1.10 \times 10^{-1}$ | $3.91 \times 10^{-2}$ | Human | Primate | MRP2 | 7 | 37 | | **512** | 0 | (Yasunaga et al., 2008) |
| $2.07 \times 10^{-2}$ | NaN | Human | HepG2 hepatoma | ABCB1 | 7.4 | 37 | | **2048** | 0 | (Fong et al., 2007) |

**Table ST.10.15.1.2.2.** The log-normal distribution properties of the ABC $K_{ms}$ distribution. These values were calculated using the functions described in Section 2.6.2.

| Mode (mM) | Confidence Interval | Location parameter (μ) | Scale parameter (σ) |
|---|---|---|---|
| $2.10 \times 10^{-2}$ | $2.37 \times 10^{1}$ | -2.60 | 1.12 |

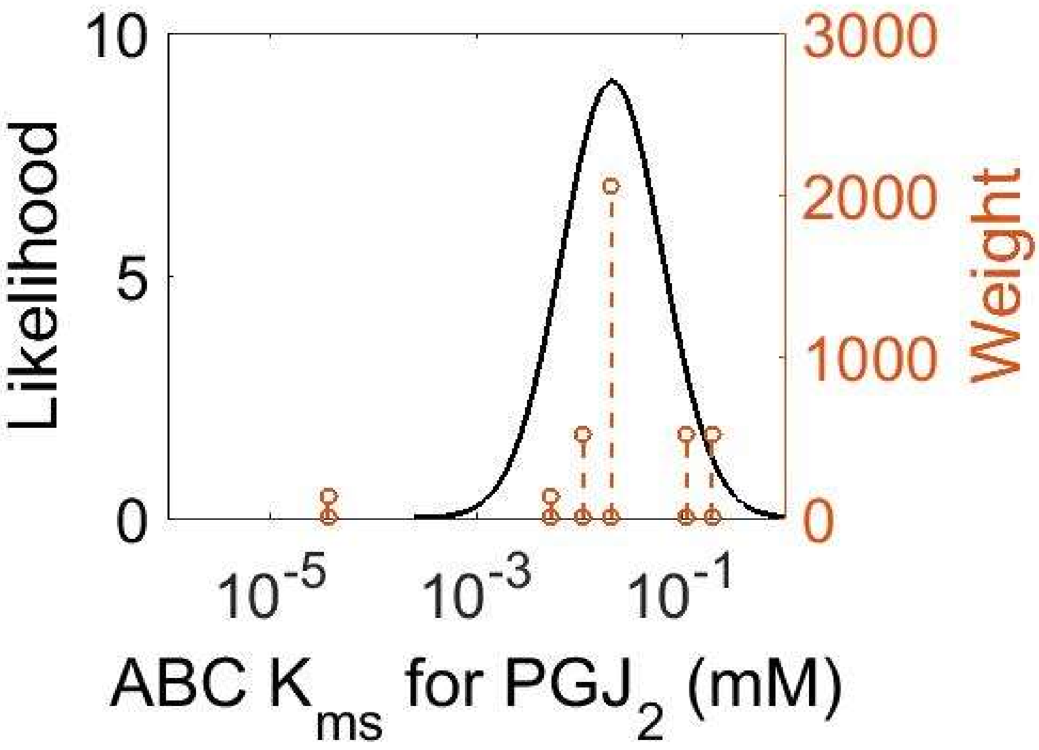


**Figure SF.10.15.1.2.1.** The estimated probability distribution for ABC $K_{ms}$. The value and weight of the literature values used to define the distribution are indicated by an orange dashed line. The x axis is plotted on a log-scale.

**S.10.15.1.3. Parameter: ABC concentration**

Parameter values for the ABC concentration of Reaction 36 were obtained from the literature and summarised in Table ST.10.15.1.3.1. The log-normal distribution properties for the ABC concentration of Reaction 36 are shown in Table ST.10.15.1.3.2 and plotted in Figure SF.10.15.1.3.1. To convert the enzyme concentration from ppm to mM, **Equation S.6.2** was used.

**Table ST.10.15.1.3.1.** Literature information used to design the ABC concentration parameter distribution. Each value was assigned a weight using the protocol described in Section 2.6.2. The type of error was assigned as 0 if it was additive and 1 if it was multiplicative.

| Value (ppm) | Error (ppm) | Experimental details | | | | | | Weight | Type of error | Reference |
|---|---|---|---|---|---|---|---|---|---|---|
| | | Species | Expression Vector | Enzyme | pH | Temperature (°C) | Other | | | |
| 5.15 | NaN | Human | Stomach | ABC | 7.5 | 37 | | 2048 | 0 | (Wilhelm et al., 2014) |
| 5.94 | NaN | Human | Lung | ABC | 7.5 | 37 | | 2048 | 0 | (Kim et al., 2014) |
| 2.66 | NaN | Human | Gut | ABC | 7.5 | 37 | | 2048 | 0 | (Kim et al., 2014) |

**Table ST.10.15.1.3.2.** The log-normal distribution properties of the ABC concentration distribution. These values were calculated using the functions described in Section 2.6.2.

| Mode (ppm) | Mode (mM) | Confidence Interval | Location parameter (μ) | Scale parameter (σ) |
|---|---|---|---|---|
| 5.06 | $2.80 \times 10^{-5}$ | 1.44 | 1.74 | $3.42 \times 10^{-1}$ |

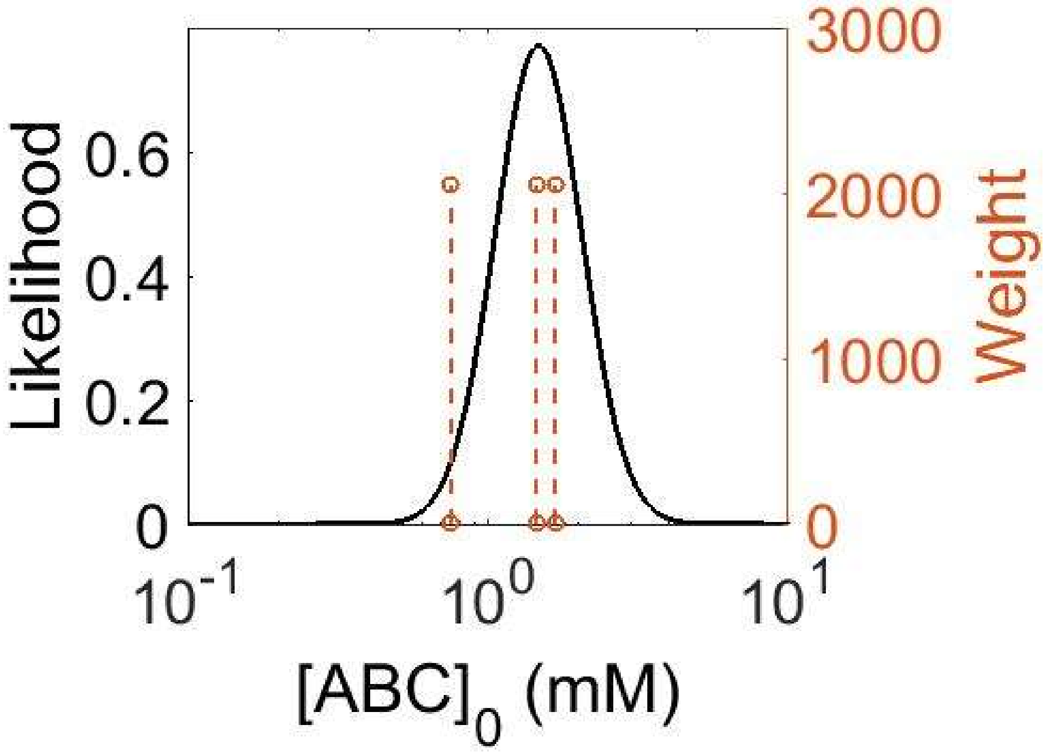


**Figure SF.10.15.1.3.1.** The estimated probability distribution for the ABC concentration. The value and weight of the literature values used to define the distribution are indicated by an orange dashed line. The x axis is plotted on a log-scale.

### S.10.16. Reaction 37: 12-HPETE ⇌ ex12-HPETE

12-HPETE is relocated from the intracellular compartment to the extracellular compartment via the ATP-binding cassette (ABC) transporter.

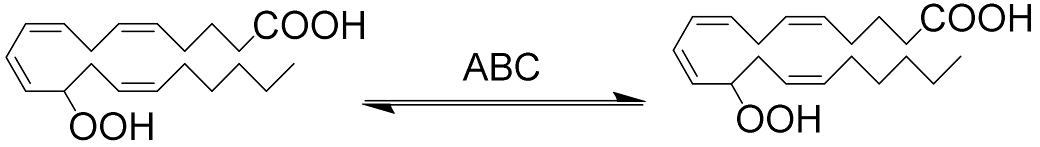


12-HPETE (Intracellular Compartment)    ex12-HPETE (Extracellular Compartment)

**Figure SF.10.16.** The transport of 12-hydroperoxyeicosatetraenoic acid (12-HPETE) from the intracellular compartment to the extracellular compartment by ATP Binding Cassette protein (ABC) (Reaction 37).

### SEq.10.16. Reaction rate law for Reaction 37.

$$v_{37} = [ABC] \cdot k_{cat} \cdot \frac{\text{12−HPETE}/K_m \cdot \left(1 - \frac{\text{ex12−HPETE}}{\text{12−HPETE} \cdot e^{-0.5 \cdot \left(G + R \cdot T \cdot \ln\left(\frac{ATP}{ADP}\right)\right)/R \cdot T}}\right)}{1 + \frac{\text{12−HPETE}}{K_m} + \frac{\text{ex12−HPETE}}{K_m} + \text{ABC\_CI}}$$

## S.10.16.1. Reaction parameters

### S.10.16.1.1. Parameter: ABC $k_{cat}$

Parameter values for the ABC $k_{cat}$ of Reaction 37 were obtained from the literature and summarised in Table ST.10.16.1.1.1. The log-normal distribution properties for the ABC $k_{cat}$ of Reaction 37 are shown in Table ST.10.16.1.1.2 and plotted in Figure SF.10.16.1.1.1.

**Table ST.10.16.1.1.1.** Literature information used to design the ABC $k_{cat}$ parameter distribution. Each value was assigned a weight using the protocol described in Section 2.6.2. The type of error was assigned as 0 if it was additive and 1 if it was multiplicative.

| Value ($min^{-1}$) | Error ($min^{-1}$) | Experimental details | | | | | | Weight | Type of error | Reference |
|---|---|---|---|---|---|---|---|---|---|---|
| | | Species | Expression Vector | Enzyme | pH | Temperature (°C) | Other | | | |
| 3.50 | NaN | Bacteria | E. coli. | Glucose transporter | Unknown | Unknown | Bionumbers calculation | **8** | 0 | (Waygood and Steeves, 1980) |
| 3.30 x$10^{-1}$ | NaN | Bacteria | E. coli. | Lactose transporter | Unknown | Unknown | Bionumbers calculation | **8** | 0 | (Naftalin et al., 2007) |
| 8.30 x$10^{-1}$ | NaN | Bacteria | E. coli. | Lactose transporter | Unknown | Unknown | Bionumbers calculation | **8** | 0 | (Naftalin et al., 2007) |
| 3.28 | NaN | Yeast | Saccharomyces cerevisiae | HXT7 transporter | Unknown | Unknown | Bionumbers calculation | **8** | 0 | (Ye et al., 2001) |
| 1.13 x$10^{1}$ | | Primate | Baculovirus | MRP2 | 7 | 37 | | 512 | | (Yasunaga et al., 2008) |

**Table ST.10.16.1.1.2.** The log-normal distribution properties of the ABC $k_{cat}$ distribution. These values were calculated using the functions described in Section 2.6.2.

| **Mode ($min^{-1}$)** | **Confidence Interval** | **Location parameter (μ)** | **Scale parameter (σ)** |
|---|---|---|---|
| 1.13 $x10^{1}$ | 1.00 $x10^{1}$ | 3.22 | 8.91 $x10^{-1}$ |

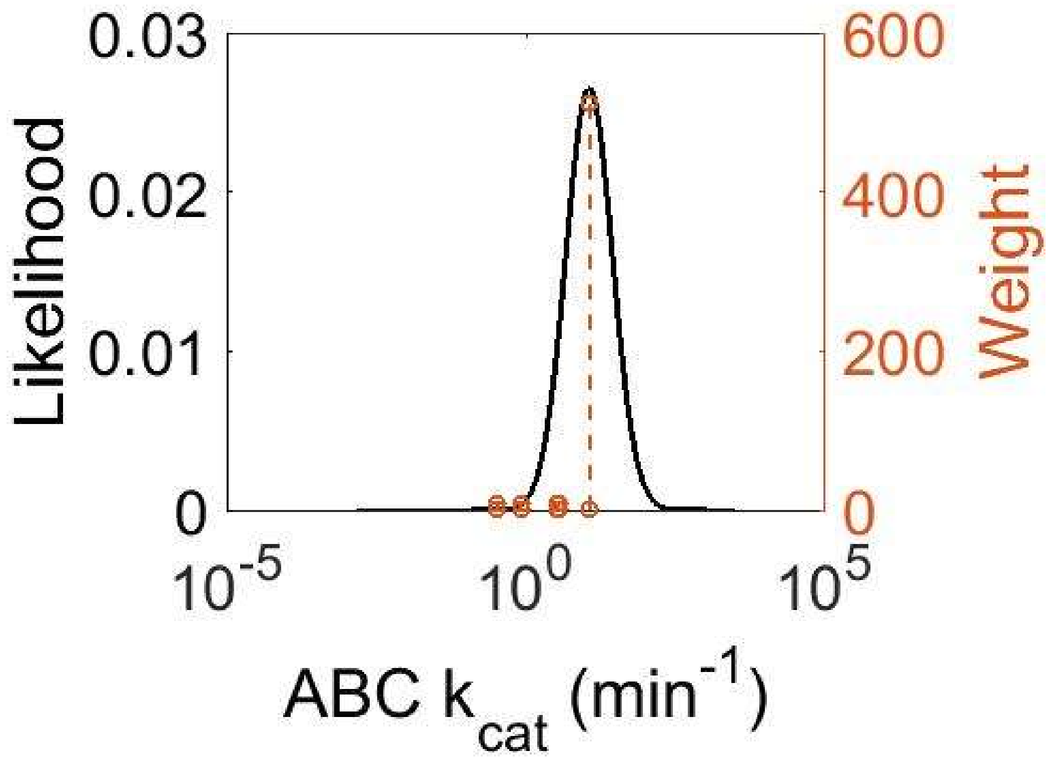


**Figure SF.10.16.1.1.1.** The estimated probability distribution for ABC $k_{cat}$. The value and weight of the literature values used to define the distribution are indicated by an orange dashed line. The x axis is plotted on a log-scale.

### S.10.16.1.2. Parameter: ABC $K_{ms}$

Parameter values for the ABC $K_{ms}$ of Reaction 37 were obtained from the literature and summarised in Table ST.10.16.1.2.1. The log-normal distribution properties for the ABC $K_{ms}$ of Reaction 37 are shown in Table ST.10.16.1.2.2 and plotted in Figure SF.10.16.1.2.1.

**Table ST.10.16.1.2.1.** Literature information used to design the ABC $K_{ms}$ parameter distribution. Each value was assigned a weight using the protocol described in Section 2.6.2. The type of error was assigned as 0 if it was additive and 1 if it was multiplicative.

| Value (mM) | Error (mM) | Experimental details | | | | | | Weight | Type of error | Reference |
|---|---|---|---|---|---|---|---|---|---|---|
| | | Species | Expression Vector | Enzyme | pH | Temperature (°C) | Other | | | |
| $1.09 \times 10^{-2}$ | $4.10 \times 10^{-3}$ | Human | Primate | MRP2 | 7 | 37 | | **512** | 0 | (Yasunaga et al., 2008) |
| $3.66 \times 10^{-5}$ | $3.80 \times 10^{-6}$ | Unknown | H69AR plasma membrane | MRP1 | 7.4 | 37 | | **128** | 0 | (Mao et al., 2000) |
| $5.30 \times 10^{-3}$ | $2.60 \times 10^{-3}$ | Human | HEK293 cells | MRP3 | 7.4 | 37 | | **128** | 0 | (Zeng et al., 2000) |
| $1.95 \times 10^{-1}$ | $6.12 \times 10^{-2}$ | Human | Primate | MRP2 | 7 | 37 | | **512** | 0 | (Yasunaga et al., 2008) |
| $1.10 \times 10^{-1}$ | $3.91 \times 10^{-2}$ | Human | Primate | MRP2 | 7 | 37 | | **512** | 0 | (Yasunaga et al., 2008) |
| $2.07 \times 10^{-2}$ | NaN | Human | HepG2 hepatoma | ABCB1 | 7.4 | 37 | | **2048** | 0 | (Fong et al., 2007) |

**Table ST.10.16.1.2.2.** The log-normal distribution properties of the ABC $K_{ms}$ distribution. These values were calculated using the functions described in Section 2.6.2.

| **Mode (mM)** | **Confidence Interval** | **Location parameter (μ)** | **Scale parameter (σ)** |
|---|---|---|---|
| $2.10 \times 10^{-2}$ | $2.37 \times 10^{1}$ | -2.60 | 1.12 |

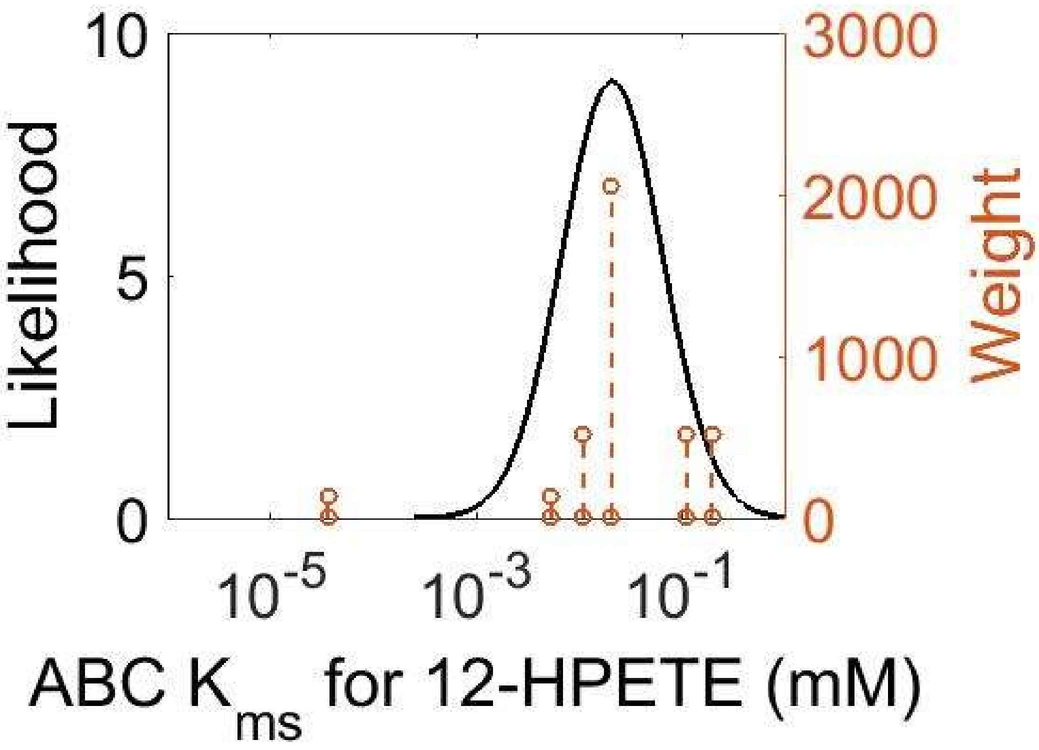


**Figure SF.10.16.1.2.1.** The estimated probability distribution for ABC $K_{ms}$. The value and weight of the literature values used to define the distribution are indicated by an orange dashed line. The x axis is plotted on a log-scale.

**S.10.16.1.3. Parameter: ABC concentration**

Parameter values for the ABC concentration of Reaction 37 were obtained from the literature and summarised in Table ST.10.16.1.3.1. The log-normal distribution properties for the ABC concentration of Reaction 37 are shown in Table ST.10.16.1.3.2 and plotted in Figure SF.10.16.1.3.1. To convert the enzyme concentration from ppm to mM, **Equation S.6.2** was used.

**Table ST.10.16.1.3.1.** Literature information used to design the ABC concentration parameter distribution. Each value was assigned a weight using the protocol described in Section 2.6.2. The type of error was assigned as 0 if it was additive and 1 if it was multiplicative.

| Value (ppm) | Error (ppm) | Experimental details | | | | | | Weight | Type of error | Reference |
|---|---|---|---|---|---|---|---|---|---|---|
| | | Species | Expression Vector | Enzyme | pH | Temperature (°C) | Other | | | |
| 5.15 | NaN | Human | Stomach | ABC | 7.5 | 37 | | 2048 | 0 | (Wilhelm et al., 2014) |
| 5.94 | NaN | Human | Lung | ABC | 7.5 | 37 | | 2048 | 0 | (Kim et al., 2014) |
| 2.66 | NaN | Human | Gut | ABC | 7.5 | 37 | | 2048 | 0 | (Kim et al., 2014) |

**Table ST.10.16.1.3.2.** The log-normal distribution properties of the ABC concentration distribution. These values were calculated using the functions described in Section 2.6.2.

| Mode (ppm) | Mode (mM) | Confidence Interval | Location parameter (μ) | Scale parameter (σ) |
|---|---|---|---|---|
| 5.06 | $2.80 \times 10^{-5}$ | 1.44 | 1.74 | $3.42 \times 10^{-1}$ |

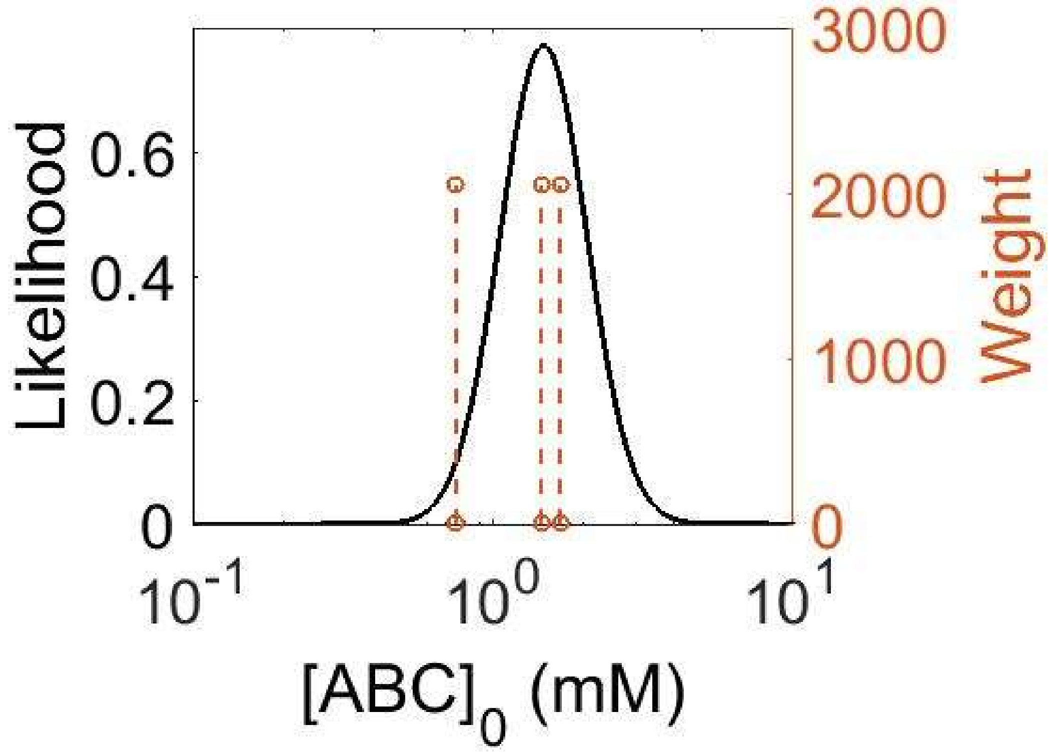


**Figure SF.10.16.1.3.1.** The estimated probability distribution for the ABC concentration. The value and weight of the literature values used to define the distribution are indicated by an orange dashed line. The x axis is plotted on a log-scale.

**S.10.17. Reaction 38: 15-HPETE ⇌ ex15-HPETE**

15-HPETE is relocated from the intracellular compartment to the extracellular compartment via the ATP-binding cassette (ABC) transporter.

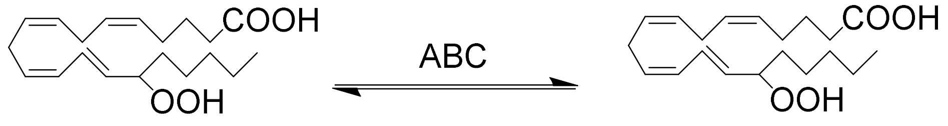


15-HPETE (Intracellular Compartment)

ex15-HPETE (Extracellular Compartment)

**Figure SF.10.17.** The transport of 15-hydroperoxyeicosatetraenoic acid (15-HPETE) from the intracellular compartment to the extracellular compartment by ATP Binding Cassette protein (ABC) (Reaction 38).

**SEq.10.17.** Reaction rate law for Reaction 38.

$$\boldsymbol{v_{38}} = [\boldsymbol{ABC}] \cdot \boldsymbol{k_{cat}} \cdot \frac{\mathbf{15{-}HPETE}/\boldsymbol{K_m} \cdot \left(1 - \frac{\mathbf{ex15{-}HPETE}}{\mathbf{15{-}HPETE} \cdot e^{-0.5 \cdot \left(G + R \cdot T \cdot \ln\left(\frac{ATP}{ADP}\right)\right) / R \cdot T}}\right)}{1 + \frac{\mathbf{15{-}HPETE}}{K_m} + \frac{\mathbf{ex15{-}HPETE}}{K_m} + \mathbf{ABC\_CI}}$$

## S.10.17.1 Reaction parameters

### S.10.17.1.1. Parameter: ABC $k_{cat}$

Parameter values for the ABC $k_{cat}$ of Reaction 38 were obtained from the literature and summarised in Table ST.10.17.1.1.1. The log-normal distribution properties for the ABC $k_{cat}$ of Reaction 38 are shown in Table ST.10.17.1.1.2 and plotted in Figure SF.10.17.1.1.1.

**Table ST.10.17.1.1.1.** Literature information used to design the ABC $k_{cat}$ parameter distribution. Each value was assigned a weight using the protocol described in Section 2.6.2. The type of error was assigned as 0 if it was additive and 1 if it was multiplicative.

| Value ($min^{-1}$) | Error ($min^{-1}$) | Experimental details | | | | | | Weight | Type of error | Reference |
|---|---|---|---|---|---|---|---|---|---|---|
| | | Species | Expression Vector | Enzyme | pH | Temperature (°C) | Other | | | |
| 3.50 | NaN | Bacteria | E. coli. | Glucose transporter | Unknown | Unknown | Bionumbers calculation | **8** | 0 | (Waygood and Steeves, 1980) |
| 3.30 $x10^{-1}$ | NaN | Bacteria | E. coli. | Lactose transporter | Unknown | Unknown | Bionumbers calculation | **8** | 0 | (Naftalin et al., 2007) |
| 8.30 $x10^{-1}$ | NaN | Bacteria | E. coli. | Lactose transporter | Unknown | Unknown | Bionumbers calculation | **8** | 0 | (Naftalin et al., 2007) |
| 3.28 | NaN | Yeast | Saccharomyces cerevisiae | HXT7 transporter | Unknown | Unknown | Bionumbers calculation | **8** | 0 | (Ye et al., 2001) |
| 1.13 $x10^{1}$ | | Primate | Baculovirus | MRP2 | 7 | 37 | | 512 | | (Yasunaga et al., 2008) |

**Table ST.10.17.1.1.2.** The log-normal distribution properties of the ABC $k_{cat}$ distribution. These values were calculated using the functions described in Section 2.6.2.

| **Mode ($min^{-1}$)** | **Confidence Interval** | **Location parameter (μ)** | **Scale parameter (σ)** |
|---|---|---|---|
| $1.13 \times 10^{1}$ | $1.00 \times 10^{1}$ | 3.22 | $8.91 \times 10^{-1}$ |

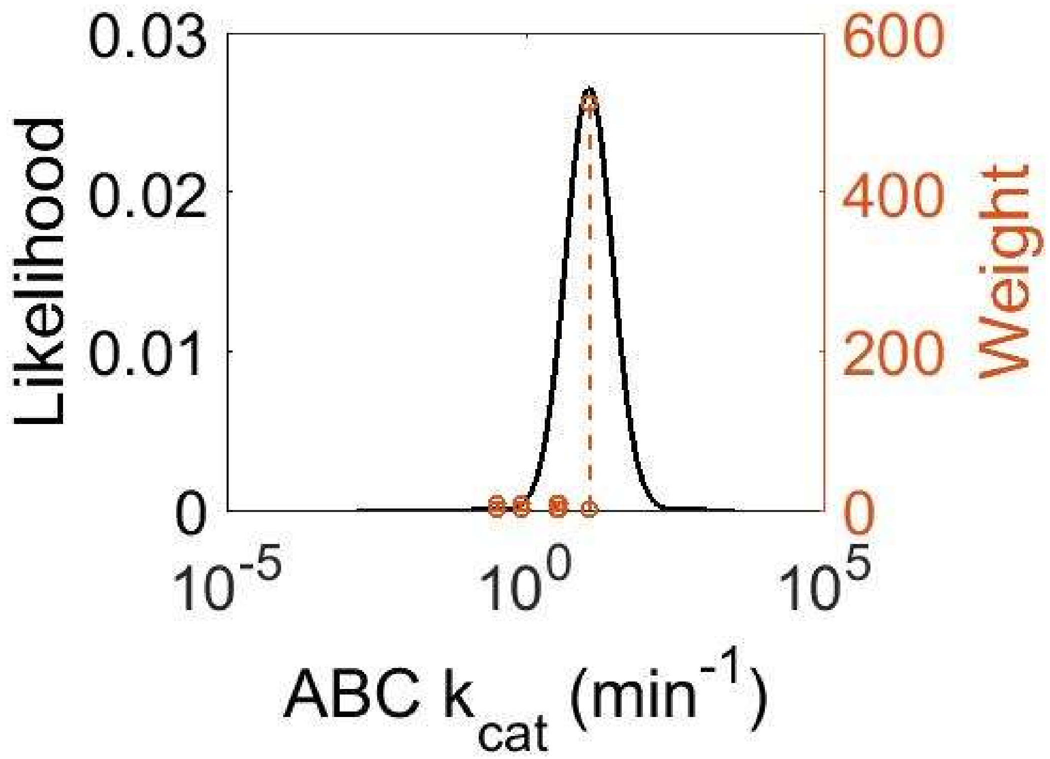


**Figure SF.10.17.1.1.1.** The estimated probability distribution for ABC $k_{cat}$. The value and weight of the literature values used to define the distribution are indicated by an orange dashed line. The x axis is plotted on a log-scale.

**S.10.17.1.2. Parameter: ABC $K_{ms}$**

Parameter values for the ABC $K_{ms}$ of Reaction 38 were obtained from the literature and summarised in Table ST.10.17.1.2.1. The log-normal distribution properties for the ABC $K_{ms}$ of Reaction 38 are shown in Table ST.10.17.1.2.2 and plotted in Figure SF.10.17.1.2.1.

**Table ST.10.17.1.2.1.** Literature information used to design the ABC $K_{ms}$ parameter distribution. Each value was assigned a weight using the protocol described in Section 2.6.2. The type of error was assigned as 0 if it was additive and 1 if it was multiplicative.

| Value (mM) | Error (mM) | Experimental details | | | | | | Weight | Type of error | Reference |
|---|---|---|---|---|---|---|---|---|---|---|
| | | Species | Expression Vector | Enzyme | pH | Temperature (°C) | Other | | | |
| $1.09 \times 10^{-2}$ | $4.10 \times 10^{-3}$ | Human | Primate | MRP2 | 7 | 37 | | **512** | 0 | (Yasunaga et al., 2008) |
| $3.66 \times 10^{-5}$ | $3.80 \times 10^{-6}$ | Unknown | H69AR plasma membrane | MRP1 | 7.4 | 37 | | **128** | 0 | (Mao et al., 2000) |
| $5.30 \times 10^{-3}$ | $2.60 \times 10^{-3}$ | Human | HEK293 cells | MRP3 | 7.4 | 37 | | **128** | 0 | (Zeng et al., 2000) |
| $1.95 \times 10^{-1}$ | $6.12 \times 10^{-2}$ | Human | Primate | MRP2 | 7 | 37 | | **512** | 0 | (Yasunaga et al., 2008) |
| $1.10 \times 10^{-1}$ | $3.91 \times 10^{-2}$ | Human | Primate | MRP2 | 7 | 37 | | **512** | 0 | (Yasunaga et al., 2008) |
| $2.07 \times 10^{-2}$ | NaN | Human | HepG2 hepatoma | ABCB1 | 7.4 | 37 | | **2048** | 0 | (Fong et al., 2007) |

**Table ST.10.17.1.2.2.** The log-normal distribution properties of the ABC $K_{ms}$ distribution. These values were calculated using the functions described in Section 2.6.2.

| Mode (mM) | Confidence Interval | Location parameter (μ) | Scale parameter (σ) |
|---|---|---|---|
| $2.10 \times 10^{-2}$ | $2.37 \times 10^{1}$ | -2.60 | 1.12 |

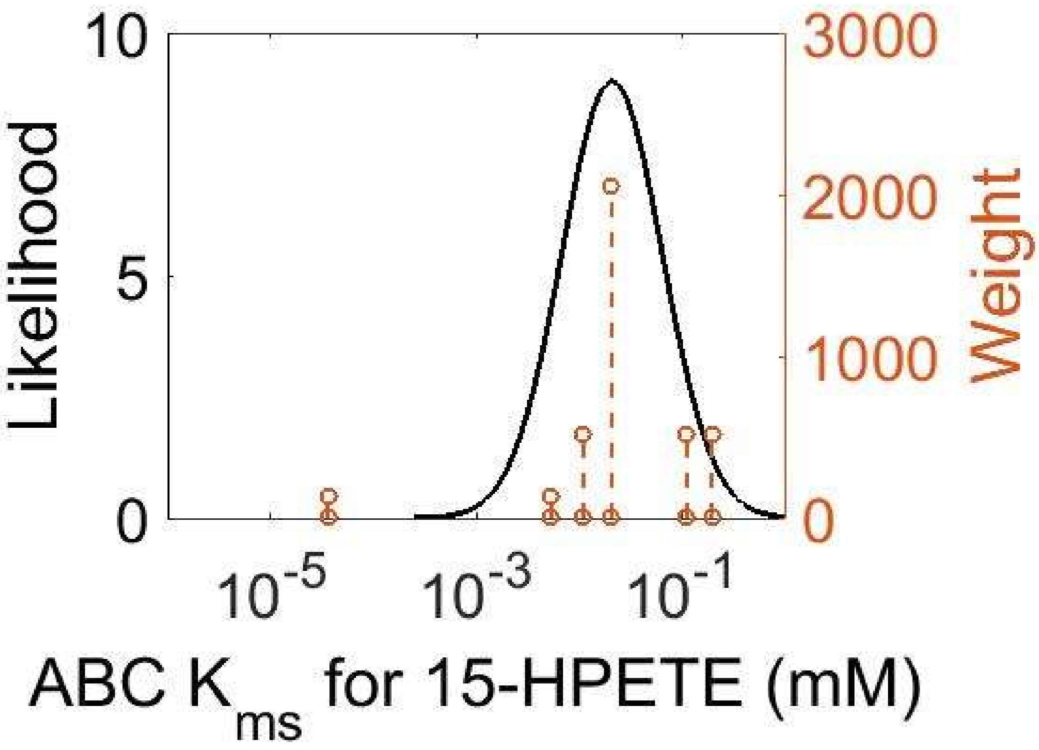


**Figure SF.10.17.1.2.1.** The estimated probability distribution for ABC $K_{ms}$. The value and weight of the literature values used to define the distribution are indicated by an orange dashed line. The x axis is plotted on a log-scale.

**S.10.17.1.3. Parameter: ABC concentration**

Parameter values for the ABC concentration of Reaction 38 were obtained from the literature and summarised in Table ST.10.17.1.3.1. The log-normal distribution properties for the ABC concentration of Reaction 38 are shown in Table ST.10.17.1.3.2 and plotted in Figure SF.10.17.1.3.1. To convert the enzyme concentration from ppm to mM, **Equation S.6.2** was used.

**Table ST.10.17.1.3.1.** Literature information used to design the ABC concentration parameter distribution. Each value was assigned a weight using the protocol described in Section 2.6.2. The type of error was assigned as 0 if it was additive and 1 if it was multiplicative.

| Value (ppm) | Error (ppm) | Experimental details | | | | | | Weight | Type of error | Reference |
|---|---|---|---|---|---|---|---|---|---|---|
| | | Species | Expression Vector | Enzyme | pH | Temperature (°C) | Other | | | |
| 5.15 | NaN | Human | Stomach | ABC | 7.5 | 37 | | 2048 | 0 | (Wilhelm et al., 2014) |
| 5.94 | NaN | Human | Lung | ABC | 7.5 | 37 | | 2048 | 0 | (Kim et al., 2014) |
| 2.66 | NaN | Human | Gut | ABC | 7.5 | 37 | | 2048 | 0 | (Kim et al., 2014) |

**Table ST.10.17.1.3.2.** The log-normal distribution properties of the ABC concentration distribution. These values were calculated using the functions described in Section 2.6.2.

| Mode (ppm) | Mode (mM) | Confidence Interval | Location parameter (μ) | Scale parameter (σ) |
|---|---|---|---|---|
| 5.06 | $2.80 \times 10^{-5}$ | 1.44 | 1.74 | $3.42 \times 10^{-1}$ |

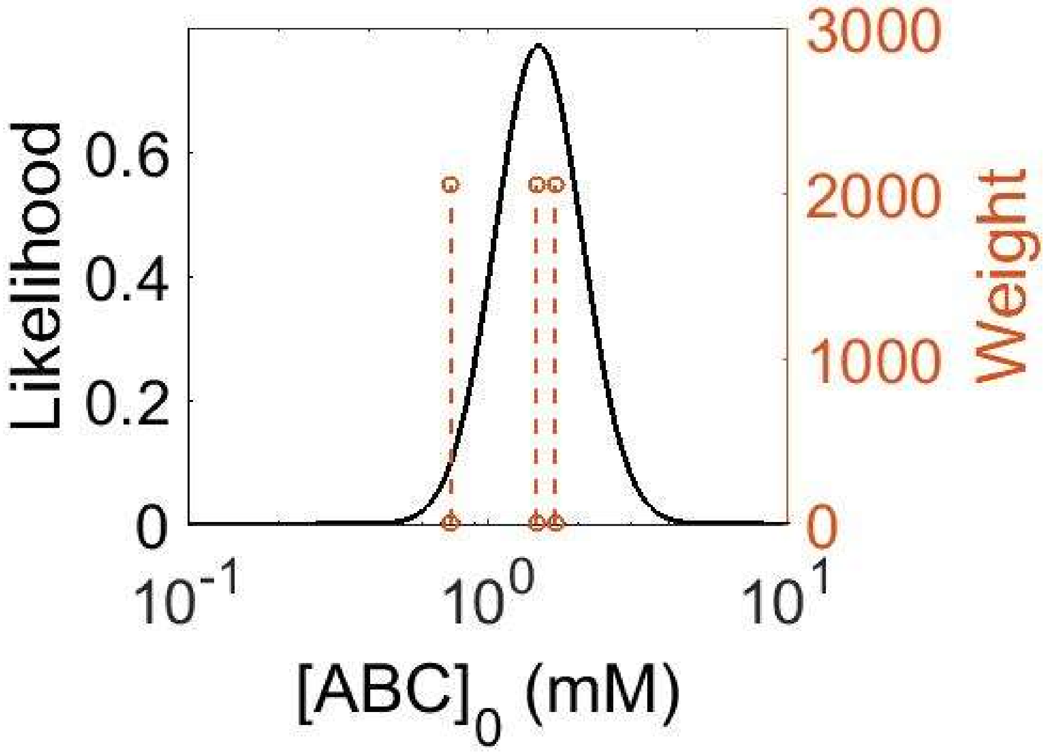


**Figure SF.10.17.1.3.1.** The estimated probability distribution for the ABC concentration. The value and weight of the literature values used to define the distribution are indicated by an orange dashed line. The x axis is plotted on a log-scale.

**S.10.18. Reaction 39: 5-HPETE ⇌ ex5-HPETE**

5-HPETE is relocated from the intracellular compartment to the extracellular compartment via the ATP-binding cassette (ABC) transporter.

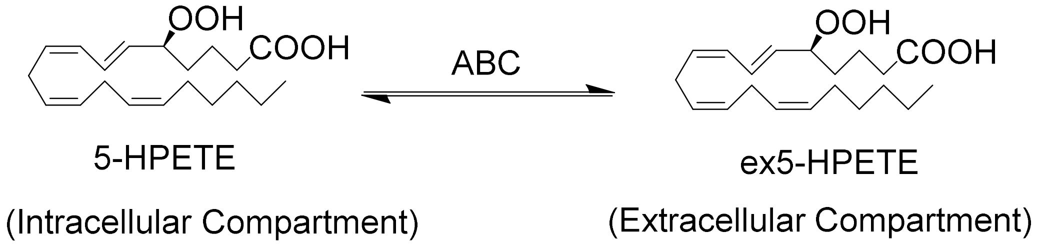


**Figure SF.10.18.** The transport of 5-hydroperoxyeicosatetraenoic acid (5-HPETE) from the intracellular compartment to the extracellular compartment by ATP Binding Cassette protein (ABC) (Reaction 39).

**SEq.10.18.** Reaction rate law for Reaction 39.

$$v_{39} = [ABC] \cdot k_{cat} \cdot \frac{{}^{\text{5−HPETE}}/_{K_m} \cdot \left(1 - \frac{\text{ex5−HPETE}}{\text{5−HPETE} \cdot e^{-0.5 \cdot \left(G + R \cdot T \cdot \ln\left(\frac{ATP}{ADP}\right)\right)/_{R \cdot T}}}\right)}{1 + \frac{\text{5−HPETE}}{K_m} + \frac{\text{ex5−HPETE}}{K_m} + \text{ABC\_CI}}$$

### S.10.18.1. Reaction parameters

#### S.10.18.1.1. Parameter: ABC $k_{cat}$

Parameter values for the ABC $k_{cat}$ of Reaction 39 were obtained from the literature and summarised in Table ST.10.18.1.1.1. The log-normal distribution properties for the ABC $k_{cat}$ of Reaction 39 are shown in Table ST.10.18.1.1.2 and plotted in Figure SF.10.18.1.1.1.

**Table ST.10.18.1.1.1.** Literature information used to design the ABC $k_{cat}$ parameter distribution. Each value was assigned a weight using the protocol described in Section 2.6.2. The type of error was assigned as 0 if it was additive and 1 if it was multiplicative.

| Value ($min^{-1}$) | Error ($min^{-1}$) | Experimental details | | | | | | Weight | Type of error | Reference |
|---|---|---|---|---|---|---|---|---|---|---|
| | | Species | Expression Vector | Enzyme | pH | Temperature (°C) | Other | | | |
| 3.50 | NaN | Bacteria | E. coli. | Glucose transporter | Unknown | Unknown | Bionumbers calculation | **8** | 0 | (Waygood and Steeves, 1980) |
| 3.30 $x10^{-1}$ | NaN | Bacteria | E. coli. | Lactose transporter | Unknown | Unknown | Bionumbers calculation | **8** | 0 | (Naftalin et al., 2007) |
| 8.30 $x10^{-1}$ | NaN | Bacteria | E. coli. | Lactose transporter | Unknown | Unknown | Bionumbers calculation | **8** | 0 | (Naftalin et al., 2007) |
| 3.28 | NaN | Yeast | Saccharomyces cerevisiae | HXT7 transporter | Unknown | Unknown | Bionumbers calculation | **8** | 0 | (Ye et al., 2001) |
| 1.13 $x10^{1}$ | | Primate | Baculovirus | MRP2 | 7 | 37 | | 512 | | (Yasunaga et al., 2008) |

**Table ST.10.18.1.1.2.** The log-normal distribution properties of the ABC $k_{cat}$ distribution. These values were calculated using the functions described in Section 2.6.2.

| Mode ($min^{-1}$) | Confidence Interval | Location parameter (μ) | Scale parameter (σ) |
|---|---|---|---|
| 1.13 $x10^{1}$ | 1.00 $x10^{1}$ | 3.22 | 8.91 $x10^{-1}$ |

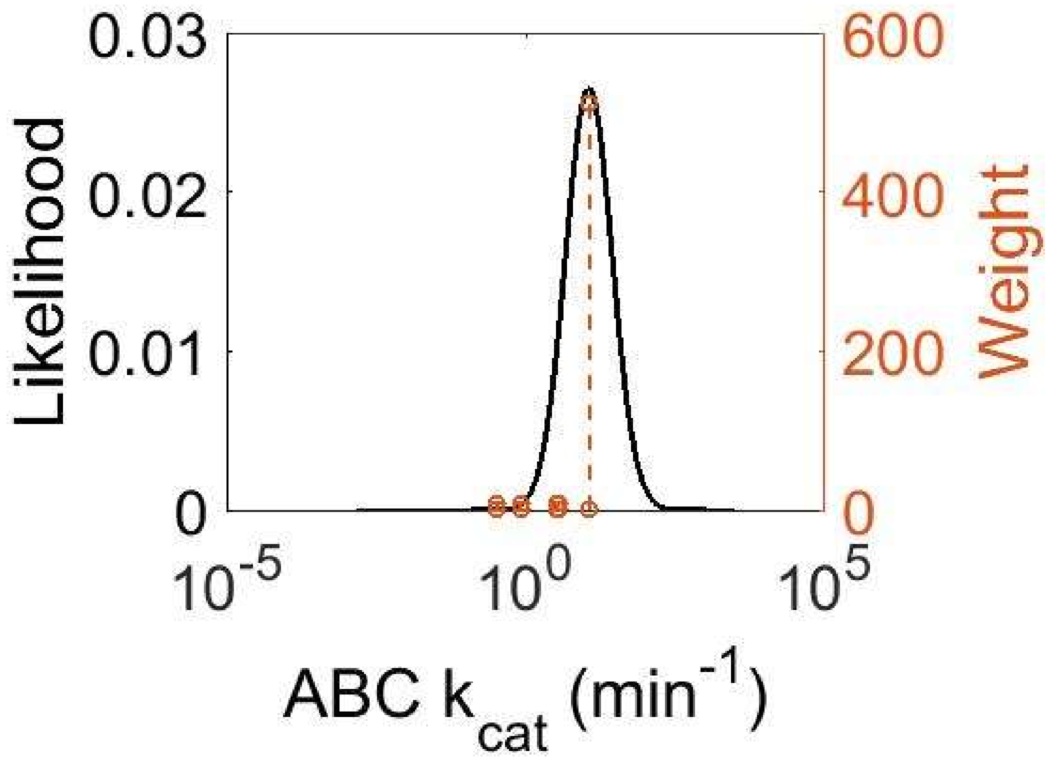


**Figure SF.10.18.1.1.1.** The estimated probability distribution for ABC $k_{cat}$. The value and weight of the literature values used to define the distribution are indicated by an orange dashed line. The x axis is plotted on a log-scale.

**S.10.18.1.2. Parameter: ABC $K_{ms}$**

Parameter values for the ABC $K_{ms}$ of Reaction 39 were obtained from the literature and summarised in Table ST.10.18.1.2.1. The log-normal distribution properties for the ABC $K_{ms}$ of Reaction 39 are shown in Table ST.10.18.1.2.2 and plotted in Figure SF.10.18.1.2.1.

**Table ST.10.18.1.2.1.** Literature information used to design the ABC $K_{ms}$ parameter distribution. Each value was assigned a weight using the protocol described in Section 2.6.2. The type of error was assigned as 0 if it was additive and 1 if it was multiplicative.

| Value (mM) | Error (mM) | Experimental details | | | | | | Weight | Type of error | Reference |
|---|---|---|---|---|---|---|---|---|---|---|
| | | Species | Expression Vector | Enzyme | pH | Temperature (°C) | Other | | | |
| $1.09 \times 10^{-2}$ | $4.10 \times 10^{-3}$ | Human | Primate | MRP2 | 7 | 37 | | **512** | 0 | (Yasunaga et al., 2008) |
| $3.66 \times 10^{-5}$ | $3.80 \times 10^{-6}$ | Unknown | H69AR plasma membrane | MRP1 | 7.4 | 37 | | **128** | 0 | (Mao et al., 2000) |
| $5.30 \times 10^{-3}$ | $2.60 \times 10^{-3}$ | Human | HEK293 cells | MRP3 | 7.4 | 37 | | **128** | 0 | (Zeng et al., 2000) |
| $1.95 \times 10^{-1}$ | $6.12 \times 10^{-2}$ | Human | Primate | MRP2 | 7 | 37 | | **512** | 0 | (Yasunaga et al., 2008) |
| $1.10 \times 10^{-1}$ | $3.91 \times 10^{-2}$ | Human | Primate | MRP2 | 7 | 37 | | **512** | 0 | (Yasunaga et al., 2008) |
| $2.07 \times 10^{-2}$ | NaN | Human | HepG2 hepatoma | ABCB1 | 7.4 | 37 | | **2048** | 0 | (Fong et al., 2007) |

**Table ST.10.18.1.2.2.** The log-normal distribution properties of the ABC $K_{ms}$ distribution. These values were calculated using the functions described in Section 2.6.2.

| Mode (mM) | Confidence Interval | Location parameter (μ) | Scale parameter (σ) |
|---|---|---|---|
| $2.10 \times 10^{-2}$ | $2.37 \times 10^{1}$ | -2.60 | 1.12 |

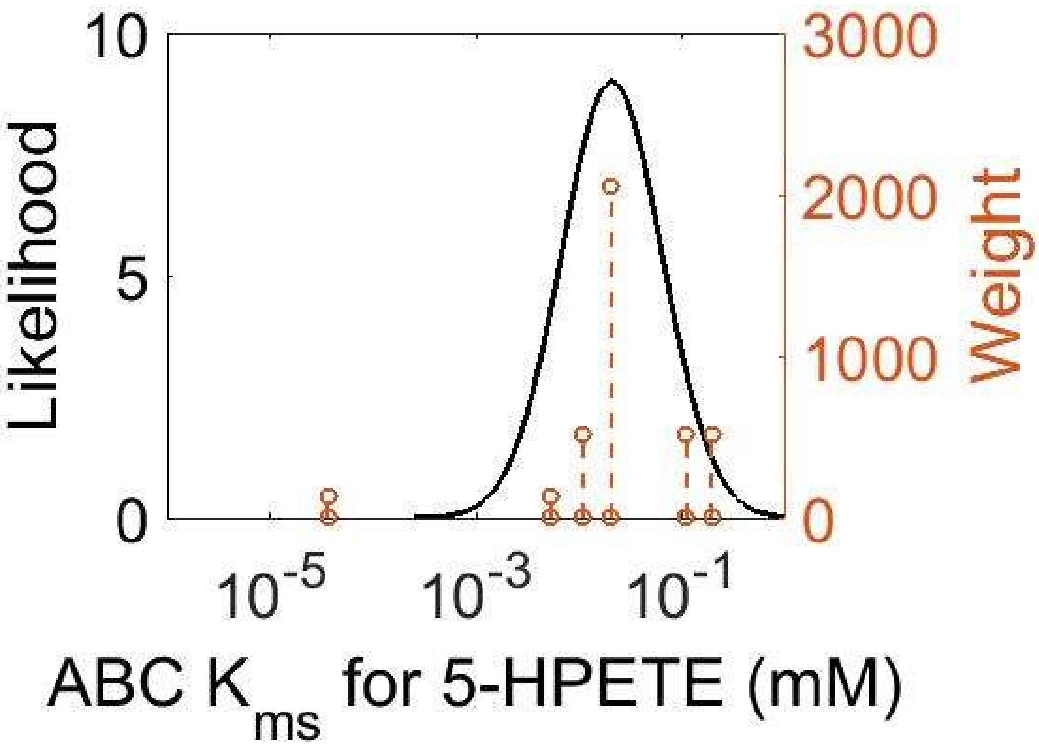


**Figure SF.10.18.1.2.1.** The estimated probability distribution for ABC $K_{ms}$. The value and weight of the literature values used to define the distribution are indicated by an orange dashed line. The x axis is plotted on a log-scale.

**S.10.18.1.3. Parameter: ABC concentration**

Parameter values for the ABC concentration of Reaction 39 were obtained from the literature and summarised in Table ST.10.18.1.3.1. The log-normal distribution properties for the ABC concentration of Reaction 39 are shown in Table ST.10.18.1.3.2 and plotted in Figure SF.10.18.1.3.1. To convert the enzyme concentration from ppm to mM, **Equation S.6.2** was used.

**Table ST.10.18.1.3.1.** Literature information used to design the ABC concentration parameter distribution. Each value was assigned a weight using the protocol described in Section 2.6.2. The type of error was assigned as 0 if it was additive and 1 if it was multiplicative.

| Value (ppm) | Error (ppm) | Experimental details | | | | | | Weight | Type of error | Reference |
|---|---|---|---|---|---|---|---|---|---|---|
| | | Species | Expression Vector | Enzyme | pH | Temperature (°C) | Other | | | |
| 5.15 | NaN | Human | Stomach | ABC | 7.5 | 37 | | 2048 | 0 | (Wilhelm et al., 2014) |
| 5.94 | NaN | Human | Lung | ABC | 7.5 | 37 | | 2048 | 0 | (Kim et al., 2014) |
| 2.66 | NaN | Human | Gut | ABC | 7.5 | 37 | | 2048 | 0 | (Kim et al., 2014) |

**Table ST.10.18.1.3.2.** The log-normal distribution properties of the ABC concentration distribution. These values were calculated using the functions described in Section 2.6.2.

| Mode (ppm) | Mode (mM) | Confidence Interval | Location parameter (μ) | Scale parameter (σ) |
|---|---|---|---|---|
| 5.06 | $2.80 \times 10^{-5}$ | 1.44 | 1.74 | $3.42 \times 10^{-1}$ |

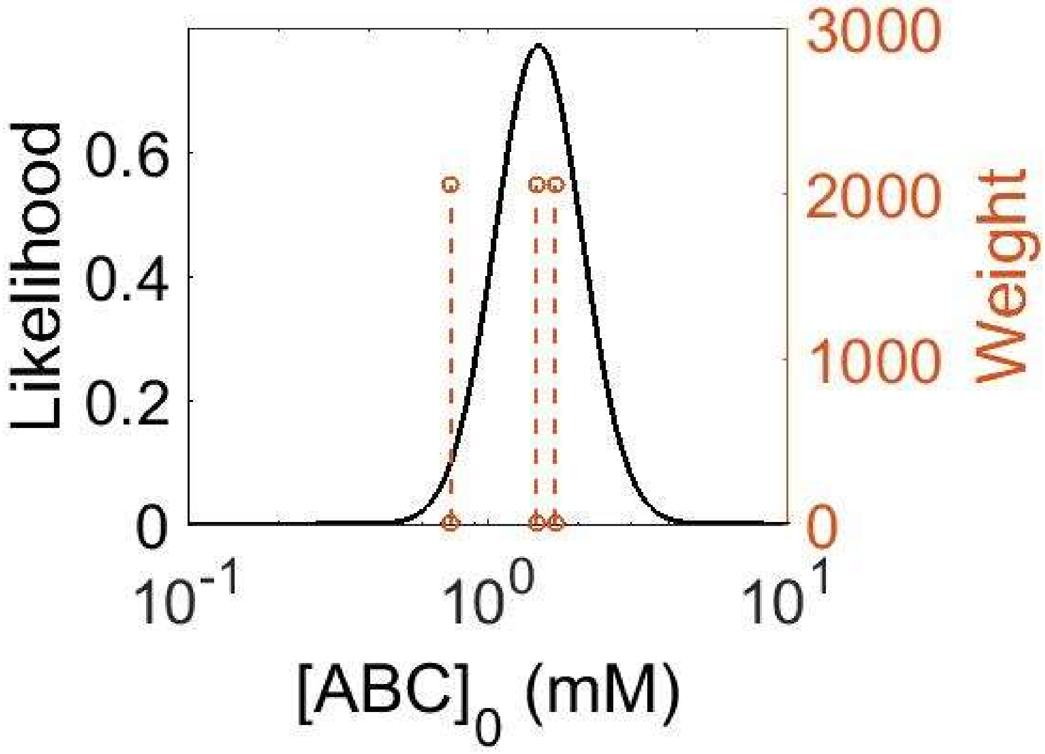


**Figure SF.10.18.1.3.1.** The estimated probability distribution for the ABC concentration. The value and weight of the literature values used to define the distribution are indicated by an orange dashed line. The x axis is plotted on a log-scale.

### S.10.19. Reaction 40: 5-HETE ⇌ ex5-HETE

5-HETE is relocated from the intracellular compartment to the extracellular compartment via the ATP-binding cassette (ABC) transporter.

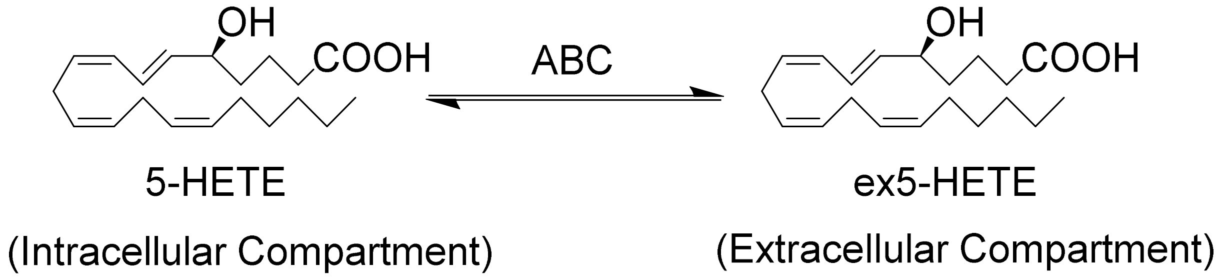


**Figure SF.10.19.** The transport of 5-hydroxyeicosatetraenoic acid (5-HETE) from the intracellular compartment to the extracellular compartment by ATP Binding Cassette protein (ABC) (Reaction 40).

**SEq.10.19.** Reaction rate law for Reaction 40.

$$v_{40} = [ABC] \cdot k_{cat} \cdot \frac{\mathrm{5{-}HETE}/K_m \cdot \left(1 - \frac{\mathrm{ex5{-}HETE}}{\mathrm{5{-}HETE} \cdot e^{-0.5 \cdot \left(G + R \cdot T \cdot \ln\left(\frac{ATP}{ADP}\right)\right)/R \cdot T}}\right)}{1 + \frac{\mathrm{5{-}HETE}}{K_m} + \frac{\mathrm{ex5{-}HETE}}{K_m} + \mathrm{ABC\_CI}}$$

### S.10.19.1. Reaction parameters

#### S.10.19.1.1. Parameter: ABC $k_{cat}$

Parameter values for the ABC $k_{cat}$ of Reaction 40 were obtained from the literature and summarised in Table ST.10.19.1.1.1. The log-normal distribution properties for the ABC $k_{cat}$ of Reaction 40 are shown in Table ST.10.19.1.1.2 and plotted in Figure SF.10.19.1.1.1.

**Table ST.10.19.1.1.1.** Literature information used to design the ABC $k_{cat}$ parameter distribution. Each value was assigned a weight using the protocol described in Section 2.6.2. The type of error was assigned as 0 if it was additive and 1 if it was multiplicative.

| Value ($min^{-1}$) | Error ($min^{-1}$) | Experimental details | | | | | | Weight | Type of error | Reference |
|---|---|---|---|---|---|---|---|---|---|---|
| | | Species | Expression Vector | Enzyme | pH | Temperature (°C) | Other | | | |
| 3.50 | NaN | Bacteria | E. coli. | Glucose transporter | Unknown | Unknown | Bionumbers calculation | **8** | 0 | (Waygood and Steeves, 1980) |
| 3.30 x$10^{-1}$ | NaN | Bacteria | E. coli. | Lactose transporter | Unknown | Unknown | Bionumbers calculation | **8** | 0 | (Naftalin et al., 2007) |
| 8.30 x$10^{-1}$ | NaN | Bacteria | E. coli. | Lactose transporter | Unknown | Unknown | Bionumbers calculation | **8** | 0 | (Naftalin et al., 2007) |
| 3.28 | NaN | Yeast | Saccharomyces cerevisiae | HXT7 transporter | Unknown | Unknown | Bionumbers calculation | **8** | 0 | (Ye et al., 2001) |
| 1.13 x$10^{1}$ | | Primate | Baculovirus | MRP2 | 7 | 37 | | 512 | | (Yasunaga et al., 2008) |

**Table ST.10.19.1.1.2.** The log-normal distribution properties of the ABC $k_{cat}$ distribution. These values were calculated using the functions described in Section 2.6.2.

| Mode ($min^{-1}$) | Confidence Interval | Location parameter (μ) | Scale parameter (σ) |
|---|---|---|---|
| 1.13 $x10^{1}$ | 1.00 $x10^{1}$ | 3.22 | $8.9110^{-1}$ |

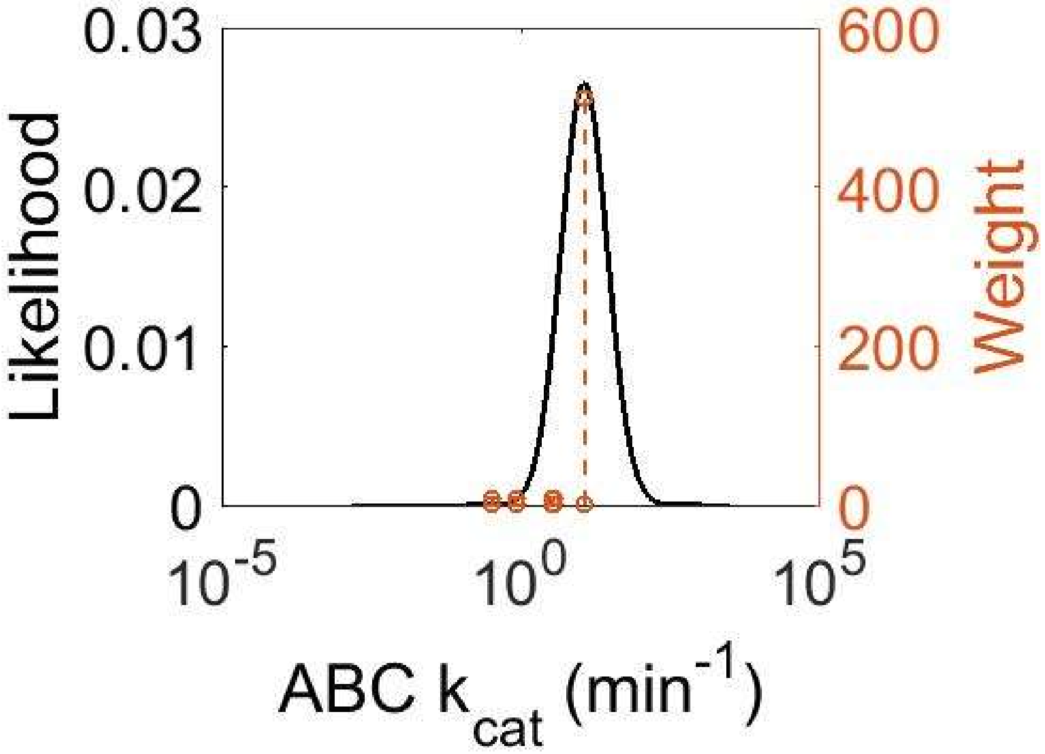


**Figure SF.10.19.1.1.1.** The estimated probability distribution for ABC $k_{cat}$. The value and weight of the literature values used to define the distribution are indicated by an orange dashed line. The x axis is plotted on a log-scale.

**S.10.19.1.2. Parameter: ABC $K_{ms}$**

Parameter values for the ABC $K_{ms}$ of Reaction 40 were obtained from the literature and summarised in Table ST.10.19.1.2.1. The log-normal distribution properties for the ABC $K_{ms}$ of Reaction 40 are shown in Table ST.10.19.1.2.2 and plotted in Figure SF.10.19.1.2.1.

**Table ST.10.19.1.2.1.** Literature information used to design the ABC $K_{ms}$ parameter distribution. Each value was assigned a weight using the protocol described in Section 2.6.2. The type of error was assigned as 0 if it was additive and 1 if it was multiplicative.

| Value (mM) | Error (mM) | Experimental details | | | | | | Weight | Type of error | Reference |
|---|---|---|---|---|---|---|---|---|---|---|
| | | Species | Expression Vector | Enzyme | pH | Temperature (°C) | Other | | | |
| $1.09 \times 10^{-2}$ | $4.10 \times 10^{-3}$ | Human | Primate | MRP2 | 7 | 37 | | **512** | 0 | (Yasunaga et al., 2008) |
| $3.66 \times 10^{-5}$ | $3.80 \times 10^{-6}$ | Unknown | H69AR plasma membrane | MRP1 | 7.4 | 37 | | **128** | 0 | (Mao et al., 2000) |
| $5.30 \times 10^{-3}$ | $2.60 \times 10^{-3}$ | Human | HEK293 cells | MRP3 | 7.4 | 37 | | **128** | 0 | (Zeng et al., 2000) |
| $1.95 \times 10^{-1}$ | $6.12 \times 10^{-2}$ | Human | Primate | MRP2 | 7 | 37 | | **512** | 0 | (Yasunaga et al., 2008) |
| $1.10 \times 10^{-1}$ | $3.91 \times 10^{-2}$ | Human | Primate | MRP2 | 7 | 37 | | **512** | 0 | (Yasunaga et al., 2008) |
| $2.07 \times 10^{-2}$ | NaN | Human | HepG2 hepatoma | ABCB1 | 7.4 | 37 | | **2048** | 0 | (Fong et al., 2007) |

**Table ST.10.19.1.2.2.** The log-normal distribution properties of the ABC $K_{ms}$ distribution. These values were calculated using the functions described in Section 2.6.2.

| Mode (mM) | Confidence Interval | Location parameter (μ) | Scale parameter (σ) |
|---|---|---|---|
| 2.10 $\times 10^{-2}$ | 2.37 $\times 10^{1}$ | -2.60 | 1.12 |

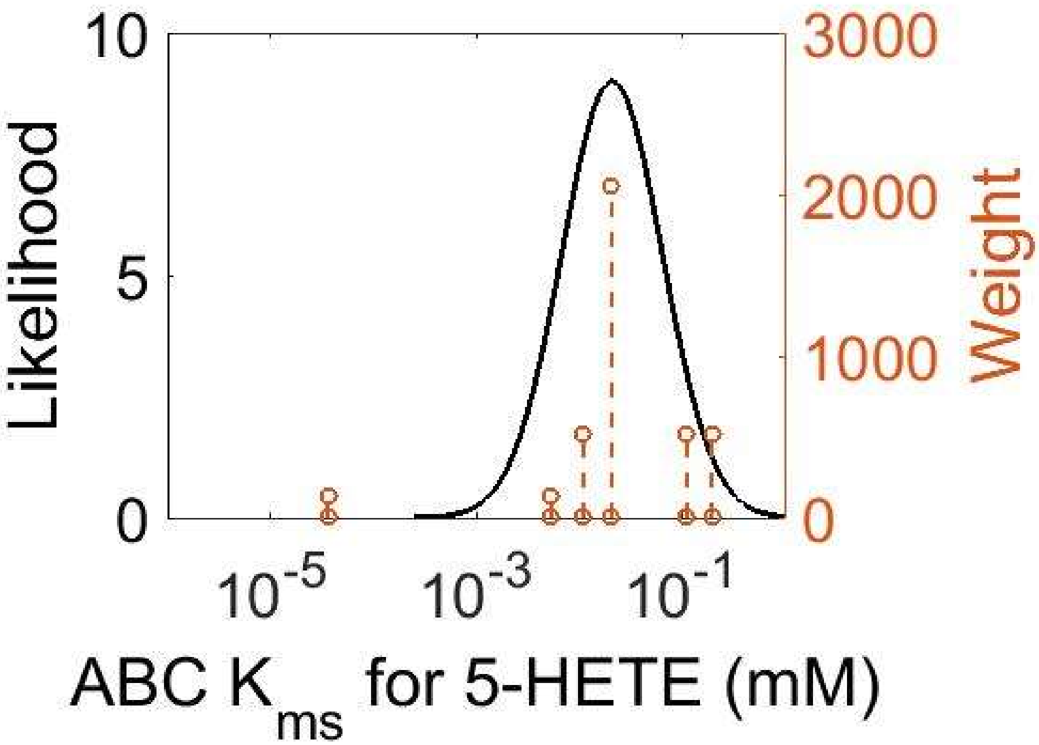


**Figure SF.10.19.1.2.1.** The estimated probability distribution for ABC $K_{ms}$. The value and weight of the literature values used to define the distribution are indicated by an orange dashed line. The x axis is plotted on a log-scale.

**S.10.19.1.3. Parameter: ABC concentration**

Parameter values for the ABC concentration of Reaction 40 were obtained from the literature and summarised in Table ST.10.19.1.3.1. The log-normal distribution properties for the ABC concentration of Reaction 40 are shown in Table ST.10.19.1.3.2 and plotted in Figure SF.10.19.1.3.1. To convert the enzyme concentration from ppm to mM, **Equation S.6.2** was used.

**Table ST.10.19.1.3.1.** Literature information used to design the ABC concentration parameter distribution. Each value was assigned a weight using the protocol described in Section 2.6.2. The type of error was assigned as 0 if it was additive and 1 if it was multiplicative.

| Value (ppm) | Error (ppm) | Experimental details | | | | | | Weight | Type of error | Reference |
|---|---|---|---|---|---|---|---|---|---|---|
| | | Species | Expression Vector | Enzyme | pH | Temperature (°C) | Other | | | |
| 5.15 | NaN | Human | Stomach | ABC | 7.5 | 37 | | 2048 | 0 | (Wilhelm et al., 2014) |
| 5.94 | NaN | Human | Lung | ABC | 7.5 | 37 | | 2048 | 0 | (Kim et al., 2014) |
| 2.66 | NaN | Human | Gut | ABC | 7.5 | 37 | | 2048 | 0 | (Kim et al., 2014) |

**Table ST.10.19.1.3.2.** The log-normal distribution properties of the ABC concentration distribution. These values were calculated using the functions described in Section 2.6.2.

| Mode (ppm) | Mode (mM) | Confidence Interval | Location parameter (μ) | Scale parameter (σ) |
|---|---|---|---|---|
| 5.06 | $2.80 \text{ x}10^{-5}$ | 1.44 | 1.74 | $3.4210^{-1}$ |

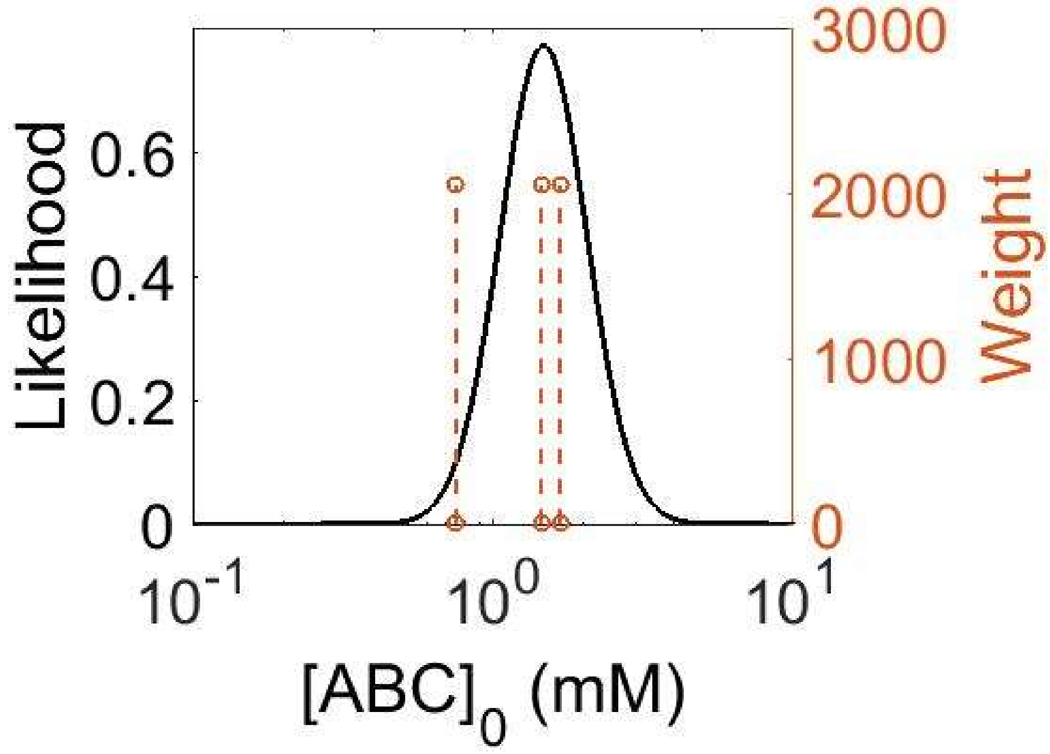


**Figure SF.10.19.1.3.1.** The estimated probability distribution for the ABC concentration. The value and weight of the literature values used to define the distribution are indicated by an orange dashed line. The x axis is plotted on a log-scale.

**S.10.20. Reaction 41: $LTA_4 \rightleftharpoons exLTA_4$**

$LTA_4$ is relocated from the intracellular compartment to the extracellular compartment via the ATP-binding cassette (ABC) transporter.

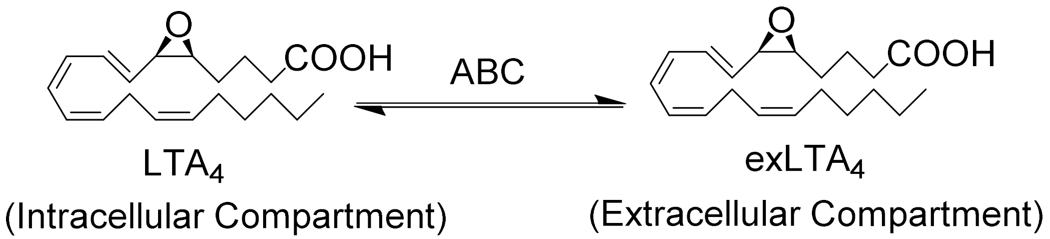


**Figure SF.10.20.** The transport of leukotriene $A_4$ ($LTA_4$) from the intracellular compartment to the extracellular compartment by ATP Binding Cassette protein (ABC) (Reaction 41).

**SEq.10.20. Reaction rate law for Reaction 41.**

$$\boldsymbol{v_{41}} = [\boldsymbol{ABC}] \cdot \boldsymbol{k_{cat}} \cdot \frac{\mathbf{LTA_4}/K_m \cdot \left(1 - \frac{\mathbf{exLTA_4}}{\mathbf{LTA_4} \cdot e^{-0.5\cdot\left(G + R\cdot T\cdot \ln\left(\frac{ATP}{ADP}\right)\right)/R\cdot T}}\right)}{1 + \frac{\mathbf{LTA_4}}{K_m} + \frac{\mathbf{exLTA_4}}{K_m} + \mathbf{ABC\_CI}}$$

### S.10.20.1. Reaction parameters

#### S.10.20.1.1. Parameter: ABC $k_{cat}$

Parameter values for the ABC $k_{cat}$ of Reaction 41 were obtained from the literature and summarised in Table ST.10.20.1.1.1. The log-normal distribution properties for the ABC $k_{cat}$ of Reaction 41 are shown in Table ST.10.20.1.1.2 and plotted in Figure SF.10.20.1.1.1.

**Table ST.10.20.1.1.1.** Literature information used to design the ABC $k_{cat}$ parameter distribution. Each value was assigned a weight using the protocol described in Section 2.6.2. The type of error was assigned as 0 if it was additive and 1 if it was multiplicative.

| Value ($min^{-1}$) | Error ($min^{-1}$) | Experimental details | | | | | | Weight | Type of error | Reference |
|---|---|---|---|---|---|---|---|---|---|---|
| | | Species | Expression Vector | Enzyme | pH | Temperature (°C) | Other | | | |
| 3.50 | NaN | Bacteria | E. coli. | Glucose transporter | Unknown | Unknown | Bionumbers calculation | **8** | 0 | (Waygood and Steeves, 1980) |
| 3.30 x$10^{-1}$ | NaN | Bacteria | E. coli. | Lactose transporter | Unknown | Unknown | Bionumbers calculation | **8** | 0 | (Naftalin et al., 2007) |
| 8.30 x$10^{-1}$ | NaN | Bacteria | E. coli. | Lactose transporter | Unknown | Unknown | Bionumbers calculation | **8** | 0 | (Naftalin et al., 2007) |
| 3.28 | NaN | Yeast | Saccharomyces cerevisiae | HXT7 transporter | Unknown | Unknown | Bionumbers calculation | **8** | 0 | (Ye et al., 2001) |
| 1.13 x$10^{1}$ | | Primate | Baculovirus | MRP2 | 7 | 37 | | 512 | | (Yasunaga et al., 2008) |

**Table ST.10.20.1.1.2.** The log-normal distribution properties of the ABC $k_{cat}$ distribution. These values were calculated using the functions described in Section 2.6.2.

| Mode ($min^{-1}$) | Confidence Interval | Location parameter (μ) | Scale parameter (σ) |
|---|---|---|---|
| $1.13 \times 10^{1}$ | $1.00 \times 10^{1}$ | 3.22 | $8.91 \times 10^{-1}$ |

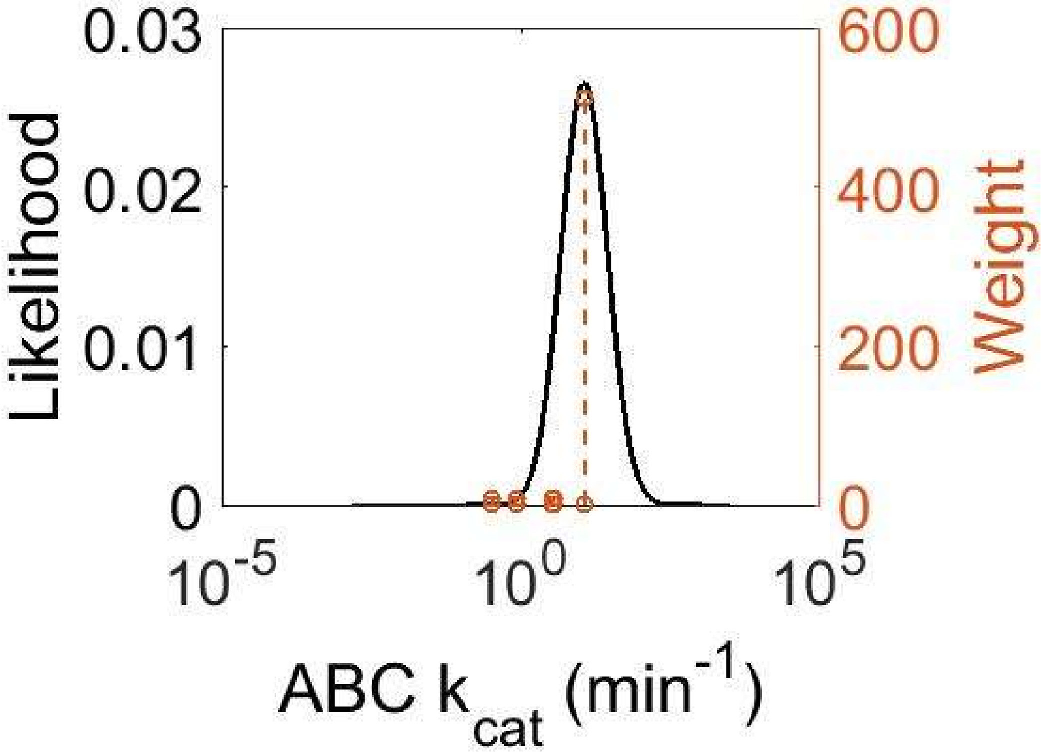


**Figure SF.10.20.1.1.1.** The estimated probability distribution for ABC $k_{cat}$. The value and weight of the literature values used to define the distribution are indicated by an orange dashed line. The x axis is plotted on a log-scale.

### S.10.20.1.2. Parameter: ABC $K_{ms}$

Parameter values for the ABC $K_{ms}$ of Reaction 41 were obtained from the literature and summarised in Table ST.10.20.1.2.1. The log-normal distribution properties for the ABC $K_{ms}$ of Reaction 41 are shown in Table ST.10.20.1.2.2 and plotted in Figure SF.10.20.1.2.1.

**Table ST.10.20.1.2.1.** Literature information used to design the ABC $K_{ms}$ parameter distribution. Each value was assigned a weight using the protocol described in Section 2.6.2. The type of error was assigned as 0 if it was additive and 1 if it was multiplicative.

| Value (mM) | Error (mM) | Experimental details | | | | | | Weight | Type of error | Reference |
|---|---|---|---|---|---|---|---|---|---|---|
| | | Species | Expression Vector | Enzyme | pH | Temperature (°C) | Other | | | |
| $1.09 \times 10^{-2}$ | $4.10 \times 10^{-3}$ | Human | Primate | MRP2 | 7 | 37 | | **512** | 0 | (Yasunaga et al., 2008) |
| $3.66 \times 10^{-5}$ | $3.80 \times 10^{-6}$ | Unknown | H69AR plasma membrane | MRP1 | 7.4 | 37 | | **128** | 0 | (Mao et al., 2000) |
| $5.30 \times 10^{-3}$ | $2.60 \times 10^{-3}$ | Human | HEK293 cells | MRP3 | 7.4 | 37 | | **128** | 0 | (Zeng et al., 2000) |
| $1.95 \times 10^{-1}$ | $6.12 \times 10^{-2}$ | Human | Primate | MRP2 | 7 | 37 | | **512** | 0 | (Yasunaga et al., 2008) |
| $1.10 \times 10^{-1}$ | $3.91 \times 10^{-2}$ | Human | Primate | MRP2 | 7 | 37 | | **512** | 0 | (Yasunaga et al., 2008) |
| $2.07 \times 10^{-2}$ | NaN | Human | HepG2 hepatoma | ABCB1 | 7.4 | 37 | | **2048** | 0 | (Fong et al., 2007) |

**Table ST.10.20.1.2.2.** The log-normal distribution properties of the ABC $K_{ms}$ distribution. These values were calculated using the functions described in Section 2.6.2.

| Mode (mM) | Confidence Interval | Location parameter (μ) | Scale parameter (σ) |
|---|---|---|---|
| 2.10 $x10^{-2}$ | 2.37 $x10^{1}$ | -2.60 | 1.12 |

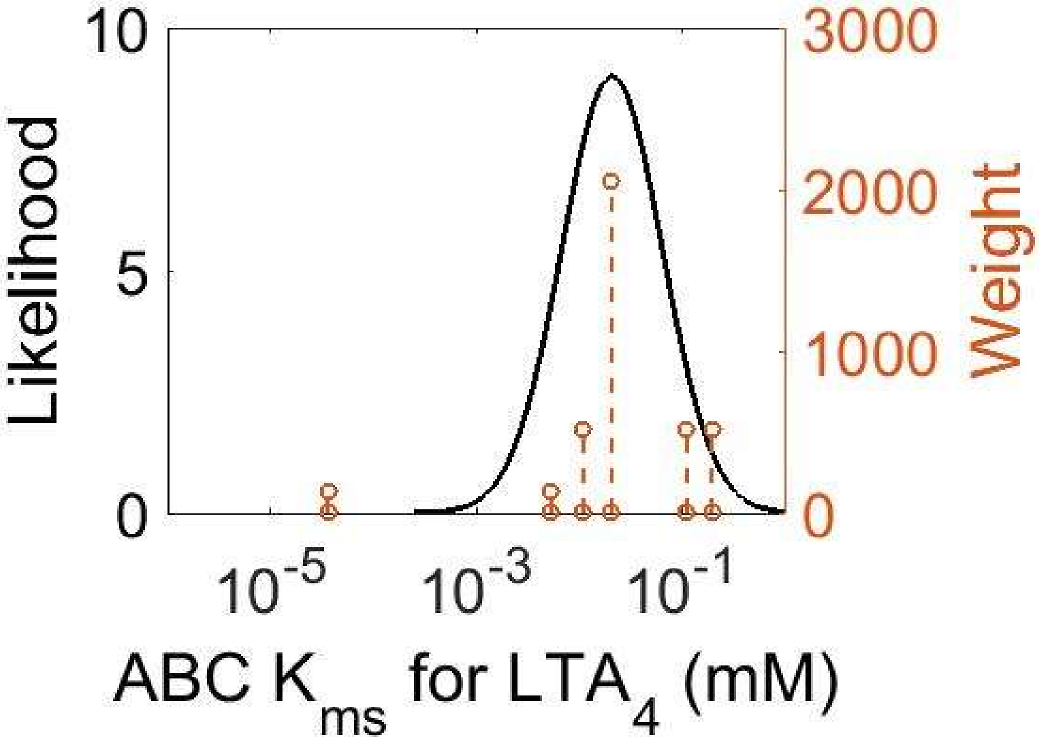


**Figure SF.10.20.1.2.1.** The estimated probability distribution for ABC $K_{ms}$. The value and weight of the literature values used to define the distribution are indicated by an orange dashed line. The x axis is plotted on a log-scale.

**S.10.20.1.3. Parameter: ABC concentration**

Parameter values for the ABC concentration of Reaction 41 were obtained from the literature and summarised in Table ST.10.20.1.3.1. The log-normal distribution properties for the ABC concentration of Reaction 41 are shown in Table ST.10.20.1.3.2 and plotted in Figure SF.10.20.1.3.1. To convert the enzyme concentration from ppm to mM, **Equation S.6.2** was used.

**Table ST.10.20.1.3.1.** Literature information used to design the ABC concentration parameter distribution. Each value was assigned a weight using the protocol described in Section 2.6.2. The type of error was assigned as 0 if it was additive and 1 if it was multiplicative.

| Value (ppm) | Error (ppm) | Experimental details | | | | | | Weight | Type of error | Reference |
|---|---|---|---|---|---|---|---|---|---|---|
| | | Species | Expression Vector | Enzyme | pH | Temperature (°C) | Other | | | |
| 5.15 | NaN | Human | Stomach | ABC | 7.5 | 37 | | 2048 | 0 | (Wilhelm et al., 2014) |
| 5.94 | NaN | Human | Lung | ABC | 7.5 | 37 | | 2048 | 0 | (Kim et al., 2014) |
| 2.66 | NaN | Human | Gut | ABC | 7.5 | 37 | | 2048 | 0 | (Kim et al., 2014) |

**Table ST.10.20.1.3.2.** The log-normal distribution properties of the ABC concentration distribution. These values were calculated using the functions described in Section 2.6.2.

| Mode (ppm) | Mode (mM) | Confidence Interval | Location parameter (μ) | Scale parameter (σ) |
|---|---|---|---|---|
| 5.06 | $2.80 \times 10^{-5}$ | 1.44 | 1.74 | 3.42 x10-1 |

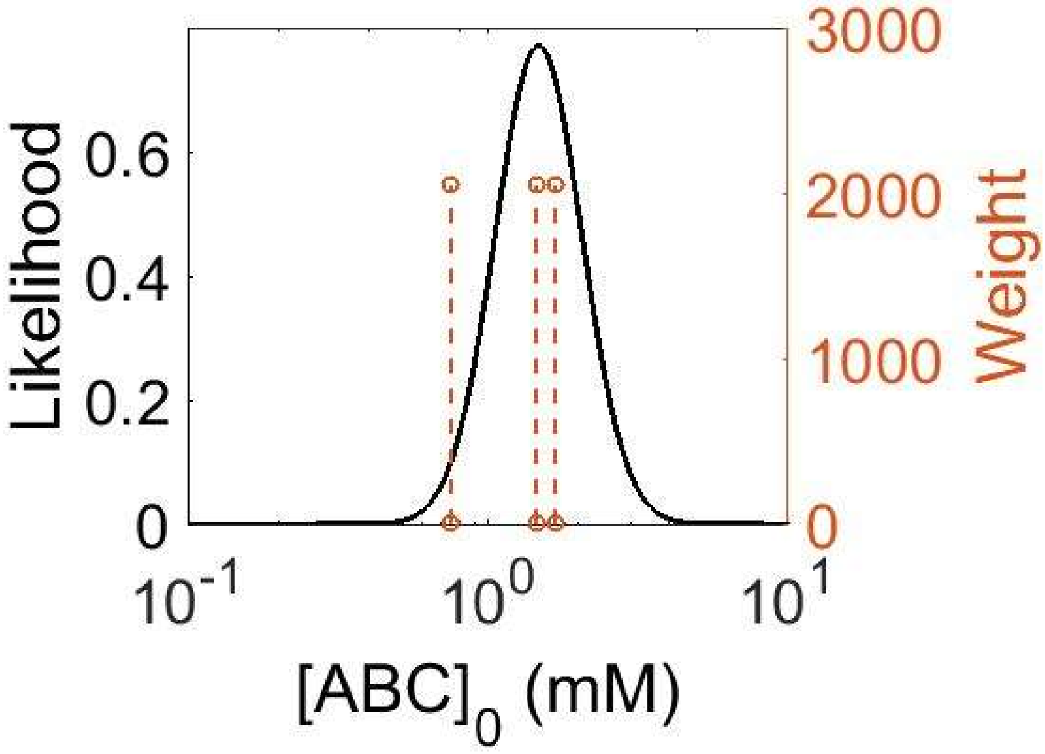


**Figure SF.10.20.1.3.1.** The estimated probability distribution for the ABC concentration. The value and weight of the literature values used to define the distribution are indicated by an orange dashed line. The x axis is plotted on a log-scale.

### S.10.21. Reaction 42: AA ⇌ exAA

AA is relocated from the intracellular compartment to the extracellular compartment via the ATP-binding cassette (ABC) transporter.

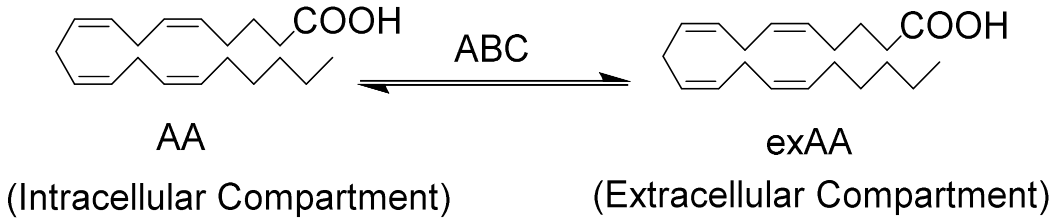


**Figure SF.10.21.** The transport of arachidonic acid (AA) from the intracellular compartment to the extracellular compartment by ATP Binding Cassette protein (ABC) (Reaction 42).

### SEq.10.21. Reaction rate law for Reaction 42.

$$\boldsymbol{v_{42}} = [\boldsymbol{ABC}] \cdot \boldsymbol{k_{cat}} \cdot \frac{\mathbf{AA}/K_m \cdot \left(1 - \frac{\mathbf{exAA}}{\mathbf{AA} \cdot e^{-0.5 \cdot \left(G + R \cdot T \cdot \ln\left(\frac{ATP}{ADP}\right)\right)/R \cdot T}}\right)}{1 + \frac{\mathbf{AA}}{K_m} + \frac{\mathbf{exAA}}{K_m} + \mathbf{ABC\_CI}}$$

### S.10.21.1. Reaction parameters

#### S.10.21.1.1. Parameter: ABC $k_{cat}$

Parameter values for the ABC $k_{cat}$ of Reaction 42 were obtained from the literature and summarised in Table ST.10.21.1.1.1. The log-normal distribution properties for the ABC $k_{cat}$ of Reaction 42 are shown in Table ST.10.21.1.1.2 and plotted in Figure SF.10.21.1.1.1.

**Table ST.10.21.1.1.1.** Literature information used to design the ABC $k_{cat}$ parameter distribution. Each value was assigned a weight using the protocol described in Section 2.6.2. The type of error was assigned as 0 if it was additive and 1 if it was multiplicative.

| Value ($min^{-1}$) | Error ($min^{-1}$) | Experimental details | | | | | | Weight | Type of error | Reference |
|---|---|---|---|---|---|---|---|---|---|---|
| | | Species | Expression Vector | Enzyme | pH | Temperature (°C) | Other | | | |
| 3.50 | NaN | Bacteria | E. coli. | Glucose transporter | Unknown | Unknown | Bionumbers calculation | **8** | 0 | (Waygood and Steeves, 1980) |
| 3.30 x$10^{-1}$ | NaN | Bacteria | E. coli. | Lactose transporter | Unknown | Unknown | Bionumbers calculation | **8** | 0 | (Naftalin et al., 2007) |
| 8.30 x$10^{-1}$ | NaN | Bacteria | E. coli. | Lactose transporter | Unknown | Unknown | Bionumbers calculation | **8** | 0 | (Naftalin et al., 2007) |
| 3.28 | NaN | Yeast | Saccharomyces cerevisiae | HXT7 transporter | Unknown | Unknown | Bionumbers calculation | **8** | 0 | (Ye et al., 2001) |
| 1.13 x$10^{1}$ | | Primate | Baculovirus | MRP2 | 7 | 37 | | 512 | | (Yasunaga et al., 2008) |

**Table ST.10.21.1.1.2.** The log-normal distribution properties of the ABC $k_{cat}$ distribution. These values were calculated using the functions described in Section 2.6.2.

| **Mode ($min^{-1}$)** | **Confidence Interval** | **Location parameter (μ)** | **Scale parameter (σ)** |
|---|---|---|---|
| 1.13 $x10^1$ | 1.00 $x10^1$ | 3.22 | $8.9110^{-1}$ |

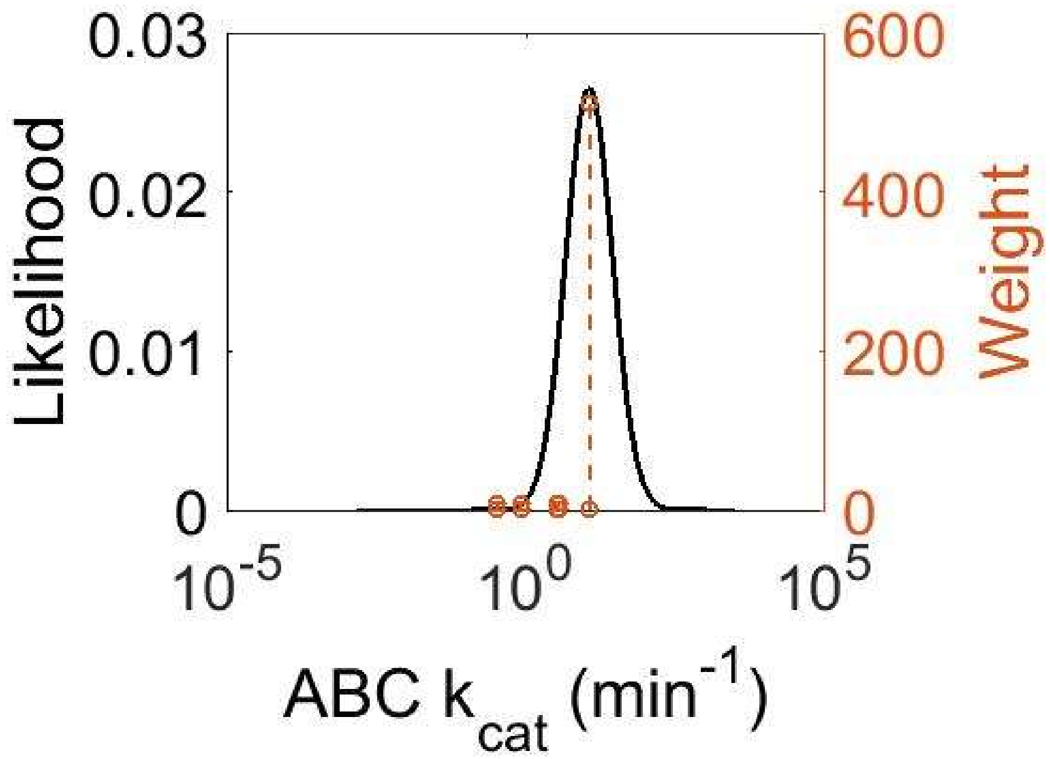


**Figure SF.10.21.1.1.1.** The estimated probability distribution for ABC $k_{cat}$. The value and weight of the literature values used to define the distribution are indicated by an orange dashed line. The x axis is plotted on a log-scale.

**S.10.21.1.2. Parameter: ABC $K_{ms}$**

In this model numerous simplifications have been made and as a result, metabolites, such as AA, occur in higher concentrations than are found in reality. To prevent a large percentage of AA being exported before it is metabolised, the $K_{ms}$ of the ABC transporter has been increased (mode= 25 mM, CIF=10, $\mu$ = 4.01, $\sigma$ = 0.89) (Table ST.10.21.1.2.1.). This is plotted in Figure SF.10.21.1.2.1.

**Table ST.10.21.1.2.1.** The log-normal distribution properties of the ABC $K_{ms}$ distribution. These values were calculated using the functions described in Section 2.6.2.

| Mode (mM) | Confidence Interval | Location parameter (μ) | Scale parameter (σ) |
|---|---|---|---|
| 25 | 10 | 4.01 | 0.89 |

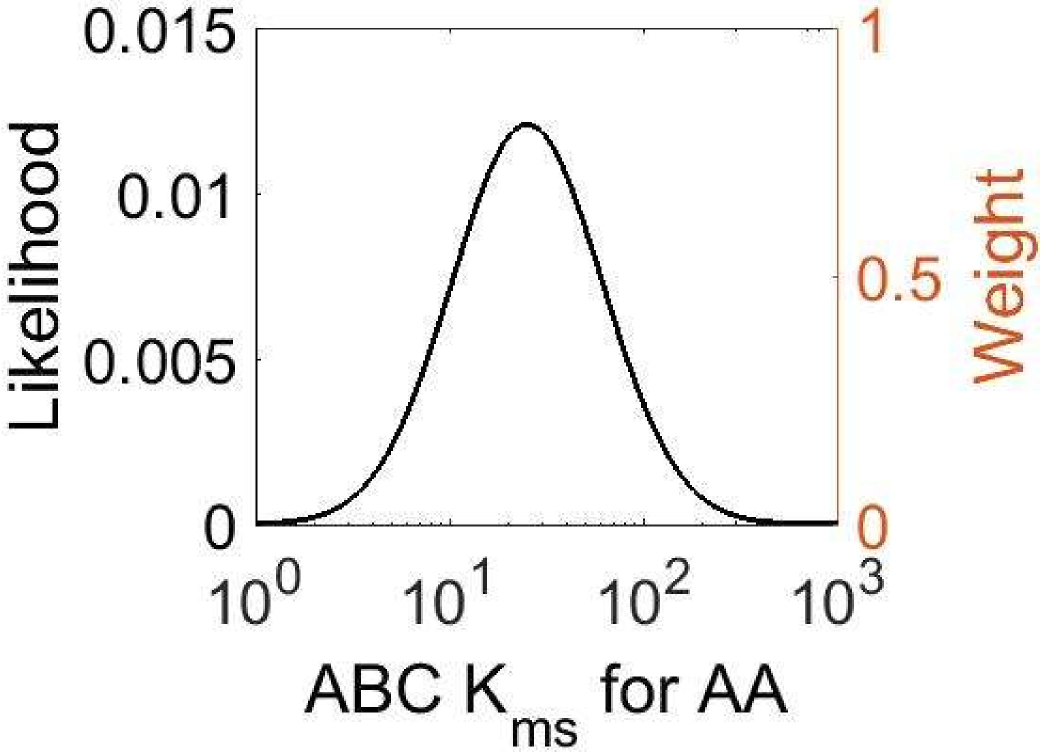


**Figure SF.10.21.1.2.1.** The estimated probability distribution for ABC $K_{ms}$. The value and weight of the literature values used to define the distribution are indicated by an orange dashed line. The x axis is plotted on a log-scale.

### S.10.21.1.3. Parameter: ABC concentration

Parameter values for the ABC concentration of Reaction 42 were obtained from the literature and summarised in Table ST.10.21.1.3.1. The log-normal distribution properties for the ABC concentration of Reaction 42 are shown in Table ST.10.21.1.3.2 and plotted in Figure SF.10.21.1.3.1. To convert the enzyme concentration from ppm to mM, **Equation S.6.2** was used.

**Table ST.10.21.1.3.1.** Literature information used to design the ABC concentration parameter distribution. Each value was assigned a weight using the protocol described in Section 2.6.2. The type of error was assigned as 0 if it was additive and 1 if it was multiplicative.

| Value (ppm) | Error (ppm) | Experimental details | | | | | | Weight | Type of error | Reference |
|---|---|---|---|---|---|---|---|---|---|---|
| | | Species | Expression Vector | Enzyme | pH | Temperature (°C) | Other | | | |
| 5.15 | NaN | Human | Stomach | ABC | 7.5 | 37 | | 2048 | 0 | (Wilhelm et al., 2014) |
| 5.94 | NaN | Human | Lung | ABC | 7.5 | 37 | | 2048 | 0 | (Kim et al., 2014) |
| 2.66 | NaN | Human | Gut | ABC | 7.5 | 37 | | 2048 | 0 | (Kim et al., 2014) |

**Table ST.10.21.1.3.2.** The log-normal distribution properties of the ABC concentration distribution. These values were calculated using the functions described in Section 2.6.2.

| Mode (ppm) | Mode (mM) | Confidence Interval | Location parameter (μ) | Scale parameter (σ) |
|---|---|---|---|---|
| 5.06 | $2.80 \times 10^{-5}$ | 1.44 | 1.74 | $3.42 \times 10^{-1}$ |

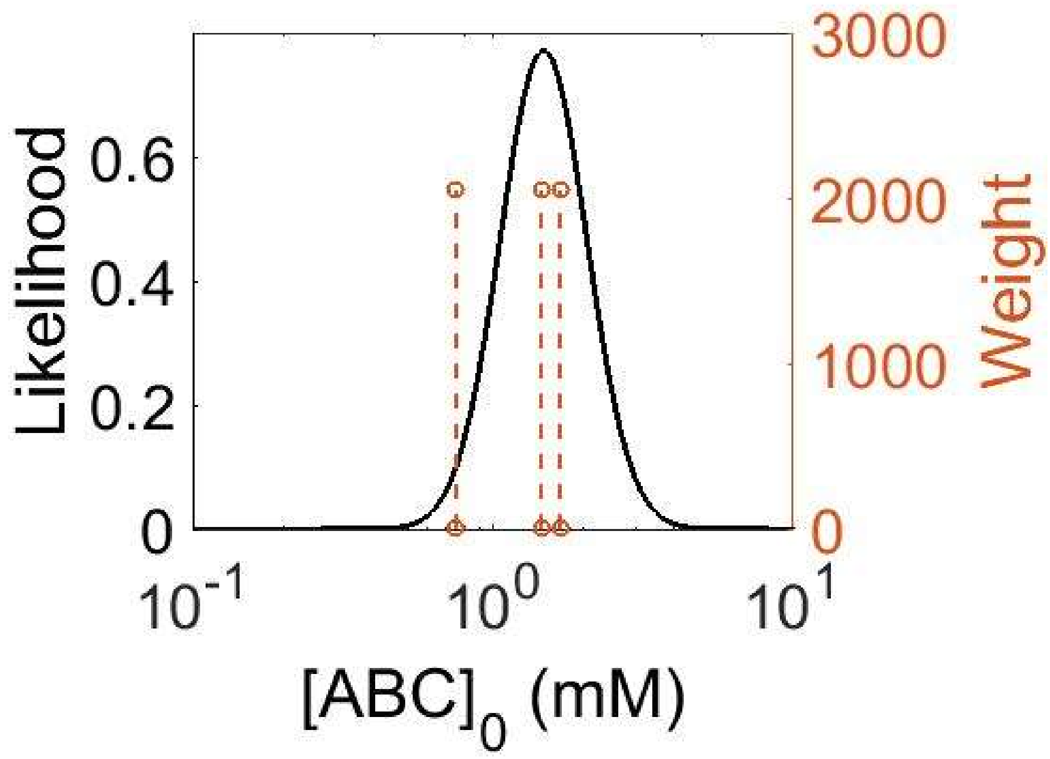


**Figure SF.10.21.1.3.1.** The estimated probability distribution for the ABC concentration. The value and weight of the literature values used to define the distribution are indicated by an orange dashed line. The x axis is plotted on a log-scale.

**S.10.22. Reaction 67: 15-keto-$PGE_2$ ⇌ ex15-keto-$PGE_2$**

15-keto-$PGE_2$ is relocated from the intracellular compartment to the extracellular compartment via the ATP-binding cassette (ABC) transporter.

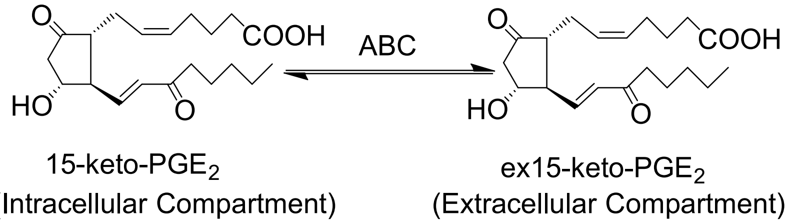


**Figure SF.10.22.** The transport of 15-keto-prostaglandin $E_2$ (15-keto-$PGE_2$) from the intracellular compartment to the extracellular compartment by ATP Binding Cassette protein (ABC) (Reaction 67).

**SEq.10.22. Reaction rate law for Reaction 67.**

$$v_{67} = [ABC] \cdot k_{cat} \cdot \frac{\text{15−keto−PGE}_2 / K_m \cdot \left( 1 - \frac{\text{ex15−keto−PGE}_2}{\text{15−keto−PGE}_2 \cdot e^{-0.5 \cdot \left( G + R \cdot T \cdot \ln\left(\frac{ATP}{ADP}\right)\right) / R \cdot T}} \right)}{1 + \frac{\text{15−keto−PGE}_2}{K_m} + \frac{ex\text{15−keto−PGE}_2}{K_m} + ABC\_CI}$$

### S.10.22.1. Reaction parameters

#### S.10.22.1.1. Parameter: ABC $k_{cat}$

Parameter values for the ABC $k_{cat}$ of Reaction 67 were obtained from the literature and summarised in Table ST.10.22.1.1.1. The log-normal distribution properties for the ABC $k_{cat}$ of Reaction 67 are shown in Table ST.10.22.1.1.2 and plotted in Figure SF.10.22.1.1.1.

**Table ST.10.22.1.1.1.** Literature information used to design the ABC $k_{cat}$ parameter distribution. Each value was assigned a weight using the protocol described in Section 2.6.2. The type of error was assigned as 0 if it was additive and 1 if it was multiplicative.

| Value ($min^{-1}$) | Error ($min^{-1}$) | Experimental details | | | | | | Weight | Type of error | Reference |
|---|---|---|---|---|---|---|---|---|---|---|
| | | Species | Expression Vector | Enzyme | pH | Temperature (°C) | Other | | | |
| 3.50 | NaN | Bacteria | E. coli. | Glucose transporter | Unknown | Unknown | Bionumbers calculation | **8** | 0 | (Waygood and Steeves, 1980) |
| $3.30 \times 10^{-1}$ | NaN | Bacteria | E. coli. | Lactose transporter | Unknown | Unknown | Bionumbers calculation | **8** | 0 | (Naftalin et al., 2007) |
| $8.30 \times 10^{-1}$ | NaN | Bacteria | E. coli. | Lactose transporter | Unknown | Unknown | Bionumbers calculation | **8** | 0 | (Naftalin et al., 2007) |
| 3.28 | NaN | Yeast | Saccharomyces cerevisiae | HXT7 transporter | Unknown | Unknown | Bionumbers calculation | **8** | 0 | (Ye et al., 2001) |
| $1.13 \times 10^{1}$ | | Primate | Baculovirus | MRP2 | 7 | 37 | | 512 | | (Yasunaga et al., 2008) |

**Table ST.10.22.1.1.2.** The log-normal distribution properties of the ABC $k_{cat}$ distribution. These values were calculated using the functions described in Section 2.6.2.

| **Mode ($min^{-1}$)** | **Confidence Interval** | **Location parameter (μ)** | **Scale parameter (σ)** |
|---|---|---|---|
| $1.14 \times 10^{1}$ | 9.86 | 3.22 | $8.87 \times 10^{-1}$ |

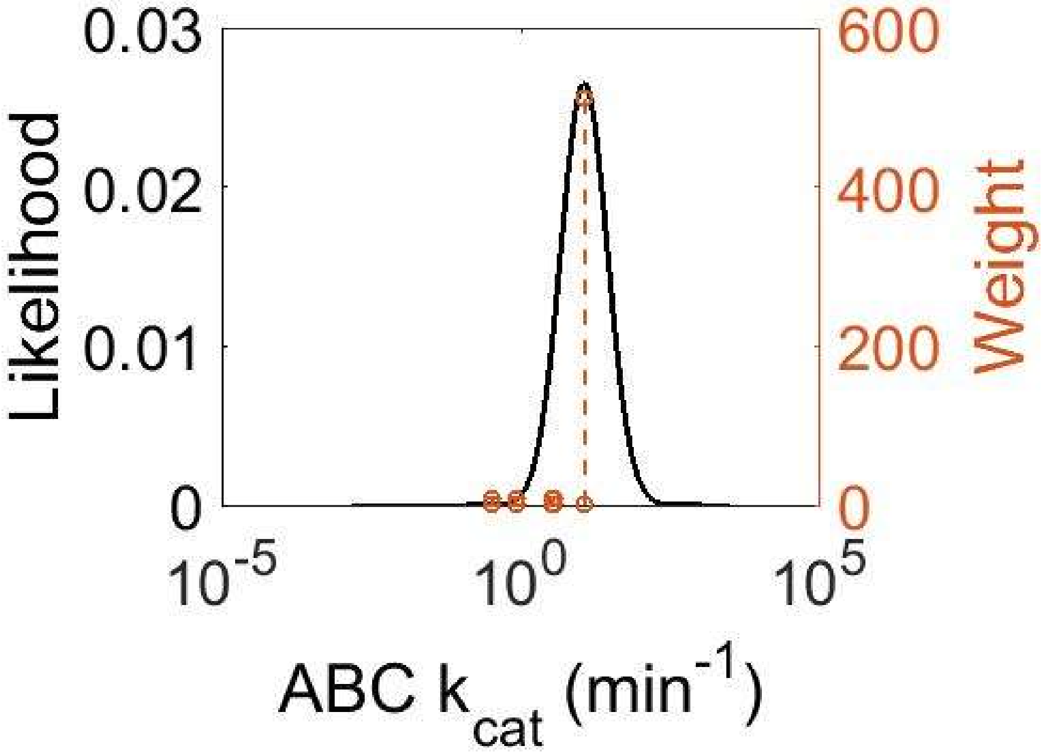


**Figure SF.10.22.1.1.1.** The estimated probability distribution for ABC $k_{cat}$. The value and weight of the literature values used to define the distribution are indicated by an orange dashed line. The x axis is plotted on a log-scale.

### S.10.22.1.2. Parameter: ABC $K_{ms}$

Parameter values for the ABC $K_{ms}$ of Reaction 67 were obtained from the literature and summarised in Table ST.10.22.1.2.1. The log-normal distribution properties for the ABC $K_{ms}$ of Reaction 67 are shown in Table ST.10.22.1.2.2 and plotted in Figure SF.10.22.1.2.1.

**Table ST.10.22.1.2.1.** Literature information used to design the ABC $K_{ms}$ parameter distribution. Each value was assigned a weight using the protocol described in Section 2.6.2. The type of error was assigned as 0 if it was additive and 1 if it was multiplicative.

| Value (mM) | Error (mM) | Experimental details | | | | | | Weight | Type of error | Reference |
|---|---|---|---|---|---|---|---|---|---|---|
| | | Species | Expression Vector | Enzyme | pH | Temperature (°C) | Other | | | |
| $1.09 \times 10^{-2}$ | $4.10 \times 10^{-3}$ | Human | Primate | MRP2 | 7 | 37 | | **512** | 0 | (Yasunaga et al., 2008) |
| $3.66 \times 10^{-5}$ | $3.80 \times 10^{-6}$ | Unknown | H69AR plasma membrane | MRP1 | 7.4 | 37 | | **128** | 0 | (Mao et al., 2000) |
| $5.30 \times 10^{-3}$ | $2.60 \times 10^{-3}$ | Human | HEK293 cells | MRP3 | 7.4 | 37 | | **128** | 0 | (Zeng et al., 2000) |
| $1.95 \times 10^{-1}$ | $6.12 \times 10^{-2}$ | Human | Primate | MRP2 | 7 | 37 | | **512** | 0 | (Yasunaga et al., 2008) |
| $1.10 \times 10^{-1}$ | $3.91 \times 10^{-2}$ | Human | Primate | MRP2 | 7 | 37 | | **512** | 0 | (Yasunaga et al., 2008) |
| $2.07 \times 10^{-2}$ | NaN | Human | HepG2 hepatoma | ABCB1 | 7.4 | 37 | | **2048** | 0 | (Fong et al., 2007) |

**Table ST.10.22.1.2.2.** The log-normal distribution properties of the ABC $K_{ms}$ distribution. These values were calculated using the functions described in Section 2.6.2.

| Mode (mM) | Confidence Interval | Location parameter (μ) | Scale parameter (σ) |
|---|---|---|---|
| $1.20 \times 10^{-2}$ | 4.92 | -3.97 | $8.7110^{-1}$ |

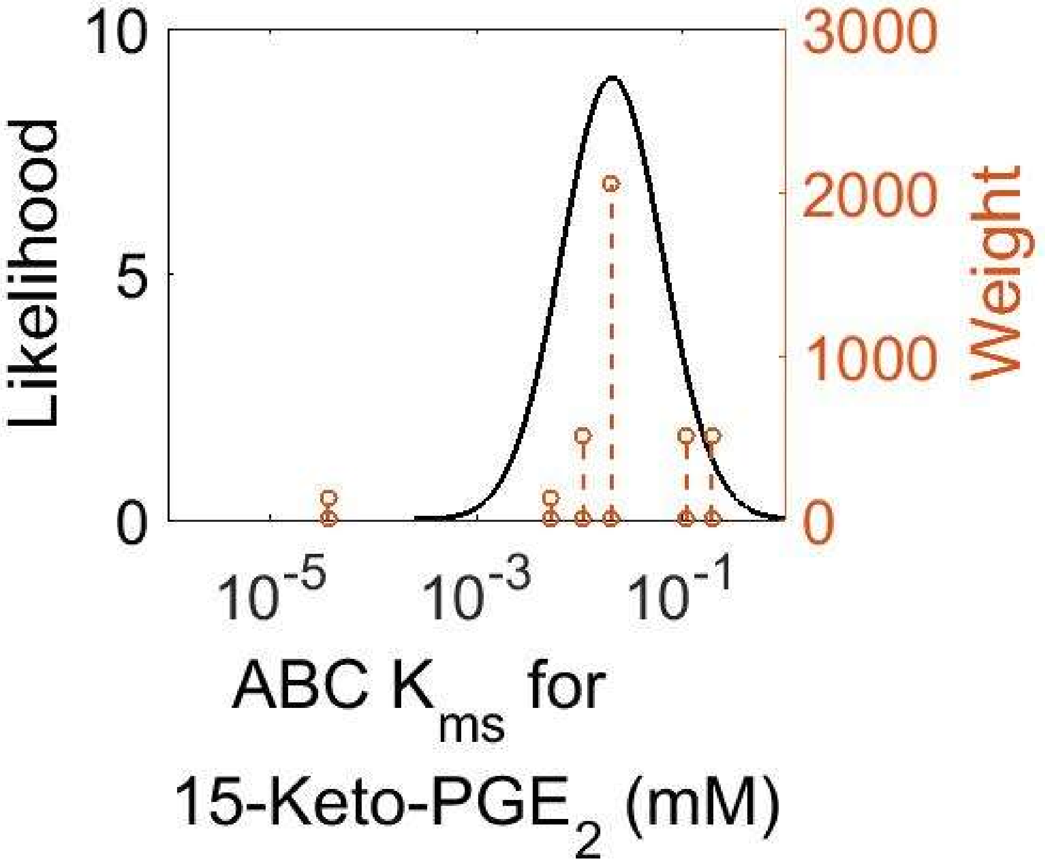


**Figure SF.10.22.1.2.1.** The estimated probability distribution for ABC $K_{ms}$. The value and weight of the literature values used to define the distribution are indicated by an orange dashed line. The x axis is plotted on a log-scale.

### S.10.22.1.3. Parameter: ABC concentration

Parameter values for the ABC concentration of Reaction 67 were obtained from the literature and summarised in Table ST.10.22.1.3.1. The log-normal distribution properties for the ABC concentration of Reaction 67 are shown in Table ST.10.22.1.3.2 and plotted in Figure SF.10.22.1.3.1. To convert the enzyme concentration from ppm to mM, **Equation S.6.2** was used.

**Table ST.10.22.1.3.1.** Literature information used to design the ABC concentration parameter distribution. Each value was assigned a weight using the protocol described in Section 2.6.2. The type of error was assigned as 0 if it was additive and 1 if it was multiplicative.

| Value (ppm) | Error (ppm) | Experimental details | | | | | | Weight | Type of error | Reference |
|---|---|---|---|---|---|---|---|---|---|---|
| | | Species | Expression Vector | Enzyme | pH | Temperature (°C) | Other | | | |
| 5.15 | NaN | Human | Stomach | ABC | 7.5 | 37 | | 2048 | 0 | (Wilhelm et al., 2014) |
| 5.94 | NaN | Human | Lung | ABC | 7.5 | 37 | | 2048 | 0 | (Kim et al., 2014) |
| 2.66 | NaN | Human | Gut | ABC | 7.5 | 37 | | 2048 | 0 | (Kim et al., 2014) |

**Table ST.10.22.1.3.2.** The log-normal distribution properties of the ABC concentration distribution. These values were calculated using the functions described in Section 2.6.2.

| Mode (ppm) | Mode (mM) | Confidence Interval | Location parameter (μ) | Scale parameter (σ) |
|---|---|---|---|---|
| 5.06 | $2.80 \times 10^{-5}$ | 1.44 | 1.74 | $3.42 \times 10^{-1}$ |

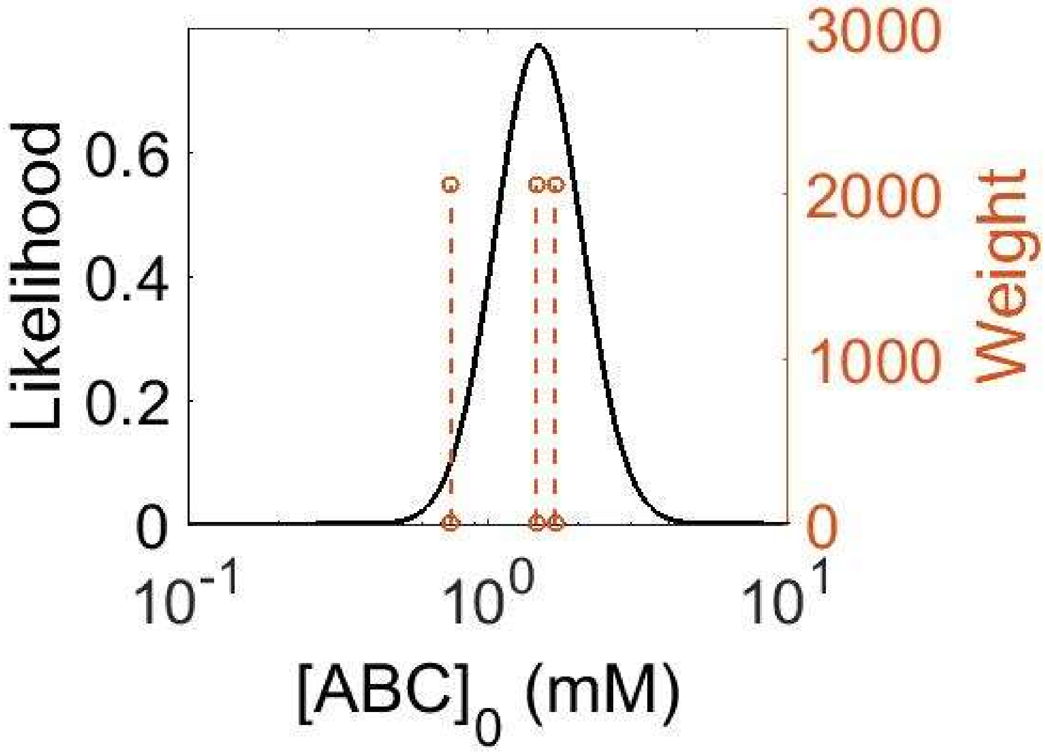


**Figure SF.10.22.1.3.1.** The estimated probability distribution for the ABC concentration. The value and weight of the literature values used to define the distribution are indicated by an orange dashed line. The x axis is plotted on a log-scale.

**S.10.23. Reaction 70: 13,14-dihydro-15-keto-PGE₂ ⇌ ex13,14-dihydro-15-keto-PGE₂**

13,14-dihydro-15-keto-$PGE_2$ is relocated from the intracellular compartment to the extracellular compartment via the ATP-binding cassette (ABC) transporter.

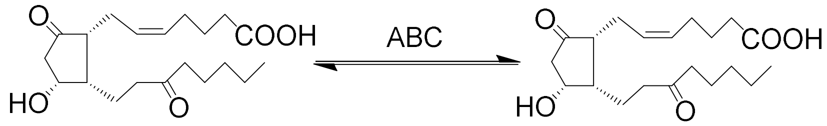


13,14-dihydro-15-keto-$PGE_2$ (Intracellular Compartment) ⇌ ex13,14-dihydro-15-keto-$PGE_2$ (Extracellular Compartment)

**Figure SF.10.23.** The transport of 13,14-dihydro-15-keto-prostaglandin $E_2$ (13,14-dihydro-15-keto-$PGE_2$) from the intracellular compartment to the extracellular compartment by ATP Binding Cassette protein (ABC) (Reaction 70).

**SEq.10.23.** Reaction rate law for Reaction 70.

$$\boldsymbol{v_{70} = [ABC] \cdot k_{cat}}$$

$$\cdot \frac{\mathbf{13,14-dihydro-15-keto-PGE_2} / K_m \cdot \left(1 - \frac{\mathbf{ex13,14-dihydro-15-keto-PGE_2}}{\mathbf{13,14-dihydro-15-keto-PGE_2} \cdot e^{-0.5\cdot\left(G+R\cdot T\cdot \ln\left(\frac{ATP}{ADP}\right)\right)/R\cdot T}}\right)}{1 + \frac{\mathbf{13,14-dihydro-15-keto-PGE_2}}{K_m} + \frac{\mathbf{ex13,14-dihydro-15-keto-PGE_2}}{K_m} + ABC\_CI}$$

## S.10.23.1. Reaction parameters

### S.10.23.1.1. Parameter: ABC $k_{cat}$

Parameter values for the ABC $k_{cat}$ of Reaction 70 were obtained from the literature and summarised in Table ST.10.23.1.1.1. The log-normal distribution properties for the ABC $k_{cat}$ of Reaction 70 are shown in Table ST.10.23.1.1.2 and plotted in Figure SF.10.23.1.1.1.

**Table ST.10.23.1.1.1.** Literature information used to design the ABC $k_{cat}$ parameter distribution. Each value was assigned a weight using the protocol described in Section 2.6.2. The type of error was assigned as 0 if it was additive and 1 if it was multiplicative.

| Value ($min^{-1}$) | Error ($min^{-1}$) | Experimental details | | | | | | Weight | Type of error | Reference |
|---|---|---|---|---|---|---|---|---|---|---|
| | | Species | Expression Vector | Enzyme | pH | Temperature (°C) | Other | | | |
| 3.50 | NaN | Bacteria | E. coli. | Glucose transporter | Unknown | Unknown | Bionumbers calculation | **8** | 0 | (Waygood and Steeves, 1980) |
| $3.30 \times 10^{-1}$ | NaN | Bacteria | E. coli. | Lactose transporter | Unknown | Unknown | Bionumbers calculation | **8** | 0 | (Naftalin et al., 2007) |
| $8.30 \times 10^{-1}$ | NaN | Bacteria | E. coli. | Lactose transporter | Unknown | Unknown | Bionumbers calculation | **8** | 0 | (Naftalin et al., 2007) |
| 3.28 | NaN | Yeast | Saccharomyces cerevisiae | HXT7 transporter | Unknown | Unknown | Bionumbers calculation | **8** | 0 | (Ye et al., 2001) |
| $1.13 \times 10^{1}$ | | Primate | Baculovirus | MRP2 | 7 | 37 | | 512 | | (Yasunaga et al., 2008) |

**Table ST.10.23.1.1.2.** The log-normal distribution properties of the ABC $k_{cat}$ distribution. These values were calculated using the functions described in Section 2.6.2.

| Mode ($min^{-1}$) | Confidence Interval | Location parameter ($\mu$) | Scale parameter ($\sigma$) |
|---|---|---|---|
| $1.13 \times 10^{1}$ | $1.00 \times 10^{1}$ | 3.22 | $8.91 \times 10^{-1}$ |

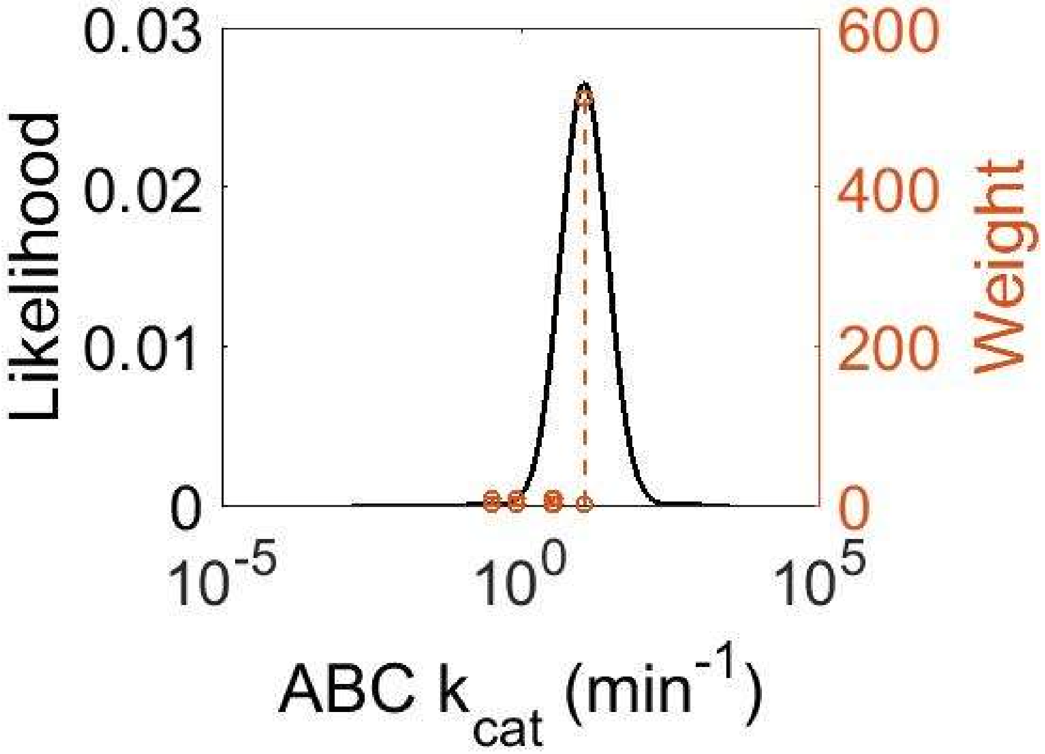


**Figure SF.10.23.1.1.1.** The estimated probability distribution for ABC $k_{cat}$. The value and weight of the literature values used to define the distribution are indicated by an orange dashed line. The x axis is plotted on a log-scale.

### S.10.23.1.2. Parameter: ABC $K_{ms}$

Parameter values for the ABC $K_{ms}$ of Reaction 70 were obtained from the literature and summarised in Table ST.10.23.1.2.1. The log-normal distribution properties for the ABC $K_{ms}$ of Reaction 70 are shown in Table ST.10.23.1.2.2 and plotted in Figure SF.10.23.1.2.1.

**Table ST.10.23.1.2.1.** Literature information used to design the ABC $K_{ms}$ parameter distribution. Each value was assigned a weight using the protocol described in Section 2.6.2. The type of error was assigned as 0 if it was additive and 1 if it was multiplicative.

| Value (mM) | Error (mM) | Experimental details | | | | | | Weight | Type of error | Reference |
|---|---|---|---|---|---|---|---|---|---|---|
| | | Species | Expression Vector | Enzyme | pH | Temperature (°C) | Other | | | |
| $1.09 \times 10^{-2}$ | $4.10 \times 10^{-3}$ | Human | Primate | MRP2 | 7 | 37 | | **512** | 0 | (Yasunaga et al., 2008) |
| $3.66 \times 10^{-5}$ | $3.80 \times 10^{-6}$ | Unknown | H69AR plasma membrane | MRP1 | 7.4 | 37 | | **128** | 0 | (Mao et al., 2000) |
| $5.30 \times 10^{-3}$ | $2.60 \times 10^{-3}$ | Human | HEK293 cells | MRP3 | 7.4 | 37 | | **128** | 0 | (Zeng et al., 2000) |
| $1.95 \times 10^{-1}$ | $6.12 \times 10^{-2}$ | Human | Primate | MRP2 | 7 | 37 | | **512** | 0 | (Yasunaga et al., 2008) |
| $1.10 \times 10^{-1}$ | $3.91 \times 10^{-2}$ | Human | Primate | MRP2 | 7 | 37 | | **512** | 0 | (Yasunaga et al., 2008) |
| $2.07 \times 10^{-2}$ | NaN | Human | HepG2 hepatoma | ABCB1 | 7.4 | 37 | | **2048** | 0 | (Fong et al., 2007) |

**Table ST.10.23.1.2.2.** The log-normal distribution properties of the ABC $K_{ms}$ distribution. These values were calculated using the functions described in Section 2.6.2.

| Mode (mM) | Confidence Interval | Location parameter (μ) | Scale parameter (σ) |
|---|---|---|---|
| $2.10 \times 10^{-2}$ | $2.37 \times 10^{1}$ | -2.60 | 1.12 |

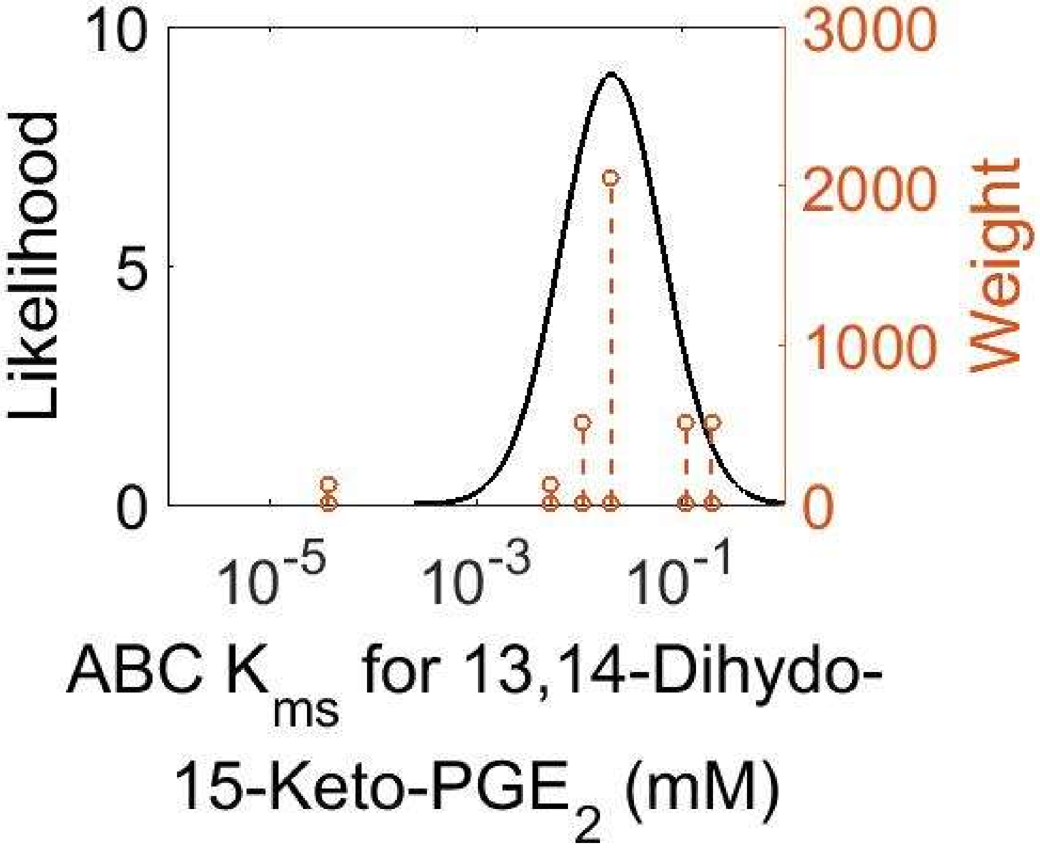


**Figure SF.10.23.1.2.1.** The estimated probability distribution for ABC $K_{ms}$. The value and weight of the literature values used to define the distribution are indicated by an orange dashed line. The x axis is plotted on a log-scale.

**S.10.23.1.3. Parameter: ABC concentration**

Parameter values for the ABC concentration of Reaction 70 were obtained from the literature and summarised in Table ST.10.23.1.3.1. The log-normal distribution properties for the ABC concentration of Reaction 70 are shown in Table ST.10.23.1.3.2 and plotted in Figure SF.10.23.1.3.1. To convert the enzyme concentration from ppm to mM, **Equation S.6.2** was used.

**Table ST.10.23.1.3.1.** Literature information used to design the ABC concentration parameter distribution. Each value was assigned a weight using the protocol described in Section 2.6.2. The type of error was assigned as 0 if it was additive and 1 if it was multiplicative.

| Value (ppm) | Error (ppm) | Experimental details | | | | | | Weight | Type of error | Reference |
|---|---|---|---|---|---|---|---|---|---|---|
| | | Species | Expression Vector | Enzyme | pH | Temperature (°C) | Other | | | |
| 5.15 | NaN | Human | Stomach | ABC | 7.5 | 37 | | 2048 | 0 | (Wilhelm et al., 2014) |
| 5.94 | NaN | Human | Lung | ABC | 7.5 | 37 | | 2048 | 0 | (Kim et al., 2014) |
| 2.66 | NaN | Human | Gut | ABC | 7.5 | 37 | | 2048 | 0 | (Kim et al., 2014) |

**Table ST.10.23.1.3.2.** The log-normal distribution properties of the ABC concentration distribution. These values were calculated using the functions described in Section 2.6.2.

| Mode (ppm) | Mode (mM) | Confidence Interval | Location parameter (μ) | Scale parameter (σ) |
|---|---|---|---|---|
| 5.06 | $2.80 \times 10^{-5}$ | 1.44 | 1.74 | $3.42 \times 10^{-1}$ |

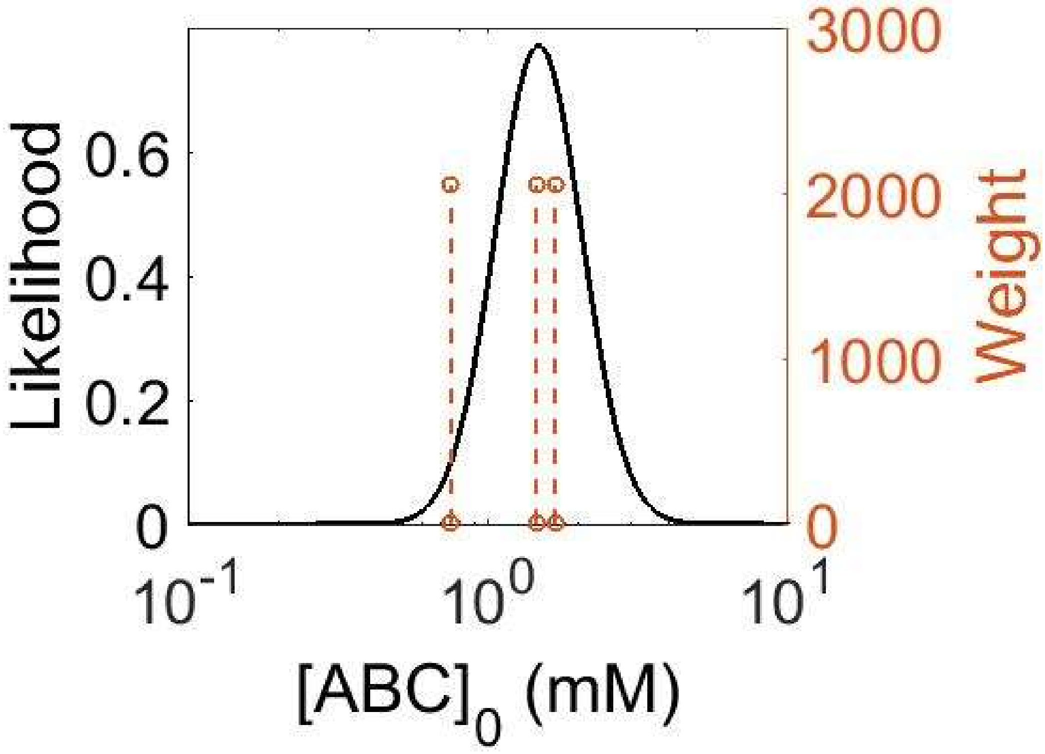


**Figure SF.10.23.1.3.1.** The estimated probability distribution for the ABC concentration. The value and weight of the literature values used to define the distribution are indicated by an orange dashed line. The x axis is plotted on a log-scale.

**S.10.24. Reaction 101: $PGF_{2\alpha} \rightleftharpoons exPGF_{2\alpha}$ (PGT)**

$PGF_{2\alpha}$ is relocated from the intracellular compartment to the extracellular compartment via the prostaglandin (PGT) transporter.

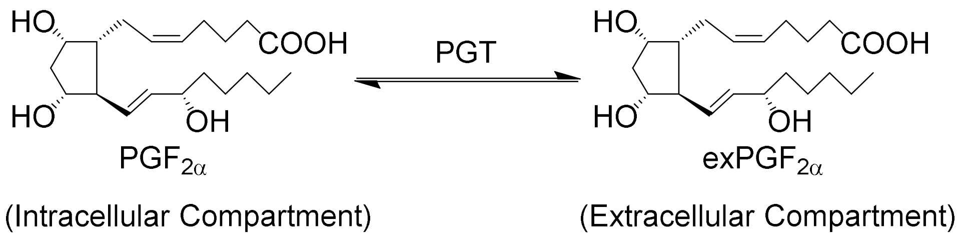


**Figure SF.10.24.** The transport of prostaglandin $F_{2\alpha}$ ($PGF_{2\alpha}$) from the intracellular compartment to the extracellular compartment by prostaglandin (PGT) transporter (Reaction 101).

**SEq.10.24. Reaction rate law for Reaction 101.**

$$\boldsymbol{v_{101}} = [\boldsymbol{PGT}] \cdot \boldsymbol{k_{cat}} \cdot \frac{{}^{PGF_{2\alpha}}/_{K_m} \cdot \left(1 - \frac{exPGF_{2\alpha}}{PGF_{2\alpha} \cdot e^{-0.5 \cdot \left(G + R \cdot T \cdot \ln\left(\frac{ATP}{ADP}\right)\right)/_{R \cdot T}}}\right)}{1 + \frac{PGF_{2\alpha}}{K_m} + \frac{exPGF_{2\alpha}}{K_m} + \text{PGT\_CI}}$$

## S.10.24.1. Reaction parameters

### S.10.24.1.1. Parameter: PGT $k_{cat}$

Parameter values for the PGT $k_{cat}$ of Reaction 101 were obtained from the literature and summarised in Table ST.10.24.1.1.1. The log-normal distribution properties for the PGT $k_{cat}$ of Reaction 101 are shown in Table ST.10.24.1.1.2 and plotted in Figure SF.10.24.1.1.1.

**Table ST.10.24.1.1.1.** Literature information used to design the PGT $k_{cat}$ parameter distribution. Each value was assigned a weight using the protocol described in Section 2.6.2. The type of error was assigned as 0 if it was additive and 1 if it was multiplicative.

| Value ($min^{-1}$) | Error ($min^{-1}$) | Experimental details | | | | | | Weight | Type of error | Reference |
|---|---|---|---|---|---|---|---|---|---|---|
| | | Species | Expression Vector | Enzyme | pH | Temperature (°C) | Other | | | |
| 3.50 | NaN | Bacteria | E. coli. | Glucose transporter | Unknown | Unknown | Bionumbers calculation | **8** | 0 | (Waygood and Steeves, 1980) |
| 3.30 x$10^{-1}$ | NaN | Bacteria | E. coli. | Lactose transporter | Unknown | Unknown | Bionumbers calculation | **8** | 0 | (Naftalin et al., 2007) |
| 8.30 x$10^{-1}$ | NaN | Bacteria | E. coli. | Lactose transporter | Unknown | Unknown | Bionumbers calculation | **8** | 0 | (Naftalin et al., 2007) |
| 3.28 | NaN | Yeast | Saccharomyces cerevisiae | HXT7 transporter | Unknown | Unknown | Bionumbers calculation | **8** | 0 | (Ye et al., 2001) |

**Table ST.10.24.1.1.2.** The log-normal distribution properties of the PGT $k_{cat}$ distribution. These values were calculated using the functions described in Section 2.6.2.

| **Mode ($min^{-1}$)** | **Confidence Interval** | **Location parameter (μ)** | **Scale parameter (σ)** |
|---|---|---|---|
| 1.33 | 7.30 | $9.20 \times 10^{-1}$ | $8.0 \times 10^{-1}$ |

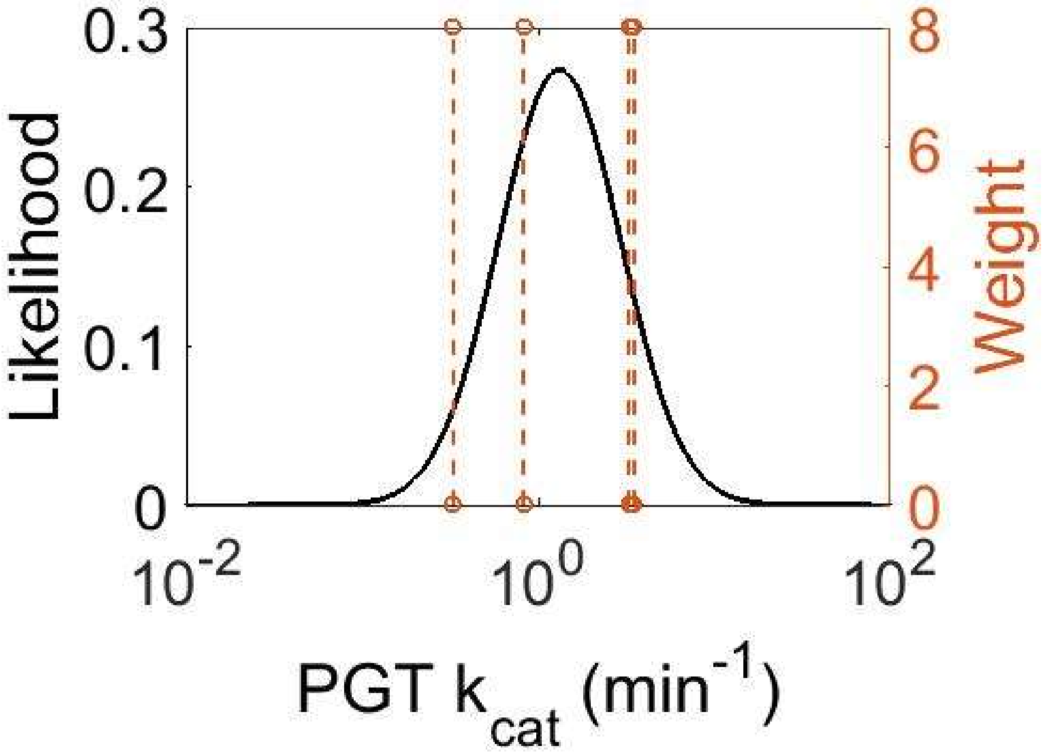


**Figure SF.10.24.1.1.1.** The estimated probability distribution for the PGT $k_{cat}$. The value and weight of the literature values used to define the distribution are indicated by an orange dashed line. The x axis is plotted on a log-scale.

**S.10.24.1.2. Parameter: PGT $K_{ms}$**

Parameter values for the PGT $K_{ms}$ of Reaction 101 were obtained from the literature and summarised in Table ST.10.24.1.2.1. The log-normal distribution properties for the PGT $K_{ms}$ of Reaction 101 are shown in Table ST.10.24.1.2.2 and plotted in Figure SF.10.24.1.2.1.

**Table ST.10.24.1.2.1.** Literature information used to design the PGT $K_{ms}$ parameter distribution. Each value was assigned a weight using the protocol described in Section 2.6.2. The type of error was assigned as 0 if it was additive and 1 if it was multiplicative.

| Value (mM) | Error (mM) | Experimental details | | | | | | | Weight | Type of error | Reference |
|---|---|---|---|---|---|---|---|---|---|---|---|
| | | Substrate | Species | Expression Vector | Enzyme | pH | Temperature (°C) | Other | | | |
| $3.31 \times 10^{-5}$ | $1.31 \times 10^{-4}$ | | Human | Eye | PGT | 7.5 | 37 | | 512 | 0 | (Gose et al., 2016) |
| $3.76 \times 10^{-4}$ | $3.40 \times 10^{-5}$ | | Human | Embryonic kidney cells (HEK293) | PGT | Unknown | 37 | | 192 | 0 | (Chi and Schuster, 2010) |
| $5.40 \times 10^{-3}$ | NaN | | Human | Embryonic kidney cells (HEK293) | PGT | Unknown | 37 | | 2048 | 0 | (Kraft et al., 2010) |
| $7.20 \times 10^{-3}$ | $5.95 \times 10^{-4}$ | | Canine | Madin-Darby Canine Kidney Epithelial Cells (MDCK) cells | PGT | Unknown | Unknown | | 512 | 0 | (Gose et al., 2016) |

| | | | | | | | | | | | |
|---|---|---|---|---|---|---|---|---|---|---|---|
| $9.40 \times 10^{-5}$ | NaN | PGE2 | Human | HeLa cells | PGT | 7 | 27 | | **1024** | 0 | (Kanai et al., 1995) |
| $4.23 \times 10^{-4}$ | NaN | TXB2 | Human | HeLa cells | PGT | 7 | 27 | | **1024** | 0 | (Kanai et al., 1995) |
| $7.57 \times 10^{-3}$ | NaN | 6-keto-PGF1a | Human | HeLa cells | PGT | 7 | 27 | | **1024** | 0 | (Kanai et al., 1995) |
| $4.87 \times 10^{-5}$ | NaN | PGE2 | Human | HeLa cells | PGT | 7 | 27 | | **1024** | 0 | (Itoh et al., 1996) |
| $5.02 \times 10^{-5}$ | NaN | PGF2a | Human | HeLa cells | PGT | 7 | 27 | | **1024** | 0 | (Itoh et al., 1996 |
| $5.00 \times 10^{-3}$ | NaN | 15-keto-PGE2 | Human | HeLa cells | PGT | 7 | 27 | | **1024** | 0 | (Itoh et al., 1996 |
| $5.00 \times 10^{-3}$ | NaN | 13,14-dihydro-15-keto-PGE2 | Human | HeLa cells | PGT | 7 | 27 | | **1024** | 0 | (Itoh et al., 1996 |
| $3.82 \times 10^{-5}$ | NaN | PGH2 | Human | HeLa cells | PGT | 7 | 27 | | **2048** | 0 | (Itoh et al., 1996 |

**Table ST.10.24.1.2.2.** The log-normal distribution properties of the PGT $K_{ms}$ distribution. These values were calculated using the functions described in Section 2.6.2.

| **Mode (mM)** | **Confidence Interval** | **Location parameter (μ)** | **Scale parameter (σ)** |
|---|---|---|---|
| $1.10 \times 10^{-3}$ | $9.42 \times 10^{1}$ | -4.67 | 1.45 |

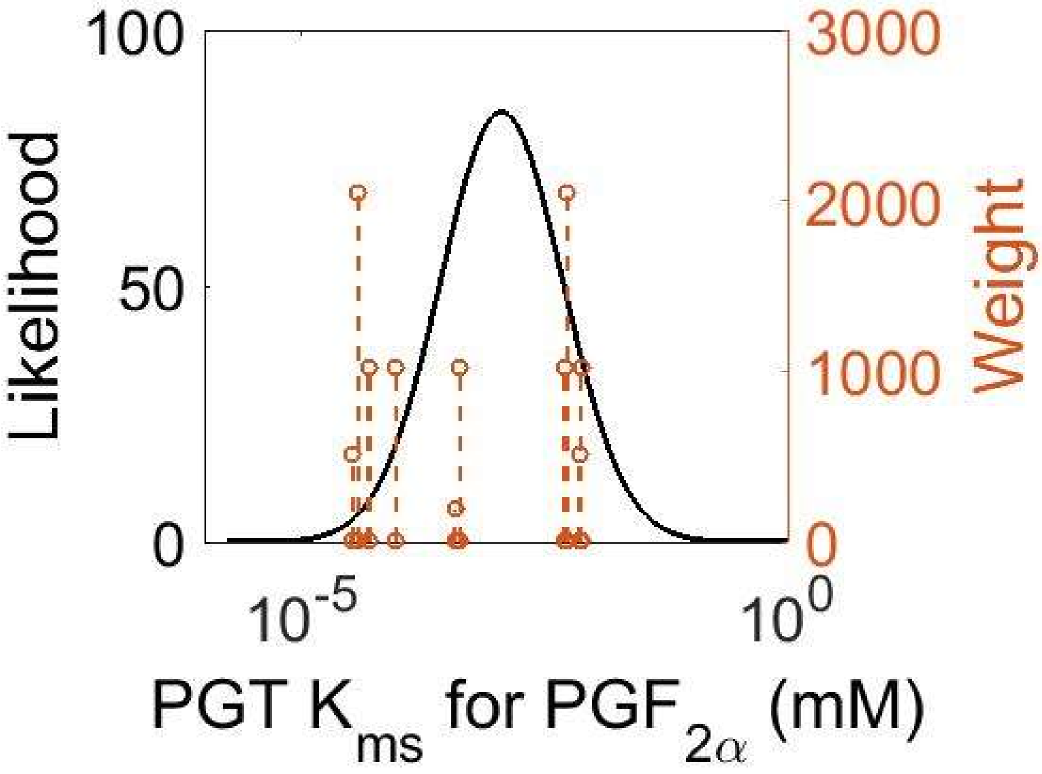


**Figure SF.10.24.1.2.1.** The estimated probability distribution for the PGT $K_{ms}$. The value and weight of the literature values used to define the distribution are indicated by an orange dashed line. The x axis is plotted on a log-scale.

**S.10.24.1.3. Parameter: PGT concentration**

Parameter values for the PGT concentration of Reaction 101 were obtained from the literature and summarised in Table ST.10.24.1.3.1. The log-normal distribution properties for the PGT concentration of Reaction 101 are shown in Table ST.10.24.1.3.2 and plotted in Figure SF.10.24.1.3.1. To convert the enzyme concentration from ppm to mM, **Equation S.6.2** was used.

**Table ST.10.24.1.3.1.** Literature information used to design the PGT concentration parameter distribution. Each value was assigned a weight using the protocol described in Section 2.6.2. The type of error was assigned as 0 if it was additive and 1 if it was multiplicative.

| Value (ppm) | Error (ppm) | Experimental details | | | | | | Weight | Type of error | Reference |
|---|---|---|---|---|---|---|---|---|---|---|
| | | Species | Expression Vector | Enzyme | pH | Temperature (°C) | Other | | | |
| 2.82 | NaN | Human | Gut | PGT | 7.5 | 37 | | 2048 | 0 | (Kim et al., 2014) |
| 1.74 $x10^1$ | NaN | Human | Oesophagus | PGT | 7.5 | 37 | | 2048 | 0 | (Wilhelm et al., 2014) |
| 1.25 $x10^2$ | NaN | Human | Lung | PGT | 7.5 | 37 | | 2048 | 0 | (Kim et al., 2014) |

**Table ST.10.24.1.3.2.** The log-normal distribution properties of the PGT concentration distribution. These values were calculated using the functions described in Section 2.6.2.

| Mode (ppm) | Mode (mM) | Confidence Interval | Location parameter (μ) | Scale parameter (σ) |
|---|---|---|---|---|
| 1.73 $x10^1$ | 9.57 $x10^{-5}$ | 4.72 | 3.90 | 1.02 |

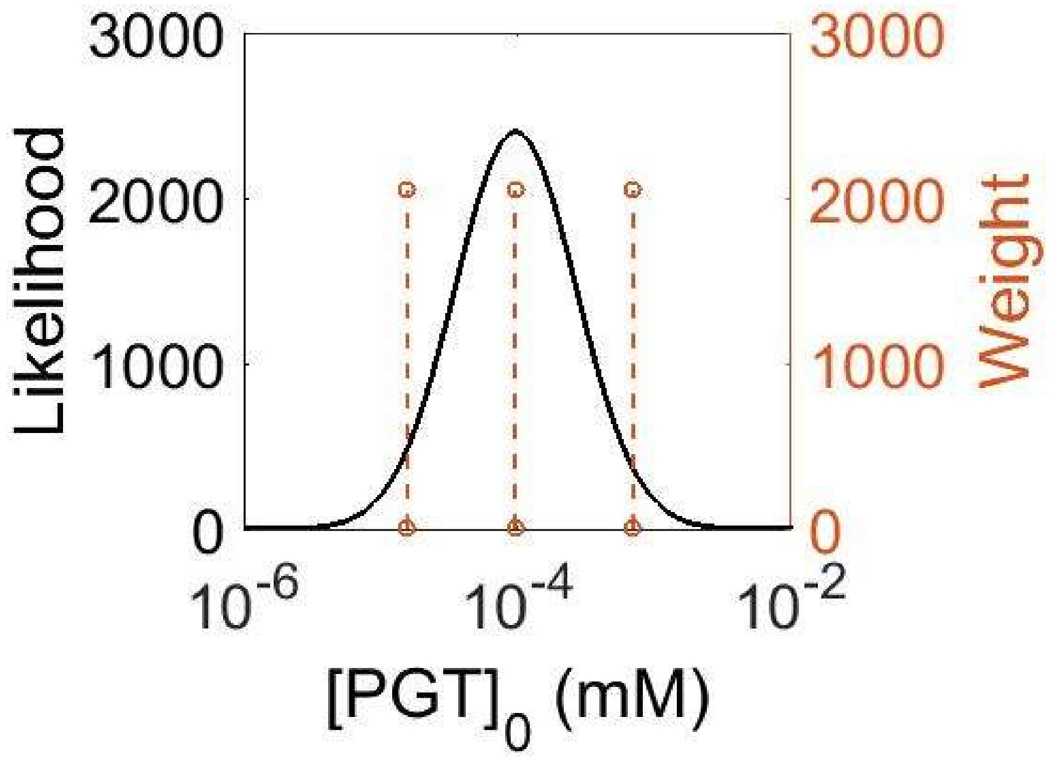


**Figure SF.10.24.1.3.1.** The estimated probability distribution for the PGT concentration. The value and weight of the literature values used to define the distribution are indicated by an orange dashed line. The x axis is plotted on a log-scale.

### S.10.25. Reaction 102: $PGE_2 \rightleftharpoons exPGE_2$ (PGT)

$PGH_2$ is relocated from the intracellular compartment to the extracellular compartment via the prostaglandin (PGT) transporter.

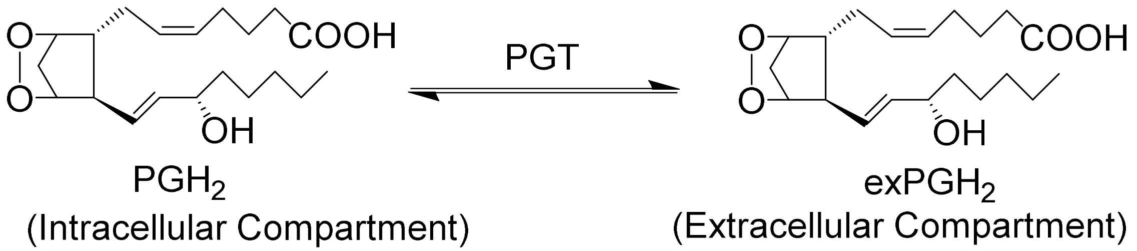


**Figure SF.10.25.** The transport of prostaglandin $H_2$ ($PGH_2$) from the intracellular compartment to the extracellular compartment by prostaglandin (PGT) transporter (Reaction 102).

**SEq.10.25.** Reaction rate law for Reaction 102.

$$\boldsymbol{v_{102}} = [\boldsymbol{PGT}] \cdot \boldsymbol{k_{cat}} \cdot \frac{{}^{\mathbf{PGE_2}}\!/_{K_m} \cdot \left(1 - \frac{ex\mathbf{PGE_2}}{\mathbf{PGE_2} \cdot e^{-0.5 \cdot \left(G + R \cdot T \cdot \ln\left(\frac{ATP}{ADP}\right)\right) / R \cdot T}}\right)}{1 + \frac{\mathbf{PGE_2}}{K_m} + \frac{ex\mathbf{PGE_2}}{K_m} + \mathbf{PGT\_CI}}$$

## S.10.25.1. Reaction parameters

### S.10.25.1.1. Parameter: PGT $k_{cat}$

Parameter values for the PGT $k_{cat}$ of Reaction 102 were obtained from the literature and summarised in Table ST.10.25.1.1.1. The log-normal distribution properties for the PGT $k_{cat}$ of Reaction 102 are shown in Table ST.10.25.1.1.2 and plotted in Figure SF.10.25.1.1.1.

**Table ST.10.25.1.1.1.** Literature information used to design the PGT $k_{cat}$ parameter distribution. Each value was assigned a weight using the protocol described in Section 2.6.2. The type of error was assigned as 0 if it was additive and 1 if it was multiplicative.

| Value ($min^{-1}$) | Error ($min^{-1}$) | Experimental details | | | | | | Weight | Type of error | Reference |
|---|---|---|---|---|---|---|---|---|---|---|
| | | Species | Expression Vector | Enzyme | pH | Temperature (°C) | Other | | | |
| 3.50 | NaN | Bacteria | E. coli. | Glucose transporter | Unknown | Unknown | Bionumbers calculation | **8** | 0 | (Waygood and Steeves, 1980) |
| 3.30 x$10^{-1}$ | NaN | Bacteria | E. coli. | Lactose transporter | Unknown | Unknown | Bionumbers calculation | **8** | 0 | (Naftalin et al., 2007) |
| 8.30 x$10^{-1}$ | NaN | Bacteria | E. coli. | Lactose transporter | Unknown | Unknown | Bionumbers calculation | **8** | 0 | (Naftalin et al., 2007) |
| 3.28 | NaN | Yeast | Saccharomyces cerevisiae | HXT7 transporter | Unknown | Unknown | Bionumbers calculation | **8** | 0 | (Ye et al., 2001) |

**Table ST.10.25.1.1.2.** The log-normal distribution properties of the PGT $k_{cat}$ distribution. These values were calculated using the functions described in Section 2.6.2.

| **Mode ($min^{-1}$)** | **Confidence Interval** | **Location parameter (μ)** | **Scale parameter (σ)** |
|---|---|---|---|
| 1.33 | 7.30 | 9.20 $x10^{-1}$ | 8.0 $10^{-1}$ |

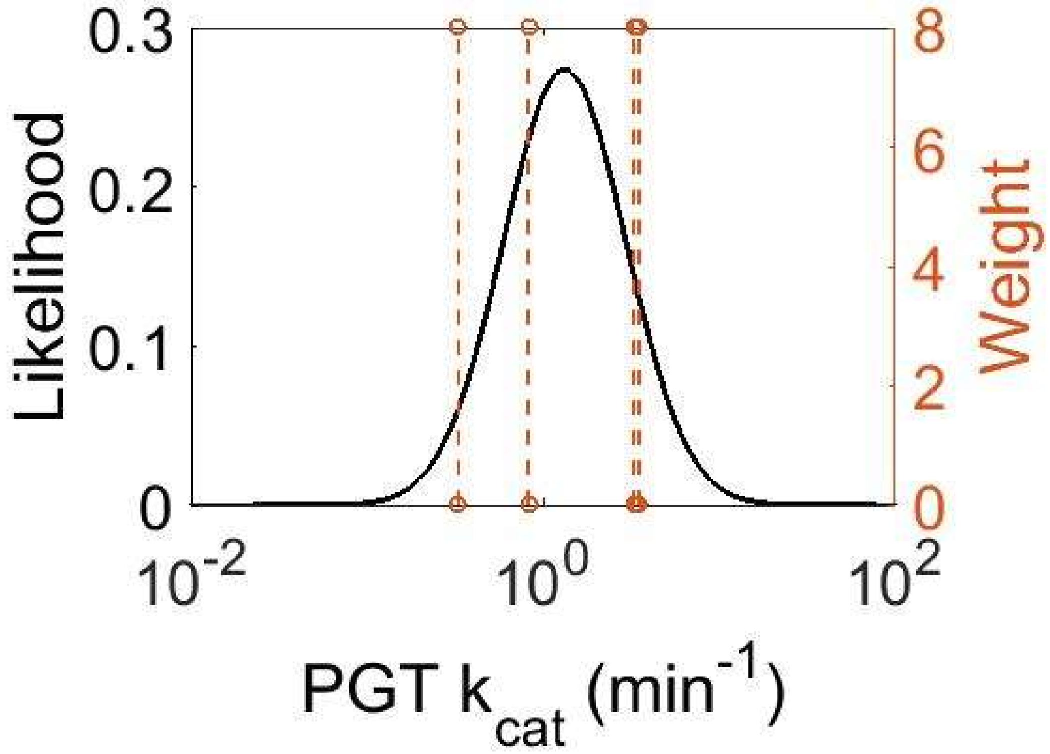


**Figure SF.10.25.1.1.1.** The estimated probability distribution for the PGT $k_{cat}$. The value and weight of the literature values used to define the distribution are indicated by an orange dashed line. The x axis is plotted on a log-scale.

### S.10.25.1.2. Parameter: PGT $K_{ms}$

Parameter values for the PGT $K_{ms}$ of Reaction 102 were obtained from the literature and summarised in Table ST.10.25.1.2.1. The log-normal distribution properties for the PGT $K_{ms}$ of Reaction 102 are shown in Table ST.10.25.1.2.2 and plotted in Figure SF.10.25.1.2.1.

**Table ST.10.25.1.2.1.** Literature information used to design the PGT $K_{ms}$ parameter distribution. Each value was assigned a weight using the protocol described in Section 2.6.2. The type of error was assigned as 0 if it was additive and 1 if it was multiplicative.

| Value (mM) | Error (mM) | Experimental details | | | | | | | Weight | Type of error | Reference |
|---|---|---|---|---|---|---|---|---|---|---|---|
| | | Substrate | Species | Expression Vector | Enzyme | pH | Temperature (°C) | Other | | | |
| 3.31 x10$^{-5}$ | 1.31 x10$^{-4}$ | | Human | Eye | PGT | 7.5 | 37 | | 512 | 0 | (Waygood and Steeves, 1980) |
| 3.76 x10$^{-4}$ | 3.40 x10$^{-5}$ | | Human | Embryonic kidney cells (HEK293) | PGT | Unknown | 37 | | 192 | 0 | (Naftalin et al., 2007) |
| 5.40 x10$^{-3}$ | NaN | | Human | Embryonic kidney cells (HEK293) | PGT | Unknown | 37 | | 2048 | 0 | (Naftalin et al., 2007) |
| 7.20 x10$^{-3}$ | 5.95 x10$^{-4}$ | | Canine | Madin-Darby Canine Kidney Epithelial Cells (MDCK) cells | PGT | Unknown | Unknown | | 512 | 0 | (Ye et al., 2001) |

| | | | | | | | | | | | |
|---|---|---|---|---|---|---|---|---|---|---|---|
| 9.40 x10$^{-5}$ | NaN | PGE2 | Human | HeLa cells | PGT | 7 | 27 | | **1024** | 0 | (Waygood and Steeves, 1980) |
| 4.23 x10$^{-4}$ | NaN | TXB2 | Human | HeLa cells | PGT | 7 | 27 | | **1024** | 0 | (Naftalin et al., 2007) |
| 7.57 x10$^{-3}$ | NaN | 6-keto-PGF1a | Human | HeLa cells | PGT | 7 | 27 | | **1024** | 0 | (Naftalin et al., 2007) |
| 4.87 x10$^{-5}$ | NaN | PGE2 | Human | HeLa cells | PGT | 7 | 27 | | **1024** | 0 | (Ye et al., 2001) |
| 5.02 x10$^{-5}$ | NaN | PGF2a | Human | HeLa cells | PGT | 7 | 27 | | **1024** | 0 | (Waygood and Steeves, 1980) |
| 5.00 x10$^{-3}$ | NaN | 15-K-PGE2 | Human | HeLa cells | PGT | 7 | 27 | | **1024** | 0 | (Naftalin et al., 2007) |
| 5.00 x10$^{-3}$ | NaN | DH-15-K-PGE2 | Human | HeLa cells | PGT | 7 | 27 | | **1024** | 0 | (Naftalin et al., 2007) |
| 3.82 x10$^{-5}$ | NaN | PGH2 | Human | HeLa cells | PGT | 7 | 27 | | **2048** | 0 | (Ye et al., 2001) |

**Table ST.10.25.1.2.2.** The log-normal distribution properties of the PGT $K_{ms}$ distribution. These values were calculated using the functions described in Section 2.6.2.

| **Mode (mM)** | **Confidence Interval** | **Location parameter (μ)** | **Scale parameter (σ)** |
|---|---|---|---|
| 1.10 x10$^{-3}$ | 9.42 x10$^{1}$ | -4.67 | 1.45 |

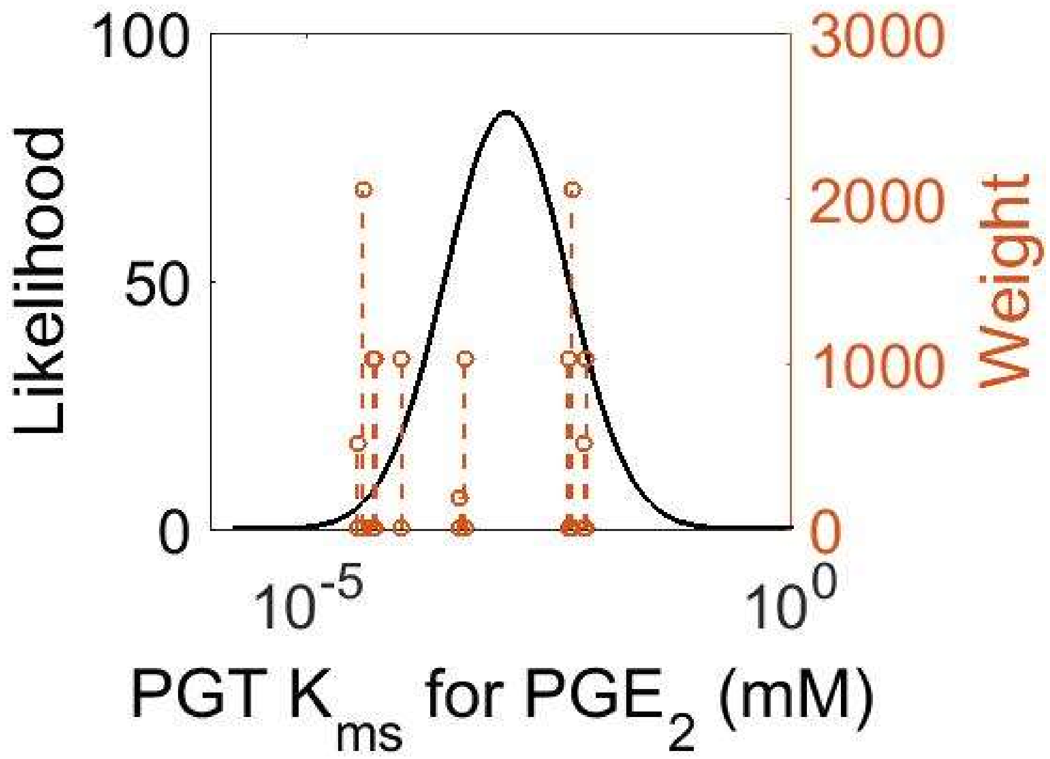


**Figure SF.10.25.1.2.1.** The estimated probability distribution for the PGT $K_{ms}$. The value and weight of the literature values used to define the distribution are indicated by an orange dashed line. The x axis is plotted on a log-scale.

### S.10.25.1.3. Parameter: PGT concentration

Parameter values for the PGT concentration of Reaction 102 were obtained from the literature and summarised in Table ST.10.25.1.3.1. The log-normal distribution properties for the PGT concentration of Reaction 102 are shown in Table ST.10.25.1.3.2 and plotted in Figure SF.10.25.1.3.1. To convert the enzyme concentration from ppm to mM, **Equation S.6.2** was used.

**Table ST.10.25.1.3.1.** Literature information used to design the PGT concentration parameter distribution. Each value was assigned a weight using the protocol described in Section 2.6.2. The type of error was assigned as 0 if it was additive and 1 if it was multiplicative.

| Value (ppm) | Error (ppm) | Experimental details | | | | | | Weight | Type of error | Reference |
|---|---|---|---|---|---|---|---|---|---|---|
| | | Species | Expression Vector | Enzyme | pH | Temperature (°C) | Other | | | |
| 2.82 | NaN | Human | Gut | PGT | 7.5 | 37 | | 2048 | 0 | (Kim et al., 2014) |
| $1.74 \times 10^{1}$ | NaN | Human | Oesophagus | PGT | 7.5 | 37 | | 2048 | 0 | (Wilhelm et al., 2014) |
| $1.25 \times 10^{2}$ | NaN | Human | Lung | PGT | 7.5 | 37 | | 2048 | 0 | (Kim et al., 2014) |

**Table ST.10.25.1.3.2.** The log-normal distribution properties of the PGT concentration distribution. These values were calculated using the functions described in Section 2.6.2.

| Mode (ppm) | Mode (mM) | Confidence Interval | Location parameter (μ) | Scale parameter (σ) |
|---|---|---|---|---|
| $1.73 \times 10^{1}$ | $9.57 \times 10^{-5}$ | 4.72 | 3.90 | 1.02 |

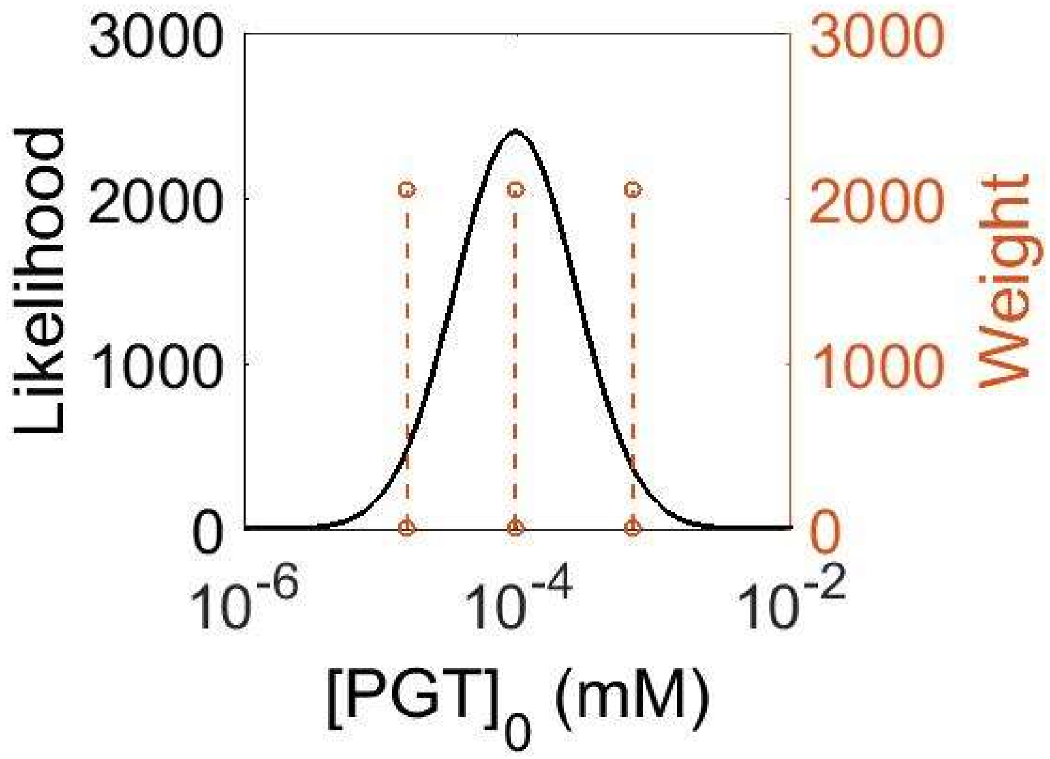


**Figure SF.10.25.1.3.1.** The estimated probability distribution for the PGT concentration. The value and weight of the literature values used to define the distribution are indicated by an orange dashed line. The x axis is plotted on a log-scale.

### S.10.26. Reaction 103: $PGI_2 \rightleftharpoons exPGI_2$ (PGT)

$PGI_2$ is relocated from the intracellular compartment to the extracellular compartment via the prostaglandin (PGT) transporter.

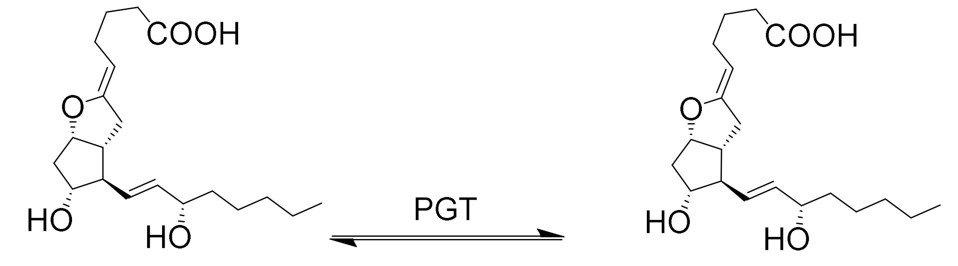


$PGI_2$ (Intracellular Compartment) | $exPGI_2$ (Extracellular Compartment)

**Figure SF.10.26.** The transport of prostaglandin $I_2$ ($PGI_2$) from the intracellular compartment to the extracellular compartment by prostaglandin (PGT) transporter (Reaction 103).

**SEq.10.26.** Reaction rate law for Reaction 103.

$$v_{103} = [PGT] \cdot k_{cat} \cdot \frac{\mathrm{PGI_2}/K_m \cdot \left(1 - \frac{ex\mathrm{PGI_2}}{\mathrm{PGI_2} \cdot e^{-0.5 \cdot \left(G + R \cdot T \cdot \ln\left(\frac{ATP}{ADP}\right)\right)/R \cdot T}}\right)}{1 + \frac{\mathrm{PGI_2}}{K_m} + \frac{ex\mathrm{PGI_2}}{K_m} + \mathrm{PGT\_CI}}$$

## S.10.26.1. Reaction parameters

### S.10.26.1.1. Parameter: PGT $k_{cat}$

Parameter values for the PGT $k_{cat}$ of Reaction 103 were obtained from the literature and summarised in Table ST.10.26.1.1.1. The log-normal distribution properties for the PGT $k_{cat}$ of Reaction 103 are shown in Table ST.10.26.1.1.2 and plotted in Figure SF.10.26.1.1.1.

**Table ST.10.26.1.1.1.** Literature information used to design the PGT $k_{cat}$ parameter distribution. Each value was assigned a weight using the protocol described in Section 2.6.2. The type of error was assigned as 0 if it was additive and 1 if it was multiplicative.

| Value ($min^{-1}$) | Error ($min^{-1}$) | Experimental details | | | | | | Weight | Type of error | Reference |
|---|---|---|---|---|---|---|---|---|---|---|
| | | Species | Expression Vector | Enzyme | pH | Temperature (°C) | Other | | | |
| 3.50 | NaN | Bacteria | E. coli. | Glucose transporter | Unknown | Unknown | Bionumbers calculation | **8** | 0 | (Waygood and Steeves, 1980) |
| 3.30 x$10^{-1}$ | NaN | Bacteria | E. coli. | Lactose transporter | Unknown | Unknown | Bionumbers calculation | **8** | 0 | (Naftalin et al., 2007) |
| 8.30 x$10^{-1}$ | NaN | Bacteria | E. coli. | Lactose transporter | Unknown | Unknown | Bionumbers calculation | **8** | 0 | (Naftalin et al., 2007) |
| 3.28 | NaN | Yeast | Saccharomyces cerevisiae | HXT7 transporter | Unknown | Unknown | Bionumbers calculation | **8** | 0 | (Ye et al., 2001) |

**Table ST.10.26.1.1.2.** The log-normal distribution properties of the PGT $k_{cat}$ distribution. These values were calculated using the functions described in Section 2.6.2.

| **Mode ($min^{-1}$)** | **Confidence Interval** | **Location parameter (μ)** | **Scale parameter (σ)** |
|---|---|---|---|
| 1.33 | 7.30 | $9.20 \times 10^{-1}$ | $8.0 \times 10^{-1}$ |

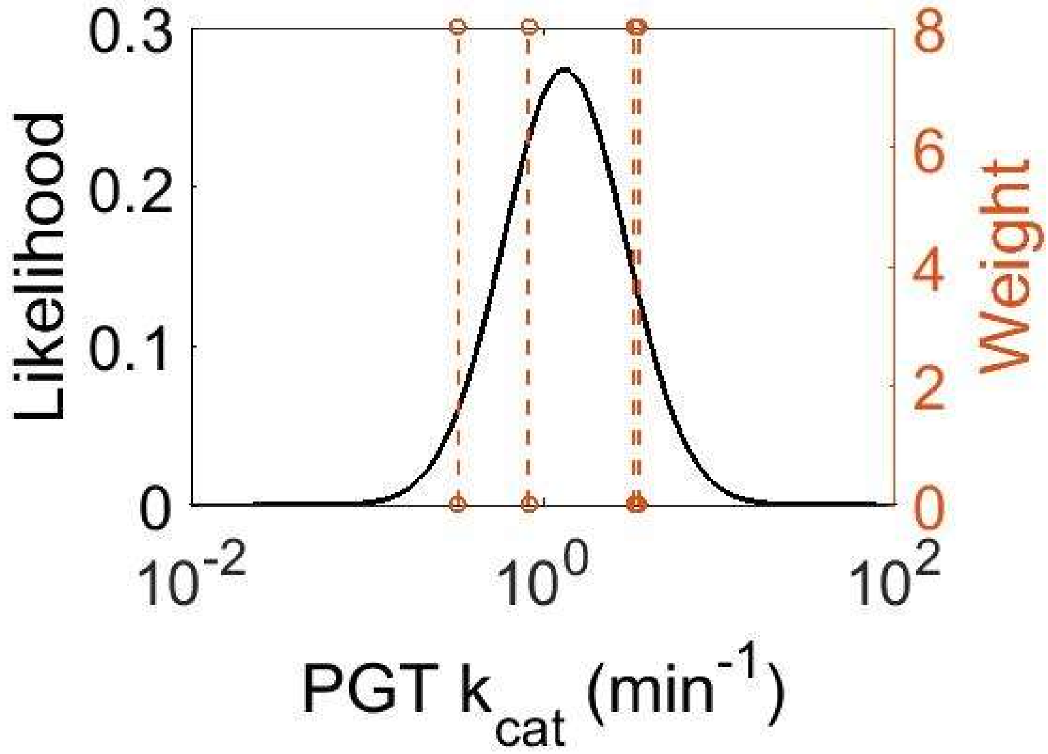


**Figure SF.10.26.1.1.1.** The estimated probability distribution for the PGT $k_{cat}$. The value and weight of the literature values used to define the distribution are indicated by an orange dashed line. The x axis is plotted on a log-scale.

### S.10.26.1.2. Parameter: PGT $K_{ms}$

Parameter values for the PGT $K_{ms}$ of Reaction 103 were obtained from the literature and summarised in Table ST.10.26.1.2.1. The log-normal distribution properties for the PGT $K_{ms}$ of Reaction 103 are shown in Table ST.10.26.1.2.2 and plotted in Figure SF.10.26.1.2.1.

**Table ST.10.26.1.2.1.** Literature information used to design the PGT $K_{ms}$ parameter distribution. Each value was assigned a weight using the protocol described in Section 2.6.2. The type of error was assigned as 0 if it was additive and 1 if it was multiplicative.

| Value (mM) | Error (mM) | Experimental details | | | | | | | Weight | Type of error | Reference |
|---|---|---|---|---|---|---|---|---|---|---|---|
| | | Substrate | Species | Expression Vector | Enzyme | pH | Temperature (°C) | Other | | | |
| 3.31 x10$^{-5}$ | 1.31 x10$^{-4}$ | | Human | Eye | PGT | 7.5 | 37 | | 512 | 0 | (Waygood and Steeves, 1980) |
| 3.76 x10$^{-4}$ | 3.40 x10$^{-5}$ | | Human | Embryonic kidney cells (HEK293) | PGT | Unknown | 37 | | 192 | 0 | (Naftalin et al., 2007) |
| 5.40 x10$^{-3}$ | NaN | | Human | Embryonic kidney cells (HEK293) | PGT | Unknown | 37 | | 2048 | 0 | (Naftalin et al., 2007) |
| 7.20 x10$^{-3}$ | 5.95 x10$^{-4}$ | | Canine | Madin-Darby Canine Kidney Epithelial Cells (MDCK) cells | PGT | Unknown | Unknown | | 512 | 0 | (Ye et al., 2001) |

| | | | | | | | | | | | |
|---|---|---|---|---|---|---|---|---|---|---|---|
| $9.40 \times 10^{-5}$ | NaN | PGE2 | Human | HeLa cells | PGT | 7 | 27 | | **1024** | 0 | (Waygood and Steeves, 1980) |
| $4.23 \times 10^{-4}$ | NaN | TXB2 | Human | HeLa cells | PGT | 7 | 27 | | **1024** | 0 | (Naftalin et al., 2007) |
| $7.57 \times 10^{-3}$ | NaN | K6PGF1a | Human | HeLa cells | PGT | 7 | 27 | | **1024** | 0 | (Naftalin et al., 2007) |
| $4.87 \times 10^{-5}$ | NaN | PGE2 | Human | HeLa cells | PGT | 7 | 27 | | **1024** | 0 | (Ye et al., 2001) |
| $5.02 \times 10^{-5}$ | NaN | PGF2a | Human | HeLa cells | PGT | 7 | 27 | | **1024** | 0 | (Waygood and Steeves, 1980) |
| $5.00 \times 10^{-3}$ | NaN | 15-K-PGE2 | Human | HeLa cells | PGT | 7 | 27 | | **1024** | 0 | (Naftalin et al., 2007) |
| $5.00 \times 10^{-3}$ | NaN | DH-15-K-PGE2 | Human | HeLa cells | PGT | 7 | 27 | | **1024** | 0 | (Naftalin et al., 2007) |
| $3.82 \times 10^{-5}$ | NaN | PGH2 | Human | HeLa cells | PGT | 7 | 27 | | **2048** | 0 | (Ye et al., 2001) |

**Table ST.10.26.1.2.2.** The log-normal distribution properties of the PGT $K_{ms}$ distribution. These values were calculated using the functions described in Section 2.6.2.

| **Mode (mM)** | **Confidence Interval** | **Location parameter (μ)** | **Scale parameter (σ)** |
|---|---|---|---|
| $1.10 \times 10^{-3}$ | $9.42 \times 10^{1}$ | -4.67 | 1.45 |

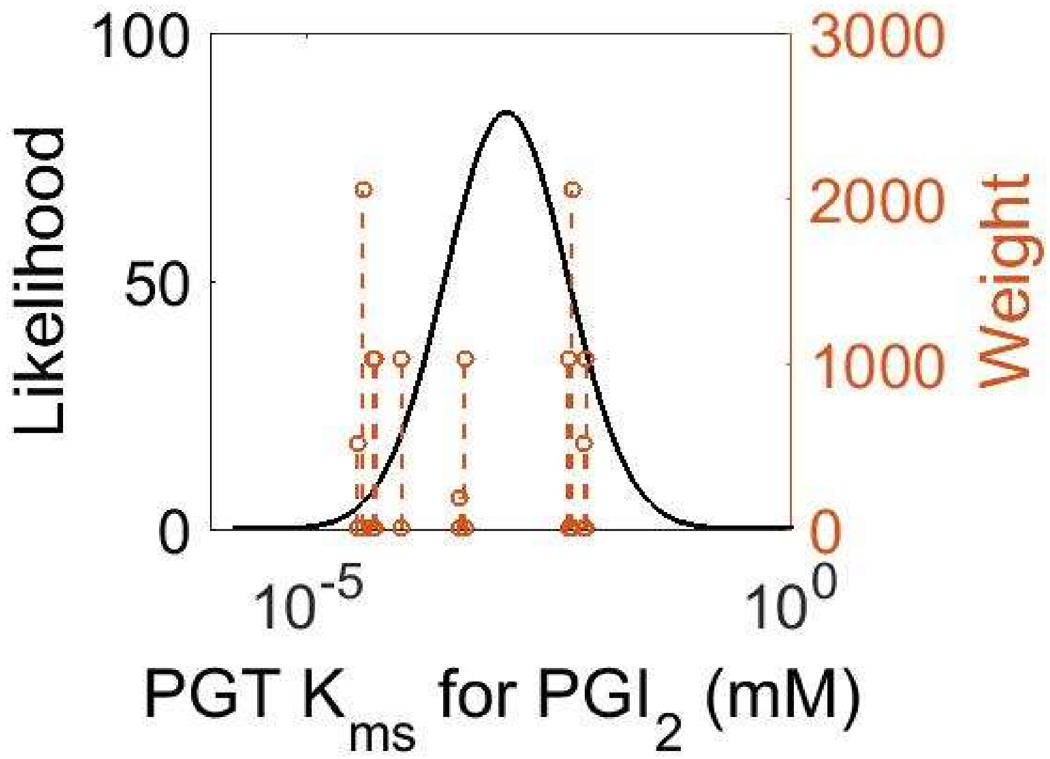


**Figure SF.10.26.1.2.1.** The estimated probability distribution for the PGT $K_{ms}$. The value and weight of the literature values used to define the distribution are indicated by an orange dashed line. The x axis is plotted on a log-scale.

**S.10.26.1.3. Parameter: PGT concentration**

Parameter values for the PGT concentration of Reaction 103 were obtained from the literature and summarised in Table ST.10.26.1.3.1. The log-normal distribution properties for the PGT concentration of Reaction 103 are shown in Table ST.10.26.1.3.2 and plotted in Figure SF.10.26.1.3.1. To convert the enzyme concentration from ppm to mM, **Equation S.6.2** was used.

**Table ST.10.26.1.3.1.** Literature information used to design the PGT concentration parameter distribution. Each value was assigned a weight using the protocol described in Section 2.6.2. The type of error was assigned as 0 if it was additive and 1 if it was multiplicative.

| Value (ppm) | Error (ppm) | Experimental details | | | | | | Weight | Type of error | Reference |
|---|---|---|---|---|---|---|---|---|---|---|
| | | Species | Expression Vector | Enzyme | pH | Temperature (°C) | Other | | | |
| 2.82 | NaN | Human | Gut | PGT | 7.5 | 37 | | 2048 | 0 | (Kim et al., 2014) |
| $1.74 \times 10^{1}$ | NaN | Human | Oesophagus | PGT | 7.5 | 37 | | 2048 | 0 | (Wilhelm et al., 2014) |
| $1.25 \times 10^{2}$ | NaN | Human | Lung | PGT | 7.5 | 37 | | 2048 | 0 | (Kim et al., 2014) |

**Table ST.10.26.1.3.2** The log-normal distribution properties of the PGT concentration distribution. These values were calculated using the functions described in Section 2.6.2.

| Mode (ppm) | Mode (mM) | Confidence Interval | Location parameter (μ) | Scale parameter (σ) |
|---|---|---|---|---|
| $1.73 \times 10^{1}$ | $9.57 \times 10^{-5}$ | 4.72 | 3.90 | 1.02 |

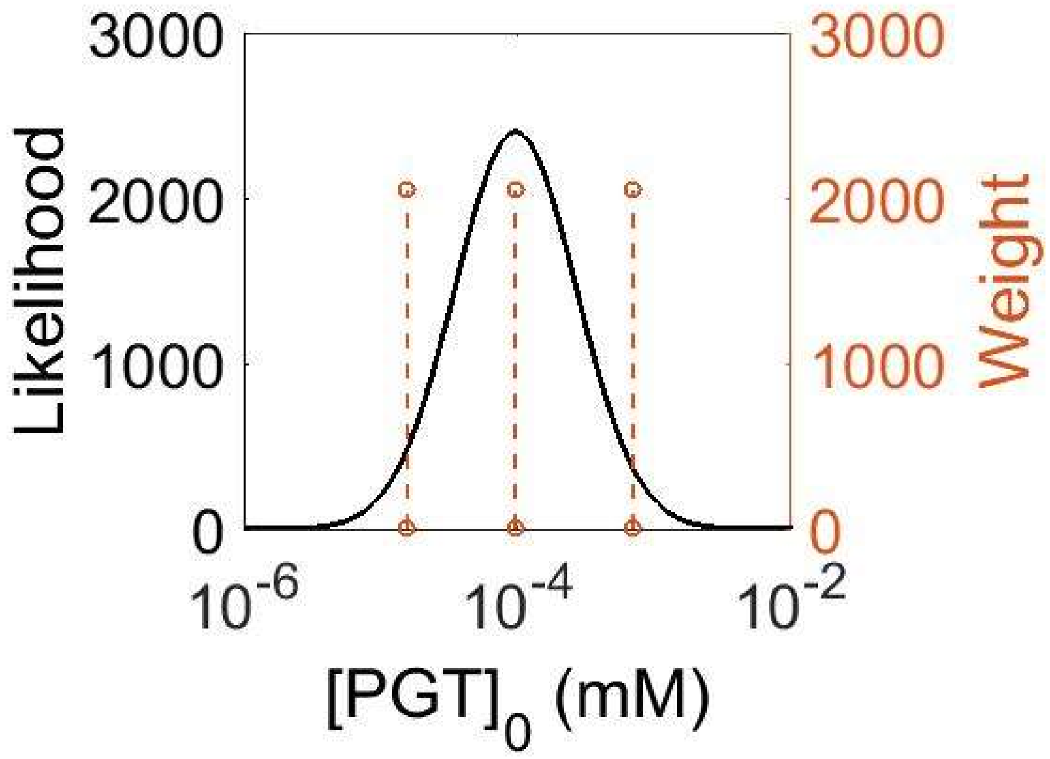


**Figure SF.10.26.1.3.1.** The estimated probability distribution for the PGT concentration. The value and weight of the literature values used to define the distribution are indicated by an orange dashed line. The x axis is plotted on a log-scale.

**S.10.27. Reaction 104: $PGD_2 \rightleftharpoons exPGD_2$ (PGT)**

$PGD_2$ is relocated from the intracellular compartment to the extracellular compartment via the prostaglandin (PGT) transporter.

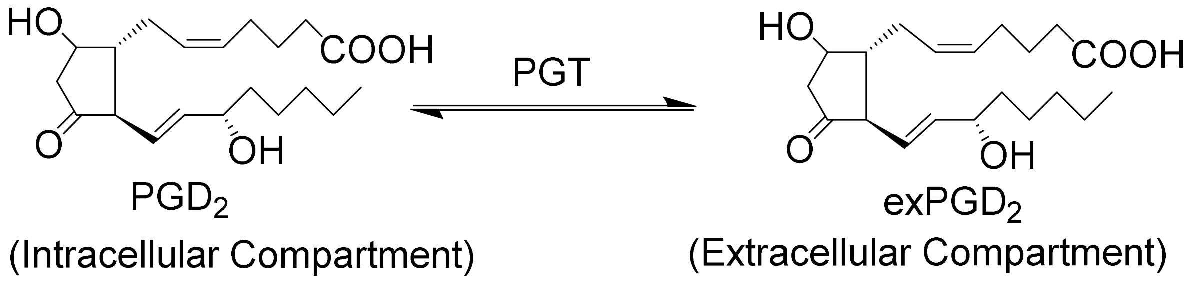


**Figure SF.10.27.** The transport of prostaglandin $D_2$ ($PGD_2$) from the intracellular compartment to the extracellular compartment by prostaglandin (PGT) transporter (Reaction 104).

**SEq.10.27.** Reaction rate law for Reaction 104.

$$v_{104} = [PGT] \cdot k_{cat} \cdot \frac{\mathrm{PGD_2}/K_m \cdot \left( 1 - \frac{ex\mathrm{PGD_2}}{\mathrm{PGD_2} \cdot e^{-0.5 \cdot \left(G + R \cdot T \cdot \ln\left(\frac{ATP}{ADP}\right)\right) / R \cdot T}} \right)}{1 + \frac{\mathrm{PGD_2}}{K_m} + \frac{ex\mathrm{PGD_2}}{K_m} + \mathrm{PGT\_CI}}$$

## S.10.27.1. Reaction parameters

### S.10.27.1.1. Parameter: PGT $k_{cat}$

Parameter values for the PGT $k_{cat}$ of Reaction 104 were obtained from the literature and summarised in Table ST.10.27.1.1.1. The log-normal distribution properties for the PGT $k_{cat}$ of Reaction 104 are shown in Table ST.10.27.1.1.2 and plotted in Figure SF.10.27.1.1.1.

**Table ST.10.27.1.1.1.** Literature information used to design the PGT $k_{cat}$ parameter distribution. Each value was assigned a weight using the protocol described in Section 2.6.2. The type of error was assigned as 0 if it was additive and 1 if it was multiplicative.

| Value ($min^{-1}$) | Error ($min^{-1}$) | Experimental details | | | | | | Weight | Type of error | Reference |
|---|---|---|---|---|---|---|---|---|---|---|
| | | Species | Expression Vector | Enzyme | pH | Temperature (°C) | Other | | | |
| 3.50 | NaN | Bacteria | E. coli. | Glucose transporter | Unknown | Unknown | Bionumbers calculation | **8** | 0 | (Waygood and Steeves, 1980) |
| 3.30 x$10^{-1}$ | NaN | Bacteria | E. coli. | Lactose transporter | Unknown | Unknown | Bionumbers calculation | **8** | 0 | (Naftalin et al., 2007) |
| 8.30 x$10^{-1}$ | NaN | Bacteria | E. coli. | Lactose transporter | Unknown | Unknown | Bionumbers calculation | **8** | 0 | (Naftalin et al., 2007) |
| 3.28 | NaN | Yeast | Saccharomyces cerevisiae | HXT7 transporter | Unknown | Unknown | Bionumbers calculation | **8** | 0 | (Ye et al., 2001) |

**Table ST.10.27.1.1.2.** The log-normal distribution properties of the PGT $k_{cat}$ distribution. These values were calculated using the functions described in Section 2.6.2.

| Mode ($min^{-1}$) | Confidence Interval | Location parameter (μ) | Scale parameter (σ) |
|---|---|---|---|
| 1.33 | 7.30 | 9.20 $x10^{-1}$ | 8.0 $x10^{-1}$ |

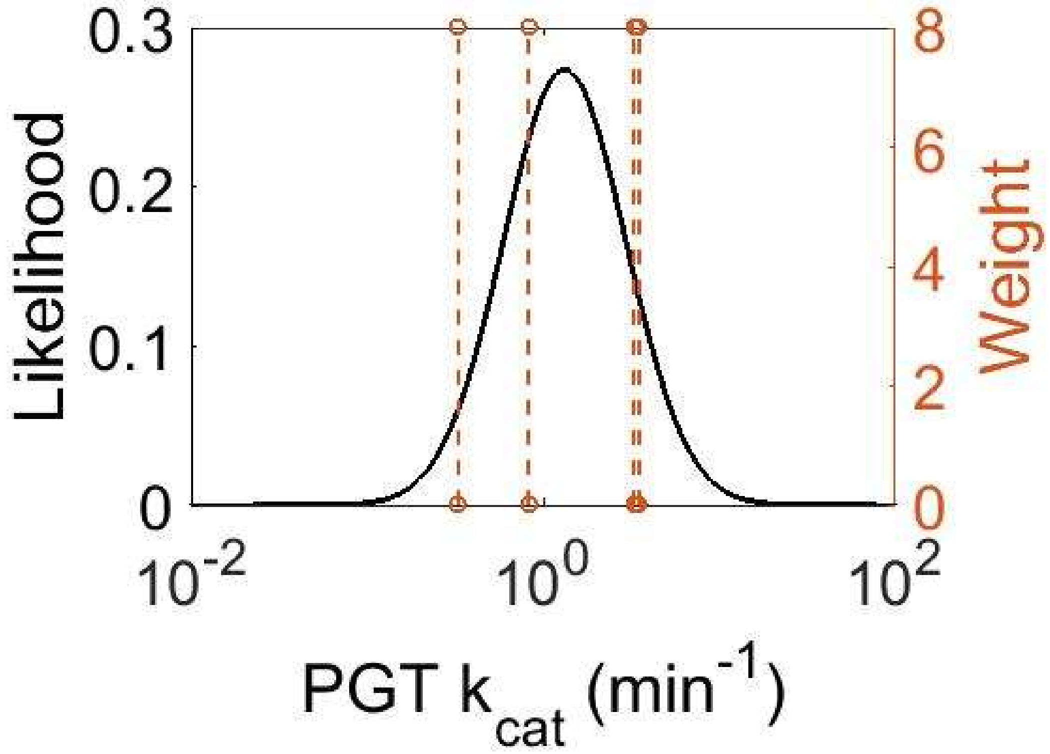


**Figure SF.10.27.1.1.1.** The estimated probability distribution for the PGT $k_{cat}$. The value and weight of the literature values used to define the distribution are indicated by an orange dashed line. The x axis is plotted on a log-scale.

### S.10.27.1.2. Parameter: PGT $K_{ms}$

Parameter values for the PGT $K_{ms}$ of Reaction 104 were obtained from the literature and summarised in Table ST.10.27.1.2.1. The log-normal distribution properties for the PGT $K_{ms}$ of Reaction 104 are shown in Table ST.10.27.1.2.2 and plotted in Figure SF.10.27.1.2.1.

**Table ST.10.27.1.2.1.** Literature information used to design the PGT $K_{ms}$ parameter distribution. Each value was assigned a weight using the protocol described in Section 2.6.2. The type of error was assigned as 0 if it was additive and 1 if it was multiplicative.

| Value (mM) | Error (mM) | Experimental details | | | | | | Other | Weight | Type of error | Reference |
|---|---|---|---|---|---|---|---|---|---|---|---|
| | | Substrate | Species | Expression Vector | Enzyme | pH | Temperature (°C) | | | | |
| $3.31 \times 10^{-5}$ | $1.31 \times 10^{-4}$ | | Human | Eye | PGT | 7.5 | 37 | | 512 | 0 | (Waygood and Steeves, 1980) |
| $3.76 \times 10^{-4}$ | $3.40 \times 10^{-5}$ | | Human | Embryonic kidney cells (HEK293) | PGT | Unknown | 37 | | 192 | 0 | (Naftalin et al., 2007) |
| $5.40 \times 10^{-3}$ | NaN | | Human | Embryonic kidney cells (HEK293) | PGT | Unknown | 37 | | 2048 | 0 | (Naftalin et al., 2007) |
| $7.20 \times 10^{-3}$ | $5.95 \times 10^{-4}$ | | Canine | Madin-Darby Canine Kidney Epithelial Cells (MDCK) cells | PGT | Unknown | Unknown | | 512 | 0 | (Ye et al., 2001) |

| | | | | | | | | | | | |
|---|---|---|---|---|---|---|---|---|---|---|---|
| 9.40 x10$^{-5}$ | NaN | PGE2 | Human | HeLa cells | PGT | 7 | 27 | | **1024** | 0 | (Waygood and Steeves, 1980) |
| 4.23 x10$^{-4}$ | NaN | TXB2 | Human | HeLa cells | PGT | 7 | 27 | | **1024** | 0 | (Naftalin et al., 2007) |
| 7.57 x10$^{-3}$ | NaN | K6PGF1a | Human | HeLa cells | PGT | 7 | 27 | | **1024** | 0 | (Naftalin et al., 2007) |
| 4.87 x10$^{-5}$ | NaN | PGE2 | Human | HeLa cells | PGT | 7 | 27 | | **1024** | 0 | (Ye et al., 2001) |
| 5.02 x10$^{-5}$ | NaN | PGF2a | Human | HeLa cells | PGT | 7 | 27 | | **1024** | 0 | (Waygood and Steeves, 1980) |
| 5.00 x10$^{-3}$ | NaN | 15-K-PGE2 | Human | HeLa cells | PGT | 7 | 27 | | **1024** | 0 | (Naftalin et al., 2007) |
| 5.00 x10$^{-3}$ | NaN | DH-15-K-PGE2 | Human | HeLa cells | PGT | 7 | 27 | | **1024** | 0 | (Naftalin et al., 2007) |
| 3.82 x10$^{-5}$ | NaN | PGH2 | Human | HeLa cells | PGT | 7 | 27 | | **2048** | 0 | (Ye et al., 2001) |

**Table ST.10.27.1.2.2.** The log-normal distribution properties of the PGT $K_{ms}$ distribution. These values were calculated using the functions described in Section 2.6.2.

| **Mode (mM)** | **Confidence Interval** | **Location parameter (μ)** | **Scale parameter (σ)** |
|---|---|---|---|
| 1.10 x10$^{-3}$ | 9.42 x10$^{1}$ | -4.67 | 1.45 |

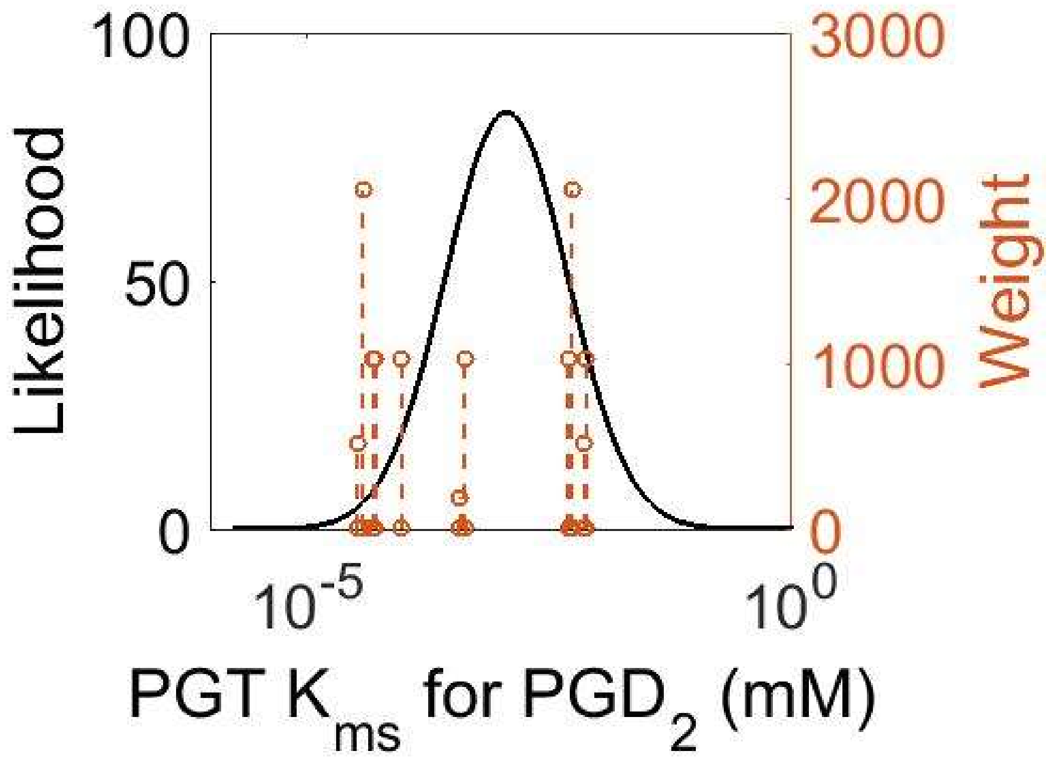


**Figure SF.10.27.1.2.1.** The estimated probability distribution for the PGT $K_{ms}$. The value and weight of the literature values used to define the distribution are indicated by an orange dashed line. The x axis is plotted on a log-scale.

### S.10.27.1.3. Parameter: PGT concentration

Parameter values for the PGT concentration of Reaction 104 were obtained from the literature and summarised in Table ST.10.27.1.3.1. The log-normal distribution properties for the PGT concentration of Reaction 104 are shown in Table ST.10.27.1.3.2 and plotted in Figure SF.10.27.1.3.1. To convert the enzyme concentration from ppm to mM, **Equation S.6.2** was used.

**Table ST.10.27.1.3.1.** Literature information used to design the PGT concentration parameter distribution. Each value was assigned a weight using the protocol described in Section 2.6.2. The type of error was assigned as 0 if it was additive and 1 if it was multiplicative.

| Value (ppm) | Error (ppm) | Experimental details | | | | | | Weight | Type of error | Reference |
|---|---|---|---|---|---|---|---|---|---|---|
| | | Species | Expression Vector | Enzyme | pH | Temperature (°C) | Other | | | |
| 2.82 | NaN | Human | Gut | PGT | 7.5 | 37 | | 2048 | 0 | (Kim et al., 2014) |
| 1.74 $x10^1$ | NaN | Human | Oesophagus | PGT | 7.5 | 37 | | 2048 | 0 | (Wilhelm et al., 2014) |
| 1.25 $x10^2$ | NaN | Human | Lung | PGT | 7.5 | 37 | | 2048 | 0 | (Kim et al., 2014) |

**Table ST.10.27.1.3.2.** The log-normal distribution properties of the PGT concentration distribution. These values were calculated using the functions described in Section 2.6.2.

| Mode (ppm) | Mode (mM) | Confidence Interval | Location parameter (μ) | Scale parameter (σ) |
|---|---|---|---|---|
| 1.73 $x10^1$ | 9.57 $x10^{-5}$ | 4.72 | 3.90 | 1.02 |

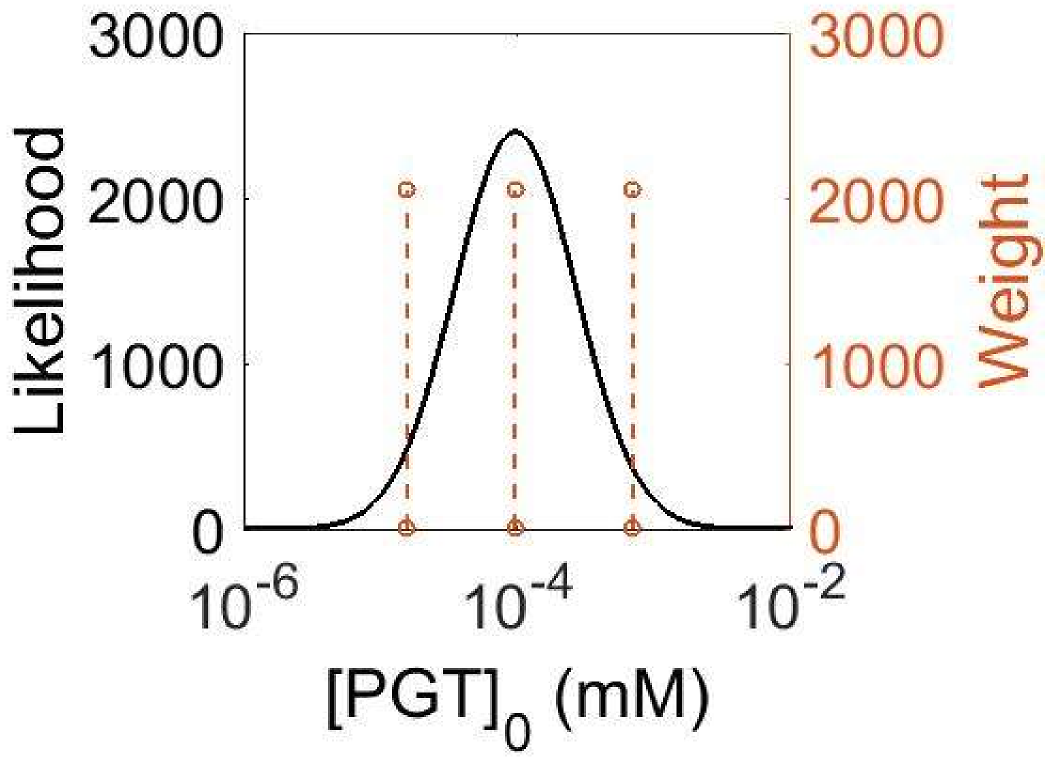


**Figure SF.10.27.1.3.1.** The estimated probability distribution for the PGT concentration. The value and weight of the literature values used to define the distribution are indicated by an orange dashed line. The x axis is plotted on a log-scale.

**S.10.28. Reaction 105: $PGJ_2 \rightleftharpoons exPGJ_2$ (PGT)**

$PGJ_2$ is relocated from the intracellular compartment to the extracellular compartment via the prostaglandin (PGT) transporter.

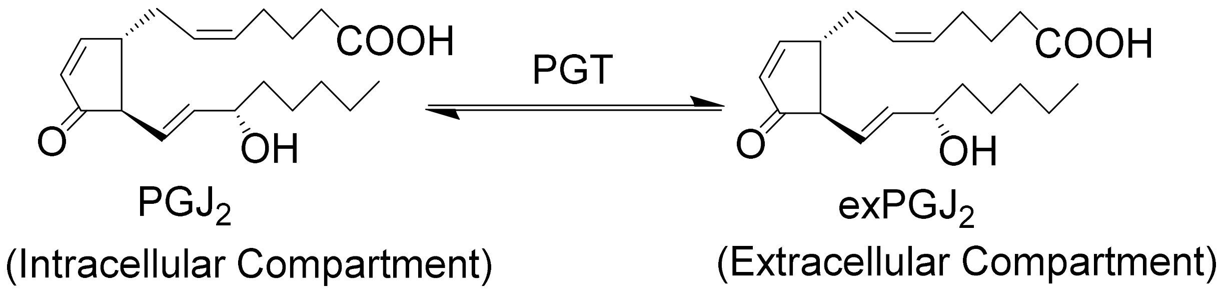


**Figure SF.10.28.** The transport of prostaglandin $J_2$ ($PGJ_2$) from the intracellular compartment to the extracellular compartment by prostaglandin (PGT) transporter (Reaction 105).

**SEq.10.28.** Reaction rate law for Reaction 105.

$$v_{105} = [PGT] \cdot k_{cat} \cdot \frac{\mathrm{PGJ2}/K_m \cdot \left(1 - \frac{ex\mathrm{PGJ2}}{\mathrm{PGJ2} \cdot e^{-0.5 \cdot \left(G + R \cdot T \cdot \ln\left(\frac{ATP}{ADP}\right)\right)/R \cdot T}}\right)}{1 + \frac{\mathrm{PGJ2}}{K_m} + \frac{ex\mathrm{PGJ2}}{K_m} + \mathrm{PGT\_CI}}$$

## S.10.28.1 Reaction parameters

### S.10.28.1.1. Parameter: PGT $k_{cat}$

Parameter values for the PGT $k_{cat}$ of Reaction 105 were obtained from the literature and summarised in Table ST.10.28.1.1.1. The log-normal distribution properties for the PGT $k_{cat}$ of Reaction 105 are shown in Table ST.10.28.1.1.2 and plotted in Figure SF.10.28.1.1.1.

**Table ST.10.28.1.1.1.** Literature information used to design the PGT $k_{cat}$ parameter distribution. Each value was assigned a weight using the protocol described in Section 2.6.2. The type of error was assigned as 0 if it was additive and 1 if it was multiplicative.

| Value ($min^{-1}$) | Error ($min^{-1}$) | Experimental details | | | | | | Weight | Type of error | Reference |
|---|---|---|---|---|---|---|---|---|---|---|
| | | Species | Expression Vector | Enzyme | pH | Temperature (°C) | Other | | | |
| 3.50 | NaN | Bacteria | E. coli. | Glucose transporter | Unknown | Unknown | Bionumbers calculation | **8** | 0 | (Waygood and Steeves, 1980) |
| 3.30 x$10^{-1}$ | NaN | Bacteria | E. coli. | Lactose transporter | Unknown | Unknown | Bionumbers calculation | **8** | 0 | (Naftalin et al., 2007) |
| 8.30 x$10^{-1}$ | NaN | Bacteria | E. coli. | Lactose transporter | Unknown | Unknown | Bionumbers calculation | **8** | 0 | (Naftalin et al., 2007) |
| 3.28 | NaN | Yeast | Saccharomyces cerevisiae | HXT7 transporter | Unknown | Unknown | Bionumbers calculation | **8** | 0 | (Ye et al., 2001) |

**Table ST.10.28.1.1.2.** The log-normal distribution properties of the PGT $k_{cat}$ distribution. These values were calculated using the functions described in Section 2.6.2.

| **Mode ($min^{-1}$)** | **Confidence Interval** | **Location parameter (μ)** | **Scale parameter (σ)** |
|---|---|---|---|
| 1.33 | 7.30 | 9.20 $x10^{-1}$ | 8.0 $10^{-1}$ |

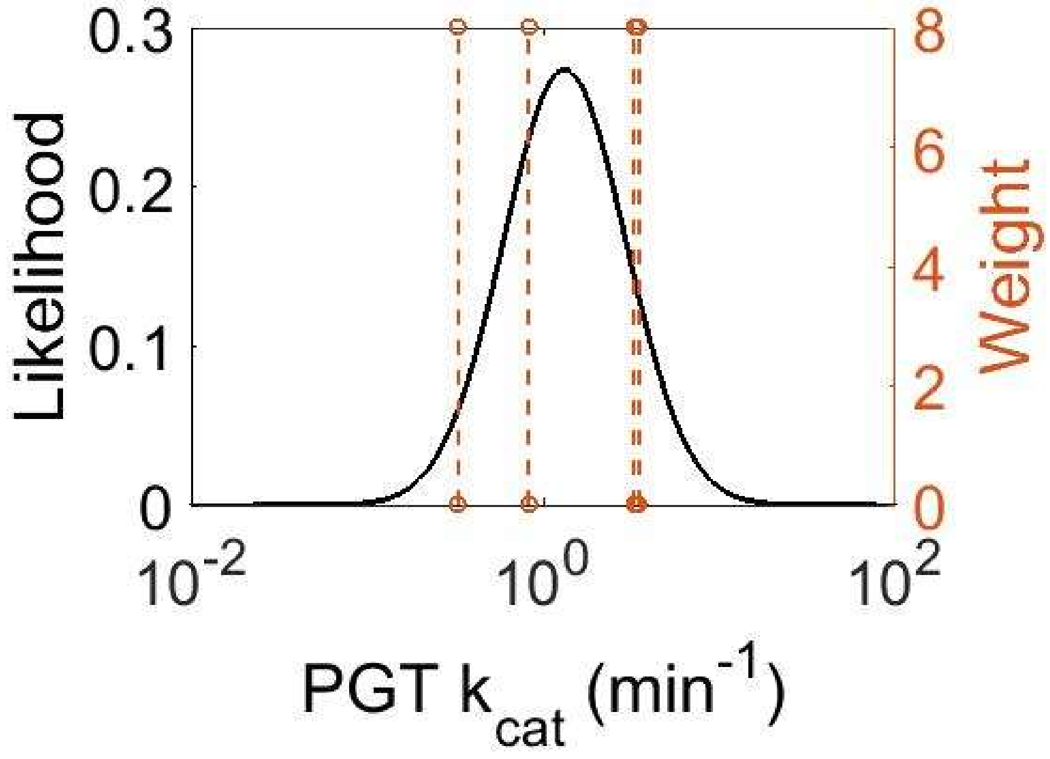


**Figure SF.10.28.1.1.1.** The estimated probability distribution for the PGT $k_{cat}$. The value and weight of the literature values used to define the distribution are indicated by an orange dashed line. The x axis is plotted on a log-scale.

### S.10.28.1.2. Parameter: PGT $K_{ms}$

Parameter values for the PGT $K_{ms}$ of Reaction 105 were obtained from the literature and summarised in Table ST.10.28.1.2.1. The log-normal distribution properties for the PGT $K_{ms}$ of Reaction 105 are shown in Table ST.10.28.1.2.2 and plotted in Figure SF.10.28.1.2.1.

**Table ST.10.28.1.2.1.** Literature information used to design the PGT $K_{ms}$ parameter distribution. Each value was assigned a weight using the protocol described in Section 2.6.2. The type of error was assigned as 0 if it was additive and 1 if it was multiplicative.

| Value (mM) | Error (mM) | Experimental details | | | | | | | Weight | Type of error | Reference |
|---|---|---|---|---|---|---|---|---|---|---|---|
| | | Substrate | Species | Expression Vector | Enzyme | pH | Temperature (°C) | Other | | | |
| 3.31 x10$^{-5}$ | 1.31 x10$^{-4}$ | | Human | Eye | PGT | 7.5 | 37 | | 512 | 0 | (Waygood and Steeves, 1980) |
| 3.76 x10$^{-4}$ | 3.40 x10$^{-5}$ | | Human | Embryonic kidney cells (HEK293) | PGT | Unknown | 37 | | 192 | 0 | (Naftalin et al., 2007) |
| 5.40 x10$^{-3}$ | NaN | | Human | Embryonic kidney cells (HEK293) | PGT | Unknown | 37 | | 2048 | 0 | (Naftalin et al., 2007) |
| 7.20 x10$^{-3}$ | 5.95 x10$^{-4}$ | | Canine | Madin-Darby Canine Kidney Epithelial Cells (MDCK) cells | PGT | Unknown | Unknown | | 512 | 0 | (Ye et al., 2001) |

| | | | | | | | | | | | |
|---|---|---|---|---|---|---|---|---|---|---|---|
| 9.40 $x10^{-5}$ | NaN | PGE2 | Human | HeLa cells | PGT | 7 | 27 | | **1024** | 0 | (Waygood and Steeves, 1980) |
| 4.23 $x10^{-4}$ | NaN | TXB2 | Human | HeLa cells | PGT | 7 | 27 | | **1024** | 0 | (Naftalin et al., 2007) |
| 7.57 $x10^{-3}$ | NaN | K6PGF1a | Human | HeLa cells | PGT | 7 | 27 | | **1024** | 0 | (Naftalin et al., 2007) |
| 4.87 $x10^{-5}$ | NaN | PGE2 | Human | HeLa cells | PGT | 7 | 27 | | **1024** | 0 | (Ye et al., 2001) |
| 5.02 $x10^{-5}$ | NaN | PGF2a | Human | HeLa cells | PGT | 7 | 27 | | **1024** | 0 | (Waygood and Steeves, 1980) |
| 5.00 $x10^{-3}$ | NaN | 15-K-PGE2 | Human | HeLa cells | PGT | 7 | 27 | | **1024** | 0 | (Naftalin et al., 2007) |
| 5.00 $x10^{-3}$ | NaN | DH-15-K-PGE2 | Human | HeLa cells | PGT | 7 | 27 | | **1024** | 0 | (Naftalin et al., 2007) |
| 3.82 $x10^{-5}$ | NaN | PGH2 | Human | HeLa cells | PGT | 7 | 27 | | **2048** | 0 | (Ye et al., 2001) |

**Table ST.10.28.1.2.2.** The log-normal distribution properties of the PGT $K_{ms}$ distribution. These values were calculated using the functions described in Section 2.6.2.

| Mode (mM) | Confidence Interval | Location parameter (μ) | Scale parameter (σ) |
|---|---|---|---|
| 1.10 $x10^{-3}$ | 9.42 $x10^{1}$ | -4.67 | 1.45 |

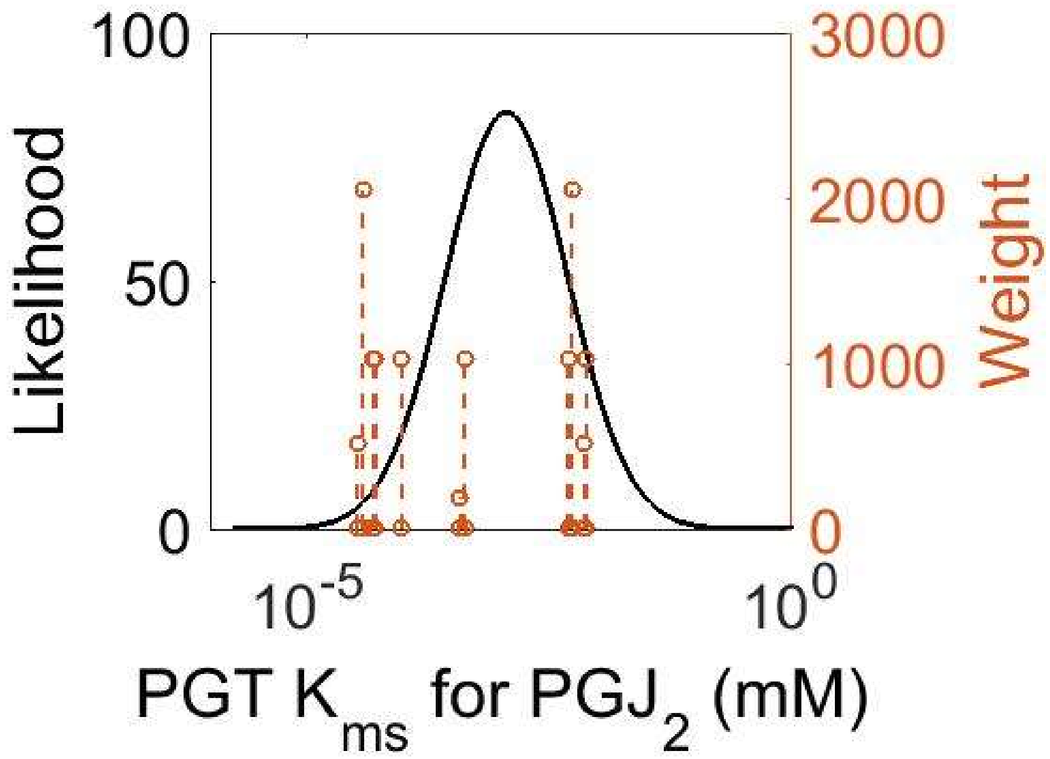


**Figure SF.10.28.1.2.1.** The estimated probability distribution for the PGT $K_{ms}$. The value and weight of the literature values used to define the distribution are indicated by an orange dashed line. The x axis is plotted on a log-scale.

### S.10.28.1.3. Parameter: PGT concentration

Parameter values for the PGT concentration of Reaction 105 were obtained from the literature and summarised in Table ST.10.28.1.3.1. The log-normal distribution properties for the PGT concentration of Reaction 105 are shown in Table ST.10.28.1.3.2 and plotted in Figure SF.10.28.1.3.1. To convert the enzyme concentration from ppm to mM, **Equation S.6.2** was used.

**Table ST.10.28.1.3.1.** Literature information used to design the PGT concentration parameter distribution. Each value was assigned a weight using the protocol described in Section 2.6.2. The type of error was assigned as 0 if it was additive and 1 if it was multiplicative.

| Value (ppm) | Error (ppm) | Experimental details | | | | | | Weight | Type of error | Reference |
|---|---|---|---|---|---|---|---|---|---|---|
| | | Species | Expression Vector | Enzyme | pH | Temperature (°C) | Other | | | |
| 2.82 | NaN | Human | Gut | PGT | 7.5 | 37 | | 2048 | 0 | (Kim et al., 2014) |
| $1.74 \times 10^{1}$ | NaN | Human | Oesophagus | PGT | 7.5 | 37 | | 2048 | 0 | (Wilhelm et al., 2014) |
| $1.25 \times 10^{2}$ | NaN | Human | Lung | PGT | 7.5 | 37 | | 2048 | 0 | (Kim et al., 2014) |

**Table ST.10.28.1.3.2.** The log-normal distribution properties of the PGT concentration distribution. These values were calculated using the functions described in Section 2.6.2.

| Mode (ppm) | Mode (mM) | Confidence Interval | Location parameter (μ) | Scale parameter (σ) |
|---|---|---|---|---|
| $1.73 \times 10^{1}$ | $9.57 \times 10^{-5}$ | 4.72 | 3.90 | 1.02 |

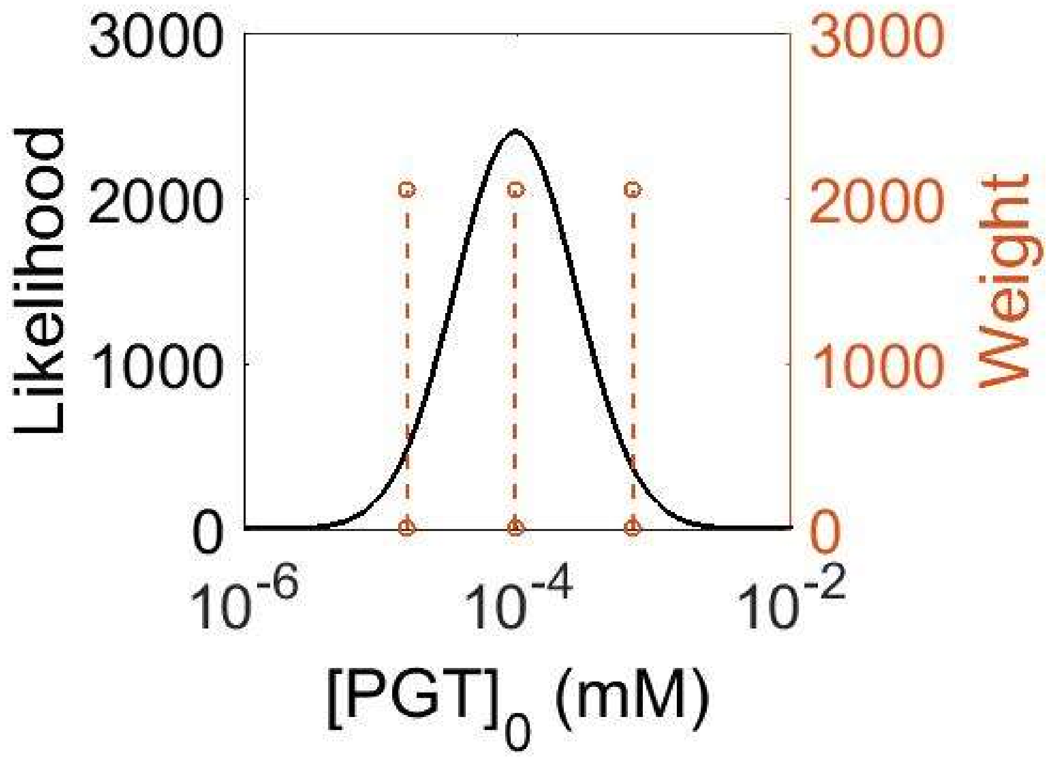


**Figure SF.10.28.1.3.1.** The estimated probability distribution for the PGT concentration. The value and weight of the literature values used to define the distribution are indicated by an orange dashed line. The x axis is plotted on a log-scale.

**S.10.29. Reaction 106: $TXB_2 \rightleftharpoons exTXB_2$ (PGT)**

$TXB_2$ is relocated from the intracellular compartment to the extracellular compartment via the prostaglandin (PGT) transporter.

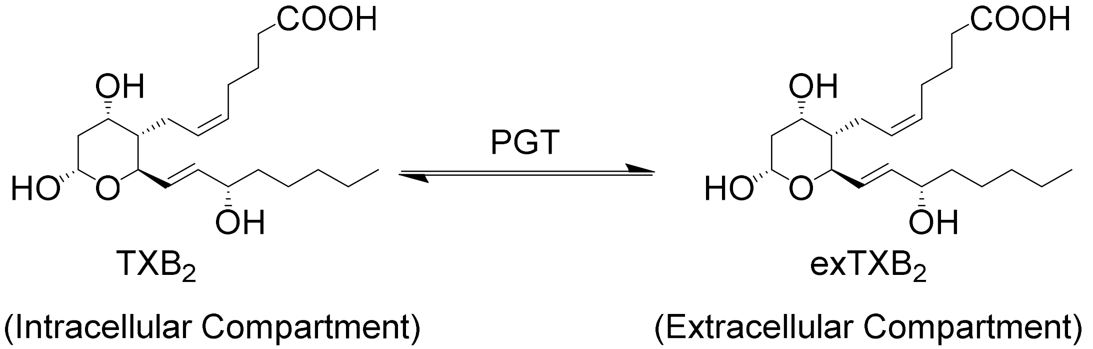


**Figure SF.10.29.** The transport of thromboxane $B_2$ ($TXB_2$) from the intracellular compartment to the extracellular compartment by prostaglandin (PGT) transporter (Reaction 106).

**SEq.10.29.** Reaction rate law for Reaction 106.

$$\boldsymbol{v_{106}} = [\boldsymbol{PGT}] \cdot \boldsymbol{k_{cat}} \cdot \frac{\mathbf{TXB_2}/K_m \cdot \left(1 - \frac{ex\mathbf{TXB_2}}{\mathbf{TXB_2} \cdot e^{-0.5 \cdot \left(G + R \cdot T \cdot \ln\left(\frac{ATP}{ADP}\right)\right)/R \cdot T}}\right)}{1 + \frac{\mathbf{TXB_2}}{K_m} + \frac{ex\mathbf{TXB_2}}{K_m} + \mathbf{PGT\_CI}}$$

### S.10.29.1. Reaction parameters

#### S.10.29.1.1. Parameter: PGT $k_{cat}$

Parameter values for the PGT $k_{cat}$ of Reaction 106 were obtained from the literature and summarised in Table ST.10.29.1.1.1. The log-normal distribution properties for the PGT $k_{cat}$ of Reaction 106 are shown in Table ST.10.29.1.1.2 and plotted in Figure SF.10.29.1.1.1.

**Table ST.10.29.1.1.1.** Literature information used to design the PGT $k_{cat}$ parameter distribution. Each value was assigned a weight using the protocol described in Section 2.6.2. The type of error was assigned as 0 if it was additive and 1 if it was multiplicative.

| Value ($min^{-1}$) | Error ($min^{-1}$) | Experimental details | | | | | | Weight | Type of error | Reference |
|---|---|---|---|---|---|---|---|---|---|---|
| | | Species | Expression Vector | Enzyme | pH | Temperature (°C) | Other | | | |
| 3.50 | NaN | Bacteria | E. coli. | Glucose transporter | Unknown | Unknown | Bionumbers calculation | **8** | 0 | (Waygood and Steeves, 1980) |
| 3.30 x10$^{-1}$ | NaN | Bacteria | E. coli. | Lactose transporter | Unknown | Unknown | Bionumbers calculation | **8** | 0 | (Naftalin et al., 2007) |
| 8.30 x10$^{-1}$ | NaN | Bacteria | E. coli. | Lactose transporter | Unknown | Unknown | Bionumbers calculation | **8** | 0 | (Naftalin et al., 2007) |
| 3.28 | NaN | Yeast | Saccharomyces cerevisiae | HXT7 transporter | Unknown | Unknown | Bionumbers calculation | **8** | 0 | (Ye et al., 2001) |

**Table ST.10.29.1.1.2** The log-normal distribution properties of the PGT $k_{cat}$ distribution. These values were calculated using the functions described in Section 2.6.2.

| Mode ($min^{-1}$) | Confidence Interval | Location parameter (μ) | Scale parameter (σ) |
|---|---|---|---|
| 1.33 | 7.30 | 9.20 x$10^{-1}$ | 8.0 $10^{-1}$ |

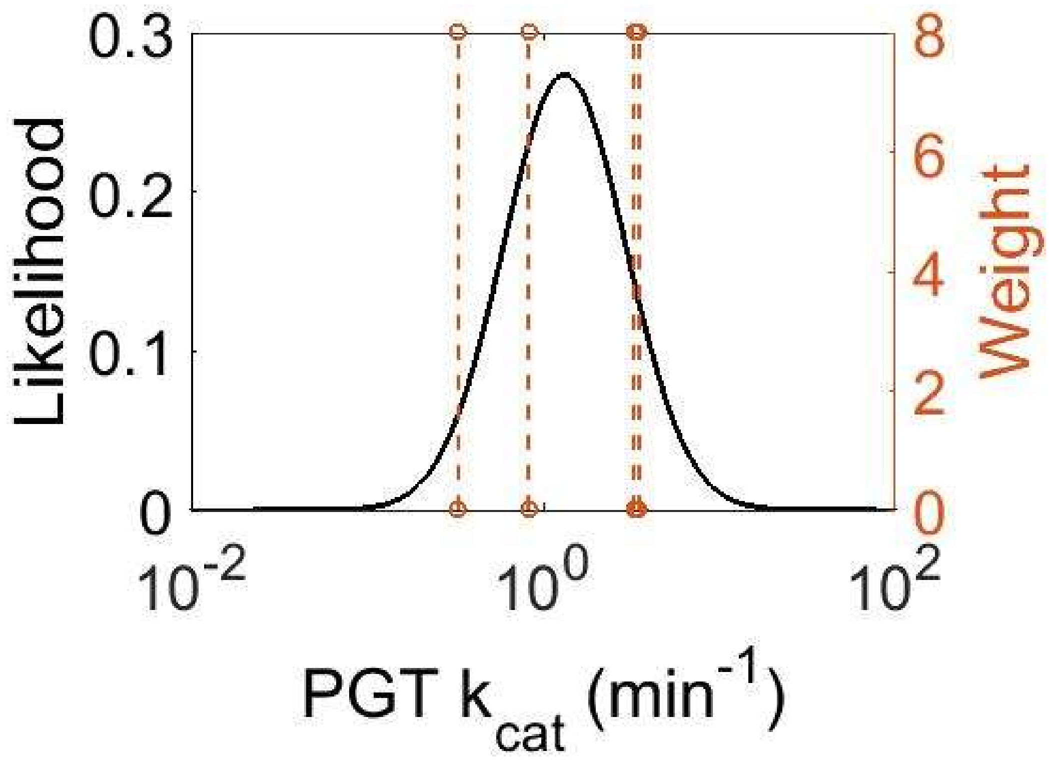


**Figure SF.10.29.1.1.1.** The estimated probability distribution for the PGT $k_{cat}$. The value and weight of the literature values used to define the distribution are indicated by an orange dashed line. The x axis is plotted on a log-scale.

**S.10.29.1.2. Parameter: PGT $K_{ms}$**

Parameter values for the PGT $K_{ms}$ of Reaction 106 were obtained from the literature and summarised in Table ST.10.29.1.2.1. The log-normal distribution properties for the PGT $K_{ms}$ of Reaction 106 are shown in Table ST.10.29.1.2.2 and plotted in Figure SF.10.29.1.2.1.

**Table ST.10.29.1.2.1.** Literature information used to design the PGT $K_{ms}$ parameter distribution. Each value was assigned a weight using the protocol described in Section 2.6.2. The type of error was assigned as 0 if it was additive and 1 if it was multiplicative.

| Value (mM) | Error (mM) | Experimental details | | | | | | | | | |
|---|---|---|---|---|---|---|---|---|---|---|---|
| | | Substrate | Species | Expression Vector | Enzyme | pH | Temperature (°C) | Other | Weight | Type of error | Reference |
| $3.31 \times 10^{-5}$ | $1.31 \times 10^{-4}$ | | Human | Eye | PGT | 7.5 | 37 | | 512 | 0 | (Waygood and Steeves, 1980) |
| $3.76 \times 10^{-4}$ | $3.40 \times 10^{-5}$ | | Human | Embryonic kidney cells (HEK293) | PGT | Unknown | 37 | | 192 | 0 | (Naftalin et al., 2007) |
| $5.40 \times 10^{-3}$ | NaN | | Human | Embryonic kidney cells (HEK293) | PGT | Unknown | 37 | | 2048 | 0 | (Naftalin et al., 2007) |
| $7.20 \times 10^{-3}$ | $5.95 \times 10^{-4}$ | | Canine | Madin-Darby Canine Kidney Epithelial Cells | PGT | Unknown | Unknown | | 512 | 0 | (Ye et al., 2001) |

| | | | | | | | | | | | |
|---|---|---|---|---|---|---|---|---|---|---|---|
| | | | | (MDCK) cells | | | | | | | |
| 9.40 x10$^{-5}$ | NaN | PGE2 | Human | HeLa cells | PGT | 7 | 27 | | **1024** | 0 | (Waygood and Steeves, 1980) |
| 4.23 x10$^{-4}$ | NaN | TXB2 | Human | HeLa cells | PGT | 7 | 27 | | **1024** | 0 | (Naftalin et al., 2007) |
| 7.57 x10$^{-3}$ | NaN | K6PGF1a | Human | HeLa cells | PGT | 7 | 27 | | **1024** | 0 | (Naftalin et al., 2007) |
| 4.87 x10$^{-5}$ | NaN | PGE2 | Human | HeLa cells | PGT | 7 | 27 | | **1024** | 0 | (Ye et al., 2001) |
| 5.02 x10$^{-5}$ | NaN | PGF2a | Human | HeLa cells | PGT | 7 | 27 | | **1024** | 0 | (Waygood and Steeves, 1980) |
| 5.00 x10$^{-3}$ | NaN | 15-K-PGE2 | Human | HeLa cells | PGT | 7 | 27 | | **1024** | 0 | (Naftalin et al., 2007) |
| 5.00 x10$^{-3}$ | NaN | DH-15-K-PGE2 | Human | HeLa cells | PGT | 7 | 27 | | **1024** | 0 | (Naftalin et al., 2007) |
| 3.82 x10$^{-5}$ | NaN | PGH2 | Human | HeLa cells | PGT | 7 | 27 | | **2048** | 0 | (Ye et al., 2001) |

**Table ST.10.29.1.2.2.** The log-normal distribution properties of the PGT $K_{ms}$ distribution. These values were calculated using the functions described in Section 2.6.2.

| Mode (mM) | Confidence Interval | Location parameter (μ) | Scale parameter (σ) |
|---|---|---|---|
| 1.10 x10$^{-3}$ | 9.42 x10$^{1}$ | -4.67 | 1.45 |

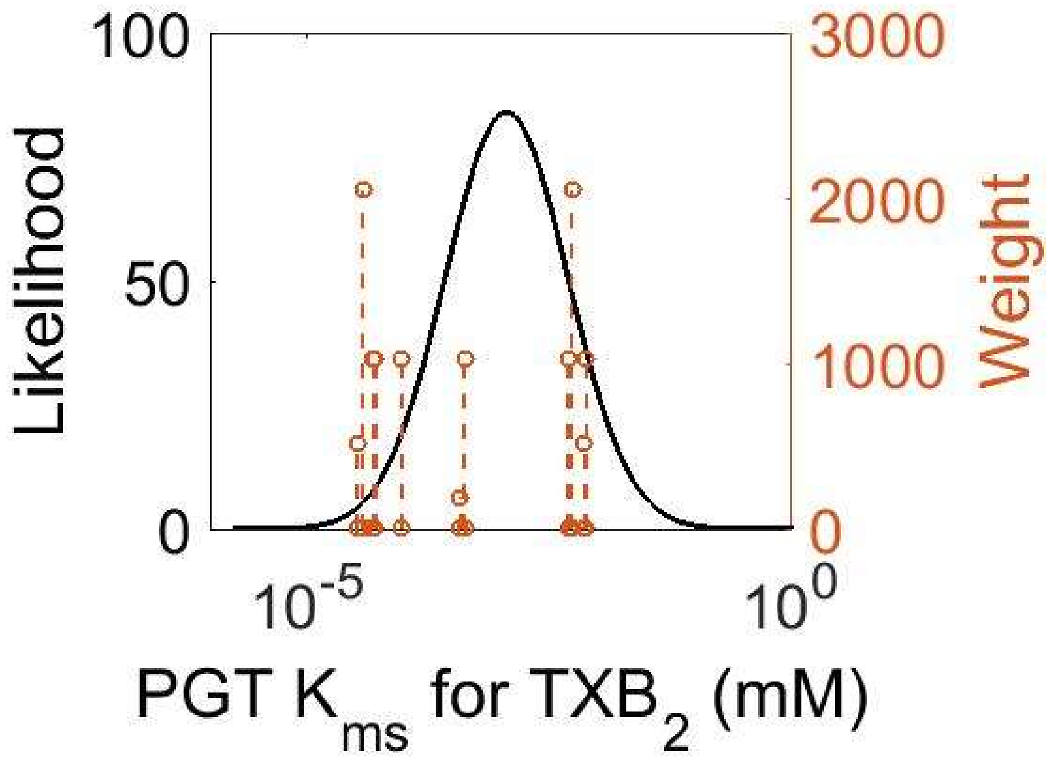


**Figure SF.10.29.1.2.1.** The estimated probability distribution for the PGT $K_{ms}$. The value and weight of the literature values used to define the distribution are indicated by an orange dashed line. The x axis is plotted on a log-scale.

**S.10.29.1.3. Parameter: PGT concentration**

Parameter values for the PGT concentration of Reaction 106 were obtained from the literature and summarised in Table ST.10.29.1.3.1. The log-normal distribution properties for the PGT concentration of Reaction 106 are shown in Table ST.10.29.1.3.2 and plotted in Figure SF.10.29.1.3.1. To convert the enzyme concentration from ppm to mM, **Equation S.6.2** was used.

**Table ST.10.29.1.3.1.** Literature information used to design the PGT concentration parameter distribution. Each value was assigned a weight using the protocol described in Section 2.6.2. The type of error was assigned as 0 if it was additive and 1 if it was multiplicative.

| Value (ppm) | Error (ppm) | Experimental details | | | | | | Weight | Type of error | Reference |
|---|---|---|---|---|---|---|---|---|---|---|
| | | Species | Expression Vector | Enzyme | pH | Temperature (°C) | Other | | | |
| 2.82 | NaN | Human | Gut | PGT | 7.5 | 37 | | 2048 | 0 | (Kim et al., 2014) |
| 1.74 $x10^1$ | NaN | Human | Oesophagus | PGT | 7.5 | 37 | | 2048 | 0 | (Wilhelm et al., 2014) |
| 1.25 $x10^2$ | NaN | Human | Lung | PGT | 7.5 | 37 | | 2048 | 0 | (Kim et al., 2014) |

**Table ST.10.29.1.3.2.** The log-normal distribution properties of the PGT concentration distribution. These values were calculated using the functions described in Section 2.6.2.

| Mode (ppm) | Mode (mM) | Confidence Interval | Location parameter (μ) | Scale parameter (σ) |
|---|---|---|---|---|
| 1.73 $x10^1$ | 9.57 $x10^{-5}$ | 4.72 | 3.90 | 1.02 |

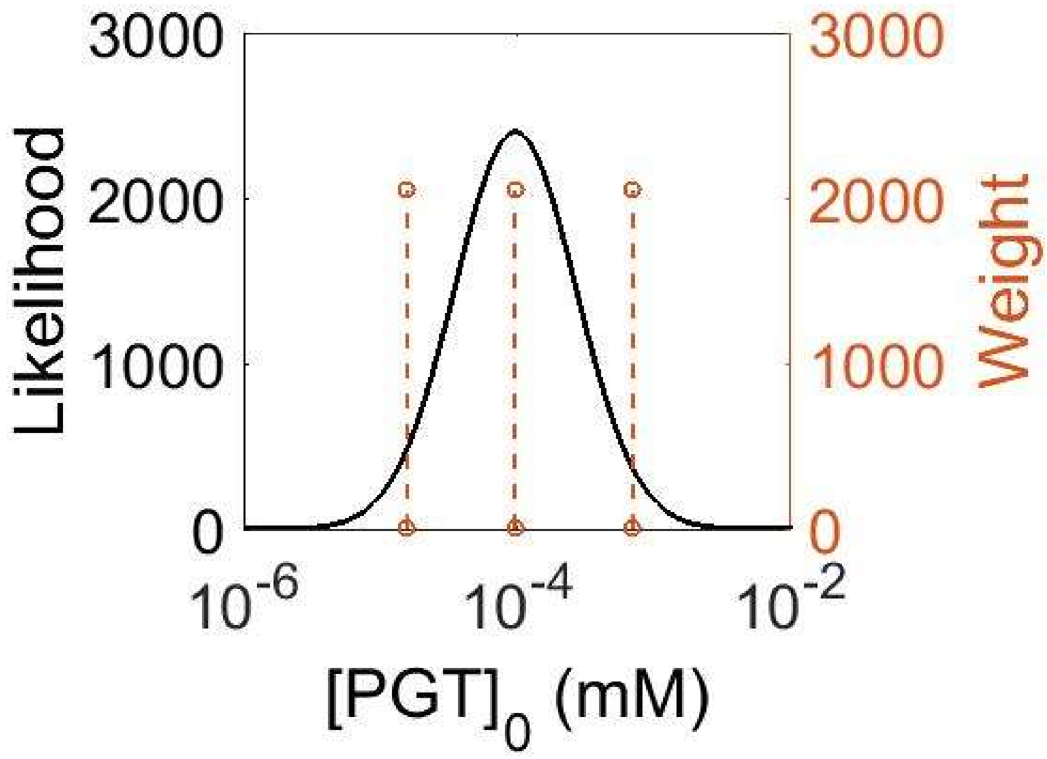


**Figure SF.10.29.1.3.1.** The estimated probability distribution for the PGT concentration. The value and weight of the literature values used to define the distribution are indicated by an orange dashed line. The x axis is plotted on a log-scale.

**S.10.30. Reaction 107: 13,14-dihydro-15-keto-PGE$_2$ ⇌ ex13,14-dihydro-15-keto-PGE$_2$ (PGT)**

13,14-dihydro-15-keto-PGE$_2$ is relocated from the intracellular compartment to the extracellular compartment via the prostaglandin (PGT) transporter.

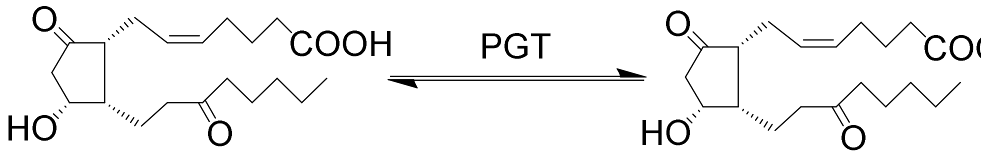


13,14-dihydro-15-keto-PGE$_2$ (Intracellular Compartment)

ex13,14-dihydro-15-keto-PGE$_2$ (Extracellular Compartment)

**Figure SF.10.30.** The transport of 13,14-dihydro-15-keto-prostaglandin E$_2$ (13,14-dihydro-15-keto-PGE$_2$) from the intracellular compartment to the extracellular compartment by prostaglandin (PGT) transporter (Reaction 107).

**SEq.10.30.** Reaction rate law for Reaction 107.

$$\boldsymbol{v_{107}} = [\boldsymbol{PGT}] \cdot \boldsymbol{k_{cat}} \cdot \frac{\text{13,14}-dihydro-\text{15}-keto-\text{PGE}_2 / K_m \cdot \left(1 - \frac{ex\text{13,14}-dihydro-\text{15}-keto-\text{PGE}_2}{\text{13,14}-dihydro-\text{15}-keto-\text{PGE}_2 \cdot e^{-0.5\cdot\left(G + R\cdot T\cdot \ln\left(\frac{ATP}{ADP}\right)\right) / R\cdot T}}\right)}{1 + \frac{\text{13,14}-dihydro-\text{15}-keto-\text{PGE}_2}{K_m} + \frac{ex\text{13,14}-dihydro-\text{15}-keto-\text{PGE}_2}{K_m} + \text{PGT\_CI}}$$

### S.10.30.1. Reaction parameters

#### S.10.30.1.1. Parameter: PGT $k_{cat}$

Parameter values for the PGT $k_{cat}$ of Reaction 107 were obtained from the literature and summarised in Table ST.10.30.1.1.1. The log-normal distribution properties for the PGT $k_{cat}$ of Reaction 107 are shown in Table ST.10.30.1.1.2 and plotted in Figure SF.10.30.1.1.1.

**Table ST.10.30.1.1.1.** Literature information used to design the PGT $k_{cat}$ parameter distribution. Each value was assigned a weight using the protocol described in Section 2.6.2. The type of error was assigned as 0 if it was additive and 1 if it was multiplicative.

| Value ($min^{-1}$) | Error ($min^{-1}$) | Experimental details | | | | | | Weight | Type of error | Reference |
|---|---|---|---|---|---|---|---|---|---|---|
| | | Species | Expression Vector | Enzyme | pH | Temperature (°C) | Other | | | |
| 3.50 | NaN | Bacteria | E. coli. | Glucose transporter | Unknown | Unknown | Bionumbers calculation | **8** | 0 | (Waygood and Steeves, 1980) |
| 3.30 $x10^{-1}$ | NaN | Bacteria | E. coli. | Lactose transporter | Unknown | Unknown | Bionumbers calculation | **8** | 0 | (Naftalin et al., 2007) |
| 8.30 $x10^{-1}$ | NaN | Bacteria | E. coli. | Lactose transporter | Unknown | Unknown | Bionumbers calculation | **8** | 0 | (Naftalin et al., 2007) |
| 3.28 | NaN | Yeast | Saccharomyces cerevisiae | HXT7 transporter | Unknown | Unknown | Bionumbers calculation | **8** | 0 | (Ye et al., 2001) |

**Table ST.10.30.1.1.2.** The log-normal distribution properties of the PGT $k_{cat}$ distribution. These values were calculated using the functions described in Section 2.6.2.

| **Mode ($min^{-1}$)** | **Confidence Interval** | **Location parameter (μ)** | **Scale parameter (σ)** |
| --- | --- | --- | --- |
| 1.33 | 7.30 | $9.20 \times 10^{-1}$ | $8.0 \times 10^{-1}$ |

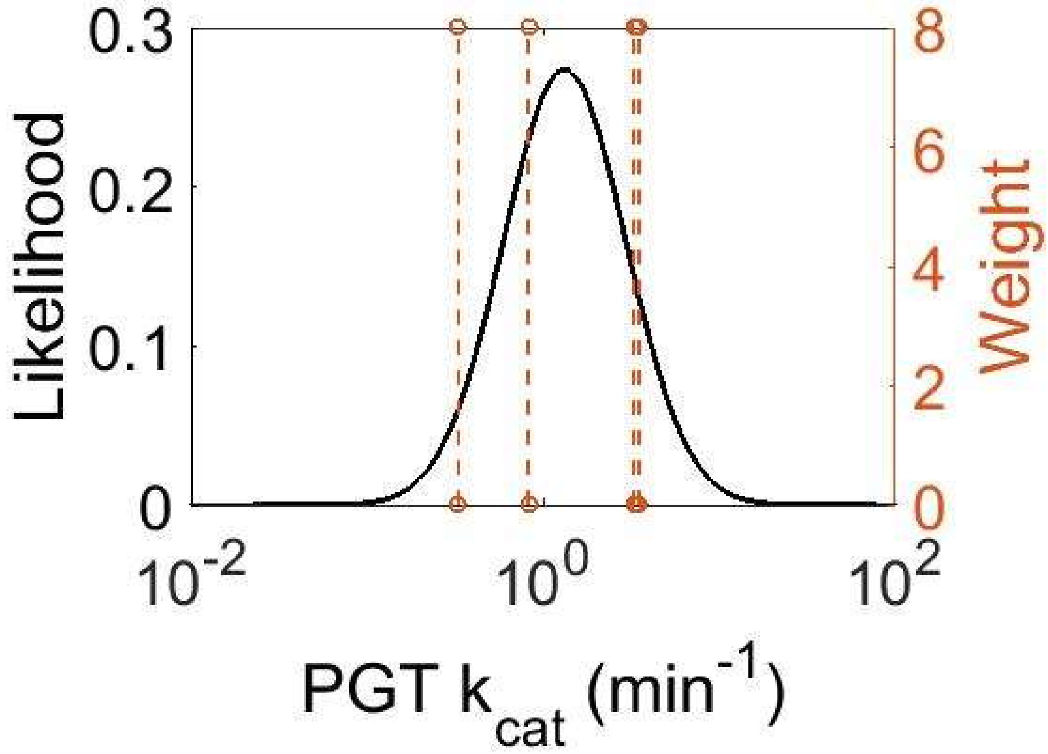


**Figure SF.10.30.1.1.1.** The estimated probability distribution for the PGT $k_{cat}$. The value and weight of the literature values used to define the distribution are indicated by an orange dashed line. The x axis is plotted on a log-scale.

**S.10.30.1.2. Parameter: PGT $K_{ms}$**

Parameter values for the PGT $K_{ms}$ of Reaction 107 were obtained from the literature and summarised in Table ST.10.30.1.2.1. The log-normal distribution properties for the PGT $K_{ms}$ of Reaction 107 are shown in Table ST.10.30.1.2.2 and plotted in Figure SF.10.30.1.2.1.

**Table ST.10.30.1.2.1.** Literature information used to design the PGT $K_{ms}$ parameter distribution. Each value was assigned a weight using the protocol described in Section 2.6.2. The type of error was assigned as 0 if it was additive and 1 if it was multiplicative.

| Value (mM) | Error (mM) | Experimental details | | | | | | | | | |
|---|---|---|---|---|---|---|---|---|---|---|---|
| | | Substrate | Species | Expression Vector | Enzyme | pH | Temperature (°C) | Other | Weight | Type of error | Reference |
| $3.31 \times 10^{-5}$ | $1.31 \times 10^{-4}$ | | Human | Eye | PGT | 7.5 | 37 | | 512 | 0 | (Waygood and Steeves, 1980) |
| $3.76 \times 10^{-4}$ | $3.40 \times 10^{-5}$ | | Human | Embryonic kidney cells (HEK293) | PGT | Unknown | 37 | | 192 | 0 | (Naftalin et al., 2007) |
| $5.40 \times 10^{-3}$ | NaN | | Human | Embryonic kidney cells (HEK293) | PGT | Unknown | 37 | | 2048 | 0 | (Naftalin et al., 2007) |
| $7.20 \times 10^{-3}$ | $5.95 \times 10^{-4}$ | | Canine | Madin-Darby Canine Kidney Epithelial Cells | PGT | Unknown | Unknown | | 512 | 0 | (Ye et al., 2001) |

| | | | | | | | | | | | |
|---|---|---|---|---|---|---|---|---|---|---|---|
| | | | | (MDCK) cells | | | | | | | |
| 9.40 x10$^{-5}$ | NaN | PGE2 | Human | HeLa cells | PGT | 7 | 27 | | **1024** | 0 | (Waygood and Steeves, 1980) |
| 4.23 x10$^{-4}$ | NaN | TXB2 | Human | HeLa cells | PGT | 7 | 27 | | **1024** | 0 | (Naftalin et al., 2007) |
| 7.57 x10$^{-3}$ | NaN | K6PGF1a | Human | HeLa cells | PGT | 7 | 27 | | **1024** | 0 | (Naftalin et al., 2007) |
| 4.87 x10$^{-5}$ | NaN | PGE2 | Human | HeLa cells | PGT | 7 | 27 | | **1024** | 0 | (Ye et al., 2001) |
| 5.02 x10$^{-5}$ | NaN | PGF2a | Human | HeLa cells | PGT | 7 | 27 | | **1024** | 0 | (Waygood and Steeves, 1980) |
| 5.00 x10$^{-3}$ | NaN | 15-K-PGE2 | Human | HeLa cells | PGT | 7 | 27 | | **1024** | 0 | (Naftalin et al., 2007) |
| 5.00 x10$^{-3}$ | NaN | DH-15-K-PGE2 | Human | HeLa cells | PGT | 7 | 27 | | **1024** | 0 | (Naftalin et al., 2007) |
| 3.82 x10$^{-5}$ | NaN | PGH2 | Human | HeLa cells | PGT | 7 | 27 | | **2048** | 0 | (Ye et al., 2001) |

**Table ST.10.30.1.2.2.** The log-normal distribution properties of the PGT $K_{ms}$ distribution. These values were calculated using the functions described in Section 2.6.2.

| **Mode (mM)** | **Confidence Interval** | **Location parameter (μ)** | **Scale parameter (σ)** |
|---|---|---|---|
| 1.10 x10$^{-3}$ | 9.42 x10$^{1}$ | -4.67 | 1.45 |

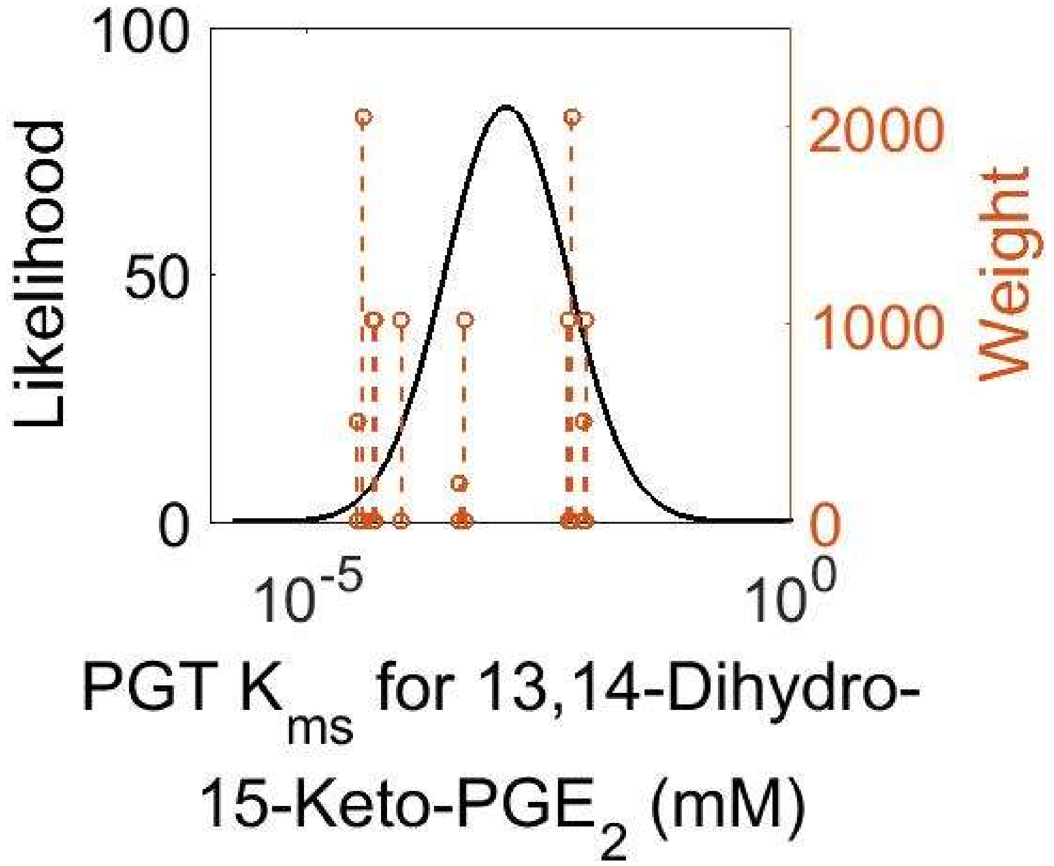


**Figure SF.10.30.1.2.1.** The estimated probability distribution for the PGT $K_{ms}$. The value and weight of the literature values used to define the distribution are indicated by an orange dashed line. The x axis is plotted on a log-scale.

**S.10.30.1.3. Parameter: PGT concentration**

Parameter values for the PGT concentration of Reaction 107 were obtained from the literature and summarised in Table ST.10.30.1.3.1. The log-normal distribution properties for the PGT concentration of Reaction 107 are shown in Table ST.10.30.1.3.2 and plotted in Figure SF.10.30.1.3.1. To convert the enzyme concentration from ppm to mM, **Equation S.6.2** was used.

**Table ST.10.30.1.3.1.** Literature information used to design the PGT concentration parameter distribution. Each value was assigned a weight using the protocol described in Section 2.6.2. The type of error was assigned as 0 if it was additive and 1 if it was multiplicative.

| Value (ppm) | Error (ppm) | Experimental details | | | | | | Weight | Type of error | Reference |
|---|---|---|---|---|---|---|---|---|---|---|
| | | Species | Expression Vector | Enzyme | pH | Temperature (°C) | Other | | | |
| 2.82 | NaN | Human | Gut | PGT | 7.5 | 37 | | 2048 | 0 | (Kim et al., 2014) |
| 1.74 $x10^1$ | NaN | Human | Oesophagus | PGT | 7.5 | 37 | | 2048 | 0 | (Wilhelm et al., 2014) |
| 1.25 $x10^2$ | NaN | Human | Lung | PGT | 7.5 | 37 | | 2048 | 0 | (Kim et al., 2014) |

**Table ST.10.30.1.3.2.** The log-normal distribution properties of the PGT concentration distribution. These values were calculated using the functions described in Section 2.6.2.

| Mode (ppm) | Mode (mM) | Confidence Interval | Location parameter (μ) | Scale parameter (σ) |
|---|---|---|---|---|
| 1.73 $x10^1$ | 9.57 $x10^{-5}$ | 4.72 | 3.90 | 1.02 |

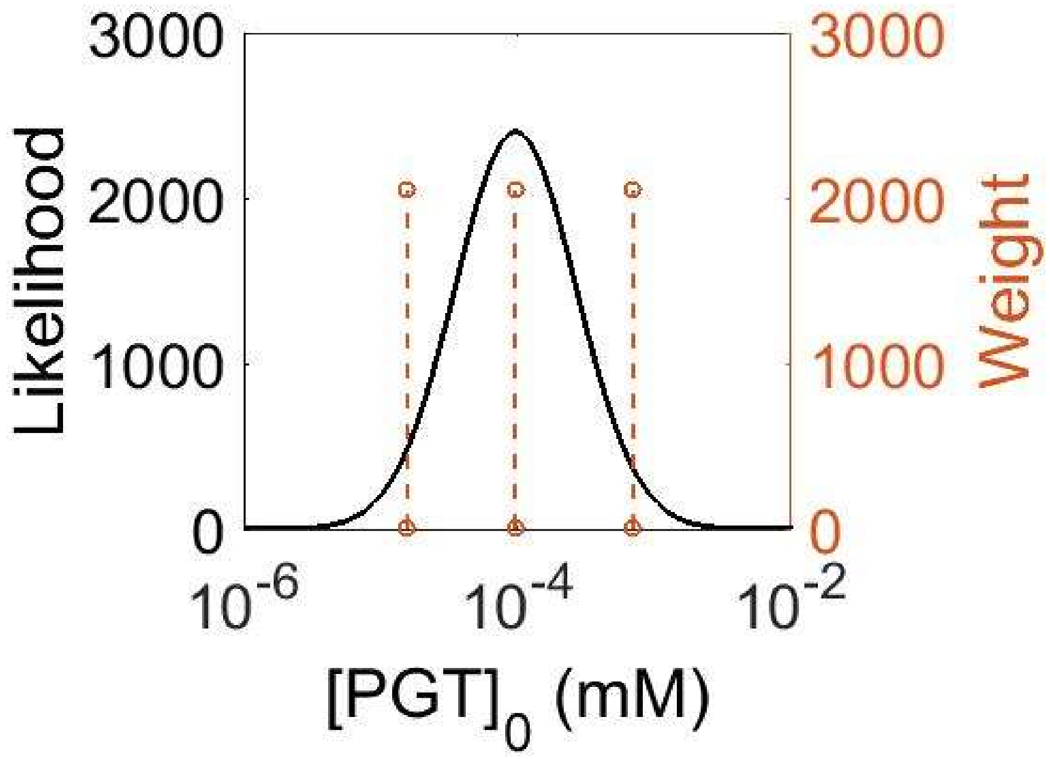


**Figure SF.10.30.1.3.1.** The estimated probability distribution for the PGT concentration. The value and weight of the literature values used to define the distribution are indicated by an orange dashed line. The x axis is plotted on a log-scale.

**S.10.31. Reaction 108: 15-keto-PGE2 ⇌ ex15-keto-PGE2 (PGT)**

15-keto-PGE2 is relocated from the intracellular compartment to the extracellular compartment via the prostaglandin (PGT) transporter.

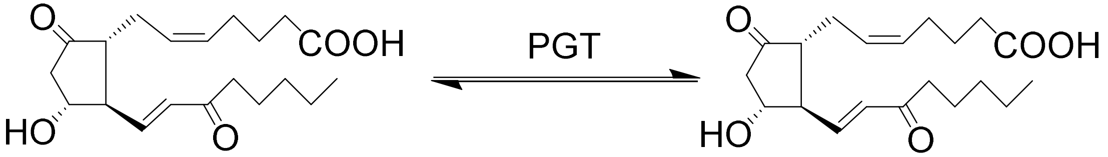


15-keto-$PGE_2$ (Intracellular Compartment)

ex15-keto-$PGE_2$ (Extracellular Compartment)

**Figure SF.10.31.** The transport of 15-keto-prostaglandin $E_2$ (15-keto-$PGE_2$) from the intracellular compartment to the extracellular compartment by prostaglandin (PGT) transporter (Reaction 108).

**SEq.10.31.** Reaction rate law for Reaction 108.

$$\boldsymbol{v_{108}} = [\boldsymbol{PGT}] \cdot \boldsymbol{k_{cat}} \cdot \frac{\text{15−keto−PGE}_2 / K_m \cdot \left( 1 - \frac{\text{ex15−keto−PGE}_2}{\text{15−keto−PGE}_2 \cdot e^{-0.5 \cdot \left(G + R \cdot T \cdot \ln\left(\frac{ATP}{ADP}\right)\right) / R \cdot T}} \right)}{1 + \frac{\text{15−keto−PGE}_2}{K_m} + \frac{\text{ex15−keto−PGE}_2}{K_m} + \text{PGT\_CI}}$$

## S.10.31.1. Reaction parameters

### S.10.31.1.1. Parameter: PGT $k_{cat}$

Parameter values for the PGT $k_{cat}$ of Reaction 108 were obtained from the literature and summarised in Table ST.10.31.1.1.1. The log-normal distribution properties for the PGT $k_{cat}$ of Reaction 108 are shown in Table ST.10.31.1.1.2 and plotted in Figure SF.10.31.1.1.1.

**Table ST.10.31.1.1.1.** Literature information used to design the PGT $k_{cat}$ parameter distribution. Each value was assigned a weight using the protocol described in Section 2.6.2. The type of error was assigned as 0 if it was additive and 1 if it was multiplicative.

| Value ($min^{-1}$) | Error ($min^{-1}$) | Experimental details | | | | | | Weight | Type of error | Reference |
|---|---|---|---|---|---|---|---|---|---|---|
| | | Species | Expression Vector | Enzyme | pH | Temperature (°C) | Other | | | |
| 3.50 | NaN | Bacteria | E. coli. | Glucose transporter | Unknown | Unknown | Bionumbers calculation | **8** | 0 | (Waygood and Steeves, 1980) |
| 3.30 x10$^{-1}$ | NaN | Bacteria | E. coli. | Lactose transporter | Unknown | Unknown | Bionumbers calculation | **8** | 0 | (Naftalin et al., 2007) |
| 8.30 x10$^{-1}$ | NaN | Bacteria | E. coli. | Lactose transporter | Unknown | Unknown | Bionumbers calculation | **8** | 0 | (Naftalin et al., 2007) |
| 3.28 | NaN | Yeast | Saccharomyces cerevisiae | HXT7 transporter | Unknown | Unknown | Bionumbers calculation | **8** | 0 | (Ye et al., 2001) |

**Table ST.10.31.1.1.2.** The log-normal distribution properties of the PGT $k_{cat}$ distribution. These values were calculated using the functions described in Section 2.6.2.

| **Mode ($min^{-1}$)** | **Confidence Interval** | **Location parameter (μ)** | **Scale parameter (σ)** |
|---|---|---|---|
| 1.33 | 7.30 | 9.20 $x10^{-1}$ | 8.0 $x10^{-1}$ |

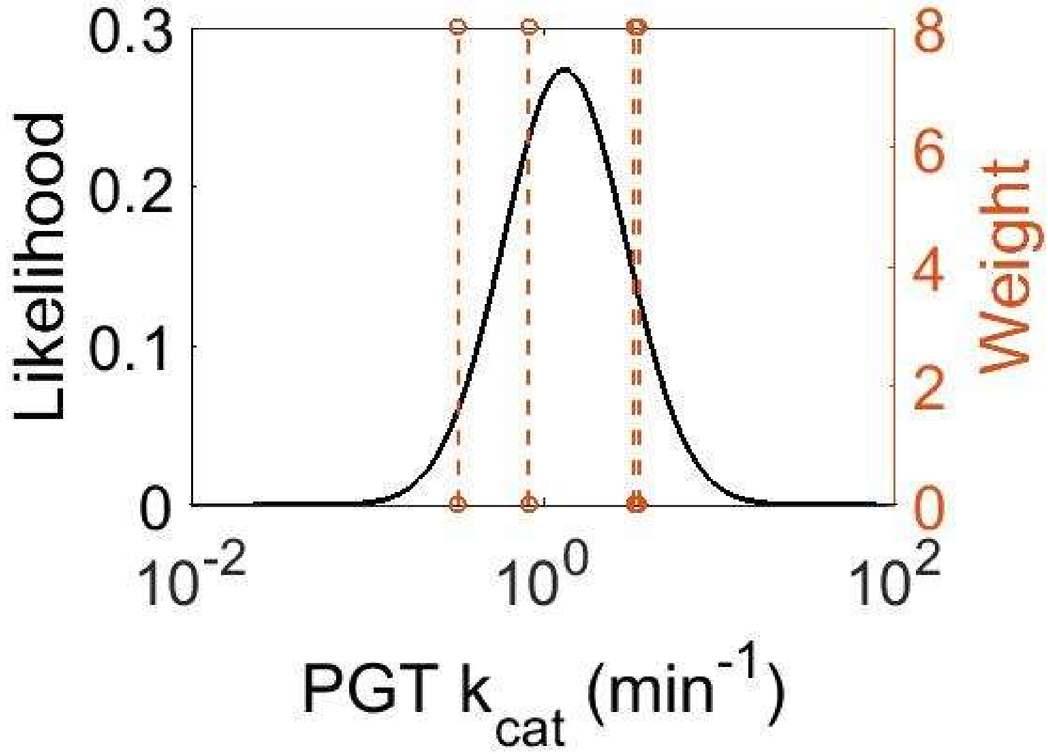


**Figure SF.10.31.1.1.1.** The estimated probability distribution for the PGT $k_{cat}$. The value and weight of the literature values used to define the distribution are indicated by an orange dashed line. The x axis is plotted on a log-scale.

**S.10.31.1.2. Parameter: PGT $K_{ms}$**

Parameter values for the PGT $K_{ms}$ of Reaction 108 were obtained from the literature and summarised in Table ST.10.31.1.2.1. The log-normal distribution properties for the PGT $K_{ms}$ of Reaction 108 are shown in Table ST.10.31.1.2.2 and plotted in Figure SF.10.31.1.2.1.

**Table ST.10.31.1.2.1.** Literature information used to design the PGT $K_{ms}$ parameter distribution. Each value was assigned a weight using the protocol described in Section 2.6.2. The type of error was assigned as 0 if it was additive and 1 if it was multiplicative.

| Value (mM) | Error (mM) | Experimental details | | | | | | | | | |
|---|---|---|---|---|---|---|---|---|---|---|---|
| | | Substrate | Species | Expression Vector | Enzyme | pH | Temperature (°C) | Other | Weight | Type of error | Reference |
| $3.31 \times 10^{-5}$ | $1.31 \times 10^{-4}$ | | Human | Eye | PGT | 7.5 | 37 | | 512 | 0 | (Waygood and Steeves, 1980) |
| $3.76 \times 10^{-4}$ | $3.40 \times 10^{-5}$ | | Human | Embryonic kidney cells (HEK293) | PGT | Unknown | 37 | | 192 | 0 | (Naftalin et al., 2007) |
| $5.40 \times 10^{-3}$ | NaN | | Human | Embryonic kidney cells (HEK293) | PGT | Unknown | 37 | | 2048 | 0 | (Naftalin et al., 2007) |
| $7.20 \times 10^{-3}$ | $5.95 \times 10^{-4}$ | | Canine | Madin-Darby Canine Kidney Epithelial Cells | PGT | Unknown | Unknown | | 512 | 0 | (Ye et al., 2001) |

| | | | | | | | | | | | |
|---|---|---|---|---|---|---|---|---|---|---|---|
| | | | | (MDCK) cells | | | | | | | |
| 9.40 x$10^{-5}$ | NaN | PGE2 | Human | HeLa cells | PGT | 7 | 27 | | **1024** | 0 | (Waygood and Steeves, 1980) |
| 4.23 x$10^{-4}$ | NaN | TXB2 | Human | HeLa cells | PGT | 7 | 27 | | **1024** | 0 | (Naftalin et al., 2007) |
| 7.57 x$10^{-3}$ | NaN | K6PGF1a | Human | HeLa cells | PGT | 7 | 27 | | **1024** | 0 | (Naftalin et al., 2007) |
| 4.87 x$10^{-5}$ | NaN | PGE2 | Human | HeLa cells | PGT | 7 | 27 | | **1024** | 0 | (Ye et al., 2001) |
| 5.02 x$10^{-5}$ | NaN | PGF2a | Human | HeLa cells | PGT | 7 | 27 | | **1024** | 0 | (Waygood and Steeves, 1980) |
| 5.00 x$10^{-3}$ | NaN | 15-K-PGE2 | Human | HeLa cells | PGT | 7 | 27 | | **1024** | 0 | (Naftalin et al., 2007) |
| 5.00 x$10^{-3}$ | NaN | DH-15-K-PGE2 | Human | HeLa cells | PGT | 7 | 27 | | **1024** | 0 | (Naftalin et al., 2007) |
| 3.82 x$10^{-5}$ | NaN | PGH2 | Human | HeLa cells | PGT | 7 | 27 | | **2048** | 0 | (Ye et al., 2001) |

**Table ST.10.31.1.2.2.** The log-normal distribution properties of the PGT $K_{ms}$ distribution. These values were calculated using the functions described in Section 2.6.2.

| **Mode (mM)** | **Confidence Interval** | **Location parameter (μ)** | **Scale parameter (σ)** |
|---|---|---|---|
| 1.10 x$10^{-3}$ | 9.42 x$10^{1}$ | -4.67 | 1.45 |

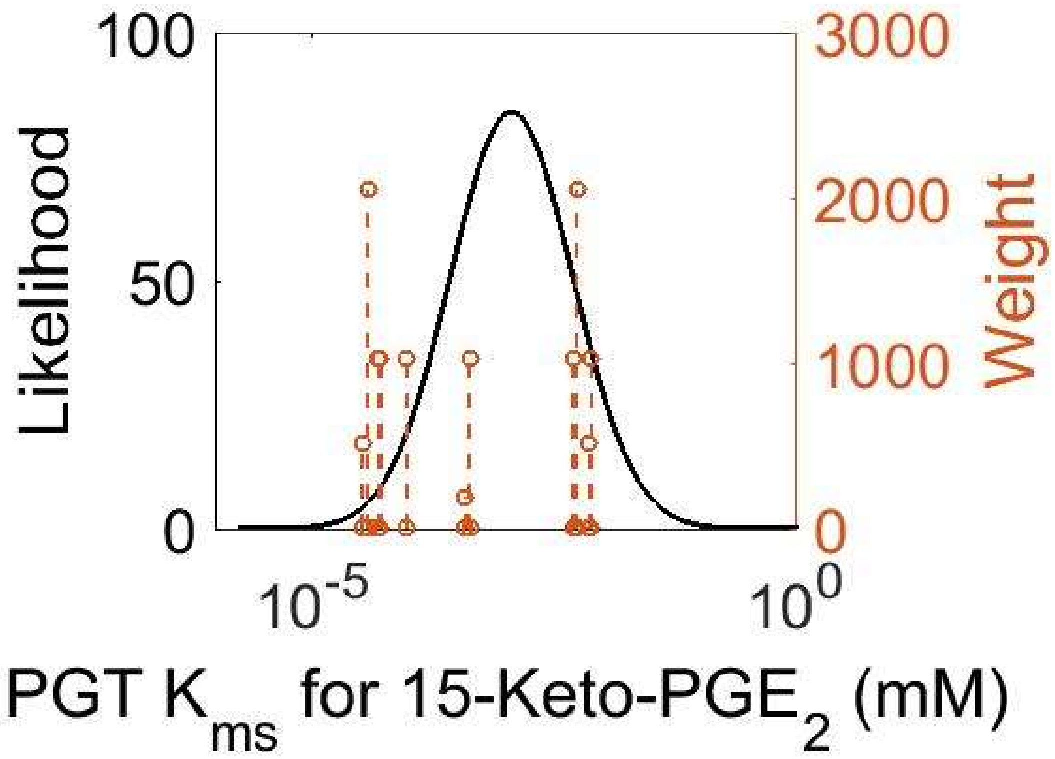


**Figure SF.10.31.1.2.1.** The estimated probability distribution for the PGT $K_{ms}$. The value and weight of the literature values used to define the distribution are indicated by an orange dashed line. The x axis is plotted on a log-scale.

**S.10.31.1.3. Parameter: PGT concentration**

Parameter values for the PGT concentration of Reaction 108 were obtained from the literature and summarised in Table ST.10.31.1.3.1. The log-normal distribution properties for the PGT concentration of Reaction 108 are shown in Table ST.10.31.1.3.2 and plotted in Figure SF.10.31.1.3.1. To convert the enzyme concentration from ppm to mM, **Equation S.6.2** was used.

**Table ST.10.31.1.3.1.** Literature information used to design the PGT concentration parameter distribution. Each value was assigned a weight using the protocol described in Section 2.6.2. The type of error was assigned as 0 if it was additive and 1 if it was multiplicative.

| Value (ppm) | Error (ppm) | Experimental details | | | | | | Weight | Type of error | Reference |
|---|---|---|---|---|---|---|---|---|---|---|
| | | Species | Expression Vector | Enzyme | pH | Temperature (°C) | Other | | | |
| 2.82 | NaN | Human | Gut | PGT | 7.5 | 37 | | 2048 | 0 | (Kim et al., 2014) |
| $1.74 \times 10^{1}$ | NaN | Human | Oesophagus | PGT | 7.5 | 37 | | 2048 | 0 | (Wilhelm et al., 2014) |
| $1.25 \times 10^{2}$ | NaN | Human | Lung | PGT | 7.5 | 37 | | 2048 | 0 | (Kim et al., 2014) |

**Table ST.10.31.1.3.2.** The log-normal distribution properties of the PGT concentration distribution. These values were calculated using the functions described in Section 2.6.2.

| Mode (ppm) | Mode (mM) | Confidence Interval | Location parameter (μ) | Scale parameter (σ) |
|---|---|---|---|---|
| $1.73 \times 10^{1}$ | $9.57 \times 10^{-5}$ | 4.72 | 3.90 | 1.02 |

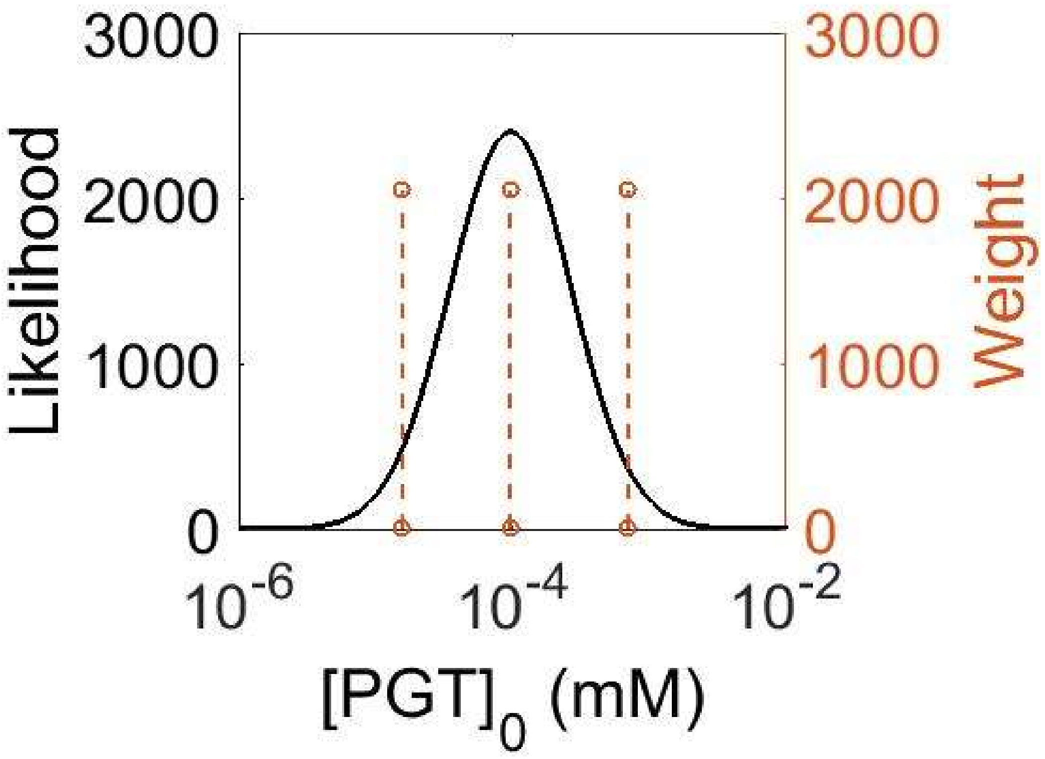


**Figure SF.10.31.1.3.1.** The estimated probability distribution for the PGT concentration. The value and weight of the literature values used to define the distribution are indicated by an orange dashed line. The x axis is plotted on a log-scale.

**S.10.32. Reaction 109: 6-keto-PGF$_{1\alpha}$ ⇌ ex6-keto-PGF$_{1\alpha}$ (PGT)**

6-keto-PGF$_{1\alpha}$ is relocated from the intracellular compartment to the extracellular compartment via the prostaglandin (PGT) transporter.

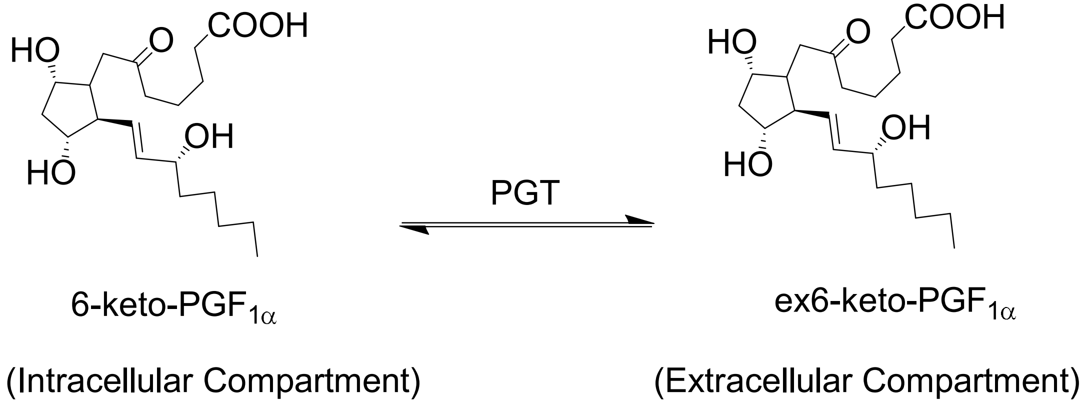


**Figure SF.10.32.** The transport of 6-keto-prostaglandin F$_{1\alpha}$ (6-keto-PGF$_{1\alpha}$) from the intracellular compartment to the extracellular compartment by prostaglandin (PGT) transporter (Reaction 109).

**SEq.10.32.** Reaction rate law for Reaction 109.

$$v_{109} = [PGT] \cdot k_{cat} \cdot \frac{\text{6-keto-PGF}_{1\alpha}/K_m \cdot \left(1 - \frac{\text{ex6-keto-PGF}_{1\alpha}}{\text{6-keto-PGF}_{1\alpha} \cdot e^{-0.5 \cdot \left(G + R \cdot T \cdot \ln\left(\frac{ATP}{ADP}\right)\right)/R \cdot T}}\right)}{1 + \frac{\text{6-keto-PGF}_{1\alpha}}{K_m} + \frac{\text{ex6-keto-PGF}_{1\alpha}}{K_m} + \text{PGT\_CI}}$$

### S.10.32.1. Reaction parameters

#### S.10.32.1.1. Parameter: PGT $k_{cat}$

Parameter values for the PGT $k_{cat}$ of Reaction 109 were obtained from the literature and summarised in Table ST.10.32.1.1.1. The log-normal distribution properties for the PGT $k_{cat}$ of Reaction 109 are shown in Table ST.10.32.1.1.2 and plotted in Figure SF.10.32.1.1.1.

**Table ST.10.32.1.1.1.** Literature information used to design the PGT $k_{cat}$ parameter distribution. Each value was assigned a weight using the protocol described in Section 2.6.2. The type of error was assigned as 0 if it was additive and 1 if it was multiplicative.

| Value ($min^{-1}$) | Error ($min^{-1}$) | Experimental details | | | | | | Weight | Type of error | Reference |
|---|---|---|---|---|---|---|---|---|---|---|
| | | Species | Expression Vector | Enzyme | pH | Temperature (°C) | Other | | | |
| 3.50 | NaN | Bacteria | E. coli. | Glucose transporter | Unknown | Unknown | Bionumbers calculation | **8** | 0 | (Waygood and Steeves, 1980) |
| 3.30 x10$^{-1}$ | NaN | Bacteria | E. coli. | Lactose transporter | Unknown | Unknown | Bionumbers calculation | **8** | 0 | (Naftalin et al., 2007) |
| 8.30 x10$^{-1}$ | NaN | Bacteria | E. coli. | Lactose transporter | Unknown | Unknown | Bionumbers calculation | **8** | 0 | (Naftalin et al., 2007) |
| 3.28 | NaN | Yeast | Saccharomyces cerevisiae | HXT7 transporter | Unknown | Unknown | Bionumbers calculation | **8** | 0 | (Ye et al., 2001) |

**Table ST.10.32.1.1.2.** The log-normal distribution properties of the PGT $K_{ms}$ distribution. These values were calculated using the functions described in Section 2.6.2.

| **Mode ($min^{-1}$)** | **Confidence Interval** | **Location parameter (μ)** | **Scale parameter (σ)** |
|---|---|---|---|
| 1.33 | 7.30 | $9.20 \times 10^{-1}$ | $8.0 \times 10^{-1}$ |

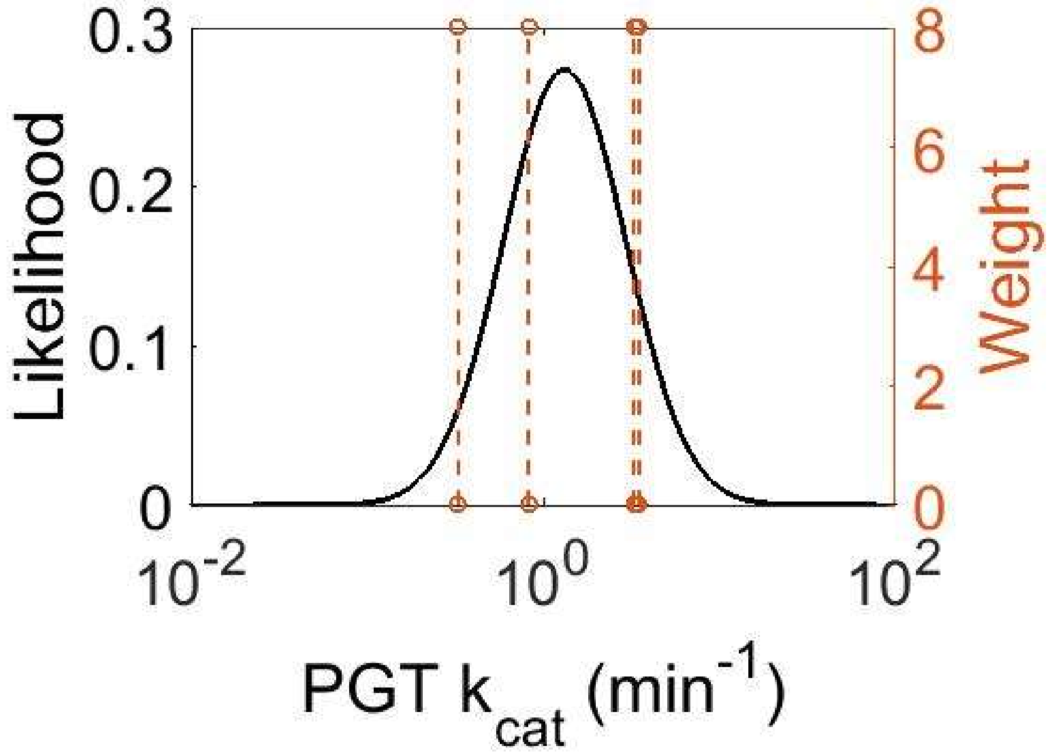


**Figure SF.10.32.1.1.1.** The estimated probability distribution for the PGT $k_{cat}$. The value and weight of the literature values used to define the distribution are indicated by an orange dashed line. The x axis is plotted on a log-scale.

### S.10.32.1.2. Parameter: PGT $K_{ms}$

Parameter values for the PGT $K_{ms}$ of Reaction 109 were obtained from the literature and summarised in Table ST.10.32.1.2.1. The log-normal distribution properties for the PGT $K_{ms}$ of Reaction 109 are shown in Table ST.10.32.1.2.2 and plotted in Figure SF.10.32.1.2.1.

**Table ST.10.32.1.2.1.** Literature information used to design the PGT $K_{ms}$ parameter distribution. Each value was assigned a weight using the protocol described in Section 2.6.2. The type of error was assigned as 0 if it was additive and 1 if it was multiplicative.

| Value (mM) | Error (mM) | Experimental details | | | | | | Other | Weight | Type of error | Reference |
|---|---|---|---|---|---|---|---|---|---|---|---|
| | | Substrate | Species | Expression Vector | Enzyme | pH | Temperature (°C) | | | | |
| $3.31 \times 10^{-5}$ | $1.31 \times 10^{-4}$ | | Human | Eye | PGT | 7.5 | 37 | | 512 | 0 | (Waygood and Steeves, 1980) |
| $3.76 \times 10^{-4}$ | $3.40 \times 10^{-5}$ | | Human | Embryonic kidney cells (HEK293) | PGT | Unknown | 37 | | 192 | 0 | (Naftalin et al., 2007) |
| $5.40 \times 10^{-3}$ | NaN | | Human | Embryonic kidney cells (HEK293) | PGT | Unknown | 37 | | 2048 | 0 | (Naftalin et al., 2007) |
| $7.20 \times 10^{-3}$ | $5.95 \times 10^{-4}$ | | Canine | Madin-Darby Canine Kidney Epithelial Cells (MDCK) cells | PGT | Unknown | Unknown | | 512 | 0 | (Ye et al., 2001) |

| | | | | | | | | | | | |
|---|---|---|---|---|---|---|---|---|---|---|---|
| $9.40 \times 10^{-5}$ | NaN | PGE2 | Human | HeLa cells | PGT | 7 | 27 | | **1024** | 0 | (Waygood and Steeves, 1980) |
| $4.23 \times 10^{-4}$ | NaN | TXB2 | Human | HeLa cells | PGT | 7 | 27 | | **1024** | 0 | (Naftalin et al., 2007) |
| $7.57 \times 10^{-3}$ | NaN | K6PGF1a | Human | HeLa cells | PGT | 7 | 27 | | **1024** | 0 | (Naftalin et al., 2007) |
| $4.87 \times 10^{-5}$ | NaN | PGE2 | Human | HeLa cells | PGT | 7 | 27 | | **1024** | 0 | (Ye et al., 2001) |
| $5.02 \times 10^{-5}$ | NaN | PGF2a | Human | HeLa cells | PGT | 7 | 27 | | **1024** | 0 | (Waygood and Steeves, 1980) |
| $5.00 \times 10^{-3}$ | NaN | 15-K-PGE2 | Human | HeLa cells | PGT | 7 | 27 | | **1024** | 0 | (Naftalin et al., 2007) |
| $5.00 \times 10^{-3}$ | NaN | DH-15-K-PGE2 | Human | HeLa cells | PGT | 7 | 27 | | **1024** | 0 | (Naftalin et al., 2007) |
| $3.82 \times 10^{-5}$ | NaN | PGH2 | Human | HeLa cells | PGT | 7 | 27 | | **2048** | 0 | (Ye et al., 2001) |

**Table ST.10.32.1.2.2.** The log-normal distribution properties of the PGT $K_{ms}$ distribution. These values were calculated using the functions described in Section 2.6.2.

| **Mode (mM)** | **Confidence Interval** | **Location parameter (μ)** | **Scale parameter (σ)** |
|---|---|---|---|
| $1.10 \times 10^{-3}$ | $9.42 \times 10^{1}$ | -4.67 | 1.45 |

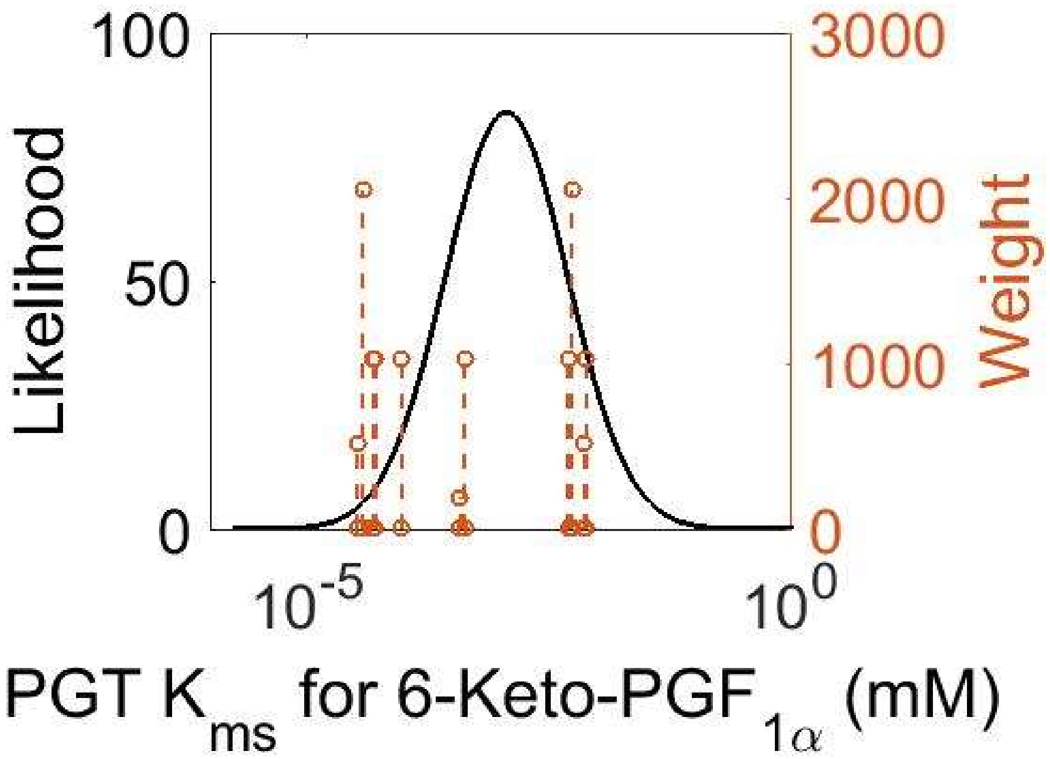


**Figure SF.10.32.1.2.1.** The estimated probability distribution for the PGT $K_{ms}$. The value and weight of the literature values used to define the distribution are indicated by an orange dashed line. The x axis is plotted on a log-scale.

**S.10.32.1.3. Parameter: PGT concentration**

Parameter values for the PGT concentration of Reaction 109 were obtained from the literature and summarised in Table ST.10.32.1.3.1. The log-normal distribution properties for the PGT concentration of Reaction 109 are shown in Table ST.10.32.1.3.2 and plotted in Figure SF.10.32.1.3.1. To convert the enzyme concentration from ppm to mM, **Equation S.6.2** was used.

**Table ST.10.32.1.3.1.** Literature information used to design the PGT concentration parameter distribution. Each value was assigned a weight using the protocol described in Section 2.6.2. The type of error was assigned as 0 if it was additive and 1 if it was multiplicative.

| Value (ppm) | Error (ppm) | Experimental details | | | | | | Weight | Type of error | Reference |
|---|---|---|---|---|---|---|---|---|---|---|
| | | Species | Expression Vector | Enzyme | pH | Temperature (°C) | Other | | | |
| 2.82 | NaN | Human | Gut | PGT | 7.5 | 37 | | 2048 | 0 | (Kim et al., 2014) |
| 1.74 $x10^{1}$ | NaN | Human | Oesophagus | PGT | 7.5 | 37 | | 2048 | 0 | (Wilhelm et al., 2014) |
| 1.25 $x10^{2}$ | NaN | Human | Lung | PGT | 7.5 | 37 | | 2048 | 0 | (Kim et al., 2014) |

**Table ST.10.32.1.3.2.** The log-normal distribution properties of the PGT concentration distribution. These values were calculated using the functions described in Section 2.6.2.

| Mode (ppm) | Mode (mM) | Confidence Interval | Location parameter (μ) | Scale parameter (σ) |
|---|---|---|---|---|
| 1.73 $x10^{1}$ | 9.57 $x10^{-5}$ | 4.72 | 3.90 | 1.02 |

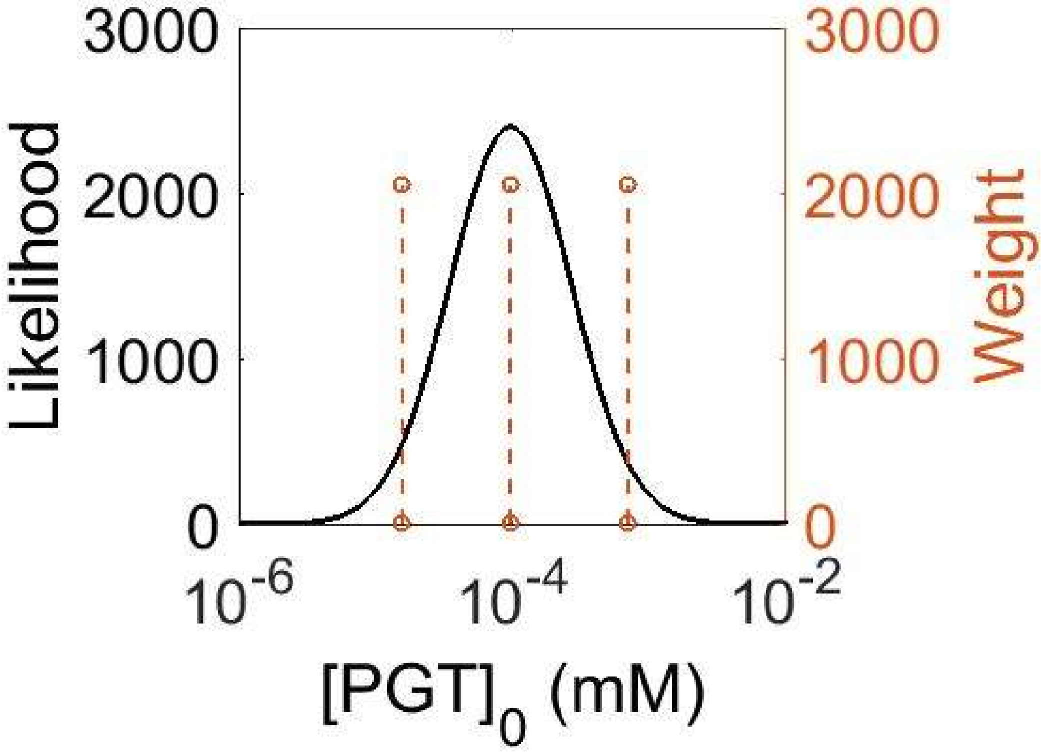


**Figure SF.10.32.1.3.1.** The estimated probability distribution for the PGT concentration. The value and weight of the literature values used to define the distribution are indicated by an orange dashed line. The x axis is plotted on a log-scale.

### S.10.33. Reaction 110: $TXA_2 \rightleftharpoons exTXA_2$ (PGT)

$TXA_2$ is relocated from the intracellular compartment to the extracellular compartment via the prostaglandin (PGT) transporter.

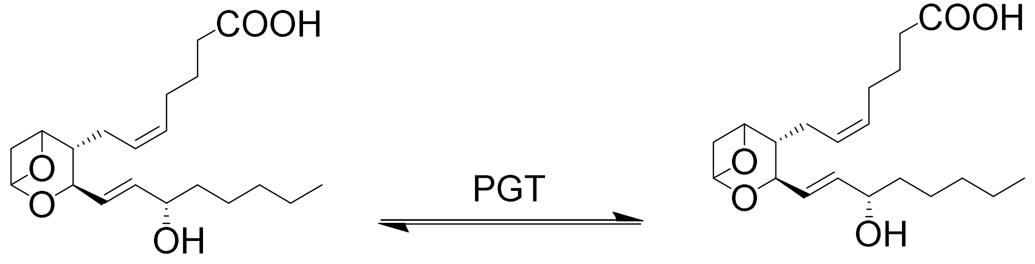


$TXA_2$ (Intracellular Compartment)

$exTXA_2$ (Extracellular Compartment)

**Figure SF.10.33.** The transport of thromboxane $A_2$ ($TXA_2$) from the intracellular compartment to the extracellular compartment by prostaglandin (PGT) transporter (Reaction 110).

**SEq.10.33.** Reaction rate law for Reaction 110.

$$v_{110} = [PGT] \cdot k_{cat} \cdot \frac{{TXA_2}/{K_m} \cdot \left(1 - \frac{exTXA_2}{TXA_2 \cdot e^{-0.5 \cdot \left(G + R \cdot T \cdot \ln\left(\frac{ATP}{ADP}\right)\right)/{R \cdot T}}}\right)}{1 + \frac{TXA_2}{K_m} + \frac{exTXA_2}{K_m} + PGT\_CI}$$

### S.10.33.1. Reaction parameters

#### S.10.33.1.1. Parameter: PGT $k_{cat}$

Parameter values for the PGT $k_{cat}$ of Reaction 110 were obtained from the literature and summarised in Table ST.10.33.1.1.1. The log-normal distribution properties for the PGT $k_{cat}$ of Reaction 110 are shown in Table ST.10.33.1.1.2 and plotted in Figure SF.10.33.1.1.1.

**Table ST.10.33.1.1.1.** Literature information used to design the PGT $k_{cat}$ parameter distribution. Each value was assigned a weight using the protocol described in Section 2.6.2. The type of error was assigned as 0 if it was additive and 1 if it was multiplicative.

| Value ($min^{-1}$) | Error ($min^{-1}$) | Experimental details | | | | | | Weight | Type of error | Reference |
|---|---|---|---|---|---|---|---|---|---|---|
| | | Species | Expression Vector | Enzyme | pH | Temperature (°C) | Other | | | |
| 3.50 | NaN | Bacteria | E. coli. | Glucose transporter | Unknown | Unknown | Bionumbers calculation | **8** | 0 | (Waygood and Steeves, 1980) |
| 3.30 $x10^{-1}$ | NaN | Bacteria | E. coli. | Lactose transporter | Unknown | Unknown | Bionumbers calculation | **8** | 0 | (Naftalin et al., 2007) |
| 8.30 $x10^{-1}$ | NaN | Bacteria | E. coli. | Lactose transporter | Unknown | Unknown | Bionumbers calculation | **8** | 0 | (Naftalin et al., 2007) |
| 3.28 | NaN | Yeast | Saccharomyces cerevisiae | HXT7 transporter | Unknown | Unknown | Bionumbers calculation | **8** | 0 | (Ye et al., 2001) |

**Table ST.10.33.1.1.2.** The log-normal distribution properties of the PGT $k_{cat}$ distribution. These values were calculated using the functions described in Section 2.6.2.

| Mode ($min^{-1}$) | Confidence Interval | Location parameter (μ) | Scale parameter (σ) |
|---|---|---|---|
| 1.33 | 7.30 | 9.20 x$10^{-1}$ | 8.0 $10^{-1}$ |

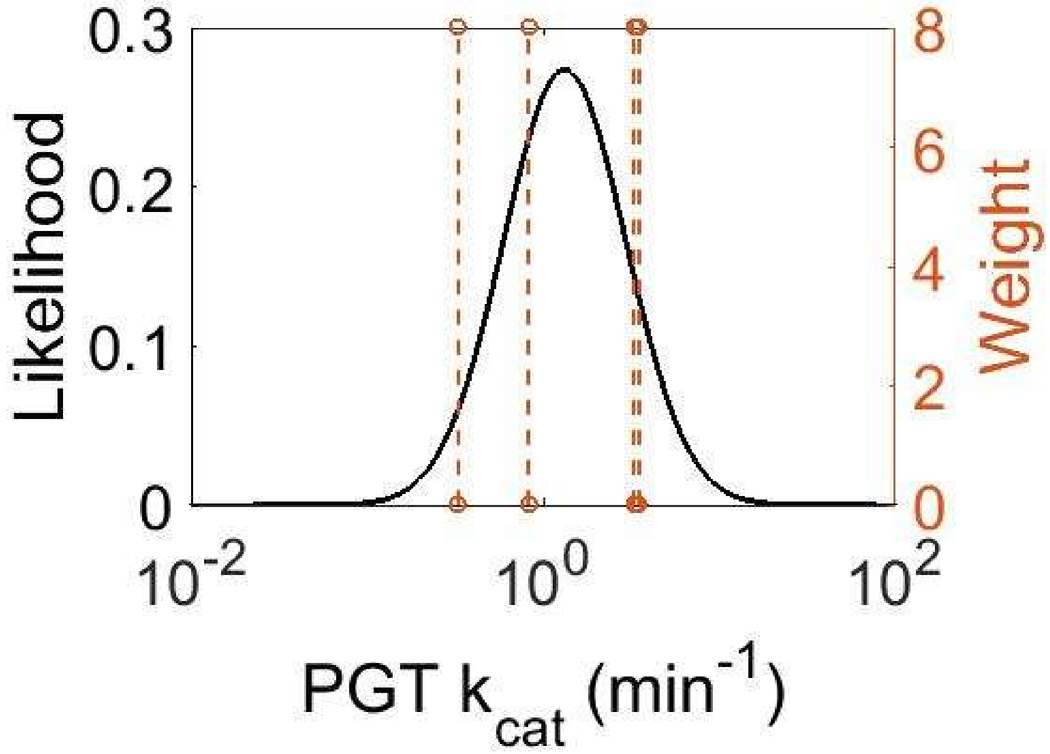


**Figure SF.10.33.1.1.1.** The estimated probability distribution for the PGT $k_{cat}$. The value and weight of the literature values used to define the distribution are indicated by an orange dashed line. The x axis is plotted on a log-scale.

**S.10.33.1.2. Parameter: PGT $K_{ms}$**

Parameter values for the PGT $K_{ms}$ of Reaction 110 were obtained from the literature and summarised in Table ST.10.33.1.2.1. The log-normal distribution properties for the PGT $K_{ms}$ of Reaction 110 are shown in Table ST.10.33.1.2.2 and plotted in Figure SF.10.33.1.2.1.

**Table ST.10.33.1.2.1.** Literature information used to design the PGT $K_{ms}$ parameter distribution. Each value was assigned a weight using the protocol described in Section 2.6.2. The type of error was assigned as 0 if it was additive and 1 if it was multiplicative.

| Value (mM) | Error (mM) | Experimental details | | | | | | Other | Weight | Type of error | Reference |
|---|---|---|---|---|---|---|---|---|---|---|---|
| | | Substrate | Species | Expression Vector | Enzyme | pH | Temperature (°C) | | | | |
| $3.31 \times 10^{-5}$ | $1.31 \times 10^{-4}$ | | Human | Eye | PGT | 7.5 | 37 | | 512 | 0 | (Waygood and Steeves, 1980) |
| $3.76 \times 10^{-4}$ | $3.40 \times 10^{-5}$ | | Human | Embryonic kidney cells (HEK293) | PGT | Unknown | 37 | | 192 | 0 | (Naftalin et al., 2007) |
| $5.40 \times 10^{-3}$ | NaN | | Human | Embryonic kidney cells (HEK293) | PGT | Unknown | 37 | | 2048 | 0 | (Naftalin et al., 2007) |
| $7.20 \times 10^{-3}$ | $5.95 \times 10^{-4}$ | | Canine | Madin-Darby Canine Kidney Epithelial Cells (MDCK) cells | PGT | Unknown | Unknown | | 512 | 0 | (Ye et al., 2001) |

| | | | | | | | | | | | |
|---|---|---|---|---|---|---|---|---|---|---|---|
| 9.40 x10$^{-5}$ | NaN | PGE2 | Human | HeLa cells | PGT | 7 | 27 | | **1024** | 0 | (Waygood and Steeves, 1980) |
| 4.23 x10$^{-4}$ | NaN | TXB2 | Human | HeLa cells | PGT | 7 | 27 | | **1024** | 0 | (Naftalin et al., 2007) |
| 7.57 x10$^{-3}$ | NaN | K6PGF1a | Human | HeLa cells | PGT | 7 | 27 | | **1024** | 0 | (Naftalin et al., 2007) |
| 4.87 x10$^{-5}$ | NaN | PGE2 | Human | HeLa cells | PGT | 7 | 27 | | **1024** | 0 | (Ye et al., 2001) |
| 5.02 x10$^{-5}$ | NaN | PGF2a | Human | HeLa cells | PGT | 7 | 27 | | **1024** | 0 | (Waygood and Steeves, 1980) |
| 5.00 x10$^{-3}$ | NaN | 15-K-PGE2 | Human | HeLa cells | PGT | 7 | 27 | | **1024** | 0 | (Naftalin et al., 2007) |
| 5.00 x10$^{-3}$ | NaN | DH-15-K-PGE2 | Human | HeLa cells | PGT | 7 | 27 | | **1024** | 0 | (Naftalin et al., 2007) |
| 3.82 x10$^{-5}$ | NaN | PGH2 | Human | HeLa cells | PGT | 7 | 27 | | **2048** | 0 | (Ye et al., 2001) |

**Table ST.10.33.1.2.2.** The log-normal distribution properties of the PGT $K_{ms}$ distribution. These values were calculated using the functions described in Section 2.6.2.

| **Mode (mM)** | **Confidence Interval** | **Location parameter (μ)** | **Scale parameter (σ)** |
|---|---|---|---|
| 1.10 x10$^{-3}$ | 9.42 x10$^{1}$ | -4.67 | 1.45 |

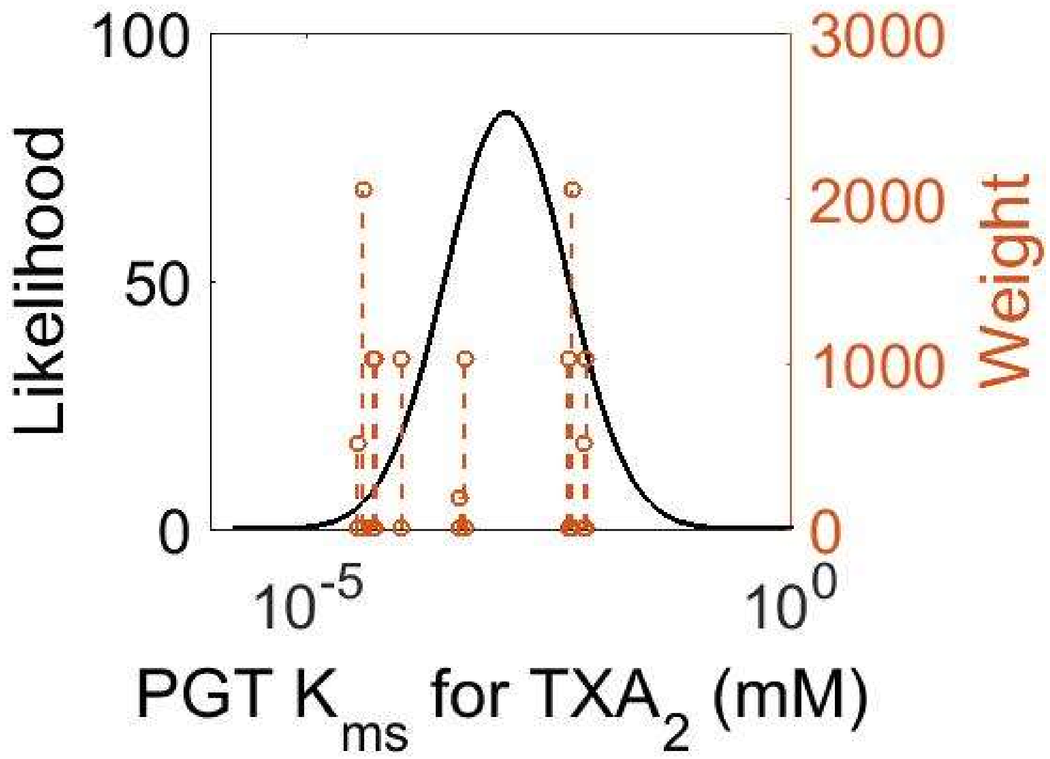


**Figure SF.10.33.1.2.1.** The estimated probability distribution for the PGT $K_{ms}$. The value and weight of the literature values used to define the distribution are indicated by an orange dashed line. The x axis is plotted on a log-scale.

### S.10.33.1.3. Parameter: PGT concentration

Parameter values for the PGT concentration of Reaction 110 were obtained from the literature and summarised in Table ST.10.33.1.3.1. The log-normal distribution properties for the PGT concentration of Reaction 110 are shown in Table ST.10.33.1.3.2 and plotted in Figure SF.10.33.1.3.1. To convert the enzyme concentration from ppm to mM, **Equation S.6.2** was used.

**Table ST.10.33.1.3.1.** Literature information used to design the PGT concentration parameter distribution. Each value was assigned a weight using the protocol described in Section 2.6.2. The type of error was assigned as 0 if it was additive and 1 if it was multiplicative.

| Value (ppm) | Error (ppm) | Experimental details | | | | | | Weight | Type of error | Reference |
|---|---|---|---|---|---|---|---|---|---|---|
| | | Species | Expression Vector | Enzyme | pH | Temperature (°C) | Other | | | |
| 2.82 | NaN | Human | Gut | PGT | 7.5 | 37 | | 2048 | 0 | (Kim et al., 2014) |
| 1.74 $x10^1$ | NaN | Human | Oesophagus | PGT | 7.5 | 37 | | 2048 | 0 | (Wilhelm et al., 2014) |
| 1.25 $x10^2$ | NaN | Human | Lung | PGT | 7.5 | 37 | | 2048 | 0 | (Kim et al., 2014) |

**Table ST.10.33.1.3.2.** The log-normal distribution properties of the PGT concentration distribution. These values were calculated using the functions described in Section 2.6.2.

| Mode (ppm) | Mode (mM) | Confidence Interval | Location parameter (μ) | Scale parameter (σ) |
|---|---|---|---|---|
| 1.73 $x10^1$ | 9.57 $x10^{-5}$ | 4.72 | 3.90 | 1.02 |

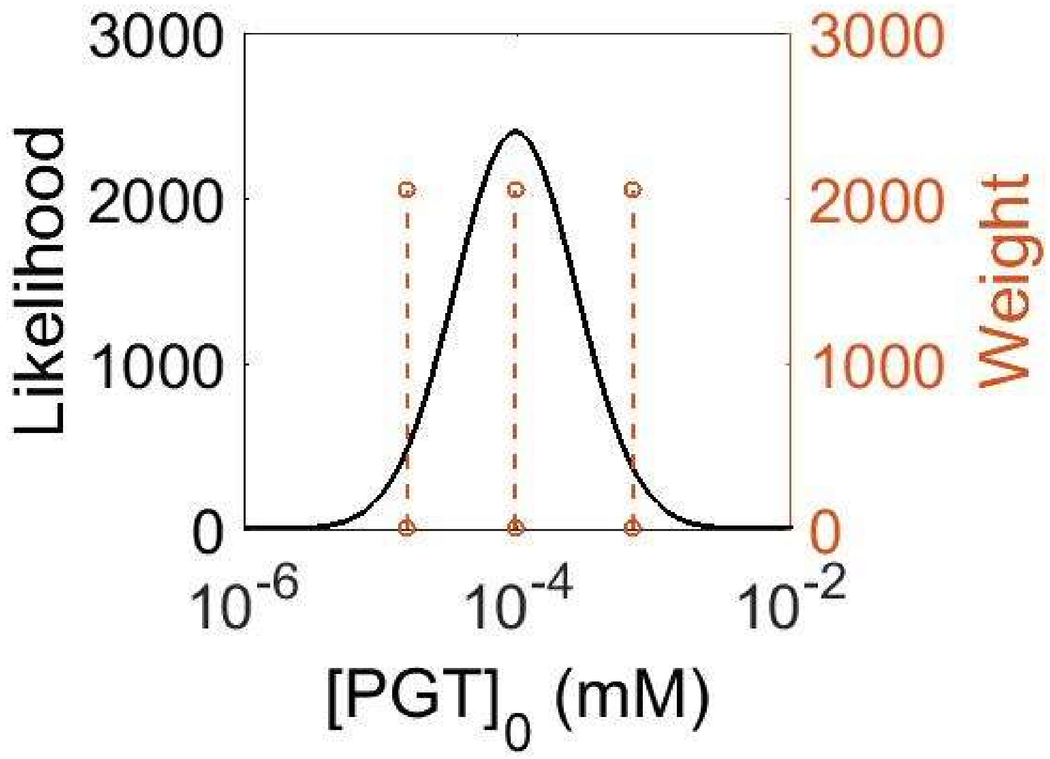


**Figure SF.10.33.1.3.1.** The estimated probability distribution for the PGT concentration. The value and weight of the literature values used to define the distribution are indicated by an orange dashed line. The x axis is plotted on a log-scale.

### S.10.34. Reaction 111: 15-deoxy-PGJ$_2$ ⇌ ex15-deoxy-PGJ$_2$ (PGT)

15-deoxy-PGJ$_2$ is relocated from the intracellular compartment to the extracellular compartment via the prostaglandin (PGT) transporter.

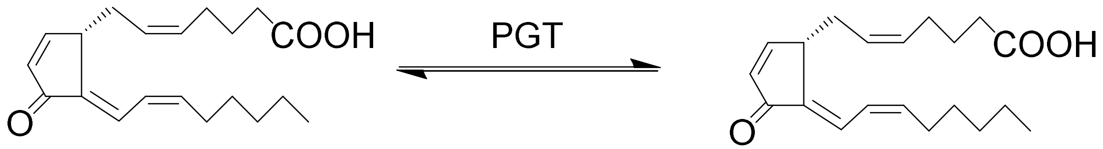


15-deoxy-PGJ$_2$
(Intracellular Compartment)

ex15-deoxy-PGJ$_2$
(Extracellular Compartment)

**Figure SF.10.34.** The transport of 15-deoxy-prostaglandin J$_2$ (15-deoxy-PGJ$_2$) from the intracellular compartment to the extracellular compartment by prostaglandin (PGT) transporter (Reaction 111).

**SEq.10.34.** Reaction rate law for Reaction 111.

$$\boldsymbol{v_{111}} = [\boldsymbol{PGT}] \cdot \boldsymbol{k_{cat}} \cdot \frac{\mathbf{15{-}deoxy{-}PGJ_2}/K_m \cdot \left(1 - \frac{\mathbf{ex15{-}deoxy{-}PGJ_2}}{\mathbf{15{-}deoxy{-}PGJ_2} \cdot e^{-0.5 \cdot \left(G + R \cdot T \cdot \ln\left(\frac{ATP}{ADP}\right)\right) / R \cdot T}}\right)}{1 + \frac{\mathbf{15{-}deoxy{-}PGJ_2}}{K_m} + \frac{\mathbf{ex15{-}deoxy{-}PGJ_2}}{K_m} + \mathbf{PGT\_CI}}$$

## S.10.34.1. Reaction parameters

### S.10.34.1.1. Parameter: PGT $k_{cat}$

Parameter values for the PGT $k_{cat}$ of Reaction 111 were obtained from the literature and summarised in Table ST.10.34.1.1.1. The log-normal distribution properties for the PGT $k_{cat}$ of Reaction 111 are shown in Table ST.10.34.1.1.2 and plotted in Figure SF.10.34.1.1.1.

**Table ST.10.34.1.1.1.** Literature information used to design the PGT $k_{cat}$ parameter distribution. Each value was assigned a weight using the protocol described in Section 2.6.2. The type of error was assigned as 0 if it was additive and 1 if it was multiplicative.

| Value ($min^{-1}$) | Error ($min^{-1}$) | Experimental details | | | | | | Weight | Type of error | Reference |
|---|---|---|---|---|---|---|---|---|---|---|
| | | **Species** | **Expression Vector** | **Enzyme** | **pH** | **Temperature (°C)** | **Other** | | | |
| 3.50 | NaN | Bacteria | E. coli. | Glucose transporter | Unknown | Unknown | Bionumbers calculation | **8** | 0 | (Waygood and Steeves, 1980) |
| 3.30 $x10^{-1}$ | NaN | Bacteria | E. coli. | Lactose transporter | Unknown | Unknown | Bionumbers calculation | **8** | 0 | (Naftalin et al., 2007) |
| 8.30 $x10^{-1}$ | NaN | Bacteria | E. coli. | Lactose transporter | Unknown | Unknown | Bionumbers calculation | **8** | 0 | (Naftalin et al., 2007) |
| 3.28 | NaN | Yeast | Saccharomyces cerevisiae | HXT7 transporter | Unknown | Unknown | Bionumbers calculation | **8** | 0 | (Ye et al., 2001) |

**Table ST.10.34.1.1.2.** The log-normal distribution properties of the PGT $k_{cat}$ distribution. These values were calculated using the functions described in Section 2.6.2.

| **Mode ($min^{-1}$)** | **Confidence Interval** | **Location parameter ($\mu$)** | **Scale parameter ($\sigma$)** |
|---|---|---|---|
| 1.33 | 7.30 | $9.20 \times 10^{-1}$ | $8.0 \times 10^{-1}$ |

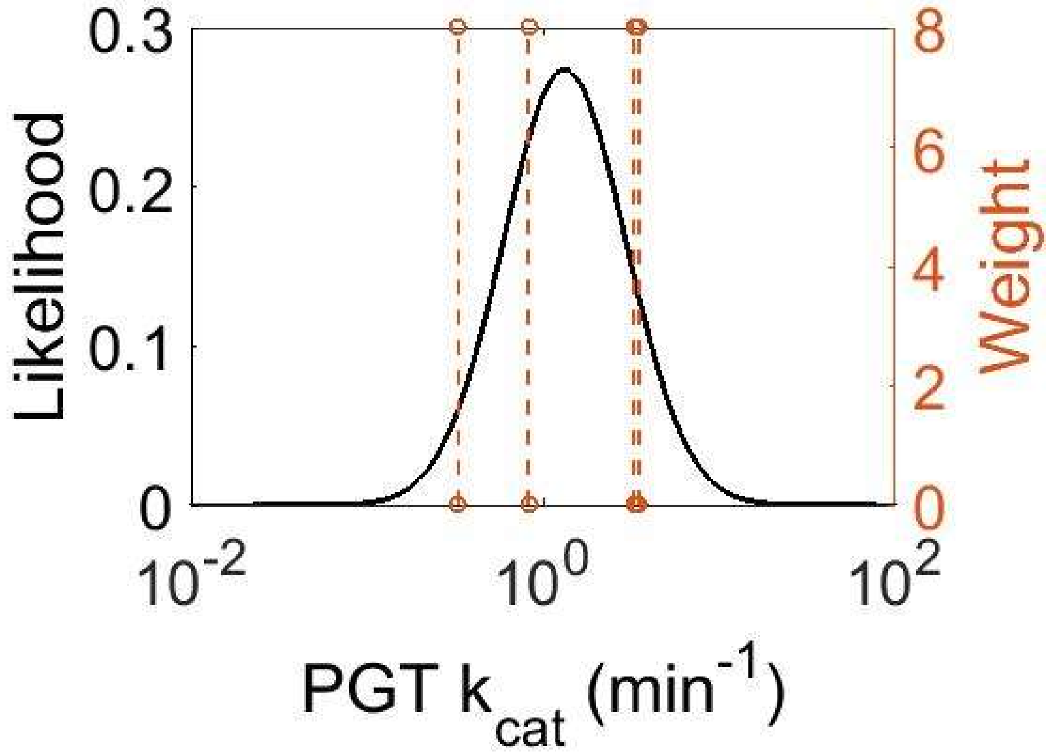


**Figure SF.10.34.1.1.1.** The estimated probability distribution for the PGT $k_{cat}$. The value and weight of the literature values used to define the distribution are indicated by an orange dashed line. The x axis is plotted on a log-scale.

### S.10.34.1.2. Parameter: PGT $K_{ms}$

Parameter values for the PGT $K_{ms}$ of Reaction 111 were obtained from the literature and summarised in Table ST.10.34.1.2.1. The log-normal distribution properties for the PGT $K_{ms}$ of Reaction 111 are shown in Table ST.10.34.1.2.2 and plotted in Figure SF.10.34.1.2.1.

**Table ST.10.34.1.2.1.** Literature information used to design the PGT $K_{ms}$ parameter distribution. Each value was assigned a weight using the protocol described in Section 2.6.2. The type of error was assigned as 0 if it was additive and 1 if it was multiplicative.

| Value (mM) | Error (mM) | Experimental details | | | | | | Other | Weight | Type of error | Reference |
|---|---|---|---|---|---|---|---|---|---|---|---|
| | | Substrate | Species | Expression Vector | Enzyme | pH | Temperature (°C) | | | | |
| $3.31 \times 10^{-5}$ | $1.31 \times 10^{-4}$ | | Human | Eye | PGT | 7.5 | 37 | | 512 | 0 | (Waygood and Steeves, 1980) |
| $3.76 \times 10^{-4}$ | $3.40 \times 10^{-5}$ | | Human | Embryonic kidney cells (HEK293) | PGT | Unknown | 37 | | 192 | 0 | (Naftalin et al., 2007) |
| $5.40 \times 10^{-3}$ | NaN | | Human | Embryonic kidney cells (HEK293) | PGT | Unknown | 37 | | 2048 | 0 | (Naftalin et al., 2007) |
| $7.20 \times 10^{-3}$ | $5.95 \times 10^{-4}$ | | Canine | Madin-Darby Canine Kidney Epithelial Cells (MDCK) cells | PGT | Unknown | Unknown | | 512 | 0 | (Ye et al., 2001) |

| | | | | | | | | | | | |
|---|---|---|---|---|---|---|---|---|---|---|---|
| 9.40 x$10^{-5}$ | NaN | PGE2 | Human | HeLa cells | PGT | 7 | 27 | | **1024** | 0 | (Waygood and Steeves, 1980) |
| 4.23 x$10^{-4}$ | NaN | TXB2 | Human | HeLa cells | PGT | 7 | 27 | | **1024** | 0 | (Naftalin et al., 2007) |
| 7.57 x$10^{-3}$ | NaN | K6PGF1a | Human | HeLa cells | PGT | 7 | 27 | | **1024** | 0 | (Naftalin et al., 2007) |
| 4.87 x$10^{-5}$ | NaN | PGE2 | Human | HeLa cells | PGT | 7 | 27 | | **1024** | 0 | (Ye et al., 2001) |
| 5.02 x$10^{-5}$ | NaN | PGF2a | Human | HeLa cells | PGT | 7 | 27 | | **1024** | 0 | (Waygood and Steeves, 1980) |
| 5.00 x$10^{-3}$ | NaN | 15-K-PGE2 | Human | HeLa cells | PGT | 7 | 27 | | **1024** | 0 | (Naftalin et al., 2007) |
| 5.00 x$10^{-3}$ | NaN | DH-15-K-PGE2 | Human | HeLa cells | PGT | 7 | 27 | | **1024** | 0 | (Naftalin et al., 2007) |
| 3.82 x$10^{-5}$ | NaN | PGH2 | Human | HeLa cells | PGT | 7 | 27 | | **2048** | 0 | (Ye et al., 2001) |

**Table ST.10.34.1.2.2.** The log-normal distribution properties of the PGT $K_{ms}$ distribution. These values were calculated using the functions described in Section 2.6.2.

| **Mode (mM)** | **Confidence Interval** | **Location parameter (μ)** | **Scale parameter (σ)** |
|---|---|---|---|
| 1.10 x$10^{-3}$ | 9.42 x$10^{1}$ | -4.67 | 1.45 |

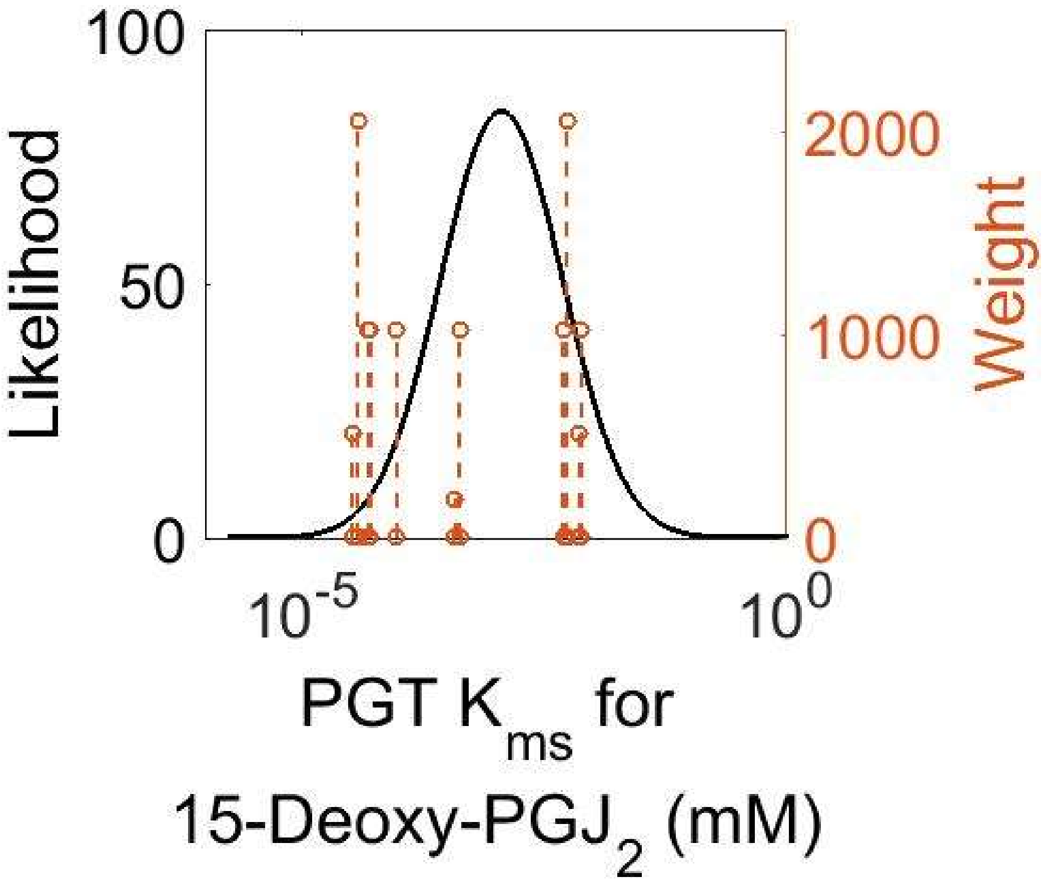


**Figure SF.10.34.1.2.1.** The estimated probability distribution for the PGT $K_{ms}$. The value and weight of the literature values used to define the distribution are indicated by an orange dashed line. The x axis is plotted on a log-scale.

**S.10.34.1.3. Parameter: PGT concentration**

Parameter values for the PGT concentration of Reaction 111 were obtained from the literature and summarised in Table ST.10.34.1.3.1. The log-normal distribution properties for the PGT concentration of Reaction 111 are shown in Table ST.10.34.1.3.2 and plotted in Figure SF.10.34.1.3.1. To convert the enzyme concentration from ppm to mM, **Equation S.6.2** was used.

**Table ST.10.34.1.3.1.** Literature information used to design the PGT concentration parameter distribution. Each value was assigned a weight using the protocol described in Section 2.6.2. The type of error was assigned as 0 if it was additive and 1 if it was multiplicative.

| Value (ppm) | Error (ppm) | Experimental details | | | | | | Weight | Type of error | Reference |
|---|---|---|---|---|---|---|---|---|---|---|
| | | Species | Expression Vector | Enzyme | pH | Temperature (°C) | Other | | | |
| 2.82 | NaN | Human | Gut | PGT | 7.5 | 37 | | 2048 | 0 | (Kim et al., 2014) |
| $1.74 \times 10^{1}$ | NaN | Human | Oesophagus | PGT | 7.5 | 37 | | 2048 | 0 | (Wilhelm et al., 2014) |
| $1.25 \times 10^{2}$ | NaN | Human | Lung | PGT | 7.5 | 37 | | 2048 | 0 | (Kim et al., 2014) |

**Table SF.10.34.1.3.1.** The log-normal distribution properties of the PGT concentration distribution. These values were calculated using the functions described in Section 2.6.2.

| Mode (ppm) | Mode (mM) | Confidence Interval | Location parameter (μ) | Scale parameter (σ) |
|---|---|---|---|---|
| $1.73 \times 10^{1}$ | $9.57 \times 10^{-5}$ | 4.72 | 3.90 | 1.02 |

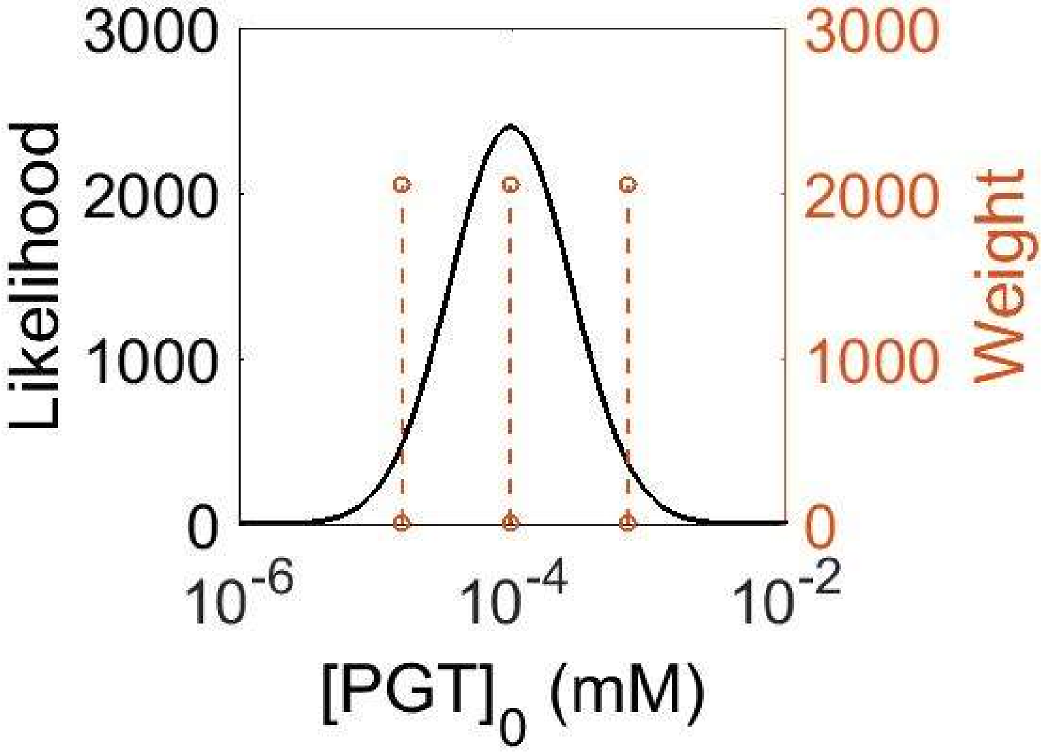


**Figure SF.10.34.1.3.1.** The estimated probability distribution for the PGT concentration. The value and weight of the literature values used to define the distribution are indicated by an orange dashed line. The x axis is plotted on a log-scale.

References


Chi, Y. & Schuster, V. L. (2010). 'The prostaglandin transporter pgt transports pgh2' *Biochem Bioph Res Co*, 395 (2), pp. 168-172. DOI: https://doi.org/10.1016/j.bbrc.2010.03.108 Available at: http://www.sciencedirect.com/science/article/pii/S0006291X10005723.

Fong, W. F., et al. (2007). 'Schisandrol a from schisandra chinensis reverses p-glycoprotein-mediated multidrug resistance by affecting pgp-substrate complexes' *Planta Med*, 73 (3), pp. 212-20. DOI: 10.1055/s-2007-967120.

Gose, T., et al. (2016). 'Prostaglandin transporter (oatp2a1/slco2a1) contributes to local disposition of eicosapentaenoic acid-derived pge3' *Prostaglandins Other Lipid Mediat*, 122 pp. 10-7. DOI: 10.1016/j.prostaglandins.2015.12.003.

Itoh, S., et al. (1996). 'Structural determinants of substrates for the prostaglandin transporter pgt' *Mol Pharmacol*, 50 (4), pp. 736-742. DOI: 10.1124/mol.50.4.736 Available at: http://molpharm.aspetjournals.org/content/molpharm/50/4/736.full.pdf.

Kanai, N., et al. (1995). 'Identification and characterization of a prostaglandin transporter' *Science*, 268 (5212), pp. 866-869. DOI: 10.1126/science.7754369 Available at: http://science.sciencemag.org/content/sci/268/5212/866.full.pdf.

Kim, M.-S., et al. (2014). 'A draft map of the human proteome' *Nature*, 509 p. 575. DOI: 10.1038/nature13302

https://www.nature.com/articles/nature13302#supplementary-information Available at: https://doi.org/10.1038/nature13302.

Kraft, M. E., et al. (2010). 'The prostaglandin transporter oatp2a1 is expressed in human ocular tissues and transports the antiglaucoma prostanoid latanoprost' *Invest Ophthalmol Vis Sci*, 51 (5), pp. 2504-11. DOI: 10.1167/iovs.09-4290.

Mao, Q., Deeley, R. G. & Cole, S. P. (2000). 'Functional reconstitution of substrate transport by purified multidrug resistance protein mrp1 (abcc1) in phospholipid vesicles' *J Biol Chem*, 275 (44), pp. 34166-72. DOI: 10.1074/jbc.M004584200.

Naftalin, R. J., Green, N. & Cunningham, P. (2007). 'Lactose permease h+-lactose symporter: Mechanical switch or brownian ratchet?' *Biophys J*, 92 (10), pp. 3474-91. DOI: 10.1529/biophysj.106.100669.

Waygood, E. B. & Steeves, T. (1980). 'Enzyme i of the phosphoenolpyruvate: Sugar phosphotransferase system of escherichia coli. Purification to homogeneity and some properties' *Can J Biochem*, 58 (1), pp. 40-8.

Wilhelm, M., et al. (2014). 'Mass-spectrometry-based draft of the human proteome' *Nature*, 509 p. 582. DOI: 10.1038/nature13319

https://www.nature.com/articles/nature13319#supplementary-information Available at: https://doi.org/10.1038/nature13319.

Yasunaga, M., et al. (2008). 'Molecular cloning and functional characterization of cynomolgus monkey multidrug resistance-associated protein 2 (mrp2)' *Eur J Pharm Sci*, 35 (4), pp. 326-334. DOI: https://doi.org/10.1016/j.ejps.2008.08.001 Available at: http://www.sciencedirect.com/science/article/pii/S0928098708003618.

Ye, L., Berden, J. A., van Dam, K. & Kruckeberg, A. L. (2001). 'Expression and activity of the hxt7 high-affinity hexose transporter of saccharomyces cerevisiae' *Yeast*, 18 (13), pp. 1257-67. DOI: 10.1002/yea.771.

Zeng, H., Liu, G., Rea, P. A. & Kruh, G. D. (2000). 'Transport of amphipathic anions by human multidrug resistance protein 3' *Cancer Res*, 60 (17), pp. 4779-84.

**Supplementary Document S11. Initiation Reaction Structure and Parameterisation.**

Documentation of parameter values obtained for all transporter-mediated reactions in the model (Reactions 95, 112, 113; Supplementary Table S1) from the literature and associated uncertainty for the eicosanoid network model. Parameterisation was performed using the method of Tsigkinopoulou *et al.,* (2018). The table includes information regarding each reaction and its respective parameters are documented. This includes information such as the reaction rate law and the literature values that were used to define parameters, including experimental conditions, total weights and literature references from which the data were obtained. In this model some parameters are referred as "Dependent parameters", meaning that the log-normal distribution for that parameter was calculated using multivariate distributions (discussed in **Section 2.6.2**). As a result, no confidence interval factor or literature values were cited for the Dependent parameters.

**Contents**

S.11.1. Reaction 95: $\phi \rightarrow$ AA ........ 765

S.11.2. Reaction 112: $\phi \rightarrow$ COX-2 ........ 768

S.11.3. Reaction 113: MAA $\rightarrow$ AA ........ 770

**S.11.1. Reaction 95: ϕ → AA**

The liberation of free AA from the phospholipid membrane. As this reaction is performed by a multitude of isoforms of PLA2, all with unique kinetics, this reaction was simplified.

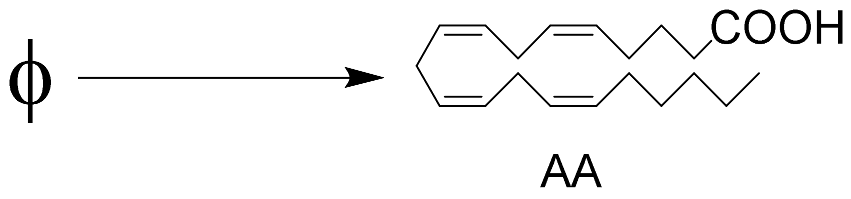


**Figure SF.11.1.** The release of arachidonic acid into the computational model (Reaction 95).

**S.Eq.11.1.** Reaction rate law for Reaction 95.

$$\boldsymbol{v_{95} = \frac{c}{AADT} - \frac{c2 \cdot \ln(2) \cdot [AA]}{AAHL}}$$

### S.11.1.1. Reaction parameters

**Table ST.11.1.1.1.** The name, shorthand, initial value, adapted value and justification for each event parameter. These parameters are adapted to represent different model scenarios.

| Parameter Name | Parameter Shorthand | Initial Value | Justification |
|---|---|---|---|
| Doubling time of the AA event | AADT | 420 min | AA was set to be released for 6h, as this was the length of the inflammatory window in the modelled system. This value was not changed in the modelled scenarios. |
| Maximum concentration of AA | c | 0 mM | The adapted value of this parameter depends on the scenario (Table S13). This value changes 60 min into the simulation for all scenarios. |
| Decay switch | c2 | 0 | If required the production of AA can decrease over time by amending this value from 0 to 1. This was set to 0 in all scenarios. |
| Half-life of the AA event | AAHL | 420 min | This value was arbitrarily set at 420 min. However, no simulations required AA release to decrease over time. |

**Table ST.11.1.1.2.** Values of the Maximum AA parameter in the eight model scenarios.

| | **Scenario** | | | | | | | |
|---|---|---|---|---|---|---|---|---|
| **Treatment** | **A23187** | | **Indomethacin and A23187** | | **ATP** | | **UVR** | |
| **Cell type** | **HaCaT keratinocyte** | **46BR.1N fibroblast** | **HaCaT keratinocyte** | **46BR.1N fibroblast** | **HaCaT keratinocyte** | **46BR.1N fibroblast** | **HaCaT keratinocyte** | **46BR.1N fibroblast** |
| **Σ AA released (pg/million cells)** | 2.03 $x10^{6}$ | 5.43 $x10^{5}$ | 1.64 $x10^{6}$ | 2.11 $x10^{5}$ | 8.43 $x10^{5}$ | 3.29 $x10^{4}$ | 1.80 $x10^{6}$ | 4.30 $x10^{5}$ |
| **Σ AA released (mM)** | 5.77 $x10^{-4}$ | 1.54 $x10^{-4}$ | 4.65 $x10^{-4}$ | 6.00 $x10^{-5}$ | 2.40 $x10^{-4}$ | 9.36 $x10^{-6}$ | 5.11 $x10^{-4}$ | 1.22E $x10^{-4}$ |

**S.11.2. Reaction 112: ϕ → COX-2**
The induced expression of COX-2. As this process is as a result of complex transcription and translation processes, this reaction was simplified.

$$\phi \rightarrow \text{COX-2}$$

**Figure SF.11.2.** The induction of COX-2 in the computational model (Reaction 112).

**SEq.11.2.** Reaction rate law for Reaction 112.
$$\boldsymbol{v_{112}} = \frac{c3}{COX2DT} - \frac{c4 \cdot \ln(2) \cdot [COX-2]}{COX2HL}$$

### S.11.2.1. Reaction parameters

### S.11.2.1.1. Fixed Parameters

**Table ST.11.2.1.1.1.** The name, shorthand, initial value, adapted value and justification for the COX-2 event parameter. These parameters are adapted to represent different model scenarios.

| Parameter Name | Parameter Shorthand | Initial Value | Justification |
|---|---|---|---|
| Doubling time of the COX-2 event | COX2DT | 180 min | This value was upon the unpublished western blot data in (Kiezel-Tsugunova, 2017). |
| Maximum concentration of COX-2 | C3 | 0 mM | The adapted value of this parameter depends on the scenario (Table S15). This value changes 240 min into the simulation for the UVR scenarios. |
| Decay switch | C4 | 0 | If required the induced expression of COX-2 can decrease over time by amending this value from 0 to 1. This was set to 0 in all scenarios. |
| Half-life of the COX-2 event | COX2HL | 0 min | This value was arbitrarily set at 0 min. However, no simulations required COX-2 release to decrease over time. |

**Table ST.11.2.1.1.2.** Values of the COX-2 concentration in the eight model scenarios.

| | Scenario | | | | | | | |
|---|---|---|---|---|---|---|---|---|
| **Treatment** | **A23187** | | **Indomethacin and A23187** | | **ATP** | | **UVR** | |
| **Cell type** | **HaCaT keratinocyte** | **46BR.1N fibroblast** | **HaCaT keratinocyte** | **46BR.1N fibroblast** | **HaCaT keratinocyte** | **46BR.1N fibroblast** | **HaCaT keratinocyte** | **46BR.1N fibroblast** |
| **COX-1 (mM) at 0h** | x | x | 1.00 $x10^{-28}$ | 1.00 $x10^{-28}$ | x | x | x | x |
| **COX-2 (mM) at 0h** | x | x | 1.00 $x10^{-28}$ | 1.00 $x10^{-28}$ | x | x | 1.00 $x10^{-28}$ | 1.00 $x10^{-28}$ |
| **COX-2 (mM) at 6h** | x | x | x | x | x | x | 2.27 $x10^{-2}$ | $2.2710^{-2}$ |

**S.11.3. Reaction 113: MAA → AA**

In the attempt to improve the AA release mechanism, reaction 113 was added. However, this reaction was not incorporated into all model scenarios as it required further refinement.

$$\text{MAA} \rightarrow \text{AA}$$

**Figure SF.11.2.** The alternative release mechanism of arachidonic acid into the computational model (Reaction 113).

**SEq.11.3.** Reaction rate law for Reaction 113.

$$\boldsymbol{v_{113}} = \boldsymbol{R113K} \times [\boldsymbol{MAA}]$$

**Supplementary Table S12. Ordinary differential equations for each metabolite in the model.** Where "r" is the reaction number assigned in Supplementary Tables S2-6. Arachidonic acid (AA), Prostaglandin $H_2$ ($PGH_2$), Prostaglandin $F_{2\alpha}$ ($PGF_{2\alpha}$), Thromboxane $A_2$ ($TXA_2$), Prostaglandin $I_2$ ($PGI_2$), Thromboxane $B_2$ ($TXB_2$), 6-Keto-prostaglandin $F_{1\alpha}$ (6-keto-$PGF_{1\alpha}$), Prostaglandin $J_2$ ($PGJ_2$), Prostaglandin $D_2$ ($PGD_2$), 15-Deoxy-prostaglandin $J_2$ (15-deoxy-$PGJ_2$), Prostaglandin $E_2$ ($PGE_2$), 5-hydroperoxy-eicosatetraenoic acid (5-HPETE), 5-Hydroxy-eicosatetraenoic acid (5-HETE), Leukotriene $A_4$ ($LTA_4$), 5-Oxo-eicosatetraenoic acid (5-oxo-ETE), Leukotriene $B_4$ ($LTB_4$), 15-hydroperoxy-eicosatetraenoic acid (15-HPETE), 15-Hydroxy-eicosatetraenoic acid (15-HETE), 12-hydroperoxy-eicosatetraenoic acid (12-HPETE), 12-Hydroxy-eicosatetraenoic acid (12-HETE), Extracellular prostaglandin $F_{2\alpha}$ (ex$PGF_{2\alpha}$), Extracellular thromboxane $B_2$ (ex$TXB_2$), Extracellular 6-keto prostaglandin $F_{1\alpha}$ (ex6-keto-$PGF_{1\alpha}$), Extracellular prostaglandin $E_2$ (ex$PGE_2$), Extracellular 15-deoxy-prostaglandin $J_2$ (ex15-deoxy-$PGJ_2$), Extracellular 5-oxo- eicosatetraenoic acid (ex5-oxo-ETE), Extracellular 15 -hydroxy-eicosatetraenoic acid (ex15-HETE), Extracellular leukotriene $B_4$ (ex$LTB_4$), Leukotriene $C_4$ ($LTC_4$), Extracellular leukotriene $C_4$ (ex$LTC_4$), 1 Extracellular 12-hydroxy-eicosatetraenoic acid (ex12-HETE), Extracellular thromboxane $A_2$ (ex$TXA_2$), Extracellular prostaglandin $I_2$ (ex$PGI_2$), Extracellular prostaglandin $H_2$ (ex$PGH_2$), Extracellular prostaglandin $D_2$ (ex$PGD_2$), Extracellular prostaglandin $J_2$ (ex$PGJ_2$), Extracellular 12-hydroperoxy-eicosatetraenoic acid (ex12-HPETE), Extracellular 15-hydroperoxy-eicosatetraenoic acid (ex15-HPETE), Extracellular 5-hydroperoxy-eicosatetraenoic acid (ex5-HPETE), Extracellular 5-hydroxy-eicosatetraenoic acid (ex5-HETE), Extracellular leukotriene $A_4$ (ex$LTA_4$), Extracellular arachidonic acid (exAA), 15-keto-prostaglandin $E_2$ (15-keto-$PGE_2$), Extracellular 15-keto-prostaglandin $E_2$ (ex15-keto-$PGE_2$), 13,14-Dihydro-15-keto-prostaglandin $E_2$ (13,14-dihydro-15-keto-$PGE_2$), Extracellular 13,14-dihydro-15-keto-prostaglandin $E_2$ (ex13,14-dihydro-15-keto-$PGE_2$), Cyclooxygenase 2 (COX-2).

| **Ordinary differential equations** |
|---|
| d[AA]/dt=r95+r1-r2-r11-r17-r19-r42-r65-r92-r100+r113 |
| d[$PGH_2$]/dt=r2+r65-r3-r4-r5-r10-r21-r81-r34 |
| d[$PGF_{2\alpha}$]/dt=r3-r22-r72-r101 |

| |
|---|
| $d[TXA_2]/dt=r4-r6-r32-r74-r110$ |
| $d[PGI_2]/dt=r5-r7-r33-r76-r103$ |
| $d[TXB_2]/dt=r6-r106-r73-r23$ |
| $d[6\text{-keto-}PGF_{1\alpha}]/dt=r7-r24-r75-r109$ |
| $d[PGJ_2]/dt=r8-r9-r36-r79-r105$ |
| $d[PGD_2]/dt=r21-r8-r35-r80-r104$ |
| $d[15\text{-deoxy-}PGJ_2]/dt=r9-r26-r78-r111$ |
| $d[PGE_2]/dt=r10-r25-r66-r77-r102$ |
| d[5-HPETE]/dt=r11-r12-r13-r39-r87 |
| d[5-HETE]/dt=r12-r14-r40-r83 |
| $d[LTA_4]/dt=r13-r15-r16-r41-r86$ |
| d[5-oxo-ETE]/dt=r14-r27-r82 |
| $d[LTB_4]/dt=r15-r29-r84$ |
| $d[LTC_4]/dt=r16-r30-r85$ |
| d[15-HPETE]/dt=r17-r18-r38-r89 |
| d[15-HETE]/dt=r18-r28-r88 |
| d[12-HPETE]/dt=r19-r20-r37-r91 |
| d[12-HETE]/dt=r20-r31-r90 |
| $d[exPGF_{2\alpha}]/dt=r22+r101-r44$ |
| $d[exTXB_2]/dt=r23+r106-r45+r96$ |
| $d[ex6\text{-keto-}PGF_{1\alpha}]/dt=r24+r109-r47+r97$ |
| $d[exPGE_2]/dt=r25+r102-r49$ |
| $d[ex15\text{-deoxy-}PGJ_2]/dt=r26+r111-r50+r99$ |
| d[ex5-oxo-ETE]/dt=r27-r54 |
| d[ex15-HETE]/dt=r28-r60 |
| $d[exLTB_4]/dt=r29-r56$ |
| $d[exLTC_4]/dt=r30-r57$ |
| d[ex12-HETE]/dt=r31-r62 |
| $d[exTXA_2]/dt=r32+r110-r46-r96$ |
| $d[exPGI_2]/dt=r33+r103-r48-r97$ |
| $d[exPGH_2]/dt=-r53$ |
| $d[exPGD_2]/dt=r35+r104-r52-r98$ |
| $d[exPGJ_2]/dt=r36+r105-r51+r98-r99$ |
| d[ex12-HPETE]/dt=r37-r63 |
| d[ex15-HPETE]/dt=r38-r61 |
| d[ex5-HPETE]/dt=r39-r59 |
| d[ex5-HETE]/dt=r40-r55 |
| $d[exLTA_4]/dt=r41-r58$ |
| d[exAA]/dt=-r64 |
| $d[15\text{-keto-}PGE_2]/dt=r66-r67-r69-r94-r108$ |
| $d[ex15\text{-keto-}PGE_2]/dt=r67+r108-r68$ |
| $d[13,14\text{-dihydro-}15\text{-keto-}PGE_2]/dt=r69-r70-r93-r107$ |

| |
|---|
| d[ex13,14-dihydro-15-keto-$PGE_2$]/dt=r70+r107-r71 |
| d[COX-2]/dt=r112-r2 |
| d[Miscellaneous metabolites]/dt=r44+r45+r46+r47+r48+r49+r50+r51+r52+r53+r54 +r55+r56+r57+r58+r59+r60+r61+r62+r63+r64+r68+r71+r72+r73+r74+r75+r76+r77 +r78+r79+r80+r81+r82+r83+r84+r85+r86+r87+r88+r89+r90+r91+r92 |

**Supplementary Table S13. Parameter changes used to model cell-specific AA release.** Depending on the *in silico* experiment, the value of parameters in equation 2 change at set time points in order to recreate experimental observations. The data used for the "maximum 6h concentration of AA" parameter was the approximate difference of AA measured by fatty acid analysis after 6h of stimulation (mean of three independent experiments). Simulated treatments were A23187 (6 h, 5 µM), indomethacin (IND) (1 h, 10 µM) followed by A23187 (6 h, 5 µM), ATP (2 mM), ultraviolet radiation (15 mJ/cm$^2$). * HaCaT + UVR utilised a minimal initial AA concentration and delayed AA release, so the "$Maximum\ 6h\ Concentration\ of\ AA$" was set as 1.80 ×10$^5$ pg/10$^6$ cells for 0-3 h post-stimulation (10 % of the experimental maximum AA concentration), followed by 1.65 ×10$^6$ pg/10$^6$ cells for 3-6 h post-stimulation (90 % of the experimental maximum AA concentration). This represented the delayed AA cascade response to UVR stimulation.

| | | Parameters | | | | | |
|---|---|---|---|---|---|---|---|
| | ***In Silico* Experiment** | "$Maximum\ 6h\ Concentration\ of\ AA$" | | | "$Doubling\ Time\ of\ AA$" (h) | "$Decay\ Switch$" | "$Half\ Life\ of\ AA$" (h) |
| | | Value (pg/10$^6$ cells) | Simulation time (h) | Duration (h) | | | |
| **HaCaT keratinocytes** | HaCaT + A23187 | 2.03 ×10$^6$ | 0 | 6 | 6 | Off | 6 |
| | HaCaT + IND + A23187 | 1.64 ×10$^6$ | 0 | 6 | 6 | Off | 6 |
| | HaCaT + ATP | 8.43 ×10$^5$ | 0 | 6 | 6 | Off | 6 |
| | HaCaT + UVR (*) | 1.80 ×10$^5$<br>1.65 ×10$^6$ | 3 | 3 | 3 | Off | 6 |
| **46BR.1N** | 46BR.1N + A23187 | 5.43 ×10$^5$ | 0 | 6 | 6 | Off | 6 |
| | 46BR.1N + IND + A23187 | 2.11 ×10$^5$ | 0 | 6 | 6 | Off | 6 |
| | 46BR.1N + ATP | 3.29 ×10$^4$ | 0 | 6 | 6 | Off | 6 |

| | | | | | | | |
|---|---|---|---|---|---|---|---|
| | 46BR.1N + UVR | $4.30 \times 10^{5}$ | 0 | 6 | 6 | Off | 6 |

**Supplementary Table S14. Examples of putative patterns of AA release: single release, decaying release, constant release, and delayed release (Figure 7B).** The value of parameters in Equation 2 were set to change at set time points in order to recreate the relevant experimental observations.

| | **Parameters** | | | | |
|---|---|---|---|---|---|
| **Simulation type** | $Maximum\ Concentration\ of\ AA$ | | $Doubling\ Time\ of\ AA$ (min) | $Decay\ Switch$ | $Half\ Life\ of\ AA$ (min) |
| | Time (min) | Duration (min) | | | |
| Single release | 0 | 1 | 1 | Off | 360 |
| Constant release | 0 | 360 | 360 | Off | 360 |
| Decaying release | 0 | 1 | 1 | On | 360 |
| Delayed release | 180 | 180 | 180 | Off | 360 |

**Supplementary Table S15. Parameter changes used to model the *in silico* COX-2 induction.** Parameter changes are described for the "HaCaT + UVR" and "46BR.1N + UVR" *in silico* experiments. The $"Maximum\ 6h\ Concentration\ of\ COX-2"$ increased from its initial value to its final value, starting at the simulation time and occuring across the duration specified. These changes were only made for UVR simulations and represented the delayed AA cascade response to UVR stimulation.

| | Parameters | | | | | | |
|---|---|---|---|---|---|---|---|
| | $"Maximum\ 6h\ Concentration\ of\ COX-2"$ | | | | $"Doubling\ Time\ of\ COX-2"$ (h) | $"Decay\ Switch"$ | $"Half\ Life\ of\ COX-2"$ (h) |
| | Initial Value (mM) | Final Value (mM) | Simulation time (h) | Duration (h) | | | |
| **HaCaT keratinocyte + UVR** | $1\times10^{-28}$ | $2.27\times10^{-2}$ | 0 | 3 | 3 | Off | 1 |
| **46BR.1N fibroblast +UVR** | $1\times10^{-28}$ | $2.27\times10^{-2}$ | 0 | 6 | 6 | Off | 1 |

**Supplementary Table S16. "Metabolite Ψ" scores used in the cell specific *in silico* experiments for HaCaT keratinocytes and 46BR.1N fibroblasts.** The proximity of the predicted and measured concentration of each eicosanoid in each cell type, at four time points (0.5h, 1h, 3h and 6h post stimulation), was used to calculate a quality score. The prediction accuracy was calculated based upon the percentage of model variants with a quality score above -40. In the HaCaT keratinocyte *in vitro* timecourse, prostaglandin $E_2$ ($PGE_2$), Prostaglandin $F_{2\alpha}$ ($PGF_{2\alpha}$), 12-Hydroxy-eicosatetraenoic acid (12-HETE), 15-Hydroxy-eicosatetraenoic acid (15-HETE), 15-Keto-prostaglandin $E_2$ (15-keto-$PGE_2$) and 13,14-Dihydro-15-keto-prostaglandin $E_2$ (13,14-dihydro-15-keto-$PGE_2$) were all detected so were all included in scoring against *in silico* predictions. In the 46BR.1N fibroblast *in vitro* timecourse, only $PGE_2$, 12-HETE and 15-HETE were detected so it was only possible to score these species against *in silico* predictions.

| | ***In Silico* Experiments** | | | | | | | |
|---|---|---|---|---|---|---|---|---|
| | HaCaT + A23187 | HaCaT + IND + A23187 | HaCaT + ATP | HaCaT + UVR | 46BR.1N + A23187 | 46BR.1N + IND + A23187 | 46BR.1N + ATP | 46BR.1N + UVR |
| $PGE_2$ | 91 | 2 | 64 | 0 | 4 | 0 | 7 | 0 |
| $PGF_{2\alpha}$ | 84 | 2 | 67 | 77 | - | - | - | - |
| 12-HETE | 26 | 26 | 0 | 4 | 61 | 69 | 30 | 22 |
| 15-HETE | 1 | 7 | 1 | 0 | 21 | 25 | 0 | 11 |
| 15-keto-$PGE_2$ | 13 | 0 | 0 | 0 | - | - | - | - |
| 13,14-dihydro-15-keto-$PGE_2$ | 65 | 0 | 68 | 1 | - | - | - | - |
