# Supplementary figures and images for: An adaptable in silico ensemble model of the arachidonic acid cascade
Source: Mol Omics. 2024 Jun 3;20(7):453–68. doi: 10.1039/d3mo00187c (PMC11318654; doi:10.1039/d3mo00187c)

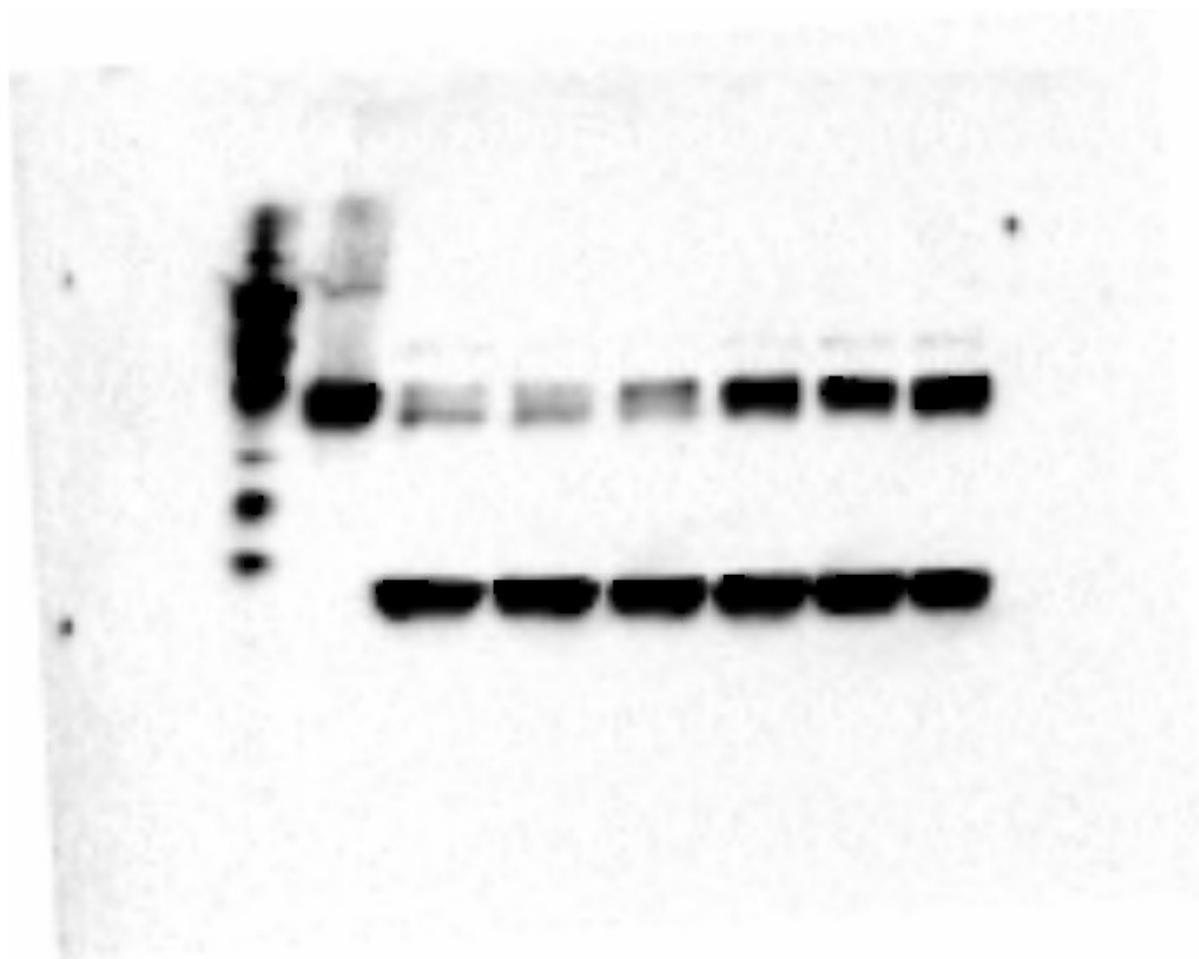

Supplement: MO-020-D3MO00187C-s003 [file MO-020-D3MO00187C-s003.pdf]

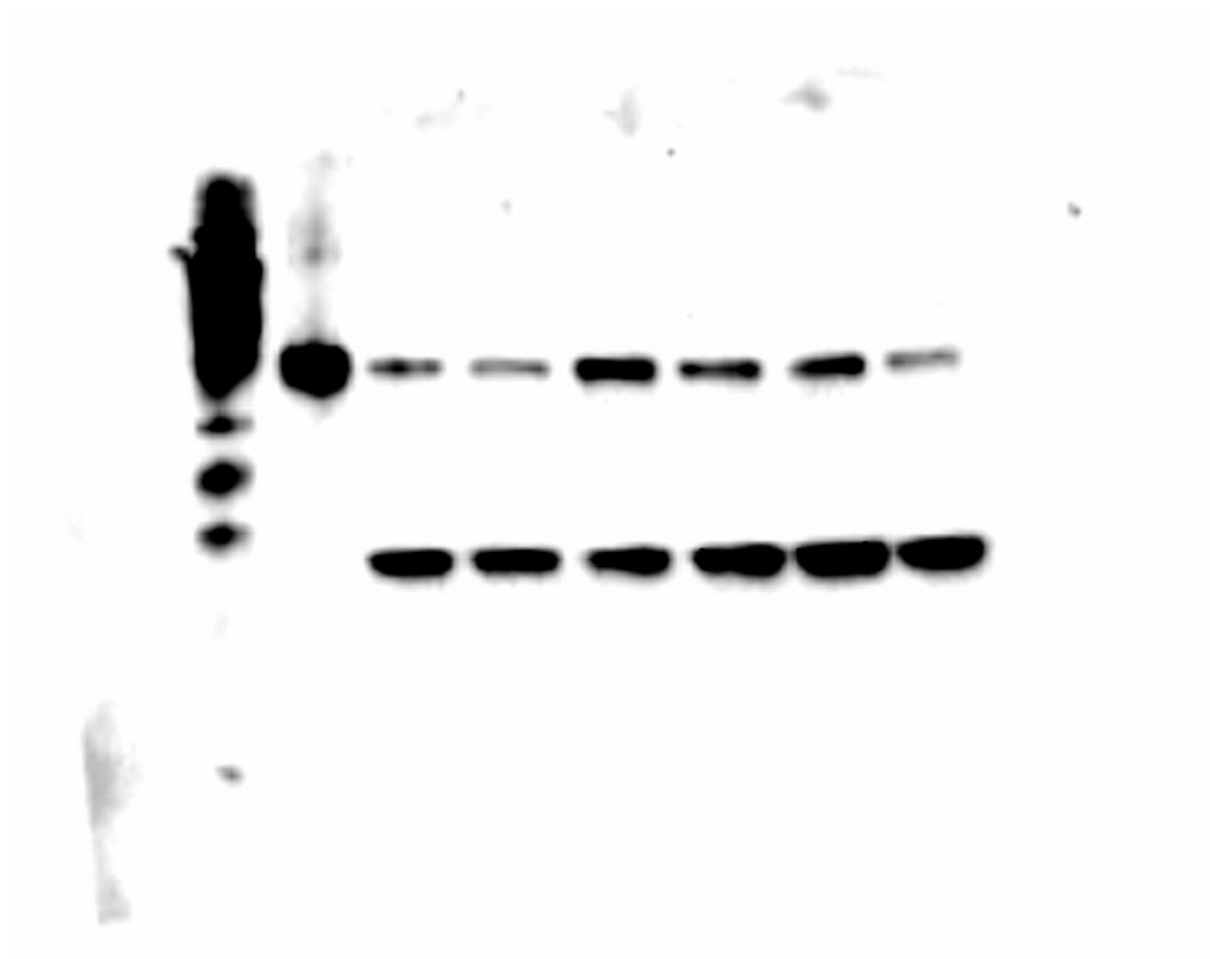

Supplement: MO-020-D3MO00187C-s004 [file MO-020-D3MO00187C-s004.pdf]
